# Ecological Interaction between Bacteriophages and Bacteria in Sub-Arctic Kongsfjorden Bay, Svalbard, Norway

**DOI:** 10.3390/microorganisms12020276

**Published:** 2024-01-28

**Authors:** Kang Eun Kim, Hyoung Min Joo, Yu Jin Kim, Donhyug Kang, Taek-Kyun Lee, Seung Won Jung, Sun-Yong Ha

**Affiliations:** 1 Library of Marine Samples, Korea Institute of Ocean Science & Technology, Geoje 53201, Republic of Korea; rkddmssl@kiost.ac.kr (K.E.K.); rladbwls06069@kiost.ac.kr (Y.J.K.); 2 Department of Ocean Science, University of Science & Technology, Daejeon 34113, Republic of Korea; tklee@kiost.ac.kr; 3 Unit of Next Generation IBRV Building Program, Korea Polar Research Institute, Incheon 21990, Republic of Korea; hmjoo77@kopri.re.kr; 4 Marine Domain & Security Research Department, Korea Institute of Ocean Science & Technology, Busan 49111, Republic of Korea; dhkang@kiost.ac.kr; 5 Risk Assessment Research Center, Korea Institute of Ocean Science & Technology, Geoje 53201, Republic of Korea; 6 Division of Polar Ocean Science Research, Korea Polar Research Institute, Incheon 21990, Republic of Korea

**Keywords:** metagenomics, bacteriophage, bacteria, Kongsfjorden Bay, sub-Arctic zone, ecological interaction

## Abstract

Marine virus diversity and their relationships with their hosts in the marine environment remain unclear. This study investigated the co-occurrence of marine DNA bacteriophages (phages) and bacteria in the sub-Arctic area of Kongsfjorden Bay in Svalbard (Norway) in April and June 2018 using metagenomics tools. Of the marine viruses identified, 48–81% were bacteriophages of the families *Myoviridae*, *Siphoviridae*, and *Podoviridae*. Puniceispirillum phage HMO-2011 was dominant (7.61%) in April, and Puniceispirillum phage HMO-2011 (3.32%) and Pelagibacter phage HTVC008M (3.28%) were dominant in June. *Gammaproteobacteria* (58%), including *Eionea flava* (14.3%) and *Pseudomonas sabulinigri* (12.2%), were dominant in April, whereas *Alphaproteobacteria* (87%), including *Sulfitobacter profundi* (51.5%) and *Loktanella acticola* (32.4%), were dominant in June. The alpha diversity of the bacteriophages and bacterial communities exhibited opposite patterns. The diversity of the bacterial community was higher in April and lower in June. Changes in water temperature and light can influence the relationship between bacteria and bacteriophages.

## 1. Introduction

Viruses, the most abundant biological entities, are estimated to exceed 10^30^ in number, and they inhabit a variety of marine ecosystems [1]. Viruses are essential components of marine microbial cycles, playing a crucial role in ecosystem functioning by supplementing dissolved organic matter [1,2,3]. Viruses are primarily classified as RNA and DNA viruses, with DNA viruses being widespread in marine environments, infecting both prokaryotes and eukaryotes [4]. New classification criteria for bacteriophages were introduced in 2023, specifically for the *Caudoviricetes* class, which includes *Autographiviridae*, *Straboviridae*, *Herelleviridae*, and *Drexlerviridae*, which are present in higher abundance than other viruses in the sea [5,6]. Bacteriophages may play a key role in regulating the bacterial community in the ocean [7] and reportedly eliminate 20–40% of the bacterial community on a daily basis [8,9]. Bacteriophages replicate using two major replication strategies, namely lysogenic and lytic replication. In lysogeny, the phage DNA integrates into the host genome, and its genetic material is replicated each time the host genome replicates. This state persists until an environmental signal induces the phage to enter the lytic pathway [10]. During the lytic cycle, host cells are lysed, and the bacteriophage progeny as well as various cellular nutrient sources are released [9].

Heterotrophic bacteria are responsible for processing a significant portion of the organic matter produced by phytoplankton. These bacteria, in turn, are consumed by predators, thereby sustaining nutrient cycling [11,12]. Owing to their ability to withstand various environmental conditions, they are ubiquitously distributed. They can thrive even in extreme conditions related to temperature, radiation, desiccation, salinity, and nutrient availability. For example, some *Pseudomonas* spp. are dominant in the Arctic and Antarctic, and the prevalence of these heterotrophic and chemoautotrophic bacteria indicates that they play a fundamental role in processes such as nitrogen fixation and nitrogen recycling via utilizing glycogen during the polar night [13,14,15]. To understand nutrient cycling, some studies have assessed the correlation between viral and prokaryotic abundance [16,17]. However, our understanding of the relationships between these elements and the broader field of viral ecology remains limited.

The coastal ecosystem of Kongsfjorden in Svalbard, Norway, is influenced by ocean currents between the Atlantic and Arctic Ocean [18]. The Kongsfjorden Sea exhibits distinctive differences from the Arctic Ocean ecosystem during the polar night when the water temperature drops below 0 °C; however, the water temperature rapidly increases after the beginning of the white nights [19]. In our previous study, we identified an ecological interplay between the eukaryotic plankton community and nucleocytoplasmic large DNA viruses in Kongsfjorden Bay in April and June 2018 [19]; NCLDVs and EPC populations were similar between the surface and bottom layers but differed between samples collected in April and June. In particular, three *Phycodnaviridae*, two *Poxviridae*, three *Pandoraviridae*, and two *Mimiviridae* viruses accounted predominantly for the NCLDV diversity. Furthermore, *Pandoraviridae* and *Mimiviridae* were strongly associated with Dinophyceae and Chlorophyta hosts, respectively. Given the wide range of viral host species, not all marine viral hosts have been defined. The study was part of a series of studies on the ecological interactions of the viral community in the Kongsfjorden marine ecosystem [19]. In this study, we aimed to (1) compare the spatial distribution between DNA phages and the bacterial community during the early white night (April) and mid-summer (June); (2) analyze changes in phage diversity in relation to changes in the bacterial community and environmental changes; and (3) identify DNA phages with a strong association and co-occurrence with specific bacteria.

## 2. Materials and Methods

### 2.1. Metaviromic Analysis of DNA Viruses

The metagenomic data of the DNA viral community were used from our previous study [19]. Detailed methods are described in the supplementary information. The bioinformatics analysis was performed in accordance with the modified protocol described by Kim et al. [19,20]. The Fastq file was trimmed with the CLC Genomics Workbench v. 20.0.4 (Qiagen, Hilden, Germany). Assembly and a quality check of viral contigs were performed using metaSPAdes v. 3.13.0 [21] and Check V (v.1.0.1) [22], respectively. Through the Check V quality check, only viral contigs of >1000 bp were retained. These viral contigs were then sorted as nucleotide identity (ANI) ≥95% using VSEARCH [23,24], and read mapping was performed with BBMap v38.51 [25] using 95% minimum alignment identity. The quality checked viral contigs were subjected to a virus taxonomy analysis using a Basic Local Alignment Search Tool (BLASTn) analysis using the Microbial Genomic Module in the CLC Genomics Workbench with the Viral RefSeq database (Release 221) of the National Center for Biotechnology Information (NCBI). Bacteriophages were sorted into dsDNA virus taxa using the modified CUTAXAC program (Customized Taxonomic Profiling Assignment Coding) developed by Kim et al. [20].

### 2.2. Metabarcoding Analyses of Bacteria

A free-living bacterial metabarcoding analysis was performed according to our previously reported methods [26]. Detailed methods are described in the supplementary information. All samples were analyzed in duplicate. To remove large-sized inorganic and organic particles, each 500 mL seawater sample was pre-filtered using a 3 μm polycarbonate filter (TSTP04700; Millipore Sigma, Bedford, MA, USA). The bacterial communities were harvested from pre-filtered seawater using a 0.2 μm polycarbonate filter (GTTP04700; Millipore Sigma, Bedford, MA, USA). The gDNA was extracted using the DNeasy Powersoil Kit (Qiagen, Hilden, Germany) and diluted to a final concentration of 20 ng μL^−1^. The first PCR was performed to amplify the V3-V4 hypervariable regions of bacterial 16S rDNA (Table S1), and the amplicons were purified using a QIAquick PCR Purification Kit (Qiagen, Hilden, Germany). The amplicons from the second PCR were purified using a Nextera XT 96 Index Kit V2 (Illumina, San Diego, CA, USA). All amplicons were pooled in equal concentrations and sequenced using the Mi-Seq platform (Illumina, San Diego, CA, USA). To analyze operational taxonomic units (OTUs), the taxonomy of the sequence with the highest similarity was assigned to the sequence read (species and genus levels with >98% and >95% similarity, respectively). CD-HIT-OTU software v.4.6.1 [27] was used for clustering and metagenomic functional information to analyze the OTUs.

### 2.3. Statistical Analysis

Among samples wherein phages and bacteria displayed a relative abundance of >0.1% in at least one sample, we selected the sample pairs with a significant positive Spearman’s correlation using SPSS v.18 (IBM Corp., Armonk, NY, USA). A circular flow chart was generated after obtaining a significantly positive Spearman correlation coefficient. The heatmap and circular chart were generated using ‘ggplot2’ in R Studio (v. 1.2.5042) [28]. A non-metric multidimensional scaling (NMDS) plots using the ranked similarity matrix were analyzed (PRIMER 6 program, Primer-E Ltd., Plymouth, UK). A clustering analysis (hierarchical agglomerative algorithm) using the group average method was performed on the most abundant OTUs. To analyze whether the sampling time and water depth affected the relationships between bacteriophages and bacteria, we conducted a permutational analysis of variance (PERMANOVA; 999 permutations) using PRIMER software version 7+ [29]. Alpha diversity, including the Simpson and Shannon indices, was analyzed using the vegan package in R Studio [30]. An extended local similarity analysis was performed using common bacteriophage and bacterial taxa [31]. P- and Q-values were calculated using permutation testing to ensure accuracy and estimate the likelihood of false positives. Network visualization was performed, and Spearman correlation coefficients of variables with *p*- and Q-values < 0.05 were visualized using Cytoscape v3.9.2 [32].

## 3. Results

**Figure 1:**
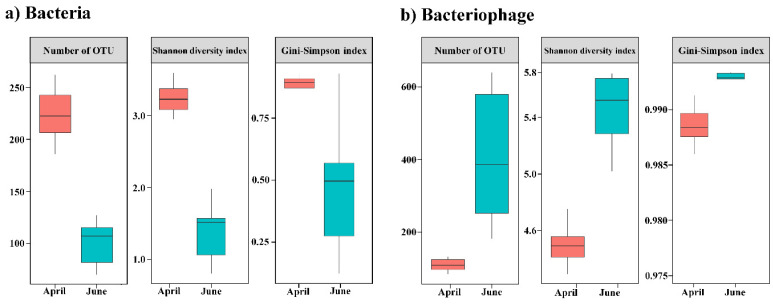
Changes in alpha diversity indices for the bacteriophage and bacterial communities in the sub-Arctic Kongsfjorden between April and June 2018. (**a**) Common bacteria and (**b**) bacteriophage operational taxonomic units (OTUs). Box plots showing alpha diversity based on the number of OTUs, Shannon diversity, and Gini–Simpson index.

The read counts are summarized in Table S2. The bacterial metabarcoding analysis generated 27,368,615 sequences and 61,037 read counts. Among the DNA viruses, 186,216 contigs were assembled, and 5996 contigs of dsDNA viruses (4077 and 1907 associated with bacteriophages and eukaryotic viruses, respectively) were assigned after quality checks using CheckV, read mapping, and taxonomic profiling (Table S3). The alpha diversity of the bacterial community was determined from the read counts based on the total number of OTUs (Figure 1). The observed mean number of OTUs in April and June was 225 and 100, respectively. The diversity indices, including the Shannon and Gini-Simpson indices, were consistent with the changes in the number of OTUs. Compared with the results obtained in April, the alpha diversity was higher in the bacteriophage community and lower in the bacterial community in June. Thus, the diversity of bacteria and bacteriophages exhibited contrasting patterns.

**Figure 2:**
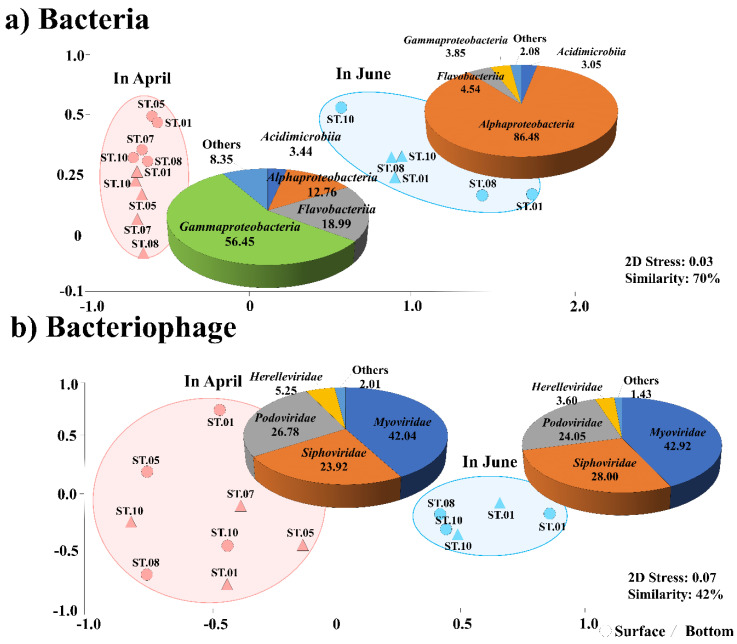
Non-metric multidimensional scaling (NMDS) plots for (**a**) the bacteria and (**b**) bacteriophage communities (**b**). Based on the results of a Bray–Curtis dissimilarity analysis, the NMDS plots were generated. All data were normalized by the square roots. The pie charts indicate the high-ranking taxonomic distribution at the family level for bacteriophage and phylum or class level for the bacterial community.

The bacterial community was classified into two groups at 70% similarity using an NMDS analysis (Figure 2). The first group showing a “dominance of Gammaproteobacteria in April”, comprised *Gammaproteobacteria* (56.5%), *Flavobacteriia* (19.0%), *Alphaproteobacteria* (12.8%), and *Acidimicrobiia* (3.4%) and was evenly distributed at most sampling sites. The other group, showing a “dominance of *Alphaproteobacteria* in June”, comprised *Alphaproteobacteria* (86.5%), *Flavobacteriia* (4.5%), *Gammaproteobacteria* (3.9%), and *Acidimicrobiia* (3.1%). Similar to the bacterial groups, the bacteriophage community was classified into two groups, April and June (42% similarity using an NMDS analysis). In April, the predominant families were *Myoviridae* (42.0%), *Siphoviridae* (24.0%), and *Podoviridae* (26.9%), while in June, the predominant families were *Myoviridae* (42.9%), *Siphoviridae* (28.0%), and *Podoviridae* (24.1%).

**Figure 3:**
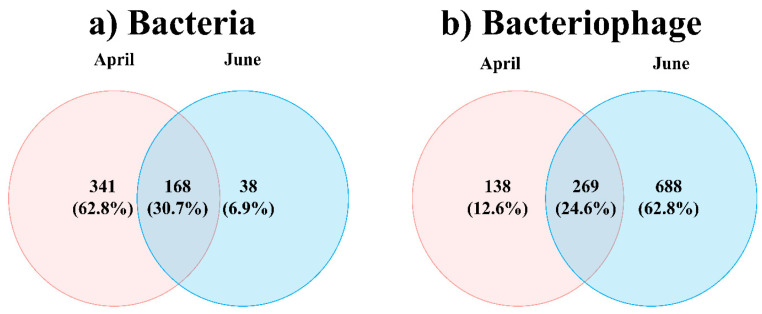
Changes in total bacterial (**a**) and bacteriophage (**b**) operational taxonomic units (OTUs) in the sub-Arctic Kongsfjorden in April and June 2018. Venn diagram showing the shared and unique total bacterial (**a**) and bacteriophage OTUs (**b**).

Consistent with the NMDS results, the PERMANOVA results indicated significant differences by month (*p* < 0.01) but not by water layer (*p* > 0.05) (Table 1). Thus, the bacteriophage and bacterial communities were divided based on sampling months but not on the basis of water depths. The Venn diagram in Figure 3 illustrates the overlap between April and June for the total bacteria and bacteriophages. The bacterial OTUs showed a 30.7% overlap (168 taxa) across the two months, whereas 62.8% (341 taxa) and 6.9% (38 taxa) represented unique bacterial OTUs in April and June, respectively. The total bacteriophage OTUs showed a 24.6% overlap (269 taxa) across the two months, whereas 12.6% (138 taxa) and 6.28% (688 taxa) were unique bacteriophage OTUs in April and June, respectively.

**Figure 4:**
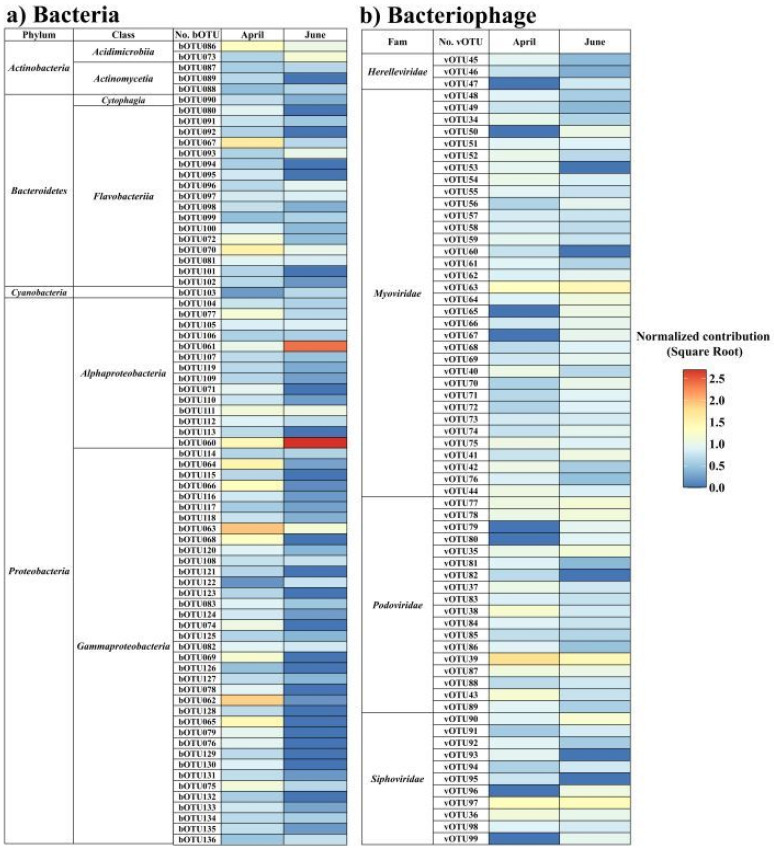
Changes in bacterial (**a**) and bacteriophage (**b**) operational taxonomic units (OTUs) in the sub-Arctic Kongsfjorden in April and June 2018. (**a**) Common bacteria OTUs (at mean relative abundances > 0.5%). (**b**) Common bacteriophage OTUs (at a mean relative abundance > 0.5%). The heatmap displays the square root normalized data.

In terms of common taxa in the bacterial OTUs (bOTU) with a relative abundance exceeding 0.5% in at least one sample, 75 and 52 taxa in April and June, respectively, were detected as common taxa (Figure 4). In April, 16 common bOTUs accounted for 72.64% of the total abundance; the dominant bOTUs were *Eionea flava* (bOTU63; 14.3%), *Pseudomonas sabulinigri* (bOTU62; 12.2%), *Lacinutrix algicola* (bOTU67; 7.3%), *Polaribacter staleyi* (bOTU70; 5.3%), and *Cognaticolwellia aestuarii* (bOTU64; 5.1%). In June, seven common bOTUs, including *Sulfitobacter* profundi (bOTU60; 51.5%) and *Loktanella acticola* (bOTU61; 32.4%), accounted for 91.2% of the total abundance. In the bacteriophage community, 58 (April) and 61 (June) virus OTUs (vOTUs) were detected at a relative abundance of over 0.5% in at least one sample (Figure 4). In April, eight bacteriophages, including Puniceispirillum phage HMO-2011 (vOTU39; 7.6%), Nonlabens phage P12024L (vOTU97; 2.5%), and Pelagibacter phage HTVC008M (vOTU63; 2.41%), accounted for 19.1% of the total relative abundance. In June, nine taxa, including Pelagibacter phage HTVC008M (vOTU63; 3.2%), Puniceispirillum phage HMO-2011 (vOTU39; 3.2%), and Nonlabens phage P12024L (vOTU97; 2.7%), accounted for 16.9% of the relative abundance. Thus, *Puniceispirillum*, *Pelagibacter*, and *Vibrio* phages were more abundant in April than in June, whereas Cellulophaga and cyanophages were more abundant in June. In particular, cyanophage, including *Synechococcus* phage and *Prochlorococcus* phage, exhibited a rapid increase in their abundance, reaching 22.14% in June, more than twice that observed in April.

**Figure 5:**
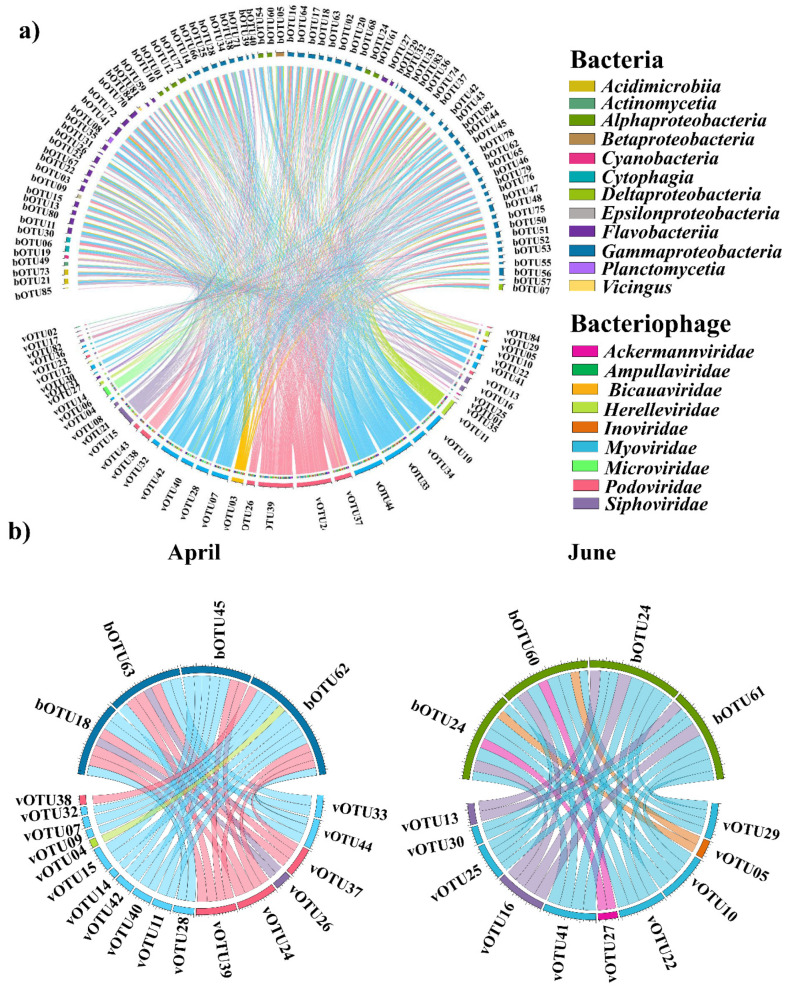
Associations between the bacterial and bacteriophage communities in the Sub-Arctic Kongsfjorden. (**a**) Correlation with total data. (**b**) Correlation with each month. Significant pairwise comparisons of the Spearman correlation coefficients between bacteria and bacteriophages. Detailed information (species names of operational taxonomic unit (OTU) numbers and correlation coefficients) are listed in Table S5.

Spearman’s correlation analyses were performed to assess the significance of the associations between the common bacteriophages and bacterial OTUs. Based on significant correlation coefficients, 24 bOTUs were correlated with 11 vOTUs (Table S4). The predominant bacterial taxa for each month correlated with certain bacteriophages (Figure 5). Specifically, *Eionea flava* (bOTU063), the predominant taxon in April, was significantly correlated with two *Podoviridae* taxa (Puniceispirillum phage HMO-2011, vOTU39, and Pelagibacter phage HTVC019P, vOTU38) and one *Myoviridae* (Yersinia phage fHe-Yen9-0, vOTU44). *Pseudomonas sabulinigri* (bOTU062) was significantly correlated with three *Myoviridae* (Sphingomonas phage PAU, vOTU40; Synechococcus phage S-WAM2, vOTU42; and Yersinia phage fHe-Yen9-04, vOTU44) and two *Podoviridae* OTUs (Pelagibacter phage HTVC019P, vOTU38, and Puniceispirillum phage HMO-2011, vOTU39). In addition, *Sulfitobacter profundi* (bOTU060) and *Loktanella acticola* (bOTU061; the predominant taxa in June) were significantly correlated with *Myoviridae* (Synechococcus phage S-WAM7, vOTU41).

**Figure 6:**
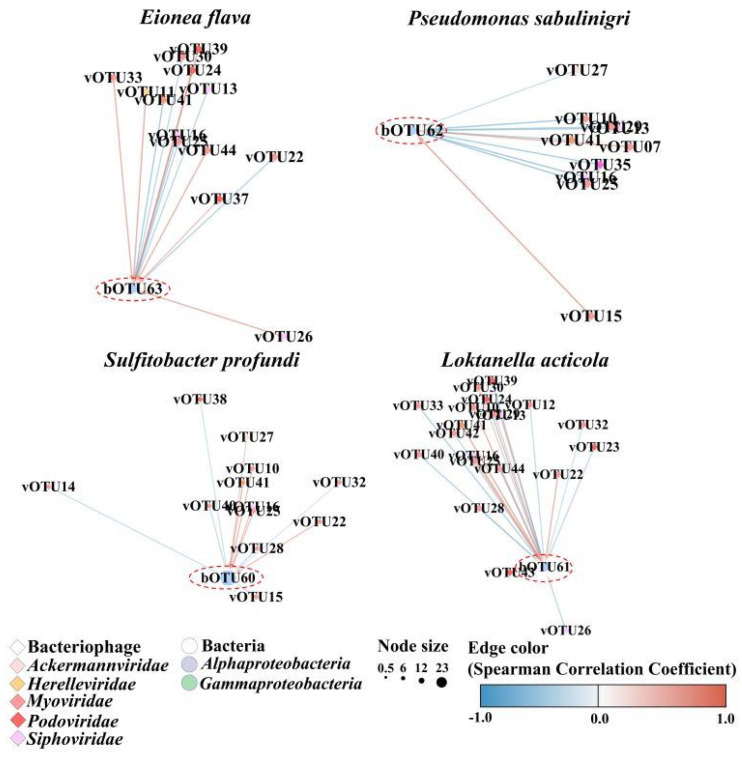
Network analysis showing co-occurrence between dominant bacteriophages and bacterial community across April and June 2018 represented as blue and beige nodes, respectively. Lines between nodes indicate positive (red) and negative (blue) Spearman’s coefficient of correlations (SCC) > |0.3| (two-sided pseudo-*p*-value < 0.05) between the abundances of linked taxa. Detailed information (species names of operational taxonomic unit (OTU) numbers and correlation coefficients) are listed in Table S5.

A network analysis of the common bacterial and bacteriophage taxa revealed specific associated co-occurrences. The network comprised 43 nodes and 80 edges, indicating significant co-occurrence between bacteriophages and bacterial communities (Figure S1, Table S6). The relationship between the predominant bacterial species and bacteriophage species was compared for each month (Figure 6). The common bOTUs correlated with at least one vOTU. Nine phage groups (family levels), *Ackermannviridae*, *Ampullaviridae*, *Bicaudaviridae*, *Herelleviridae*, *Inoviridae*, *Microviridae*, *Myoviridae*, *Siphoviridae*, and *Podoviridae*, co-occurred with eleven bacterial classes, comprising *Acidimicrobiia*, *Actinomycetia*, *Alphaproteobacteria*, *Betaproteobacteria*, *Cyanobacteriota*, *Cytophagia*, *Deltaproteobacteria*, *Epsilonproteobacteria*, *Flavobacteriia*, *Gammaproteobacteria*, and *Planctomycetia*. More specifically, the most common bacterial taxa in April, *Eionea flava* (bOTU63) and *Pseudomonas sabulinigri* (bOTU62), co-occurred with three *Podoviridae* OTUs (Pelagibacter phage HTVC010P, vOTU37; Puniceispirillum phage HMO-2011, vOTU39; and Cellulophaga phage phi38:1, vOTU35) and two *Myoviridae* OTUs (Synechococcus phage S-SSM7, vOTU41, and Yersinia phage fHe-Yen9-04, vOTU44). The most common bacterial taxa in June, *Sulfitobacter profundi* (bOTU60) and *Loktanella acticola* (bOTU67), exhibited co-occurrence with four *Myoviridae* OTUs (Phingomonas phage PAU, vOTU40; Synechococcus phage S-SSM7, vOTU41; Synechococcus phage S-WAM2, vOTU42; and Yersinia phage fHe-Yen9-04, vOTU44) and three *Podoviridae* (Puniceispirillum phage HMO-2011, vOTU39; Vibrio phage CHOED, vOTU43; and Pelagibacter phage HTVC019P, vOTU38).

## 4. Discussion

In our previous study [19], we reported environmental changes and co-variance between eukaryotic plankton and nucleocytoplasmic large DNA virus (NCLDV) communities in Kongsfjorden Bay. Specifically, we revealed that NCLDVs affect phytoplankton structure due to rapid environmental changes in early white nights and mid-summer in the sub-Arctic zone [19]. One of the most important results of the present study was the high bacterial diversity in April under extreme environmental conditions, with air and water temperatures being below −15 °C and 0 °C, respectively. Wietz et al. [14] reported an increase in the abundance of diverse bacteria in the Arctic region at the start of April. Consistently, in the present study, bacterial assemblages and diversity increased in June with the rapid increase in light intensity and organic particles such as phytoplankton. This change in bacterial assemblages was also consistent with the results of other previous studies [33,34]. Notably, the diversity of bacteria and bacteriophages exhibited opposing trends. The patterns in bacteriophage communities can be used to ascertain the lysogenic and lytic replication modes; lysogeny favors lower microbial abundance or activity, which is hypothesized as the key mechanism ensuring host survival in oligotrophic habitats and harsh environments with low viral lysis rates [35].

Viral proliferation is suppressed when photosynthesis is not active and the seawater temperature is below 0 °C [19]. When the Arctic marine environment transitions from oligotrophic and lower water temperatures in April to mesotrophic conditions and higher water temperatures in June, viruses change their replication mode from lysogenic to lytic [35,36]. Other than the lysis–lysogeny switch, changes in environmental factors (e.g., temperature and dissolved organic matter) can also directly alter viral and bacterial diversity. This transition results in organic matter release through host cell lysis, leading to increased viral diversity and decreased bacterial diversity in June. Similarly, Yau and Seth-Pasricha [37] reported that viral abundance increases during light intensity owing to changes in the water temperature and salinity of the surface ecosystem of Svalbard. Moreover, the viral shunt pathway [38] diverts microbial biomass from secondary consumers, such as plankton and fish, into the pool of dissolved organic matter that is primarily consumed by heterotrophic bacteria. These findings highlight the unique phenomenon of low viral and high bacterial diversity in extreme environments, such as early white nights and low temperatures, which significantly further our understanding of Arctic ecosystems. Notably, lysogeny was not detected in the Arctic freshwater environment during the summer, suggesting that the lytic and lysogenic pathways are strongly influenced by the environment and season [39]. Furthermore, an annual study on viral life cycles conducted in Antarctica exploring seasonal changes revealed a high incidence of lysogenic viral replication in winter and an opposing pattern in summer. Despite numerous proposed explanations for these observed patterns, a conclusive inference has not been reached [40,41,42,43,44].

In the present study, bacteria belonging to the *Alphaproteobacteria, Gammaproteobacteria*, and *Bacteroidota* families comprised a substantial proportion of the Kongsfjorden ecosystem. In a study that was part of the Tara Ocean project, *Alphaproteobacteria*, *Gammaproteobacteria*, *Bacteroidetes*, and *Actinobacteria* were prevalent in oceans worldwide, including the polar seas [45]. Furthermore, Cao et al. [45] emphasized that the metagenomes obtained from polar seawater were nearly undetectable in temperate seawater, as the environmental conditions of the Arctic and Antarctic are more similar to each other than to the temperate regions.

We noted that *Eionea flava* (family: *Cellvibrionaceae*) dominated in April, and to the best of our knowledge, this is the first study to report that *Eionea nigra* is dominant in the northern polar region. The genus *Eionea*, first described by Urios et al. [46], produces ice-binding proteins that aid survival in freezing environments by inhibiting ice recrystallization [47]. Thus, *Eionea* may grow well under the extremely low temperature conditions prevalent in April. In the present study, *Sulfitobacter profundi* (*Alphaproteobacteria*) was the predominant bacterial taxon detected in June. Although this bacterium is globally distributed [48] and frequently appears in polar regions [49], Nguyen et al. [50] reported that *Sulfitobacter profundi* is an opportunistic species and can also occur in oligotrophic environments. Moreover, *Sulfitobacter pontiacus* (*Alphaproteobacteria*) and *Pseudoalteromonas* sp. (*Gammaproteobacteria*) are frequently observed in the polar regions during phytoplankton blooms [15,51,52]. The abundance of *Aureococcus anophagefferens*, a nanosized eukaryotic phytoplankton, rapidly increased in June 2018 [19]. Similar to the *A. anophagefferens* bloom, the abundance of *Sulfitobacter* significantly increased in June, possibly attributed to phytoplankton bloom-induced nutrient release (either due to phytoplankton death or the production of extracellular polymeric substances released by phytoplankton cells) [53,54].

In the present study, *Myoviridae*, *Podoviridae*, *Siphoviridae*, and *Herelleviridae* were the most common bacteriophages identified, consistent with their common occurrence in oceans [55]. The presence of Pelagibacter phage HTVC008M and Puniceispirillum phage HMO-2011, including the Pelagibacter phage group, suggests that the SAR11 bacterial group is abundant in the Arctic Ocean [56]. The co-occurrence of various phages with various bacteria indicates that phages may be capable of infecting multiple host bacterial ecotypes in warm- and cold-water environments [57]. In this study, Puniceispirillum phage HMO-2011, Pelagibacter phage HTBC010P, Puniceispirillum phage HMO-2011, Vibrio phage CHOED, and Roseobacter virus SIO1 were strongly associated with *Sulfitobacter* and *Loktanella.* Qin et al. [58] reported that Puniceispirillum phage HMO-2011 is a major regulator of bacterial infection within the SAR11 (*Pelagibacterales*) clade. In addition, Du et al. [59] reported the worldwide distribution of Pelagibacter phage HTBC010P. Closely related Pelagiphages are postulated to have evolved to exhibit great adaptability to a wider range of hosts [60]. In the present study, the relative abundance of cyanobacteria-killing phages, such as cyanophages, *Prochlorococcus* phage, and *Synechococcus* phage, increased in June, concomitant with an increase in cyanobacterial abundance. Cyanophages are abundant in ocean ecosystems and play a crucial role in biogeochemical cycles, including growth regulation and the photosynthesis of cyanobacteria [61]. Specifically, *Prochlorococcus* phage and *Synechococcus* phage increase markedly in polar regions [56,62,63].

## 5. Conclusions

The present study highlights the changes in diversity between bacteriophages and bacterial communities during April and June, correlating with environmental changes in the sub-Arctic Kongsfjorden marine ecosystem. *Myoviridae*, *Podoviridae*, and *Siphoviridae* accounted for a considerable proportion of bacteriophages, while *Eionea flava*, *Pseudomonas sabulinigri*, *Sulfitobacter profundi*, and *Loktanella acticola* dominated the bacterial community. Specifically, our findings revealed differences in the community compositions of bacteria and bacteriophages, which were also correlated, suggesting that bacteriophages control the host community via their replication mode. We also identified the co-occurrence of various bacteriophages with a ubiquitous host and a correlation between single bacteriophages and multiple hosts. In June, the number of cyanophages increased rapidly, coinciding with an increase in the number of cyanobacteria. Moreover, rapid changes in the environment during the polar night and white night were associated with rapid changes in eukaryotic plankton in the Arctic ecosystem, subsequent bacterial changes, and, ultimately, the bacterial control mechanism of bacteriophages. Therefore, our results not only provide new insights into the important ecological relationships between the bacteriophage and bacterial communities, but they are particularly relevant given the expected impact of bacteriophages on the sub-Arctic Kongsfjorden ecosystem and will be useful in better understanding this ecosystem.

## Figures and Tables

**Table 1:** Changes in community composition by season and water layer based on PERMANOVA analysis.

Group	Source	*T*	*p* (perm)
Bacteria	Surface × Bottom	0.225	0.452
	April × June	5.288	0.002
Bacteriophage	Surface × Bottom	0.869	0.605
	April × June	2.598	0.002

## Data Availability

The datasets presented in this study can be found in online repositories. The names of the repository/repositories and accession numbers can be found below. https://www.ncbi.nlm.nih.gov/genbank/, PRJNA848283 and PRJNA999943 (accessed on 12 June 2022 and 29 July 2023, respectively).

## Supplementary Materials

The following supporting information can be downloaded at https://www.mdpi.com/article/10.3390/microorganisms12020276/s1, Figure S1. Network analysis showing the co-occurrence between common bacteriophages and bacteria, represented as blue and beige nodes, respectively; Table S1. Experimental information for the amplification of the V3–V4 regions in 16s rDNA; Table S2. Summary of the total bases, reads, GC (%), Q20 (%), and Q30 (%) obtained from metagenomic next-generation sequencing analysis; Table S3. Information on quality check of metavirome contigs using Check V; Table S4. Information on species names of operational taxonomic unit numbers and significant correlation coefficients between bacterial and bacteriophage lineages; Table S5. Classification information on the operational taxonomic units (OTUs) of common bacteria and bacteriophages; and Table S6. The significant results of local similarity correlations (LSA) in the network analysis in Figure 6 [19,20,21,22,23,24,25,26,27,64,65,66,67,68].
