# Supplementary material for: Ecological Interaction between Bacteriophages and Bacteria in Sub-Arctic Kongsfjorden Bay, Svalbard, Norway
Source: Microorganisms. 2024 Jan 28;12(2):276. doi: 10.3390/microorganisms12020276 (PMC10893223; doi:10.3390/microorganisms12020276)
Supplement: Supplementary file 1 [file microorganisms-12-00276-s001.zip › microorganisms-2825695-supplementary.pdf]

Supplementary information

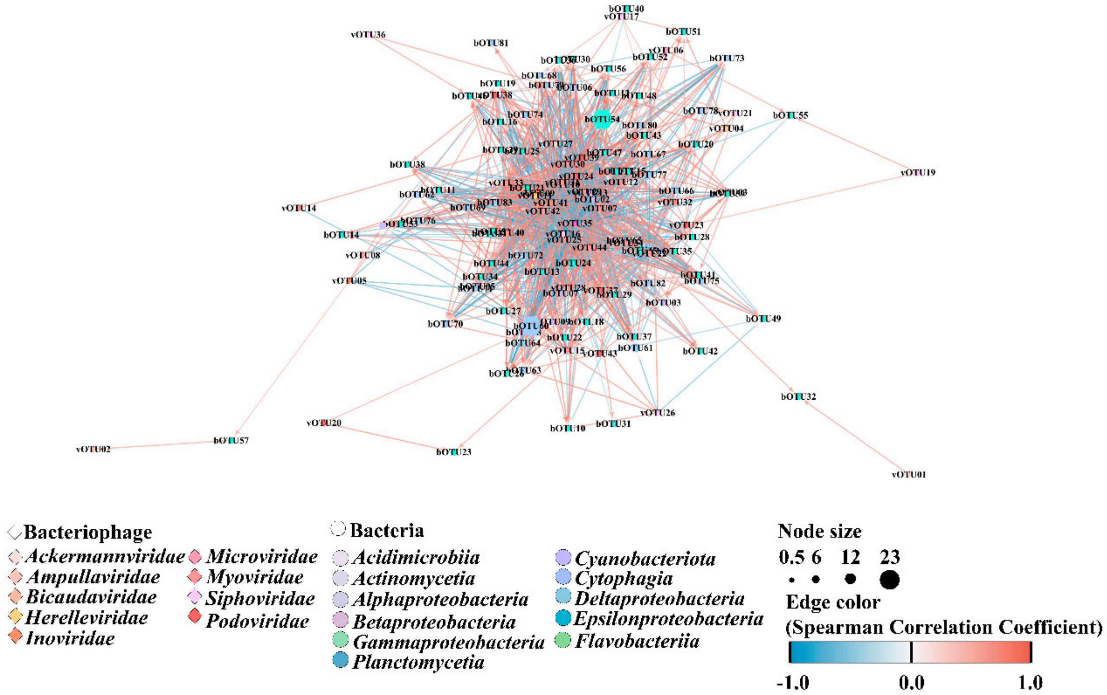

Figure S1. Network analysis showing the co-occurrence between common bacteriophages and bacteria, represented as blue and beige nodes, respectively. Lines between nodes indicate positive (red) and negative (blue) Spearman's correlation coefficient (SCC) correlation > |0.3| (two -sided pseudo P-value < 0.05) between the abundance of linked taxa. Detailed information (species names of operational taxonomic unit [OTU] numbers and correlation coefficients) are described in Table S5.

Table S1. Experimental information for amplification of V3-V4 regions in 16s rDNA.

Target region	Step	Primer		PCR reaction mixtures (25-µL)	PCR condition	
		Forward	Reverse			
16s rDNA (V3-V4 region)	First PCR	Ilumina preadapter+Sequencing primer sequence+341F (5'-CCTACGGGN GGCWGCAG-3′)	Ilumina preadapter +Sequencing primer sequence+800R (5'-TACCAGGGT ATCTAATCC-3′)	200 µmol L^{-1} each dNTP, 1.5 mmol L^{-1} MgCl2, 0.3 µmol L^{-1} each primer, 2.5 U Taq DNA polymerase (TaKaRa, EX Taq, Kyoto, Japan), and DNA template (20 ng μL^{-1})	initial denaturation step at 95°C for 3 min, followed by 35 cycles of denaturation at 95°C for 10 s, annealing at 52°C for 45 s, and extension at 72°C for 1 min, and a final extension step of 72°C for 5 min.	[9]
	Second PCR	Ilumina preadapter +Sequencing primer sequence+341F (5'-CCTACGGGN GGCWGCAG-3')	Ilumina preadapter +Sequencing primer sequence+800R (5'-TACCAGGGT ATCTAATCC-3′)	200 µmol L^{-1} each dNTP, 1.5 mmol L^{-1} MgCl2, 0.3 µmol L^{-1} each primer, 2.5 U Taq DNA polymerase (TaKaRa, EX Taq, Kyoto, Japan), and DNA template (800 ng), and Nextera XT index Kit (Illumina, CA, USA)	initial denaturation step at 95°C for 3 min, followed by 12 cycles of denaturation at 95°C for 10 s, annealing at 52°C for 45 s, and extension at 72°C for 1 min, and a final extension step of 72°C for 5 min.	
	Pooling	Triplicate reaction products (same amount) were pooled and purified using an Qiaquick PCR purification Kit (No. 28104, Qiagen Inc.). Their DNA concentration was measured in a Bio-analyzer 2100 (Agilent Technologies, Palo Alto, CA, USA).				

Table S2. Summary of the total bases, reads, GC (%), Q20 (%) and Q30 (%) obtained from metagenomic next-generation sequencing analysis.

Month	Bacteria							Bacteriophage				
	No. Site	Water depth	Total Bases	Reads	GC (%)	Q20 (%)	Q30 (%)	Total Bases	Reads	GC (%)	Q20 (%)	Q30 (%)
April	St.1	Surface	40,843,703	91,801	51.27	98.21	94.01	18,359,656	40,235	52.20	98.07	93.66
		Bottom	36,550,370	82,887	50.85	97.80	92.84	14,596,720	32,092	52.30	97.84	92.94
	St.5	Surface	19,445,775	42,596	52.59	98.23	94.07	14,810,612	32,679	52.17	98.08	93.65
		Bottom	18,374,303	40,300	52.10	98.19	93.96	13,457,881	29,601	50.65	98.03	93.49
	St.7	Surface	24,882,126	54,372	51.92	98.30	94.27	**No data**				
		Bottom	22,330,844	48,775	52.61	98.22	94.03	15,951,805	34,928	51.76	98.17	93.84
	St.8	Surface	29,670,795	65,008	52.47	98.24	94.13	26,493,985	58,156	51.73	98.06	93.63
		Bottom	28,318,361	61,722	52.34	98.15	93.85	**No data**				
	St.10	Surface	31,420,464	68,918	52.27	98.22	94.03	25,970,249	56,914	50.62	98.05	93.63
		Bottom	30,066,251	66,133	52.48	98.17	93.90	21,035,174	46,306	51.62	98.01	93.51
June	St.1	Surface	19,156,361	43,760	52.28	99.22	97.06	7,142,018	16,285	52.38	99.11	96.72
		Bottom	14,761,903	33,451	51.80	99.01	96.49	1,013,801	2,292	51.94	98.78	95.95
	St.8	Surface	35,018,581	79,698	51.95	99.17	96.90	30,322,041	68,585	51.39	99.08	96.73
		Bottom	29,355,631	66,668	51.89	99.08	96.68	**No data**				
	St.10	Surface	26,413,183	59,323	51.65	99.11	96.74	1,997,498	4,547	52.81	99.09	96.68
		Bottom	29,188,186	65,241	51.23	98.99	96.37	28,764,524	63,056	51.22	98.77	95.75

Table S3. Information of quality check of metavirome contigs using Check V.

contig_id	contig_length	provirus	gene_count	viral_genes	checkv_quality	completeness	completeness_method
NODE_1_length_43046_cov_0.587189	43046	No	43	11	Medium-quality	70.98	AAI-based (high-confidence)
NODE_106_length_3128_cov_0.136018	3128	No	1	1	Medium-quality	58.05	AAI-based (high-confidence)
NODE_108_length_3120_cov_0.099305	3120	No	1	1	Medium-quality	68.16	AAI-based (high-confidence)
NODE_108_length_3599_cov_0.200571	3599	No	1	1	Medium-quality	70.6	AAI-based (high-confidence)
NODE_109_length_3114_cov_0.147595	3114	No	1	1	Medium-quality	55.17	AAI-based (high-confidence)
NODE_109_length_3585_cov_0.196213	3585	No	1	1	Medium-quality	76.92	AAI-based (high-confidence)
NODE_112_length_3077_cov_0.085964	3077	No	1	1	Medium-quality	57.1	AAI-based (high-confidence)
NODE_113_length_3336_cov_0.151066	3336	No	1	1	Medium-quality	68.88	AAI-based (high-confidence)
NODE_117_length_2950_cov_0.267275	2950	No	1	1	Medium-quality	63.91	AAI-based (high-confidence)
NODE_119_length_15803_cov_0.302407	15803	No	29	12	Medium-quality	50.49	AAI-based (high-confidence)
NODE_12_length_5828_cov_0.134055	5828	No	2	1	Medium-quality	86.53	AAI-based (high-confidence)
NODE_125_length_23794_cov_0.589913	23794	No	36	11	Medium-quality	75.71	AAI-based (high-confidence)
NODE_129_length_3208_cov_0.121904	3208	No	1	1	Medium-quality	58.5	AAI-based (high-confidence)
NODE_130_length_2832_cov_0.131357	2832	No	1	1	Medium-quality	60.34	AAI-based (high-confidence)
NODE_130_length_2917_cov_0.158623	2917	No	1	1	Medium-quality	52.36	AAI-based (high-confidence)
NODE_134_length_3289_cov_0.216301	3289	No	1	1	Medium-quality	71.44	AAI-based (high-confidence)
NODE_155_length_3200_cov_0.177685	3200	No	3	1	Medium-quality	51.05	AAI-based (high-confidence)
NODE_159_length_25030_cov_0.171473	25030	No	30	14	Medium-quality	60.18	AAI-based (high-confidence)
NODE_16_length_6001_cov_0.222467	6001	No	2	1	Medium-quality	82.3	AAI-based (high-confidence)
NODE_163_length_2976_cov_0.282586	2976	No	1	1	Medium-quality	65.54	AAI-based (high-confidence)
NODE_165_length_2704_cov_0.144722	2704	No	1	1	Medium-quality	57.71	AAI-based (high-confidence)
NODE_166_length_24211_cov_0.472752	24211	No	35	9	Medium-quality	70.51	AAI-based (high-confidence)
NODE_166_length_2684_cov_0.171373	2684	No	1	1	Medium-quality	56.01	AAI-based (high-confidence)
NODE_17_length_5613_cov_0.136017	5613	No	3	1	Medium-quality	88.74	AAI-based (high-confidence)
NODE_17_length_6586_cov_0.176199	6586	No	3	1	Medium-quality	86.9	AAI-based (high-confidence)
NODE_179_length_3009_cov_0.158763	3009	No	2	1	Medium-quality	65.27	AAI-based (high-confidence)
NODE_18_length_5602_cov_0.169726	5602	No	3	1	Medium-quality	86.83	AAI-based (high-confidence)
NODE_2_length_30332_cov_0.443324	30332	No	22	8	Medium-quality	50.01	AAI-based (high-confidence)
NODE_2_length_30507_cov_0.522954	30507	No	34	11	Medium-quality	50.22	AAI-based (high-confidence)
NODE_2_length_32084_cov_0.659903	32084	No	37	10	Medium-quality	52.82	AAI-based (high-confidence)
NODE_224_length_2668_cov_0.097703	2668	No	2	1	Medium-quality	51.61	AAI-based (high-confidence)
NODE_23_length_4779_cov_0.144872	4779	No	3	1	Medium-quality	73.2	AAI-based (high-confidence)
NODE_231_length_2741_cov_0.165026	2741	No	1	1	Medium-quality	58.91	AAI-based (high-confidence)
NODE_24_length_4751_cov_0.211092	4751	No	2	1	Medium-quality	85.65	AAI-based (high-confidence)
NODE_249_length_2564_cov_0.171197	2564	No	1	1	Medium-quality	55.12	AAI-based (high-confidence)
NODE_25_length_4511_cov_0.127833	4511	No	1	1	Medium-quality	69.61	AAI-based (high-confidence)
NODE_25_length_4736_cov_0.248868	4736	No	2	1	Medium-quality	65.55	AAI-based (high-confidence)
NODE_267_length_2606_cov_0.160351	2606	No	2	1	Medium-quality	56.61	AAI-based (high-confidence)
NODE_2686_length_4630_cov_0.037519	4630	No	1	1	Medium-quality	85.95	AAI-based (high-confidence)
NODE_28_length_4634_cov_0.166042	4634	No	2	1	Medium-quality	71.54	AAI-based (high-confidence)
NODE_288_length_2558_cov_0.133794	2558	No	2	1	Medium-quality	54.99	AAI-based (high-confidence)
NODE_3_length_20417_cov_0.218082	20417	No	27	16	Medium-quality	59.08	AAI-based (high-confidence)
NODE_3_length_22677_cov_0.312649	22677	No	29	13	Medium-quality	63.06	AAI-based (high-confidence)
NODE_3_length_25350_cov_0.308423	25350	No	32	19	Medium-quality	71.13	AAI-based (high-confidence)

NODE_3_length_26876_cov_0.251634	26876	No	38	18	Medium-quality	76.6	AAI-based (high-confidence)
NODE_31_length_4529_cov_0.186456	4529	No	1	1	Medium-quality	81.34	AAI-based (high-confidence)
NODE_318_length_2552_cov_0.112515	2552	No	1	1	Medium-quality	55.04	AAI-based (high-confidence)
NODE_32_length_4258_cov_0.156288	4258	No	1	1	Medium-quality	66.12	AAI-based (high-confidence)
NODE_33_length_4903_cov_0.289967	4903	No	1	1	Medium-quality	51.47	AAI-based (high-confidence)
NODE_35_length_4369_cov_0.221311	4369	No	2	1	Medium-quality	77.72	AAI-based (high-confidence)
NODE_36_length_4248_cov_0.205833	4248	No	1	1	Medium-quality	75.43	AAI-based (high-confidence)
NODE_37_length_4270_cov_0.137377	4270	No	1	1	Medium-quality	61.31	AAI-based (high-confidence)
NODE_38_length_4303_cov_0.178164	4303	No	1	1	Medium-quality	88.83	AAI-based (high-confidence)
NODE_39_length_4229_cov_0.243584	4229	No	1	1	Medium-quality	63.5	AAI-based (high-confidence)
NODE_39_length_4247_cov_0.160077	4247	No	1	1	Medium-quality	65.68	AAI-based (high-confidence)
NODE_4_length_21733_cov_0.178238	21733	No	33	14	Medium-quality	59.2	AAI-based (high-confidence)
NODE_40_length_29436_cov_0.359682	29436	No	41	10	Medium-quality	86.21	AAI-based (high-confidence)
NODE_40_length_4040_cov_0.151484	4040	No	2	1	Medium-quality	72.15	AAI-based (high-confidence)
NODE_40_length_4210_cov_0.111895	4210	No	2	1	Medium-quality	57.96	AAI-based (high-confidence)
NODE_41_length_4055_cov_0.175177	4055	No	1	1	Medium-quality	60.68	AAI-based (high-confidence)
NODE_42_length_4000_cov_0.259164	4000	No	1	1	Medium-quality	59	AAI-based (high-confidence)
NODE_42_length_4232_cov_0.181708	4232	No	1	1	Medium-quality	82.97	AAI-based (high-confidence)
NODE_43_length_4200_cov_0.157279	4200	No	2	1	Medium-quality	61.68	AAI-based (high-confidence)
NODE_449_length_4008_cov_0.118189	4008	No	1	1	Medium-quality	54.13	AAI-based (high-confidence)
NODE_45_length_27244_cov_0.224351	27244	No	37	17	Medium-quality	74.44	AAI-based (high-confidence)
NODE_45_length_29726_cov_0.369224	29726	No	39	9	Medium-quality	86.74	AAI-based (high-confidence)
NODE_46_length_4156_cov_0.219127	4156	No	3	1	Medium-quality	63.35	AAI-based (high-confidence)
NODE_46_length_4509_cov_0.138776	4509	No	4	1	Medium-quality	68.12	AAI-based (high-confidence)
NODE_47_length_4076_cov_0.132260	4076	No	3	1	Medium-quality	89.8	AAI-based (high-confidence)
NODE_47_length_4392_cov_0.120196	4392	No	2	1	Medium-quality	64.12	AAI-based (high-confidence)
NODE_48_length_4043_cov_0.196755	4043	No	2	1	Medium-quality	59.5	AAI-based (high-confidence)
NODE_52_length_23787_cov_0.206687	23787	No	41	16	Medium-quality	75.33	AAI-based (high-confidence)
NODE_52_length_4321_cov_0.196116	4321	No	1	1	Medium-quality	77.9	AAI-based (high-confidence)
NODE_53_length_27925_cov_0.245849	27925	No	44	20	Medium-quality	76.77	AAI-based (high-confidence)
NODE_53_length_4335_cov_0.202314	4335	No	2	1	Medium-quality	70.5	AAI-based (high-confidence)
NODE_54_length_4312_cov_0.267743	4312	No	1	1	Medium-quality	59.99	AAI-based (high-confidence)
NODE_56_length_3850_cov_0.255399	3850	No	2	2	Medium-quality	85.56	AAI-based (high-confidence)
NODE_56_length_3920_cov_0.147082	3920	No	2	1	Medium-quality	86.75	AAI-based (high-confidence)
NODE_56_length_4304_cov_0.121522	4304	No	3	1	Medium-quality	64.65	AAI-based (high-confidence)
NODE_564_length_3635_cov_0.061652	3635	No	1	1	Medium-quality	68.17	AAI-based (high-confidence)
NODE_58_length_3116_cov_0.008286	3116	No	4	1	Medium-quality	50.25	AAI-based (high-confidence)
NODE_58_length_3830_cov_0.172608	3830	No	1	1	Medium-quality	56.96	AAI-based (high-confidence)
NODE_58_length_4223_cov_0.117119	4223	No	4	1	Medium-quality	77	AAI-based (high-confidence)
NODE_583_length_2273_cov_0.106256	2273	No	2	1	Medium-quality	50.75	AAI-based (high-confidence)
NODE_60_length_3696_cov_0.147901	3696	No	2	1	Medium-quality	55.15	AAI-based (high-confidence)
NODE_60_length_4212_cov_0.127158	4212	No	1	1	Medium-quality	89.75	AAI-based (high-confidence)
NODE_61_length_3580_cov_0.137030	3580	No	2	1	Medium-quality	52.49	AAI-based (high-confidence)
NODE_63_length_32555_cov_0.417550	32555	No	42	20	Medium-quality	72.07	AAI-based (high-confidence)
NODE_64_length_4164_cov_0.181796	4164	No	2	1	Medium-quality	64.84	AAI-based (high-confidence)
NODE_66_length_4113_cov_0.117090	4113	No	1	1	Medium-quality	83.27	AAI-based (high-confidence)

NODE_67_length_3835_cov_0.163544	3835	No	1	1	Medium-quality	57.71	AAI-based (high-confidence)
NODE_68_length_3819_cov_0.142742	3819	No	3	1	Medium-quality	82.03	AAI-based (high-confidence)
NODE_71_length_4019_cov_0.193622	4019	No	1	1	Medium-quality	60.19	AAI-based (high-confidence)
NODE_72_length_3432_cov_0.254125	3432	No	1	1	Medium-quality	75.32	AAI-based (high-confidence)
NODE_74_length_3965_cov_0.176668	3965	No	1	1	Medium-quality	61.67	AAI-based (high-confidence)
NODE_75_length_3475_cov_0.075237	3475	No	2	1	Medium-quality	55.03	AAI-based (high-confidence)
NODE_75_length_3621_cov_0.155877	3621	No	1	1	Medium-quality	56.56	AAI-based (high-confidence)
NODE_75_length_3719_cov_0.156077	3719	No	2	1	Medium-quality	74.29	AAI-based (high-confidence)
NODE_81_length_3668_cov_0.133931	3668	No	2	1	Medium-quality	79.05	AAI-based (high-confidence)
NODE_81_length_3915_cov_0.123952	3915	No	1	1	Medium-quality	59.03	AAI-based (high-confidence)
NODE_82_length_3661_cov_0.177428	3661	No	1	1	Medium-quality	56.37	AAI-based (high-confidence)
NODE_82_length_3780_cov_0.105678	3780	No	4	1	Medium-quality	77.59	AAI-based (high-confidence)
NODE_82_length_3912_cov_0.193811	3912	No	2	1	Medium-quality	62.52	AAI-based (high-confidence)
NODE_83_length_3638_cov_0.198644	3638	No	1	1	Medium-quality	55.02	AAI-based (high-confidence)
NODE_84_length_3357_cov_0.143033	3357	No	1	1	Medium-quality	69.86	AAI-based (high-confidence)
NODE_9_length_6142_cov_0.173424	6142	No	2	1	Medium-quality	82.57	AAI-based (high-confidence)
NODE_91_length_3263_cov_0.181732	3263	No	1	1	Medium-quality	70.76	AAI-based (high-confidence)
NODE_95_length_20308_cov_0.518086	20308	No	24	6	Medium-quality	59.45	AAI-based (high-confidence)
NODE_98_length_3205_cov_0.166452	3205	No	1	1	Medium-quality	66.63	AAI-based (high-confidence)
NODE_98_length_3690_cov_0.170426	3690	No	1	1	Medium-quality	66.18	AAI-based (high-confidence)
NODE_10_length_17641_cov_0.263140	17641	No	8	1	Low-quality	28.61	AAI-based (high-confidence)
NODE_10_length_5514_cov_0.104709	5514	No	7	2	Low-quality	18.34	AAI-based (high-confidence)
NODE_10_length_6071_cov_0.108674	6071	No	9	4	Low-quality	18.91	AAI-based (high-confidence)
NODE_10_length_7509_cov_0.129960	7509	No	20	4	Low-quality	23.09	AAI-based (high-confidence)
NODE_10_length_8634_cov_0.128881	8634	No	3	1	Low-quality	14.12	AAI-based (high-confidence)
NODE_10003_length_1797_cov_0.074205	1797	No	4	2	Low-quality	4.32	AAI-based (high-confidence)
NODE_10010_length_1067_cov_0.133264	1067	No	3	1	Low-quality	3.29	AAI-based (high-confidence)
NODE_10031_length_1793_cov_0.393152	1793	No	5	1	Low-quality	2.9	AAI-based (high-confidence)
NODE_10035_length_1623_cov_0.119423	1623	No	4	2	Low-quality	4.47	AAI-based (high-confidence)
NODE_10049_length_1065_cov_0.046584	1065	No	2	2	Low-quality	1.92	AAI-based (high-confidence)
NODE_1005_length_1398_cov_0.038491	1398	No	1	1	Low-quality	3.56	AAI-based (high-confidence)
NODE_10052_length_1805_cov_0.120750	1805	No	1	1	Low-quality	4.23	AAI-based (high-confidence)
NODE_10053_length_1805_cov_0.111372	1805	No	3	2	Low-quality	2.28	AAI-based (high-confidence)
NODE_10054_length_1805_cov_0.110785	1805	No	2	2	Low-quality	4.35	AAI-based (high-confidence)
NODE_10056_length_1034_cov_0.060963	1034	No	3	1	Low-quality	2.78	AAI-based (high-confidence)
NODE_10058_length_1805_cov_0.090856	1805	No	4	2	Low-quality	4.32	AAI-based (high-confidence)
NODE_10062_length_1790_cov_1.044352	1790	No	3	1	Low-quality	5.35	AAI-based (high-confidence)
NODE_10062_length_1804_cov_0.326686	1804	No	3	1	Low-quality	5.44	AAI-based (high-confidence)
NODE_10070_length_1790_cov_0.113542	1790	No	4	3	Low-quality	4.03	AAI-based (high-confidence)
NODE_1008_length_5346_cov_0.278826	5346	No	13	3	Low-quality	14.48	AAI-based (high-confidence)
NODE_1009_length_1580_cov_0.041188	1580	No	4	2	Low-quality	4.37	AAI-based (high-confidence)
NODE_101_length_3645_cov_0.095883	3645	No	8	2	Low-quality	10.53	AAI-based (high-confidence)
NODE_1010_length_1579_cov_0.123649	1579	No	2	1	Low-quality	32.93	AAI-based (high-confidence)
NODE_10103_length_1787_cov_0.100118	1787	No	2	1	Low-quality	3.7	AAI-based (high-confidence)
NODE_10110_length_1799_cov_0.338824	1799	No	2	2	Low-quality	2.96	AAI-based (high-confidence)
NODE_1012_length_1207_cov_0.055054	1207	No	1	1	Low-quality	1.98	AAI-based (high-confidence)

NODE_1012_length_1394_cov_0.140541	1394	No	2	1	Low-quality	2.58	AAI-based (high-confidence)
NODE_10127_length_1614_cov_0.461386	1614	No	4	1	Low-quality	3.71	AAI-based (high-confidence)
NODE_10133_length_1784_cov_0.204154	1784	No	2	1	Low-quality	3.14	AAI-based (high-confidence)
NODE_10147_length_1783_cov_0.100950	1783	No	2	2	Low-quality	4.06	AAI-based (high-confidence)
NODE_10167_length_1057_cov_0.083507	1057	No	2	1	Low-quality	2.85	AAI-based (high-confidence)
NODE_1017_length_6737_cov_0.298885	6737	No	9	2	Low-quality	15.66	AAI-based (high-confidence)
NODE_10171_length_1611_cov_0.256614	1611	No	3	1	Low-quality	2.55	AAI-based (high-confidence)
NODE_10173_length_1793_cov_0.217828	1793	No	4	2	Low-quality	4.93	AAI-based (high-confidence)
NODE_10174_length_1793_cov_0.210744	1793	No	5	3	Low-quality	5.37	AAI-based (high-confidence)
NODE_10183_length_1778_cov_0.114949	1778	No	5	3	Low-quality	5.01	AAI-based (high-confidence)
NODE_10185_length_1610_cov_0.078094	1610	No	2	1	Low-quality	5.1	AAI-based (high-confidence)
NODE_10195_length_1777_cov_0.113826	1777	No	4	4	Low-quality	2.88	AAI-based (high-confidence)
NODE_10198_length_1791_cov_0.115839	1791	No	2	2	Low-quality	2.83	AAI-based (high-confidence)
NODE_102_length_12337_cov_0.238438	12337	No	13	9	Low-quality	38.72	AAI-based (high-confidence)
NODE_102_length_3123_cov_0.136243	3123	No	1	1	Low-quality	43.02	AAI-based (high-confidence)
NODE_1020_length_5312_cov_0.167082	5312	No	9	5	Low-quality	2.83	AAI-based (high-confidence)
NODE_10205_length_1833_cov_0.108420	1833	No	4	2	Low-quality	3.99	AAI-based (high-confidence)
NODE_1021_length_2741_cov_0.384936	2741	No	4	2	Low-quality	7.67	AAI-based (high-confidence)
NODE_10210_length_1789_cov_0.208284	1789	No	2	1	Low-quality	2.73	AAI-based (high-confidence)
NODE_10227_length_1606_cov_0.096218	1606	No	2	2	Low-quality	3.3	AAI-based (high-confidence)
NODE_10229_length_1026_cov_0.072276	1026	No	1	1	Low-quality	2.33	AAI-based (high-confidence)
NODE_10229_length_1605_cov_0.151394	1605	No	1	1	Low-quality	4.89	AAI-based (high-confidence)
NODE_1025_length_5868_cov_0.551569	5868	No	6	2	Low-quality	11.98	AAI-based (high-confidence)
NODE_1026_length_5298_cov_0.166378	5298	No	7	5	Low-quality	11.69	AAI-based (high-confidence)
NODE_10262_length_1614_cov_0.122112	1614	No	1	1	Low-quality	3.63	AAI-based (high-confidence)
NODE_10266_length_1784_cov_0.125223	1784	No	6	1	Low-quality	2.9	AAI-based (high-confidence)
NODE_10267_length_1024_cov_0.104865	1024	No	2	1	Low-quality	2.66	AAI-based (high-confidence)
NODE_1027_length_2798_cov_0.210078	2798	No	7	1	Low-quality	6.54	AAI-based (high-confidence)
NODE_10274_length_1769_cov_0.104790	1769	No	3	1	Low-quality	4.76	AAI-based (high-confidence)
NODE_10289_length_1050_cov_0.084122	1050	No	1	1	Low-quality	3.28	AAI-based (high-confidence)
NODE_1029_length_2796_cov_0.093066	2796	No	5	2	Low-quality	5.73	AAI-based (high-confidence)
NODE_10297_length_1765_cov_0.143457	1765	No	3	3	Low-quality	2.93	AAI-based (high-confidence)
NODE_10308_length_1598_cov_0.321548	1598	No	6	3	Low-quality	2.6	AAI-based (high-confidence)
NODE_10309_length_1763_cov_0.097957	1763	No	4	1	Low-quality	3.25	AAI-based (high-confidence)
NODE_1031_length_1567_cov_0.111717	1567	No	1	1	Low-quality	22.12	AAI-based (high-confidence)
NODE_10318_length_1762_cov_0.164762	1762	No	2	1	Low-quality	5.44	AAI-based (high-confidence)
NODE_10318_length_1778_cov_0.258487	1778	No	4	1	Low-quality	2.79	AAI-based (high-confidence)
NODE_1032_length_5293_cov_0.156527	5293	No	7	2	Low-quality	5.39	AAI-based (high-confidence)
NODE_1032_length_6684_cov_0.369324	6684	No	13	8	Low-quality	11.05	AAI-based (high-confidence)
NODE_1033_length_1395_cov_0.050926	1395	No	3	1	Low-quality	2.23	AAI-based (high-confidence)
NODE_10331_length_1761_cov_0.120337	1761	No	5	2	Low-quality	5.61	AAI-based (high-confidence)
NODE_10335_length_1021_cov_0.112798	1021	No	2	1	Low-quality	2.22	AAI-based (high-confidence)
NODE_10344_length_1775_cov_0.627088	1775	No	2	2	Low-quality	2.93	AAI-based (high-confidence)
NODE_1035_length_5290_cov_0.356001	5290	No	7	5	Low-quality	2.93	AAI-based (high-confidence)
NODE_1037_length_1387_cov_0.062112	1387	No	1	1	Low-quality	29.04	AAI-based (high-confidence)
NODE_10370_length_1593_cov_0.247657	1593	No	5	2	Low-quality	3.43	AAI-based (high-confidence)

NODE_10379_length_1772_cov_0.095039	1772	No	3	1	Low-quality	4.41	AAI-based (high-confidence)
NODE_10386_length_1593_cov_0.062249	1593	No	4	2	Low-quality	2.77	AAI-based (high-confidence)
NODE_1039_length_5871_cov_0.145877	5871	No	11	5	Low-quality	2.87	AAI-based (high-confidence)
NODE_10393_length_1770_cov_0.341113	1770	No	4	2	Low-quality	3.92	AAI-based (high-confidence)
NODE_104_length_2706_cov_0.178750	2706	No	10	2	Low-quality	8.98	AAI-based (high-confidence)
NODE_1040_length_2785_cov_0.151899	2785	No	4	1	Low-quality	4.58	AAI-based (high-confidence)
NODE_10411_length_1769_cov_0.319162	1769	No	2	1	Low-quality	4	AAI-based (high-confidence)
NODE_1043_length_1624_cov_0.114098	1624	No	5	1	Low-quality	3.28	AAI-based (high-confidence)
NODE_10431_length_1595_cov_0.304813	1595	No	2	1	Low-quality	3.17	AAI-based (high-confidence)
NODE_10439_length_1588_cov_0.378778	1588	No	2	1	Low-quality	2.62	AAI-based (high-confidence)
NODE_1044_length_5273_cov_0.299768	5273	No	8	7	Low-quality	12.52	AAI-based (high-confidence)
NODE_10445_length_1588_cov_0.075218	1588	No	4	1	Low-quality	4.99	AAI-based (high-confidence)
NODE_1045_length_1423_cov_0.066465	1423	No	2	1	Low-quality	2.65	AAI-based (high-confidence)
NODE_1045_length_1560_cov_0.060917	1560	No	2	1	Low-quality	3.81	AAI-based (high-confidence)
NODE_1046_length_5850_cov_0.160668	5850	No	8	1	Low-quality	18.28	AAI-based (high-confidence)
NODE_1047_length_5263_cov_0.180287	5263	No	10	2	Low-quality	7.06	AAI-based (high-confidence)
NODE_10484_length_1763_cov_0.090745	1763	No	3	1	Low-quality	2.86	AAI-based (high-confidence)
NODE_10489_length_1037_cov_0.076759	1037	No	2	2	Low-quality	3.16	AAI-based (high-confidence)
NODE_105_length_3618_cov_0.171924	3618	No	4	3	Low-quality	6.95	AAI-based (high-confidence)
NODE_1050_length_2691_cov_0.195216	2691	No	8	2	Low-quality	4.21	AAI-based (high-confidence)
NODE_1051_length_5256_cov_0.179950	5256	No	9	5	Low-quality	2.96	AAI-based (high-confidence)
NODE_1052_length_5836_cov_0.292662	5836	No	10	5	Low-quality	2.79	AAI-based (high-confidence)
NODE_10529_length_1739_cov_0.409146	1739	No	3	1	Low-quality	2.81	AAI-based (high-confidence)
NODE_10537_length_1757_cov_0.521713	1757	No	5	1	Low-quality	2.89	AAI-based (high-confidence)
NODE_1054_length_1383_cov_0.070093	1383	No	2	1	Low-quality	2.33	AAI-based (high-confidence)
NODE_10547_length_1580_cov_0.139770	1580	No	2	2	Low-quality	2.63	AAI-based (high-confidence)
NODE_10547_length_1584_cov_0.148822	1584	No	3	2	Low-quality	4.99	AAI-based (high-confidence)
NODE_1055_length_1196_cov_0.044667	1196	No	4	1	Low-quality	2.74	AAI-based (high-confidence)
NODE_10563_length_1736_cov_0.874160	1736	No	3	2	Low-quality	5.28	AAI-based (high-confidence)
NODE_10566_length_1736_cov_0.166158	1736	No	3	1	Low-quality	7.09	AAI-based (high-confidence)
NODE_1057_length_5245_cov_0.149048	5245	No	3	3	Low-quality	8.61	AAI-based (high-confidence)
NODE_10577_length_1032_cov_0.192926	1032	No	2	1	Low-quality	2.33	AAI-based (high-confidence)
NODE_1059_length_1373_cov_0.079278	1373	No	1	1	Low-quality	2.65	AAI-based (high-confidence)
NODE_10590_length_1010_cov_0.045005	1010	No	2	1	Low-quality	2.39	AAI-based (high-confidence)
NODE_106_length_3604_cov_0.087874	3604	No	6	2	Low-quality	10.57	AAI-based (high-confidence)
NODE_1061_length_3809_cov_0.101078	3809	No	3	2	Low-quality	10.74	AAI-based (high-confidence)
NODE_10612_length_1009_cov_0.061538	1009	No	3	3	Low-quality	3.47	AAI-based (high-confidence)
NODE_10618_length_1577_cov_0.240866	1577	No	5	1	Low-quality	4.49	AAI-based (high-confidence)
NODE_1062_length_1549_cov_0.071724	1549	No	2	1	Low-quality	3.53	AAI-based (high-confidence)
NODE_1062_length_1584_cov_0.082155	1584	No	3	1	Low-quality	2.94	AAI-based (high-confidence)
NODE_10635_length_1029_cov_0.078495	1029	No	3	2	Low-quality	2.41	AAI-based (high-confidence)
NODE_10636_length_1571_cov_0.203804	1571	No	1	1	Low-quality	3.34	AAI-based (high-confidence)
NODE_10638_length_1730_cov_0.085837	1730	No	2	1	Low-quality	4.75	AAI-based (high-confidence)
NODE_1065_length_1580_cov_0.103984	1580	No	4	1	Low-quality	2.73	AAI-based (high-confidence)
NODE_1065_length_5229_cov_0.344834	5229	No	11	2	Low-quality	16.66	AAI-based (high-confidence)
NODE_10653_length_1727_cov_0.232801	1727	No	2	1	Low-quality	5.37	AAI-based (high-confidence)

NODE_10655_length_1028_cov_0.069968	1028	No	2	2	Low-quality	2.28	AAI-based (high-confidence)
NODE_10655_length_1570_cov_0.186268	1570	No	4	2	Low-quality	3.75	AAI-based (high-confidence)
NODE_10691_length_1740_cov_0.128580	1740	No	5	2	Low-quality	3.58	AAI-based (high-confidence)
NODE_10695_length_1740_cov_0.065204	1740	No	3	2	Low-quality	3.19	AAI-based (high-confidence)
NODE_107_length_3127_cov_0.162153	3127	No	1	1	Low-quality	49.41	AAI-based (high-confidence)
NODE_1070_length_2755_cov_0.176581	2755	No	4	1	Low-quality	4.28	AAI-based (high-confidence)
NODE_10710_length_1024_cov_0.099459	1024	No	4	1	Low-quality	2	AAI-based (high-confidence)
NODE_1072_length_5800_cov_0.170496	5800	No	6	3	Low-quality	16.32	AAI-based (high-confidence)
NODE_10723_length_1766_cov_0.185963	1766	No	2	1	Low-quality	4.85	AAI-based (high-confidence)
NODE_10747_length_1717_cov_0.163782	1717	No	6	1	Low-quality	5.21	AAI-based (high-confidence)
NODE_10749_length_1717_cov_0.059333	1717	No	4	1	Low-quality	3.16	AAI-based (high-confidence)
NODE_1076_length_2748_cov_0.126085	2748	No	4	1	Low-quality	4.83	AAI-based (high-confidence)
NODE_1077_length_1541_cov_0.085298	1541	No	1	1	Low-quality	24.56	AAI-based (high-confidence)
NODE_10776_length_1713_cov_0.188352	1713	No	5	3	Low-quality	2.75	AAI-based (high-confidence)
NODE_1079_length_5194_cov_0.325025	5194	No	5	1	Low-quality	4.63	AAI-based (high-confidence)
NODE_10795_length_1712_cov_0.098574	1712	No	2	2	Low-quality	2.82	AAI-based (high-confidence)
NODE_1080_length_1568_cov_0.093261	1568	No	2	1	Low-quality	3.52	AAI-based (high-confidence)
NODE_1080_length_5193_cov_0.230860	5193	No	4	4	Low-quality	8.57	AAI-based (high-confidence)
NODE_1080_length_6507_cov_0.431180	6507	No	17	2	Low-quality	10.13	AAI-based (high-confidence)
NODE_1081_length_1369_cov_0.062205	1369	No	1	1	Low-quality	21.86	AAI-based (high-confidence)
NODE_10830_length_1556_cov_0.108442	1556	No	2	1	Low-quality	2.89	AAI-based (high-confidence)
NODE_1084_length_5762_cov_0.420449	5762	No	8	1	Low-quality	10.76	AAI-based (high-confidence)
NODE_10847_length_1555_cov_0.067308	1555	No	4	3	Low-quality	2.82	AAI-based (high-confidence)
NODE_10848_length_1706_cov_0.258867	1706	No	4	1	Low-quality	2.82	AAI-based (high-confidence)
NODE_1086_length_1537_cov_0.069541	1537	No	3	2	Low-quality	2.85	AAI-based (high-confidence)
NODE_1086_length_1600_cov_0.088608	1600	No	1	1	Low-quality	24.54	AAI-based (high-confidence)
NODE_10861_length_1552_cov_0.152787	1552	No	4	1	Low-quality	4.84	AAI-based (high-confidence)
NODE_10873_length_1551_cov_0.146694	1551	No	2	1	Low-quality	3.98	AAI-based (high-confidence)
NODE_1088_length_1185_cov_0.048803	1185	No	2	1	Low-quality	2	AAI-based (high-confidence)
NODE_10880_length_1720_cov_0.415793	1720	No	3	1	Low-quality	2.85	AAI-based (high-confidence)
NODE_10890_length_1551_cov_0.046832	1551	No	2	1	Low-quality	3.57	AAI-based (high-confidence)
NODE_10897_length_1719_cov_0.208025	1719	No	2	2	Low-quality	4.67	AAI-based (high-confidence)
NODE_109_length_3531_cov_0.134907	3531	No	6	1	Low-quality	6.81	AAI-based (high-confidence)
NODE_1090_length_3760_cov_0.342529	3760	No	4	3	Low-quality	10.24	AAI-based (high-confidence)
NODE_1090_length_5161_cov_0.155077	5161	No	8	1	Low-quality	7.98	AAI-based (high-confidence)
NODE_10908_length_1549_cov_0.144138	1549	No	2	2	Low-quality	2.63	AAI-based (high-confidence)
NODE_10925_length_1699_cov_0.047500	1699	No	3	2	Low-quality	5.19	AAI-based (high-confidence)
NODE_1093_length_5739_cov_0.361702	5739	No	6	1	Low-quality	9.07	AAI-based (high-confidence)
NODE_10931_length_1547_cov_0.161602	1547	No	3	1	Low-quality	2.46	AAI-based (high-confidence)
NODE_1094_length_3754_cov_0.161970	3754	No	2	1	Low-quality	5.89	AAI-based (high-confidence)
NODE_10942_length_1547_cov_0.055939	1547	No	2	1	Low-quality	4.98	AAI-based (high-confidence)
NODE_1095_length_1363_cov_0.078323	1363	No	1	1	Low-quality	20.86	AAI-based (high-confidence)
NODE_1095_length_1396_cov_0.057055	1396	No	3	3	Low-quality	3.58	AAI-based (high-confidence)
NODE_10955_length_1011_cov_0.138158	1011	No	3	1	Low-quality	3.11	AAI-based (high-confidence)
NODE_10969_length_1713_cov_0.220570	1713	No	2	2	Low-quality	4.53	AAI-based (high-confidence)
NODE_1097_length_6436_cov_0.182105	6436	No	10	1	Low-quality	7.95	AAI-based (high-confidence)

NODE_10972_length_1543_cov_0.114266	1543	No	2	1	Low-quality	2.84	AAI-based (high-confidence)
NODE_1098_length_6431_cov_0.194883	6431	No	5	1	Low-quality	10	AAI-based (high-confidence)
NODE_1099_length_5721_cov_0.377446	5721	No	8	3	Low-quality	2.88	AAI-based (high-confidence)
NODE_11_length_17706_cov_0.186006	17706	No	32	15	Low-quality	37.35	AAI-based (high-confidence)
NODE_11_length_5499_cov_0.116852	5499	No	10	3	Low-quality	12.7	AAI-based (high-confidence)
NODE_11_length_6487_cov_0.166093	6487	No	14	6	Low-quality	18.02	AAI-based (high-confidence)
NODE_11_length_6592_cov_0.177730	6592	No	11	5	Low-quality	14.42	AAI-based (high-confidence)
NODE_110_length_11867_cov_0.376869	11867	No	15	5	Low-quality	37.1	AAI-based (high-confidence)
NODE_110_length_7044_cov_0.188625	7044	No	9	1	Low-quality	20.16	AAI-based (high-confidence)
NODE_1100_length_2726_cov_0.197944	2726	No	4	1	Low-quality	7.77	AAI-based (high-confidence)
NODE_1101_length_2639_cov_0.334252	2639	No	3	1	Low-quality	7.38	AAI-based (high-confidence)
NODE_11016_length_1008_cov_0.282728	1008	No	3	1	Low-quality	2.74	AAI-based (high-confidence)
NODE_11027_length_1540_cov_0.204719	1540	No	2	1	Low-quality	2.55	AAI-based (high-confidence)
NODE_11028_length_1707_cov_0.536692	1707	No	3	1	Low-quality	4.33	AAI-based (high-confidence)
NODE_1104_length_1358_cov_0.088959	1358	No	2	1	Low-quality	3.93	AAI-based (high-confidence)
NODE_1105_length_3733_cov_0.254265	3733	No	6	4	Low-quality	9.91	AAI-based (high-confidence)
NODE_11053_length_1688_cov_0.250472	1688	No	4	3	Low-quality	2.34	AAI-based (high-confidence)
NODE_11058_length_1005_cov_1.746137	1005	No	3	1	Low-quality	3.08	AAI-based (high-confidence)
NODE_11062_length_1687_cov_0.634761	1687	No	4	1	Low-quality	4.98	AAI-based (high-confidence)
NODE_11067_length_1687_cov_0.107053	1687	No	1	1	Low-quality	2.78	AAI-based (high-confidence)
NODE_1107_length_5107_cov_0.672524	5107	No	10	2	Low-quality	9.32	AAI-based (high-confidence)
NODE_11074_length_1686_cov_0.095148	1686	No	3	1	Low-quality	2.75	AAI-based (high-confidence)
NODE_11077_length_1686_cov_0.084436	1686	No	5	2	Low-quality	5.05	AAI-based (high-confidence)
NODE_11088_length_1536_cov_0.136395	1536	No	2	1	Low-quality	2.83	AAI-based (high-confidence)
NODE_111_length_3514_cov_0.075256	3514	No	8	2	Low-quality	12.01	AAI-based (high-confidence)
NODE_11106_length_1531_cov_0.095670	1531	No	1	1	Low-quality	3.09	AAI-based (high-confidence)
NODE_1111_length_5092_cov_0.435209	5092	No	11	3	Low-quality	11.45	AAI-based (high-confidence)
NODE_11110_length_1700_cov_0.150531	1700	No	2	1	Low-quality	4.11	AAI-based (high-confidence)
NODE_1112_length_1389_cov_0.067442	1389	No	1	1	Low-quality	3.18	AAI-based (high-confidence)
NODE_11122_length_1002_cov_0.101883	1002	No	1	1	Low-quality	2.71	AAI-based (high-confidence)
NODE_1113_length_1549_cov_0.060690	1549	No	2	1	Low-quality	2.6	AAI-based (high-confidence)
NODE_1113_length_5612_cov_0.226193	5612	No	8	2	Low-quality	6.13	AAI-based (high-confidence)
NODE_1114_length_1522_cov_0.444132	1522	No	5	1	Low-quality	4.69	AAI-based (high-confidence)
NODE_1116_length_1521_cov_0.194796	1521	No	4	2	Low-quality	2.85	AAI-based (high-confidence)
NODE_1116_length_5083_cov_0.269262	5083	No	4	2	Low-quality	9.64	AAI-based (high-confidence)
NODE_1118_length_1351_cov_0.115815	1351	No	1	1	Low-quality	20.97	AAI-based (high-confidence)
NODE_1118_length_3718_cov_0.210832	3718	No	8	3	Low-quality	11.62	AAI-based (high-confidence)
NODE_11182_length_1714_cov_0.097833	1714	No	2	1	Low-quality	5.27	AAI-based (high-confidence)
NODE_11195_length_1693_cov_0.275408	1693	No	4	2	Low-quality	4.69	AAI-based (high-confidence)
NODE_112_length_6998_cov_0.181765	6998	No	14	3	Low-quality	11.82	AAI-based (high-confidence)
NODE_1120_length_1519_cov_0.101408	1519	No	3	1	Low-quality	4.46	AAI-based (high-confidence)
NODE_1120_length_5070_cov_0.164957	5070	No	5	3	Low-quality	2.37	AAI-based (high-confidence)
NODE_1120_length_6371_cov_1.300702	6371	No	9	6	Low-quality	3.55	AAI-based (high-confidence)
NODE_11205_length_1693_cov_0.063363	1693	No	5	1	Low-quality	4.47	AAI-based (high-confidence)
NODE_1121_length_5663_cov_0.433321	5663	No	5	2	Low-quality	2.6	AAI-based (high-confidence)
NODE_1122_length_1895_cov_0.079621	1895	No	2	2	Low-quality	3.3	AAI-based (high-confidence)

NODE_1122_length_5066_cov_0.403463	5066	No	7	5	Low-quality	2.89	AAI-based (high-confidence)
NODE_11220_length_1671_cov_0.218193	1671	No	2	2	Low-quality	2.62	AAI-based (high-confidence)
NODE_1123_length_1583_cov_0.043127	1583	No	3	1	Low-quality	4.35	AAI-based (high-confidence)
NODE_1123_length_1894_cov_0.100279	1894	No	4	1	Low-quality	3.31	AAI-based (high-confidence)
NODE_11232_length_1689_cov_0.125786	1689	No	2	2	Low-quality	3.21	AAI-based (high-confidence)
NODE_1124_length_1349_cov_0.168800	1349	No	1	1	Low-quality	26.1	AAI-based (high-confidence)
NODE_1124_length_5657_cov_0.172364	5657	No	12	2	Low-quality	8.2	AAI-based (high-confidence)
NODE_11242_length_1525_cov_0.092567	1525	No	4	1	Low-quality	3.98	AAI-based (high-confidence)
NODE_11251_length_1524_cov_0.101053	1524	No	3	1	Low-quality	3.71	AAI-based (high-confidence)
NODE_11257_length_1523_cov_0.370787	1523	No	1	1	Low-quality	3.69	AAI-based (high-confidence)
NODE_1127_length_3706_cov_0.116440	3706	No	3	3	Low-quality	6.72	AAI-based (high-confidence)
NODE_1127_length_6355_cov_0.167040	6355	No	16	3	Low-quality	20.27	AAI-based (high-confidence)
NODE_11284_length_1514_cov_0.145583	1514	No	1	1	Low-quality	4.76	AAI-based (high-confidence)
NODE_113_length_3068_cov_0.118895	3068	No	6	2	Low-quality	9.7	AAI-based (high-confidence)
NODE_113_length_6997_cov_0.636851	6997	No	7	6	Low-quality	3.89	AAI-based (high-confidence)
NODE_1130_length_1542_cov_0.101178	1542	No	2	1	Low-quality	2.58	AAI-based (high-confidence)
NODE_1130_length_3701_cov_0.285119	3701	No	4	1	Low-quality	10.22	AAI-based (high-confidence)
NODE_11302_length_1520_cov_0.125968	1520	No	2	2	Low-quality	4.28	AAI-based (high-confidence)
NODE_11319_length_1696_cov_0.107702	1696	No	2	2	Low-quality	3.98	AAI-based (high-confidence)
NODE_1132_length_6337_cov_0.308913	6337	No	15	2	Low-quality	3.7	AAI-based (high-confidence)
NODE_11320_length_1661_cov_1.075544	1661	No	4	1	Low-quality	4.67	AAI-based (high-confidence)
NODE_11320_length_1682_cov_0.060644	1682	No	1	1	Low-quality	5.14	AAI-based (high-confidence)
NODE_1135_length_1513_cov_0.094767	1513	No	1	1	Low-quality	22.35	AAI-based (high-confidence)
NODE_1135_length_2598_cov_0.142057	2598	No	3	3	Low-quality	7.87	AAI-based (high-confidence)
NODE_11353_length_1658_cov_0.202694	1658	No	5	1	Low-quality	4.6	AAI-based (high-confidence)
NODE_1136_length_1573_cov_0.104478	1573	No	1	1	Low-quality	30.46	AAI-based (high-confidence)
NODE_1136_length_3687_cov_0.116778	3687	No	5	3	Low-quality	9	AAI-based (high-confidence)
NODE_1136_length_5036_cov_0.230099	5036	No	5	3	Low-quality	9.37	AAI-based (high-confidence)
NODE_11364_length_1515_cov_0.151836	1515	No	2	1	Low-quality	3.51	AAI-based (high-confidence)
NODE_11366_length_1679_cov_0.033544	1679	No	3	2	Low-quality	3.06	AAI-based (high-confidence)
NODE_11369_length_1689_cov_0.132075	1689	No	2	1	Low-quality	5.21	AAI-based (high-confidence)
NODE_1137_length_1341_cov_0.049919	1341	No	4	1	Low-quality	2.41	AAI-based (high-confidence)
NODE_1138_length_5623_cov_0.241854	5623	No	11	3	Low-quality	8.75	AAI-based (high-confidence)
NODE_11385_length_1654_cov_0.190997	1654	No	3	1	Low-quality	3.63	AAI-based (high-confidence)
NODE_114_length_3039_cov_0.106463	3039	No	3	1	Low-quality	4.93	AAI-based (high-confidence)
NODE_1140_length_3681_cov_0.160246	3681	No	4	1	Low-quality	6.85	AAI-based (high-confidence)
NODE_1140_length_6313_cov_0.341648	6313	No	20	1	Low-quality	10.04	AAI-based (high-confidence)
NODE_1141_length_1572_cov_0.054311	1572	No	4	2	Low-quality	3.88	AAI-based (high-confidence)
NODE_11411_length_1653_cov_0.075290	1653	No	6	1	Low-quality	3.46	AAI-based (high-confidence)
NODE_11413_length_1652_cov_0.292337	1652	No	3	3	Low-quality	3.06	AAI-based (high-confidence)
NODE_1142_length_1572_cov_0.048880	1572	No	3	1	Low-quality	3.33	AAI-based (high-confidence)
NODE_11421_length_1651_cov_1.568943	1651	No	4	2	Low-quality	4.8	AAI-based (high-confidence)
NODE_11447_length_1510_cov_0.065202	1510	No	2	1	Low-quality	3.39	AAI-based (high-confidence)
NODE_11448_length_1680_cov_0.087919	1680	No	4	1	Low-quality	3.92	AAI-based (high-confidence)
NODE_1145_length_3672_cov_0.487545	3672	No	6	2	Low-quality	2.07	AAI-based (high-confidence)
NODE_11450_length_1671_cov_0.215649	1671	No	3	1	Low-quality	4.97	AAI-based (high-confidence)

NODE_11456_length_1498_cov_0.290922	1498	No	2	1	Low-quality	4.07	AAI-based (high-confidence)
NODE_11457_length_1671_cov_0.105598	1671	No	3	1	Low-quality	4.6	AAI-based (high-confidence)
NODE_11460_length_1508_cov_0.130589	1508	No	3	1	Low-quality	3.51	AAI-based (high-confidence)
NODE_11461_length_1670_cov_0.285805	1670	No	6	1	Low-quality	4.82	AAI-based (high-confidence)
NODE_11470_length_1670_cov_0.087206	1670	No	4	2	Low-quality	3.19	AAI-based (high-confidence)
NODE_11473_length_1507_cov_0.123580	1507	No	4	1	Low-quality	2.72	AAI-based (high-confidence)
NODE_1148_length_6290_cov_0.364561	6290	No	9	2	Low-quality	3.02	AAI-based (high-confidence)
NODE_11485_length_1669_cov_0.075796	1669	No	1	1	Low-quality	5.03	AAI-based (high-confidence)
NODE_11486_length_1668_cov_0.245379	1668	No	5	1	Low-quality	4.8	AAI-based (high-confidence)
NODE_11488_length_1496_cov_0.281317	1496	No	2	2	Low-quality	3.07	AAI-based (high-confidence)
NODE_11491_length_1668_cov_0.096877	1668	No	3	1	Low-quality	4.57	AAI-based (high-confidence)
NODE_11497_length_1646_cov_0.110537	1646	No	3	1	Low-quality	3.31	AAI-based (high-confidence)
NODE_115_length_3012_cov_0.147957	3012	No	5	2	Low-quality	9.37	AAI-based (high-confidence)
NODE_115_length_3052_cov_0.144260	3052	No	8	1	Low-quality	9.34	AAI-based (high-confidence)
NODE_115_length_3520_cov_0.079801	3520	No	6	1	Low-quality	6.37	AAI-based (high-confidence)
NODE_1150_length_2689_cov_0.164479	2689	No	4	2	Low-quality	6.57	AAI-based (high-confidence)
NODE_11501_length_1667_cov_0.230867	1667	No	2	1	Low-quality	3.24	AAI-based (high-confidence)
NODE_11502_length_1495_cov_0.176934	1495	No	4	2	Low-quality	2.49	AAI-based (high-confidence)
NODE_11508_length_1666_cov_0.185705	1666	No	4	2	Low-quality	3.54	AAI-based (high-confidence)
NODE_1151_length_1339_cov_0.074194	1339	No	2	1	Low-quality	3.69	AAI-based (high-confidence)
NODE_11510_length_1666_cov_0.114231	1666	No	2	2	Low-quality	4.63	AAI-based (high-confidence)
NODE_1152_length_5583_cov_0.818381	5583	No	15	3	Low-quality	16.51	AAI-based (high-confidence)
NODE_11530_length_1503_cov_0.103276	1503	No	4	1	Low-quality	4.69	AAI-based (high-confidence)
NODE_11535_length_1502_cov_0.484676	1502	No	3	2	Low-quality	3.87	AAI-based (high-confidence)
NODE_11539_length_1492_cov_0.106246	1492	No	4	2	Low-quality	4.11	AAI-based (high-confidence)
NODE_11541_length_1664_cov_0.111182	1664	No	4	1	Low-quality	3.04	AAI-based (high-confidence)
NODE_1155_length_5008_cov_0.117539	5008	No	9	3	Low-quality	4.74	AAI-based (high-confidence)
NODE_11555_length_1663_cov_0.121483	1663	No	2	2	Low-quality	3.16	AAI-based (high-confidence)
NODE_11557_length_1663_cov_0.118926	1663	No	2	1	Low-quality	3.74	AAI-based (high-confidence)
NODE_11568_length_1501_cov_0.055635	1501	No	3	3	Low-quality	3.67	AAI-based (high-confidence)
NODE_11573_length_1500_cov_0.147038	1500	No	3	1	Low-quality	4.63	AAI-based (high-confidence)
NODE_1158_length_6258_cov_1.286897	6258	No	11	3	Low-quality	3.62	AAI-based (high-confidence)
NODE_11580_length_1499_cov_0.180714	1499	No	1	1	Low-quality	3.52	AAI-based (high-confidence)
NODE_11581_length_1639_cov_0.105195	1639	No	3	2	Low-quality	2.7	AAI-based (high-confidence)
NODE_1159_length_1162_cov_0.096896	1162	No	3	1	Low-quality	2.31	AAI-based (high-confidence)
NODE_1159_length_2680_cov_0.183650	2680	No	3	2	Low-quality	4.2	AAI-based (high-confidence)
NODE_11596_length_1487_cov_0.095821	1487	No	2	1	Low-quality	3.75	AAI-based (high-confidence)
NODE_116_length_3010_cov_0.107523	3010	No	4	1	Low-quality	8.67	AAI-based (high-confidence)
NODE_1160_length_5562_cov_0.922936	5562	No	6	2	Low-quality	11.34	AAI-based (high-confidence)
NODE_1160_length_6256_cov_0.380867	6256	No	7	3	Low-quality	19.24	AAI-based (high-confidence)
NODE_11600_length_1659_cov_0.082051	1659	No	3	1	Low-quality	2.1	AAI-based (high-confidence)
NODE_11616_length_1497_cov_0.075823	1497	No	3	2	Low-quality	2.49	AAI-based (high-confidence)
NODE_1162_length_2572_cov_0.195309	2572	No	5	1	Low-quality	7.5	AAI-based (high-confidence)
NODE_1162_length_2679_cov_0.253876	2679	No	4	2	Low-quality	8.38	AAI-based (high-confidence)
NODE_1163_length_1323_cov_0.140523	1323	No	1	1	Low-quality	27.02	AAI-based (high-confidence)
NODE_1163_length_4988_cov_0.203723	4988	No	2	1	Low-quality	10.17	AAI-based (high-confidence)

NODE_11639_length_1495_cov_0.078080	1495	No	3	1	Low-quality	4.19	AAI-based (high-confidence)
NODE_1164_length_2679_cov_0.160853	2679	No	3	1	Low-quality	6.1	AAI-based (high-confidence)
NODE_11666_length_1652_cov_0.106246	1652	No	2	2	Low-quality	4.1	AAI-based (high-confidence)
NODE_1167_length_1871_cov_0.111738	1871	No	1	1	Low-quality	36.01	AAI-based (high-confidence)
NODE_1167_length_5553_cov_0.259443	5553	No	6	3	Low-quality	10.17	AAI-based (high-confidence)
NODE_11682_length_1629_cov_0.189542	1629	No	2	1	Low-quality	5.08	AAI-based (high-confidence)
NODE_11689_length_1650_cov_0.562863	1650	No	4	1	Low-quality	2.19	AAI-based (high-confidence)
NODE_1170_length_6216_cov_0.169691	6216	No	9	6	Low-quality	18.31	AAI-based (high-confidence)
NODE_11700_length_1650_cov_0.098646	1650	No	2	1	Low-quality	3.1	AAI-based (high-confidence)
NODE_1172_length_1521_cov_0.080169	1521	No	3	2	Low-quality	5.02	AAI-based (high-confidence)
NODE_1173_length_1868_cov_0.042962	1868	No	5	1	Low-quality	3.34	AAI-based (high-confidence)
NODE_11739_length_1646_cov_0.237880	1646	No	2	2	Low-quality	2.19	AAI-based (high-confidence)
NODE_11740_length_1624_cov_0.191475	1624	No	2	1	Low-quality	3.24	AAI-based (high-confidence)
NODE_11744_length_1646_cov_0.133161	1646	No	5	1	Low-quality	4.26	AAI-based (high-confidence)
NODE_1175_length_1551_cov_0.086777	1551	No	2	1	Low-quality	4.64	AAI-based (high-confidence)
NODE_11766_length_1622_cov_0.640840	1622	No	4	1	Low-quality	3.49	AAI-based (high-confidence)
NODE_11769_length_1622_cov_0.261326	1622	No	3	2	Low-quality	3.72	AAI-based (high-confidence)
NODE_1177_length_1550_cov_0.217781	1550	No	7	1	Low-quality	3.13	AAI-based (high-confidence)
NODE_1177_length_2558_cov_0.221228	2558	No	3	2	Low-quality	8.07	AAI-based (high-confidence)
NODE_11772_length_1643_cov_0.352332	1643	No	2	1	Low-quality	5.11	AAI-based (high-confidence)
NODE_1178_length_3618_cov_0.142086	3618	No	8	1	Low-quality	11.36	AAI-based (high-confidence)
NODE_1178_length_6185_cov_0.438383	6185	No	6	4	Low-quality	9.7	AAI-based (high-confidence)
NODE_11781_length_1643_cov_0.324482	1643	No	3	2	Low-quality	3.02	AAI-based (high-confidence)
NODE_11788_length_1621_cov_0.155059	1621	No	3	2	Low-quality	4.36	AAI-based (high-confidence)
NODE_11790_length_1621_cov_0.143890	1621	No	7	3	Low-quality	3.34	AAI-based (high-confidence)
NODE_11795_length_1641_cov_0.192607	1641	No	2	1	Low-quality	5.09	AAI-based (high-confidence)
NODE_118_length_3005_cov_0.134893	3005	No	4	1	Low-quality	9.39	AAI-based (high-confidence)
NODE_11814_length_1640_cov_0.112265	1640	No	5	3	Low-quality	3.12	AAI-based (high-confidence)
NODE_1183_length_6170_cov_0.345907	6170	No	5	2	Low-quality	2.99	AAI-based (high-confidence)
NODE_11832_length_1482_cov_0.135213	1482	No	1	1	Low-quality	2.76	AAI-based (high-confidence)
NODE_11840_length_1637_cov_0.249025	1637	No	5	2	Low-quality	4.57	AAI-based (high-confidence)
NODE_11848_length_1616_cov_0.123270	1616	No	4	2	Low-quality	2.93	AAI-based (high-confidence)
NODE_11866_length_1614_cov_0.216502	1614	No	3	1	Low-quality	4.75	AAI-based (high-confidence)
NODE_11869_length_1635_cov_0.151693	1635	No	3	2	Low-quality	3.1	AAI-based (high-confidence)
NODE_1188_length_3600_cov_0.252499	3600	No	5	3	Low-quality	6.48	AAI-based (high-confidence)
NODE_11889_length_1478_cov_0.379985	1478	No	4	1	Low-quality	3.24	AAI-based (high-confidence)
NODE_11889_length_1613_cov_0.070013	1613	No	2	1	Low-quality	5.33	AAI-based (high-confidence)
NODE_1189_length_1323_cov_0.112745	1323	No	1	1	Low-quality	2.55	AAI-based (high-confidence)
NODE_11890_length_1633_cov_0.440678	1633	No	4	2	Low-quality	3.85	AAI-based (high-confidence)
NODE_1190_length_5515_cov_0.704764	5515	No	6	2	Low-quality	10.32	AAI-based (high-confidence)
NODE_1190_length_6155_cov_0.320674	6155	No	10	5	Low-quality	3.44	AAI-based (high-confidence)
NODE_1192_length_2657_cov_0.079359	2657	No	3	3	Low-quality	8.35	AAI-based (high-confidence)
NODE_11927_length_1609_cov_0.116556	1609	No	4	2	Low-quality	2.9	AAI-based (high-confidence)
NODE_11953_length_1453_cov_0.087149	1453	No	4	1	Low-quality	2.26	AAI-based (high-confidence)
NODE_1196_length_1322_cov_0.089943	1322	No	3	1	Low-quality	2.65	AAI-based (high-confidence)
NODE_11960_length_1452_cov_0.178862	1452	No	2	2	Low-quality	2.53	AAI-based (high-confidence)

NODE_11990_length_1625_cov_0.450197	1625	No	3	2	Low-quality	2.55	AAI-based (high-confidence)
NODE_12_length_3298_cov_0.127540	3298	No	7	2	Low-quality	7.86	AAI-based (high-confidence)
NODE_1200_length_1343_cov_0.153537	1343	No	2	1	Low-quality	2.51	AAI-based (high-confidence)
NODE_12045_length_1445_cov_0.189450	1445	No	2	2	Low-quality	4.49	AAI-based (high-confidence)
NODE_12051_length_1467_cov_0.076754	1467	No	2	1	Low-quality	3.97	AAI-based (high-confidence)
NODE_12055_length_1619_cov_0.444079	1619	No	3	1	Low-quality	5.11	AAI-based (high-confidence)
NODE_12058_length_1466_cov_0.252377	1466	No	5	4	Low-quality	4.43	AAI-based (high-confidence)
NODE_12064_length_1599_cov_0.075333	1599	No	3	1	Low-quality	4.47	AAI-based (high-confidence)
NODE_1207_length_6102_cov_0.256872	6102	No	4	2	Low-quality	9.49	AAI-based (high-confidence)
NODE_12087_length_1464_cov_0.182418	1464	No	2	2	Low-quality	2.38	AAI-based (high-confidence)
NODE_12092_length_1616_cov_0.317732	1616	No	3	1	Low-quality	4.91	AAI-based (high-confidence)
NODE_121_length_6833_cov_0.215325	6833	No	5	1	Low-quality	19.2	AAI-based (high-confidence)
NODE_12118_length_1594_cov_0.426756	1594	No	6	2	Low-quality	4.04	AAI-based (high-confidence)
NODE_12118_length_1614_cov_0.166337	1614	No	6	3	Low-quality	3.38	AAI-based (high-confidence)
NODE_1213_length_2632_cov_0.143308	2632	No	5	1	Low-quality	4.13	AAI-based (high-confidence)
NODE_12131_length_1614_cov_0.084488	1614	No	2	2	Low-quality	3.06	AAI-based (high-confidence)
NODE_12135_length_1461_cov_0.203377	1461	No	1	1	Low-quality	2.43	AAI-based (high-confidence)
NODE_12138_length_1461_cov_0.145374	1461	No	3	1	Low-quality	4.59	AAI-based (high-confidence)
NODE_1214_length_4888_cov_0.286072	4888	No	13	3	Low-quality	10.32	AAI-based (high-confidence)
NODE_12146_length_1460_cov_0.227774	1460	No	3	1	Low-quality	2.42	AAI-based (high-confidence)
NODE_1215_length_1496_cov_0.187545	1496	No	2	1	Low-quality	27.58	AAI-based (high-confidence)
NODE_1215_length_2631_cov_0.161927	2631	No	6	1	Low-quality	4.27	AAI-based (high-confidence)
NODE_1216_length_1303_cov_0.038206	1303	No	4	2	Low-quality	3.62	AAI-based (high-confidence)
NODE_1216_length_5457_cov_0.268384	5457	No	1	1	Low-quality	2.46	AAI-based (high-confidence)
NODE_1217_length_2513_cov_0.148716	2513	No	5	1	Low-quality	9.67	AAI-based (high-confidence)
NODE_12170_length_1603_cov_2.811170	1603	No	5	1	Low-quality	4.82	AAI-based (high-confidence)
NODE_12176_length_1589_cov_0.157047	1589	No	1	1	Low-quality	2.76	AAI-based (high-confidence)
NODE_12186_length_1588_cov_0.198120	1588	No	4	2	Low-quality	4.84	AAI-based (high-confidence)
NODE_1219_length_3555_cov_0.118056	3555	No	4	4	Low-quality	6.72	AAI-based (high-confidence)
NODE_122_length_2092_cov_0.061214	2092	No	2	1	Low-quality	4.44	AAI-based (high-confidence)
NODE_122_length_3409_cov_0.115408	3409	No	4	1	Low-quality	9.85	AAI-based (high-confidence)
NODE_12200_length_1458_cov_0.077263	1458	No	4	2	Low-quality	3.42	AAI-based (high-confidence)
NODE_12208_length_1606_cov_0.114798	1606	No	2	1	Low-quality	3.43	AAI-based (high-confidence)
NODE_1221_length_2623_cov_0.104200	2623	No	4	1	Low-quality	3.54	AAI-based (high-confidence)
NODE_12214_length_1587_cov_0.081317	1587	No	1	1	Low-quality	4.38	AAI-based (high-confidence)
NODE_12221_length_1605_cov_0.140770	1605	No	1	1	Low-quality	2.75	AAI-based (high-confidence)
NODE_1223_length_1299_cov_0.066667	1299	No	3	1	Low-quality	3.96	AAI-based (high-confidence)
NODE_1223_length_2620_cov_0.119000	2620	No	6	1	Low-quality	8.41	AAI-based (high-confidence)
NODE_1223_length_3552_cov_0.136693	3552	No	8	3	Low-quality	1.92	AAI-based (high-confidence)
NODE_1223_length_4876_cov_0.184216	4876	No	5	4	Low-quality	8.04	AAI-based (high-confidence)
NODE_12231_length_1455_cov_0.261799	1455	No	3	1	Low-quality	4.36	AAI-based (high-confidence)
NODE_1224_length_1492_cov_0.042355	1492	No	3	1	Low-quality	4.17	AAI-based (high-confidence)
NODE_1224_length_6067_cov_1.150972	6067	No	9	5	Low-quality	3.45	AAI-based (high-confidence)
NODE_12248_length_1454_cov_0.199262	1454	No	1	1	Low-quality	3.39	AAI-based (high-confidence)
NODE_1227_length_6064_cov_0.190277	6064	No	7	6	Low-quality	3.4	AAI-based (high-confidence)
NODE_1229_length_1491_cov_0.087644	1491	No	3	2	Low-quality	2.73	AAI-based (high-confidence)

NODE_123_length_2597_cov_0.078863	2597	No	7	3	Low-quality	8.07	AAI-based (high-confidence)
NODE_1230_length_1308_cov_0.146402	1308	No	1	1	Low-quality	2.5	AAI-based (high-confidence)
NODE_12300_length_1578_cov_0.077755	1578	No	3	1	Low-quality	2	AAI-based (high-confidence)
NODE_12341_length_1448_cov_0.058562	1448	No	2	1	Low-quality	4.5	AAI-based (high-confidence)
NODE_12341_length_1594_cov_1.029431	1594	No	3	1	Low-quality	4.74	AAI-based (high-confidence)
NODE_1235_length_3530_cov_0.118624	3530	No	8	1	Low-quality	9.02	AAI-based (high-confidence)
NODE_12353_length_1593_cov_0.232262	1593	No	4	1	Low-quality	4.97	AAI-based (high-confidence)
NODE_1236_length_1517_cov_0.135402	1517	No	3	2	Low-quality	2.94	AAI-based (high-confidence)
NODE_12375_length_1572_cov_0.226069	1572	No	2	1	Low-quality	2.47	AAI-based (high-confidence)
NODE_1238_length_1461_cov_0.111601	1461	No	3	3	Low-quality	3.68	AAI-based (high-confidence)
NODE_1239_length_6014_cov_0.291801	6014	No	16	2	Low-quality	18.68	AAI-based (high-confidence)
NODE_12397_length_1590_cov_0.393696	1590	No	6	1	Low-quality	2.56	AAI-based (high-confidence)
NODE_12399_length_1418_cov_0.090978	1418	No	3	1	Low-quality	2.3	AAI-based (high-confidence)
NODE_124_length_2963_cov_0.134777	2963	No	2	1	Low-quality	4.93	AAI-based (high-confidence)
NODE_12400_length_1444_cov_0.085502	1444	No	2	1	Low-quality	2.69	AAI-based (high-confidence)
NODE_12400_length_1570_cov_0.076818	1570	No	1	1	Low-quality	4.31	AAI-based (high-confidence)
NODE_12415_length_1417_cov_0.083460	1417	No	2	1	Low-quality	3.16	AAI-based (high-confidence)
NODE_12419_length_1417_cov_0.053111	1417	No	2	1	Low-quality	4.35	AAI-based (high-confidence)
NODE_1243_length_1246_cov_0.089799	1246	No	4	2	Low-quality	3.87	AAI-based (high-confidence)
NODE_1243_length_3518_cov_0.112314	3518	No	4	1	Low-quality	5.39	AAI-based (high-confidence)
NODE_1243_length_5292_cov_0.155402	5292	No	6	4	Low-quality	9.13	AAI-based (high-confidence)
NODE_1244_length_1515_cov_0.110169	1515	No	2	1	Low-quality	4.33	AAI-based (high-confidence)
NODE_1244_length_5999_cov_0.179153	5999	No	5	2	Low-quality	20.45	AAI-based (high-confidence)
NODE_1245_length_1457_cov_0.092047	1457	No	2	1	Low-quality	3.5	AAI-based (high-confidence)
NODE_1245_length_1484_cov_0.174007	1484	No	2	2	Low-quality	4.58	AAI-based (high-confidence)
NODE_1245_length_5401_cov_0.235383	5401	No	9	4	Low-quality	10.83	AAI-based (high-confidence)
NODE_1248_length_3512_cov_0.165837	3512	No	5	2	Low-quality	10.82	AAI-based (high-confidence)
NODE_125_length_3404_cov_0.080182	3404	No	3	1	Low-quality	6.34	AAI-based (high-confidence)
NODE_12533_length_1579_cov_0.156757	1579	No	2	2	Low-quality	2.84	AAI-based (high-confidence)
NODE_1255_length_4822_cov_0.337709	4822	No	3	3	Low-quality	7.96	AAI-based (high-confidence)
NODE_1256_length_1510_cov_0.051028	1510	No	4	3	Low-quality	4.14	AAI-based (high-confidence)
NODE_12560_length_1577_cov_0.283491	1577	No	5	1	Low-quality	3.22	AAI-based (high-confidence)
NODE_12562_length_1434_cov_0.080150	1434	No	1	1	Low-quality	3.25	AAI-based (high-confidence)
NODE_12570_length_1433_cov_0.124438	1433	No	3	1	Low-quality	3.17	AAI-based (high-confidence)
NODE_1260_length_1238_cov_0.113257	1238	No	2	1	Low-quality	4.18	AAI-based (high-confidence)
NODE_1260_length_1451_cov_0.150148	1451	No	3	2	Low-quality	2.82	AAI-based (high-confidence)
NODE_12607_length_1431_cov_0.051802	1431	No	6	1	Low-quality	4.35	AAI-based (high-confidence)
NODE_1262_length_2585_cov_0.173371	2585	No	4	4	Low-quality	6.98	AAI-based (high-confidence)
NODE_1263_length_1133_cov_0.085106	1133	No	2	2	Low-quality	2.01	AAI-based (high-confidence)
NODE_12634_length_1399_cov_0.346923	1399	No	2	1	Low-quality	4.23	AAI-based (high-confidence)
NODE_12635_length_1554_cov_0.090034	1554	No	4	1	Low-quality	4.78	AAI-based (high-confidence)
NODE_1265_length_2584_cov_0.387928	2584	No	5	3	Low-quality	5.91	AAI-based (high-confidence)
NODE_1265_length_7440_cov_0.848658	7440	No	18	2	Low-quality	23.88	AAI-based (high-confidence)
NODE_12651_length_1571_cov_0.092391	1571	No	2	1	Low-quality	2.63	AAI-based (high-confidence)
NODE_1266_length_5350_cov_1.444677	5350	No	8	4	Low-quality	2.96	AAI-based (high-confidence)
NODE_1267_length_1447_cov_0.094955	1447	No	2	1	Low-quality	3.7	AAI-based (high-confidence)

NODE_1268_length_2466_cov_0.177440	2466	No	6	2	Low-quality	7.15	AAI-based (high-confidence)
NODE_12691_length_1549_cov_0.030345	1549	No	2	2	Low-quality	2.49	AAI-based (high-confidence)
NODE_127_length_16142_cov_0.186810	16142	No	18	4	Low-quality	9.78	AAI-based (high-confidence)
NODE_127_length_3355_cov_0.143428	3355	No	4	3	Low-quality	6.69	AAI-based (high-confidence)
NODE_1270_length_5957_cov_0.297542	5957	No	5	2	Low-quality	16	AAI-based (high-confidence)
NODE_1271_length_2575_cov_0.179725	2575	No	3	2	Low-quality	5.94	AAI-based (high-confidence)
NODE_12712_length_1394_cov_0.153668	1394	No	2	2	Low-quality	4.39	AAI-based (high-confidence)
NODE_1272_length_4769_cov_0.416060	4769	No	7	1	Low-quality	14.73	AAI-based (high-confidence)
NODE_12724_length_1565_cov_0.062756	1565	No	3	1	Low-quality	4.66	AAI-based (high-confidence)
NODE_12732_length_1545_cov_0.244813	1545	No	5	2	Low-quality	2.88	AAI-based (high-confidence)
NODE_12736_length_1564_cov_0.041638	1564	No	4	1	Low-quality	3.81	AAI-based (high-confidence)
NODE_1275_length_3466_cov_0.515296	3466	No	5	1	Low-quality	6.84	AAI-based (high-confidence)
NODE_12766_length_1542_cov_0.082467	1542	No	4	2	Low-quality	2.87	AAI-based (high-confidence)
NODE_12771_length_1541_cov_0.378641	1541	No	2	1	Low-quality	2.96	AAI-based (high-confidence)
NODE_12772_length_1391_cov_0.071981	1391	No	3	2	Low-quality	2.58	AAI-based (high-confidence)
NODE_1278_length_5928_cov_0.272259	5928	No	5	4	Low-quality	2.78	AAI-based (high-confidence)
NODE_12783_length_1561_cov_0.073187	1561	No	2	2	Low-quality	3.96	AAI-based (high-confidence)
NODE_1279_length_2568_cov_0.109761	2568	No	4	3	Low-quality	4.37	AAI-based (high-confidence)
NODE_1279_length_5336_cov_0.150659	5336	No	6	6	Low-quality	2.99	AAI-based (high-confidence)
NODE_12797_length_1539_cov_0.115972	1539	No	1	1	Low-quality	2.33	AAI-based (high-confidence)
NODE_1280_length_1500_cov_0.121342	1500	No	2	1	Low-quality	4.2	AAI-based (high-confidence)
NODE_1280_length_5328_cov_0.371199	5328	No	7	5	Low-quality	2.97	AAI-based (high-confidence)
NODE_1281_length_1277_cov_0.057725	1277	No	5	3	Low-quality	2.29	AAI-based (high-confidence)
NODE_1281_length_2565_cov_0.433901	2565	No	4	1	Low-quality	7.13	AAI-based (high-confidence)
NODE_12819_length_1538_cov_0.085476	1538	No	4	1	Low-quality	4.57	AAI-based (high-confidence)
NODE_12821_length_1557_cov_0.102195	1557	No	2	2	Low-quality	2.79	AAI-based (high-confidence)
NODE_12838_length_1538_cov_0.287700	1538	No	4	2	Low-quality	4.92	AAI-based (high-confidence)
NODE_12838_length_1555_cov_0.571429	1555	No	2	1	Low-quality	3.75	AAI-based (high-confidence)
NODE_12843_length_1415_cov_0.230243	1415	No	2	2	Low-quality	2.29	AAI-based (high-confidence)
NODE_1285_length_5201_cov_0.200314	5201	No	6	5	Low-quality	11.4	AAI-based (high-confidence)
NODE_12868_length_1533_cov_0.258717	1533	No	1	1	Low-quality	2.4	AAI-based (high-confidence)
NODE_1287_length_1285_cov_0.059022	1285	No	1	1	Low-quality	2.96	AAI-based (high-confidence)
NODE_1288_length_3450_cov_1.190689	3450	No	3	1	Low-quality	6.89	AAI-based (high-confidence)
NODE_12881_length_1414_cov_0.066160	1414	No	2	1	Low-quality	4.38	AAI-based (high-confidence)
NODE_12886_length_1532_cov_0.140265	1532	No	5	1	Low-quality	4.87	AAI-based (high-confidence)
NODE_129_length_2558_cov_0.140301	2558	No	7	2	Low-quality	3.91	AAI-based (high-confidence)
NODE_129_length_3340_cov_0.156125	3340	No	1	1	Low-quality	47.67	AAI-based (high-confidence)
NODE_129_length_6548_cov_0.211506	6548	No	11	6	Low-quality	3.77	AAI-based (high-confidence)
NODE_1290_length_5908_cov_0.141160	5908	No	9	6	Low-quality	11.26	AAI-based (high-confidence)
NODE_12901_length_1551_cov_0.116391	1551	No	1	1	Low-quality	2.56	AAI-based (high-confidence)
NODE_12903_length_1412_cov_0.164509	1412	No	2	2	Low-quality	2.44	AAI-based (high-confidence)
NODE_12912_length_1412_cov_0.086062	1412	No	3	2	Low-quality	3.24	AAI-based (high-confidence)
NODE_12915_length_1379_cov_0.089844	1379	No	1	1	Low-quality	4.13	AAI-based (high-confidence)
NODE_12923_length_1549_cov_0.573793	1549	No	3	1	Low-quality	4.79	AAI-based (high-confidence)
NODE_1293_length_4739_cov_0.182112	4739	No	12	3	Low-quality	13.36	AAI-based (high-confidence)
NODE_1293_length_5902_cov_0.187317	5902	No	10	3	Low-quality	10.01	AAI-based (high-confidence)

NODE_12937_length_1548_cov_0.251898	1548	No	2	2	Low-quality	2.76	AAI-based (high-confidence)
NODE_12952_length_1530_cov_0.062194	1530	No	4	3	Low-quality	3.53	AAI-based (high-confidence)
NODE_1296_length_7332_cov_0.758330	7332	No	11	6	Low-quality	16.86	AAI-based (high-confidence)
NODE_12972_length_1546_cov_0.062889	1546	No	2	1	Low-quality	4.64	AAI-based (high-confidence)
NODE_1298_length_1458_cov_0.243561	1458	No	3	2	Low-quality	2.84	AAI-based (high-confidence)
NODE_12980_length_1408_cov_0.167303	1408	No	3	1	Low-quality	3.48	AAI-based (high-confidence)
NODE_12981_length_1525_cov_0.265778	1525	No	5	1	Low-quality	2.41	AAI-based (high-confidence)
NODE_12989_length_1374_cov_0.103529	1374	No	3	1	Low-quality	4.11	AAI-based (high-confidence)
NODE_12989_length_1525_cov_0.098878	1525	No	3	3	Low-quality	3.59	AAI-based (high-confidence)
NODE_12998_length_1407_cov_0.269113	1407	No	1	1	Low-quality	3.1	AAI-based (high-confidence)
NODE_13_length_5163_cov_0.110585	5163	No	10	4	Low-quality	13.37	AAI-based (high-confidence)
NODE_13_length_6429_cov_0.139810	6429	No	8	5	Low-quality	12.3	AAI-based (high-confidence)
NODE_13005_length_1523_cov_0.382725	1523	No	2	2	Low-quality	3.56	AAI-based (high-confidence)
NODE_1301_length_3429_cov_0.140541	3429	No	7	1	Low-quality	2.08	AAI-based (high-confidence)
NODE_13027_length_1406_cov_0.104055	1406	No	4	2	Low-quality	3.86	AAI-based (high-confidence)
NODE_13051_length_1540_cov_0.108952	1540	No	1	1	Low-quality	2.86	AAI-based (high-confidence)
NODE_1306_length_2545_cov_0.187653	2545	No	6	4	Low-quality	7.82	AAI-based (high-confidence)
NODE_1306_length_5878_cov_0.166984	5878	No	5	3	Low-quality	2.76	AAI-based (high-confidence)
NODE_13073_length_1518_cov_0.193094	1518	No	2	2	Low-quality	3.74	AAI-based (high-confidence)
NODE_13084_length_1536_cov_0.922060	1536	No	4	1	Low-quality	2.84	AAI-based (high-confidence)
NODE_1309_length_3423_cov_0.147714	3423	No	12	1	Low-quality	5.55	AAI-based (high-confidence)
NODE_1309_length_5869_cov_0.088735	5869	No	7	4	Low-quality	13.99	AAI-based (high-confidence)
NODE_131_length_14979_cov_0.360820	14979	No	25	12	Low-quality	17.84	AAI-based (high-confidence)
NODE_1310_length_1276_cov_0.080714	1276	No	4	1	Low-quality	3.74	AAI-based (high-confidence)
NODE_13117_length_1513_cov_0.204385	1513	No	3	1	Low-quality	2.88	AAI-based (high-confidence)
NODE_1312_length_3421_cov_0.128236	3421	No	6	1	Low-quality	6.38	AAI-based (high-confidence)
NODE_1313_length_3420_cov_0.151159	3420	No	9	1	Low-quality	9.51	AAI-based (high-confidence)
NODE_13133_length_1533_cov_0.176430	1533	No	2	1	Low-quality	4.02	AAI-based (high-confidence)
NODE_13135_length_1513_cov_0.762376	1513	No	4	1	Low-quality	4.2	AAI-based (high-confidence)
NODE_13135_length_1533_cov_0.133891	1533	No	3	1	Low-quality	4.78	AAI-based (high-confidence)
NODE_13139_length_1533_cov_0.105300	1533	No	4	2	Low-quality	4.71	AAI-based (high-confidence)
NODE_13169_length_1510_cov_0.700921	1510	No	5	3	Low-quality	2.51	AAI-based (high-confidence)
NODE_13179_length_1530_cov_0.143256	1530	No	4	1	Low-quality	2.89	AAI-based (high-confidence)
NODE_1318_length_1482_cov_0.070860	1482	No	2	2	Low-quality	3.75	AAI-based (high-confidence)
NODE_13188_length_1507_cov_0.128551	1507	No	4	1	Low-quality	3.2	AAI-based (high-confidence)
NODE_13189_length_1359_cov_0.126190	1359	No	3	2	Low-quality	3.74	AAI-based (high-confidence)
NODE_13195_length_1529_cov_0.106993	1529	No	3	1	Low-quality	2.07	AAI-based (high-confidence)
NODE_132_length_3325_cov_0.105704	3325	No	9	3	Low-quality	5.68	AAI-based (high-confidence)
NODE_1320_length_1302_cov_0.052369	1302	No	2	1	Low-quality	3.54	AAI-based (high-confidence)
NODE_1320_length_1443_cov_0.123512	1443	No	1	1	Low-quality	28.33	AAI-based (high-confidence)
NODE_13207_length_1394_cov_0.155985	1394	No	2	1	Low-quality	2.52	AAI-based (high-confidence)
NODE_1322_length_1261_cov_0.151463	1261	No	1	1	Low-quality	26.73	AAI-based (high-confidence)
NODE_1323_length_2533_cov_0.231306	2533	No	4	1	Low-quality	7.85	AAI-based (high-confidence)
NODE_1325_length_5824_cov_0.141310	5824	No	10	5	Low-quality	13.07	AAI-based (high-confidence)
NODE_13250_length_1392_cov_0.095128	1392	No	4	1	Low-quality	3.55	AAI-based (high-confidence)
NODE_1326_length_5123_cov_0.315287	5123	No	11	3	Low-quality	14.8	AAI-based (high-confidence)

NODE_1327_length_5266_cov_0.206309	5266	No	14	4	Low-quality	12.7	AAI-based (high-confidence)
NODE_13271_length_1499_cov_3.400000	1499	No	3	1	Low-quality	4.46	AAI-based (high-confidence)
NODE_1328_length_1271_cov_0.049488	1271	No	2	1	Low-quality	2.36	AAI-based (high-confidence)
NODE_13280_length_1390_cov_0.092177	1390	No	3	1	Low-quality	4.38	AAI-based (high-confidence)
NODE_13285_length_1523_cov_0.046348	1523	No	3	1	Low-quality	2.74	AAI-based (high-confidence)
NODE_13290_length_1522_cov_0.206606	1522	No	4	1	Low-quality	2.8	AAI-based (high-confidence)
NODE_133_length_6356_cov_0.731661	6356	No	12	3	Low-quality	3.67	AAI-based (high-confidence)
NODE_13303_length_1522_cov_0.083626	1522	No	3	1	Low-quality	4.79	AAI-based (high-confidence)
NODE_1331_length_1257_cov_0.056995	1257	No	2	1	Low-quality	2.12	AAI-based (high-confidence)
NODE_1331_length_1477_cov_0.108853	1477	No	4	1	Low-quality	2.38	AAI-based (high-confidence)
NODE_13316_length_1388_cov_0.102405	1388	No	2	2	Low-quality	4.41	AAI-based (high-confidence)
NODE_13327_length_1500_cov_0.094932	1500	No	2	1	Low-quality	4.3	AAI-based (high-confidence)
NODE_13339_length_1499_cov_0.075714	1499	No	2	2	Low-quality	4.76	AAI-based (high-confidence)
NODE_1334_length_4655_cov_0.214004	4655	No	5	2	Low-quality	2.81	AAI-based (high-confidence)
NODE_13340_length_1494_cov_0.105376	1494	No	3	1	Low-quality	4.74	AAI-based (high-confidence)
NODE_13342_length_1498_cov_0.170122	1498	No	3	1	Low-quality	3.39	AAI-based (high-confidence)
NODE_13359_length_1519_cov_0.091549	1519	No	2	1	Low-quality	3.35	AAI-based (high-confidence)
NODE_1336_length_1437_cov_0.166667	1437	No	2	1	Low-quality	2.78	AAI-based (high-confidence)
NODE_1336_length_3392_cov_0.124507	3392	No	6	3	Low-quality	8.19	AAI-based (high-confidence)
NODE_13362_length_1347_cov_0.110577	1347	No	2	1	Low-quality	2.99	AAI-based (high-confidence)
NODE_1337_length_2523_cov_0.160479	2523	No	3	3	Low-quality	7.91	AAI-based (high-confidence)
NODE_13377_length_1496_cov_0.142448	1496	No	2	1	Low-quality	3.24	AAI-based (high-confidence)
NODE_1338_length_5098_cov_0.204241	5098	No	6	3	Low-quality	13.77	AAI-based (high-confidence)
NODE_13393_length_1345_cov_0.065811	1345	No	1	1	Low-quality	3.04	AAI-based (high-confidence)
NODE_13394_length_1495_cov_0.194126	1495	No	4	3	Low-quality	4.49	AAI-based (high-confidence)
NODE_13395_length_1517_cov_0.043018	1517	No	4	2	Low-quality	4.28	AAI-based (high-confidence)
NODE_134_length_6333_cov_0.189766	6333	No	8	7	Low-quality	3.56	AAI-based (high-confidence)
NODE_1340_length_1295_cov_0.115385	1295	No	1	1	Low-quality	18.64	AAI-based (high-confidence)
NODE_13402_length_1383_cov_0.223520	1383	No	5	1	Low-quality	2.52	AAI-based (high-confidence)
NODE_1341_length_1435_cov_0.049401	1435	No	2	2	Low-quality	3.52	AAI-based (high-confidence)
NODE_1342_length_1253_cov_0.128250	1253	No	1	1	Low-quality	18.49	AAI-based (high-confidence)
NODE_1342_length_5240_cov_0.238670	5240	No	7	1	Low-quality	6.41	AAI-based (high-confidence)
NODE_13429_length_1515_cov_0.097458	1515	No	5	1	Low-quality	2.44	AAI-based (high-confidence)
NODE_13442_length_1381_cov_0.141966	1381	No	2	1	Low-quality	2.62	AAI-based (high-confidence)
NODE_13445_length_1514_cov_0.091873	1514	No	3	2	Low-quality	2.56	AAI-based (high-confidence)
NODE_1345_length_3371_cov_0.131724	3371	No	4	3	Low-quality	7.64	AAI-based (high-confidence)
NODE_13455_length_1491_cov_0.637213	1491	No	3	1	Low-quality	2.64	AAI-based (high-confidence)
NODE_1347_length_1433_cov_0.085457	1433	No	1	1	Low-quality	2.33	AAI-based (high-confidence)
NODE_1347_length_1792_cov_0.109864	1792	No	6	1	Low-quality	5.71	AAI-based (high-confidence)
NODE_13476_length_1379_cov_0.421094	1379	No	2	2	Low-quality	2.83	AAI-based (high-confidence)
NODE_1349_length_3366_cov_0.174166	3366	No	12	4	Low-quality	10.98	AAI-based (high-confidence)
NODE_1349_length_5229_cov_0.406628	5229	No	3	1	Low-quality	16.61	AAI-based (high-confidence)
NODE_13491_length_1489_cov_0.041727	1489	No	4	1	Low-quality	4.46	AAI-based (high-confidence)
NODE_1350_length_3366_cov_0.073156	3366	No	5	3	Low-quality	10.36	AAI-based (high-confidence)
NODE_1351_length_4622_cov_0.342693	4622	No	5	3	Low-quality	14.78	AAI-based (high-confidence)
NODE_13513_length_1487_cov_0.121037	1487	No	2	1	Low-quality	3.28	AAI-based (high-confidence)

NODE_1353_length_1409_cov_0.175573	1409	No	1	1	Low-quality	22.07	AAI-based (high-confidence)
NODE_13533_length_1473_cov_2.862445	1473	No	3	1	Low-quality	4.48	AAI-based (high-confidence)
NODE_1354_length_2510_cov_0.164662	2510	No	2	1	Low-quality	2.92	AAI-based (high-confidence)
NODE_13540_length_1507_cov_0.181108	1507	No	3	1	Low-quality	4.6	AAI-based (high-confidence)
NODE_13551_length_1484_cov_0.605776	1484	No	6	1	Low-quality	4.73	AAI-based (high-confidence)
NODE_1356_length_1246_cov_0.108980	1246	No	5	1	Low-quality	2.62	AAI-based (high-confidence)
NODE_1356_length_3362_cov_0.115231	3362	No	5	2	Low-quality	2.22	AAI-based (high-confidence)
NODE_13570_length_1505_cov_0.584637	1505	No	3	1	Low-quality	4.65	AAI-based (high-confidence)
NODE_13574_length_1332_cov_0.094891	1332	No	3	1	Low-quality	2.95	AAI-based (high-confidence)
NODE_13586_length_1504_cov_0.071886	1504	No	1	1	Low-quality	5	AAI-based (high-confidence)
NODE_13599_length_1330_cov_0.149472	1330	No	2	1	Low-quality	3.95	AAI-based (high-confidence)
NODE_136_length_14741_cov_0.264991	14741	No	26	12	Low-quality	46.84	AAI-based (high-confidence)
NODE_136_length_3282_cov_0.142633	3282	No	5	2	Low-quality	10.22	AAI-based (high-confidence)
NODE_13614_length_1502_cov_0.105488	1502	No	1	1	Low-quality	2.97	AAI-based (high-confidence)
NODE_1364_length_4596_cov_0.132978	4596	No	5	1	Low-quality	8.67	AAI-based (high-confidence)
NODE_13647_length_1478_cov_0.451777	1478	No	2	2	Low-quality	2.32	AAI-based (high-confidence)
NODE_13654_length_1499_cov_0.081429	1499	No	2	2	Low-quality	3.77	AAI-based (high-confidence)
NODE_13662_length_1326_cov_0.176854	1326	No	5	1	Low-quality	3.35	AAI-based (high-confidence)
NODE_13668_length_1498_cov_0.095783	1498	No	3	1	Low-quality	2.79	AAI-based (high-confidence)
NODE_1367_length_5040_cov_0.120826	5040	No	7	4	Low-quality	13.54	AAI-based (high-confidence)
NODE_1367_length_5197_cov_0.496077	5197	No	7	1	Low-quality	13.93	AAI-based (high-confidence)
NODE_13680_length_1476_cov_0.314452	1476	No	4	2	Low-quality	3.56	AAI-based (high-confidence)
NODE_1369_length_1286_cov_0.102780	1286	No	2	1	Low-quality	2.79	AAI-based (high-confidence)
NODE_1369_length_1406_cov_0.040551	1406	No	2	1	Low-quality	3.15	AAI-based (high-confidence)
NODE_137_length_2873_cov_0.118962	2873	No	2	2	Low-quality	5.63	AAI-based (high-confidence)
NODE_137_length_6301_cov_0.332312	6301	No	9	3	Low-quality	15.78	AAI-based (high-confidence)
NODE_13713_length_1364_cov_0.192095	1364	No	5	1	Low-quality	4.17	AAI-based (high-confidence)
NODE_13715_length_1458_cov_0.192789	1458	No	2	2	Low-quality	2.98	AAI-based (high-confidence)
NODE_13716_length_1364_cov_0.147826	1364	No	4	3	Low-quality	2.61	AAI-based (high-confidence)
NODE_1372_length_1241_cov_0.117338	1241	No	1	1	Low-quality	3.44	AAI-based (high-confidence)
NODE_1373_length_1285_cov_0.109612	1285	No	1	1	Low-quality	2.14	AAI-based (high-confidence)
NODE_1373_length_5182_cov_0.169585	5182	No	2	1	Low-quality	4.89	AAI-based (high-confidence)
NODE_1375_length_5700_cov_0.148366	5700	No	6	2	Low-quality	17.51	AAI-based (high-confidence)
NODE_13752_length_1473_cov_0.072052	1473	No	4	2	Low-quality	3.44	AAI-based (high-confidence)
NODE_13758_length_1472_cov_0.141296	1472	No	5	1	Low-quality	3.35	AAI-based (high-confidence)
NODE_13760_length_1493_cov_0.062410	1493	No	2	2	Low-quality	2.74	AAI-based (high-confidence)
NODE_13769_length_1361_cov_0.232171	1361	No	2	2	Low-quality	4.19	AAI-based (high-confidence)
NODE_13787_length_1361_cov_0.058637	1361	No	3	1	Low-quality	2.19	AAI-based (high-confidence)
NODE_13799_length_1490_cov_0.138030	1490	No	3	1	Low-quality	2.69	AAI-based (high-confidence)
NODE_13805_length_1490_cov_0.112868	1490	No	2	1	Low-quality	2.77	AAI-based (high-confidence)
NODE_1381_length_3324_cov_0.087132	3324	No	5	3	Low-quality	5.73	AAI-based (high-confidence)
NODE_1381_length_4559_cov_0.188565	4559	No	7	3	Low-quality	7.53	AAI-based (high-confidence)
NODE_13811_length_1315_cov_0.180099	1315	No	3	1	Low-quality	4.07	AAI-based (high-confidence)
NODE_1382_length_1236_cov_0.095866	1236	No	4	1	Low-quality	2.01	AAI-based (high-confidence)
NODE_1382_length_5162_cov_0.210942	5162	No	2	1	Low-quality	6.32	AAI-based (high-confidence)
NODE_1382_length_5692_cov_0.142857	5692	No	8	6	Low-quality	3.19	AAI-based (high-confidence)

NODE_1383_length_5690_cov_0.176891	5690	No	9	3	Low-quality	14.57	AAI-based (high-confidence)
NODE_1384_length_5157_cov_0.249901	5157	No	7	5	Low-quality	2.88	AAI-based (high-confidence)
NODE_13845_length_1488_cov_0.056875	1488	No	2	1	Low-quality	5.84	AAI-based (high-confidence)
NODE_13847_length_1313_cov_0.361614	1313	No	1	1	Low-quality	2.17	AAI-based (high-confidence)
NODE_1385_length_4553_cov_0.187023	4553	No	6	5	Low-quality	2.53	AAI-based (high-confidence)
NODE_1388_length_5148_cov_0.112101	5148	No	8	5	Low-quality	16.19	AAI-based (high-confidence)
NODE_13888_length_1485_cov_0.078644	1485	No	3	1	Low-quality	2.84	AAI-based (high-confidence)
NODE_1389_length_1196_cov_0.064722	1196	No	3	3	Low-quality	2.72	AAI-based (high-confidence)
NODE_13897_length_1484_cov_0.180505	1484	No	3	2	Low-quality	3.54	AAI-based (high-confidence)
NODE_139_length_3142_cov_0.185672	3142	No	1	1	Low-quality	47.83	AAI-based (high-confidence)
NODE_1390_length_4543_cov_0.182043	4543	No	3	1	Low-quality	7.06	AAI-based (high-confidence)
NODE_13904_length_1484_cov_0.101805	1484	No	2	2	Low-quality	2.43	AAI-based (high-confidence)
NODE_13905_length_1354_cov_0.262151	1354	No	4	1	Low-quality	2.87	AAI-based (high-confidence)
NODE_1391_length_5136_cov_0.254120	5136	No	9	3	Low-quality	8.73	AAI-based (high-confidence)
NODE_13923_length_1353_cov_0.364434	1353	No	4	1	Low-quality	4.41	AAI-based (high-confidence)
NODE_13924_length_1353_cov_0.283094	1353	No	4	2	Low-quality	1.95	AAI-based (high-confidence)
NODE_13926_length_1482_cov_0.311641	1482	No	1	1	Low-quality	2.32	AAI-based (high-confidence)
NODE_1393_length_5668_cov_0.352128	5668	No	5	2	Low-quality	12.6	AAI-based (high-confidence)
NODE_13940_length_1352_cov_0.079808	1352	No	3	1	Low-quality	4.57	AAI-based (high-confidence)
NODE_13954_length_1351_cov_0.095048	1351	No	1	1	Low-quality	2.52	AAI-based (high-confidence)
NODE_13959_length_1351_cov_0.029553	1351	No	1	1	Low-quality	2.32	AAI-based (high-confidence)
NODE_13959_length_1460_cov_0.063189	1460	No	2	2	Low-quality	2.8	AAI-based (high-confidence)
NODE_13965_length_1480_cov_0.347574	1480	No	4	1	Low-quality	2.53	AAI-based (high-confidence)
NODE_13975_length_1349_cov_0.389600	1349	No	3	2	Low-quality	2.42	AAI-based (high-confidence)
NODE_1398_length_5662_cov_0.285098	5662	No	6	2	Low-quality	9.35	AAI-based (high-confidence)
NODE_1399_length_2471_cov_0.114250	2471	No	3	2	Low-quality	10.03	AAI-based (high-confidence)
NODE_14_length_5581_cov_0.127508	5581	No	13	6	Low-quality	8.93	AAI-based (high-confidence)
NODE_14_length_6187_cov_0.109560	6187	No	9	3	Low-quality	19.31	AAI-based (high-confidence)
NODE_140_length_3352_cov_0.071626	3352	No	6	4	Low-quality	8.53	AAI-based (high-confidence)
NODE_1400_length_1277_cov_0.050085	1277	No	2	1	Low-quality	3.93	AAI-based (high-confidence)
NODE_1400_length_1398_cov_0.592764	1398	No	2	1	Low-quality	2.83	AAI-based (high-confidence)
NODE_14007_length_1478_cov_0.130529	1478	No	3	3	Low-quality	2.73	AAI-based (high-confidence)
NODE_14018_length_1347_cov_0.115385	1347	No	4	2	Low-quality	4.03	AAI-based (high-confidence)
NODE_14019_length_1455_cov_0.544248	1455	No	4	1	Low-quality	2.53	AAI-based (high-confidence)
NODE_1402_length_1101_cov_0.048902	1101	No	2	2	Low-quality	3.39	AAI-based (high-confidence)
NODE_1402_length_4532_cov_0.092714	4532	No	5	3	Low-quality	2.78	AAI-based (high-confidence)
NODE_14022_length_1477_cov_0.614659	1477	No	3	1	Low-quality	2.31	AAI-based (high-confidence)
NODE_14034_length_1454_cov_0.261255	1454	No	5	2	Low-quality	4.31	AAI-based (high-confidence)
NODE_14041_length_1454_cov_0.103321	1454	No	3	1	Low-quality	2.44	AAI-based (high-confidence)
NODE_14049_length_1453_cov_1.073117	1453	No	1	1	Low-quality	4.45	AAI-based (high-confidence)
NODE_1405_length_5645_cov_0.137577	5645	No	8	5	Low-quality	5.04	AAI-based (high-confidence)
NODE_14066_length_1453_cov_0.076809	1453	No	4	1	Low-quality	3.04	AAI-based (high-confidence)
NODE_14076_length_1344_cov_0.419277	1344	No	2	1	Low-quality	2.11	AAI-based (high-confidence)
NODE_14087_length_1451_cov_0.371302	1451	No	3	1	Low-quality	4.47	AAI-based (high-confidence)
NODE_1409_length_1245_cov_0.064572	1245	No	4	3	Low-quality	3.41	AAI-based (high-confidence)
NODE_14092_length_1344_cov_0.073092	1344	No	3	1	Low-quality	2.17	AAI-based (high-confidence)

NODE_141_length_6257_cov_0.196005	6257	No	10	5	Low-quality	6.01	AAI-based (high-confidence)
NODE_141_length_9825_cov_0.181986	9825	No	12	4	Low-quality	16.43	AAI-based (high-confidence)
NODE_1410_length_2461_cov_0.574513	2461	No	4	1	Low-quality	7.62	AAI-based (high-confidence)
NODE_14115_length_1449_cov_0.203704	1449	No	2	1	Low-quality	2.32	AAI-based (high-confidence)
NODE_14124_length_1471_cov_0.053207	1471	No	1	1	Low-quality	3.43	AAI-based (high-confidence)
NODE_14126_length_1470_cov_0.297593	1470	No	5	3	Low-quality	3.01	AAI-based (high-confidence)
NODE_1413_length_1273_cov_0.037479	1273	No	1	1	Low-quality	2.38	AAI-based (high-confidence)
NODE_14133_length_1448_cov_0.670867	1448	No	2	2	Low-quality	2.9	AAI-based (high-confidence)
NODE_14137_length_1341_cov_0.105475	1341	No	1	1	Low-quality	3.49	AAI-based (high-confidence)
NODE_14138_length_1469_cov_0.240876	1469	No	2	1	Low-quality	2.51	AAI-based (high-confidence)
NODE_1414_length_3276_cov_0.076172	3276	No	7	1	Low-quality	3.33	AAI-based (high-confidence)
NODE_14145_length_1469_cov_0.139416	1469	No	4	2	Low-quality	4.73	AAI-based (high-confidence)
NODE_1415_length_1404_cov_0.137165	1404	No	1	1	Low-quality	21.27	AAI-based (high-confidence)
NODE_1415_length_2456_cov_0.109037	2456	No	4	1	Low-quality	6.08	AAI-based (high-confidence)
NODE_14153_length_1469_cov_0.100730	1469	No	4	1	Low-quality	2.35	AAI-based (high-confidence)
NODE_14169_length_1339_cov_0.154839	1339	No	2	1	Low-quality	2.48	AAI-based (high-confidence)
NODE_1417_length_4496_cov_0.127132	4496	No	4	1	Low-quality	7.86	AAI-based (high-confidence)
NODE_14183_length_1468_cov_0.079620	1468	No	2	1	Low-quality	2.42	AAI-based (high-confidence)
NODE_14208_length_1443_cov_1.075893	1443	No	3	1	Low-quality	4.08	AAI-based (high-confidence)
NODE_1423_length_2337_cov_0.116175	2337	No	6	1	Low-quality	3.78	AAI-based (high-confidence)
NODE_1424_length_1223_cov_0.142349	1223	No	1	1	Low-quality	18.42	AAI-based (high-confidence)
NODE_1426_length_3259_cov_0.259177	3259	No	4	3	Low-quality	5.37	AAI-based (high-confidence)
NODE_14278_length_1461_cov_0.278267	1461	No	4	1	Low-quality	2.84	AAI-based (high-confidence)
NODE_1431_length_1265_cov_0.066895	1265	No	1	1	Low-quality	2.1	AAI-based (high-confidence)
NODE_14331_length_1331_cov_0.099838	1331	No	2	2	Low-quality	2.21	AAI-based (high-confidence)
NODE_14332_length_1283_cov_0.199324	1283	No	1	1	Low-quality	4.01	AAI-based (high-confidence)
NODE_14333_length_1331_cov_0.090909	1331	No	3	2	Low-quality	2.64	AAI-based (high-confidence)
NODE_1435_length_4465_cov_0.518094	4465	No	12	4	Low-quality	7.16	AAI-based (high-confidence)
NODE_1437_length_1385_cov_0.054432	1385	No	3	1	Low-quality	3.77	AAI-based (high-confidence)
NODE_1437_length_5574_cov_0.381735	5574	No	12	3	Low-quality	9.03	AAI-based (high-confidence)
NODE_1438_length_1761_cov_0.080626	1761	No	2	1	Low-quality	2.96	AAI-based (high-confidence)
NODE_1439_length_3246_cov_0.193835	3246	No	7	1	Low-quality	5.39	AAI-based (high-confidence)
NODE_14397_length_1327_cov_0.049674	1327	No	4	1	Low-quality	2.25	AAI-based (high-confidence)
NODE_144_length_15568_cov_0.583166	15568	No	22	7	Low-quality	43.86	AAI-based (high-confidence)
NODE_144_length_2827_cov_0.086144	2827	No	4	2	Low-quality	8.26	AAI-based (high-confidence)
NODE_14401_length_1326_cov_0.193969	1326	No	2	1	Low-quality	3.95	AAI-based (high-confidence)
NODE_14405_length_1430_cov_0.131480	1430	No	3	1	Low-quality	1.99	AAI-based (high-confidence)
NODE_1442_length_5038_cov_0.290747	5038	No	7	1	Low-quality	2.27	AAI-based (high-confidence)
NODE_1443_length_1262_cov_0.075666	1262	No	3	2	Low-quality	2.34	AAI-based (high-confidence)
NODE_1444_length_3239_cov_0.231847	3239	No	5	2	Low-quality	13.2	AAI-based (high-confidence)
NODE_14443_length_1396_cov_0.090208	1396	No	1	1	Low-quality	3.15	AAI-based (high-confidence)
NODE_1445_length_5558_cov_0.459791	5558	No	10	4	Low-quality	3.44	AAI-based (high-confidence)
NODE_14475_length_1275_cov_0.221939	1275	No	3	2	Low-quality	2.9	AAI-based (high-confidence)
NODE_14482_length_1322_cov_0.098937	1322	No	5	2	Low-quality	2.1	AAI-based (high-confidence)
NODE_1449_length_1429_cov_0.070677	1429	No	5	1	Low-quality	4.34	AAI-based (high-confidence)
NODE_1449_length_5554_cov_0.658845	5554	No	4	4	Low-quality	9.18	AAI-based (high-confidence)

NODE_14493_length_1392_cov_0.199536	1392	No	3	1	Low-quality	3.95	AAI-based (high-confidence)
NODE_1450_length_1379_cov_0.110937	1379	No	2	1	Low-quality	2.3	AAI-based (high-confidence)
NODE_1450_length_2436_cov_0.070175	2436	No	3	1	Low-quality	4.18	AAI-based (high-confidence)
NODE_14500_length_1447_cov_0.717359	1447	No	3	1	Low-quality	4.48	AAI-based (high-confidence)
NODE_14529_length_1423_cov_0.055891	1423	No	2	1	Low-quality	4.99	AAI-based (high-confidence)
NODE_1453_length_1216_cov_0.035810	1216	No	1	1	Low-quality	3.34	AAI-based (high-confidence)
NODE_14547_length_1445_cov_0.170877	1445	No	2	1	Low-quality	3.14	AAI-based (high-confidence)
NODE_1456_length_3224_cov_0.163840	3224	No	2	1	Low-quality	2.04	AAI-based (high-confidence)
NODE_14576_length_1443_cov_0.238839	1443	No	4	1	Low-quality	3.8	AAI-based (high-confidence)
NODE_1458_length_2433_cov_0.094687	2433	No	3	1	Low-quality	7.58	AAI-based (high-confidence)
NODE_146_length_6171_cov_0.170619	6171	No	11	2	Low-quality	10.2	AAI-based (high-confidence)
NODE_1461_length_5002_cov_0.187028	5002	No	5	1	Low-quality	14.5	AAI-based (high-confidence)
NODE_1461_length_6781_cov_2.423077	6781	No	7	3	Low-quality	19.33	AAI-based (high-confidence)
NODE_14611_length_1266_cov_0.113111	1266	No	2	1	Low-quality	2.69	AAI-based (high-confidence)
NODE_1462_length_1210_cov_0.062106	1210	No	2	1	Low-quality	3.09	AAI-based (high-confidence)
NODE_1462_length_5522_cov_0.125576	5522	No	13	5	Low-quality	17.61	AAI-based (high-confidence)
NODE_14627_length_1416_cov_0.159453	1416	No	4	1	Low-quality	4.3	AAI-based (high-confidence)
NODE_14631_length_1315_cov_0.078947	1315	No	2	2	Low-quality	2.41	AAI-based (high-confidence)
NODE_14640_length_1439_cov_0.184328	1439	No	3	1	Low-quality	4.01	AAI-based (high-confidence)
NODE_14651_length_1313_cov_0.162273	1313	No	3	1	Low-quality	4.13	AAI-based (high-confidence)
NODE_1467_length_5502_cov_0.317231	5502	No	7	2	Low-quality	3.6	AAI-based (high-confidence)
NODE_1468_length_1387_cov_0.112578	1387	No	3	2	Low-quality	2.4	AAI-based (high-confidence)
NODE_1469_length_1226_cov_0.055901	1226	No	3	1	Low-quality	3.29	AAI-based (high-confidence)
NODE_1469_length_1254_cov_0.084848	1254	No	3	2	Low-quality	4.27	AAI-based (high-confidence)
NODE_1469_length_4994_cov_0.403677	4994	No	9	4	Low-quality	8.26	AAI-based (high-confidence)
NODE_14692_length_1436_cov_0.102468	1436	No	3	2	Low-quality	4.3	AAI-based (high-confidence)
NODE_147_length_15391_cov_0.215407	15391	No	9	5	Low-quality	20.55	AAI-based (high-confidence)
NODE_147_length_3207_cov_0.151223	3207	No	2	1	Low-quality	47.58	AAI-based (high-confidence)
NODE_14701_length_1378_cov_0.155590	1378	No	2	1	Low-quality	2.56	AAI-based (high-confidence)
NODE_14708_length_1260_cov_0.180017	1260	No	2	1	Low-quality	3.89	AAI-based (high-confidence)
NODE_14712_length_1311_cov_0.046205	1311	No	1	1	Low-quality	2.98	AAI-based (high-confidence)
NODE_14730_length_1259_cov_0.089655	1259	No	3	1	Low-quality	3.96	AAI-based (high-confidence)
NODE_14747_length_1309_cov_0.079339	1309	No	1	1	Low-quality	2.19	AAI-based (high-confidence)
NODE_1475_length_3199_cov_0.302258	3199	No	9	1	Low-quality	5.18	AAI-based (high-confidence)
NODE_1475_length_5484_cov_0.641226	5484	No	6	3	Low-quality	9.42	AAI-based (high-confidence)
NODE_14764_length_1408_cov_0.090145	1408	No	2	2	Low-quality	2.59	AAI-based (high-confidence)
NODE_14765_length_1408_cov_0.075630	1408	No	3	3	Low-quality	4.16	AAI-based (high-confidence)
NODE_14776_length_1407_cov_0.115443	1407	No	5	2	Low-quality	2.57	AAI-based (high-confidence)
NODE_1478_length_1745_cov_0.154313	1745	No	3	1	Low-quality	3.29	AAI-based (high-confidence)
NODE_1479_length_4849_cov_0.248211	4849	No	8	3	Low-quality	2.44	AAI-based (high-confidence)
NODE_14796_length_1429_cov_0.117293	1429	No	4	2	Low-quality	4.28	AAI-based (high-confidence)
NODE_14800_length_1429_cov_0.093233	1429	No	4	3	Low-quality	2.78	AAI-based (high-confidence)
NODE_14807_length_1428_cov_0.141460	1428	No	1	1	Low-quality	2.24	AAI-based (high-confidence)
NODE_1481_length_3196_cov_1.547950	3196	No	5	1	Low-quality	8.63	AAI-based (high-confidence)
NODE_14837_length_1403_cov_0.366564	1403	No	4	1	Low-quality	3.5	AAI-based (high-confidence)
NODE_1484_length_4393_cov_0.333256	4393	No	5	3	Low-quality	13.69	AAI-based (high-confidence)

NODE_1485_length_4392_cov_0.156301	4392	No	3	2	Low-quality	2.45	AAI-based (high-confidence)
NODE_14864_length_1402_cov_0.112817	1402	No	3	2	Low-quality	2.65	AAI-based (high-confidence)
NODE_14876_length_1249_cov_0.287826	1249	No	2	1	Low-quality	3.77	AAI-based (high-confidence)
NODE_1488_length_1364_cov_0.071146	1364	No	2	1	Low-quality	4.38	AAI-based (high-confidence)
NODE_1488_length_2293_cov_0.286691	2293	No	5	2	Low-quality	7.48	AAI-based (high-confidence)
NODE_1488_length_4835_cov_0.177154	4835	No	7	2	Low-quality	7.09	AAI-based (high-confidence)
NODE_1489_length_1378_cov_0.114152	1378	No	3	1	Low-quality	4.13	AAI-based (high-confidence)
NODE_14892_length_1301_cov_0.084859	1301	No	3	1	Low-quality	3.94	AAI-based (high-confidence)
NODE_149_length_6134_cov_0.125766	6134	No	6	6	Low-quality	11.43	AAI-based (high-confidence)
NODE_1490_length_1378_cov_0.110242	1378	No	1	1	Low-quality	21.5	AAI-based (high-confidence)
NODE_1490_length_4969_cov_0.180698	4969	No	4	2	Low-quality	9.24	AAI-based (high-confidence)
NODE_14900_length_1300_cov_0.170691	1300	No	3	1	Low-quality	2.47	AAI-based (high-confidence)
NODE_1491_length_1201_cov_0.092559	1201	No	1	1	Low-quality	26.04	AAI-based (high-confidence)
NODE_14911_length_1399_cov_0.219231	1399	No	2	2	Low-quality	3.49	AAI-based (high-confidence)
NODE_1492_length_4377_cov_0.156849	4377	No	8	6	Low-quality	12.03	AAI-based (high-confidence)
NODE_1493_length_4965_cov_0.190917	4965	No	4	1	Low-quality	7.95	AAI-based (high-confidence)
NODE_14937_length_1246_cov_0.077594	1246	No	3	1	Low-quality	3.34	AAI-based (high-confidence)
NODE_14944_length_1398_cov_0.046189	1398	No	1	1	Low-quality	2.32	AAI-based (high-confidence)
NODE_14974_length_1396_cov_0.127217	1396	No	2	1	Low-quality	3.28	AAI-based (high-confidence)
NODE_14984_length_1418_cov_0.062926	1418	No	3	2	Low-quality	3.5	AAI-based (high-confidence)
NODE_15_length_6129_cov_0.151078	6129	No	6	1	Low-quality	17.84	AAI-based (high-confidence)
NODE_15_length_7260_cov_0.126519	7260	No	15	4	Low-quality	22.25	AAI-based (high-confidence)
NODE_150_length_15244_cov_0.341499	15244	No	27	13	Low-quality	47.44	AAI-based (high-confidence)
NODE_150_length_3189_cov_0.110356	3189	No	5	3	Low-quality	5.58	AAI-based (high-confidence)
NODE_15007_length_1395_cov_0.059414	1395	No	3	2	Low-quality	2.23	AAI-based (high-confidence)
NODE_15007_length_1416_cov_0.214123	1416	No	3	1	Low-quality	4.43	AAI-based (high-confidence)
NODE_15025_length_1415_cov_0.287234	1415	No	4	2	Low-quality	4.19	AAI-based (high-confidence)
NODE_15038_length_1240_cov_0.106924	1240	No	2	1	Low-quality	3.79	AAI-based (high-confidence)
NODE_1504_length_4364_cov_0.166002	4364	No	4	4	Low-quality	2.48	AAI-based (high-confidence)
NODE_1505_length_4944_cov_0.189061	4944	No	5	3	Low-quality	12.09	AAI-based (high-confidence)
NODE_15055_length_1391_cov_0.438854	1391	No	2	1	Low-quality	2.18	AAI-based (high-confidence)
NODE_1506_length_3162_cov_0.249755	3162	No	8	2	Low-quality	8.2	AAI-based (high-confidence)
NODE_15065_length_1414_cov_0.025856	1414	No	2	2	Low-quality	2.44	AAI-based (high-confidence)
NODE_15068_length_1413_cov_0.276256	1413	No	1	1	Low-quality	4.28	AAI-based (high-confidence)
NODE_15071_length_1391_cov_0.104489	1391	No	3	1	Low-quality	4.33	AAI-based (high-confidence)
NODE_15075_length_1391_cov_0.072755	1391	No	3	1	Low-quality	4.03	AAI-based (high-confidence)
NODE_15099_length_1237_cov_0.089631	1237	No	4	1	Low-quality	2.32	AAI-based (high-confidence)
NODE_15099_length_1290_cov_0.167926	1290	No	2	1	Low-quality	3.92	AAI-based (high-confidence)
NODE_15109_length_1290_cov_0.100756	1290	No	3	1	Low-quality	2.09	AAI-based (high-confidence)
NODE_1512_length_2276_cov_0.260910	2276	No	8	1	Low-quality	6.52	AAI-based (high-confidence)
NODE_1512_length_5408_cov_0.094933	5408	No	9	3	Low-quality	16.86	AAI-based (high-confidence)
NODE_15136_length_1408_cov_0.304813	1408	No	1	1	Low-quality	2.25	AAI-based (high-confidence)
NODE_1515_length_6622_cov_0.539629	6622	No	17	5	Low-quality	21.68	AAI-based (high-confidence)
NODE_15158_length_1385_cov_0.111975	1385	No	2	1	Low-quality	2.58	AAI-based (high-confidence)
NODE_15164_length_1384_cov_0.261479	1384	No	2	2	Low-quality	2.52	AAI-based (high-confidence)
NODE_15169_length_1384_cov_0.112062	1384	No	4	2	Low-quality	3.01	AAI-based (high-confidence)

NODE_15173_length_1384_cov_0.081712	1384	No	4	2	Low-quality	3.25	AAI-based (high-confidence)
NODE_15176_length_1407_cov_0.011468	1407	No	4	2	Low-quality	2.23	AAI-based (high-confidence)
NODE_1518_length_4783_cov_0.139197	4783	No	4	2	Low-quality	15	AAI-based (high-confidence)
NODE_15180_length_1383_cov_0.557632	1383	No	2	2	Low-quality	2.09	AAI-based (high-confidence)
NODE_152_length_15141_cov_0.272038	15141	No	19	7	Low-quality	24.02	AAI-based (high-confidence)
NODE_152_length_2776_cov_0.175943	2776	No	1	1	Low-quality	49.77	AAI-based (high-confidence)
NODE_1520_length_1192_cov_0.115279	1192	No	1	1	Low-quality	18.78	AAI-based (high-confidence)
NODE_1520_length_2391_cov_0.186736	2391	No	4	2	Low-quality	2.16	AAI-based (high-confidence)
NODE_1520_length_4927_cov_0.631317	4927	No	6	3	Low-quality	2.88	AAI-based (high-confidence)
NODE_15200_length_1382_cov_0.279813	1382	No	6	1	Low-quality	2.18	AAI-based (high-confidence)
NODE_1522_length_1185_cov_0.060773	1185	No	3	3	Low-quality	1.99	AAI-based (high-confidence)
NODE_1522_length_2390_cov_0.142732	2390	No	5	1	Low-quality	8.2	AAI-based (high-confidence)
NODE_1522_length_4927_cov_0.211060	4927	No	3	3	Low-quality	8.93	AAI-based (high-confidence)
NODE_15221_length_1404_cov_0.082759	1404	No	2	1	Low-quality	2.63	AAI-based (high-confidence)
NODE_15224_length_1403_cov_0.746933	1403	No	1	1	Low-quality	2.31	AAI-based (high-confidence)
NODE_1524_length_1353_cov_0.180223	1353	No	1	1	Low-quality	13.74	AAI-based (high-confidence)
NODE_1524_length_5392_cov_0.127149	5392	No	6	2	Low-quality	14.87	AAI-based (high-confidence)
NODE_15252_length_1402_cov_0.106677	1402	No	2	2	Low-quality	2.71	AAI-based (high-confidence)
NODE_15259_length_1227_cov_0.065603	1227	No	4	1	Low-quality	2.2	AAI-based (high-confidence)
NODE_15262_length_1401_cov_0.552995	1401	No	4	1	Low-quality	2.62	AAI-based (high-confidence)
NODE_1527_length_1190_cov_0.067828	1190	No	2	1	Low-quality	2.76	AAI-based (high-confidence)
NODE_15270_length_1283_cov_0.079392	1283	No	3	1	Low-quality	3.96	AAI-based (high-confidence)
NODE_15288_length_1401_cov_0.036866	1401	No	2	1	Low-quality	2.35	AAI-based (high-confidence)
NODE_153_length_6063_cov_0.150905	6063	No	8	7	Low-quality	15.77	AAI-based (high-confidence)
NODE_15321_length_1399_cov_0.092308	1399	No	3	1	Low-quality	2.44	AAI-based (high-confidence)
NODE_15326_length_1332_cov_0.274939	1332	No	2	1	Low-quality	2.47	AAI-based (high-confidence)
NODE_15327_length_1280_cov_0.101609	1280	No	2	1	Low-quality	3.41	AAI-based (high-confidence)
NODE_15329_length_1224_cov_0.068444	1224	No	5	1	Low-quality	3.09	AAI-based (high-confidence)
NODE_1534_length_1164_cov_0.074178	1164	No	1	1	Low-quality	27.03	AAI-based (high-confidence)
NODE_15348_length_1279_cov_0.112712	1279	No	3	1	Low-quality	3.48	AAI-based (high-confidence)
NODE_15351_length_1374_cov_0.120000	1374	No	4	2	Low-quality	3.33	AAI-based (high-confidence)
NODE_15362_length_1279_cov_0.063559	1279	No	1	1	Low-quality	3.08	AAI-based (high-confidence)
NODE_15365_length_1330_cov_0.118603	1330	No	1	1	Low-quality	2.74	AAI-based (high-confidence)
NODE_15365_length_1396_cov_0.202005	1396	No	5	1	Low-quality	3.37	AAI-based (high-confidence)
NODE_1537_length_4893_cov_0.755528	4893	No	4	2	Low-quality	8.41	AAI-based (high-confidence)
NODE_15371_length_1396_cov_0.089437	1396	No	2	1	Low-quality	3.7	AAI-based (high-confidence)
NODE_15378_length_1395_cov_0.537037	1395	No	1	1	Low-quality	2.95	AAI-based (high-confidence)
NODE_1538_length_1230_cov_0.076923	1230	No	5	3	Low-quality	2.53	AAI-based (high-confidence)
NODE_1538_length_2380_cov_0.085489	2380	No	4	2	Low-quality	5.14	AAI-based (high-confidence)
NODE_1539_length_3122_cov_0.138273	3122	No	5	2	Low-quality	2.81	AAI-based (high-confidence)
NODE_1539_length_4889_cov_0.289979	4889	No	4	2	Low-quality	2.2	AAI-based (high-confidence)
NODE_15392_length_1371_cov_0.194182	1371	No	2	1	Low-quality	2.98	AAI-based (high-confidence)
NODE_154_length_13585_cov_0.497775	13585	No	24	4	Low-quality	22.81	AAI-based (high-confidence)
NODE_154_length_6049_cov_0.245042	6049	No	7	5	Low-quality	3.42	AAI-based (high-confidence)
NODE_15405_length_1371_cov_0.060535	1371	No	1	1	Low-quality	2.57	AAI-based (high-confidence)
NODE_15406_length_1327_cov_0.114821	1327	No	2	2	Low-quality	3.3	AAI-based (high-confidence)

NODE_15422_length_1393_cov_0.064915	1393	No	4	2	Low-quality	4.1	AAI-based (high-confidence)
NODE_15423_length_1369_cov_0.233071	1369	No	2	1	Low-quality	4.33	AAI-based (high-confidence)
NODE_1543_length_1178_cov_0.061168	1178	No	2	1	Low-quality	3.61	AAI-based (high-confidence)
NODE_15430_length_1369_cov_0.114961	1369	No	2	2	Low-quality	2.49	AAI-based (high-confidence)
NODE_15432_length_1274_cov_0.387234	1274	No	5	2	Low-quality	3.88	AAI-based (high-confidence)
NODE_1544_length_2257_cov_0.084801	2257	No	6	2	Low-quality	6.82	AAI-based (high-confidence)
NODE_1547_length_4878_cov_0.321825	4878	No	3	2	Low-quality	2.28	AAI-based (high-confidence)
NODE_15472_length_1390_cov_0.108443	1390	No	3	1	Low-quality	3.34	AAI-based (high-confidence)
NODE_15482_length_1389_cov_0.155039	1389	No	2	2	Low-quality	3.03	AAI-based (high-confidence)
NODE_155_length_2757_cov_0.271633	2757	No	1	1	Low-quality	39.42	AAI-based (high-confidence)
NODE_15516_length_1214_cov_0.105830	1214	No	1	1	Low-quality	3.4	AAI-based (high-confidence)
NODE_15519_length_1387_cov_0.770186	1387	No	2	1	Low-quality	4.28	AAI-based (high-confidence)
NODE_1552_length_1387_cov_0.048137	1387	No	2	2	Low-quality	2.59	AAI-based (high-confidence)
NODE_1553_length_4868_cov_0.247431	4868	No	7	2	Low-quality	8.09	AAI-based (high-confidence)
NODE_15538_length_1363_cov_0.851266	1363	No	2	1	Low-quality	4.14	AAI-based (high-confidence)
NODE_1555_length_4864_cov_0.562644	4864	No	4	3	Low-quality	7.65	AAI-based (high-confidence)
NODE_15569_length_1385_cov_0.150855	1385	No	1	1	Low-quality	3.11	AAI-based (high-confidence)
NODE_1558_length_4861_cov_0.149727	4861	No	5	3	Low-quality	15.03	AAI-based (high-confidence)
NODE_156_length_2755_cov_0.090361	2755	No	6	2	Low-quality	5.16	AAI-based (high-confidence)
NODE_156_length_3170_cov_0.063497	3170	No	4	2	Low-quality	8.07	AAI-based (high-confidence)
NODE_156_length_9451_cov_0.217387	9451	No	8	4	Low-quality	26.24	AAI-based (high-confidence)
NODE_1560_length_1353_cov_0.062201	1353	No	1	1	Low-quality	2.36	AAI-based (high-confidence)
NODE_15606_length_1383_cov_0.169003	1383	No	2	2	Low-quality	2.05	AAI-based (high-confidence)
NODE_1561_length_1059_cov_0.027083	1059	No	2	1	Low-quality	3.12	AAI-based (high-confidence)
NODE_1562_length_4851_cov_0.226221	4851	No	6	3	Low-quality	13.3	AAI-based (high-confidence)
NODE_15630_length_1358_cov_0.084194	1358	No	3	1	Low-quality	2.52	AAI-based (high-confidence)
NODE_1564_length_2363_cov_0.220848	2363	No	5	2	Low-quality	7.71	AAI-based (high-confidence)
NODE_15646_length_1311_cov_0.155941	1311	No	3	2	Low-quality	2.59	AAI-based (high-confidence)
NODE_15654_length_1381_cov_0.105304	1381	No	1	1	Low-quality	2.53	AAI-based (high-confidence)
NODE_15663_length_1356_cov_0.135243	1356	No	5	1	Low-quality	3.06	AAI-based (high-confidence)
NODE_15666_length_1264_cov_0.386266	1264	No	2	2	Low-quality	2.25	AAI-based (high-confidence)
NODE_1567_length_5306_cov_1.308239	5306	No	6	2	Low-quality	14.75	AAI-based (high-confidence)
NODE_15671_length_1310_cov_0.066061	1310	No	3	1	Low-quality	3.02	AAI-based (high-confidence)
NODE_1568_length_3089_cov_0.164214	3089	No	3	2	Low-quality	4.85	AAI-based (high-confidence)
NODE_1568_length_4694_cov_0.170185	4694	No	9	3	Low-quality	12.65	AAI-based (high-confidence)
NODE_1568_length_4846_cov_0.257215	4846	No	7	3	Low-quality	5.98	AAI-based (high-confidence)
NODE_1569_length_5304_cov_0.184630	5304	No	6	6	Low-quality	11.4	AAI-based (high-confidence)
NODE_157_length_13283_cov_0.390701	13283	No	13	7	Low-quality	32.83	AAI-based (high-confidence)
NODE_157_length_14891_cov_0.314359	14891	No	14	4	Low-quality	9.6	AAI-based (high-confidence)
NODE_157_length_2751_cov_0.113499	2751	No	1	1	Low-quality	43.01	AAI-based (high-confidence)
NODE_15711_length_1377_cov_0.111111	1377	No	2	1	Low-quality	3.99	AAI-based (high-confidence)
NODE_15733_length_1262_cov_0.075666	1262	No	3	1	Low-quality	3.73	AAI-based (high-confidence)
NODE_15737_length_1375_cov_0.304075	1375	No	4	2	Low-quality	2.21	AAI-based (high-confidence)
NODE_1574_length_1056_cov_0.056426	1056	No	3	1	Low-quality	3.27	AAI-based (high-confidence)
NODE_15761_length_1199_cov_0.085455	1199	No	1	1	Low-quality	3.98	AAI-based (high-confidence)
NODE_15782_length_1304_cov_0.137759	1304	No	3	2	Low-quality	2.35	AAI-based (high-confidence)

NODE_1579_length_2237_cov_0.100094	2237	No	5	1	Low-quality	4.29	AAI-based (high-confidence)
NODE_158_length_13220_cov_0.579758	13220	No	18	11	Low-quality	41.47	AAI-based (high-confidence)
NODE_158_length_25107_cov_0.383317	25107	No	14	8	Low-quality	38.58	AAI-based (high-confidence)
NODE_15821_length_1370_cov_0.249410	1370	No	2	1	Low-quality	4.07	AAI-based (high-confidence)
NODE_15828_length_1347_cov_0.084135	1347	No	3	3	Low-quality	2.18	AAI-based (high-confidence)
NODE_15829_length_1370_cov_0.147915	1370	No	1	1	Low-quality	2.57	AAI-based (high-confidence)
NODE_1583_length_1707_cov_0.087065	1707	No	5	1	Low-quality	5.14	AAI-based (high-confidence)
NODE_1584_length_1332_cov_0.056772	1332	No	2	1	Low-quality	2.48	AAI-based (high-confidence)
NODE_15876_length_1345_cov_0.051364	1345	No	4	2	Low-quality	2.42	AAI-based (high-confidence)
NODE_15878_length_1298_cov_2.123436	1298	No	1	1	Low-quality	3.31	AAI-based (high-confidence)
NODE_1588_length_2345_cov_0.143811	2345	No	3	3	Low-quality	7.21	AAI-based (high-confidence)
NODE_1589_length_1170_cov_0.129785	1170	No	3	1	Low-quality	3.32	AAI-based (high-confidence)
NODE_15898_length_1256_cov_0.044944	1256	No	3	3	Low-quality	2.06	AAI-based (high-confidence)
NODE_159_length_9308_cov_0.203171	9308	No	10	3	Low-quality	4.43	AAI-based (high-confidence)
NODE_15924_length_1365_cov_0.118483	1365	No	4	2	Low-quality	5.54	AAI-based (high-confidence)
NODE_15932_length_1365_cov_0.074250	1365	No	2	1	Low-quality	2.1	AAI-based (high-confidence)
NODE_1594_length_4813_cov_0.222741	4813	No	6	1	Low-quality	8.99	AAI-based (high-confidence)
NODE_15940_length_1364_cov_0.163636	1364	No	1	1	Low-quality	3.82	AAI-based (high-confidence)
NODE_15947_length_1294_cov_0.134728	1294	No	3	1	Low-quality	2.63	AAI-based (high-confidence)
NODE_15949_length_1341_cov_0.055556	1341	No	3	1	Low-quality	2.2	AAI-based (high-confidence)
NODE_1595_length_1168_cov_0.082320	1168	No	2	1	Low-quality	2.68	AAI-based (high-confidence)
NODE_15953_length_1294_cov_0.072803	1294	No	2	1	Low-quality	3.78	AAI-based (high-confidence)
NODE_15981_length_1339_cov_0.082258	1339	No	4	1	Low-quality	3.99	AAI-based (high-confidence)
NODE_16_length_5913_cov_0.120915	5913	No	7	2	Low-quality	19.83	AAI-based (high-confidence)
NODE_1600_length_2334_cov_0.280984	2334	No	6	2	Low-quality	3.91	AAI-based (high-confidence)
NODE_16018_length_1359_cov_0.129365	1359	No	2	1	Low-quality	2.39	AAI-based (high-confidence)
NODE_16031_length_1185_cov_0.074586	1185	No	1	1	Low-quality	2.73	AAI-based (high-confidence)
NODE_16037_length_1336_cov_0.141471	1336	No	3	1	Low-quality	2.53	AAI-based (high-confidence)
NODE_1604_length_4806_cov_0.166136	4806	No	6	1	Low-quality	2.16	AAI-based (high-confidence)
NODE_16048_length_1358_cov_0.120731	1358	No	3	1	Low-quality	3.81	AAI-based (high-confidence)
NODE_16049_length_1336_cov_0.042037	1336	No	3	2	Low-quality	3.11	AAI-based (high-confidence)
NODE_1605_length_4197_cov_0.538067	4197	No	10	3	Low-quality	12.35	AAI-based (high-confidence)
NODE_1606_length_5217_cov_0.153380	5217	No	7	2	Low-quality	2.45	AAI-based (high-confidence)
NODE_16072_length_1356_cov_0.282418	1356	No	4	3	Low-quality	3.61	AAI-based (high-confidence)
NODE_16073_length_1247_cov_0.120209	1247	No	1	1	Low-quality	2.3	AAI-based (high-confidence)
NODE_16074_length_1182_cov_0.090489	1182	No	3	2	Low-quality	3.25	AAI-based (high-confidence)
NODE_1609_length_4803_cov_0.174107	4803	No	9	3	Low-quality	14.98	AAI-based (high-confidence)
NODE_16098_length_1333_cov_0.079417	1333	No	2	1	Low-quality	3.58	AAI-based (high-confidence)
NODE_161_length_14656_cov_0.492890	14656	No	19	6	Low-quality	33.67	AAI-based (high-confidence)
NODE_161_length_6000_cov_0.187595	6000	No	7	3	Low-quality	9.63	AAI-based (high-confidence)
NODE_1611_length_1209_cov_0.090090	1209	No	2	1	Low-quality	3.57	AAI-based (high-confidence)
NODE_16135_length_1245_cov_0.078534	1245	No	2	2	Low-quality	1.99	AAI-based (high-confidence)
NODE_1614_length_2327_cov_0.281418	2327	No	3	2	Low-quality	6.52	AAI-based (high-confidence)
NODE_1615_length_5200_cov_0.362870	5200	No	5	2	Low-quality	11.9	AAI-based (high-confidence)
NODE_16168_length_1351_cov_0.485623	1351	No	4	1	Low-quality	3.21	AAI-based (high-confidence)
NODE_1617_length_5199_cov_0.124314	5199	No	4	1	Low-quality	16.34	AAI-based (high-confidence)

NODE_16177_length_1351_cov_0.115016	1351	No	2	2	Low-quality	2.2	AAI-based (high-confidence)
NODE_1618_length_1323_cov_0.070261	1323	No	2	1	Low-quality	2.55	AAI-based (high-confidence)
NODE_1619_length_1362_cov_0.060174	1362	No	2	1	Low-quality	3.76	AAI-based (high-confidence)
NODE_162_length_3186_cov_0.172012	3186	No	3	2	Low-quality	6.27	AAI-based (high-confidence)
NODE_16207_length_1174_cov_0.076279	1174	No	3	3	Low-quality	2.21	AAI-based (high-confidence)
NODE_1621_length_4789_cov_0.128785	4789	No	4	1	Low-quality	9.01	AAI-based (high-confidence)
NODE_16222_length_1325_cov_0.697390	1325	No	3	1	Low-quality	2.46	AAI-based (high-confidence)
NODE_16233_length_1348_cov_0.070456	1348	No	1	1	Low-quality	3.97	AAI-based (high-confidence)
NODE_16236_length_1274_cov_0.773617	1274	No	3	1	Low-quality	3.81	AAI-based (high-confidence)
NODE_1626_length_3039_cov_0.161565	3039	No	4	3	Low-quality	3.27	AAI-based (high-confidence)
NODE_16265_length_1345_cov_0.300161	1345	No	2	2	Low-quality	3.27	AAI-based (high-confidence)
NODE_1627_length_1158_cov_0.098206	1158	No	4	1	Low-quality	3.11	AAI-based (high-confidence)
NODE_1627_length_4169_cov_0.510811	4169	No	8	3	Low-quality	7.63	AAI-based (high-confidence)
NODE_1627_length_5184_cov_0.509931	5184	No	6	5	Low-quality	2.91	AAI-based (high-confidence)
NODE_16277_length_1170_cov_0.081232	1170	No	3	1	Low-quality	2.33	AAI-based (high-confidence)
NODE_16282_length_1344_cov_0.435341	1344	No	4	1	Low-quality	2.55	AAI-based (high-confidence)
NODE_16290_length_1322_cov_0.074407	1322	No	5	1	Low-quality	2.72	AAI-based (high-confidence)
NODE_16297_length_1321_cov_0.199673	1321	No	3	1	Low-quality	2.63	AAI-based (high-confidence)
NODE_1630_length_1357_cov_0.112878	1357	No	4	1	Low-quality	4.06	AAI-based (high-confidence)
NODE_16308_length_1237_cov_0.089631	1237	No	2	2	Low-quality	2.32	AAI-based (high-confidence)
NODE_16316_length_1270_cov_0.128950	1270	No	2	1	Low-quality	3.16	AAI-based (high-confidence)
NODE_1632_length_4590_cov_0.547762	4590	No	7	3	Low-quality	7.8	AAI-based (high-confidence)
NODE_16354_length_1318_cov_0.493027	1318	No	3	1	Low-quality	2.14	AAI-based (high-confidence)
NODE_16355_length_1341_cov_0.070048	1341	No	2	1	Low-quality	4.17	AAI-based (high-confidence)
NODE_16356_length_1341_cov_0.064412	1341	No	4	2	Low-quality	3.94	AAI-based (high-confidence)
NODE_1636_length_1157_cov_0.065217	1157	No	1	1	Low-quality	2.83	AAI-based (high-confidence)
NODE_1636_length_4150_cov_0.444335	4150	No	5	1	Low-quality	12.67	AAI-based (high-confidence)
NODE_16360_length_1318_cov_0.130435	1318	No	3	1	Low-quality	3.97	AAI-based (high-confidence)
NODE_1637_length_1317_cov_0.100164	1317	No	1	1	Low-quality	20.41	AAI-based (high-confidence)
NODE_16375_length_1164_cov_0.126761	1164	No	2	1	Low-quality	2.83	AAI-based (high-confidence)
NODE_1638_length_1317_cov_0.096880	1317	No	2	1	Low-quality	2.05	AAI-based (high-confidence)
NODE_164_length_3122_cov_0.200132	3122	No	1	1	Low-quality	49.13	AAI-based (high-confidence)
NODE_164_length_9219_cov_0.459868	9219	No	19	5	Low-quality	10.1	AAI-based (high-confidence)
NODE_1641_length_1316_cov_0.129827	1316	No	3	1	Low-quality	2.53	AAI-based (high-confidence)
NODE_16418_length_1337_cov_0.100969	1337	No	2	2	Low-quality	2.74	AAI-based (high-confidence)
NODE_16419_length_1232_cov_0.122683	1232	No	1	1	Low-quality	2.31	AAI-based (high-confidence)
NODE_16419_length_1315_cov_0.051809	1315	No	3	2	Low-quality	2.9	AAI-based (high-confidence)
NODE_1643_length_2191_cov_0.108987	2191	No	3	3	Low-quality	6.95	AAI-based (high-confidence)
NODE_1643_length_3026_cov_0.086095	3026	No	8	2	Low-quality	9.37	AAI-based (high-confidence)
NODE_1643_length_4144_cov_0.625958	4144	No	4	2	Low-quality	7.13	AAI-based (high-confidence)
NODE_16433_length_1336_cov_0.147130	1336	No	3	2	Low-quality	2.5	AAI-based (high-confidence)
NODE_1644_length_4578_cov_0.193793	4578	No	6	4	Low-quality	2.6	AAI-based (high-confidence)
NODE_1644_length_5135_cov_0.366362	5135	No	8	2	Low-quality	15.76	AAI-based (high-confidence)
NODE_1645_length_3021_cov_0.116359	3021	No	4	1	Low-quality	9.34	AAI-based (high-confidence)
NODE_1645_length_6332_cov_0.368202	6332	No	7	2	Low-quality	4.74	AAI-based (high-confidence)
NODE_16450_length_1335_cov_0.440129	1335	No	2	1	Low-quality	2.32	AAI-based (high-confidence)

NODE_16451_length_1335_cov_0.332524	1335	No	3	2	Low-quality	2.4	AAI-based (high-confidence)
NODE_16468_length_1260_cov_0.591731	1260	No	3	1	Low-quality	3.31	AAI-based (high-confidence)
NODE_1647_length_6326_cov_0.522724	6326	No	8	4	Low-quality	10.92	AAI-based (high-confidence)
NODE_16499_length_1333_cov_0.097245	1333	No	4	2	Low-quality	3.1	AAI-based (high-confidence)
NODE_165_length_3122_cov_0.108171	3122	No	5	2	Low-quality	9.03	AAI-based (high-confidence)
NODE_1651_length_3015_cov_0.312414	3015	No	2	1	Low-quality	7.19	AAI-based (high-confidence)
NODE_16518_length_1332_cov_0.102190	1332	No	3	1	Low-quality	2.48	AAI-based (high-confidence)
NODE_16519_length_1332_cov_0.081103	1332	No	3	1	Low-quality	2.83	AAI-based (high-confidence)
NODE_1653_length_4565_cov_0.709807	4565	No	6	2	Low-quality	12.75	AAI-based (high-confidence)
NODE_1654_length_1193_cov_0.069470	1193	No	1	1	Low-quality	21.99	AAI-based (high-confidence)
NODE_16542_length_1307_cov_0.196192	1307	No	4	1	Low-quality	2.12	AAI-based (high-confidence)
NODE_1655_length_3013_cov_0.108442	3013	No	3	3	Low-quality	5.11	AAI-based (high-confidence)
NODE_1656_length_1682_cov_0.027795	1682	No	4	1	Low-quality	3.42	AAI-based (high-confidence)
NODE_16569_length_1330_cov_0.048741	1330	No	2	1	Low-quality	3.73	AAI-based (high-confidence)
NODE_1658_length_2300_cov_0.130850	2300	No	3	2	Low-quality	5.55	AAI-based (high-confidence)
NODE_1658_length_5102_cov_0.174695	5102	No	8	5	Low-quality	4.93	AAI-based (high-confidence)
NODE_16583_length_1306_cov_0.101077	1306	No	5	2	Low-quality	3.14	AAI-based (high-confidence)
NODE_16585_length_1225_cov_0.280639	1225	No	1	1	Low-quality	2.67	AAI-based (high-confidence)
NODE_16589_length_1225_cov_0.149201	1225	No	2	1	Low-quality	2.77	AAI-based (high-confidence)
NODE_16590_length_1306_cov_0.052196	1306	No	3	2	Low-quality	4.08	AAI-based (high-confidence)
NODE_166_length_14357_cov_0.442629	14357	No	25	2	Low-quality	22.53	AAI-based (high-confidence)
NODE_1660_length_4112_cov_0.318714	4112	No	3	2	Low-quality	6.44	AAI-based (high-confidence)
NODE_16607_length_1304_cov_0.248133	1304	No	2	1	Low-quality	4.22	AAI-based (high-confidence)
NODE_1661_length_2299_cov_0.123636	2299	No	2	1	Low-quality	7.07	AAI-based (high-confidence)
NODE_1661_length_4727_cov_0.191011	4727	No	14	2	Low-quality	9.89	AAI-based (high-confidence)
NODE_16635_length_1326_cov_0.249389	1326	No	2	1	Low-quality	3.75	AAI-based (high-confidence)
NODE_16641_length_1303_cov_0.054817	1303	No	3	1	Low-quality	2.27	AAI-based (high-confidence)
NODE_16653_length_1326_cov_0.045640	1326	No	1	1	Low-quality	2.52	AAI-based (high-confidence)
NODE_1666_length_4726_cov_0.149125	4726	No	4	1	Low-quality	5.35	AAI-based (high-confidence)
NODE_167_length_2945_cov_0.121574	2945	No	5	4	Low-quality	4.77	AAI-based (high-confidence)
NODE_16702_length_1299_cov_0.212500	1299	No	2	2	Low-quality	2.95	AAI-based (high-confidence)
NODE_16725_length_1322_cov_0.112020	1322	No	1	1	Low-quality	2.27	AAI-based (high-confidence)
NODE_16733_length_1298_cov_0.048374	1298	No	2	2	Low-quality	3.64	AAI-based (high-confidence)
NODE_1674_length_4096_cov_0.387290	4096	No	5	3	Low-quality	12.19	AAI-based (high-confidence)
NODE_16742_length_1297_cov_0.177796	1297	No	2	1	Low-quality	4.07	AAI-based (high-confidence)
NODE_16746_length_1218_cov_0.032172	1218	No	2	1	Low-quality	2.75	AAI-based (high-confidence)
NODE_16748_length_1217_cov_0.180680	1217	No	3	2	Low-quality	2.24	AAI-based (high-confidence)
NODE_16756_length_1296_cov_0.275689	1296	No	5	3	Low-quality	2.55	AAI-based (high-confidence)
NODE_1676_length_2990_cov_0.659979	2990	No	5	2	Low-quality	9.03	AAI-based (high-confidence)
NODE_16790_length_1216_cov_0.059982	1216	No	3	1	Low-quality	1.94	AAI-based (high-confidence)
NODE_16817_length_1240_cov_0.102542	1240	No	3	1	Low-quality	2.74	AAI-based (high-confidence)
NODE_1682_length_4086_cov_0.135189	4086	No	5	1	Low-quality	6.51	AAI-based (high-confidence)
NODE_1683_length_4706_cov_0.206642	4706	No	7	2	Low-quality	2.45	AAI-based (high-confidence)
NODE_16839_length_1316_cov_0.131471	1316	No	3	1	Low-quality	2.59	AAI-based (high-confidence)
NODE_16850_length_1292_cov_0.058676	1292	No	2	1	Low-quality	3.69	AAI-based (high-confidence)
NODE_16852_length_1316_cov_0.065735	1316	No	3	1	Low-quality	3.95	AAI-based (high-confidence)

NODE_1686_length_1313_cov_0.057661	1313	No	2	2	Low-quality	3.8	AAI-based (high-confidence)
NODE_1686_length_2982_cov_0.121401	2982	No	5	1	Low-quality	6.72	AAI-based (high-confidence)
NODE_1688_length_4702_cov_0.165327	4702	No	11	1	Low-quality	2.22	AAI-based (high-confidence)
NODE_16880_length_1314_cov_0.387654	1314	No	3	3	Low-quality	2.42	AAI-based (high-confidence)
NODE_169_length_14294_cov_0.608806	14294	No	15	7	Low-quality	39.03	AAI-based (high-confidence)
NODE_169_length_3100_cov_0.117961	3100	No	7	4	Low-quality	5.31	AAI-based (high-confidence)
NODE_169_length_5873_cov_0.427260	5873	No	10	1	Low-quality	16.4	AAI-based (high-confidence)
NODE_1691_length_2280_cov_0.438790	2280	No	7	1	Low-quality	7.08	AAI-based (high-confidence)
NODE_1692_length_4074_cov_0.177610	4074	No	10	1	Low-quality	2.78	AAI-based (high-confidence)
NODE_16923_length_1313_cov_0.070016	1313	No	3	1	Low-quality	2.29	AAI-based (high-confidence)
NODE_1694_length_6232_cov_0.135496	6232	No	2	1	Low-quality	9.43	AAI-based (high-confidence)
NODE_16951_length_1233_cov_0.082011	1233	No	2	1	Low-quality	3.02	AAI-based (high-confidence)
NODE_16953_length_1233_cov_0.065256	1233	No	1	1	Low-quality	3.76	AAI-based (high-confidence)
NODE_16958_length_1208_cov_0.476105	1208	No	3	2	Low-quality	2.11	AAI-based (high-confidence)
NODE_16961_length_1232_cov_0.453663	1232	No	2	1	Low-quality	2.17	AAI-based (high-confidence)
NODE_16989_length_1135_cov_0.139961	1135	No	2	1	Low-quality	3.36	AAI-based (high-confidence)
NODE_1699_length_1179_cov_0.075000	1179	No	2	1	Low-quality	21.43	AAI-based (high-confidence)
NODE_16991_length_1309_cov_0.134711	1309	No	2	2	Low-quality	3.42	AAI-based (high-confidence)
NODE_16992_length_1135_cov_0.130309	1135	No	1	1	Low-quality	2.12	AAI-based (high-confidence)
NODE_16994_length_1207_cov_0.107401	1207	No	2	2	Low-quality	2.05	AAI-based (high-confidence)
NODE_17_length_14211_cov_0.265590	14211	No	25	7	Low-quality	24.16	AAI-based (high-confidence)
NODE_17_length_4831_cov_0.164624	4831	No	10	2	Low-quality	15.39	AAI-based (high-confidence)
NODE_17_length_5013_cov_0.093203	5013	No	8	4	Low-quality	7.98	AAI-based (high-confidence)
NODE_1700_length_5030_cov_0.159197	5030	No	6	6	Low-quality	2.83	AAI-based (high-confidence)
NODE_17011_length_1283_cov_0.648649	1283	No	2	2	Low-quality	2.27	AAI-based (high-confidence)
NODE_17029_length_1283_cov_0.085304	1283	No	4	2	Low-quality	3.44	AAI-based (high-confidence)
NODE_1703_length_4062_cov_0.098663	4062	No	5	3	Low-quality	11.05	AAI-based (high-confidence)
NODE_1704_length_1119_cov_0.059804	1119	No	1	1	Low-quality	2.37	AAI-based (high-confidence)
NODE_1706_length_2155_cov_0.162451	2155	No	6	2	Low-quality	6.63	AAI-based (high-confidence)
NODE_17060_length_1227_cov_0.116135	1227	No	3	1	Low-quality	2.57	AAI-based (high-confidence)
NODE_1708_length_1118_cov_0.096173	1118	No	4	2	Low-quality	2.43	AAI-based (high-confidence)
NODE_17083_length_1304_cov_0.080498	1304	No	3	1	Low-quality	2.55	AAI-based (high-confidence)
NODE_171_length_2646_cov_0.188850	2646	No	3	1	Low-quality	7.37	AAI-based (high-confidence)
NODE_17107_length_1278_cov_0.552163	1278	No	2	1	Low-quality	2.11	AAI-based (high-confidence)
NODE_1712_length_4488_cov_0.246981	4488	No	6	3	Low-quality	12.55	AAI-based (high-confidence)
NODE_17125_length_1277_cov_0.719864	1277	No	3	1	Low-quality	3.81	AAI-based (high-confidence)
NODE_17125_length_1302_cov_0.078138	1302	No	2	1	Low-quality	2.42	AAI-based (high-confidence)
NODE_1714_length_4044_cov_0.597719	4044	No	4	3	Low-quality	11.29	AAI-based (high-confidence)
NODE_17156_length_1300_cov_0.157369	1300	No	1	1	Low-quality	3.93	AAI-based (high-confidence)
NODE_17161_length_1276_cov_0.050127	1276	No	3	1	Low-quality	3.94	AAI-based (high-confidence)
NODE_1717_length_1134_cov_0.094686	1134	No	3	1	Low-quality	3.48	AAI-based (high-confidence)
NODE_17176_length_1300_cov_0.060783	1300	No	3	1	Low-quality	2.48	AAI-based (high-confidence)
NODE_17196_length_1199_cov_0.076364	1199	No	3	2	Low-quality	2.47	AAI-based (high-confidence)
NODE_17200_length_1199_cov_0.043636	1199	No	1	1	Low-quality	2.23	AAI-based (high-confidence)
NODE_1721_length_2264_cov_0.124249	2264	No	2	2	Low-quality	4.26	AAI-based (high-confidence)
NODE_17213_length_1298_cov_0.080067	1298	No	2	2	Low-quality	3.92	AAI-based (high-confidence)

NODE_1723_length_1289_cov_0.060504	1289	No	3	1	Low-quality	3.46	AAI-based (high-confidence)
NODE_1723_length_4652_cov_0.181858	4652	No	6	2	Low-quality	12.84	AAI-based (high-confidence)
NODE_17242_length_1271_cov_0.090444	1271	No	1	1	Low-quality	2.69	AAI-based (high-confidence)
NODE_1726_length_4648_cov_0.214992	4648	No	5	4	Low-quality	2.65	AAI-based (high-confidence)
NODE_17281_length_1195_cov_0.141423	1195	No	2	1	Low-quality	3.57	AAI-based (high-confidence)
NODE_17285_length_1268_cov_0.893926	1268	No	3	2	Low-quality	3.24	AAI-based (high-confidence)
NODE_1729_length_4471_cov_0.201281	4471	No	8	3	Low-quality	13.98	AAI-based (high-confidence)
NODE_173_length_23328_cov_0.202462	23328	No	36	6	Low-quality	28.97	AAI-based (high-confidence)
NODE_17300_length_1268_cov_0.088109	1268	No	4	1	Low-quality	2.07	AAI-based (high-confidence)
NODE_1732_length_1324_cov_0.179592	1324	No	2	1	Low-quality	2.13	AAI-based (high-confidence)
NODE_17326_length_1293_cov_0.112228	1293	No	1	1	Low-quality	3.94	AAI-based (high-confidence)
NODE_1733_length_1658_cov_0.091725	1658	No	3	1	Low-quality	3.82	AAI-based (high-confidence)
NODE_17331_length_1266_cov_0.271637	1266	No	3	3	Low-quality	3.44	AAI-based (high-confidence)
NODE_17353_length_1193_cov_0.019196	1193	No	4	1	Low-quality	1.99	AAI-based (high-confidence)
NODE_17355_length_1192_cov_0.209515	1192	No	2	2	Low-quality	2.12	AAI-based (high-confidence)
NODE_1737_length_4980_cov_0.228642	4980	No	7	2	Low-quality	3.05	AAI-based (high-confidence)
NODE_17375_length_1191_cov_0.252747	1191	No	3	2	Low-quality	3.59	AAI-based (high-confidence)
NODE_174_length_2601_cov_0.081535	2601	No	5	1	Low-quality	7.98	AAI-based (high-confidence)
NODE_1740_length_4971_cov_0.651273	4971	No	4	2	Low-quality	15.25	AAI-based (high-confidence)
NODE_17421_length_1262_cov_0.156492	1262	No	3	1	Low-quality	3.93	AAI-based (high-confidence)
NODE_1743_length_1294_cov_0.125523	1294	No	3	1	Low-quality	2.45	AAI-based (high-confidence)
NODE_17437_length_1189_cov_0.147706	1189	No	2	2	Low-quality	3.6	AAI-based (high-confidence)
NODE_17439_length_1262_cov_0.022356	1262	No	4	3	Low-quality	3.52	AAI-based (high-confidence)
NODE_17440_length_1261_cov_0.407057	1261	No	4	1	Low-quality	2.83	AAI-based (high-confidence)
NODE_17460_length_1188_cov_0.179063	1188	No	1	1	Low-quality	2.05	AAI-based (high-confidence)
NODE_17474_length_1286_cov_0.150800	1286	No	4	1	Low-quality	2.53	AAI-based (high-confidence)
NODE_175_length_3041_cov_0.135282	3041	No	4	1	Low-quality	4.94	AAI-based (high-confidence)
NODE_175_length_5724_cov_0.211733	5724	No	8	3	Low-quality	16.56	AAI-based (high-confidence)
NODE_17502_length_1112_cov_0.185587	1112	No	4	1	Low-quality	3.63	AAI-based (high-confidence)
NODE_17516_length_1284_cov_0.266667	1284	No	2	2	Low-quality	2.11	AAI-based (high-confidence)
NODE_17517_length_1284_cov_0.260759	1284	No	4	2	Low-quality	3.03	AAI-based (high-confidence)
NODE_17537_length_1185_cov_0.127993	1185	No	4	1	Low-quality	3.55	AAI-based (high-confidence)
NODE_17549_length_1256_cov_0.094209	1256	No	1	1	Low-quality	3.39	AAI-based (high-confidence)
NODE_1755_length_1125_cov_0.042885	1125	No	1	1	Low-quality	1.92	AAI-based (high-confidence)
NODE_17567_length_1110_cov_0.067260	1110	No	2	1	Low-quality	2.98	AAI-based (high-confidence)
NODE_1757_length_1123_cov_0.103516	1123	No	4	1	Low-quality	3.68	AAI-based (high-confidence)
NODE_17583_length_1280_cov_0.140559	1280	No	4	1	Low-quality	3.97	AAI-based (high-confidence)
NODE_17591_length_1254_cov_0.160173	1254	No	2	1	Low-quality	2.27	AAI-based (high-confidence)
NODE_17599_length_1108_cov_0.163528	1108	No	4	2	Low-quality	3.47	AAI-based (high-confidence)
NODE_17615_length_1182_cov_0.085873	1182	No	3	1	Low-quality	3.43	AAI-based (high-confidence)
NODE_17625_length_1253_cov_0.067591	1253	No	1	1	Low-quality	4	AAI-based (high-confidence)
NODE_1764_length_3995_cov_0.134240	3995	No	7	2	Low-quality	11.78	AAI-based (high-confidence)
NODE_17646_length_1251_cov_0.161458	1251	No	2	1	Low-quality	2.84	AAI-based (high-confidence)
NODE_17648_length_1277_cov_0.070458	1277	No	3	2	Low-quality	2.42	AAI-based (high-confidence)
NODE_17651_length_1276_cov_0.548853	1276	No	3	2	Low-quality	2.05	AAI-based (high-confidence)
NODE_17667_length_1250_cov_0.482189	1250	No	3	1	Low-quality	3.29	AAI-based (high-confidence)

NODE_17694_length_1178_cov_0.390176	1178	No	2	1	Low-quality	3.61	AAI-based (high-confidence)
NODE_17696_length_1249_cov_0.105217	1249	No	2	1	Low-quality	17.92	AAI-based (high-confidence)
NODE_177_length_14063_cov_0.741979	14063	No	26	4	Low-quality	23.71	AAI-based (high-confidence)
NODE_177_length_2884_cov_0.188151	2884	No	1	1	Low-quality	45.24	AAI-based (high-confidence)
NODE_177_length_5686_cov_0.219438	5686	No	8	2	Low-quality	17.39	AAI-based (high-confidence)
NODE_17711_length_1248_cov_0.147084	1248	No	2	1	Low-quality	3.96	AAI-based (high-confidence)
NODE_17723_length_1177_cov_0.130798	1177	No	3	1	Low-quality	2.32	AAI-based (high-confidence)
NODE_1773_length_2242_cov_0.207653	2242	No	3	1	Low-quality	3.95	AAI-based (high-confidence)
NODE_17755_length_1102_cov_0.155533	1102	No	3	1	Low-quality	2.86	AAI-based (high-confidence)
NODE_17755_length_1271_cov_0.427474	1271	No	4	2	Low-quality	2.11	AAI-based (high-confidence)
NODE_1776_length_2239_cov_0.304673	2239	No	2	1	Low-quality	4.44	AAI-based (high-confidence)
NODE_1777_length_4573_cov_0.224408	4573	No	4	1	Low-quality	8.54	AAI-based (high-confidence)
NODE_17775_length_1245_cov_0.087260	1245	No	3	1	Low-quality	2.87	AAI-based (high-confidence)
NODE_17785_length_1187_cov_0.030331	1187	No	1	1	Low-quality	1.98	AAI-based (high-confidence)
NODE_1779_length_4573_cov_0.128297	4573	No	8	5	Low-quality	2.56	AAI-based (high-confidence)
NODE_1779_length_6032_cov_0.519130	6032	No	7	2	Low-quality	11.9	AAI-based (high-confidence)
NODE_17797_length_1270_cov_0.084543	1270	No	2	1	Low-quality	2.83	AAI-based (high-confidence)
NODE_17798_length_1186_cov_0.084637	1186	No	3	1	Low-quality	2.39	AAI-based (high-confidence)
NODE_1781_length_3969_cov_0.199742	3969	No	6	3	Low-quality	6.74	AAI-based (high-confidence)
NODE_17820_length_1243_cov_0.083042	1243	No	2	1	Low-quality	3.1	AAI-based (high-confidence)
NODE_17832_length_1242_cov_0.153981	1242	No	1	1	Low-quality	2.06	AAI-based (high-confidence)
NODE_1784_length_2884_cov_0.216517	2884	No	5	2	Low-quality	9.14	AAI-based (high-confidence)
NODE_1784_length_4916_cov_0.443845	4916	No	11	1	Low-quality	4.51	AAI-based (high-confidence)
NODE_1785_length_2107_cov_0.180279	2107	No	3	1	Low-quality	3.5	AAI-based (high-confidence)
NODE_1787_length_4909_cov_0.123077	4909	No	5	3	Low-quality	13.99	AAI-based (high-confidence)
NODE_179_length_2512_cov_0.096560	2512	No	1	1	Low-quality	48.15	AAI-based (high-confidence)
NODE_179_length_2605_cov_0.098563	2605	No	6	3	Low-quality	4.51	AAI-based (high-confidence)
NODE_1790_length_2234_cov_0.155504	2234	No	6	2	Low-quality	4.17	AAI-based (high-confidence)
NODE_17901_length_1095_cov_0.102410	1095	No	1	1	Low-quality	2.71	AAI-based (high-confidence)
NODE_17906_length_1239_cov_0.196491	1239	No	2	2	Low-quality	2.13	AAI-based (high-confidence)
NODE_17908_length_1170_cov_0.086835	1170	No	3	1	Low-quality	3.63	AAI-based (high-confidence)
NODE_17917_length_1094_cov_0.116583	1094	No	3	1	Low-quality	3.06	AAI-based (high-confidence)
NODE_17928_length_1263_cov_0.544674	1263	No	4	1	Low-quality	2.23	AAI-based (high-confidence)
NODE_17940_length_1238_cov_0.122915	1238	No	2	1	Low-quality	3.68	AAI-based (high-confidence)
NODE_1795_length_1101_cov_0.059880	1101	No	2	1	Low-quality	2	AAI-based (high-confidence)
NODE_1796_length_1136_cov_0.099325	1136	No	3	2	Low-quality	2.27	AAI-based (high-confidence)
NODE_1797_length_2098_cov_0.116058	2098	No	5	2	Low-quality	4.5	AAI-based (high-confidence)
NODE_1797_length_3958_cov_0.349832	3958	No	11	1	Low-quality	6.21	AAI-based (high-confidence)
NODE_17971_length_1237_cov_0.090510	1237	No	3	2	Low-quality	2.92	AAI-based (high-confidence)
NODE_17974_length_1176_cov_0.710306	1176	No	4	1	Low-quality	2.58	AAI-based (high-confidence)
NODE_17977_length_1092_cov_0.076536	1092	No	3	2	Low-quality	2.69	AAI-based (high-confidence)
NODE_17979_length_1236_cov_0.384345	1236	No	3	2	Low-quality	2.06	AAI-based (high-confidence)
NODE_1798_length_1135_cov_0.084942	1135	No	3	2	Low-quality	1.95	AAI-based (high-confidence)
NODE_17989_length_1091_cov_0.131048	1091	No	1	1	Low-quality	3.27	AAI-based (high-confidence)
NODE_17994_length_1235_cov_0.692782	1235	No	4	1	Low-quality	2.8	AAI-based (high-confidence)
NODE_18_length_5040_cov_0.196114	5040	No	7	5	Low-quality	9.71	AAI-based (high-confidence)

NODE_180_length_14045_cov_0.941130	14045	No	20	11	Low-quality	44.05	AAI-based (high-confidence)
NODE_1800_length_3954_cov_0.225681	3954	No	5	5	Low-quality	6.23	AAI-based (high-confidence)
NODE_18013_length_1260_cov_0.091301	1260	No	3	1	Low-quality	3.74	AAI-based (high-confidence)
NODE_18016_length_1234_cov_1.520705	1234	No	2	1	Low-quality	3.58	AAI-based (high-confidence)
NODE_18035_length_1090_cov_0.069627	1090	No	1	1	Low-quality	2.49	AAI-based (high-confidence)
NODE_18037_length_1234_cov_0.090749	1234	No	2	2	Low-quality	2.84	AAI-based (high-confidence)
NODE_1806_length_4883_cov_0.394858	4883	No	9	5	Low-quality	2.71	AAI-based (high-confidence)
NODE_1807_length_3946_cov_0.188718	3946	No	4	2	Low-quality	6.84	AAI-based (high-confidence)
NODE_1808_length_3945_cov_0.523921	3945	No	8	1	Low-quality	5.96	AAI-based (high-confidence)
NODE_18081_length_1257_cov_0.518135	1257	No	1	1	Low-quality	1.97	AAI-based (high-confidence)
NODE_18084_length_1232_cov_0.058252	1232	No	5	1	Low-quality	3.75	AAI-based (high-confidence)
NODE_181_length_22271_cov_0.701696	22271	No	22	9	Low-quality	36.58	AAI-based (high-confidence)
NODE_1810_length_4880_cov_0.212090	4880	No	5	1	Low-quality	9.53	AAI-based (high-confidence)
NODE_18102_length_1257_cov_0.088946	1257	No	3	3	Low-quality	2.98	AAI-based (high-confidence)
NODE_1811_length_4365_cov_0.122832	4365	No	4	2	Low-quality	14.86	AAI-based (high-confidence)
NODE_1813_length_1110_cov_0.075173	1110	No	2	1	Low-quality	3.63	AAI-based (high-confidence)
NODE_18144_length_1084_cov_0.121827	1084	No	2	2	Low-quality	1.93	AAI-based (high-confidence)
NODE_1816_length_1100_cov_0.105894	1100	No	1	1	Low-quality	16.2	AAI-based (high-confidence)
NODE_18165_length_1083_cov_0.129065	1083	No	1	1	Low-quality	2.03	AAI-based (high-confidence)
NODE_18174_length_1254_cov_0.103030	1254	No	3	2	Low-quality	2.77	AAI-based (high-confidence)
NODE_182_length_1888_cov_0.134712	1888	No	2	1	Low-quality	5.22	AAI-based (high-confidence)
NODE_182_length_5622_cov_0.148651	5622	No	7	2	Low-quality	13.59	AAI-based (high-confidence)
NODE_18202_length_1082_cov_0.045778	1082	No	1	1	Low-quality	2.56	AAI-based (high-confidence)
NODE_18204_length_1227_cov_0.090426	1227	No	2	1	Low-quality	2.26	AAI-based (high-confidence)
NODE_1821_length_1264_cov_0.077253	1264	No	1	1	Low-quality	3.5	AAI-based (high-confidence)
NODE_1822_length_1264_cov_0.055794	1264	No	2	1	Low-quality	3.58	AAI-based (high-confidence)
NODE_18251_length_1251_cov_0.036458	1251	No	2	1	Low-quality	3.75	AAI-based (high-confidence)
NODE_18257_length_1225_cov_0.063055	1225	No	2	2	Low-quality	4.58	AAI-based (high-confidence)
NODE_1826_length_2844_cov_0.131148	2844	No	2	2	Low-quality	4.7	AAI-based (high-confidence)
NODE_18263_length_1079_cov_0.081633	1079	No	3	1	Low-quality	2.14	AAI-based (high-confidence)
NODE_18280_length_1160_cov_1.360038	1160	No	1	1	Low-quality	3.52	AAI-based (high-confidence)
NODE_18283_length_1249_cov_0.116522	1249	No	3	1	Low-quality	3.88	AAI-based (high-confidence)
NODE_18284_length_1249_cov_0.116522	1249	No	3	1	Low-quality	4.23	AAI-based (high-confidence)
NODE_18298_length_1222_cov_0.485307	1222	No	3	1	Low-quality	3.71	AAI-based (high-confidence)
NODE_18299_length_1222_cov_0.315227	1222	No	1	1	Low-quality	2.27	AAI-based (high-confidence)
NODE_183_length_2565_cov_0.074615	2565	No	3	2	Low-quality	4.39	AAI-based (high-confidence)
NODE_1830_length_1261_cov_0.081756	1261	No	2	2	Low-quality	3.22	AAI-based (high-confidence)
NODE_1830_length_2216_cov_0.082664	2216	No	2	1	Low-quality	4.08	AAI-based (high-confidence)
NODE_18314_length_1159_cov_0.080189	1159	No	1	1	Low-quality	2.83	AAI-based (high-confidence)
NODE_18327_length_1222_cov_0.024043	1222	No	2	2	Low-quality	3.82	AAI-based (high-confidence)
NODE_18349_length_1154_cov_0.125118	1154	No	4	1	Low-quality	3.57	AAI-based (high-confidence)
NODE_18353_length_1246_cov_0.436792	1246	No	5	1	Low-quality	1.95	AAI-based (high-confidence)
NODE_18355_length_1246_cov_0.237140	1246	No	3	1	Low-quality	3.98	AAI-based (high-confidence)
NODE_18374_length_1074_cov_0.111795	1074	No	2	1	Low-quality	3.24	AAI-based (high-confidence)
NODE_18375_length_1246_cov_0.088928	1246	No	3	2	Low-quality	3.86	AAI-based (high-confidence)
NODE_1839_length_2835_cov_0.379020	2835	No	3	1	Low-quality	6.63	AAI-based (high-confidence)

NODE_184_length_2524_cov_0.173608	2524	No	1	1	Low-quality	45.12	AAI-based (high-confidence)
NODE_1840_length_2835_cov_0.172149	2835	No	3	1	Low-quality	4.4	AAI-based (high-confidence)
NODE_18418_length_1153_cov_0.094877	1153	No	3	1	Low-quality	2.95	AAI-based (high-confidence)
NODE_1843_length_1124_cov_0.058537	1124	No	2	1	Low-quality	3.41	AAI-based (high-confidence)
NODE_1850_length_2071_cov_0.159229	2071	No	4	2	Low-quality	6.29	AAI-based (high-confidence)
NODE_18509_length_1149_cov_0.080000	1149	No	3	1	Low-quality	2.86	AAI-based (high-confidence)
NODE_1851_length_4822_cov_0.341732	4822	No	4	2	Low-quality	13.47	AAI-based (high-confidence)
NODE_1854_length_1088_cov_0.170880	1088	No	2	1	Low-quality	3.41	AAI-based (high-confidence)
NODE_18543_length_1211_cov_0.322842	1211	No	2	1	Low-quality	3.8	AAI-based (high-confidence)
NODE_1855_length_2200_cov_0.190861	2200	No	2	1	Low-quality	7.16	AAI-based (high-confidence)
NODE_18566_length_1210_cov_0.142214	1210	No	2	2	Low-quality	2.41	AAI-based (high-confidence)
NODE_1857_length_1618_cov_0.231073	1618	No	1	1	Low-quality	25.19	AAI-based (high-confidence)
NODE_1861_length_3879_cov_0.347354	3879	No	4	1	Low-quality	12.3	AAI-based (high-confidence)
NODE_18612_length_1236_cov_0.341249	1236	No	3	1	Low-quality	3.74	AAI-based (high-confidence)
NODE_18614_length_1236_cov_0.202287	1236	No	5	2	Low-quality	2.25	AAI-based (high-confidence)
NODE_1862_length_2197_cov_0.155386	2197	No	5	2	Low-quality	3.76	AAI-based (high-confidence)
NODE_1865_length_2809_cov_0.140959	2809	No	3	3	Low-quality	4.42	AAI-based (high-confidence)
NODE_1868_length_4456_cov_0.403718	4456	No	4	2	Low-quality	7	AAI-based (high-confidence)
NODE_18681_length_1234_cov_0.084581	1234	No	2	2	Low-quality	2.3	AAI-based (high-confidence)
NODE_187_length_2508_cov_0.130760	2508	No	3	1	Low-quality	4.72	AAI-based (high-confidence)
NODE_18703_length_1142_cov_0.191755	1142	No	6	1	Low-quality	3.61	AAI-based (high-confidence)
NODE_18724_length_1060_cov_0.105099	1060	No	1	1	Low-quality	2.33	AAI-based (high-confidence)
NODE_18730_length_1203_cov_0.127717	1203	No	2	1	Low-quality	1.91	AAI-based (high-confidence)
NODE_1874_length_1247_cov_0.056620	1247	No	5	1	Low-quality	3.4	AAI-based (high-confidence)
NODE_18747_length_1202_cov_0.295558	1202	No	3	2	Low-quality	2.24	AAI-based (high-confidence)
NODE_1876_length_4781_cov_1.018795	4781	No	6	3	Low-quality	13.4	AAI-based (high-confidence)
NODE_18763_length_1230_cov_0.119363	1230	No	2	1	Low-quality	2.83	AAI-based (high-confidence)
NODE_18769_length_1140_cov_0.127762	1140	No	3	1	Low-quality	2.96	AAI-based (high-confidence)
NODE_18778_length_1058_cov_0.061522	1058	No	1	1	Low-quality	2.57	AAI-based (high-confidence)
NODE_1879_length_3858_cov_0.128492	3858	No	5	2	Low-quality	6.14	AAI-based (high-confidence)
NODE_18797_length_1200_cov_0.207084	1200	No	3	2	Low-quality	3.67	AAI-based (high-confidence)
NODE_188_length_1875_cov_0.079392	1875	No	3	1	Low-quality	27.43	AAI-based (high-confidence)
NODE_188_length_2230_cov_0.091976	2230	No	4	2	Low-quality	6.95	AAI-based (high-confidence)
NODE_18818_length_1228_cov_0.173605	1228	No	2	2	Low-quality	1.96	AAI-based (high-confidence)
NODE_18819_length_1056_cov_0.115987	1056	No	1	1	Low-quality	2.39	AAI-based (high-confidence)
NODE_1882_length_5823_cov_0.261880	5823	No	12	2	Low-quality	11.71	AAI-based (high-confidence)
NODE_18829_length_1139_cov_0.046154	1139	No	1	1	Low-quality	2.2	AAI-based (high-confidence)
NODE_1884_length_1080_cov_0.083588	1080	No	3	1	Low-quality	3.37	AAI-based (high-confidence)
NODE_1884_length_1255_cov_0.082180	1255	No	2	1	Low-quality	3.92	AAI-based (high-confidence)
NODE_1884_length_5822_cov_0.096453	5822	No	7	4	Low-quality	13.59	AAI-based (high-confidence)
NODE_18843_length_1055_cov_0.108787	1055	No	3	2	Low-quality	2.06	AAI-based (high-confidence)
NODE_1885_length_2786_cov_0.161891	2786	No	5	3	Low-quality	7.32	AAI-based (high-confidence)
NODE_18866_length_1227_cov_0.049645	1227	No	3	1	Low-quality	2.21	AAI-based (high-confidence)
NODE_18867_length_1227_cov_0.046986	1227	No	1	1	Low-quality	3.27	AAI-based (high-confidence)
NODE_1887_length_1254_cov_0.042424	1254	No	1	1	Low-quality	3.21	AAI-based (high-confidence)
NODE_1887_length_4774_cov_0.182246	4774	No	13	2	Low-quality	10.15	AAI-based (high-confidence)

NODE_18870_length_1226_cov_0.401065	1226	No	4	1	Low-quality	3.8	AAI-based (high-confidence)
NODE_189_length_13388_cov_0.525773	13388	No	16	3	Low-quality	6.91	AAI-based (high-confidence)
NODE_189_length_21634_cov_0.226979	21634	No	21	8	Low-quality	34.32	AAI-based (high-confidence)
NODE_18915_length_1135_cov_0.179537	1135	No	2	1	Low-quality	3.48	AAI-based (high-confidence)
NODE_18916_length_1053_cov_0.074423	1053	No	3	2	Low-quality	2.56	AAI-based (high-confidence)
NODE_1893_length_4424_cov_0.131792	4424	No	7	5	Low-quality	2.43	AAI-based (high-confidence)
NODE_18931_length_1128_cov_0.064140	1128	No	1	1	Low-quality	3.46	AAI-based (high-confidence)
NODE_18958_length_1134_cov_0.101449	1134	No	1	1	Low-quality	2.07	AAI-based (high-confidence)
NODE_18976_length_1050_cov_0.191377	1050	No	2	1	Low-quality	2.27	AAI-based (high-confidence)
NODE_1898_length_2780_cov_0.280492	2780	No	9	1	Low-quality	8.93	AAI-based (high-confidence)
NODE_19_length_4799_cov_0.124894	4799	No	11	2	Low-quality	13.9	AAI-based (high-confidence)
NODE_19_length_5006_cov_0.126758	5006	No	12	2	Low-quality	11.82	AAI-based (high-confidence)
NODE_19011_length_1220_cov_0.107939	1220	No	3	1	Low-quality	3.72	AAI-based (high-confidence)
NODE_19012_length_1192_cov_0.112534	1192	No	3	1	Low-quality	2.49	AAI-based (high-confidence)
NODE_19028_length_1131_cov_0.133721	1131	No	3	1	Low-quality	3.52	AAI-based (high-confidence)
NODE_1903_length_2172_cov_0.474192	2172	No	3	1	Low-quality	6.27	AAI-based (high-confidence)
NODE_1903_length_4261_cov_0.283758	4261	No	7	3	Low-quality	13.1	AAI-based (high-confidence)
NODE_19037_length_1219_cov_0.090179	1219	No	1	1	Low-quality	2.19	AAI-based (high-confidence)
NODE_1904_length_2172_cov_0.150989	2172	No	6	1	Low-quality	3.32	AAI-based (high-confidence)
NODE_1905_length_1603_cov_0.043883	1603	No	2	1	Low-quality	4.36	AAI-based (high-confidence)
NODE_19066_length_1130_cov_0.103783	1130	No	3	1	Low-quality	3.56	AAI-based (high-confidence)
NODE_19078_length_1190_cov_0.076994	1190	No	3	1	Low-quality	2.48	AAI-based (high-confidence)
NODE_19078_length_1217_cov_0.125224	1217	No	3	2	Low-quality	2.21	AAI-based (high-confidence)
NODE_1909_length_4744_cov_0.472982	4744	No	8	3	Low-quality	12.3	AAI-based (high-confidence)
NODE_19093_length_1129_cov_0.120388	1129	No	2	2	Low-quality	3.46	AAI-based (high-confidence)
NODE_191_length_11951_cov_0.164529	11951	No	17	8	Low-quality	31.08	AAI-based (high-confidence)
NODE_191_length_13310_cov_0.209371	13310	No	24	7	Low-quality	22.96	AAI-based (high-confidence)
NODE_1910_length_1123_cov_0.070312	1123	No	1	1	Low-quality	3.28	AAI-based (high-confidence)
NODE_19119_length_1188_cov_0.066116	1188	No	2	2	Low-quality	1.94	AAI-based (high-confidence)
NODE_1915_length_3827_cov_0.704667	3827	No	6	4	Low-quality	6.51	AAI-based (high-confidence)
NODE_19160_length_1214_cov_0.084305	1214	No	4	1	Low-quality	2.48	AAI-based (high-confidence)
NODE_19163_length_1186_cov_0.067157	1186	No	2	1	Low-quality	2.19	AAI-based (high-confidence)
NODE_1917_length_1087_cov_0.085020	1087	No	4	1	Low-quality	2.03	AAI-based (high-confidence)
NODE_19172_length_1185_cov_0.124309	1185	No	1	1	Low-quality	2.8	AAI-based (high-confidence)
NODE_19176_length_1044_cov_0.088889	1044	No	2	1	Low-quality	2.16	AAI-based (high-confidence)
NODE_1918_length_2766_cov_0.239970	2766	No	4	2	Low-quality	11.36	AAI-based (high-confidence)
NODE_19189_length_1184_cov_0.159447	1184	No	2	1	Low-quality	2.19	AAI-based (high-confidence)
NODE_19206_length_1212_cov_0.144654	1212	No	2	1	Low-quality	2.74	AAI-based (high-confidence)
NODE_19213_length_1184_cov_0.070046	1184	No	1	1	Low-quality	2.2	AAI-based (high-confidence)
NODE_19214_length_1184_cov_0.069124	1184	No	4	1	Low-quality	2.4	AAI-based (high-confidence)
NODE_1923_length_3820_cov_0.206396	3820	No	5	1	Low-quality	1.92	AAI-based (high-confidence)
NODE_19237_length_1124_cov_0.399024	1124	No	2	1	Low-quality	1.97	AAI-based (high-confidence)
NODE_19239_length_1211_cov_0.052158	1211	No	2	1	Low-quality	2.02	AAI-based (high-confidence)
NODE_1925_length_4245_cov_0.136517	4245	No	12	1	Low-quality	8.53	AAI-based (high-confidence)
NODE_19250_length_1124_cov_0.095610	1124	No	1	1	Low-quality	3.14	AAI-based (high-confidence)
NODE_19251_length_1210_cov_0.112511	1210	No	2	1	Low-quality	2.49	AAI-based (high-confidence)

NODE_1926_length_2031_cov_0.186853	2031	No	5	1	Low-quality	3.17	AAI-based (high-confidence)
NODE_19265_length_1040_cov_0.150903	1040	No	1	1	Low-quality	3.02	AAI-based (high-confidence)
NODE_19272_length_1111_cov_0.089921	1111	No	4	1	Low-quality	2.3	AAI-based (high-confidence)
NODE_19280_length_1181_cov_0.125693	1181	No	2	1	Low-quality	3.63	AAI-based (high-confidence)
NODE_19288_length_1039_cov_0.121277	1039	No	1	1	Low-quality	1.92	AAI-based (high-confidence)
NODE_19290_length_1208_cov_0.341749	1208	No	3	2	Low-quality	2.89	AAI-based (high-confidence)
NODE_19291_length_1122_cov_0.133920	1122	No	2	1	Low-quality	3.44	AAI-based (high-confidence)
NODE_193_length_2471_cov_0.083052	2471	No	1	1	Low-quality	39.06	AAI-based (high-confidence)
NODE_19303_length_1039_cov_0.040426	1039	No	1	1	Low-quality	2.43	AAI-based (high-confidence)
NODE_19304_length_1039_cov_0.034043	1039	No	2	1	Low-quality	2	AAI-based (high-confidence)
NODE_1935_length_2749_cov_0.111698	2749	No	2	2	Low-quality	6.39	AAI-based (high-confidence)
NODE_19351_length_1120_cov_0.498531	1120	No	2	1	Low-quality	2.01	AAI-based (high-confidence)
NODE_19357_length_1206_cov_0.085818	1206	No	2	1	Low-quality	2.26	AAI-based (high-confidence)
NODE_19361_length_1206_cov_0.041554	1206	No	3	1	Low-quality	2.75	AAI-based (high-confidence)
NODE_19393_length_1119_cov_0.078431	1119	No	2	1	Low-quality	3.04	AAI-based (high-confidence)
NODE_19395_length_1204_cov_0.085068	1204	No	3	1	Low-quality	3.42	AAI-based (high-confidence)
NODE_19399_length_1204_cov_0.067873	1204	No	1	1	Low-quality	2.16	AAI-based (high-confidence)
NODE_19403_length_1204_cov_0.041629	1204	No	5	3	Low-quality	2.66	AAI-based (high-confidence)
NODE_19411_length_1176_cov_0.084494	1176	No	1	1	Low-quality	2.08	AAI-based (high-confidence)
NODE_1942_length_4366_cov_0.186548	4366	No	8	5	Low-quality	8.28	AAI-based (high-confidence)
NODE_19439_length_1117_cov_0.106090	1117	No	1	1	Low-quality	2.26	AAI-based (high-confidence)
NODE_19446_length_1117_cov_0.096267	1117	No	2	2	Low-quality	3.45	AAI-based (high-confidence)
NODE_19473_length_1201_cov_0.108893	1201	No	2	1	Low-quality	2.2	AAI-based (high-confidence)
NODE_19475_length_1174_cov_0.063256	1174	No	4	1	Low-quality	3.55	AAI-based (high-confidence)
NODE_1949_length_2148_cov_0.079063	2148	No	4	2	Low-quality	3.45	AAI-based (high-confidence)
NODE_1949_length_4701_cov_0.193177	4701	No	8	8	Low-quality	12.54	AAI-based (high-confidence)
NODE_1951_length_4354_cov_0.436428	4354	No	4	4	Low-quality	2.44	AAI-based (high-confidence)
NODE_19520_length_1199_cov_0.100000	1199	No	3	2	Low-quality	2.17	AAI-based (high-confidence)
NODE_1953_length_4697_cov_0.375381	4697	No	6	3	Low-quality	14.57	AAI-based (high-confidence)
NODE_1954_length_1229_cov_0.130973	1229	No	4	1	Low-quality	3.58	AAI-based (high-confidence)
NODE_1954_length_4696_cov_0.414183	4696	No	5	2	Low-quality	2.62	AAI-based (high-confidence)
NODE_19548_length_1098_cov_0.086086	1098	No	1	1	Low-quality	2.59	AAI-based (high-confidence)
NODE_19566_length_1170_cov_0.081232	1170	No	1	1	Low-quality	3.2	AAI-based (high-confidence)
NODE_19593_length_1112_cov_0.096742	1112	No	2	1	Low-quality	3.03	AAI-based (high-confidence)
NODE_196_length_2186_cov_0.168663	2186	No	2	1	Low-quality	6.86	AAI-based (high-confidence)
NODE_196_length_8519_cov_0.175891	8519	No	7	2	Low-quality	13.36	AAI-based (high-confidence)
NODE_1960_length_1590_cov_0.066398	1590	No	4	2	Low-quality	4.08	AAI-based (high-confidence)
NODE_1962_length_1228_cov_0.074402	1228	No	2	2	Low-quality	3.21	AAI-based (high-confidence)
NODE_19626_length_1167_cov_0.533708	1167	No	3	1	Low-quality	3.67	AAI-based (high-confidence)
NODE_19631_length_1167_cov_0.160112	1167	No	3	2	Low-quality	2.39	AAI-based (high-confidence)
NODE_19635_length_1110_cov_0.167161	1110	No	4	1	Low-quality	3.05	AAI-based (high-confidence)
NODE_19636_length_1194_cov_0.105936	1194	No	2	1	Low-quality	3.78	AAI-based (high-confidence)
NODE_1964_length_1234_cov_0.139207	1234	No	3	1	Low-quality	2.38	AAI-based (high-confidence)
NODE_19648_length_1167_cov_0.071161	1167	No	2	2	Low-quality	3.2	AAI-based (high-confidence)
NODE_1965_length_2728_cov_0.218334	2728	No	2	2	Low-quality	8.41	AAI-based (high-confidence)
NODE_19654_length_1193_cov_0.127971	1193	No	2	2	Low-quality	3.54	AAI-based (high-confidence)

NODE_19656_length_1025_cov_0.127430	1025	No	3	1	Low-quality	2.23	AAI-based (high-confidence)
NODE_19662_length_1093_cov_2.789738	1093	No	3	1	Low-quality	2.48	AAI-based (high-confidence)
NODE_1967_length_4204_cov_0.157369	4204	No	11	5	Low-quality	13.07	AAI-based (high-confidence)
NODE_1968_length_4339_cov_0.168868	4339	No	7	4	Low-quality	2.08	AAI-based (high-confidence)
NODE_1969_length_2140_cov_0.147967	2140	No	5	1	Low-quality	3.36	AAI-based (high-confidence)
NODE_19698_length_1108_cov_0.202180	1108	No	3	1	Low-quality	2.16	AAI-based (high-confidence)
NODE_197_length_5422_cov_0.404283	5422	No	14	2	Low-quality	15.92	AAI-based (high-confidence)
NODE_19703_length_1108_cov_0.136769	1108	No	2	1	Low-quality	2.18	AAI-based (high-confidence)
NODE_1971_length_4334_cov_0.253601	4334	No	3	2	Low-quality	1.95	AAI-based (high-confidence)
NODE_19715_length_1108_cov_0.105055	1108	No	2	2	Low-quality	2.04	AAI-based (high-confidence)
NODE_19726_length_1164_cov_0.090141	1164	No	1	1	Low-quality	3.23	AAI-based (high-confidence)
NODE_19731_length_1164_cov_0.048826	1164	No	2	1	Low-quality	1.93	AAI-based (high-confidence)
NODE_19734_length_1163_cov_1.082707	1163	No	4	1	Low-quality	2.55	AAI-based (high-confidence)
NODE_19738_length_1163_cov_0.270677	1163	No	1	1	Low-quality	3.54	AAI-based (high-confidence)
NODE_1975_length_1254_cov_0.126407	1254	No	3	1	Low-quality	2.87	AAI-based (high-confidence)
NODE_19757_length_1107_cov_0.058532	1107	No	2	1	Low-quality	3.42	AAI-based (high-confidence)
NODE_19772_length_1189_cov_0.066972	1189	No	2	1	Low-quality	1.98	AAI-based (high-confidence)
NODE_1978_length_2713_cov_0.135042	2713	No	9	1	Low-quality	8.55	AAI-based (high-confidence)
NODE_19785_length_1162_cov_0.137347	1162	No	2	1	Low-quality	2.13	AAI-based (high-confidence)
NODE_1979_length_4647_cov_0.150396	4647	No	3	3	Low-quality	2.18	AAI-based (high-confidence)
NODE_19792_length_1162_cov_0.093133	1162	No	3	1	Low-quality	3.14	AAI-based (high-confidence)
NODE_198_length_2980_cov_0.098230	2980	No	2	1	Low-quality	7.46	AAI-based (high-confidence)
NODE_1983_length_1105_cov_0.114314	1105	No	2	1	Low-quality	3.42	AAI-based (high-confidence)
NODE_1983_length_1229_cov_0.085841	1229	No	2	1	Low-quality	3.1	AAI-based (high-confidence)
NODE_19868_length_1186_cov_0.023919	1186	No	2	2	Low-quality	1.92	AAI-based (high-confidence)
NODE_1988_length_4313_cov_0.252729	4313	No	9	1	Low-quality	17.13	AAI-based (high-confidence)
NODE_19886_length_1102_cov_0.298106	1102	No	4	1	Low-quality	2.46	AAI-based (high-confidence)
NODE_1989_length_1228_cov_0.082374	1228	No	2	1	Low-quality	2.35	AAI-based (high-confidence)
NODE_1990_length_5610_cov_0.165306	5610	No	9	4	Low-quality	15.93	AAI-based (high-confidence)
NODE_1991_length_2129_cov_0.163054	2129	No	3	1	Low-quality	4.1	AAI-based (high-confidence)
NODE_19917_length_1156_cov_0.213813	1156	No	1	1	Low-quality	2.62	AAI-based (high-confidence)
NODE_1995_length_1997_cov_0.148577	1997	No	5	1	Low-quality	5.78	AAI-based (high-confidence)
NODE_19963_length_1182_cov_0.193906	1182	No	1	1	Low-quality	2.96	AAI-based (high-confidence)
NODE_1997_length_4175_cov_0.094701	4175	No	6	2	Low-quality	4.02	AAI-based (high-confidence)
NODE_1997_length_4305_cov_0.277461	4305	No	6	4	Low-quality	2.41	AAI-based (high-confidence)
NODE_2_length_22834_cov_0.701210	22834	No	32	8	Low-quality	37.5	AAI-based (high-confidence)
NODE_2_length_24398_cov_0.440265	24398	No	21	8	Low-quality	40.17	AAI-based (high-confidence)
NODE_200_length_2895_cov_0.183119	2895	No	1	1	Low-quality	45.64	AAI-based (high-confidence)
NODE_2000_length_2124_cov_0.201975	2124	No	3	2	Low-quality	6.72	AAI-based (high-confidence)
NODE_20001_length_1098_cov_0.105105	1098	No	2	2	Low-quality	3.44	AAI-based (high-confidence)
NODE_2002_length_1070_cov_0.092688	1070	No	2	1	Low-quality	3.3	AAI-based (high-confidence)
NODE_2002_length_1994_cov_0.246438	1994	No	2	2	Low-quality	3.3	AAI-based (high-confidence)
NODE_20042_length_1097_cov_0.073146	1097	No	3	1	Low-quality	2.11	AAI-based (high-confidence)
NODE_2006_length_1991_cov_0.139006	1991	No	3	1	Low-quality	3.53	AAI-based (high-confidence)
NODE_2007_length_4298_cov_0.170517	4298	No	9	1	Low-quality	7.61	AAI-based (high-confidence)
NODE_20080_length_1178_cov_0.070436	1178	No	3	1	Low-quality	2.01	AAI-based (high-confidence)

NODE_20088_length_1177_cov_0.255102	1177	No	2	1	Low-quality	3.53	AAI-based (high-confidence)
NODE_2012_length_1989_cov_0.108995	1989	No	3	1	Low-quality	3.68	AAI-based (high-confidence)
NODE_2012_length_2117_cov_0.117443	2117	No	2	2	Low-quality	3.96	AAI-based (high-confidence)
NODE_2012_length_2694_cov_0.124855	2694	No	3	1	Low-quality	2.42	AAI-based (high-confidence)
NODE_2014_length_1097_cov_0.082164	1097	No	2	2	Low-quality	3.02	AAI-based (high-confidence)
NODE_2014_length_4152_cov_0.188749	4152	No	6	5	Low-quality	2.29	AAI-based (high-confidence)
NODE_2014_length_4605_cov_0.565246	4605	No	6	2	Low-quality	14.33	AAI-based (high-confidence)
NODE_2015_length_1247_cov_0.098432	1247	No	2	1	Low-quality	2.21	AAI-based (high-confidence)
NODE_20157_length_1175_cov_0.096654	1175	No	2	1	Low-quality	2.48	AAI-based (high-confidence)
NODE_2017_length_3736_cov_0.179544	3736	No	5	2	Low-quality	2.19	AAI-based (high-confidence)
NODE_20173_length_1007_cov_0.136564	1007	No	1	1	Low-quality	3.24	AAI-based (high-confidence)
NODE_20178_length_1148_cov_0.050524	1148	No	1	1	Low-quality	2.77	AAI-based (high-confidence)
NODE_202_length_13037_cov_0.257149	13037	No	17	10	Low-quality	28.68	AAI-based (high-confidence)
NODE_202_length_2960_cov_0.096470	2960	No	5	3	Low-quality	7.52	AAI-based (high-confidence)
NODE_20207_length_1147_cov_0.064885	1147	No	3	2	Low-quality	1.91	AAI-based (high-confidence)
NODE_20209_length_1173_cov_0.095903	1173	No	2	2	Low-quality	3.06	AAI-based (high-confidence)
NODE_2021_length_3735_cov_0.166942	3735	No	7	2	Low-quality	2.09	AAI-based (high-confidence)
NODE_2022_length_2114_cov_0.263524	2114	No	5	3	Low-quality	5.16	AAI-based (high-confidence)
NODE_2023_length_1095_cov_0.060241	1095	No	3	2	Low-quality	3.02	AAI-based (high-confidence)
NODE_20230_length_1091_cov_0.082661	1091	No	4	1	Low-quality	2.31	AAI-based (high-confidence)
NODE_20251_length_1171_cov_0.224813	1171	No	1	1	Low-quality	1.93	AAI-based (high-confidence)
NODE_2028_length_3729_cov_0.220937	3729	No	4	1	Low-quality	7.27	AAI-based (high-confidence)
NODE_2029_length_4267_cov_0.145393	4267	No	8	4	Low-quality	2.2	AAI-based (high-confidence)
NODE_20302_length_1088_cov_0.184024	1088	No	4	1	Low-quality	1.95	AAI-based (high-confidence)
NODE_2031_length_1063_cov_0.039419	1063	No	2	1	Low-quality	2.89	AAI-based (high-confidence)
NODE_2032_length_2684_cov_0.234429	2684	No	2	2	Low-quality	4.62	AAI-based (high-confidence)
NODE_2032_length_4586_cov_0.133720	4586	No	6	4	Low-quality	2.58	AAI-based (high-confidence)
NODE_2033_length_1570_cov_0.107410	1570	No	5	2	Low-quality	2.69	AAI-based (high-confidence)
NODE_2033_length_3721_cov_0.154887	3721	No	4	4	Low-quality	8.79	AAI-based (high-confidence)
NODE_2034_length_1211_cov_0.074640	1211	No	3	2	Low-quality	3.39	AAI-based (high-confidence)
NODE_2036_length_4577_cov_0.190710	4577	No	6	3	Low-quality	14	AAI-based (high-confidence)
NODE_20367_length_1087_cov_0.084008	1087	No	3	1	Low-quality	2.79	AAI-based (high-confidence)
NODE_2041_length_1047_cov_0.037975	1047	No	2	1	Low-quality	3.21	AAI-based (high-confidence)
NODE_2041_length_3709_cov_0.226316	3709	No	3	2	Low-quality	2.08	AAI-based (high-confidence)
NODE_2042_length_2105_cov_0.159023	2105	No	5	1	Low-quality	3.4	AAI-based (high-confidence)
NODE_2043_length_3702_cov_0.364974	3702	No	7	4	Low-quality	8.93	AAI-based (high-confidence)
NODE_20432_length_1085_cov_0.077079	1085	No	2	1	Low-quality	3.21	AAI-based (high-confidence)
NODE_20436_length_1165_cov_0.112570	1165	No	5	2	Low-quality	3.13	AAI-based (high-confidence)
NODE_2044_length_4256_cov_0.603560	4256	No	8	2	Low-quality	13.19	AAI-based (high-confidence)
NODE_2046_length_3701_cov_0.176291	3701	No	4	1	Low-quality	6.88	AAI-based (high-confidence)
NODE_2047_length_2103_cov_0.067864	2103	No	2	1	Low-quality	31.7	AAI-based (high-confidence)
NODE_2050_length_3695_cov_0.525028	3695	No	4	3	Low-quality	6.36	AAI-based (high-confidence)
NODE_20525_length_1082_cov_0.070193	1082	No	3	2	Low-quality	2.02	AAI-based (high-confidence)
NODE_2057_length_2097_cov_0.119620	2097	No	3	2	Low-quality	4.83	AAI-based (high-confidence)
NODE_2058_length_2097_cov_0.051552	2097	No	4	3	Low-quality	4.22	AAI-based (high-confidence)
NODE_2058_length_4539_cov_0.354730	4539	No	6	5	Low-quality	11.49	AAI-based (high-confidence)

NODE_20608_length_1159_cov_0.286792	1159	No	3	1	Low-quality	3.09	AAI-based (high-confidence)
NODE_20644_length_1158_cov_0.384325	1158	No	4	1	Low-quality	3.66	AAI-based (high-confidence)
NODE_20664_length_1158_cov_0.101039	1158	No	3	1	Low-quality	4.54	AAI-based (high-confidence)
NODE_2067_length_5491_cov_0.141320	5491	No	4	2	Low-quality	9.07	AAI-based (high-confidence)
NODE_20673_length_1077_cov_0.106339	1077	No	3	2	Low-quality	2.57	AAI-based (high-confidence)
NODE_2068_length_1085_cov_0.167343	1085	No	2	1	Low-quality	2.22	AAI-based (high-confidence)
NODE_2068_length_2092_cov_0.196688	2092	No	3	1	Low-quality	5.22	AAI-based (high-confidence)
NODE_20690_length_1129_cov_0.085437	1129	No	3	2	Low-quality	3.38	AAI-based (high-confidence)
NODE_20698_length_1076_cov_0.134084	1076	No	2	1	Low-quality	3.07	AAI-based (high-confidence)
NODE_207_length_2765_cov_0.135784	2765	No	7	1	Low-quality	8.39	AAI-based (high-confidence)
NODE_207_length_5283_cov_0.481481	5283	No	19	3	Low-quality	14.97	AAI-based (high-confidence)
NODE_2070_length_4522_cov_0.301153	4522	No	11	3	Low-quality	14.42	AAI-based (high-confidence)
NODE_20721_length_1076_cov_0.034800	1076	No	4	3	Low-quality	2.91	AAI-based (high-confidence)
NODE_20723_length_1155_cov_0.174242	1155	No	2	1	Low-quality	2.09	AAI-based (high-confidence)
NODE_20725_length_1128_cov_0.071914	1128	No	2	1	Low-quality	3.51	AAI-based (high-confidence)
NODE_2077_length_1040_cov_0.211477	1040	No	2	1	Low-quality	2.45	AAI-based (high-confidence)
NODE_20772_length_1074_cov_0.135385	1074	No	1	1	Low-quality	2.59	AAI-based (high-confidence)
NODE_2078_length_4508_cov_0.190973	4508	No	6	5	Low-quality	9.23	AAI-based (high-confidence)
NODE_20795_length_1073_cov_0.204312	1073	No	2	1	Low-quality	2.94	AAI-based (high-confidence)
NODE_208_length_12650_cov_0.267708	12650	No	10	7	Low-quality	20.15	AAI-based (high-confidence)
NODE_2080_length_4222_cov_0.503274	4222	No	5	3	Low-quality	2.52	AAI-based (high-confidence)
NODE_20811_length_1152_cov_0.125356	1152	No	1	1	Low-quality	3.22	AAI-based (high-confidence)
NODE_2082_length_2085_cov_0.149043	2085	No	6	2	Low-quality	3.29	AAI-based (high-confidence)
NODE_2084_length_3657_cov_0.238055	3657	No	8	4	Low-quality	5.86	AAI-based (high-confidence)
NODE_2085_length_4221_cov_0.490781	4221	No	8	4	Low-quality	4.06	AAI-based (high-confidence)
NODE_20851_length_1151_cov_0.093156	1151	No	2	1	Low-quality	2.14	AAI-based (high-confidence)
NODE_20855_length_1071_cov_0.208848	1071	No	2	1	Low-quality	2.19	AAI-based (high-confidence)
NODE_20858_length_1151_cov_0.053232	1151	No	4	1	Low-quality	2.45	AAI-based (high-confidence)
NODE_2086_length_4497_cov_0.579809	4497	No	9	4	Low-quality	14.16	AAI-based (high-confidence)
NODE_20862_length_1046_cov_0.145723	1046	No	3	1	Low-quality	2.72	AAI-based (high-confidence)
NODE_2087_length_1204_cov_0.014480	1204	No	3	2	Low-quality	2.37	AAI-based (high-confidence)
NODE_2089_length_1049_cov_0.081053	1049	No	3	1	Low-quality	2.17	AAI-based (high-confidence)
NODE_209_length_8256_cov_0.123697	8256	No	8	7	Low-quality	15.37	AAI-based (high-confidence)
NODE_20919_length_1043_cov_0.713983	1043	No	4	1	Low-quality	3.19	AAI-based (high-confidence)
NODE_2092_length_4209_cov_0.581022	4209	No	2	2	Low-quality	6.6	AAI-based (high-confidence)
NODE_2093_length_4488_cov_0.194350	4488	No	7	4	Low-quality	2.47	AAI-based (high-confidence)
NODE_20952_length_1120_cov_0.101861	1120	No	3	2	Low-quality	2.69	AAI-based (high-confidence)
NODE_20976_length_1147_cov_0.083969	1147	No	2	1	Low-quality	3.18	AAI-based (high-confidence)
NODE_2098_length_2079_cov_0.131818	2079	No	4	2	Low-quality	6.6	AAI-based (high-confidence)
NODE_20989_length_1119_cov_0.088235	1119	No	3	1	Low-quality	3.12	AAI-based (high-confidence)
NODE_21_length_5536_cov_0.216664	5536	No	6	4	Low-quality	10.64	AAI-based (high-confidence)
NODE_210_length_2118_cov_0.128777	2118	No	5	2	Low-quality	4.68	AAI-based (high-confidence)
NODE_21000_length_1118_cov_0.273798	1118	No	2	1	Low-quality	2.73	AAI-based (high-confidence)
NODE_21020_length_1145_cov_0.120459	1145	No	5	1	Low-quality	3.09	AAI-based (high-confidence)
NODE_2103_length_1198_cov_0.083712	1198	No	1	1	Low-quality	17.75	AAI-based (high-confidence)
NODE_2103_length_3641_cov_0.293337	3641	No	7	2	Low-quality	5.71	AAI-based (high-confidence)

NODE_2105_length_1047_cov_0.070675	1047	No	1	1	Low-quality	2.02	AAI-based (high-confidence)
NODE_2105_length_1062_cov_0.061267	1062	No	2	1	Low-quality	2.07	AAI-based (high-confidence)
NODE_2105_length_3640_cov_0.237504	3640	No	4	3	Low-quality	6.67	AAI-based (high-confidence)
NODE_21053_length_1117_cov_0.066798	1117	No	4	2	Low-quality	3.4	AAI-based (high-confidence)
NODE_2106_length_4198_cov_0.122469	4198	No	11	4	Low-quality	13.16	AAI-based (high-confidence)
NODE_2108_length_4478_cov_0.245947	4478	No	12	2	Low-quality	2.1	AAI-based (high-confidence)
NODE_211_length_5208_cov_0.138383	5208	No	7	1	Low-quality	8.66	AAI-based (high-confidence)
NODE_211_length_5231_cov_0.252338	5231	No	7	5	Low-quality	2.88	AAI-based (high-confidence)
NODE_211_length_8219_cov_0.216256	8219	No	9	5	Low-quality	13.08	AAI-based (high-confidence)
NODE_2112_length_2628_cov_0.128114	2628	No	4	1	Low-quality	8.18	AAI-based (high-confidence)
NODE_21126_length_1114_cov_0.088670	1114	No	2	1	Low-quality	3.5	AAI-based (high-confidence)
NODE_2113_length_2628_cov_0.126928	2628	No	3	2	Low-quality	8.17	AAI-based (high-confidence)
NODE_2113_length_4469_cov_0.225629	4469	No	9	3	Low-quality	10.71	AAI-based (high-confidence)
NODE_2115_length_2071_cov_0.109026	2071	No	6	2	Low-quality	3.61	AAI-based (high-confidence)
NODE_2115_length_2627_cov_0.726661	2627	No	2	1	Low-quality	7.72	AAI-based (high-confidence)
NODE_21150_length_1034_cov_2.730481	1034	No	3	2	Low-quality	3.02	AAI-based (high-confidence)
NODE_21150_length_1140_cov_0.111431	1140	No	2	2	Low-quality	1.99	AAI-based (high-confidence)
NODE_2116_length_3635_cov_0.122738	3635	No	9	2	Low-quality	11.58	AAI-based (high-confidence)
NODE_21160_length_1140_cov_0.013449	1140	No	5	2	Low-quality	2.03	AAI-based (high-confidence)
NODE_2117_length_4190_cov_0.519922	4190	No	7	4	Low-quality	3.77	AAI-based (high-confidence)
NODE_2118_length_1552_cov_0.044047	1552	No	3	2	Low-quality	3	AAI-based (high-confidence)
NODE_2121_length_4188_cov_0.127415	4188	No	3	3	Low-quality	1.96	AAI-based (high-confidence)
NODE_21217_length_1111_cov_0.072134	1111	No	1	1	Low-quality	2.91	AAI-based (high-confidence)
NODE_21225_length_1061_cov_0.076923	1061	No	3	2	Low-quality	1.91	AAI-based (high-confidence)
NODE_2126_length_4453_cov_0.643087	4453	No	4	3	Low-quality	6.95	AAI-based (high-confidence)
NODE_2127_length_4453_cov_0.224621	4453	No	8	5	Low-quality	7.63	AAI-based (high-confidence)
NODE_21277_length_1030_cov_0.092374	1030	No	2	1	Low-quality	2.47	AAI-based (high-confidence)
NODE_2128_length_1192_cov_0.061299	1192	No	2	1	Low-quality	3.26	AAI-based (high-confidence)
NODE_213_length_12450_cov_0.207028	12450	No	20	6	Low-quality	21.17	AAI-based (high-confidence)
NODE_21314_length_1058_cov_0.079249	1058	No	3	1	Low-quality	3.35	AAI-based (high-confidence)
NODE_2132_length_3615_cov_0.150739	3615	No	3	3	Low-quality	7.75	AAI-based (high-confidence)
NODE_2133_length_1042_cov_0.080594	1042	No	1	1	Low-quality	2.78	AAI-based (high-confidence)
NODE_214_length_2366_cov_0.086458	2366	No	5	2	Low-quality	4.63	AAI-based (high-confidence)
NODE_214_length_2904_cov_0.055615	2904	No	5	1	Low-quality	8.08	AAI-based (high-confidence)
NODE_214_length_5208_cov_0.331767	5208	No	12	4	Low-quality	8.57	AAI-based (high-confidence)
NODE_214_length_8197_cov_1.422697	8197	No	8	4	Low-quality	14.14	AAI-based (high-confidence)
NODE_21400_length_1026_cov_0.079827	1026	No	1	1	Low-quality	2.01	AAI-based (high-confidence)
NODE_2142_length_4167_cov_0.737463	4167	No	7	3	Low-quality	2.12	AAI-based (high-confidence)
NODE_2143_length_1219_cov_0.081250	1219	No	4	2	Low-quality	2.62	AAI-based (high-confidence)
NODE_21435_length_1130_cov_0.164888	1130	No	4	2	Low-quality	1.91	AAI-based (high-confidence)
NODE_21444_length_1102_cov_0.326022	1102	No	2	1	Low-quality	3.29	AAI-based (high-confidence)
NODE_2147_length_3603_cov_0.170662	3603	No	4	2	Low-quality	9.7	AAI-based (high-confidence)
NODE_21485_length_1053_cov_0.121593	1053	No	1	1	Low-quality	3.7	AAI-based (high-confidence)
NODE_215_length_2106_cov_0.140010	2106	No	9	2	Low-quality	6.64	AAI-based (high-confidence)
NODE_215_length_5199_cov_0.251373	5199	No	4	3	Low-quality	8.15	AAI-based (high-confidence)
NODE_2150_length_1039_cov_0.087234	1039	No	1	1	Low-quality	15.93	AAI-based (high-confidence)

NODE_21530_length_1100_cov_0.052947	1100	No	3	1	Low-quality	2.11	AAI-based (high-confidence)
NODE_2154_length_3599_cov_0.115714	3599	No	8	6	Low-quality	2.01	AAI-based (high-confidence)
NODE_21552_length_1099_cov_0.078000	1099	No	1	1	Low-quality	19.61	AAI-based (high-confidence)
NODE_2156_length_2052_cov_0.178699	2052	No	2	2	Low-quality	4.45	AAI-based (high-confidence)
NODE_2156_length_2608_cov_0.467118	2608	No	6	1	Low-quality	7.12	AAI-based (high-confidence)
NODE_2156_length_3598_cov_0.267219	3598	No	4	2	Low-quality	7.85	AAI-based (high-confidence)
NODE_2156_length_4420_cov_0.118723	4420	No	6	3	Low-quality	3.95	AAI-based (high-confidence)
NODE_21567_length_1125_cov_0.306043	1125	No	5	1	Low-quality	3.55	AAI-based (high-confidence)
NODE_2157_length_2052_cov_0.149514	2052	No	6	3	Low-quality	3.77	AAI-based (high-confidence)
NODE_2158_length_1184_cov_0.070046	1184	No	2	2	Low-quality	3.29	AAI-based (high-confidence)
NODE_21588_length_1097_cov_0.609218	1097	No	1	1	Low-quality	3.22	AAI-based (high-confidence)
NODE_21605_length_1050_cov_0.084122	1050	No	2	2	Low-quality	1.98	AAI-based (high-confidence)
NODE_2163_length_1051_cov_0.042017	1051	No	2	1	Low-quality	3.13	AAI-based (high-confidence)
NODE_2163_length_1183_cov_0.084871	1183	No	3	1	Low-quality	3.3	AAI-based (high-confidence)
NODE_21641_length_1124_cov_0.028293	1124	No	2	1	Low-quality	2.39	AAI-based (high-confidence)
NODE_21650_length_1048_cov_0.120126	1048	No	2	1	Low-quality	2.03	AAI-based (high-confidence)
NODE_2167_length_3583_cov_0.251435	3583	No	5	5	Low-quality	1.99	AAI-based (high-confidence)
NODE_2167_length_4005_cov_0.136201	4005	No	5	2	Low-quality	12.7	AAI-based (high-confidence)
NODE_21675_length_1095_cov_0.072289	1095	No	3	1	Low-quality	2.66	AAI-based (high-confidence)
NODE_21676_length_1048_cov_0.050580	1048	No	2	2	Low-quality	2.62	AAI-based (high-confidence)
NODE_21684_length_1047_cov_0.128692	1047	No	2	1	Low-quality	2.14	AAI-based (high-confidence)
NODE_217_length_11189_cov_0.337872	11189	No	11	2	Low-quality	18.48	AAI-based (high-confidence)
NODE_217_length_2704_cov_0.079463	2704	No	6	1	Low-quality	5.93	AAI-based (high-confidence)
NODE_2171_length_4147_cov_0.333498	4147	No	4	2	Low-quality	2.36	AAI-based (high-confidence)
NODE_2172_length_1214_cov_0.068161	1214	No	3	1	Low-quality	2.54	AAI-based (high-confidence)
NODE_21725_length_1120_cov_0.163565	1120	No	3	1	Low-quality	2.02	AAI-based (high-confidence)
NODE_21727_length_1093_cov_0.133803	1093	No	3	1	Low-quality	3.26	AAI-based (high-confidence)
NODE_2173_length_1922_cov_0.365332	1922	No	3	1	Low-quality	5.39	AAI-based (high-confidence)
NODE_2173_length_4386_cov_0.162118	4386	No	10	1	Low-quality	3.22	AAI-based (high-confidence)
NODE_2174_length_2045_cov_0.120761	2045	No	3	2	Low-quality	4.38	AAI-based (high-confidence)
NODE_21751_length_1092_cov_0.202417	1092	No	3	1	Low-quality	2.06	AAI-based (high-confidence)
NODE_21758_length_1045_cov_0.249471	1045	No	2	1	Low-quality	3.29	AAI-based (high-confidence)
NODE_2176_length_1213_cov_0.094255	1213	No	3	1	Low-quality	3.66	AAI-based (high-confidence)
NODE_2178_length_4136_cov_0.145653	4136	No	4	2	Low-quality	2.41	AAI-based (high-confidence)
NODE_21795_length_1044_cov_0.238095	1044	No	1	1	Low-quality	1.96	AAI-based (high-confidence)
NODE_218_length_11179_cov_0.136733	11179	No	25	5	Low-quality	31.04	AAI-based (high-confidence)
NODE_21808_length_1090_cov_0.218971	1090	No	2	2	Low-quality	2.11	AAI-based (high-confidence)
NODE_2181_length_2592_cov_0.395106	2592	No	5	2	Low-quality	6.34	AAI-based (high-confidence)
NODE_21830_length_1117_cov_0.076621	1117	No	2	1	Low-quality	2.08	AAI-based (high-confidence)
NODE_21846_length_1089_cov_0.634343	1089	No	2	1	Low-quality	3.43	AAI-based (high-confidence)
NODE_21847_length_1116_cov_0.129794	1116	No	2	2	Low-quality	2.53	AAI-based (high-confidence)
NODE_2186_length_1063_cov_0.091286	1063	No	4	1	Low-quality	3.48	AAI-based (high-confidence)
NODE_21862_length_1042_cov_0.112407	1042	No	1	1	Low-quality	2.4	AAI-based (high-confidence)
NODE_2188_length_4366_cov_0.273026	4366	No	12	1	Low-quality	12.9	AAI-based (high-confidence)
NODE_21896_length_1041_cov_0.157113	1041	No	5	1	Low-quality	2.01	AAI-based (high-confidence)
NODE_219_length_8130_cov_0.654962	8130	No	10	4	Low-quality	23.25	AAI-based (high-confidence)

NODE_2191_length_1177_cov_0.076994	1177	No	2	1	Low-quality	2.86	AAI-based (high-confidence)
NODE_2193_length_1033_cov_0.078158	1033	No	2	1	Low-quality	3.16	AAI-based (high-confidence)
NODE_2194_length_1028_cov_0.047363	1028	No	3	2	Low-quality	2.86	AAI-based (high-confidence)
NODE_2194_length_1062_cov_0.046729	1062	No	1	1	Low-quality	3.5	AAI-based (high-confidence)
NODE_2194_length_3560_cov_0.167293	3560	No	4	4	Low-quality	4.8	AAI-based (high-confidence)
NODE_21940_length_1087_cov_0.062753	1087	No	2	1	Low-quality	2.56	AAI-based (high-confidence)
NODE_21956_length_1086_cov_0.113475	1086	No	1	1	Low-quality	3.19	AAI-based (high-confidence)
NODE_2198_length_1044_cov_0.055026	1044	No	2	2	Low-quality	2.7	AAI-based (high-confidence)
NODE_2199_length_1027_cov_0.048491	1027	No	1	1	Low-quality	2.8	AAI-based (high-confidence)
NODE_22_length_4842_cov_0.160658	4842	No	7	2	Low-quality	13.48	AAI-based (high-confidence)
NODE_220_length_11160_cov_0.421481	11160	No	14	7	Low-quality	31.34	AAI-based (high-confidence)
NODE_220_length_2428_cov_0.089309	2428	No	6	1	Low-quality	7.15	AAI-based (high-confidence)
NODE_2202_length_2034_cov_0.236693	2034	No	5	3	Low-quality	5.21	AAI-based (high-confidence)
NODE_2204_length_4117_cov_0.096068	4117	No	7	2	Low-quality	16.54	AAI-based (high-confidence)
NODE_22045_length_1109_cov_0.134653	1109	No	1	1	Low-quality	2.67	AAI-based (high-confidence)
NODE_2209_length_5270_cov_0.113131	5270	No	14	3	Low-quality	8.54	AAI-based (high-confidence)
NODE_22156_length_1106_cov_0.089374	1106	No	3	1	Low-quality	2.28	AAI-based (high-confidence)
NODE_2217_length_1173_cov_0.121043	1173	No	4	3	Low-quality	1.92	AAI-based (high-confidence)
NODE_2217_length_2028_cov_0.129601	2028	No	4	2	Low-quality	6.51	AAI-based (high-confidence)
NODE_2218_length_1029_cov_0.067742	1029	No	4	2	Low-quality	3.27	AAI-based (high-confidence)
NODE_2219_length_4328_cov_0.141405	4328	No	5	4	Low-quality	13.89	AAI-based (high-confidence)
NODE_222_length_12228_cov_0.246187	12228	No	20	9	Low-quality	6.8	AAI-based (high-confidence)
NODE_222_length_2869_cov_0.111913	2869	No	4	2	Low-quality	4.66	AAI-based (high-confidence)
NODE_2222_length_4091_cov_1.180862	4091	No	9	2	Low-quality	8.75	AAI-based (high-confidence)
NODE_22230_length_1104_cov_0.084577	1104	No	4	2	Low-quality	2.1	AAI-based (high-confidence)
NODE_22240_length_1077_cov_0.119632	1077	No	2	2	Low-quality	2.14	AAI-based (high-confidence)
NODE_2226_length_5238_cov_1.679510	5238	No	12	3	Low-quality	14.34	AAI-based (high-confidence)
NODE_223_length_2353_cov_0.568767	2353	No	1	1	Low-quality	37.06	AAI-based (high-confidence)
NODE_223_length_2678_cov_0.502520	2678	No	1	1	Low-quality	42.06	AAI-based (high-confidence)
NODE_22301_length_1031_cov_0.066524	1031	No	5	1	Low-quality	3.06	AAI-based (high-confidence)
NODE_22307_length_1031_cov_0.039700	1031	No	1	1	Low-quality	2.77	AAI-based (high-confidence)
NODE_22329_length_1030_cov_0.092374	1030	No	3	1	Low-quality	1.91	AAI-based (high-confidence)
NODE_2233_length_2561_cov_0.076361	2561	No	4	2	Low-quality	7.49	AAI-based (high-confidence)
NODE_2234_length_1057_cov_0.070981	1057	No	1	1	Low-quality	2.61	AAI-based (high-confidence)
NODE_2234_length_4078_cov_0.216386	4078	No	5	4	Low-quality	9.93	AAI-based (high-confidence)
NODE_2235_length_4317_cov_0.278805	4317	No	6	2	Low-quality	7.76	AAI-based (high-confidence)
NODE_22365_length_1074_cov_0.071795	1074	No	2	1	Low-quality	3.4	AAI-based (high-confidence)
NODE_22382_length_1099_cov_0.124000	1099	No	2	1	Low-quality	2.51	AAI-based (high-confidence)
NODE_2239_length_4312_cov_0.120342	4312	No	2	1	Low-quality	2.13	AAI-based (high-confidence)
NODE_224_length_5150_cov_0.410018	5150	No	12	2	Low-quality	15.11	AAI-based (high-confidence)
NODE_224_length_8009_cov_0.149178	8009	No	12	3	Low-quality	16.46	AAI-based (high-confidence)
NODE_2241_length_4071_cov_0.129658	4071	No	4	3	Low-quality	7.42	AAI-based (high-confidence)
NODE_22445_length_1097_cov_0.228457	1097	No	2	2	Low-quality	2.03	AAI-based (high-confidence)
NODE_2247_length_4067_cov_0.117440	4067	No	6	2	Low-quality	5.67	AAI-based (high-confidence)
NODE_22496_length_1025_cov_0.112311	1025	No	1	1	Low-quality	2.77	AAI-based (high-confidence)
NODE_225_length_5148_cov_0.555952	5148	No	9	5	Low-quality	16.59	AAI-based (high-confidence)

NODE_22509_length_1069_cov_0.404124	1069	No	5	1	Low-quality	3.38	AAI-based (high-confidence)
NODE_2251_length_2013_cov_0.101358	2013	No	6	2	Low-quality	6.14	AAI-based (high-confidence)
NODE_22511_length_1069_cov_0.344330	1069	No	6	1	Low-quality	3.38	AAI-based (high-confidence)
NODE_2254_length_3934_cov_0.132464	3934	No	6	1	Low-quality	4.5	AAI-based (high-confidence)
NODE_2254_length_4059_cov_0.608838	4059	No	3	2	Low-quality	7.57	AAI-based (high-confidence)
NODE_2256_length_3509_cov_0.133431	3509	No	8	2	Low-quality	5.43	AAI-based (high-confidence)
NODE_2257_length_4290_cov_0.163923	4290	No	5	3	Low-quality	13.09	AAI-based (high-confidence)
NODE_22581_length_1023_cov_0.097403	1023	No	3	2	Low-quality	3.15	AAI-based (high-confidence)
NODE_22588_length_1093_cov_0.070423	1093	No	1	1	Low-quality	2.11	AAI-based (high-confidence)
NODE_226_length_2859_cov_0.373551	2859	No	1	1	Low-quality	39.22	AAI-based (high-confidence)
NODE_2263_length_1166_cov_0.029991	1166	No	3	1	Low-quality	3.23	AAI-based (high-confidence)
NODE_2263_length_2007_cov_0.175577	2007	No	4	1	Low-quality	2.07	AAI-based (high-confidence)
NODE_2264_length_3503_cov_0.345182	3503	No	6	3	Low-quality	1.95	AAI-based (high-confidence)
NODE_2265_length_3502_cov_0.213047	3502	No	4	2	Low-quality	2.15	AAI-based (high-confidence)
NODE_22692_length_1090_cov_0.068618	1090	No	2	1	Low-quality	2.63	AAI-based (high-confidence)
NODE_22694_length_1064_cov_0.165803	1064	No	1	1	Low-quality	3.26	AAI-based (high-confidence)
NODE_227_length_1805_cov_0.074443	1805	No	3	1	Low-quality	7.38	AAI-based (high-confidence)
NODE_2270_length_2005_cov_0.156348	2005	No	6	1	Low-quality	6.54	AAI-based (high-confidence)
NODE_22704_length_1089_cov_0.600000	1089	No	1	1	Low-quality	2.63	AAI-based (high-confidence)
NODE_2274_length_1010_cov_0.086718	1010	No	2	1	Low-quality	2.82	AAI-based (high-confidence)
NODE_2277_length_4270_cov_0.275713	4270	No	6	3	Low-quality	2.27	AAI-based (high-confidence)
NODE_2278_length_3493_cov_0.331173	3493	No	2	1	Low-quality	5.48	AAI-based (high-confidence)
NODE_22840_length_1060_cov_0.131113	1060	No	3	1	Low-quality	3.33	AAI-based (high-confidence)
NODE_22868_length_1084_cov_0.112690	1084	No	5	1	Low-quality	2.55	AAI-based (high-confidence)
NODE_2288_length_4020_cov_1.637592	4020	No	7	3	Low-quality	2.25	AAI-based (high-confidence)
NODE_2293_length_2526_cov_0.177585	2526	No	2	2	Low-quality	4.22	AAI-based (high-confidence)
NODE_2295_length_1027_cov_0.034483	1027	No	2	1	Low-quality	2.5	AAI-based (high-confidence)
NODE_23_length_4700_cov_0.192132	4700	No	1	1	Low-quality	48.28	AAI-based (high-confidence)
NODE_230_length_2367_cov_0.087302	2367	No	2	1	Low-quality	6.87	AAI-based (high-confidence)
NODE_2300_length_4248_cov_0.516992	4248	No	8	2	Low-quality	13.17	AAI-based (high-confidence)
NODE_2301_length_4247_cov_0.346673	4247	No	4	2	Low-quality	12.87	AAI-based (high-confidence)
NODE_2303_length_1991_cov_0.124736	1991	No	3	1	Low-quality	8.14	AAI-based (high-confidence)
NODE_23033_length_1011_cov_0.114035	1011	No	2	2	Low-quality	2.02	AAI-based (high-confidence)
NODE_2304_length_1877_cov_0.183352	1877	No	2	2	Low-quality	5.29	AAI-based (high-confidence)
NODE_23068_length_1078_cov_0.102145	1078	No	2	1	Low-quality	3.09	AAI-based (high-confidence)
NODE_2307_length_1005_cov_0.167770	1005	No	4	1	Low-quality	2.68	AAI-based (high-confidence)
NODE_2307_length_1153_cov_0.046490	1153	No	3	2	Low-quality	1.94	AAI-based (high-confidence)
NODE_23072_length_1078_cov_0.093973	1078	No	1	1	Low-quality	1.95	AAI-based (high-confidence)
NODE_2308_length_1005_cov_0.143488	1005	No	2	1	Low-quality	1.93	AAI-based (high-confidence)
NODE_2308_length_1187_cov_0.061581	1187	No	5	1	Low-quality	2.38	AAI-based (high-confidence)
NODE_23080_length_1053_cov_0.090147	1053	No	2	1	Low-quality	3.21	AAI-based (high-confidence)
NODE_2309_length_1153_cov_0.043643	1153	No	2	1	Low-quality	3.16	AAI-based (high-confidence)
NODE_2309_length_1186_cov_0.123275	1186	No	2	1	Low-quality	2.3	AAI-based (high-confidence)
NODE_2309_length_4235_cov_0.351064	4235	No	4	2	Low-quality	3.01	AAI-based (high-confidence)
NODE_231_length_17888_cov_0.318174	17888	No	17	5	Low-quality	11.5	AAI-based (high-confidence)
NODE_231_length_1792_cov_0.091553	1792	No	4	1	Low-quality	5.41	AAI-based (high-confidence)

NODE_2311_length_4003_cov_0.142930	4003	No	4	4	Low-quality	7.45	AAI-based (high-confidence)
NODE_2312_length_4230_cov_0.365287	4230	No	6	3	Low-quality	6.86	AAI-based (high-confidence)
NODE_23122_length_1077_cov_0.046012	1077	No	2	1	Low-quality	2.05	AAI-based (high-confidence)
NODE_23126_length_1052_cov_0.040923	1052	No	2	2	Low-quality	2.81	AAI-based (high-confidence)
NODE_2313_length_3882_cov_0.192704	3882	No	5	3	Low-quality	6.24	AAI-based (high-confidence)
NODE_23139_length_1008_cov_0.138614	1008	No	3	1	Low-quality	2	AAI-based (high-confidence)
NODE_23143_length_1051_cov_0.095588	1051	No	2	1	Low-quality	3.21	AAI-based (high-confidence)
NODE_2315_length_3468_cov_0.294153	3468	No	4	4	Low-quality	5.72	AAI-based (high-confidence)
NODE_23156_length_1051_cov_0.052521	1051	No	2	1	Low-quality	2.88	AAI-based (high-confidence)
NODE_2317_length_1004_cov_0.086188	1004	No	3	3	Low-quality	2.25	AAI-based (high-confidence)
NODE_23187_length_1050_cov_0.056782	1050	No	2	1	Low-quality	2.83	AAI-based (high-confidence)
NODE_23194_length_1007_cov_0.071586	1007	No	3	1	Low-quality	3.17	AAI-based (high-confidence)
NODE_232_length_2740_cov_0.087088	2740	No	1	1	Low-quality	43.38	AAI-based (high-confidence)
NODE_2321_length_1150_cov_0.144624	1150	No	1	1	Low-quality	17.64	AAI-based (high-confidence)
NODE_2322_length_4221_cov_0.299369	4221	No	8	3	Low-quality	11.42	AAI-based (high-confidence)
NODE_2325_length_4219_cov_0.183738	4219	No	7	2	Low-quality	2.39	AAI-based (high-confidence)
NODE_2326_length_1021_cov_0.207158	1021	No	1	1	Low-quality	14.65	AAI-based (high-confidence)
NODE_2328_length_1984_cov_0.169761	1984	No	3	1	Low-quality	5.06	AAI-based (high-confidence)
NODE_23283_length_1004_cov_0.213260	1004	No	3	2	Low-quality	2.85	AAI-based (high-confidence)
NODE_233_length_13109_cov_0.221676	13109	No	17	9	Low-quality	35.22	AAI-based (high-confidence)
NODE_233_length_2629_cov_0.120949	2629	No	5	1	Low-quality	4.96	AAI-based (high-confidence)
NODE_2330_length_3981_cov_0.290314	3981	No	4	3	Low-quality	10.6	AAI-based (high-confidence)
NODE_23309_length_1004_cov_0.085083	1004	No	2	1	Low-quality	3.08	AAI-based (high-confidence)
NODE_2331_length_3448_cov_0.448193	3448	No	5	2	Low-quality	6.3	AAI-based (high-confidence)
NODE_2331_length_3981_cov_0.197063	3981	No	4	3	Low-quality	7.21	AAI-based (high-confidence)
NODE_2331_length_4213_cov_0.339815	4213	No	7	3	Low-quality	2.04	AAI-based (high-confidence)
NODE_2332_length_1147_cov_0.045802	1147	No	4	1	Low-quality	3.41	AAI-based (high-confidence)
NODE_23330_length_1045_cov_0.087738	1045	No	1	1	Low-quality	1.93	AAI-based (high-confidence)
NODE_2334_length_3446_cov_0.195698	3446	No	5	2	Low-quality	5.96	AAI-based (high-confidence)
NODE_2336_length_3978_cov_0.060067	3978	No	4	2	Low-quality	8.77	AAI-based (high-confidence)
NODE_23367_length_1044_cov_0.103704	1044	No	2	2	Low-quality	2.41	AAI-based (high-confidence)
NODE_234_length_5052_cov_0.119524	5052	No	6	5	Low-quality	12.35	AAI-based (high-confidence)
NODE_23422_length_1043_cov_0.072034	1043	No	2	2	Low-quality	2.56	AAI-based (high-confidence)
NODE_23472_length_1065_cov_0.138716	1065	No	1	1	Low-quality	2.28	AAI-based (high-confidence)
NODE_2349_length_4195_cov_0.391113	4195	No	4	1	Low-quality	3.74	AAI-based (high-confidence)
NODE_2351_length_5044_cov_1.292619	5044	No	5	1	Low-quality	11.63	AAI-based (high-confidence)
NODE_23555_length_1039_cov_0.105319	1039	No	3	1	Low-quality	3.16	AAI-based (high-confidence)
NODE_23559_length_1039_cov_0.089362	1039	No	1	1	Low-quality	3.28	AAI-based (high-confidence)
NODE_2356_length_3956_cov_0.372051	3956	No	5	3	Low-quality	2.12	AAI-based (high-confidence)
NODE_2357_length_2486_cov_0.733138	2486	No	4	2	Low-quality	7.6	AAI-based (high-confidence)
NODE_23578_length_1062_cov_0.208723	1062	No	1	1	Low-quality	3.06	AAI-based (high-confidence)
NODE_23606_length_1038_cov_0.075612	1038	No	4	1	Low-quality	2.05	AAI-based (high-confidence)
NODE_2363_length_3430_cov_0.526569	3430	No	4	1	Low-quality	6.33	AAI-based (high-confidence)
NODE_2364_length_1140_cov_0.068204	1140	No	1	1	Low-quality	3.35	AAI-based (high-confidence)
NODE_2365_length_3950_cov_0.112698	3950	No	8	5	Low-quality	10.91	AAI-based (high-confidence)
NODE_23650_length_1061_cov_0.044699	1061	No	4	2	Low-quality	2.14	AAI-based (high-confidence)

NODE_23671_length_1036_cov_0.516542	1036	No	2	1	Low-quality	2.41	AAI-based (high-confidence)
NODE_2368_length_3948_cov_0.274357	3948	No	8	5	Low-quality	6.71	AAI-based (high-confidence)
NODE_23694_length_1059_cov_0.114583	1059	No	3	2	Low-quality	2.97	AAI-based (high-confidence)
NODE_23700_length_1036_cov_0.096051	1036	No	2	1	Low-quality	3.15	AAI-based (high-confidence)
NODE_2371_length_3425_cov_0.247745	3425	No	5	1	Low-quality	7.71	AAI-based (high-confidence)
NODE_2371_length_3945_cov_0.182007	3945	No	5	1	Low-quality	4.27	AAI-based (high-confidence)
NODE_23738_length_1035_cov_0.115385	1035	No	2	1	Low-quality	3.27	AAI-based (high-confidence)
NODE_23744_length_1035_cov_0.099359	1035	No	3	1	Low-quality	3.3	AAI-based (high-confidence)
NODE_2376_length_4160_cov_0.212263	4160	No	4	4	Low-quality	6.87	AAI-based (high-confidence)
NODE_2377_length_1034_cov_0.070588	1034	No	2	1	Low-quality	2.85	AAI-based (high-confidence)
NODE_23796_length_1034_cov_0.069519	1034	No	3	2	Low-quality	2.86	AAI-based (high-confidence)
NODE_238_length_2935_cov_0.142102	2935	No	1	1	Low-quality	44.52	AAI-based (high-confidence)
NODE_23801_length_1034_cov_0.025668	1034	No	2	1	Low-quality	1.92	AAI-based (high-confidence)
NODE_23809_length_1056_cov_0.100313	1056	No	3	2	Low-quality	3.24	AAI-based (high-confidence)
NODE_23812_length_1056_cov_0.088819	1056	No	2	2	Low-quality	3.19	AAI-based (high-confidence)
NODE_23876_length_1032_cov_0.064309	1032	No	3	3	Low-quality	2.59	AAI-based (high-confidence)
NODE_239_length_2930_cov_0.088308	2930	No	6	3	Low-quality	8.61	AAI-based (high-confidence)
NODE_23905_length_1031_cov_0.096567	1031	No	2	1	Low-quality	2.86	AAI-based (high-confidence)
NODE_23909_length_1031_cov_0.090129	1031	No	2	1	Low-quality	2.56	AAI-based (high-confidence)
NODE_2392_length_2461_cov_2.609653	2461	No	5	1	Low-quality	4.71	AAI-based (high-confidence)
NODE_2392_length_3818_cov_0.197634	3818	No	9	1	Low-quality	15.18	AAI-based (high-confidence)
NODE_23958_length_1052_cov_0.034627	1052	No	3	2	Low-quality	2.93	AAI-based (high-confidence)
NODE_23959_length_1029_cov_0.375269	1029	No	2	2	Low-quality	1.91	AAI-based (high-confidence)
NODE_2396_length_3401_cov_0.208359	3401	No	9	3	Low-quality	10.71	AAI-based (high-confidence)
NODE_24_length_4338_cov_0.099316	4338	No	11	3	Low-quality	13.72	AAI-based (high-confidence)
NODE_240_length_5000_cov_0.123648	5000	No	14	3	Low-quality	13.98	AAI-based (high-confidence)
NODE_24015_length_1050_cov_0.095689	1050	No	3	2	Low-quality	1.97	AAI-based (high-confidence)
NODE_2404_length_2457_cov_0.156064	2457	No	5	3	Low-quality	2.95	AAI-based (high-confidence)
NODE_2404_length_3920_cov_0.180843	3920	No	7	2	Low-quality	2.2	AAI-based (high-confidence)
NODE_2405_length_1956_cov_0.181475	1956	No	3	1	Low-quality	3.25	AAI-based (high-confidence)
NODE_2405_length_4131_cov_0.113839	4131	No	9	3	Low-quality	1.94	AAI-based (high-confidence)
NODE_2406_length_4127_cov_0.182721	4127	No	3	3	Low-quality	7.46	AAI-based (high-confidence)
NODE_2409_length_3808_cov_0.106498	3808	No	6	1	Low-quality	2.57	AAI-based (high-confidence)
NODE_24091_length_1048_cov_0.076923	1048	No	3	1	Low-quality	2.67	AAI-based (high-confidence)
NODE_241_length_10622_cov_0.290982	10622	No	10	5	Low-quality	32.73	AAI-based (high-confidence)
NODE_241_length_2335_cov_0.117621	2335	No	7	1	Low-quality	6.95	AAI-based (high-confidence)
NODE_242_length_2328_cov_0.147151	2328	No	1	1	Low-quality	35.69	AAI-based (high-confidence)
NODE_242_length_7763_cov_0.153445	7763	No	6	5	Low-quality	12.8	AAI-based (high-confidence)
NODE_2422_length_4113_cov_0.104385	4113	No	5	3	Low-quality	10.37	AAI-based (high-confidence)
NODE_24227_length_1022_cov_0.078007	1022	No	1	1	Low-quality	2.65	AAI-based (high-confidence)
NODE_2424_length_1026_cov_0.094930	1026	No	1	1	Low-quality	15.83	AAI-based (high-confidence)
NODE_2424_length_1829_cov_0.424855	1829	No	3	2	Low-quality	3.02	AAI-based (high-confidence)
NODE_2426_length_3899_cov_0.243684	3899	No	3	2	Low-quality	10.76	AAI-based (high-confidence)
NODE_24265_length_1020_cov_0.651466	1020	No	4	1	Low-quality	2.21	AAI-based (high-confidence)
NODE_2427_length_3794_cov_0.111231	3794	No	9	3	Low-quality	10.8	AAI-based (high-confidence)
NODE_2428_length_1949_cov_0.216216	1949	No	2	1	Low-quality	3	AAI-based (high-confidence)

NODE_24282_length_1020_cov_0.102063	1020	No	1	1	Low-quality	2.75	AAI-based (high-confidence)
NODE_24307_length_1019_cov_0.135870	1019	No	3	2	Low-quality	3.07	AAI-based (high-confidence)
NODE_24338_length_1041_cov_0.040340	1041	No	3	1	Low-quality	3.06	AAI-based (high-confidence)
NODE_2436_length_3893_cov_0.197153	3893	No	8	2	Low-quality	7.16	AAI-based (high-confidence)
NODE_24374_length_1039_cov_0.440426	1039	No	2	1	Low-quality	2.51	AAI-based (high-confidence)
NODE_244_length_10580_cov_0.212671	10580	No	23	6	Low-quality	17.12	AAI-based (high-confidence)
NODE_2440_length_1128_cov_0.171040	1128	No	1	1	Low-quality	18.86	AAI-based (high-confidence)
NODE_24468_length_1037_cov_0.132196	1037	No	2	1	Low-quality	2.82	AAI-based (high-confidence)
NODE_24478_length_1037_cov_0.083156	1037	No	1	1	Low-quality	2.83	AAI-based (high-confidence)
NODE_2449_length_1126_cov_0.068160	1126	No	2	1	Low-quality	3.13	AAI-based (high-confidence)
NODE_245_length_12743_cov_0.198118	12743	No	23	6	Low-quality	35.62	AAI-based (high-confidence)
NODE_2451_length_1163_cov_0.069549	1163	No	3	2	Low-quality	3.08	AAI-based (high-confidence)
NODE_2452_length_3372_cov_0.165903	3372	No	6	2	Low-quality	9.17	AAI-based (high-confidence)
NODE_2455_length_1125_cov_0.107212	1125	No	2	2	Low-quality	3.31	AAI-based (high-confidence)
NODE_2456_length_3370_cov_0.350046	3370	No	5	2	Low-quality	3.02	AAI-based (high-confidence)
NODE_24565_length_1034_cov_0.245989	1034	No	3	1	Low-quality	1.92	AAI-based (high-confidence)
NODE_24566_length_1034_cov_0.232086	1034	No	2	1	Low-quality	2.01	AAI-based (high-confidence)
NODE_24599_length_1011_cov_0.103070	1011	No	6	1	Low-quality	2.1	AAI-based (high-confidence)
NODE_246_length_11799_cov_0.356923	11799	No	14	5	Low-quality	5.94	AAI-based (high-confidence)
NODE_246_length_4934_cov_0.178077	4934	No	6	2	Low-quality	7.73	AAI-based (high-confidence)
NODE_24601_length_1033_cov_0.213062	1033	No	2	1	Low-quality	3.13	AAI-based (high-confidence)
NODE_24608_length_1033_cov_0.119914	1033	No	1	1	Low-quality	2.83	AAI-based (high-confidence)
NODE_2461_length_3367_cov_0.167381	3367	No	5	1	Low-quality	3.48	AAI-based (high-confidence)
NODE_2464_length_1120_cov_0.799216	1120	No	1	1	Low-quality	16.75	AAI-based (high-confidence)
NODE_2464_length_4076_cov_0.170480	4076	No	4	2	Low-quality	2.52	AAI-based (high-confidence)
NODE_24668_length_1009_cov_0.232967	1009	No	1	1	Low-quality	3.06	AAI-based (high-confidence)
NODE_2470_length_3361_cov_0.108522	3361	No	4	1	Low-quality	6.05	AAI-based (high-confidence)
NODE_2475_length_1120_cov_0.050930	1120	No	2	2	Low-quality	2.87	AAI-based (high-confidence)
NODE_24753_length_1007_cov_0.414097	1007	No	3	1	Low-quality	3.23	AAI-based (high-confidence)
NODE_2479_length_4070_cov_0.232687	4070	No	5	4	Low-quality	12.84	AAI-based (high-confidence)
NODE_248_length_2902_cov_0.097752	2902	No	7	1	Low-quality	5.39	AAI-based (high-confidence)
NODE_2481_length_3351_cov_0.181119	3351	No	9	2	Low-quality	5.43	AAI-based (high-confidence)
NODE_2483_length_3350_cov_0.187019	3350	No	7	1	Low-quality	1.94	AAI-based (high-confidence)
NODE_24840_length_1005_cov_0.073951	1005	No	2	1	Low-quality	2.87	AAI-based (high-confidence)
NODE_24875_length_1026_cov_0.071197	1026	No	2	1	Low-quality	2.26	AAI-based (high-confidence)
NODE_2490_length_1926_cov_0.126984	1926	No	3	1	Low-quality	42.37	AAI-based (high-confidence)
NODE_2495_length_3345_cov_0.469501	3345	No	6	2	Low-quality	7.7	AAI-based (high-confidence)
NODE_2499_length_2408_cov_0.196622	2408	No	3	3	Low-quality	3.98	AAI-based (high-confidence)
NODE_25_length_4694_cov_0.099238	4694	No	8	3	Low-quality	9.27	AAI-based (high-confidence)
NODE_25_length_5743_cov_0.112686	5743	No	7	3	Low-quality	17	AAI-based (high-confidence)
NODE_2501_length_3839_cov_0.090374	3839	No	6	3	Low-quality	11.97	AAI-based (high-confidence)
NODE_25073_length_1021_cov_0.057484	1021	No	1	1	Low-quality	3.06	AAI-based (high-confidence)
NODE_2509_length_1801_cov_0.095182	1801	No	3	1	Low-quality	7.06	AAI-based (high-confidence)
NODE_251_length_2559_cov_0.078049	2559	No	5	2	Low-quality	7.84	AAI-based (high-confidence)
NODE_2510_length_4040_cov_0.258818	4040	No	5	1	Low-quality	10.76	AAI-based (high-confidence)
NODE_2511_length_1113_cov_0.097633	1113	No	1	1	Low-quality	20.51	AAI-based (high-confidence)

NODE_2511_length_3836_cov_0.875033	3836	No	4	1	Low-quality	8.76	AAI-based (high-confidence)
NODE_2514_length_1114_cov_0.050246	1114	No	4	1	Low-quality	2.97	AAI-based (high-confidence)
NODE_2515_length_1114_cov_0.045320	1114	No	1	1	Low-quality	2.46	AAI-based (high-confidence)
NODE_2515_length_2399_cov_0.177826	2399	No	4	3	Low-quality	7.36	AAI-based (high-confidence)
NODE_2516_length_4034_cov_0.205083	4034	No	4	2	Low-quality	4.62	AAI-based (high-confidence)
NODE_2519_length_2398_cov_1.111353	2398	No	4	1	Low-quality	7.43	AAI-based (high-confidence)
NODE_252_length_2273_cov_0.132015	2273	No	1	1	Low-quality	32.52	AAI-based (high-confidence)
NODE_2522_length_1917_cov_0.341584	1917	No	3	1	Low-quality	5.37	AAI-based (high-confidence)
NODE_2524_length_3829_cov_0.140483	3829	No	12	3	Low-quality	12.11	AAI-based (high-confidence)
NODE_2525_length_3325_cov_0.351829	3325	No	3	3	Low-quality	7.96	AAI-based (high-confidence)
NODE_253_length_10347_cov_0.414715	10347	No	9	7	Low-quality	23.57	AAI-based (high-confidence)
NODE_2533_length_4021_cov_0.558644	4021	No	5	2	Low-quality	12.62	AAI-based (high-confidence)
NODE_2534_length_4020_cov_0.199439	4020	No	3	2	Low-quality	7.48	AAI-based (high-confidence)
NODE_2535_length_4019_cov_1.116327	4019	No	9	4	Low-quality	3.57	AAI-based (high-confidence)
NODE_2538_length_2391_cov_0.136998	2391	No	1	1	Low-quality	7.08	AAI-based (high-confidence)
NODE_254_length_16890_cov_0.234233	16890	No	23	7	Low-quality	7.94	AAI-based (high-confidence)
NODE_2542_length_1108_cov_0.111001	1108	No	1	1	Low-quality	21.36	AAI-based (high-confidence)
NODE_25449_length_1011_cov_0.169956	1011	No	3	1	Low-quality	1.91	AAI-based (high-confidence)
NODE_2547_length_3810_cov_0.243870	3810	No	5	1	Low-quality	10.4	AAI-based (high-confidence)
NODE_2548_length_2388_cov_0.115771	2388	No	3	1	Low-quality	7.76	AAI-based (high-confidence)
NODE_25485_length_1010_cov_0.305159	1010	No	3	1	Low-quality	2.24	AAI-based (high-confidence)
NODE_255_length_2258_cov_0.137564	2258	No	1	1	Low-quality	47.27	AAI-based (high-confidence)
NODE_2554_length_3997_cov_0.158286	3997	No	8	3	Low-quality	2.28	AAI-based (high-confidence)
NODE_25541_length_1009_cov_0.096703	1009	No	3	1	Low-quality	2.9	AAI-based (high-confidence)
NODE_2556_length_3996_cov_1.584809	3996	No	8	4	Low-quality	9.55	AAI-based (high-confidence)
NODE_2559_length_3993_cov_1.142013	3993	No	9	3	Low-quality	11.39	AAI-based (high-confidence)
NODE_256_length_16826_cov_0.174568	16826	No	11	5	Low-quality	22.7	AAI-based (high-confidence)
NODE_256_length_2258_cov_0.108384	2258	No	4	2	Low-quality	6.98	AAI-based (high-confidence)
NODE_2560_length_2382_cov_0.293035	2382	No	4	2	Low-quality	3.53	AAI-based (high-confidence)
NODE_2562_length_4768_cov_2.579782	4768	No	10	3	Low-quality	13.38	AAI-based (high-confidence)
NODE_2563_length_1901_cov_0.232519	1901	No	5	2	Low-quality	1.93	AAI-based (high-confidence)
NODE_25634_length_1006_cov_0.185226	1006	No	3	1	Low-quality	2.83	AAI-based (high-confidence)
NODE_2568_length_3792_cov_0.109938	3792	No	4	4	Low-quality	8.61	AAI-based (high-confidence)
NODE_257_length_2255_cov_0.060297	2255	No	2	1	Low-quality	6.32	AAI-based (high-confidence)
NODE_257_length_7607_cov_0.226425	7607	No	9	5	Low-quality	24.33	AAI-based (high-confidence)
NODE_2571_length_1101_cov_0.059880	1101	No	1	1	Low-quality	16.75	AAI-based (high-confidence)
NODE_2571_length_3790_cov_0.146302	3790	No	11	1	Low-quality	12.75	AAI-based (high-confidence)
NODE_25711_length_1004_cov_0.155801	1004	No	1	1	Low-quality	2.19	AAI-based (high-confidence)
NODE_2572_length_2377_cov_0.129939	2377	No	3	2	Low-quality	5.73	AAI-based (high-confidence)
NODE_2572_length_3298_cov_0.286652	3298	No	3	3	Low-quality	5.97	AAI-based (high-confidence)
NODE_2578_length_1102_cov_0.097707	1102	No	3	2	Low-quality	2.84	AAI-based (high-confidence)
NODE_2579_length_3680_cov_0.356604	3680	No	6	3	Low-quality	7.66	AAI-based (high-confidence)
NODE_2579_length_3783_cov_0.106406	3783	No	5	5	Low-quality	7.08	AAI-based (high-confidence)
NODE_258_length_2533_cov_0.082169	2533	No	3	2	Low-quality	7.38	AAI-based (high-confidence)
NODE_258_length_2756_cov_0.092586	2756	No	7	2	Low-quality	8.05	AAI-based (high-confidence)
NODE_258_length_4778_cov_0.206882	4778	No	10	4	Low-quality	7.73	AAI-based (high-confidence)

NODE_2585_length_3961_cov_0.530813	3961	No	4	4	Low-quality	6.59	AAI-based (high-confidence)
NODE_2588_length_3956_cov_0.342494	3956	No	7	2	Low-quality	12.51	AAI-based (high-confidence)
NODE_2589_length_1101_cov_0.073852	1101	No	1	1	Low-quality	3.13	AAI-based (high-confidence)
NODE_259_length_11448_cov_0.202837	11448	No	11	4	Low-quality	14.01	AAI-based (high-confidence)
NODE_259_length_2269_cov_0.094931	2269	No	6	2	Low-quality	7.77	AAI-based (high-confidence)
NODE_259_length_2654_cov_0.109980	2654	No	6	3	Low-quality	4.55	AAI-based (high-confidence)
NODE_2591_length_1099_cov_0.093000	1099	No	2	2	Low-quality	2.66	AAI-based (high-confidence)
NODE_2591_length_1890_cov_0.297599	1890	No	2	2	Low-quality	3.15	AAI-based (high-confidence)
NODE_2596_length_3281_cov_0.155877	3281	No	4	1	Low-quality	9.05	AAI-based (high-confidence)
NODE_2599_length_3765_cov_0.186307	3765	No	9	2	Low-quality	6.86	AAI-based (high-confidence)
NODE_2600_length_1098_cov_0.052052	1098	No	2	2	Low-quality	2.45	AAI-based (high-confidence)
NODE_2602_length_3279_cov_0.475472	3279	No	7	1	Low-quality	5.49	AAI-based (high-confidence)
NODE_2602_length_3661_cov_0.160303	3661	No	8	2	Low-quality	5.94	AAI-based (high-confidence)
NODE_261_length_1992_cov_0.091918	1992	No	5	1	Low-quality	6.68	AAI-based (high-confidence)
NODE_261_length_2250_cov_0.105997	2250	No	6	3	Low-quality	3.68	AAI-based (high-confidence)
NODE_261_length_2521_cov_0.107349	2521	No	5	3	Low-quality	8.46	AAI-based (high-confidence)
NODE_2612_length_1767_cov_0.130096	1767	No	2	2	Low-quality	3.31	AAI-based (high-confidence)
NODE_262_length_2261_cov_0.101295	2261	No	2	1	Low-quality	33.42	AAI-based (high-confidence)
NODE_262_length_7520_cov_0.144455	7520	No	12	5	Low-quality	16.97	AAI-based (high-confidence)
NODE_2621_length_3649_cov_0.171549	3649	No	6	1	Low-quality	8.23	AAI-based (high-confidence)
NODE_2622_length_3649_cov_0.171268	3649	No	1	1	Low-quality	9.72	AAI-based (high-confidence)
NODE_2625_length_1094_cov_0.070352	1094	No	3	2	Low-quality	2.68	AAI-based (high-confidence)
NODE_2626_length_1094_cov_0.058291	1094	No	4	2	Low-quality	1.98	AAI-based (high-confidence)
NODE_2629_length_2344_cov_0.418263	2344	No	1	1	Low-quality	7.2	AAI-based (high-confidence)
NODE_2634_length_1131_cov_0.104651	1131	No	3	1	Low-quality	3.45	AAI-based (high-confidence)
NODE_2635_length_3912_cov_0.492788	3912	No	6	2	Low-quality	2.26	AAI-based (high-confidence)
NODE_2636_length_1876_cov_0.158132	1876	No	2	2	Low-quality	5.77	AAI-based (high-confidence)
NODE_2639_length_1876_cov_0.067530	1876	No	6	1	Low-quality	5.92	AAI-based (high-confidence)
NODE_264_length_16460_cov_0.461952	16460	No	15	7	Low-quality	44.6	AAI-based (high-confidence)
NODE_264_length_2256_cov_0.202133	2256	No	1	1	Low-quality	36.61	AAI-based (high-confidence)
NODE_264_length_4846_cov_0.539077	4846	No	9	2	Low-quality	12.88	AAI-based (high-confidence)
NODE_264_length_7467_cov_0.318132	7467	No	8	3	Low-quality	20.22	AAI-based (high-confidence)
NODE_2640_length_1875_cov_0.218468	1875	No	2	2	Low-quality	2.93	AAI-based (high-confidence)
NODE_2640_length_3909_cov_0.409449	3909	No	5	1	Low-quality	2.15	AAI-based (high-confidence)
NODE_2641_length_3734_cov_1.246217	3734	No	5	2	Low-quality	10.47	AAI-based (high-confidence)
NODE_2647_length_2336_cov_0.150201	2336	No	6	1	Low-quality	3.78	AAI-based (high-confidence)
NODE_2647_length_3732_cov_0.650702	3732	No	6	1	Low-quality	5.99	AAI-based (high-confidence)
NODE_265_length_2514_cov_0.151553	2514	No	4	2	Low-quality	5.04	AAI-based (high-confidence)
NODE_265_length_2619_cov_0.130556	2619	No	1	1	Low-quality	37.92	AAI-based (high-confidence)
NODE_265_length_2726_cov_0.189951	2726	No	1	1	Low-quality	48.69	AAI-based (high-confidence)
NODE_2651_length_1872_cov_0.226734	1872	No	3	3	Low-quality	2.98	AAI-based (high-confidence)
NODE_2653_length_1090_cov_0.069627	1090	No	3	1	Low-quality	1.95	AAI-based (high-confidence)
NODE_2655_length_3898_cov_0.134772	3898	No	4	2	Low-quality	6.42	AAI-based (high-confidence)
NODE_2658_length_3726_cov_0.309898	3726	No	6	6	Low-quality	8.75	AAI-based (high-confidence)
NODE_2659_length_2329_cov_0.095964	2329	No	4	3	Low-quality	7.21	AAI-based (high-confidence)
NODE_266_length_2514_cov_0.112215	2514	No	4	1	Low-quality	7.84	AAI-based (high-confidence)

NODE_266_length_7400_cov_0.238871	7400	No	10	4	Low-quality	23.04	AAI-based (high-confidence)
NODE_2666_length_3723_cov_0.496689	3723	No	8	2	Low-quality	10.08	AAI-based (high-confidence)
NODE_267_length_2250_cov_0.117155	2250	No	5	1	Low-quality	3.97	AAI-based (high-confidence)
NODE_267_length_4731_cov_0.212435	4731	No	5	5	Low-quality	8.19	AAI-based (high-confidence)
NODE_267_length_7398_cov_0.212084	7398	No	5	5	Low-quality	12.23	AAI-based (high-confidence)
NODE_2670_length_1088_cov_0.208291	1088	No	1	1	Low-quality	16.7	AAI-based (high-confidence)
NODE_2674_length_1865_cov_0.137599	1865	No	5	3	Low-quality	3.05	AAI-based (high-confidence)
NODE_2677_length_3884_cov_0.138177	3884	No	6	4	Low-quality	2.18	AAI-based (high-confidence)
NODE_2678_length_1434_cov_0.055431	1434	No	3	3	Low-quality	2.59	AAI-based (high-confidence)
NODE_2685_length_1085_cov_0.042596	1085	No	1	1	Low-quality	2.32	AAI-based (high-confidence)
NODE_2685_length_3877_cov_0.123346	3877	No	10	1	Low-quality	3.19	AAI-based (high-confidence)
NODE_2686_length_1742_cov_0.124163	1742	No	3	1	Low-quality	5.45	AAI-based (high-confidence)
NODE_2686_length_3708_cov_0.105569	3708	No	3	2	Low-quality	10.12	AAI-based (high-confidence)
NODE_2692_length_3593_cov_0.169720	3593	No	3	2	Low-quality	5.48	AAI-based (high-confidence)
NODE_2695_length_1860_cov_0.145372	1860	No	4	1	Low-quality	6.2	AAI-based (high-confidence)
NODE_27_length_2856_cov_0.153065	2856	No	5	2	Low-quality	8.41	AAI-based (high-confidence)
NODE_271_length_2241_cov_0.092437	2241	No	2	1	Low-quality	41.32	AAI-based (high-confidence)
NODE_272_length_2210_cov_0.127901	2210	No	3	1	Low-quality	3.37	AAI-based (high-confidence)
NODE_272_length_2597_cov_0.070456	2597	No	1	1	Low-quality	39.08	AAI-based (high-confidence)
NODE_2722_length_3576_cov_0.223469	3576	No	6	2	Low-quality	2.01	AAI-based (high-confidence)
NODE_2723_length_2297_cov_0.196997	2297	No	5	1	Low-quality	3.58	AAI-based (high-confidence)
NODE_2725_length_3197_cov_0.195933	3197	No	7	1	Low-quality	5.19	AAI-based (high-confidence)
NODE_2726_length_1077_cov_0.098160	1077	No	1	1	Low-quality	3.31	AAI-based (high-confidence)
NODE_2730_length_1077_cov_0.054192	1077	No	3	1	Low-quality	3.43	AAI-based (high-confidence)
NODE_2733_length_1727_cov_0.107494	1727	No	6	2	Low-quality	4.92	AAI-based (high-confidence)
NODE_2737_length_2292_cov_0.437301	2292	No	9	1	Low-quality	6.63	AAI-based (high-confidence)
NODE_274_length_2207_cov_0.087287	2207	No	4	1	Low-quality	3.84	AAI-based (high-confidence)
NODE_274_length_4777_cov_0.343309	4777	No	9	2	Low-quality	4.84	AAI-based (high-confidence)
NODE_2741_length_2290_cov_0.123231	2290	No	6	2	Low-quality	6.19	AAI-based (high-confidence)
NODE_2742_length_1847_cov_0.144737	1847	No	3	1	Low-quality	3	AAI-based (high-confidence)
NODE_2742_length_3672_cov_0.441086	3672	No	7	5	Low-quality	2.05	AAI-based (high-confidence)
NODE_2747_length_1112_cov_0.092794	1112	No	4	3	Low-quality	1.98	AAI-based (high-confidence)
NODE_275_length_2214_cov_0.157447	2214	No	4	2	Low-quality	4.26	AAI-based (high-confidence)
NODE_2756_length_1071_cov_0.105967	1071	No	1	1	Low-quality	19.23	AAI-based (high-confidence)
NODE_2756_length_3822_cov_0.273167	3822	No	5	3	Low-quality	1.92	AAI-based (high-confidence)
NODE_2757_length_3554_cov_0.186397	3554	No	10	1	Low-quality	11.36	AAI-based (high-confidence)
NODE_276_length_11067_cov_0.167761	11067	No	15	4	Low-quality	20.23	AAI-based (high-confidence)
NODE_276_length_4672_cov_0.147387	4672	No	8	1	Low-quality	14.66	AAI-based (high-confidence)
NODE_2763_length_2281_cov_0.268561	2281	No	7	1	Low-quality	3.56	AAI-based (high-confidence)
NODE_277_length_9927_cov_0.410053	9927	No	11	3	Low-quality	22.83	AAI-based (high-confidence)
NODE_2772_length_3163_cov_0.183747	3163	No	3	2	Low-quality	1.93	AAI-based (high-confidence)
NODE_2776_length_1069_cov_0.053608	1069	No	3	1	Low-quality	2.87	AAI-based (high-confidence)
NODE_2785_length_3805_cov_0.221802	3805	No	3	2	Low-quality	7.08	AAI-based (high-confidence)
NODE_2787_length_3530_cov_0.191489	3530	No	2	1	Low-quality	1.98	AAI-based (high-confidence)
NODE_2787_length_3644_cov_0.280959	3644	No	6	5	Low-quality	8.41	AAI-based (high-confidence)
NODE_279_length_2204_cov_0.142043	2204	No	5	1	Low-quality	6.72	AAI-based (high-confidence)

NODE_2792_length_2263_cov_0.151109	2263	No	10	2	Low-quality	3.58	AAI-based (high-confidence)
NODE_2792_length_3154_cov_0.178069	3154	No	4	1	Low-quality	1.93	AAI-based (high-confidence)
NODE_2792_length_3642_cov_0.211685	3642	No	5	3	Low-quality	3.73	AAI-based (high-confidence)
NODE_2797_length_2262_cov_0.141933	2262	No	5	3	Low-quality	4.17	AAI-based (high-confidence)
NODE_28_length_4406_cov_0.462271	4406	No	5	1	Low-quality	7.28	AAI-based (high-confidence)
NODE_28_length_5131_cov_0.233704	5131	No	8	4	Low-quality	10.27	AAI-based (high-confidence)
NODE_280_length_2206_cov_0.139535	2206	No	4	1	Low-quality	3.89	AAI-based (high-confidence)
NODE_280_length_2473_cov_0.151643	2473	No	4	1	Low-quality	5.26	AAI-based (high-confidence)
NODE_280_length_7133_cov_0.149275	7133	No	10	3	Low-quality	24.51	AAI-based (high-confidence)
NODE_2800_length_3150_cov_0.494264	3150	No	4	1	Low-quality	5.44	AAI-based (high-confidence)
NODE_281_length_2205_cov_0.121557	2205	No	1	1	Low-quality	34.47	AAI-based (high-confidence)
NODE_2811_length_3782_cov_0.145534	3782	No	8	2	Low-quality	2.15	AAI-based (high-confidence)
NODE_2812_length_1065_cov_0.056936	1065	No	2	1	Low-quality	3.36	AAI-based (high-confidence)
NODE_2812_length_1823_cov_0.165893	1823	No	3	1	Low-quality	3.01	AAI-based (high-confidence)
NODE_2815_length_1411_cov_0.085366	1411	No	2	1	Low-quality	3.37	AAI-based (high-confidence)
NODE_2815_length_3623_cov_0.461975	3623	No	6	2	Low-quality	8.69	AAI-based (high-confidence)
NODE_282_length_4740_cov_0.186813	4740	No	5	1	Low-quality	7.4	AAI-based (high-confidence)
NODE_282_length_9832_cov_0.466249	9832	No	13	9	Low-quality	5.52	AAI-based (high-confidence)
NODE_2820_length_3137_cov_0.140553	3137	No	5	1	Low-quality	5.72	AAI-based (high-confidence)
NODE_2822_length_3619_cov_0.158239	3619	No	6	1	Low-quality	2.22	AAI-based (high-confidence)
NODE_2825_length_2249_cov_0.160000	2249	No	2	2	Low-quality	3.71	AAI-based (high-confidence)
NODE_2828_length_3495_cov_0.143993	3495	No	10	5	Low-quality	11.31	AAI-based (high-confidence)
NODE_283_length_10836_cov_0.413896	10836	No	11	4	Low-quality	7.21	AAI-based (high-confidence)
NODE_283_length_2562_cov_0.160780	2562	No	1	1	Low-quality	40.18	AAI-based (high-confidence)
NODE_2837_length_3771_cov_0.723856	3771	No	5	2	Low-quality	10.21	AAI-based (high-confidence)
NODE_284_length_10829_cov_0.283597	10829	No	14	6	Low-quality	4.88	AAI-based (high-confidence)
NODE_2842_length_3766_cov_0.091083	3766	No	11	3	Low-quality	6.21	AAI-based (high-confidence)
NODE_2845_length_3762_cov_0.276276	3762	No	5	2	Low-quality	1.96	AAI-based (high-confidence)
NODE_2849_length_3599_cov_0.150571	3599	No	4	1	Low-quality	9.8	AAI-based (high-confidence)
NODE_285_length_4717_cov_0.108489	4717	No	4	2	Low-quality	3.11	AAI-based (high-confidence)
NODE_2853_length_2239_cov_0.144393	2239	No	1	1	Low-quality	3.95	AAI-based (high-confidence)
NODE_2854_length_3595_cov_0.148741	3595	No	5	3	Low-quality	1.98	AAI-based (high-confidence)
NODE_2854_length_4471_cov_1.867338	4471	No	3	1	Low-quality	12.81	AAI-based (high-confidence)
NODE_2855_length_1058_cov_0.062565	1058	No	2	1	Low-quality	2.54	AAI-based (high-confidence)
NODE_2857_length_3756_cov_0.141920	3756	No	8	3	Low-quality	10.43	AAI-based (high-confidence)
NODE_2859_length_1688_cov_0.144745	1688	No	2	2	Low-quality	5.18	AAI-based (high-confidence)
NODE_286_length_2561_cov_0.067425	2561	No	6	1	Low-quality	8.69	AAI-based (high-confidence)
NODE_2860_length_1405_cov_0.084227	1405	No	2	1	Low-quality	3.54	AAI-based (high-confidence)
NODE_2861_length_3589_cov_0.225501	3589	No	10	2	Low-quality	10.66	AAI-based (high-confidence)
NODE_2862_length_3751_cov_1.065444	3751	No	4	4	Low-quality	2.09	AAI-based (high-confidence)
NODE_2868_length_3108_cov_0.334995	3108	No	4	1	Low-quality	5.79	AAI-based (high-confidence)
NODE_2869_length_3583_cov_0.562285	3583	No	13	2	Low-quality	10.07	AAI-based (high-confidence)
NODE_287_length_2772_cov_0.156004	2772	No	5	3	Low-quality	6.63	AAI-based (high-confidence)
NODE_2870_length_3744_cov_0.391221	3744	No	10	2	Low-quality	11.92	AAI-based (high-confidence)
NODE_288_length_7040_cov_0.143495	7040	No	11	6	Low-quality	6.78	AAI-based (high-confidence)
NODE_2880_length_3573_cov_0.198043	3573	No	11	3	Low-quality	10.33	AAI-based (high-confidence)

NODE_289_length_2200_cov_0.130414	2200	No	1	1	Low-quality	34.39	AAI-based (high-confidence)
NODE_2890_length_3564_cov_0.263203	3564	No	6	2	Low-quality	6.52	AAI-based (high-confidence)
NODE_2892_length_1798_cov_0.220129	1798	No	6	2	Low-quality	6.19	AAI-based (high-confidence)
NODE_2892_length_3725_cov_0.527854	3725	No	4	4	Low-quality	2.11	AAI-based (high-confidence)
NODE_2894_length_1675_cov_0.135787	1675	No	4	1	Low-quality	5.23	AAI-based (high-confidence)
NODE_2896_length_3562_cov_0.102512	3562	No	5	3	Low-quality	7.46	AAI-based (high-confidence)
NODE_29_length_11983_cov_0.243016	11983	No	16	7	Low-quality	19.91	AAI-based (high-confidence)
NODE_29_length_4546_cov_0.156510	4546	No	8	2	Low-quality	10.56	AAI-based (high-confidence)
NODE_290_length_10721_cov_0.345792	10721	No	15	6	Low-quality	5.14	AAI-based (high-confidence)
NODE_2904_length_4419_cov_0.168056	4419	No	7	3	Low-quality	12.86	AAI-based (high-confidence)
NODE_2905_length_1673_cov_0.291614	1673	No	5	2	Low-quality	2.6	AAI-based (high-confidence)
NODE_2905_length_2217_cov_0.097262	2217	No	3	1	Low-quality	3.4	AAI-based (high-confidence)
NODE_2908_length_3554_cov_0.097250	3554	No	7	1	Low-quality	5.92	AAI-based (high-confidence)
NODE_2909_length_3086_cov_0.187144	3086	No	5	2	Low-quality	7.12	AAI-based (high-confidence)
NODE_2909_length_3553_cov_0.193399	3553	No	6	2	Low-quality	14.37	AAI-based (high-confidence)
NODE_291_length_15363_cov_0.256093	15363	No	10	5	Low-quality	7.28	AAI-based (high-confidence)
NODE_291_length_4697_cov_0.190300	4697	No	4	4	Low-quality	7.78	AAI-based (high-confidence)
NODE_2911_length_1792_cov_0.180154	1792	No	3	2	Low-quality	3.67	AAI-based (high-confidence)
NODE_2912_length_2214_cov_0.059574	2214	No	3	2	Low-quality	5	AAI-based (high-confidence)
NODE_2913_length_3083_cov_0.488271	3083	No	5	1	Low-quality	3.59	AAI-based (high-confidence)
NODE_2914_length_3708_cov_0.169299	3708	No	7	2	Low-quality	8.42	AAI-based (high-confidence)
NODE_2917_length_3078_cov_0.213494	3078	No	7	1	Low-quality	2.31	AAI-based (high-confidence)
NODE_2919_length_1790_cov_0.442933	1790	No	1	1	Low-quality	5.66	AAI-based (high-confidence)
NODE_292_length_15329_cov_0.744977	15329	No	19	12	Low-quality	48.09	AAI-based (high-confidence)
NODE_292_length_9687_cov_0.177305	9687	No	13	3	Low-quality	17.9	AAI-based (high-confidence)
NODE_2922_length_1670_cov_0.098663	1670	No	4	2	Low-quality	5.25	AAI-based (high-confidence)
NODE_2924_length_1046_cov_0.057022	1046	No	1	1	Low-quality	3.18	AAI-based (high-confidence)
NODE_2925_length_3074_cov_0.109580	3074	No	5	1	Low-quality	4.44	AAI-based (high-confidence)
NODE_2927_length_3541_cov_0.194073	3541	No	7	2	Low-quality	10.65	AAI-based (high-confidence)
NODE_293_length_2644_cov_0.191356	2644	No	6	2	Low-quality	5.2	AAI-based (high-confidence)
NODE_293_length_7006_cov_0.174316	7006	No	6	2	Low-quality	21.03	AAI-based (high-confidence)
NODE_2933_length_3537_cov_0.141943	3537	No	4	3	Low-quality	6.38	AAI-based (high-confidence)
NODE_2936_length_1784_cov_0.435015	1784	No	3	1	Low-quality	5.01	AAI-based (high-confidence)
NODE_2938_length_1667_cov_0.156888	1667	No	2	1	Low-quality	2.72	AAI-based (high-confidence)
NODE_2939_length_1392_cov_0.041763	1392	No	3	2	Low-quality	3.89	AAI-based (high-confidence)
NODE_294_length_1928_cov_0.131219	1928	No	2	1	Low-quality	5.55	AAI-based (high-confidence)
NODE_294_length_2419_cov_0.175431	2419	No	1	1	Low-quality	37.43	AAI-based (high-confidence)
NODE_2942_length_1783_cov_0.086698	1783	No	3	1	Low-quality	3.61	AAI-based (high-confidence)
NODE_2944_length_2204_cov_0.152969	2204	No	6	2	Low-quality	6.14	AAI-based (high-confidence)
NODE_2944_length_3529_cov_0.730904	3529	No	5	3	Low-quality	9.51	AAI-based (high-confidence)
NODE_2945_length_2203_cov_0.525190	2203	No	5	1	Low-quality	3.84	AAI-based (high-confidence)
NODE_2945_length_3684_cov_0.893724	3684	No	7	1	Low-quality	4.52	AAI-based (high-confidence)
NODE_2948_length_1664_cov_0.162939	1664	No	2	2	Low-quality	2.46	AAI-based (high-confidence)
NODE_295_length_15264_cov_0.209034	15264	No	28	13	Low-quality	47.5	AAI-based (high-confidence)
NODE_2957_length_2196_cov_0.245112	2196	No	3	3	Low-quality	3.85	AAI-based (high-confidence)
NODE_296_length_4669_cov_0.137418	4669	No	6	1	Low-quality	4.64	AAI-based (high-confidence)

NODE_296_length_6977_cov_0.232771	6977	No	10	4	Low-quality	16.08	AAI-based (high-confidence)
NODE_2966_length_1776_cov_0.149076	1776	No	3	1	Low-quality	5.37	AAI-based (high-confidence)
NODE_2966_length_2192_cov_0.099379	2192	No	3	3	Low-quality	6.03	AAI-based (high-confidence)
NODE_2968_length_3514_cov_0.091362	3514	No	13	3	Low-quality	10.45	AAI-based (high-confidence)
NODE_297_length_2414_cov_0.130454	2414	No	3	3	Low-quality	4.2	AAI-based (high-confidence)
NODE_2970_length_1776_cov_0.085868	1776	No	2	2	Low-quality	3.09	AAI-based (high-confidence)
NODE_298_length_11433_cov_0.287454	11433	No	11	3	Low-quality	33.31	AAI-based (high-confidence)
NODE_2980_length_2188_cov_0.096218	2188	No	3	1	Low-quality	3.98	AAI-based (high-confidence)
NODE_2983_length_1037_cov_0.053305	1037	No	4	1	Low-quality	2.11	AAI-based (high-confidence)
NODE_299_length_2528_cov_0.228901	2528	No	1	1	Low-quality	45.23	AAI-based (high-confidence)
NODE_2999_length_1767_cov_0.094125	1767	No	1	1	Low-quality	4.33	AAI-based (high-confidence)
NODE_3_length_17856_cov_0.200766	17856	No	25	12	Low-quality	49.94	AAI-based (high-confidence)
NODE_3_length_20346_cov_0.525263	20346	No	25	8	Low-quality	33.3	AAI-based (high-confidence)
NODE_3_length_24549_cov_0.707280	24549	No	33	4	Low-quality	41	AAI-based (high-confidence)
NODE_300_length_4647_cov_0.183817	4647	No	7	4	Low-quality	4.17	AAI-based (high-confidence)
NODE_300_length_6946_cov_0.124142	6946	No	9	5	Low-quality	15.62	AAI-based (high-confidence)
NODE_3003_length_2178_cov_0.119288	2178	No	5	1	Low-quality	2.21	AAI-based (high-confidence)
NODE_3005_length_3029_cov_0.202048	3029	No	2	2	Low-quality	5	AAI-based (high-confidence)
NODE_3005_length_3490_cov_0.120613	3490	No	5	1	Low-quality	5.04	AAI-based (high-confidence)
NODE_3009_length_2176_cov_0.098700	2176	No	4	2	Low-quality	5.37	AAI-based (high-confidence)
NODE_301_length_10542_cov_0.169108	10542	No	15	7	Low-quality	41.38	AAI-based (high-confidence)
NODE_3011_length_3639_cov_0.160734	3639	No	7	5	Low-quality	12.01	AAI-based (high-confidence)
NODE_3013_length_1646_cov_0.252101	1646	No	4	1	Low-quality	5.37	AAI-based (high-confidence)
NODE_3013_length_3638_cov_0.572478	3638	No	8	2	Low-quality	6.03	AAI-based (high-confidence)
NODE_3015_length_1072_cov_0.049332	1072	No	1	1	Low-quality	2.32	AAI-based (high-confidence)
NODE_3018_length_2171_cov_0.126448	2171	No	4	1	Low-quality	5.79	AAI-based (high-confidence)
NODE_302_length_1661_cov_0.019206	1661	No	1	1	Low-quality	4.92	AAI-based (high-confidence)
NODE_302_length_4508_cov_0.136085	4508	No	12	2	Low-quality	14.09	AAI-based (high-confidence)
NODE_3022_length_3370_cov_0.115867	3370	No	6	2	Low-quality	3.42	AAI-based (high-confidence)
NODE_3027_length_3631_cov_0.238958	3631	No	4	2	Low-quality	2.13	AAI-based (high-confidence)
NODE_3028_length_1033_cov_0.072805	1033	No	3	1	Low-quality	2.35	AAI-based (high-confidence)
NODE_3028_length_1641_cov_0.113489	1641	No	3	1	Low-quality	4.66	AAI-based (high-confidence)
NODE_3030_length_1070_cov_0.098867	1070	No	2	1	Low-quality	2.78	AAI-based (high-confidence)
NODE_3031_length_3474_cov_0.680889	3474	No	7	3	Low-quality	8.95	AAI-based (high-confidence)
NODE_304_length_10501_cov_0.447414	10501	No	22	9	Low-quality	17	AAI-based (high-confidence)
NODE_3049_length_1031_cov_0.068670	1031	No	1	1	Low-quality	2.81	AAI-based (high-confidence)
NODE_3053_length_3462_cov_0.167113	3462	No	9	2	Low-quality	6.3	AAI-based (high-confidence)
NODE_3056_length_1754_cov_0.215710	1754	No	3	3	Low-quality	3	AAI-based (high-confidence)
NODE_3058_length_3459_cov_0.524107	3459	No	3	3	Low-quality	10.95	AAI-based (high-confidence)
NODE_3061_length_1029_cov_0.076344	1029	No	1	1	Low-quality	2.59	AAI-based (high-confidence)
NODE_3061_length_2151_cov_0.082846	2151	No	4	1	Low-quality	4.74	AAI-based (high-confidence)
NODE_3068_length_2148_cov_0.614446	2148	No	2	1	Low-quality	6.5	AAI-based (high-confidence)
NODE_3068_length_2998_cov_0.177647	2998	No	5	3	Low-quality	5.7	AAI-based (high-confidence)
NODE_3069_length_3608_cov_0.635794	3608	No	3	1	Low-quality	11.36	AAI-based (high-confidence)
NODE_307_length_2512_cov_0.134687	2512	No	2	1	Low-quality	40.05	AAI-based (high-confidence)
NODE_3073_length_3452_cov_0.198926	3452	No	4	4	Low-quality	10.19	AAI-based (high-confidence)

NODE_3076_length_1628_cov_0.130150	1628	No	4	2	Low-quality	5.16	AAI-based (high-confidence)
NODE_3082_length_3599_cov_0.186286	3599	No	4	1	Low-quality	10.91	AAI-based (high-confidence)
NODE_309_length_14838_cov_0.206188	14838	No	36	2	Low-quality	9.31	AAI-based (high-confidence)
NODE_3093_length_3444_cov_0.124365	3444	No	5	3	Low-quality	10.72	AAI-based (high-confidence)
NODE_3094_length_2982_cov_0.161984	2982	No	2	2	Low-quality	5.55	AAI-based (high-confidence)
NODE_3095_length_1741_cov_0.233252	1741	No	1	1	Low-quality	3.87	AAI-based (high-confidence)
NODE_3096_length_2138_cov_0.065228	2138	No	2	2	Low-quality	4.95	AAI-based (high-confidence)
NODE_31_length_11760_cov_0.184890	11760	No	2	2	Low-quality	4.09	AAI-based (high-confidence)
NODE_310_length_2148_cov_0.128843	2148	No	1	1	Low-quality	31.39	AAI-based (high-confidence)
NODE_310_length_2507_cov_0.132475	2507	No	3	1	Low-quality	8	AAI-based (high-confidence)
NODE_3101_length_1023_cov_0.062771	1023	No	1	1	Low-quality	2.28	AAI-based (high-confidence)
NODE_3108_length_3435_cov_0.100120	3435	No	5	2	Low-quality	9.37	AAI-based (high-confidence)
NODE_3112_length_1021_cov_0.061822	1021	No	1	1	Low-quality	14	AAI-based (high-confidence)
NODE_3116_length_1059_cov_0.110417	1059	No	6	1	Low-quality	2.55	AAI-based (high-confidence)
NODE_3117_length_3572_cov_0.583069	3572	No	8	2	Low-quality	5.78	AAI-based (high-confidence)
NODE_3119_length_1020_cov_0.072747	1020	No	1	1	Low-quality	14.58	AAI-based (high-confidence)
NODE_312_length_2378_cov_0.088197	2378	No	3	1	Low-quality	7	AAI-based (high-confidence)
NODE_3121_length_2126_cov_0.266897	2126	No	4	2	Low-quality	6.67	AAI-based (high-confidence)
NODE_3130_length_2124_cov_0.108642	2124	No	2	1	Low-quality	3.33	AAI-based (high-confidence)
NODE_3134_length_2123_cov_0.224308	2123	No	2	2	Low-quality	3.58	AAI-based (high-confidence)
NODE_3138_length_2963_cov_0.343925	2963	No	4	1	Low-quality	2.77	AAI-based (high-confidence)
NODE_3140_length_2121_cov_0.145401	2121	No	2	2	Low-quality	6.63	AAI-based (high-confidence)
NODE_3146_length_4187_cov_0.301370	4187	No	8	3	Low-quality	8.21	AAI-based (high-confidence)
NODE_3147_length_2959_cov_0.161538	2959	No	4	1	Low-quality	4.86	AAI-based (high-confidence)
NODE_3149_length_2957_cov_0.251225	2957	No	6	2	Low-quality	9.12	AAI-based (high-confidence)
NODE_3155_length_1356_cov_0.044551	1356	No	1	1	Low-quality	3.34	AAI-based (high-confidence)
NODE_3155_length_1727_cov_0.186118	1727	No	3	1	Low-quality	5.31	AAI-based (high-confidence)
NODE_3157_length_1055_cov_0.087866	1055	No	2	2	Low-quality	2.65	AAI-based (high-confidence)
NODE_3158_length_1055_cov_0.083682	1055	No	2	1	Low-quality	2.21	AAI-based (high-confidence)
NODE_3161_length_4170_cov_0.362564	4170	No	5	3	Low-quality	7.46	AAI-based (high-confidence)
NODE_317_length_2493_cov_0.106099	2493	No	7	1	Low-quality	6.78	AAI-based (high-confidence)
NODE_3171_length_1723_cov_0.201970	1723	No	5	1	Low-quality	2.83	AAI-based (high-confidence)
NODE_3171_length_3285_cov_0.158820	3285	No	7	1	Low-quality	5.44	AAI-based (high-confidence)
NODE_3175_length_3537_cov_0.262362	3537	No	7	3	Low-quality	6.58	AAI-based (high-confidence)
NODE_318_length_2135_cov_0.091356	2135	No	5	2	Low-quality	3.63	AAI-based (high-confidence)
NODE_3187_length_1052_cov_0.073452	1052	No	3	1	Low-quality	2.17	AAI-based (high-confidence)
NODE_3187_length_3383_cov_0.531060	3383	No	10	5	Low-quality	10.41	AAI-based (high-confidence)
NODE_319_length_2132_cov_0.153468	2132	No	1	1	Low-quality	34.04	AAI-based (high-confidence)
NODE_3190_length_2940_cov_0.117564	2940	No	6	3	Low-quality	5.94	AAI-based (high-confidence)
NODE_32_length_4303_cov_0.084681	4303	No	8	5	Low-quality	6.95	AAI-based (high-confidence)
NODE_3205_length_1049_cov_0.093684	1049	No	4	1	Low-quality	2.36	AAI-based (high-confidence)
NODE_3205_length_2933_cov_0.212421	2933	No	7	1	Low-quality	6.79	AAI-based (high-confidence)
NODE_3206_length_2097_cov_0.173173	2097	No	2	1	Low-quality	3.91	AAI-based (high-confidence)
NODE_3207_length_3376_cov_0.137016	3376	No	4	4	Low-quality	3.02	AAI-based (high-confidence)
NODE_3213_length_3508_cov_0.336462	3508	No	4	3	Low-quality	5.77	AAI-based (high-confidence)
NODE_3216_length_1713_cov_0.200124	1713	No	6	1	Low-quality	5.56	AAI-based (high-confidence)

NODE_3216_length_2093_cov_0.122367	2093	No	2	1	Low-quality	5.05	AAI-based (high-confidence)
NODE_322_length_10250_cov_0.351394	10250	No	9	7	Low-quality	23.32	AAI-based (high-confidence)
NODE_3221_length_2090_cov_0.098443	2090	No	4	2	Low-quality	6.88	AAI-based (high-confidence)
NODE_3227_length_1001_cov_0.044346	1001	No	3	1	Low-quality	2.47	AAI-based (high-confidence)
NODE_3229_length_3360_cov_0.550751	3360	No	4	1	Low-quality	3.23	AAI-based (high-confidence)
NODE_323_length_2130_cov_0.111275	2130	No	4	2	Low-quality	3.47	AAI-based (high-confidence)
NODE_323_length_2145_cov_0.087977	2145	No	3	3	Low-quality	6.37	AAI-based (high-confidence)
NODE_3236_length_3356_cov_0.525944	3356	No	7	1	Low-quality	10.02	AAI-based (high-confidence)
NODE_324_length_2142_cov_0.091532	2142	No	5	1	Low-quality	7.27	AAI-based (high-confidence)
NODE_3240_length_1004_cov_0.088398	1004	No	3	1	Low-quality	2.54	AAI-based (high-confidence)
NODE_3242_length_3493_cov_0.539187	3493	No	12	2	Low-quality	9.79	AAI-based (high-confidence)
NODE_3243_length_1003_cov_0.231195	1003	No	2	2	Low-quality	2	AAI-based (high-confidence)
NODE_3244_length_1043_cov_0.148305	1043	No	1	1	Low-quality	15.36	AAI-based (high-confidence)
NODE_3245_length_1043_cov_0.082627	1043	No	4	1	Low-quality	2.29	AAI-based (high-confidence)
NODE_3247_length_2079_cov_0.235354	2079	No	2	1	Low-quality	6.01	AAI-based (high-confidence)
NODE_3249_length_2079_cov_0.150000	2079	No	3	2	Low-quality	5.45	AAI-based (high-confidence)
NODE_3249_length_3236_cov_0.147274	3236	No	4	1	Low-quality	3.29	AAI-based (high-confidence)
NODE_3249_length_3347_cov_0.140702	3347	No	4	2	Low-quality	9.26	AAI-based (high-confidence)
NODE_325_length_2477_cov_0.097561	2477	No	5	3	Low-quality	6.25	AAI-based (high-confidence)
NODE_3250_length_1706_cov_0.065961	1706	No	7	3	Low-quality	3.12	AAI-based (high-confidence)
NODE_3250_length_2079_cov_0.079798	2079	No	3	1	Low-quality	8.35	AAI-based (high-confidence)
NODE_3252_length_1342_cov_0.065969	1342	No	3	2	Low-quality	2.16	AAI-based (high-confidence)
NODE_3259_length_3340_cov_0.135761	3340	No	4	2	Low-quality	1.93	AAI-based (high-confidence)
NODE_326_length_2100_cov_0.080460	2100	No	2	1	Low-quality	6.98	AAI-based (high-confidence)
NODE_3260_length_3340_cov_0.130824	3340	No	10	2	Low-quality	8.54	AAI-based (high-confidence)
NODE_3263_length_3478_cov_0.317550	3478	No	8	2	Low-quality	10.38	AAI-based (high-confidence)
NODE_3265_length_3337_cov_0.144225	3337	No	5	3	Low-quality	10.76	AAI-based (high-confidence)
NODE_3270_length_3472_cov_1.025497	3472	No	4	2	Low-quality	9.67	AAI-based (high-confidence)
NODE_3274_length_2898_cov_0.117899	2898	No	5	2	Low-quality	11.35	AAI-based (high-confidence)
NODE_3275_length_3220_cov_0.210509	3220	No	4	2	Low-quality	9.83	AAI-based (high-confidence)
NODE_328_length_2140_cov_0.141107	2140	No	1	1	Low-quality	31.54	AAI-based (high-confidence)
NODE_328_length_4451_cov_0.513097	4451	No	6	2	Low-quality	12.89	AAI-based (high-confidence)
NODE_3281_length_2895_cov_0.150930	2895	No	6	3	Low-quality	5.06	AAI-based (high-confidence)
NODE_3283_length_3325_cov_0.132982	3325	No	6	1	Low-quality	11.07	AAI-based (high-confidence)
NODE_3285_length_3214_cov_0.190048	3214	No	5	2	Low-quality	10.01	AAI-based (high-confidence)
NODE_329_length_4441_cov_0.203823	4441	No	7	2	Low-quality	2.27	AAI-based (high-confidence)
NODE_3290_length_2889_cov_0.310036	2889	No	3	1	Low-quality	3.97	AAI-based (high-confidence)
NODE_3295_length_2886_cov_0.147470	2886	No	4	4	Low-quality	5.25	AAI-based (high-confidence)
NODE_3296_length_2065_cov_0.398271	2065	No	3	1	Low-quality	5.76	AAI-based (high-confidence)
NODE_33_length_4302_cov_0.080657	4302	No	4	1	Low-quality	13.61	AAI-based (high-confidence)
NODE_330_length_2118_cov_0.182268	2118	No	1	1	Low-quality	26.45	AAI-based (high-confidence)
NODE_3304_length_1574_cov_0.147119	1574	No	4	2	Low-quality	4.26	AAI-based (high-confidence)
NODE_331_length_10069_cov_0.281244	10069	No	11	8	Low-quality	5.57	AAI-based (high-confidence)
NODE_331_length_2090_cov_0.104972	2090	No	3	1	Low-quality	6.56	AAI-based (high-confidence)
NODE_3312_length_1334_cov_0.098785	1334	No	2	1	Low-quality	4.18	AAI-based (high-confidence)
NODE_3312_length_3447_cov_0.242234	3447	No	3	2	Low-quality	9.52	AAI-based (high-confidence)

NODE_332_length_2126_cov_0.345338	2126	No	1	1	Low-quality	33.86	AAI-based (high-confidence)
NODE_332_length_2451_cov_0.127551	2451	No	1	1	Low-quality	38.86	AAI-based (high-confidence)
NODE_3321_length_1034_cov_0.086631	1034	No	4	1	Low-quality	2.22	AAI-based (high-confidence)
NODE_3323_length_1688_cov_0.298930	1688	No	5	2	Low-quality	5.04	AAI-based (high-confidence)
NODE_3326_length_3441_cov_0.269599	3441	No	6	2	Low-quality	10.44	AAI-based (high-confidence)
NODE_3329_length_3440_cov_0.210416	3440	No	6	1	Low-quality	6.45	AAI-based (high-confidence)
NODE_3335_length_3439_cov_0.143413	3439	No	5	4	Low-quality	7.73	AAI-based (high-confidence)
NODE_3337_length_2054_cov_0.151407	2054	No	5	2	Low-quality	5.57	AAI-based (high-confidence)
NODE_3337_length_3436_cov_0.222056	3436	No	5	2	Low-quality	2.26	AAI-based (high-confidence)
NODE_3344_length_4022_cov_0.567678	4022	No	11	1	Low-quality	12.44	AAI-based (high-confidence)
NODE_3346_length_2867_cov_0.098988	2867	No	3	3	Low-quality	6.51	AAI-based (high-confidence)
NODE_3348_length_1685_cov_0.390921	1685	No	2	2	Low-quality	5.28	AAI-based (high-confidence)
NODE_335_length_4422_cov_0.149896	4422	No	3	2	Low-quality	7.7	AAI-based (high-confidence)
NODE_3354_length_3176_cov_0.116997	3176	No	7	1	Low-quality	12.45	AAI-based (high-confidence)
NODE_3355_length_2863_cov_0.169320	2863	No	2	1	Low-quality	2.81	AAI-based (high-confidence)
NODE_3356_length_3286_cov_0.108252	3286	No	2	2	Low-quality	6.08	AAI-based (high-confidence)
NODE_3359_length_2862_cov_0.157076	2862	No	5	3	Low-quality	4.86	AAI-based (high-confidence)
NODE_336_length_10010_cov_0.157704	10010	No	5	5	Low-quality	4.51	AAI-based (high-confidence)
NODE_3360_length_2862_cov_0.151285	2862	No	5	3	Low-quality	5.33	AAI-based (high-confidence)
NODE_3362_length_3282_cov_0.101791	3282	No	5	3	Low-quality	5.99	AAI-based (high-confidence)
NODE_3365_length_3421_cov_0.875978	3421	No	2	1	Low-quality	8.86	AAI-based (high-confidence)
NODE_3368_length_2859_cov_0.573188	2859	No	3	1	Low-quality	6.69	AAI-based (high-confidence)
NODE_337_length_2105_cov_0.032403	2105	No	3	1	Low-quality	5.79	AAI-based (high-confidence)
NODE_337_length_2123_cov_0.103755	2123	No	2	2	Low-quality	3.52	AAI-based (high-confidence)
NODE_337_length_4418_cov_0.223894	4418	No	8	3	Low-quality	8.71	AAI-based (high-confidence)
NODE_337_length_6597_cov_0.560172	6597	No	16	3	Low-quality	18.3	AAI-based (high-confidence)
NODE_3371_length_1028_cov_0.067815	1028	No	2	1	Low-quality	2.35	AAI-based (high-confidence)
NODE_3374_length_3415_cov_0.193908	3415	No	5	3	Low-quality	6.34	AAI-based (high-confidence)
NODE_338_length_4304_cov_0.213555	4304	No	12	2	Low-quality	16.62	AAI-based (high-confidence)
NODE_338_length_9090_cov_1.042932	9090	No	17	2	Low-quality	9.92	AAI-based (high-confidence)
NODE_3381_length_1677_cov_0.075412	1677	No	6	1	Low-quality	4.44	AAI-based (high-confidence)
NODE_3382_length_1561_cov_0.170999	1561	No	4	2	Low-quality	4.85	AAI-based (high-confidence)
NODE_3382_length_1677_cov_0.061470	1677	No	4	1	Low-quality	3.14	AAI-based (high-confidence)
NODE_3387_length_1675_cov_0.119289	1675	No	3	1	Low-quality	5.12	AAI-based (high-confidence)
NODE_339_length_10003_cov_0.277060	10003	No	12	7	Low-quality	5.62	AAI-based (high-confidence)
NODE_3396_length_1324_cov_0.065306	1324	No	2	1	Low-quality	2.23	AAI-based (high-confidence)
NODE_3397_length_3405_cov_0.254991	3405	No	4	2	Low-quality	6.22	AAI-based (high-confidence)
NODE_34_length_11425_cov_0.468480	11425	No	10	3	Low-quality	33.11	AAI-based (high-confidence)
NODE_34_length_4187_cov_0.169765	4187	No	5	3	Low-quality	8.18	AAI-based (high-confidence)
NODE_34_length_4389_cov_0.111888	4389	No	10	4	Low-quality	7.55	AAI-based (high-confidence)
NODE_34_length_4440_cov_0.125317	4440	No	7	3	Low-quality	10.58	AAI-based (high-confidence)
NODE_34_length_4842_cov_0.155809	4842	No	11	1	Low-quality	14.75	AAI-based (high-confidence)
NODE_340_length_13739_cov_0.525367	13739	No	20	6	Low-quality	9.58	AAI-based (high-confidence)
NODE_340_length_2075_cov_0.114879	2075	No	1	1	Low-quality	44.13	AAI-based (high-confidence)
NODE_340_length_9997_cov_0.475652	9997	No	13	4	Low-quality	5.6	AAI-based (high-confidence)
NODE_3400_length_3404_cov_0.188502	3404	No	4	3	Low-quality	8.37	AAI-based (high-confidence)

NODE_3402_length_1672_cov_0.124603	1672	No	1	1	Low-quality	2.63	AAI-based (high-confidence)
NODE_3403_length_1553_cov_0.378267	1553	No	3	1	Low-quality	4.29	AAI-based (high-confidence)
NODE_3404_length_3988_cov_0.149653	3988	No	4	3	Low-quality	7.23	AAI-based (high-confidence)
NODE_3406_length_3401_cov_0.250454	3401	No	6	3	Low-quality	2	AAI-based (high-confidence)
NODE_3414_length_1669_cov_0.261146	1669	No	4	3	Low-quality	4.3	AAI-based (high-confidence)
NODE_3420_length_3139_cov_0.380263	3139	No	6	3	Low-quality	7.61	AAI-based (high-confidence)
NODE_3423_length_2837_cov_0.345508	2837	No	4	2	Low-quality	2.58	AAI-based (high-confidence)
NODE_3428_length_3137_cov_0.191573	3137	No	6	4	Low-quality	8.15	AAI-based (high-confidence)
NODE_343_length_2426_cov_0.123765	2426	No	4	3	Low-quality	3.93	AAI-based (high-confidence)
NODE_3433_length_1667_cov_0.058036	1667	No	4	3	Low-quality	3.05	AAI-based (high-confidence)
NODE_3434_length_2027_cov_0.155602	2027	No	3	3	Low-quality	3.73	AAI-based (high-confidence)
NODE_3437_length_1666_cov_0.106573	1666	No	5	1	Low-quality	4.5	AAI-based (high-confidence)
NODE_3439_length_3960_cov_0.161875	3960	No	9	1	Low-quality	7.69	AAI-based (high-confidence)
NODE_344_length_4270_cov_0.331335	4270	No	5	4	Low-quality	7.27	AAI-based (high-confidence)
NODE_3440_length_2831_cov_0.779649	2831	No	5	1	Low-quality	8.73	AAI-based (high-confidence)
NODE_3445_length_1545_cov_0.098202	1545	No	2	1	Low-quality	5.43	AAI-based (high-confidence)
NODE_3454_length_3127_cov_0.194188	3127	No	3	3	Low-quality	5.19	AAI-based (high-confidence)
NODE_3455_length_3237_cov_0.478011	3237	No	4	1	Low-quality	7.15	AAI-based (high-confidence)
NODE_3458_length_2826_cov_0.103777	2826	No	4	2	Low-quality	5.44	AAI-based (high-confidence)
NODE_346_length_2463_cov_0.139594	2463	No	1	1	Low-quality	37.58	AAI-based (high-confidence)
NODE_3460_length_3370_cov_0.464384	3370	No	7	1	Low-quality	5.64	AAI-based (high-confidence)
NODE_3465_length_3232_cov_0.182892	3232	No	5	2	Low-quality	6.94	AAI-based (high-confidence)
NODE_3469_length_3115_cov_0.701260	3115	No	5	2	Low-quality	8.71	AAI-based (high-confidence)
NODE_3469_length_3937_cov_0.895518	3937	No	7	1	Low-quality	6.76	AAI-based (high-confidence)
NODE_347_length_6479_cov_0.489185	6479	No	8	7	Low-quality	3.66	AAI-based (high-confidence)
NODE_3474_length_2010_cov_0.163265	2010	No	3	1	Low-quality	2.66	AAI-based (high-confidence)
NODE_3476_length_1540_cov_0.149896	1540	No	2	2	Low-quality	2.53	AAI-based (high-confidence)
NODE_3476_length_3227_cov_0.249680	3227	No	8	2	Low-quality	5.3	AAI-based (high-confidence)
NODE_348_length_6477_cov_0.129664	6477	No	9	4	Low-quality	19.74	AAI-based (high-confidence)
NODE_348_length_8953_cov_0.234019	8953	No	15	8	Low-quality	19.92	AAI-based (high-confidence)
NODE_3483_length_1656_cov_0.353243	1656	No	4	1	Low-quality	5.13	AAI-based (high-confidence)
NODE_3483_length_3107_cov_0.125997	3107	No	8	1	Low-quality	7.34	AAI-based (high-confidence)
NODE_3487_length_3356_cov_0.344796	3356	No	3	2	Low-quality	5.27	AAI-based (high-confidence)
NODE_349_length_13243_cov_0.236077	13243	No	27	7	Low-quality	37.33	AAI-based (high-confidence)
NODE_3490_length_3354_cov_0.137942	3354	No	10	1	Low-quality	10.51	AAI-based (high-confidence)
NODE_3492_length_2004_cov_0.107612	2004	No	6	1	Low-quality	3.98	AAI-based (high-confidence)
NODE_35_length_4243_cov_0.175193	4243	No	7	4	Low-quality	8.36	AAI-based (high-confidence)
NODE_35_length_4792_cov_0.135521	4792	No	9	6	Low-quality	7.75	AAI-based (high-confidence)
NODE_35_length_4839_cov_0.101055	4839	No	7	2	Low-quality	14.22	AAI-based (high-confidence)
NODE_350_length_10633_cov_0.198025	10633	No	14	3	Low-quality	34.09	AAI-based (high-confidence)
NODE_3500_length_1535_cov_0.103760	1535	No	4	2	Low-quality	3.83	AAI-based (high-confidence)
NODE_3501_length_2809_cov_0.250554	2809	No	4	2	Low-quality	11.42	AAI-based (high-confidence)
NODE_3508_length_3213_cov_0.217405	3213	No	3	3	Low-quality	5.32	AAI-based (high-confidence)
NODE_3509_length_2805_cov_0.471914	2805	No	3	3	Low-quality	7.16	AAI-based (high-confidence)
NODE_351_length_2279_cov_0.072936	2279	No	1	1	Low-quality	3.83	AAI-based (high-confidence)
NODE_351_length_4354_cov_0.140541	4354	No	5	3	Low-quality	11.94	AAI-based (high-confidence)

NODE_3510_length_1650_cov_0.221792	1650	No	3	2	Low-quality	4.96	AAI-based (high-confidence)
NODE_3511_length_3913_cov_0.349764	3913	No	7	1	Low-quality	8.27	AAI-based (high-confidence)
NODE_3514_length_3092_cov_0.180421	3092	No	4	4	Low-quality	4.85	AAI-based (high-confidence)
NODE_3519_length_1306_cov_0.106048	1306	No	3	1	Low-quality	3.49	AAI-based (high-confidence)
NODE_352_length_4225_cov_0.178139	4225	No	6	2	Low-quality	12.98	AAI-based (high-confidence)
NODE_3522_length_1649_cov_0.049677	1649	No	2	1	Low-quality	3.92	AAI-based (high-confidence)
NODE_3523_length_3329_cov_0.399381	3329	No	6	2	Low-quality	6.08	AAI-based (high-confidence)
NODE_3525_length_3203_cov_0.132410	3203	No	3	3	Low-quality	5.28	AAI-based (high-confidence)
NODE_353_length_2395_cov_0.111498	2395	No	5	2	Low-quality	4.68	AAI-based (high-confidence)
NODE_353_length_4354_cov_0.109988	4354	No	3	2	Low-quality	11.55	AAI-based (high-confidence)
NODE_3531_length_3200_cov_0.479845	3200	No	3	1	Low-quality	4.96	AAI-based (high-confidence)
NODE_3531_length_3325_cov_0.352139	3325	No	4	1	Low-quality	9.88	AAI-based (high-confidence)
NODE_3537_length_1305_cov_0.053068	1305	No	3	2	Low-quality	3.99	AAI-based (high-confidence)
NODE_3537_length_3078_cov_0.145686	3078	No	4	1	Low-quality	2.34	AAI-based (high-confidence)
NODE_354_length_2097_cov_0.077578	2097	No	3	1	Low-quality	4.16	AAI-based (high-confidence)
NODE_3547_length_2787_cov_0.187872	2787	No	4	1	Low-quality	2.8	AAI-based (high-confidence)
NODE_3552_length_3316_cov_0.441405	3316	No	3	3	Low-quality	10.11	AAI-based (high-confidence)
NODE_3555_length_2785_cov_0.256888	2785	No	6	3	Low-quality	6.15	AAI-based (high-confidence)
NODE_3569_length_1004_cov_0.072928	1004	No	2	1	Low-quality	2.34	AAI-based (high-confidence)
NODE_3573_length_1004_cov_0.046409	1004	No	1	1	Low-quality	3.22	AAI-based (high-confidence)
NODE_3573_length_1976_cov_0.291422	1976	No	2	1	Low-quality	5.52	AAI-based (high-confidence)
NODE_3573_length_3181_cov_0.167424	3181	No	4	2	Low-quality	9.9	AAI-based (high-confidence)
NODE_3575_length_3180_cov_0.135670	3180	No	4	1	Low-quality	5.95	AAI-based (high-confidence)
NODE_358_length_4344_cov_0.269729	4344	No	9	1	Low-quality	12.03	AAI-based (high-confidence)
NODE_3580_length_1975_cov_0.094883	1975	No	3	3	Low-quality	3.91	AAI-based (high-confidence)
NODE_3588_length_1634_cov_0.145928	1634	No	4	1	Low-quality	4.36	AAI-based (high-confidence)
NODE_3588_length_2774_cov_0.597383	2774	No	4	1	Low-quality	8.5	AAI-based (high-confidence)
NODE_3589_length_3297_cov_0.136648	3297	No	6	2	Low-quality	9.72	AAI-based (high-confidence)
NODE_359_length_2091_cov_0.104920	2091	No	3	1	Low-quality	6.54	AAI-based (high-confidence)
NODE_3598_length_3049_cov_0.143051	3049	No	6	1	Low-quality	9.14	AAI-based (high-confidence)
NODE_36_length_3736_cov_0.106406	3736	No	7	2	Low-quality	10.81	AAI-based (high-confidence)
NODE_36_length_4086_cov_0.097567	4086	No	2	1	Low-quality	13.5	AAI-based (high-confidence)
NODE_36_length_4270_cov_0.143131	4270	No	13	5	Low-quality	11.76	AAI-based (high-confidence)
NODE_360_length_6376_cov_0.434762	6376	No	12	2	Low-quality	13.08	AAI-based (high-confidence)
NODE_360_length_8766_cov_0.383062	8766	No	13	5	Low-quality	24.62	AAI-based (high-confidence)
NODE_3600_length_1632_cov_0.114808	1632	No	2	1	Low-quality	2.72	AAI-based (high-confidence)
NODE_3602_length_3166_cov_1.036192	3166	No	10	3	Low-quality	4.91	AAI-based (high-confidence)
NODE_3603_length_3166_cov_0.579394	3166	No	5	2	Low-quality	3.07	AAI-based (high-confidence)
NODE_361_length_12903_cov_0.289831	12903	No	16	14	Low-quality	7.18	AAI-based (high-confidence)
NODE_361_length_2087_cov_0.092555	2087	No	4	1	Low-quality	3.88	AAI-based (high-confidence)
NODE_361_length_6375_cov_0.139420	6375	No	8	3	Low-quality	6.16	AAI-based (high-confidence)
NODE_3610_length_3163_cov_0.141971	3163	No	4	2	Low-quality	2.13	AAI-based (high-confidence)
NODE_3616_length_1508_cov_0.095103	1508	No	3	1	Low-quality	3.35	AAI-based (high-confidence)
NODE_362_length_1809_cov_0.077193	1809	No	8	1	Low-quality	5.77	AAI-based (high-confidence)
NODE_3621_length_1507_cov_0.114347	1507	No	2	1	Low-quality	3.4	AAI-based (high-confidence)
NODE_3622_length_3279_cov_0.194969	3279	No	2	2	Low-quality	6.83	AAI-based (high-confidence)

NODE_363_length_2041_cov_0.124614	2041	No	6	1	Low-quality	3.74	AAI-based (high-confidence)
NODE_3631_length_1504_cov_0.106050	1504	No	4	1	Low-quality	4.74	AAI-based (high-confidence)
NODE_3634_length_3272_cov_0.390482	3272	No	3	1	Low-quality	9.92	AAI-based (high-confidence)
NODE_3635_length_1959_cov_0.104301	1959	No	4	2	Low-quality	5.11	AAI-based (high-confidence)
NODE_364_length_2080_cov_0.131247	2080	No	1	1	Low-quality	44.48	AAI-based (high-confidence)
NODE_3643_length_3270_cov_0.216020	3270	No	6	3	Low-quality	9.02	AAI-based (high-confidence)
NODE_365_length_2238_cov_0.384759	2238	No	3	1	Low-quality	5.98	AAI-based (high-confidence)
NODE_365_length_4328_cov_0.279499	4328	No	7	3	Low-quality	7.38	AAI-based (high-confidence)
NODE_3650_length_3266_cov_0.341332	3266	No	5	2	Low-quality	3.14	AAI-based (high-confidence)
NODE_3655_length_2752_cov_0.090841	2752	No	4	2	Low-quality	6.24	AAI-based (high-confidence)
NODE_366_length_8674_cov_0.444198	8674	No	9	6	Low-quality	21.18	AAI-based (high-confidence)
NODE_3666_length_1617_cov_0.153491	1617	No	3	2	Low-quality	4.78	AAI-based (high-confidence)
NODE_367_length_6341_cov_0.364467	6341	No	6	3	Low-quality	17.68	AAI-based (high-confidence)
NODE_3671_length_3010_cov_0.627619	3010	No	6	2	Low-quality	3.05	AAI-based (high-confidence)
NODE_3672_length_1949_cov_0.104324	1949	No	3	3	Low-quality	3.44	AAI-based (high-confidence)
NODE_368_length_4327_cov_0.153737	4327	No	7	3	Low-quality	7.94	AAI-based (high-confidence)
NODE_3683_length_1286_cov_0.045493	1286	No	1	1	Low-quality	3.55	AAI-based (high-confidence)
NODE_3683_length_1492_cov_0.073223	1492	No	3	1	Low-quality	3.4	AAI-based (high-confidence)
NODE_3684_length_1285_cov_0.247049	1285	No	1	1	Low-quality	23.6	AAI-based (high-confidence)
NODE_3691_length_1490_cov_0.077642	1490	No	4	2	Low-quality	4.32	AAI-based (high-confidence)
NODE_3694_length_1612_cov_0.134831	1612	No	5	3	Low-quality	4.09	AAI-based (high-confidence)
NODE_3698_length_1612_cov_0.087905	1612	No	3	2	Low-quality	4.38	AAI-based (high-confidence)
NODE_3699_length_2736_cov_0.103527	2736	No	3	2	Low-quality	5.18	AAI-based (high-confidence)
NODE_370_length_6321_cov_0.179042	6321	No	7	6	Low-quality	3.6	AAI-based (high-confidence)
NODE_3700_length_2735_cov_0.141882	2735	No	6	1	Low-quality	8.02	AAI-based (high-confidence)
NODE_3702_length_3125_cov_0.247852	3125	No	5	1	Low-quality	3.18	AAI-based (high-confidence)
NODE_371_length_12626_cov_0.361539	12626	No	12	4	Low-quality	29.1	AAI-based (high-confidence)
NODE_371_length_2346_cov_0.108589	2346	No	5	3	Low-quality	4.46	AAI-based (high-confidence)
NODE_3710_length_2731_cov_0.153116	2731	No	4	4	Low-quality	7.36	AAI-based (high-confidence)
NODE_3718_length_3229_cov_0.104792	3229	No	9	2	Low-quality	9.55	AAI-based (high-confidence)
NODE_372_length_1997_cov_0.083246	1997	No	2	1	Low-quality	5.71	AAI-based (high-confidence)
NODE_372_length_2059_cov_0.140816	2059	No	1	1	Low-quality	33.54	AAI-based (high-confidence)
NODE_372_length_9517_cov_0.240497	9517	No	19	5	Low-quality	26.83	AAI-based (high-confidence)
NODE_3721_length_2727_cov_0.131279	2727	No	6	2	Low-quality	5.32	AAI-based (high-confidence)
NODE_3724_length_2725_cov_0.226961	2725	No	4	1	Low-quality	4.65	AAI-based (high-confidence)
NODE_3728_length_1932_cov_0.319694	1932	No	3	2	Low-quality	3.11	AAI-based (high-confidence)
NODE_373_length_2014_cov_0.119582	2014	No	1	1	Low-quality	28.78	AAI-based (high-confidence)
NODE_3738_length_1929_cov_0.161202	1929	No	2	1	Low-quality	2.25	AAI-based (high-confidence)
NODE_3739_length_3110_cov_0.882099	3110	No	10	1	Low-quality	8.58	AAI-based (high-confidence)
NODE_374_length_8623_cov_0.188292	8623	No	9	6	Low-quality	16.04	AAI-based (high-confidence)
NODE_3748_length_1924_cov_0.198904	1924	No	4	1	Low-quality	5.19	AAI-based (high-confidence)
NODE_375_length_4289_cov_0.485680	4289	No	8	6	Low-quality	11.39	AAI-based (high-confidence)
NODE_3758_length_1600_cov_0.069953	1600	No	4	2	Low-quality	4.84	AAI-based (high-confidence)
NODE_376_length_2388_cov_0.130625	2388	No	2	1	Low-quality	3.83	AAI-based (high-confidence)
NODE_3760_length_3756_cov_0.124145	3756	No	6	1	Low-quality	6.96	AAI-based (high-confidence)
NODE_3763_length_1476_cov_0.120552	1476	No	5	1	Low-quality	4.66	AAI-based (high-confidence)

NODE_3767_length_1275_cov_0.257653	1275	No	3	1	Low-quality	2.48	AAI-based (high-confidence)
NODE_3773_length_1916_cov_0.174463	1916	No	5	1	Low-quality	4.17	AAI-based (high-confidence)
NODE_3783_length_2956_cov_0.242912	2956	No	6	1	Low-quality	8.64	AAI-based (high-confidence)
NODE_379_length_1984_cov_0.101857	1984	No	4	1	Low-quality	5.52	AAI-based (high-confidence)
NODE_379_length_4278_cov_0.230199	4278	No	2	2	Low-quality	7.36	AAI-based (high-confidence)
NODE_3790_length_2705_cov_0.131619	2705	No	2	1	Low-quality	8.34	AAI-based (high-confidence)
NODE_3794_length_1912_cov_0.146718	1912	No	3	1	Low-quality	5.8	AAI-based (high-confidence)
NODE_38_length_4744_cov_0.128525	4744	No	11	6	Low-quality	7.67	AAI-based (high-confidence)
NODE_3802_length_2702_cov_0.113331	2702	No	6	2	Low-quality	7.3	AAI-based (high-confidence)
NODE_3807_length_1592_cov_0.080375	1592	No	3	1	Low-quality	3.46	AAI-based (high-confidence)
NODE_3813_length_1590_cov_0.155600	1590	No	6	1	Low-quality	4.99	AAI-based (high-confidence)
NODE_3818_length_1467_cov_0.142544	1467	No	3	2	Low-quality	4.08	AAI-based (high-confidence)
NODE_3818_length_3078_cov_0.109433	3078	No	3	1	Low-quality	5.67	AAI-based (high-confidence)
NODE_382_length_6257_cov_0.171809	6257	No	8	6	Low-quality	3.49	AAI-based (high-confidence)
NODE_3821_length_1906_cov_0.175429	1906	No	3	1	Low-quality	5.94	AAI-based (high-confidence)
NODE_3830_length_1268_cov_0.063302	1268	No	3	1	Low-quality	3.44	AAI-based (high-confidence)
NODE_3833_length_3174_cov_0.173984	3174	No	2	1	Low-quality	4.95	AAI-based (high-confidence)
NODE_3842_length_3171_cov_0.207031	3171	No	5	2	Low-quality	8.63	AAI-based (high-confidence)
NODE_3846_length_3065_cov_0.096089	3065	No	3	3	Low-quality	5.88	AAI-based (high-confidence)
NODE_3858_length_3058_cov_0.693140	3058	No	6	3	Low-quality	7.66	AAI-based (high-confidence)
NODE_386_length_2194_cov_0.083532	2194	No	4	1	Low-quality	3.83	AAI-based (high-confidence)
NODE_386_length_9401_cov_0.583853	9401	No	12	4	Low-quality	28.8	AAI-based (high-confidence)
NODE_3863_length_1458_cov_0.181751	1458	No	2	2	Low-quality	2.39	AAI-based (high-confidence)
NODE_3864_length_1458_cov_0.181015	1458	No	3	2	Low-quality	2.45	AAI-based (high-confidence)
NODE_3868_length_3056_cov_0.119716	3056	No	3	1	Low-quality	5.73	AAI-based (high-confidence)
NODE_3873_length_1581_cov_0.085695	1581	No	2	1	Low-quality	3.4	AAI-based (high-confidence)
NODE_3874_length_1456_cov_0.214444	1456	No	1	1	Low-quality	2.41	AAI-based (high-confidence)
NODE_3877_length_1895_cov_0.228842	1895	No	4	1	Low-quality	4.45	AAI-based (high-confidence)
NODE_3893_length_3145_cov_0.384439	3145	No	5	1	Low-quality	6.25	AAI-based (high-confidence)
NODE_3898_length_3045_cov_0.668024	3045	No	8	2	Low-quality	7.2	AAI-based (high-confidence)
NODE_390_length_4237_cov_0.166747	4237	No	4	2	Low-quality	7.89	AAI-based (high-confidence)
NODE_3903_length_1258_cov_0.081104	1258	No	4	3	Low-quality	2.13	AAI-based (high-confidence)
NODE_391_length_12207_cov_0.440948	12207	No	16	7	Low-quality	34.28	AAI-based (high-confidence)
NODE_3912_length_2666_cov_0.401247	2666	No	4	1	Low-quality	7.7	AAI-based (high-confidence)
NODE_3926_length_3131_cov_0.393470	3131	No	5	2	Low-quality	2.97	AAI-based (high-confidence)
NODE_3927_length_1571_cov_0.236413	1571	No	1	1	Low-quality	2.58	AAI-based (high-confidence)
NODE_3927_length_2662_cov_0.481077	2662	No	5	2	Low-quality	6.17	AAI-based (high-confidence)
NODE_3930_length_1448_cov_0.122313	1448	No	4	1	Low-quality	3.04	AAI-based (high-confidence)
NODE_3933_length_1254_cov_0.099567	1254	No	3	1	Low-quality	3.8	AAI-based (high-confidence)
NODE_3937_length_2658_cov_0.086362	2658	No	3	2	Low-quality	7.32	AAI-based (high-confidence)
NODE_394_length_6189_cov_0.260755	6189	No	7	4	Low-quality	11.48	AAI-based (high-confidence)
NODE_3942_length_2656_cov_0.176770	2656	No	5	2	Low-quality	8.12	AAI-based (high-confidence)
NODE_3943_length_1569_cov_0.075510	1569	No	4	1	Low-quality	4.25	AAI-based (high-confidence)
NODE_395_length_2025_cov_0.117861	2025	No	1	1	Low-quality	30.87	AAI-based (high-confidence)
NODE_3954_length_1875_cov_0.118243	1875	No	3	2	Low-quality	5.15	AAI-based (high-confidence)
NODE_3959_length_2881_cov_0.152768	2881	No	3	2	Low-quality	5.65	AAI-based (high-confidence)

NODE_396_length_12137_cov_0.261838	12137	No	16	3	Low-quality	12.33	AAI-based (high-confidence)
NODE_3960_length_1874_cov_0.110423	1874	No	1	1	Low-quality	4.61	AAI-based (high-confidence)
NODE_3961_length_3018_cov_0.350120	3018	No	3	2	Low-quality	9.31	AAI-based (high-confidence)
NODE_3969_length_3114_cov_0.186070	3114	No	6	1	Low-quality	6.52	AAI-based (high-confidence)
NODE_397_length_9334_cov_0.498322	9334	No	18	3	Low-quality	27.17	AAI-based (high-confidence)
NODE_3972_length_1564_cov_0.105802	1564	No	2	1	Low-quality	3.92	AAI-based (high-confidence)
NODE_3974_length_1869_cov_1.128814	1869	No	2	1	Low-quality	5.04	AAI-based (high-confidence)
NODE_3977_length_1869_cov_0.167232	1869	No	2	1	Low-quality	4.66	AAI-based (high-confidence)
NODE_3980_length_3011_cov_0.393887	3011	No	8	1	Low-quality	4.87	AAI-based (high-confidence)
NODE_3981_length_3623_cov_0.089103	3623	No	6	3	Low-quality	5.27	AAI-based (high-confidence)
NODE_3994_length_3008_cov_0.200413	3008	No	6	3	Low-quality	6.69	AAI-based (high-confidence)
NODE_3999_length_2635_cov_0.207019	2635	No	6	2	Low-quality	4.16	AAI-based (high-confidence)
NODE_4_length_13381_cov_0.253877	13381	No	19	8	Low-quality	37.46	AAI-based (high-confidence)
NODE_4_length_21122_cov_0.726918	21122	No	29	8	Low-quality	34.62	AAI-based (high-confidence)
NODE_4_length_4652_cov_0.148913	4652	No	8	5	Low-quality	12.12	AAI-based (high-confidence)
NODE_4_length_9271_cov_0.543938	9271	No	18	3	Low-quality	15.24	AAI-based (high-confidence)
NODE_400_length_1938_cov_0.033714	1938	No	1	1	Low-quality	34.18	AAI-based (high-confidence)
NODE_400_length_4195_cov_0.101074	4195	No	4	4	Low-quality	2.35	AAI-based (high-confidence)
NODE_400_length_9296_cov_0.486246	9296	No	12	7	Low-quality	22.09	AAI-based (high-confidence)
NODE_4001_length_1860_cov_0.151051	1860	No	4	4	Low-quality	3.12	AAI-based (high-confidence)
NODE_4002_length_3099_cov_0.299000	3099	No	4	2	Low-quality	9.62	AAI-based (high-confidence)
NODE_4006_length_3003_cov_0.197658	3003	No	5	1	Low-quality	3.63	AAI-based (high-confidence)
NODE_4009_length_1857_cov_0.128555	1857	No	4	1	Low-quality	4.61	AAI-based (high-confidence)
NODE_4011_length_3096_cov_0.163830	3096	No	3	3	Low-quality	5.11	AAI-based (high-confidence)
NODE_4020_length_1555_cov_0.141484	1555	No	4	1	Low-quality	3.47	AAI-based (high-confidence)
NODE_4032_length_1553_cov_0.157497	1553	No	3	1	Low-quality	4.13	AAI-based (high-confidence)
NODE_4033_length_3595_cov_0.723970	3595	No	12	2	Low-quality	10.9	AAI-based (high-confidence)
NODE_4034_length_2627_cov_0.206487	2627	No	3	3	Low-quality	6.18	AAI-based (high-confidence)
NODE_4041_length_2990_cov_0.759599	2990	No	8	1	Low-quality	4.83	AAI-based (high-confidence)
NODE_405_length_2162_cov_0.387785	2162	No	1	1	Low-quality	30.25	AAI-based (high-confidence)
NODE_4056_length_2621_cov_0.130056	2621	No	3	2	Low-quality	4.86	AAI-based (high-confidence)
NODE_4067_length_1547_cov_0.044199	1547	No	1	1	Low-quality	4.01	AAI-based (high-confidence)
NODE_4080_length_2614_cov_0.160636	2614	No	3	3	Low-quality	4.27	AAI-based (high-confidence)
NODE_4083_length_1544_cov_0.107958	1544	No	1	1	Low-quality	3.55	AAI-based (high-confidence)
NODE_4084_length_3060_cov_0.371158	3060	No	4	2	Low-quality	2.83	AAI-based (high-confidence)
NODE_4090_length_1833_cov_0.444060	1833	No	3	1	Low-quality	5.81	AAI-based (high-confidence)
NODE_4092_length_1420_cov_0.177139	1420	No	5	1	Low-quality	2.29	AAI-based (high-confidence)
NODE_4094_length_1543_cov_0.052632	1543	No	4	1	Low-quality	4.21	AAI-based (high-confidence)
NODE_41_length_10419_cov_0.656202	10419	No	15	10	Low-quality	5.85	AAI-based (high-confidence)
NODE_41_length_4417_cov_0.138258	4417	No	9	3	Low-quality	8.68	AAI-based (high-confidence)
NODE_410_length_2003_cov_0.158088	2003	No	1	1	Low-quality	31.43	AAI-based (high-confidence)
NODE_4103_length_2970_cov_0.135493	2970	No	3	1	Low-quality	9.91	AAI-based (high-confidence)
NODE_4104_length_1418_cov_0.264594	1418	No	2	1	Low-quality	2.3	AAI-based (high-confidence)
NODE_4107_length_2608_cov_0.176963	2608	No	10	1	Low-quality	4.23	AAI-based (high-confidence)
NODE_4109_length_3046_cov_0.332881	3046	No	8	1	Low-quality	4.93	AAI-based (high-confidence)
NODE_411_length_2002_cov_0.358382	2002	No	5	2	Low-quality	4.06	AAI-based (high-confidence)

NODE_411_length_4129_cov_0.129280	4129	No	6	4	Low-quality	1.95	AAI-based (high-confidence)
NODE_4110_length_1827_cov_0.134838	1827	No	5	3	Low-quality	5.47	AAI-based (high-confidence)
NODE_4111_length_1540_cov_0.102012	1540	No	1	1	Low-quality	25.74	AAI-based (high-confidence)
NODE_4111_length_2607_cov_0.186204	2607	No	7	2	Low-quality	4.14	AAI-based (high-confidence)
NODE_4111_length_2967_cov_0.428870	2967	No	8	1	Low-quality	8.9	AAI-based (high-confidence)
NODE_4113_length_1826_cov_0.160973	1826	No	4	2	Low-quality	4.03	AAI-based (high-confidence)
NODE_412_length_11841_cov_0.294498	11841	No	21	2	Low-quality	18.58	AAI-based (high-confidence)
NODE_412_length_4127_cov_0.132572	4127	No	5	2	Low-quality	6.49	AAI-based (high-confidence)
NODE_4121_length_2961_cov_1.121943	2961	No	4	1	Low-quality	8.23	AAI-based (high-confidence)
NODE_4124_length_3042_cov_0.098879	3042	No	8	1	Low-quality	6.74	AAI-based (high-confidence)
NODE_4125_length_1824_cov_0.086377	1824	No	4	1	Low-quality	5.72	AAI-based (high-confidence)
NODE_4128_length_1821_cov_0.191638	1821	No	3	1	Low-quality	5.56	AAI-based (high-confidence)
NODE_4129_length_1821_cov_0.098722	1821	No	2	2	Low-quality	4.64	AAI-based (high-confidence)
NODE_413_length_1914_cov_0.304132	1914	No	1	1	Low-quality	29.01	AAI-based (high-confidence)
NODE_4136_length_1818_cov_0.237347	1818	No	1	1	Low-quality	3	AAI-based (high-confidence)
NODE_4146_length_1817_cov_0.056461	1817	No	1	1	Low-quality	3.15	AAI-based (high-confidence)
NODE_4151_length_1411_cov_0.407774	1411	No	4	1	Low-quality	2.81	AAI-based (high-confidence)
NODE_4154_length_1814_cov_0.491545	1814	No	3	1	Low-quality	3.75	AAI-based (high-confidence)
NODE_4159_length_2793_cov_0.349295	2793	No	3	3	Low-quality	4.61	AAI-based (high-confidence)
NODE_416_length_4031_cov_0.190997	4031	No	6	2	Low-quality	11.95	AAI-based (high-confidence)
NODE_4169_length_1530_cov_0.175402	1530	No	3	1	Low-quality	4.68	AAI-based (high-confidence)
NODE_4176_length_2584_cov_0.084105	2584	No	3	1	Low-quality	10.12	AAI-based (high-confidence)
NODE_4179_length_1809_cov_0.375439	1809	No	1	1	Low-quality	4.94	AAI-based (high-confidence)
NODE_418_length_6049_cov_0.369916	6049	No	13	4	Low-quality	9.93	AAI-based (high-confidence)
NODE_4180_length_1809_cov_0.282456	1809	No	4	2	Low-quality	5.01	AAI-based (high-confidence)
NODE_4180_length_2581_cov_0.196213	2581	No	3	1	Low-quality	4.27	AAI-based (high-confidence)
NODE_4186_length_1527_cov_0.191877	1527	No	4	2	Low-quality	2.54	AAI-based (high-confidence)
NODE_419_length_1906_cov_0.097952	1906	No	4	1	Low-quality	5.76	AAI-based (high-confidence)
NODE_419_length_1991_cov_0.075581	1991	No	2	2	Low-quality	4.93	AAI-based (high-confidence)
NODE_419_length_4102_cov_0.120909	4102	No	6	2	Low-quality	12.25	AAI-based (high-confidence)
NODE_4195_length_1807_cov_0.138173	1807	No	3	1	Low-quality	5.79	AAI-based (high-confidence)
NODE_4196_length_1807_cov_0.134660	1807	No	2	2	Low-quality	2.98	AAI-based (high-confidence)
NODE_4197_length_3010_cov_0.159395	3010	No	3	3	Low-quality	5.43	AAI-based (high-confidence)
NODE_42_length_4402_cov_0.237509	4402	No	6	1	Low-quality	12.26	AAI-based (high-confidence)
NODE_4202_length_3005_cov_0.432897	3005	No	7	3	Low-quality	4.91	AAI-based (high-confidence)
NODE_4203_length_2777_cov_0.229276	2777	No	2	2	Low-quality	4.58	AAI-based (high-confidence)
NODE_4205_length_1403_cov_0.131902	1403	No	1	1	Low-quality	4.28	AAI-based (high-confidence)
NODE_4208_length_1804_cov_0.237537	1804	No	4	1	Low-quality	3.23	AAI-based (high-confidence)
NODE_421_length_1916_cov_0.092460	1916	No	3	2	Low-quality	3.77	AAI-based (high-confidence)
NODE_421_length_9093_cov_0.292195	9093	No	14	9	Low-quality	4.29	AAI-based (high-confidence)
NODE_4211_length_2571_cov_0.181230	2571	No	2	2	Low-quality	4.25	AAI-based (high-confidence)
NODE_4218_length_2925_cov_0.117127	2925	No	5	2	Low-quality	4.83	AAI-based (high-confidence)
NODE_423_length_1751_cov_0.140436	1751	No	4	2	Low-quality	3.67	AAI-based (high-confidence)
NODE_423_length_2281_cov_0.100367	2281	No	1	1	Low-quality	36.49	AAI-based (high-confidence)
NODE_4233_length_2565_cov_0.196675	2565	No	4	1	Low-quality	4.39	AAI-based (high-confidence)
NODE_4247_length_3478_cov_0.125777	3478	No	4	4	Low-quality	5.8	AAI-based (high-confidence)

NODE_4257_length_1792_cov_0.108092	1792	No	1	1	Low-quality	4.41	AAI-based (high-confidence)
NODE_426_length_2247_cov_0.195065	2247	No	1	1	Low-quality	36.14	AAI-based (high-confidence)
NODE_426_length_9066_cov_0.180216	9066	No	13	4	Low-quality	17.91	AAI-based (high-confidence)
NODE_4267_length_2755_cov_0.450678	2755	No	3	1	Low-quality	4.78	AAI-based (high-confidence)
NODE_4267_length_2978_cov_0.146231	2978	No	7	4	Low-quality	4.21	AAI-based (high-confidence)
NODE_4268_length_2977_cov_0.520153	2977	No	5	2	Low-quality	6.85	AAI-based (high-confidence)
NODE_4269_length_1788_cov_0.069864	1788	No	5	1	Low-quality	5.7	AAI-based (high-confidence)
NODE_427_length_9564_cov_0.230534	9564	No	20	5	Low-quality	30.1	AAI-based (high-confidence)
NODE_4270_length_2552_cov_0.165919	2552	No	4	3	Low-quality	2.3	AAI-based (high-confidence)
NODE_4284_length_2970_cov_0.585162	2970	No	4	2	Low-quality	4.9	AAI-based (high-confidence)
NODE_4291_length_2548_cov_0.280523	2548	No	8	2	Low-quality	4.04	AAI-based (high-confidence)
NODE_4293_length_1781_cov_0.056480	1781	No	1	1	Low-quality	4.03	AAI-based (high-confidence)
NODE_4299_length_2898_cov_0.589496	2898	No	4	3	Low-quality	4.77	AAI-based (high-confidence)
NODE_43_length_3933_cov_0.158059	3933	No	2	1	Low-quality	40.24	AAI-based (high-confidence)
NODE_4305_length_2896_cov_0.400429	2896	No	4	1	Low-quality	5.4	AAI-based (high-confidence)
NODE_4307_length_2962_cov_0.615788	2962	No	5	3	Low-quality	4.79	AAI-based (high-confidence)
NODE_4308_length_2542_cov_0.150225	2542	No	7	1	Low-quality	4.63	AAI-based (high-confidence)
NODE_431_length_2476_cov_0.089188	2476	No	4	3	Low-quality	3.99	AAI-based (high-confidence)
NODE_431_length_3969_cov_0.219897	3969	No	8	1	Low-quality	11.56	AAI-based (high-confidence)
NODE_4315_length_2540_cov_0.284719	2540	No	7	2	Low-quality	4.21	AAI-based (high-confidence)
NODE_4319_length_2540_cov_0.079066	2540	No	2	1	Low-quality	6.75	AAI-based (high-confidence)
NODE_432_length_1740_cov_0.085923	1740	No	5	1	Low-quality	5.08	AAI-based (high-confidence)
NODE_432_length_9005_cov_0.454637	9005	No	13	1	Low-quality	14.49	AAI-based (high-confidence)
NODE_4324_length_1382_cov_0.219018	1382	No	2	1	Low-quality	3.2	AAI-based (high-confidence)
NODE_433_length_11509_cov_0.218755	11509	No	9	7	Low-quality	18.84	AAI-based (high-confidence)
NODE_4331_length_1773_cov_0.158303	1773	No	2	2	Low-quality	3.1	AAI-based (high-confidence)
NODE_4335_length_2727_cov_0.168189	2727	No	7	1	Low-quality	5.71	AAI-based (high-confidence)
NODE_4335_length_2888_cov_0.226605	2888	No	4	3	Low-quality	5.33	AAI-based (high-confidence)
NODE_4337_length_2946_cov_0.276431	2946	No	6	1	Low-quality	5.32	AAI-based (high-confidence)
NODE_434_length_11493_cov_0.204932	11493	No	13	8	Low-quality	31.69	AAI-based (high-confidence)
NODE_434_length_5945_cov_0.103319	5945	No	7	2	Low-quality	11.06	AAI-based (high-confidence)
NODE_4345_length_3421_cov_0.209512	3421	No	4	3	Low-quality	6.89	AAI-based (high-confidence)
NODE_4349_length_1769_cov_0.187425	1769	No	2	1	Low-quality	2.72	AAI-based (high-confidence)
NODE_435_length_4037_cov_0.606653	4037	No	6	3	Low-quality	2.27	AAI-based (high-confidence)
NODE_4354_length_2883_cov_0.169540	2883	No	7	3	Low-quality	8	AAI-based (high-confidence)
NODE_4359_length_2938_cov_0.395210	2938	No	10	1	Low-quality	4.77	AAI-based (high-confidence)
NODE_4367_length_1765_cov_0.081633	1765	No	2	2	Low-quality	4.02	AAI-based (high-confidence)
NODE_4368_length_2526_cov_0.422332	2526	No	4	1	Low-quality	7.4	AAI-based (high-confidence)
NODE_437_length_11401_cov_0.532207	11401	No	16	7	Low-quality	6.39	AAI-based (high-confidence)
NODE_4375_length_2715_cov_0.198777	2715	No	3	1	Low-quality	4.2	AAI-based (high-confidence)
NODE_4378_length_2931_cov_0.352048	2931	No	2	2	Low-quality	4.89	AAI-based (high-confidence)
NODE_4390_length_1371_cov_0.183176	1371	No	2	1	Low-quality	4.27	AAI-based (high-confidence)
NODE_4391_length_1496_cov_0.112384	1496	No	3	1	Low-quality	4.69	AAI-based (high-confidence)
NODE_4397_length_1758_cov_0.041591	1758	No	5	2	Low-quality	5.31	AAI-based (high-confidence)
NODE_440_length_11329_cov_0.224221	11329	No	17	9	Low-quality	5.34	AAI-based (high-confidence)
NODE_4401_length_2516_cov_0.111295	2516	No	5	3	Low-quality	7.93	AAI-based (high-confidence)

NODE_4403_length_2864_cov_0.262206	2864	No	7	4	Low-quality	6.34	AAI-based (high-confidence)
NODE_4405_length_2864_cov_0.171429	2864	No	4	2	Low-quality	11.27	AAI-based (high-confidence)
NODE_4408_length_2708_cov_0.128402	2708	No	6	2	Low-quality	7.4	AAI-based (high-confidence)
NODE_4415_length_2860_cov_0.268019	2860	No	2	2	Low-quality	6.89	AAI-based (high-confidence)
NODE_4420_length_1751_cov_0.137409	1751	No	3	1	Low-quality	4.22	AAI-based (high-confidence)
NODE_4425_length_2510_cov_0.214434	2510	No	5	2	Low-quality	4.05	AAI-based (high-confidence)
NODE_4429_length_1489_cov_0.100719	1489	No	2	2	Low-quality	4.08	AAI-based (high-confidence)
NODE_4430_length_2698_cov_0.131589	2698	No	4	1	Low-quality	2.42	AAI-based (high-confidence)
NODE_4432_length_2506_cov_0.368093	2506	No	3	1	Low-quality	7.77	AAI-based (high-confidence)
NODE_4434_length_1202_cov_0.059837	1202	No	2	1	Low-quality	2.34	AAI-based (high-confidence)
NODE_444_length_8904_cov_0.145940	8904	No	11	5	Low-quality	2.44	AAI-based (high-confidence)
NODE_4442_length_1748_cov_0.180109	1748	No	5	1	Low-quality	2.9	AAI-based (high-confidence)
NODE_4451_length_1485_cov_0.153680	1485	No	5	1	Low-quality	2.41	AAI-based (high-confidence)
NODE_4453_length_1485_cov_0.124820	1485	No	5	1	Low-quality	3.27	AAI-based (high-confidence)
NODE_4455_length_2844_cov_0.574863	2844	No	4	2	Low-quality	7.01	AAI-based (high-confidence)
NODE_4457_length_2692_cov_0.160818	2692	No	3	1	Low-quality	5	AAI-based (high-confidence)
NODE_446_length_5889_cov_0.335406	5889	No	14	3	Low-quality	18.76	AAI-based (high-confidence)
NODE_4460_length_2903_cov_0.200071	2903	No	5	1	Low-quality	5	AAI-based (high-confidence)
NODE_4461_length_2903_cov_0.107703	2903	No	4	1	Low-quality	6.08	AAI-based (high-confidence)
NODE_4463_length_2841_cov_0.158643	2841	No	3	1	Low-quality	5.98	AAI-based (high-confidence)
NODE_447_length_11189_cov_0.438233	11189	No	11	3	Low-quality	7.58	AAI-based (high-confidence)
NODE_447_length_2224_cov_0.099294	2224	No	1	1	Low-quality	35.01	AAI-based (high-confidence)
NODE_4481_length_2896_cov_0.148373	2896	No	4	1	Low-quality	7.38	AAI-based (high-confidence)
NODE_4485_length_2491_cov_0.211120	2491	No	7	2	Low-quality	7.17	AAI-based (high-confidence)
NODE_449_length_7880_cov_0.175684	7880	No	5	4	Low-quality	3.67	AAI-based (high-confidence)
NODE_45_length_2542_cov_0.090463	2542	No	3	1	Low-quality	7.07	AAI-based (high-confidence)
NODE_45_length_4512_cov_0.130523	4512	No	6	3	Low-quality	7.16	AAI-based (high-confidence)
NODE_450_length_4002_cov_0.258263	4002	No	7	4	Low-quality	10.14	AAI-based (high-confidence)
NODE_450_length_5870_cov_0.123895	5870	No	6	3	Low-quality	3.55	AAI-based (high-confidence)
NODE_4505_length_1478_cov_0.093546	1478	No	1	1	Low-quality	2.3	AAI-based (high-confidence)
NODE_4505_length_2887_cov_0.124462	2887	No	7	1	Low-quality	9.1	AAI-based (high-confidence)
NODE_4507_length_2483_cov_0.193792	2483	No	2	1	Low-quality	4.29	AAI-based (high-confidence)
NODE_451_length_1895_cov_0.097996	1895	No	2	1	Low-quality	27.61	AAI-based (high-confidence)
NODE_4514_length_1729_cov_0.158896	1729	No	6	1	Low-quality	2.8	AAI-based (high-confidence)
NODE_4517_length_2884_cov_0.219749	2884	No	6	2	Low-quality	5.16	AAI-based (high-confidence)
NODE_452_length_8836_cov_0.371523	8836	No	7	2	Low-quality	13.7	AAI-based (high-confidence)
NODE_4523_length_1727_cov_0.101351	1727	No	3	2	Low-quality	6.65	AAI-based (high-confidence)
NODE_4524_length_1727_cov_0.070025	1727	No	4	1	Low-quality	5.38	AAI-based (high-confidence)
NODE_4531_length_2878_cov_0.267722	2878	No	3	1	Low-quality	8.89	AAI-based (high-confidence)
NODE_4532_length_1724_cov_0.102769	1724	No	3	3	Low-quality	2.7	AAI-based (high-confidence)
NODE_4532_length_2877_cov_0.103672	2877	No	5	1	Low-quality	9.5	AAI-based (high-confidence)
NODE_454_length_2223_cov_0.159605	2223	No	2	2	Low-quality	4.26	AAI-based (high-confidence)
NODE_4546_length_1722_cov_0.071473	1722	No	3	3	Low-quality	4.59	AAI-based (high-confidence)
NODE_4549_length_3308_cov_0.102836	3308	No	5	2	Low-quality	7.57	AAI-based (high-confidence)
NODE_4550_length_1345_cov_0.320225	1345	No	2	2	Low-quality	2.44	AAI-based (high-confidence)
NODE_4551_length_1345_cov_0.296950	1345	No	3	1	Low-quality	2.17	AAI-based (high-confidence)

NODE_456_length_3986_cov_0.321842	3986	No	9	2	Low-quality	12.7	AAI-based (high-confidence)
NODE_456_length_5846_cov_0.125283	5846	No	7	3	Low-quality	13.35	AAI-based (high-confidence)
NODE_457_length_3976_cov_0.462471	3976	No	11	3	Low-quality	11.94	AAI-based (high-confidence)
NODE_4577_length_1712_cov_0.145691	1712	No	6	2	Low-quality	2.85	AAI-based (high-confidence)
NODE_4581_length_2859_cov_0.814855	2859	No	6	1	Low-quality	8.28	AAI-based (high-confidence)
NODE_4582_length_2858_cov_0.569047	2858	No	5	1	Low-quality	9.32	AAI-based (high-confidence)
NODE_4589_length_1710_cov_0.164494	1710	No	6	1	Low-quality	2.78	AAI-based (high-confidence)
NODE_459_length_1487_cov_0.085735	1487	No	3	3	Low-quality	2.56	AAI-based (high-confidence)
NODE_459_length_1843_cov_0.127294	1843	No	1	1	Low-quality	36.99	AAI-based (high-confidence)
NODE_459_length_8803_cov_0.458525	8803	No	20	4	Low-quality	27.9	AAI-based (high-confidence)
NODE_4590_length_1466_cov_0.100951	1466	No	2	1	Low-quality	2.57	AAI-based (high-confidence)
NODE_4591_length_1710_cov_0.106766	1710	No	5	1	Low-quality	5.42	AAI-based (high-confidence)
NODE_4597_length_2456_cov_0.448876	2456	No	5	2	Low-quality	5.06	AAI-based (high-confidence)
NODE_4598_length_1708_cov_0.123058	1708	No	3	1	Low-quality	5.29	AAI-based (high-confidence)
NODE_4598_length_3285_cov_0.150345	3285	No	4	2	Low-quality	6.08	AAI-based (high-confidence)
NODE_4611_length_1705_cov_0.247821	1705	No	2	1	Low-quality	3.28	AAI-based (high-confidence)
NODE_4612_length_1462_cov_0.592076	1462	No	2	1	Low-quality	4.54	AAI-based (high-confidence)
NODE_4613_length_1705_cov_0.174969	1705	No	4	1	Low-quality	3.73	AAI-based (high-confidence)
NODE_462_length_1918_cov_0.098406	1918	No	3	1	Low-quality	4.16	AAI-based (high-confidence)
NODE_462_length_2202_cov_0.143604	2202	No	1	1	Low-quality	32.92	AAI-based (high-confidence)
NODE_4621_length_2786_cov_0.457387	2786	No	7	3	Low-quality	4.43	AAI-based (high-confidence)
NODE_4622_length_2786_cov_0.365836	2786	No	6	1	Low-quality	8.45	AAI-based (high-confidence)
NODE_4629_length_1460_cov_0.149155	1460	No	6	1	Low-quality	2.98	AAI-based (high-confidence)
NODE_463_length_3957_cov_0.089943	3957	No	6	3	Low-quality	9.41	AAI-based (high-confidence)
NODE_4639_length_1459_cov_0.081618	1459	No	1	1	Low-quality	2.22	AAI-based (high-confidence)
NODE_464_length_2200_cov_0.284626	2200	No	1	1	Low-quality	32.12	AAI-based (high-confidence)
NODE_464_length_2201_cov_0.103711	2201	No	1	1	Low-quality	34.99	AAI-based (high-confidence)
NODE_464_length_7757_cov_0.229825	7757	No	16	10	Low-quality	4.59	AAI-based (high-confidence)
NODE_4641_length_2779_cov_0.221642	2779	No	5	3	Low-quality	8.46	AAI-based (high-confidence)
NODE_4644_length_1332_cov_0.103812	1332	No	2	1	Low-quality	4.19	AAI-based (high-confidence)
NODE_4649_length_1457_cov_0.167158	1457	No	5	1	Low-quality	2.36	AAI-based (high-confidence)
NODE_466_length_1481_cov_0.114327	1481	No	2	2	Low-quality	5.89	AAI-based (high-confidence)
NODE_466_length_2431_cov_0.107204	2431	No	1	1	Low-quality	33.84	AAI-based (high-confidence)
NODE_466_length_5787_cov_0.126758	5787	No	8	7	Low-quality	15.05	AAI-based (high-confidence)
NODE_466_length_7743_cov_0.182496	7743	No	11	1	Low-quality	7.69	AAI-based (high-confidence)
NODE_4660_length_3256_cov_0.132087	3256	No	2	1	Low-quality	7.5	AAI-based (high-confidence)
NODE_4664_length_2442_cov_0.076398	2442	No	4	2	Low-quality	7.35	AAI-based (high-confidence)
NODE_4665_length_2441_cov_0.186593	2441	No	3	1	Low-quality	4.61	AAI-based (high-confidence)
NODE_4669_length_2773_cov_0.219147	2773	No	5	2	Low-quality	6.17	AAI-based (high-confidence)
NODE_467_length_1910_cov_0.134180	1910	No	1	1	Low-quality	40.02	AAI-based (high-confidence)
NODE_467_length_5784_cov_0.105013	5784	No	9	3	Low-quality	10.72	AAI-based (high-confidence)
NODE_467_length_7724_cov_0.159869	7724	No	10	7	Low-quality	22.23	AAI-based (high-confidence)
NODE_4678_length_2768_cov_0.642188	2768	No	3	1	Low-quality	8.77	AAI-based (high-confidence)
NODE_4685_length_2436_cov_0.100128	2436	No	4	3	Low-quality	6.29	AAI-based (high-confidence)
NODE_4697_length_2432_cov_0.100300	2432	No	4	3	Low-quality	2.16	AAI-based (high-confidence)
NODE_4698_length_1175_cov_0.133829	1175	No	3	1	Low-quality	2.35	AAI-based (high-confidence)

NODE_470_length_10841_cov_0.384658	10841	No	2	2	Low-quality	3.77	AAI-based (high-confidence)
NODE_470_length_1702_cov_0.077979	1702	No	3	1	Low-quality	5.16	AAI-based (high-confidence)
NODE_4704_length_2760_cov_0.246148	2760	No	5	3	Low-quality	3.41	AAI-based (high-confidence)
NODE_4708_length_3236_cov_0.118903	3236	No	4	4	Low-quality	5.78	AAI-based (high-confidence)
NODE_4714_length_2425_cov_0.234738	2425	No	6	2	Low-quality	5.26	AAI-based (high-confidence)
NODE_4715_length_2814_cov_0.114549	2814	No	4	2	Low-quality	4.35	AAI-based (high-confidence)
NODE_4724_length_2754_cov_0.116761	2754	No	2	2	Low-quality	4.55	AAI-based (high-confidence)
NODE_4725_length_2754_cov_0.105838	2754	No	5	3	Low-quality	3.48	AAI-based (high-confidence)
NODE_4725_length_3225_cov_1.802943	3225	No	9	3	Low-quality	9.44	AAI-based (high-confidence)
NODE_4729_length_2806_cov_0.404876	2806	No	6	1	Low-quality	4.56	AAI-based (high-confidence)
NODE_4733_length_2420_cov_0.134856	2420	No	6	2	Low-quality	2.96	AAI-based (high-confidence)
NODE_474_length_3924_cov_0.204183	3924	No	9	2	Low-quality	6.43	AAI-based (high-confidence)
NODE_4741_length_2747_cov_0.233761	2747	No	2	2	Low-quality	6.2	AAI-based (high-confidence)
NODE_4749_length_2746_cov_0.162826	2746	No	3	2	Low-quality	6.47	AAI-based (high-confidence)
NODE_4758_length_2745_cov_0.086546	2745	No	3	1	Low-quality	4.33	AAI-based (high-confidence)
NODE_476_length_2037_cov_0.094943	2037	No	3	3	Low-quality	5.15	AAI-based (high-confidence)
NODE_4765_length_1673_cov_0.210928	1673	No	4	1	Low-quality	2.96	AAI-based (high-confidence)
NODE_477_length_2036_cov_0.166753	2036	No	5	2	Low-quality	6.32	AAI-based (high-confidence)
NODE_4770_length_2410_cov_0.236694	2410	No	5	2	Low-quality	7.32	AAI-based (high-confidence)
NODE_4775_length_2740_cov_0.244983	2740	No	3	2	Low-quality	6.08	AAI-based (high-confidence)
NODE_478_length_7617_cov_0.320431	7617	No	13	5	Low-quality	12.31	AAI-based (high-confidence)
NODE_4785_length_2735_cov_0.105842	2735	No	7	3	Low-quality	8.86	AAI-based (high-confidence)
NODE_4792_length_2733_cov_0.153379	2733	No	5	2	Low-quality	4.67	AAI-based (high-confidence)
NODE_48_length_3791_cov_0.113489	3791	No	6	2	Low-quality	8.74	AAI-based (high-confidence)
NODE_48_length_3970_cov_0.115216	3970	No	8	3	Low-quality	7.17	AAI-based (high-confidence)
NODE_481_length_1808_cov_0.064365	1808	No	6	2	Low-quality	6.2	AAI-based (high-confidence)
NODE_4819_length_1165_cov_0.089118	1165	No	3	1	Low-quality	3.41	AAI-based (high-confidence)
NODE_4819_length_1661_cov_0.540333	1661	No	3	2	Low-quality	5.1	AAI-based (high-confidence)
NODE_4819_length_2774_cov_0.332336	2774	No	9	1	Low-quality	6.12	AAI-based (high-confidence)
NODE_4825_length_2772_cov_0.200150	2772	No	5	4	Low-quality	5.67	AAI-based (high-confidence)
NODE_483_length_5644_cov_0.113616	5644	No	5	1	Low-quality	12.51	AAI-based (high-confidence)
NODE_483_length_7591_cov_0.153097	7591	No	12	6	Low-quality	3.63	AAI-based (high-confidence)
NODE_4837_length_1658_cov_0.097498	1658	No	1	1	Low-quality	5.23	AAI-based (high-confidence)
NODE_4838_length_1657_cov_0.309371	1657	No	6	1	Low-quality	4.21	AAI-based (high-confidence)
NODE_4838_length_2766_cov_0.447319	2766	No	4	3	Low-quality	2.53	AAI-based (high-confidence)
NODE_4844_length_2391_cov_0.234293	2391	No	7	1	Low-quality	9.53	AAI-based (high-confidence)
NODE_4845_length_2763_cov_0.139640	2763	No	4	2	Low-quality	9.22	AAI-based (high-confidence)
NODE_4846_length_1301_cov_0.201331	1301	No	2	1	Low-quality	2.67	AAI-based (high-confidence)
NODE_485_length_5632_cov_0.928791	5632	No	16	2	Low-quality	11.79	AAI-based (high-confidence)
NODE_4852_length_1431_cov_0.082583	1431	No	1	1	Low-quality	2.66	AAI-based (high-confidence)
NODE_4854_length_2712_cov_0.203215	2712	No	2	1	Low-quality	8.2	AAI-based (high-confidence)
NODE_4859_length_1654_cov_0.152412	1654	No	2	2	Low-quality	2.55	AAI-based (high-confidence)
NODE_486_length_1468_cov_0.086925	1468	No	2	2	Low-quality	2.6	AAI-based (high-confidence)
NODE_486_length_2159_cov_0.127184	2159	No	3	1	Low-quality	7.38	AAI-based (high-confidence)
NODE_4860_length_1654_cov_0.118328	1654	No	3	1	Low-quality	3.71	AAI-based (high-confidence)
NODE_4860_length_2711_cov_0.109495	2711	No	4	2	Low-quality	8.45	AAI-based (high-confidence)

NODE_4861_length_2385_cov_0.089676	2385	No	4	4	Low-quality	4.02	AAI-based (high-confidence)
NODE_4861_length_2710_cov_0.442359	2710	No	3	3	Low-quality	5.03	AAI-based (high-confidence)
NODE_4865_length_2710_cov_0.124856	2710	No	4	2	Low-quality	7.45	AAI-based (high-confidence)
NODE_487_length_3786_cov_0.122864	3786	No	1	1	Low-quality	5.23	AAI-based (high-confidence)
NODE_4887_length_1647_cov_0.142119	1647	No	3	1	Low-quality	3.27	AAI-based (high-confidence)
NODE_4888_length_2706_cov_0.095129	2706	No	5	1	Low-quality	7.77	AAI-based (high-confidence)
NODE_4889_length_2706_cov_0.065209	2706	No	5	2	Low-quality	6.59	AAI-based (high-confidence)
NODE_489_length_1801_cov_0.063455	1801	No	2	1	Low-quality	4.43	AAI-based (high-confidence)
NODE_49_length_4042_cov_0.196551	4042	No	5	2	Low-quality	11.26	AAI-based (high-confidence)
NODE_490_length_10458_cov_0.364417	10458	No	13	11	Low-quality	5.78	AAI-based (high-confidence)
NODE_490_length_8904_cov_0.177853	8904	No	13	8	Low-quality	28.04	AAI-based (high-confidence)
NODE_4901_length_1424_cov_0.224906	1424	No	5	1	Low-quality	4.44	AAI-based (high-confidence)
NODE_4902_length_2742_cov_0.185017	2742	No	3	2	Low-quality	5.77	AAI-based (high-confidence)
NODE_4903_length_3146_cov_0.179849	3146	No	4	3	Low-quality	6.34	AAI-based (high-confidence)
NODE_4906_length_2373_cov_0.112577	2373	No	4	3	Low-quality	5.83	AAI-based (high-confidence)
NODE_491_length_1800_cov_0.059965	1800	No	5	1	Low-quality	4	AAI-based (high-confidence)
NODE_4916_length_2696_cov_0.115518	2696	No	5	1	Low-quality	8.07	AAI-based (high-confidence)
NODE_492_length_2011_cov_0.096757	2011	No	4	2	Low-quality	5.07	AAI-based (high-confidence)
NODE_492_length_7515_cov_0.416262	7515	No	18	3	Low-quality	18.99	AAI-based (high-confidence)
NODE_492_length_8473_cov_0.366133	8473	No	17	11	Low-quality	4.98	AAI-based (high-confidence)
NODE_4925_length_1641_cov_0.081064	1641	No	3	2	Low-quality	2.94	AAI-based (high-confidence)
NODE_493_length_1796_cov_0.084856	1796	No	2	1	Low-quality	4.74	AAI-based (high-confidence)
NODE_4931_length_2692_cov_0.097956	2692	No	2	2	Low-quality	4.87	AAI-based (high-confidence)
NODE_4940_length_2361_cov_0.137047	2361	No	8	4	Low-quality	5.31	AAI-based (high-confidence)
NODE_495_length_1878_cov_0.136594	1878	No	2	1	Low-quality	28.7	AAI-based (high-confidence)
NODE_495_length_3762_cov_0.118482	3762	No	4	1	Low-quality	6.96	AAI-based (high-confidence)
NODE_495_length_3856_cov_0.124834	3856	No	6	4	Low-quality	9.4	AAI-based (high-confidence)
NODE_4959_length_2725_cov_0.091394	2725	No	2	2	Low-quality	4.58	AAI-based (high-confidence)
NODE_4960_length_2724_cov_0.497143	2724	No	5	2	Low-quality	8.16	AAI-based (high-confidence)
NODE_4965_length_1415_cov_0.076748	1415	No	2	1	Low-quality	2.33	AAI-based (high-confidence)
NODE_4967_length_2721_cov_0.919146	2721	No	4	2	Low-quality	6.82	AAI-based (high-confidence)
NODE_4969_length_1153_cov_0.047438	1153	No	2	2	Low-quality	3.2	AAI-based (high-confidence)
NODE_4969_length_2354_cov_0.189357	2354	No	3	1	Low-quality	5.69	AAI-based (high-confidence)
NODE_4977_length_2353_cov_0.413487	2353	No	2	2	Low-quality	4.07	AAI-based (high-confidence)
NODE_4979_length_1413_cov_0.280061	1413	No	3	2	Low-quality	2.31	AAI-based (high-confidence)
NODE_498_length_1812_cov_0.164040	1812	No	2	2	Low-quality	3.53	AAI-based (high-confidence)
NODE_498_length_2383_cov_0.100263	2383	No	2	2	Low-quality	4.87	AAI-based (high-confidence)
NODE_4986_length_2517_cov_0.215467	2517	No	6	3	Low-quality	7.53	AAI-based (high-confidence)
NODE_4987_length_2517_cov_0.129859	2517	No	3	2	Low-quality	8.03	AAI-based (high-confidence)
NODE_499_length_1783_cov_0.166865	1783	No	3	1	Low-quality	3.4	AAI-based (high-confidence)
NODE_4990_length_1279_cov_0.144915	1279	No	1	1	Low-quality	4	AAI-based (high-confidence)
NODE_4994_length_1625_cov_0.202490	1625	No	3	2	Low-quality	5.16	AAI-based (high-confidence)
NODE_4997_length_2347_cov_0.438612	2347	No	2	1	Low-quality	4.37	AAI-based (high-confidence)
NODE_5_length_10209_cov_0.496637	10209	No	22	2	Low-quality	16.81	AAI-based (high-confidence)
NODE_5_length_10988_cov_0.485169	10988	No	22	2	Low-quality	18.04	AAI-based (high-confidence)
NODE_5_length_13830_cov_0.549924	13830	No	29	3	Low-quality	22.69	AAI-based (high-confidence)

NODE_5_length_3892_cov_0.118376	3892	No	6	3	Low-quality	12.69	AAI-based (high-confidence)
NODE_5_length_9013_cov_0.609042	9013	No	17	3	Low-quality	14.83	AAI-based (high-confidence)
NODE_50_length_28475_cov_0.459896	28475	No	26	10	Low-quality	47.47	AAI-based (high-confidence)
NODE_50_length_3762_cov_0.067704	3762	No	12	5	Low-quality	6.49	AAI-based (high-confidence)
NODE_50_length_3797_cov_0.091130	3797	No	7	3	Low-quality	6.14	AAI-based (high-confidence)
NODE_50_length_4025_cov_0.135507	4025	No	10	2	Low-quality	12.3	AAI-based (high-confidence)
NODE_50_length_9694_cov_0.270974	9694	No	13	7	Low-quality	27.24	AAI-based (high-confidence)
NODE_500_length_3838_cov_0.216635	3838	No	3	2	Low-quality	5.91	AAI-based (high-confidence)
NODE_5000_length_1411_cov_0.086128	1411	No	3	2	Low-quality	2.57	AAI-based (high-confidence)
NODE_5004_length_1624_cov_0.128525	1624	No	4	4	Low-quality	4.46	AAI-based (high-confidence)
NODE_5007_length_1150_cov_0.070409	1150	No	1	1	Low-quality	2.84	AAI-based (high-confidence)
NODE_5009_length_1410_cov_0.097635	1410	No	5	1	Low-quality	3.65	AAI-based (high-confidence)
NODE_5009_length_2705_cov_0.173830	2705	No	3	3	Low-quality	6.17	AAI-based (high-confidence)
NODE_501_length_2379_cov_0.096491	2379	No	4	1	Low-quality	6.85	AAI-based (high-confidence)
NODE_501_length_3833_cov_0.129888	3833	No	4	4	Low-quality	9.07	AAI-based (high-confidence)
NODE_5019_length_2342_cov_0.218903	2342	No	4	1	Low-quality	7.36	AAI-based (high-confidence)
NODE_502_length_5554_cov_0.120440	5554	No	14	1	Low-quality	17.4	AAI-based (high-confidence)
NODE_5020_length_1621_cov_0.069645	1621	No	2	2	Low-quality	2.84	AAI-based (high-confidence)
NODE_5022_length_2662_cov_1.005853	2662	No	3	3	Low-quality	4.38	AAI-based (high-confidence)
NODE_5024_length_2341_cov_0.236842	2341	No	3	3	Low-quality	7.1	AAI-based (high-confidence)
NODE_5037_length_1274_cov_0.134468	1274	No	2	1	Low-quality	2.9	AAI-based (high-confidence)
NODE_504_length_1802_cov_0.110393	1802	No	1	1	Low-quality	2.99	AAI-based (high-confidence)
NODE_5048_length_2657_cov_0.132525	2657	No	2	1	Low-quality	2.37	AAI-based (high-confidence)
NODE_5064_length_2495_cov_0.142321	2495	No	6	1	Low-quality	6.87	AAI-based (high-confidence)
NODE_5067_length_2651_cov_0.096787	2651	No	1	1	Low-quality	8.08	AAI-based (high-confidence)
NODE_5069_length_2495_cov_0.101002	2495	No	5	4	Low-quality	6.02	AAI-based (high-confidence)
NODE_507_length_1775_cov_0.105012	1775	No	1	1	Low-quality	28.25	AAI-based (high-confidence)
NODE_507_length_2121_cov_0.105836	2121	No	4	1	Low-quality	6.49	AAI-based (high-confidence)
NODE_507_length_5521_cov_0.248432	5521	No	8	3	Low-quality	17.41	AAI-based (high-confidence)
NODE_507_length_8347_cov_0.393307	8347	No	9	3	Low-quality	5.05	AAI-based (high-confidence)
NODE_5079_length_2688_cov_0.096562	2688	No	4	3	Low-quality	10.58	AAI-based (high-confidence)
NODE_5080_length_2326_cov_0.161652	2326	No	4	2	Low-quality	6.29	AAI-based (high-confidence)
NODE_5086_length_2325_cov_0.094789	2325	No	6	1	Low-quality	6.55	AAI-based (high-confidence)
NODE_5087_length_1608_cov_0.133863	1608	No	2	1	Low-quality	4.52	AAI-based (high-confidence)
NODE_509_length_8326_cov_0.233499	8326	No	10	1	Low-quality	10.29	AAI-based (high-confidence)
NODE_5090_length_1269_cov_0.058120	1269	No	2	2	Low-quality	3.26	AAI-based (high-confidence)
NODE_5095_length_1401_cov_0.072197	1401	No	2	1	Low-quality	3.84	AAI-based (high-confidence)
NODE_511_length_3723_cov_0.258278	3723	No	4	2	Low-quality	6.4	AAI-based (high-confidence)
NODE_5111_length_2679_cov_0.341473	2679	No	8	2	Low-quality	4.44	AAI-based (high-confidence)
NODE_5112_length_2679_cov_0.075969	2679	No	2	2	Low-quality	6.5	AAI-based (high-confidence)
NODE_5115_length_2639_cov_0.262205	2639	No	7	2	Low-quality	7.42	AAI-based (high-confidence)
NODE_5120_length_2676_cov_0.123787	2676	No	4	3	Low-quality	8.33	AAI-based (high-confidence)
NODE_5130_length_2311_cov_0.150090	2311	No	3	2	Low-quality	6.79	AAI-based (high-confidence)
NODE_5138_length_1395_cov_0.048611	1395	No	2	1	Low-quality	4.46	AAI-based (high-confidence)
NODE_5144_length_1598_cov_0.294863	1598	No	5	1	Low-quality	2.82	AAI-based (high-confidence)
NODE_5155_length_2667_cov_0.182632	2667	No	3	2	Low-quality	4.6	AAI-based (high-confidence)

NODE_5156_length_1597_cov_0.141522	1597	No	4	2	Low-quality	2.66	AAI-based (high-confidence)
NODE_5157_length_1391_cov_0.116099	1391	No	2	1	Low-quality	2.67	AAI-based (high-confidence)
NODE_516_length_8260_cov_0.706654	8260	No	12	3	Low-quality	9.24	AAI-based (high-confidence)
NODE_517_length_1861_cov_0.065834	1861	No	1	1	Low-quality	40.09	AAI-based (high-confidence)
NODE_517_length_3701_cov_0.159634	3701	No	11	1	Low-quality	5.77	AAI-based (high-confidence)
NODE_5174_length_2663_cov_0.118955	2663	No	7	1	Low-quality	8.32	AAI-based (high-confidence)
NODE_5178_length_2661_cov_0.225605	2661	No	4	2	Low-quality	6.92	AAI-based (high-confidence)
NODE_518_length_3779_cov_0.393207	3779	No	5	3	Low-quality	11.57	AAI-based (high-confidence)
NODE_519_length_1855_cov_0.283599	1855	No	1	1	Low-quality	37.38	AAI-based (high-confidence)
NODE_519_length_7294_cov_0.359416	7294	No	8	4	Low-quality	1.99	AAI-based (high-confidence)
NODE_52_length_3242_cov_0.122176	3242	No	4	2	Low-quality	9.92	AAI-based (high-confidence)
NODE_52_length_4358_cov_0.134539	4358	No	6	2	Low-quality	13.58	AAI-based (high-confidence)
NODE_52_length_9555_cov_0.193211	9555	No	19	7	Low-quality	31.58	AAI-based (high-confidence)
NODE_5206_length_2295_cov_0.105647	2295	No	2	2	Low-quality	4.27	AAI-based (high-confidence)
NODE_5206_length_2651_cov_0.397335	2651	No	3	2	Low-quality	3.45	AAI-based (high-confidence)
NODE_521_length_3763_cov_0.229803	3763	No	5	3	Low-quality	10.53	AAI-based (high-confidence)
NODE_5217_length_1587_cov_0.075269	1587	No	2	2	Low-quality	2.64	AAI-based (high-confidence)
NODE_5220_length_2454_cov_0.221656	2454	No	3	3	Low-quality	4.04	AAI-based (high-confidence)
NODE_523_length_2355_cov_0.088652	2355	No	6	2	Low-quality	3.78	AAI-based (high-confidence)
NODE_5231_length_2287_cov_0.133455	2287	No	3	3	Low-quality	3.55	AAI-based (high-confidence)
NODE_5234_length_1382_cov_0.081060	1382	No	1	1	Low-quality	3.77	AAI-based (high-confidence)
NODE_5234_length_2608_cov_0.156238	2608	No	2	1	Low-quality	3.44	AAI-based (high-confidence)
NODE_524_length_7237_cov_0.409779	7237	No	11	6	Low-quality	3.99	AAI-based (high-confidence)
NODE_5252_length_2603_cov_0.146166	2603	No	4	3	Low-quality	8.08	AAI-based (high-confidence)
NODE_5255_length_1244_cov_0.157205	1244	No	1	1	Low-quality	3.37	AAI-based (high-confidence)
NODE_5256_length_2603_cov_0.088259	2603	No	5	1	Low-quality	7	AAI-based (high-confidence)
NODE_5259_length_2637_cov_0.109929	2637	No	4	2	Low-quality	7.92	AAI-based (high-confidence)
NODE_526_length_1643_cov_0.227332	1643	No	2	1	Low-quality	4.98	AAI-based (high-confidence)
NODE_526_length_5418_cov_0.151344	5418	No	12	4	Low-quality	8.76	AAI-based (high-confidence)
NODE_526_length_8187_cov_0.453882	8187	No	18	4	Low-quality	25.64	AAI-based (high-confidence)
NODE_5261_length_2635_cov_0.940457	2635	No	2	1	Low-quality	7.63	AAI-based (high-confidence)
NODE_5267_length_2634_cov_0.762130	2634	No	5	2	Low-quality	8.38	AAI-based (high-confidence)
NODE_5278_length_2277_cov_0.183655	2277	No	2	1	Low-quality	3.68	AAI-based (high-confidence)
NODE_5283_length_2433_cov_0.120823	2433	No	3	1	Low-quality	3.27	AAI-based (high-confidence)
NODE_5289_length_2628_cov_0.143140	2628	No	3	2	Low-quality	3.66	AAI-based (high-confidence)
NODE_529_length_1642_cov_0.100454	1642	No	3	2	Low-quality	5.23	AAI-based (high-confidence)
NODE_5293_length_1574_cov_0.446102	1574	No	6	1	Low-quality	3.27	AAI-based (high-confidence)
NODE_5297_length_2625_cov_0.452890	2625	No	5	1	Low-quality	8.01	AAI-based (high-confidence)
NODE_5300_length_2586_cov_0.390028	2586	No	5	1	Low-quality	1.94	AAI-based (high-confidence)
NODE_5304_length_1238_cov_0.138718	1238	No	3	1	Low-quality	3.3	AAI-based (high-confidence)
NODE_5308_length_2271_cov_0.606814	2271	No	2	1	Low-quality	4.53	AAI-based (high-confidence)
NODE_531_length_7187_cov_0.190886	7187	No	12	6	Low-quality	3.74	AAI-based (high-confidence)
NODE_5319_length_2580_cov_0.605401	2580	No	3	3	Low-quality	4.27	AAI-based (high-confidence)
NODE_5321_length_1570_cov_0.091094	1570	No	1	1	Low-quality	4.21	AAI-based (high-confidence)
NODE_5324_length_2618_cov_0.371576	2618	No	5	2	Low-quality	7.28	AAI-based (high-confidence)
NODE_5326_length_1371_cov_0.091195	1371	No	4	1	Low-quality	4.34	AAI-based (high-confidence)

NODE_5331_length_1568_cov_0.074200	1568	No	4	2	Low-quality	27.67	AAI-based (high-confidence)
NODE_534_length_1637_cov_0.118986	1637	No	3	1	Low-quality	4.49	AAI-based (high-confidence)
NODE_5343_length_2613_cov_0.165473	2613	No	4	4	Low-quality	8.03	AAI-based (high-confidence)
NODE_5345_length_1368_cov_0.338849	1368	No	2	1	Low-quality	3.9	AAI-based (high-confidence)
NODE_5345_length_2574_cov_0.228283	2574	No	5	1	Low-quality	4.6	AAI-based (high-confidence)
NODE_5358_length_2607_cov_0.112440	2607	No	6	2	Low-quality	5.64	AAI-based (high-confidence)
NODE_5365_length_1560_cov_0.077344	1560	No	1	1	Low-quality	25.61	AAI-based (high-confidence)
NODE_5366_length_1559_cov_3.234247	1559	No	3	2	Low-quality	3.68	AAI-based (high-confidence)
NODE_5367_length_1365_cov_0.208531	1365	No	1	1	Low-quality	2.25	AAI-based (high-confidence)
NODE_5375_length_2405_cov_0.228101	2405	No	3	2	Low-quality	6.72	AAI-based (high-confidence)
NODE_5389_length_2600_cov_0.175130	2600	No	6	2	Low-quality	4.48	AAI-based (high-confidence)
NODE_539_length_1739_cov_0.153659	1739	No	5	3	Low-quality	4.52	AAI-based (high-confidence)
NODE_5392_length_1554_cov_0.134708	1554	No	4	3	Low-quality	3.95	AAI-based (high-confidence)
NODE_5392_length_2600_cov_0.120352	2600	No	5	2	Low-quality	7.64	AAI-based (high-confidence)
NODE_5392_length_2932_cov_2.021885	2932	No	9	2	Low-quality	4.58	AAI-based (high-confidence)
NODE_54_length_4143_cov_0.109792	4143	No	7	5	Low-quality	6.7	AAI-based (high-confidence)
NODE_540_length_1739_cov_0.136585	1739	No	1	1	Low-quality	23.98	AAI-based (high-confidence)
NODE_5402_length_2252_cov_0.148630	2252	No	7	4	Low-quality	4.04	AAI-based (high-confidence)
NODE_5413_length_2593_cov_0.504812	2593	No	4	2	Low-quality	7.84	AAI-based (high-confidence)
NODE_5414_length_2593_cov_0.378107	2593	No	2	2	Low-quality	4.29	AAI-based (high-confidence)
NODE_542_length_1735_cov_0.300733	1735	No	4	1	Low-quality	3.52	AAI-based (high-confidence)
NODE_5421_length_1547_cov_0.241713	1547	No	4	1	Low-quality	4.59	AAI-based (high-confidence)
NODE_5428_length_2588_cov_0.339092	2588	No	3	2	Low-quality	4.05	AAI-based (high-confidence)
NODE_543_length_1759_cov_0.077711	1759	No	1	1	Low-quality	4.38	AAI-based (high-confidence)
NODE_543_length_3696_cov_0.091743	3696	No	4	2	Low-quality	6.32	AAI-based (high-confidence)
NODE_544_length_1825_cov_0.075898	1825	No	7	2	Low-quality	2.85	AAI-based (high-confidence)
NODE_545_length_3695_cov_0.125973	3695	No	9	1	Low-quality	14.7	AAI-based (high-confidence)
NODE_5456_length_2546_cov_0.255006	2546	No	2	1	Low-quality	4.26	AAI-based (high-confidence)
NODE_5466_length_2543_cov_0.184534	2543	No	6	3	Low-quality	6.61	AAI-based (high-confidence)
NODE_547_length_2052_cov_0.051203	2052	No	4	4	Low-quality	5.78	AAI-based (high-confidence)
NODE_5476_length_1354_cov_0.109163	1354	No	1	1	Low-quality	4.13	AAI-based (high-confidence)
NODE_548_length_8004_cov_0.677925	8004	No	10	6	Low-quality	4.52	AAI-based (high-confidence)
NODE_549_length_1949_cov_0.117297	1949	No	5	2	Low-quality	3.73	AAI-based (high-confidence)
NODE_549_length_3683_cov_0.121931	3683	No	3	2	Low-quality	2.05	AAI-based (high-confidence)
NODE_55_length_3693_cov_0.102671	3693	No	7	5	Low-quality	5.98	AAI-based (high-confidence)
NODE_55_length_3928_cov_0.072343	3928	No	3	1	Low-quality	7.23	AAI-based (high-confidence)
NODE_5501_length_2231_cov_0.385553	2231	No	8	1	Low-quality	5.84	AAI-based (high-confidence)
NODE_5503_length_2564_cov_0.096146	2564	No	5	3	Low-quality	5.49	AAI-based (high-confidence)
NODE_5506_length_1349_cov_0.209600	1349	No	2	1	Low-quality	2.17	AAI-based (high-confidence)
NODE_5509_length_1349_cov_0.086400	1349	No	2	2	Low-quality	3.05	AAI-based (high-confidence)
NODE_551_length_5326_cov_0.299790	5326	No	12	2	Low-quality	15.64	AAI-based (high-confidence)
NODE_5517_length_2559_cov_0.485772	2559	No	3	2	Low-quality	5.08	AAI-based (high-confidence)
NODE_552_length_1819_cov_0.112791	1819	No	2	1	Low-quality	4.26	AAI-based (high-confidence)
NODE_552_length_2042_cov_0.107051	2042	No	3	1	Low-quality	3.51	AAI-based (high-confidence)
NODE_552_length_7988_cov_0.226011	7988	No	11	4	Low-quality	13.81	AAI-based (high-confidence)
NODE_5522_length_2558_cov_0.332656	2558	No	3	2	Low-quality	4.76	AAI-based (high-confidence)

NODE_553_length_1942_cov_0.248508	1942	No	3	2	Low-quality	3.79	AAI-based (high-confidence)
NODE_553_length_5312_cov_0.153462	5312	No	6	3	Low-quality	16.34	AAI-based (high-confidence)
NODE_5530_length_2226_cov_0.132581	2226	No	2	1	Low-quality	6.87	AAI-based (high-confidence)
NODE_5539_length_2526_cov_0.565719	2526	No	4	1	Low-quality	7.95	AAI-based (high-confidence)
NODE_554_length_1941_cov_0.163952	1941	No	5	1	Low-quality	3.45	AAI-based (high-confidence)
NODE_554_length_2040_cov_0.130860	2040	No	1	1	Low-quality	32.22	AAI-based (high-confidence)
NODE_5547_length_2550_cov_0.341085	2550	No	7	2	Low-quality	6.16	AAI-based (high-confidence)
NODE_555_length_5308_cov_0.167211	5308	No	10	5	Low-quality	11.63	AAI-based (high-confidence)
NODE_5553_length_1208_cov_0.079351	1208	No	1	1	Low-quality	3.75	AAI-based (high-confidence)
NODE_5556_length_2876_cov_0.821390	2876	No	6	1	Low-quality	8.91	AAI-based (high-confidence)
NODE_5557_length_2548_cov_0.190282	2548	No	4	2	Low-quality	4.58	AAI-based (high-confidence)
NODE_5558_length_2548_cov_0.175174	2548	No	4	2	Low-quality	10.35	AAI-based (high-confidence)
NODE_5559_length_1207_cov_0.080325	1207	No	3	1	Low-quality	3.3	AAI-based (high-confidence)
NODE_556_length_2037_cov_0.384933	2037	No	1	1	Low-quality	42.56	AAI-based (high-confidence)
NODE_5561_length_2547_cov_1.083333	2547	No	4	1	Low-quality	7.88	AAI-based (high-confidence)
NODE_5562_length_2522_cov_0.040858	2522	No	3	1	Low-quality	4.01	AAI-based (high-confidence)
NODE_5568_length_2363_cov_0.261042	2363	No	3	2	Low-quality	9.6	AAI-based (high-confidence)
NODE_557_length_1810_cov_0.078317	1810	No	5	4	Low-quality	5.24	AAI-based (high-confidence)
NODE_557_length_3648_cov_0.170471	3648	No	3	3	Low-quality	6.05	AAI-based (high-confidence)
NODE_557_length_7042_cov_0.203802	7042	No	7	3	Low-quality	3.92	AAI-based (high-confidence)
NODE_5575_length_2518_cov_0.336089	2518	No	3	2	Low-quality	7.67	AAI-based (high-confidence)
NODE_5576_length_2867_cov_0.138367	2867	No	6	2	Low-quality	6.12	AAI-based (high-confidence)
NODE_558_length_1716_cov_0.239332	1716	No	1	1	Low-quality	30.8	AAI-based (high-confidence)
NODE_559_length_5287_cov_0.133770	5287	No	11	3	Low-quality	8.55	AAI-based (high-confidence)
NODE_5590_length_2536_cov_0.613459	2536	No	5	1	Low-quality	5.04	AAI-based (high-confidence)
NODE_5591_length_1518_cov_0.140944	1518	No	4	1	Low-quality	1.98	AAI-based (high-confidence)
NODE_5591_length_2214_cov_0.147518	2214	No	6	1	Low-quality	4.01	AAI-based (high-confidence)
NODE_56_length_9283_cov_0.569360	9283	No	5	3	Low-quality	5.88	AAI-based (high-confidence)
NODE_560_length_2032_cov_0.069840	2032	No	5	1	Low-quality	5.66	AAI-based (high-confidence)
NODE_560_length_7871_cov_0.182321	7871	No	8	3	Low-quality	5.23	AAI-based (high-confidence)
NODE_560_length_8344_cov_0.230443	8344	No	16	5	Low-quality	13.51	AAI-based (high-confidence)
NODE_5603_length_1339_cov_0.262903	1339	No	3	2	Low-quality	2.4	AAI-based (high-confidence)
NODE_5605_length_1516_cov_0.222301	1516	No	2	1	Low-quality	3.08	AAI-based (high-confidence)
NODE_562_length_1808_cov_0.079579	1808	No	4	2	Low-quality	5.52	AAI-based (high-confidence)
NODE_562_length_1935_cov_0.114379	1935	No	3	2	Low-quality	5.54	AAI-based (high-confidence)
NODE_562_length_3635_cov_0.147907	3635	No	10	2	Low-quality	11.32	AAI-based (high-confidence)
NODE_562_length_9459_cov_0.563355	9459	No	7	6	Low-quality	5.17	AAI-based (high-confidence)
NODE_5620_length_1338_cov_0.112187	1338	No	1	1	Low-quality	3.62	AAI-based (high-confidence)
NODE_5622_length_2353_cov_0.127773	2353	No	3	2	Low-quality	4.3	AAI-based (high-confidence)
NODE_563_length_1420_cov_0.076457	1420	No	2	2	Low-quality	2.32	AAI-based (high-confidence)
NODE_5634_length_2207_cov_0.087287	2207	No	2	1	Low-quality	4.07	AAI-based (high-confidence)
NODE_5636_length_1511_cov_0.232295	1511	No	4	1	Low-quality	2.86	AAI-based (high-confidence)
NODE_5638_length_1511_cov_0.151558	1511	No	2	2	Low-quality	2.69	AAI-based (high-confidence)
NODE_564_length_1807_cov_0.089578	1807	No	3	1	Low-quality	5.95	AAI-based (high-confidence)
NODE_5645_length_2519_cov_0.119835	2519	No	7	2	Low-quality	6.89	AAI-based (high-confidence)
NODE_566_length_7836_cov_0.382836	7836	No	11	3	Low-quality	4.11	AAI-based (high-confidence)

NODE_567_length_6985_cov_0.141882	6985	No	13	1	Low-quality	8.56	AAI-based (high-confidence)
NODE_568_length_1930_cov_0.112507	1930	No	3	1	Low-quality	3.83	AAI-based (high-confidence)
NODE_5683_length_2197_cov_0.255481	2197	No	3	2	Low-quality	3.45	AAI-based (high-confidence)
NODE_5684_length_1331_cov_0.076299	1331	No	2	1	Low-quality	3.03	AAI-based (high-confidence)
NODE_5686_length_1331_cov_0.072240	1331	No	4	2	Low-quality	2.89	AAI-based (high-confidence)
NODE_5690_length_2494_cov_0.106054	2494	No	2	2	Low-quality	4.11	AAI-based (high-confidence)
NODE_5692_length_2493_cov_0.129490	2493	No	2	1	Low-quality	4	AAI-based (high-confidence)
NODE_57_length_3145_cov_0.110637	3145	No	9	1	Low-quality	8.49	AAI-based (high-confidence)
NODE_57_length_3903_cov_0.047319	3903	No	4	3	Low-quality	10.8	AAI-based (high-confidence)
NODE_5700_length_2337_cov_0.170688	2337	No	3	1	Low-quality	4.77	AAI-based (high-confidence)
NODE_571_length_1798_cov_0.150088	1798	No	2	2	Low-quality	3.49	AAI-based (high-confidence)
NODE_5717_length_2487_cov_0.134841	2487	No	3	1	Low-quality	2.21	AAI-based (high-confidence)
NODE_5717_length_2503_cov_0.232529	2503	No	6	1	Low-quality	4.63	AAI-based (high-confidence)
NODE_5728_length_1498_cov_0.854182	1498	No	3	1	Low-quality	4.63	AAI-based (high-confidence)
NODE_573_length_6945_cov_0.182734	6945	No	10	3	Low-quality	7.05	AAI-based (high-confidence)
NODE_5737_length_1497_cov_0.157368	1497	No	4	2	Low-quality	3.41	AAI-based (high-confidence)
NODE_574_length_1919_cov_0.126923	1919	No	5	1	Low-quality	5.61	AAI-based (high-confidence)
NODE_574_length_2282_cov_0.077416	2282	No	4	2	Low-quality	4.49	AAI-based (high-confidence)
NODE_5741_length_2493_cov_0.243943	2493	No	3	1	Low-quality	4.12	AAI-based (high-confidence)
NODE_575_length_1693_cov_0.087829	1693	No	4	2	Low-quality	4.29	AAI-based (high-confidence)
NODE_575_length_3554_cov_0.183213	3554	No	7	2	Low-quality	5.95	AAI-based (high-confidence)
NODE_575_length_6931_cov_0.276347	6931	No	10	8	Low-quality	3.89	AAI-based (high-confidence)
NODE_5753_length_2479_cov_0.131933	2479	No	5	2	Low-quality	6.84	AAI-based (high-confidence)
NODE_5757_length_1324_cov_0.062041	1324	No	3	2	Low-quality	4.2	AAI-based (high-confidence)
NODE_5759_length_1493_cov_0.536585	1493	No	3	2	Low-quality	4.63	AAI-based (high-confidence)
NODE_576_length_3607_cov_0.114025	3607	No	2	1	Low-quality	5.38	AAI-based (high-confidence)
NODE_5768_length_1322_cov_0.031071	1322	No	4	1	Low-quality	2.38	AAI-based (high-confidence)
NODE_577_length_1412_cov_0.057121	1412	No	2	1	Low-quality	3.66	AAI-based (high-confidence)
NODE_577_length_1916_cov_0.063841	1916	No	3	1	Low-quality	6.29	AAI-based (high-confidence)
NODE_5772_length_2474_cov_0.213053	2474	No	2	2	Low-quality	7.56	AAI-based (high-confidence)
NODE_5776_length_2474_cov_0.112842	2474	No	3	3	Low-quality	5.67	AAI-based (high-confidence)
NODE_5778_length_2473_cov_0.431340	2473	No	4	1	Low-quality	4.11	AAI-based (high-confidence)
NODE_579_length_1790_cov_0.144885	1790	No	5	2	Low-quality	3.57	AAI-based (high-confidence)
NODE_579_length_2008_cov_0.113148	2008	No	1	1	Low-quality	41.52	AAI-based (high-confidence)
NODE_579_length_3604_cov_0.142939	3604	No	7	1	Low-quality	6.71	AAI-based (high-confidence)
NODE_579_length_9281_cov_0.228599	9281	No	11	6	Low-quality	21.72	AAI-based (high-confidence)
NODE_5800_length_2481_cov_0.128463	2481	No	4	1	Low-quality	2.87	AAI-based (high-confidence)
NODE_5801_length_2466_cov_0.122095	2466	No	5	2	Low-quality	6.69	AAI-based (high-confidence)
NODE_5805_length_1486_cov_0.115357	1486	No	4	3	Low-quality	3.06	AAI-based (high-confidence)
NODE_5805_length_2465_cov_0.454776	2465	No	3	1	Low-quality	4.55	AAI-based (high-confidence)
NODE_5806_length_2479_cov_0.181933	2479	No	4	3	Low-quality	5.77	AAI-based (high-confidence)
NODE_581_length_2005_cov_0.112802	2005	No	4	2	Low-quality	4.41	AAI-based (high-confidence)
NODE_581_length_3602_cov_0.181559	3602	No	7	1	Low-quality	3.83	AAI-based (high-confidence)
NODE_582_length_1787_cov_0.088863	1787	No	4	1	Low-quality	3.12	AAI-based (high-confidence)
NODE_5821_length_1084_cov_0.095431	1084	No	3	2	Low-quality	2.08	AAI-based (high-confidence)
NODE_5826_length_2306_cov_0.193475	2306	No	6	1	Low-quality	4.36	AAI-based (high-confidence)

NODE_5829_length_1315_cov_0.111020	1315	No	2	1	Low-quality	2.09	AAI-based (high-confidence)
NODE_583_length_1726_cov_0.122926	1726	No	2	2	Low-quality	5.01	AAI-based (high-confidence)
NODE_5835_length_1481_cov_0.113603	1481	No	3	1	Low-quality	3.48	AAI-based (high-confidence)
NODE_584_length_1905_cov_0.076412	1905	No	3	1	Low-quality	3.28	AAI-based (high-confidence)
NODE_584_length_3600_cov_0.172808	3600	No	4	2	Low-quality	3.34	AAI-based (high-confidence)
NODE_584_length_6875_cov_0.162633	6875	No	7	1	Low-quality	19.38	AAI-based (high-confidence)
NODE_5841_length_1314_cov_0.214815	1314	No	5	1	Low-quality	2.06	AAI-based (high-confidence)
NODE_5841_length_2302_cov_0.164775	2302	No	3	2	Low-quality	4.24	AAI-based (high-confidence)
NODE_5847_length_2456_cov_0.193891	2456	No	2	1	Low-quality	4.56	AAI-based (high-confidence)
NODE_5849_length_1478_cov_0.577955	1478	No	5	1	Low-quality	4.53	AAI-based (high-confidence)
NODE_5851_length_1313_cov_0.126853	1313	No	2	1	Low-quality	4.23	AAI-based (high-confidence)
NODE_5857_length_2454_cov_0.154565	2454	No	5	2	Low-quality	7.72	AAI-based (high-confidence)
NODE_5865_length_2466_cov_0.458809	2466	No	7	2	Low-quality	7.81	AAI-based (high-confidence)
NODE_5866_length_2155_cov_0.061770	2155	No	3	1	Low-quality	6.08	AAI-based (high-confidence)
NODE_5875_length_2463_cov_0.378596	2463	No	2	1	Low-quality	4.58	AAI-based (high-confidence)
NODE_5878_length_1311_cov_0.084983	1311	No	3	1	Low-quality	4.06	AAI-based (high-confidence)
NODE_588_length_7701_cov_0.576559	7701	No	12	4	Low-quality	8.44	AAI-based (high-confidence)
NODE_5883_length_1473_cov_0.531295	1473	No	3	1	Low-quality	3.21	AAI-based (high-confidence)
NODE_5885_length_2291_cov_0.147354	2291	No	7	1	Low-quality	7.21	AAI-based (high-confidence)
NODE_5886_length_2150_cov_0.169673	2150	No	2	2	Low-quality	5.26	AAI-based (high-confidence)
NODE_5887_length_2449_cov_0.108936	2449	No	5	2	Low-quality	7.97	AAI-based (high-confidence)
NODE_5888_length_2150_cov_0.074110	2150	No	5	2	Low-quality	4	AAI-based (high-confidence)
NODE_589_length_5158_cov_0.125124	5158	No	5	4	Low-quality	10.51	AAI-based (high-confidence)
NODE_59_length_26258_cov_0.335793	26258	No	20	3	Low-quality	26.69	AAI-based (high-confidence)
NODE_59_length_3626_cov_0.121917	3626	No	8	4	Low-quality	8.69	AAI-based (high-confidence)
NODE_590_length_1772_cov_0.281530	1772	No	1	1	Low-quality	24.78	AAI-based (high-confidence)
NODE_590_length_2000_cov_0.112572	2000	No	2	1	Low-quality	6.25	AAI-based (high-confidence)
NODE_590_length_3577_cov_0.224267	3577	No	3	1	Low-quality	12.15	AAI-based (high-confidence)
NODE_5902_length_1168_cov_0.082320	1168	No	3	2	Low-quality	3.29	AAI-based (high-confidence)
NODE_5905_length_1078_cov_0.055158	1078	No	4	1	Low-quality	3.05	AAI-based (high-confidence)
NODE_5905_length_1168_cov_0.060804	1168	No	3	1	Low-quality	3.5	AAI-based (high-confidence)
NODE_5907_length_2455_cov_0.104414	2455	No	4	1	Low-quality	3.69	AAI-based (high-confidence)
NODE_5911_length_2146_cov_0.094284	2146	No	2	2	Low-quality	5.12	AAI-based (high-confidence)
NODE_5911_length_2443_cov_0.522611	2443	No	4	1	Low-quality	4.52	AAI-based (high-confidence)
NODE_5917_length_2285_cov_0.106130	2285	No	4	2	Low-quality	8.62	AAI-based (high-confidence)
NODE_5923_length_1467_cov_0.206140	1467	No	3	2	Low-quality	4.52	AAI-based (high-confidence)
NODE_5927_length_2144_cov_0.198044	2144	No	4	1	Low-quality	3.64	AAI-based (high-confidence)
NODE_5927_length_2450_cov_0.372607	2450	No	3	3	Low-quality	4.09	AAI-based (high-confidence)
NODE_593_length_11038_cov_0.312551	11038	No	20	7	Low-quality	20.18	AAI-based (high-confidence)
NODE_5939_length_2439_cov_0.070940	2439	No	5	4	Low-quality	7.5	AAI-based (high-confidence)
NODE_594_length_1770_cov_0.088570	1770	No	4	1	Low-quality	3.13	AAI-based (high-confidence)
NODE_595_length_8013_cov_0.301870	8013	No	11	2	Low-quality	23.27	AAI-based (high-confidence)
NODE_596_length_1990_cov_0.133792	1990	No	1	1	Low-quality	32.07	AAI-based (high-confidence)
NODE_5962_length_1463_cov_0.060117	1463	No	4	1	Low-quality	4.46	AAI-based (high-confidence)
NODE_597_length_3516_cov_0.127012	3516	No	6	1	Low-quality	10.07	AAI-based (high-confidence)
NODE_5971_length_2274_cov_0.091034	2274	No	2	1	Low-quality	6.06	AAI-based (high-confidence)

NODE_5981_length_2134_cov_0.272236	2134	No	5	1	Low-quality	6.01	AAI-based (high-confidence)
NODE_5982_length_2437_cov_0.251069	2437	No	3	2	Low-quality	4.4	AAI-based (high-confidence)
NODE_5985_length_2429_cov_0.265236	2429	No	2	1	Low-quality	4.18	AAI-based (high-confidence)
NODE_599_length_5114_cov_0.150548	5114	No	3	3	Low-quality	8.46	AAI-based (high-confidence)
NODE_5991_length_2132_cov_0.292671	2132	No	4	1	Low-quality	4.33	AAI-based (high-confidence)
NODE_6_length_8659_cov_0.236332	8659	No	11	4	Low-quality	24.05	AAI-based (high-confidence)
NODE_60_length_20410_cov_0.846586	20410	No	27	14	Low-quality	37.36	AAI-based (high-confidence)
NODE_60_length_3087_cov_0.136546	3087	No	5	2	Low-quality	9.55	AAI-based (high-confidence)
NODE_60_length_3827_cov_0.427575	3827	No	9	1	Low-quality	6.28	AAI-based (high-confidence)
NODE_60_length_3884_cov_0.131572	3884	No	10	2	Low-quality	7.29	AAI-based (high-confidence)
NODE_6006_length_2424_cov_0.992688	2424	No	5	1	Low-quality	6.29	AAI-based (high-confidence)
NODE_6007_length_1454_cov_0.071587	1454	No	2	1	Low-quality	3.37	AAI-based (high-confidence)
NODE_6009_length_2423_cov_0.620052	2423	No	4	1	Low-quality	7	AAI-based (high-confidence)
NODE_6010_length_1453_cov_0.662482	1453	No	2	2	Low-quality	4.48	AAI-based (high-confidence)
NODE_6016_length_1295_cov_0.108696	1295	No	3	1	Low-quality	2.1	AAI-based (high-confidence)
NODE_602_length_3550_cov_0.172414	3550	No	4	1	Low-quality	7.87	AAI-based (high-confidence)
NODE_602_length_7609_cov_0.790280	7609	No	12	3	Low-quality	6.76	AAI-based (high-confidence)
NODE_6026_length_2261_cov_0.110083	2261	No	5	2	Low-quality	6.3	AAI-based (high-confidence)
NODE_6027_length_1295_cov_0.056856	1295	No	3	3	Low-quality	2.86	AAI-based (high-confidence)
NODE_6034_length_2426_cov_0.116029	2426	No	5	1	Low-quality	35.95	AAI-based (high-confidence)
NODE_6037_length_1449_cov_0.340000	1449	No	4	1	Low-quality	2.87	AAI-based (high-confidence)
NODE_604_length_1663_cov_0.065857	1663	No	8	1	Low-quality	3.36	AAI-based (high-confidence)
NODE_604_length_9098_cov_0.180242	9098	No	12	5	Low-quality	11.14	AAI-based (high-confidence)
NODE_6043_length_2255_cov_0.131725	2255	No	5	2	Low-quality	7.11	AAI-based (high-confidence)
NODE_6045_length_2124_cov_0.108148	2124	No	4	1	Low-quality	6.78	AAI-based (high-confidence)
NODE_6046_length_2124_cov_0.090864	2124	No	5	1	Low-quality	6.61	AAI-based (high-confidence)
NODE_605_length_3540_cov_0.158384	3540	No	4	2	Low-quality	5.54	AAI-based (high-confidence)
NODE_6062_length_2420_cov_0.174925	2420	No	4	3	Low-quality	3.91	AAI-based (high-confidence)
NODE_6065_length_2120_cov_0.119743	2120	No	5	2	Low-quality	5.52	AAI-based (high-confidence)
NODE_6066_length_2251_cov_0.177509	2251	No	3	3	Low-quality	6.12	AAI-based (high-confidence)
NODE_608_length_1880_cov_0.125772	1880	No	1	1	Low-quality	39.6	AAI-based (high-confidence)
NODE_6082_length_2248_cov_0.111215	2248	No	2	1	Low-quality	2.01	AAI-based (high-confidence)
NODE_6088_length_2115_cov_0.161706	2115	No	4	1	Low-quality	3.91	AAI-based (high-confidence)
NODE_6088_length_2408_cov_0.162408	2408	No	2	1	Low-quality	4.1	AAI-based (high-confidence)
NODE_609_length_1712_cov_0.115313	1712	No	3	1	Low-quality	5.37	AAI-based (high-confidence)
NODE_609_length_5081_cov_0.214773	5081	No	12	6	Low-quality	9.18	AAI-based (high-confidence)
NODE_6097_length_1442_cov_0.131050	1442	No	5	1	Low-quality	4.47	AAI-based (high-confidence)
NODE_6099_length_2113_cov_0.157895	2113	No	3	2	Low-quality	3.41	AAI-based (high-confidence)
NODE_6099_length_2412_cov_0.195850	2412	No	3	1	Low-quality	4.4	AAI-based (high-confidence)
NODE_61_length_3875_cov_0.131356	3875	No	14	2	Low-quality	12.69	AAI-based (high-confidence)
NODE_610_length_1658_cov_0.059012	1658	No	4	1	Low-quality	4.66	AAI-based (high-confidence)
NODE_6106_length_2403_cov_0.129340	2403	No	2	2	Low-quality	4.47	AAI-based (high-confidence)
NODE_6108_length_2411_cov_0.189879	2411	No	4	2	Low-quality	2.28	AAI-based (high-confidence)
NODE_611_length_9023_cov_0.343008	9023	No	13	4	Low-quality	27.65	AAI-based (high-confidence)
NODE_6112_length_1287_cov_0.105219	1287	No	2	1	Low-quality	3.91	AAI-based (high-confidence)
NODE_6118_length_2408_cov_0.186228	2408	No	7	2	Low-quality	6.86	AAI-based (high-confidence)

NODE_6119_length_1144_cov_0.090909	1144	No	1	1	Low-quality	3	AAI-based (high-confidence)
NODE_6120_length_1064_cov_0.064249	1064	No	1	1	Low-quality	1.95	AAI-based (high-confidence)
NODE_6124_length_2240_cov_0.086408	2240	No	5	3	Low-quality	5.47	AAI-based (high-confidence)
NODE_6128_length_2109_cov_0.220896	2109	No	3	1	Low-quality	2.93	AAI-based (high-confidence)
NODE_613_length_7562_cov_0.382554	7562	No	13	7	Low-quality	4.42	AAI-based (high-confidence)
NODE_6133_length_1438_cov_0.100075	1438	No	4	1	Low-quality	4.47	AAI-based (high-confidence)
NODE_614_length_6725_cov_0.408542	6725	No	10	6	Low-quality	3.73	AAI-based (high-confidence)
NODE_6146_length_2402_cov_0.136778	2402	No	3	1	Low-quality	2.17	AAI-based (high-confidence)
NODE_6156_length_2399_cov_1.256087	2399	No	6	1	Low-quality	6.67	AAI-based (high-confidence)
NODE_6157_length_2399_cov_0.782609	2399	No	4	2	Low-quality	7.34	AAI-based (high-confidence)
NODE_6161_length_2399_cov_0.123913	2399	No	2	2	Low-quality	2.16	AAI-based (high-confidence)
NODE_6161_length_2672_cov_0.129421	2672	No	7	4	Low-quality	5.98	AAI-based (high-confidence)
NODE_6176_length_1432_cov_0.043511	1432	No	1	1	Low-quality	27.42	AAI-based (high-confidence)
NODE_6180_length_2100_cov_0.198901	2100	No	1	1	Low-quality	3.9	AAI-based (high-confidence)
NODE_619_length_3509_cov_0.129912	3509	No	11	1	Low-quality	2.66	AAI-based (high-confidence)
NODE_619_length_7513_cov_0.237658	7513	No	10	1	Low-quality	11.52	AAI-based (high-confidence)
NODE_6191_length_2385_cov_0.430446	2385	No	7	1	Low-quality	7.01	AAI-based (high-confidence)
NODE_62_length_18901_cov_0.200032	18901	No	32	10	Low-quality	32.14	AAI-based (high-confidence)
NODE_62_length_2377_cov_0.076822	2377	No	5	1	Low-quality	7.04	AAI-based (high-confidence)
NODE_6204_length_2390_cov_0.361851	2390	No	8	3	Low-quality	3.95	AAI-based (high-confidence)
NODE_6207_length_1277_cov_0.101868	1277	No	1	1	Low-quality	4.05	AAI-based (high-confidence)
NODE_6207_length_2390_cov_0.139241	2390	No	3	2	Low-quality	35.63	AAI-based (high-confidence)
NODE_621_length_1869_cov_0.081356	1869	No	2	2	Low-quality	25.93	AAI-based (high-confidence)
NODE_622_length_1698_cov_0.169481	1698	No	2	1	Low-quality	26.87	AAI-based (high-confidence)
NODE_6220_length_2378_cov_0.118912	2378	No	6	2	Low-quality	3.95	AAI-based (high-confidence)
NODE_6222_length_1276_cov_0.056924	1276	No	2	1	Low-quality	2.55	AAI-based (high-confidence)
NODE_623_length_1866_cov_0.128466	1866	No	3	2	Low-quality	5.44	AAI-based (high-confidence)
NODE_623_length_7745_cov_0.185718	7745	No	14	4	Low-quality	4.59	AAI-based (high-confidence)
NODE_6230_length_2090_cov_0.160221	2090	No	5	2	Low-quality	3.82	AAI-based (high-confidence)
NODE_6231_length_1134_cov_0.134300	1134	No	2	2	Low-quality	2.02	AAI-based (high-confidence)
NODE_6231_length_1275_cov_0.058673	1275	No	3	1	Low-quality	3.97	AAI-based (high-confidence)
NODE_6235_length_2384_cov_0.143107	2384	No	5	2	Low-quality	6.61	AAI-based (high-confidence)
NODE_624_length_5040_cov_0.345072	5040	No	6	2	Low-quality	13.98	AAI-based (high-confidence)
NODE_624_length_8913_cov_0.267415	8913	No	12	5	Low-quality	5.02	AAI-based (high-confidence)
NODE_6249_length_2646_cov_0.082843	2646	No	3	3	Low-quality	6.21	AAI-based (high-confidence)
NODE_625_length_8906_cov_0.451005	8906	No	14	3	Low-quality	25.36	AAI-based (high-confidence)
NODE_6258_length_2085_cov_0.153072	2085	No	6	1	Low-quality	6.16	AAI-based (high-confidence)
NODE_6262_length_2641_cov_0.118804	2641	No	2	2	Low-quality	5.88	AAI-based (high-confidence)
NODE_6263_length_1419_cov_0.075758	1419	No	3	1	Low-quality	2.26	AAI-based (high-confidence)
NODE_6284_length_2376_cov_0.128678	2376	No	5	2	Low-quality	4.04	AAI-based (high-confidence)
NODE_6292_length_2373_cov_0.173263	2373	No	6	1	Low-quality	4.62	AAI-based (high-confidence)
NODE_63_length_4164_cov_0.137515	4164	No	3	1	Low-quality	13.73	AAI-based (high-confidence)
NODE_63_length_8934_cov_0.542275	8934	No	11	7	Low-quality	28.14	AAI-based (high-confidence)
NODE_6300_length_1269_cov_0.181197	1269	No	2	1	Low-quality	2.04	AAI-based (high-confidence)
NODE_6303_length_1127_cov_0.147860	1127	No	2	1	Low-quality	3.44	AAI-based (high-confidence)
NODE_6311_length_2369_cov_0.115419	2369	No	3	3	Low-quality	3.97	AAI-based (high-confidence)

NODE_632_length_1855_cov_0.084852	1855	No	5	2	Low-quality	6.35	AAI-based (high-confidence)
NODE_632_length_7717_cov_0.254923	7717	No	6	4	Low-quality	12.76	AAI-based (high-confidence)
NODE_6321_length_2358_cov_0.117751	2358	No	4	3	Low-quality	5.66	AAI-based (high-confidence)
NODE_6323_length_2197_cov_0.149666	2197	No	7	1	Low-quality	7.04	AAI-based (high-confidence)
NODE_633_length_3482_cov_0.125628	3482	No	1	1	Low-quality	49.29	AAI-based (high-confidence)
NODE_6331_length_1409_cov_0.439695	1409	No	3	1	Low-quality	4.21	AAI-based (high-confidence)
NODE_6338_length_2364_cov_0.330243	2364	No	2	2	Low-quality	6.58	AAI-based (high-confidence)
NODE_634_length_3421_cov_0.173992	3421	No	6	1	Low-quality	10.56	AAI-based (high-confidence)
NODE_6341_length_2070_cov_0.312024	2070	No	7	1	Low-quality	3.6	AAI-based (high-confidence)
NODE_6343_length_2070_cov_0.177575	2070	No	7	3	Low-quality	3.45	AAI-based (high-confidence)
NODE_6344_length_2070_cov_0.120751	2070	No	4	2	Low-quality	4.9	AAI-based (high-confidence)
NODE_6346_length_2352_cov_0.106968	2352	No	3	1	Low-quality	7.39	AAI-based (high-confidence)
NODE_6349_length_2351_cov_0.457815	2351	No	4	2	Low-quality	4.36	AAI-based (high-confidence)
NODE_635_length_1946_cov_0.082296	1946	No	4	1	Low-quality	3.86	AAI-based (high-confidence)
NODE_636_length_1639_cov_0.087013	1639	No	4	1	Low-quality	3.25	AAI-based (high-confidence)
NODE_6368_length_2348_cov_0.702534	2348	No	7	2	Low-quality	7.29	AAI-based (high-confidence)
NODE_6371_length_1120_cov_0.315377	1120	No	4	1	Low-quality	2.14	AAI-based (high-confidence)
NODE_6374_length_2348_cov_0.104936	2348	No	2	1	Low-quality	4.53	AAI-based (high-confidence)
NODE_6374_length_2357_cov_0.162533	2357	No	10	2	Low-quality	7.05	AAI-based (high-confidence)
NODE_6375_length_1120_cov_0.109696	1120	No	1	1	Low-quality	3.1	AAI-based (high-confidence)
NODE_6381_length_1403_cov_0.106595	1403	No	4	2	Low-quality	2.44	AAI-based (high-confidence)
NODE_6382_length_1403_cov_0.084356	1403	No	1	1	Low-quality	2.19	AAI-based (high-confidence)
NODE_6386_length_2183_cov_0.118042	2183	No	3	1	Low-quality	6.64	AAI-based (high-confidence)
NODE_639_length_1723_cov_0.033867	1723	No	1	1	Low-quality	27.38	AAI-based (high-confidence)
NODE_639_length_2214_cov_0.147991	2214	No	1	1	Low-quality	47.13	AAI-based (high-confidence)
NODE_6392_length_2061_cov_0.220183	2061	No	6	1	Low-quality	6.24	AAI-based (high-confidence)
NODE_64_length_3650_cov_0.143058	3650	No	7	2	Low-quality	11.06	AAI-based (high-confidence)
NODE_64_length_3849_cov_0.171467	3849	No	1	1	Low-quality	41.94	AAI-based (high-confidence)
NODE_640_length_6615_cov_0.378146	6615	No	7	1	Low-quality	18.79	AAI-based (high-confidence)
NODE_6406_length_1117_cov_0.112967	1117	No	3	1	Low-quality	3.52	AAI-based (high-confidence)
NODE_6407_length_1399_cov_0.054615	1399	No	3	1	Low-quality	3.68	AAI-based (high-confidence)
NODE_6409_length_2592_cov_0.220618	2592	No	5	2	Low-quality	6.83	AAI-based (high-confidence)
NODE_641_length_1721_cov_0.055487	1721	No	1	1	Low-quality	25.68	AAI-based (high-confidence)
NODE_6419_length_1397_cov_0.066256	1397	No	2	1	Low-quality	4.5	AAI-based (high-confidence)
NODE_642_length_7635_cov_0.175027	7635	No	15	6	Low-quality	12.36	AAI-based (high-confidence)
NODE_642_length_8776_cov_0.206292	8776	No	13	1	Low-quality	13.46	AAI-based (high-confidence)
NODE_6422_length_2345_cov_0.116652	2345	No	2	1	Low-quality	3.87	AAI-based (high-confidence)
NODE_6425_length_2336_cov_0.092982	2336	No	5	1	Low-quality	7.12	AAI-based (high-confidence)
NODE_643_length_7382_cov_0.835370	7382	No	16	3	Low-quality	23.23	AAI-based (high-confidence)
NODE_6432_length_2334_cov_0.079642	2334	No	5	1	Low-quality	7.07	AAI-based (high-confidence)
NODE_6434_length_2333_cov_0.247090	2333	No	4	2	Low-quality	5.58	AAI-based (high-confidence)
NODE_6438_length_2342_cov_0.255907	2342	No	7	2	Low-quality	6.63	AAI-based (high-confidence)
NODE_6439_length_2054_cov_0.094118	2054	No	1	1	Low-quality	3.39	AAI-based (high-confidence)
NODE_6441_length_2332_cov_0.266458	2332	No	3	3	Low-quality	4.33	AAI-based (high-confidence)
NODE_6443_length_2053_cov_0.116172	2053	No	1	1	Low-quality	4.65	AAI-based (high-confidence)
NODE_6444_length_2053_cov_0.102866	2053	No	3	3	Low-quality	3.66	AAI-based (high-confidence)

NODE_6445_length_2340_cov_0.157073	2340	No	4	1	Low-quality	2.08	AAI-based (high-confidence)
NODE_645_length_7373_cov_0.141188	7373	No	10	6	Low-quality	3.09	AAI-based (high-confidence)
NODE_6454_length_2338_cov_0.574810	2338	No	2	1	Low-quality	4.55	AAI-based (high-confidence)
NODE_646_length_6593_cov_0.256083	6593	No	17	2	Low-quality	10.26	AAI-based (high-confidence)
NODE_646_length_8720_cov_1.081313	8720	No	16	2	Low-quality	9.52	AAI-based (high-confidence)
NODE_6468_length_1256_cov_0.041487	1256	No	2	2	Low-quality	2.05	AAI-based (high-confidence)
NODE_6469_length_2332_cov_0.290193	2332	No	4	2	Low-quality	5.51	AAI-based (high-confidence)
NODE_647_length_3449_cov_0.139403	3449	No	6	1	Low-quality	7.4	AAI-based (high-confidence)
NODE_6471_length_1255_cov_0.125433	1255	No	3	3	Low-quality	3.25	AAI-based (high-confidence)
NODE_6474_length_2323_cov_0.104766	2323	No	7	4	Low-quality	3.93	AAI-based (high-confidence)
NODE_6475_length_2330_cov_0.240699	2330	No	3	1	Low-quality	8.52	AAI-based (high-confidence)
NODE_6482_length_2166_cov_0.132559	2166	No	6	2	Low-quality	6.8	AAI-based (high-confidence)
NODE_65_length_3646_cov_0.118692	3646	No	11	2	Low-quality	11.42	AAI-based (high-confidence)
NODE_65_length_8909_cov_0.186039	8909	No	21	6	Low-quality	5.71	AAI-based (high-confidence)
NODE_650_length_1359_cov_0.073016	1359	No	6	1	Low-quality	4.2	AAI-based (high-confidence)
NODE_6512_length_1385_cov_0.118974	1385	No	1	1	Low-quality	2.18	AAI-based (high-confidence)
NODE_6514_length_2042_cov_0.398868	2042	No	6	2	Low-quality	4.92	AAI-based (high-confidence)
NODE_6518_length_2313_cov_0.196477	2313	No	6	1	Low-quality	3.85	AAI-based (high-confidence)
NODE_6519_length_1252_cov_0.040763	1252	No	2	2	Low-quality	2.24	AAI-based (high-confidence)
NODE_6520_length_2041_cov_0.106591	2041	No	2	2	Low-quality	3.67	AAI-based (high-confidence)
NODE_6521_length_1251_cov_0.126736	1251	No	3	1	Low-quality	3.04	AAI-based (high-confidence)
NODE_6521_length_2041_cov_0.106591	2041	No	1	1	Low-quality	4.82	AAI-based (high-confidence)
NODE_6523_length_2321_cov_0.126913	2321	No	5	1	Low-quality	3.66	AAI-based (high-confidence)
NODE_6525_length_2321_cov_0.095860	2321	No	4	1	Low-quality	6.3	AAI-based (high-confidence)
NODE_6527_length_1251_cov_0.078993	1251	No	4	2	Low-quality	3.82	AAI-based (high-confidence)
NODE_6529_length_2038_cov_0.283136	2038	No	1	1	Low-quality	3.36	AAI-based (high-confidence)
NODE_653_length_1939_cov_0.132609	1939	No	3	1	Low-quality	3.85	AAI-based (high-confidence)
NODE_653_length_3433_cov_0.090582	3433	No	6	2	Low-quality	8.77	AAI-based (high-confidence)
NODE_653_length_8672_cov_0.225942	8672	No	11	6	Low-quality	3.91	AAI-based (high-confidence)
NODE_6532_length_1383_cov_0.144081	1383	No	4	1	Low-quality	4.36	AAI-based (high-confidence)
NODE_6539_length_1383_cov_0.062305	1383	No	2	1	Low-quality	4.58	AAI-based (high-confidence)
NODE_654_length_1529_cov_0.112587	1529	No	2	2	Low-quality	2.52	AAI-based (high-confidence)
NODE_6543_length_2316_cov_0.136220	2316	No	2	1	Low-quality	3.53	AAI-based (high-confidence)
NODE_6545_length_2315_cov_0.204874	2315	No	5	4	Low-quality	5.34	AAI-based (high-confidence)
NODE_6553_length_2151_cov_0.211988	2151	No	3	2	Low-quality	6.84	AAI-based (high-confidence)
NODE_6559_length_1035_cov_0.081197	1035	No	1	1	Low-quality	3.07	AAI-based (high-confidence)
NODE_656_length_1643_cov_0.097798	1643	No	5	3	Low-quality	3.55	AAI-based (high-confidence)
NODE_656_length_3420_cov_0.174947	3420	No	5	4	Low-quality	1.93	AAI-based (high-confidence)
NODE_6568_length_2033_cov_0.343847	2033	No	4	2	Low-quality	3.83	AAI-based (high-confidence)
NODE_6568_length_2310_cov_0.241067	2310	No	4	2	Low-quality	6.08	AAI-based (high-confidence)
NODE_6569_length_2301_cov_0.220254	2301	No	2	2	Low-quality	4.23	AAI-based (high-confidence)
NODE_657_length_1640_cov_0.054510	1640	No	2	1	Low-quality	4.63	AAI-based (high-confidence)
NODE_6579_length_2145_cov_0.321114	2145	No	8	1	Low-quality	3.78	AAI-based (high-confidence)
NODE_6586_length_1033_cov_0.077088	1033	No	3	1	Low-quality	2.87	AAI-based (high-confidence)
NODE_659_length_6529_cov_0.228149	6529	No	11	5	Low-quality	3.72	AAI-based (high-confidence)
NODE_659_length_7300_cov_1.739203	7300	No	8	7	Low-quality	4.11	AAI-based (high-confidence)

NODE_6593_length_1100_cov_0.118881	1100	No	4	2	Low-quality	2.79	AAI-based (high-confidence)
NODE_6595_length_1376_cov_0.145654	1376	No	2	2	Low-quality	2.25	AAI-based (high-confidence)
NODE_6595_length_2030_cov_0.106680	2030	No	7	1	Low-quality	6.29	AAI-based (high-confidence)
NODE_6599_length_2297_cov_0.067789	2297	No	2	1	Low-quality	3.47	AAI-based (high-confidence)
NODE_66_length_3512_cov_0.104307	3512	No	7	4	Low-quality	8.33	AAI-based (high-confidence)
NODE_660_length_1701_cov_0.113608	1701	No	5	3	Low-quality	3.24	AAI-based (high-confidence)
NODE_660_length_6527_cov_0.297293	6527	No	10	2	Low-quality	19.96	AAI-based (high-confidence)
NODE_661_length_1623_cov_0.083990	1623	No	1	1	Low-quality	3.5	AAI-based (high-confidence)
NODE_661_length_6519_cov_0.252804	6519	No	8	6	Low-quality	6.23	AAI-based (high-confidence)
NODE_6613_length_2294_cov_0.199089	2294	No	2	1	Low-quality	5.09	AAI-based (high-confidence)
NODE_6625_length_2298_cov_0.436562	2298	No	2	2	Low-quality	5.57	AAI-based (high-confidence)
NODE_6628_length_1098_cov_0.090090	1098	No	4	1	Low-quality	2.96	AAI-based (high-confidence)
NODE_663_length_1354_cov_0.054183	1354	No	4	1	Low-quality	4.18	AAI-based (high-confidence)
NODE_6631_length_2134_cov_0.136609	2134	No	5	1	Low-quality	6.96	AAI-based (high-confidence)
NODE_6632_length_2023_cov_0.120582	2023	No	2	2	Low-quality	6.6	AAI-based (high-confidence)
NODE_6645_length_2289_cov_0.523288	2289	No	2	2	Low-quality	4.05	AAI-based (high-confidence)
NODE_6646_length_1029_cov_0.107527	1029	No	2	1	Low-quality	2.05	AAI-based (high-confidence)
NODE_665_length_3387_cov_0.131387	3387	No	5	2	Low-quality	2.06	AAI-based (high-confidence)
NODE_6651_length_2292_cov_0.389877	2292	No	1	1	Low-quality	4.11	AAI-based (high-confidence)
NODE_6654_length_2292_cov_0.178751	2292	No	7	1	Low-quality	3.86	AAI-based (high-confidence)
NODE_6659_length_2019_cov_0.091146	2019	No	2	1	Low-quality	3.08	AAI-based (high-confidence)
NODE_6665_length_1241_cov_0.039405	1241	No	3	1	Low-quality	2.9	AAI-based (high-confidence)
NODE_667_length_4898_cov_0.570744	4898	No	11	2	Low-quality	15.21	AAI-based (high-confidence)
NODE_6670_length_2286_cov_0.112483	2286	No	5	1	Low-quality	3.74	AAI-based (high-confidence)
NODE_6678_length_1239_cov_0.150877	1239	No	6	1	Low-quality	3.92	AAI-based (high-confidence)
NODE_668_length_1348_cov_0.088070	1348	No	2	2	Low-quality	2.75	AAI-based (high-confidence)
NODE_6683_length_2125_cov_0.076505	2125	No	3	1	Low-quality	6.56	AAI-based (high-confidence)
NODE_6689_length_1364_cov_0.109091	1364	No	2	1	Low-quality	2.65	AAI-based (high-confidence)
NODE_67_length_4119_cov_0.142040	4119	No	12	3	Low-quality	3.61	AAI-based (high-confidence)
NODE_67_length_8793_cov_0.261790	8793	No	15	7	Low-quality	14.22	AAI-based (high-confidence)
NODE_6701_length_2281_cov_0.117324	2281	No	5	1	Low-quality	6.39	AAI-based (high-confidence)
NODE_6702_length_2122_cov_0.108255	2122	No	4	2	Low-quality	5.78	AAI-based (high-confidence)
NODE_6709_length_2280_cov_0.103164	2280	No	4	3	Low-quality	5.37	AAI-based (high-confidence)
NODE_671_length_1813_cov_0.070012	1813	No	3	3	Low-quality	4.65	AAI-based (high-confidence)
NODE_6718_length_2281_cov_0.090284	2281	No	4	2	Low-quality	6.19	AAI-based (high-confidence)
NODE_6721_length_2278_cov_0.112437	2278	No	3	2	Low-quality	4.37	AAI-based (high-confidence)
NODE_6727_length_2279_cov_0.217431	2279	No	2	2	Low-quality	3.77	AAI-based (high-confidence)
NODE_6730_length_1236_cov_0.055409	1236	No	3	1	Low-quality	2.9	AAI-based (high-confidence)
NODE_6734_length_2278_cov_0.313447	2278	No	4	1	Low-quality	5.57	AAI-based (high-confidence)
NODE_674_length_6465_cov_0.193999	6465	No	6	2	Low-quality	4.26	AAI-based (high-confidence)
NODE_6744_length_1358_cov_0.096902	1358	No	4	1	Low-quality	2.48	AAI-based (high-confidence)
NODE_6747_length_1235_cov_0.087148	1235	No	2	1	Low-quality	3.77	AAI-based (high-confidence)
NODE_6755_length_2275_cov_0.127298	2275	No	6	3	Low-quality	4.56	AAI-based (high-confidence)
NODE_6756_length_1235_cov_0.042254	1235	No	3	1	Low-quality	2.37	AAI-based (high-confidence)
NODE_676_length_8525_cov_0.226679	8525	No	11	2	Low-quality	5.17	AAI-based (high-confidence)
NODE_6763_length_2004_cov_0.129134	2004	No	4	2	Low-quality	5.73	AAI-based (high-confidence)

NODE_6764_length_2004_cov_0.129134	2004	No	6	3	Low-quality	3.92	AAI-based (high-confidence)
NODE_6768_length_1234_cov_0.041410	1234	No	2	2	Low-quality	2.13	AAI-based (high-confidence)
NODE_6773_length_2107_cov_0.124004	2107	No	3	1	Low-quality	4.09	AAI-based (high-confidence)
NODE_6773_length_2268_cov_0.141540	2268	No	5	1	Low-quality	3.67	AAI-based (high-confidence)
NODE_6775_length_1085_cov_0.045639	1085	No	2	1	Low-quality	2.65	AAI-based (high-confidence)
NODE_6778_length_1353_cov_0.110048	1353	No	2	1	Low-quality	4.14	AAI-based (high-confidence)
NODE_6790_length_2491_cov_0.139214	2491	No	4	2	Low-quality	7.69	AAI-based (high-confidence)
NODE_6793_length_2103_cov_0.130240	2103	No	3	1	Low-quality	4.85	AAI-based (high-confidence)
NODE_6795_length_1999_cov_0.167368	1999	No	5	3	Low-quality	4.1	AAI-based (high-confidence)
NODE_680_length_1921_cov_0.102086	1921	No	4	1	Low-quality	4.91	AAI-based (high-confidence)
NODE_6807_length_2484_cov_0.170231	2484	No	6	2	Low-quality	4.78	AAI-based (high-confidence)
NODE_6808_length_2264_cov_0.757968	2264	No	2	2	Low-quality	4	AAI-based (high-confidence)
NODE_681_length_7430_cov_0.229437	7430	No	14	2	Low-quality	23.39	AAI-based (high-confidence)
NODE_6811_length_1230_cov_0.221043	1230	No	4	2	Low-quality	3.63	AAI-based (high-confidence)
NODE_6823_length_2256_cov_0.168289	2256	No	4	2	Low-quality	6.95	AAI-based (high-confidence)
NODE_6823_length_2261_cov_0.114709	2261	No	3	2	Low-quality	4.16	AAI-based (high-confidence)
NODE_6827_length_1995_cov_0.115506	1995	No	5	1	Low-quality	3.37	AAI-based (high-confidence)
NODE_6828_length_1346_cov_0.054531	1346	No	6	1	Low-quality	2.75	AAI-based (high-confidence)
NODE_683_length_1804_cov_0.133724	1804	No	4	1	Low-quality	4.65	AAI-based (high-confidence)
NODE_683_length_6441_cov_0.216966	6441	No	16	3	Low-quality	20.18	AAI-based (high-confidence)
NODE_6838_length_1344_cov_0.142972	1344	No	3	1	Low-quality	4.02	AAI-based (high-confidence)
NODE_6847_length_2257_cov_0.109361	2257	No	6	1	Low-quality	4.24	AAI-based (high-confidence)
NODE_685_length_1918_cov_0.072567	1918	No	2	2	Low-quality	3.57	AAI-based (high-confidence)
NODE_6857_length_2249_cov_0.478605	2249	No	2	2	Low-quality	3.5	AAI-based (high-confidence)
NODE_6883_length_2244_cov_0.225641	2244	No	7	2	Low-quality	6.78	AAI-based (high-confidence)
NODE_6883_length_2248_cov_0.174965	2248	No	3	1	Low-quality	3.48	AAI-based (high-confidence)
NODE_6888_length_2082_cov_0.469491	2082	No	5	3	Low-quality	3.56	AAI-based (high-confidence)
NODE_689_length_3339_cov_0.363889	3339	No	7	1	Low-quality	3.11	AAI-based (high-confidence)
NODE_6896_length_1985_cov_0.119300	1985	No	4	2	Low-quality	7.67	AAI-based (high-confidence)
NODE_6898_length_2241_cov_0.373016	2241	No	7	1	Low-quality	3.63	AAI-based (high-confidence)
NODE_69_length_24013_cov_0.345363	24013	No	16	1	Low-quality	24.36	AAI-based (high-confidence)
NODE_6902_length_2080_cov_0.213529	2080	No	3	3	Low-quality	6.18	AAI-based (high-confidence)
NODE_691_length_8461_cov_0.400502	8461	No	8	7	Low-quality	19.88	AAI-based (high-confidence)
NODE_6914_length_2238_cov_0.263675	2238	No	5	1	Low-quality	4.65	AAI-based (high-confidence)
NODE_692_length_1874_cov_0.118310	1874	No	1	1	Low-quality	29.59	AAI-based (high-confidence)
NODE_692_length_1911_cov_0.072848	1911	No	1	1	Low-quality	4.66	AAI-based (high-confidence)
NODE_6921_length_2237_cov_0.367633	2237	No	2	2	Low-quality	3.73	AAI-based (high-confidence)
NODE_6921_length_2241_cov_0.161998	2241	No	4	1	Low-quality	6.63	AAI-based (high-confidence)
NODE_6928_length_1334_cov_0.157085	1334	No	3	1	Low-quality	2.54	AAI-based (high-confidence)
NODE_693_length_3303_cov_0.261548	3303	No	2	2	Low-quality	5.46	AAI-based (high-confidence)
NODE_6932_length_1333_cov_0.172609	1333	No	4	1	Low-quality	2.27	AAI-based (high-confidence)
NODE_6934_length_2073_cov_0.119554	2073	No	3	2	Low-quality	5.68	AAI-based (high-confidence)
NODE_6942_length_2233_cov_0.360825	2233	No	6	1	Low-quality	3.48	AAI-based (high-confidence)
NODE_6951_length_1221_cov_0.084670	1221	No	1	1	Low-quality	2.29	AAI-based (high-confidence)
NODE_696_length_1625_cov_0.301442	1625	No	1	1	Low-quality	22.07	AAI-based (high-confidence)
NODE_696_length_4807_cov_0.168012	4807	No	4	2	Low-quality	10.48	AAI-based (high-confidence)

NODE_696_length_6395_cov_0.267630	6395	No	5	2	Low-quality	9.85	AAI-based (high-confidence)
NODE_697_length_3330_cov_0.088827	3330	No	6	4	Low-quality	6.35	AAI-based (high-confidence)
NODE_697_length_4806_cov_0.170172	4806	No	8	2	Low-quality	2.71	AAI-based (high-confidence)
NODE_6973_length_1974_cov_0.205333	1974	No	3	1	Low-quality	3.28	AAI-based (high-confidence)
NODE_6977_length_2226_cov_0.109544	2226	No	5	1	Low-quality	6.52	AAI-based (high-confidence)
NODE_698_length_1598_cov_0.064043	1598	No	4	3	Low-quality	4.03	AAI-based (high-confidence)
NODE_6983_length_1327_cov_0.106678	1327	No	3	1	Low-quality	3.01	AAI-based (high-confidence)
NODE_6984_length_2230_cov_0.618020	2230	No	3	1	Low-quality	3.57	AAI-based (high-confidence)
NODE_6986_length_2230_cov_0.196152	2230	No	4	2	Low-quality	2	AAI-based (high-confidence)
NODE_6996_length_2229_cov_0.093897	2229	No	5	1	Low-quality	4.9	AAI-based (high-confidence)
NODE_7_length_23230_cov_0.187022	23230	No	43	8	Low-quality	33.15	AAI-based (high-confidence)
NODE_7_length_7225_cov_0.224109	7225	No	9	3	Low-quality	20.2	AAI-based (high-confidence)
NODE_7_length_8100_cov_0.467442	8100	No	5	2	Low-quality	13.34	AAI-based (high-confidence)
NODE_7_length_9930_cov_0.137524	9930	No	12	5	Low-quality	30.95	AAI-based (high-confidence)
NODE_70_length_3535_cov_0.109430	3535	No	8	2	Low-quality	10.26	AAI-based (high-confidence)
NODE_700_length_6385_cov_0.213490	6385	No	6	3	Low-quality	11.24	AAI-based (high-confidence)
NODE_7002_length_1325_cov_0.374388	1325	No	2	1	Low-quality	3.96	AAI-based (high-confidence)
NODE_7002_length_2219_cov_0.270755	2219	No	5	2	Low-quality	3.84	AAI-based (high-confidence)
NODE_7003_length_2219_cov_0.225000	2219	No	2	1	Low-quality	4.15	AAI-based (high-confidence)
NODE_7004_length_1969_cov_0.322995	1969	No	2	1	Low-quality	3.63	AAI-based (high-confidence)
NODE_7005_length_1969_cov_0.175401	1969	No	3	1	Low-quality	3.02	AAI-based (high-confidence)
NODE_701_length_2150_cov_0.545100	2150	No	1	1	Low-quality	33.73	AAI-based (high-confidence)
NODE_7013_length_2217_cov_0.296034	2217	No	7	2	Low-quality	3.79	AAI-based (high-confidence)
NODE_702_length_1663_cov_0.104859	1663	No	2	1	Low-quality	3.49	AAI-based (high-confidence)
NODE_703_length_8393_cov_0.725585	8393	No	20	5	Low-quality	24.86	AAI-based (high-confidence)
NODE_704_length_8391_cov_0.334057	8391	No	12	6	Low-quality	19.34	AAI-based (high-confidence)
NODE_705_length_3300_cov_0.138394	3300	No	4	2	Low-quality	4.93	AAI-based (high-confidence)
NODE_7052_length_1213_cov_0.084381	1213	No	2	1	Low-quality	3.68	AAI-based (high-confidence)
NODE_7054_length_1318_cov_0.113208	1318	No	3	1	Low-quality	4.03	AAI-based (high-confidence)
NODE_706_length_1587_cov_0.081989	1587	No	4	2	Low-quality	2.74	AAI-based (high-confidence)
NODE_706_length_7016_cov_0.398872	7016	No	18	5	Low-quality	11.37	AAI-based (high-confidence)
NODE_7066_length_2205_cov_0.158120	2205	No	5	2	Low-quality	6.13	AAI-based (high-confidence)
NODE_707_length_6346_cov_0.192733	6346	No	10	3	Low-quality	3.35	AAI-based (high-confidence)
NODE_707_length_7259_cov_0.226257	7259	No	10	7	Low-quality	6.93	AAI-based (high-confidence)
NODE_7070_length_2216_cov_0.397733	2216	No	3	3	Low-quality	3.83	AAI-based (high-confidence)
NODE_7071_length_2204_cov_0.408076	2204	No	2	1	Low-quality	4.1	AAI-based (high-confidence)
NODE_7079_length_1003_cov_0.056416	1003	No	3	2	Low-quality	2.67	AAI-based (high-confidence)
NODE_708_length_1488_cov_0.064795	1488	No	2	2	Low-quality	2.51	AAI-based (high-confidence)
NODE_7091_length_1957_cov_0.104952	1957	No	4	2	Low-quality	3.57	AAI-based (high-confidence)
NODE_7093_length_2212_cov_0.106010	2212	No	3	3	Low-quality	4.13	AAI-based (high-confidence)
NODE_7095_length_1210_cov_0.070207	1210	No	3	2	Low-quality	2.18	AAI-based (high-confidence)
NODE_710_length_3283_cov_0.118719	3283	No	7	2	Low-quality	1.99	AAI-based (high-confidence)
NODE_710_length_4766_cov_0.357617	4766	No	13	2	Low-quality	12.9	AAI-based (high-confidence)
NODE_7102_length_2200_cov_0.176583	2200	No	3	1	Low-quality	5.1	AAI-based (high-confidence)
NODE_7103_length_2209_cov_0.372038	2209	No	3	3	Low-quality	3.7	AAI-based (high-confidence)
NODE_7109_length_2199_cov_0.206667	2199	No	4	1	Low-quality	3.54	AAI-based (high-confidence)

NODE_7110_length_1209_cov_0.063964	1209	No	3	1	Low-quality	2.04	AAI-based (high-confidence)
NODE_7110_length_2208_cov_0.629208	2208	No	2	2	Low-quality	3.81	AAI-based (high-confidence)
NODE_712_length_1779_cov_0.096429	1779	No	5	1	Low-quality	5.23	AAI-based (high-confidence)
NODE_7129_length_2195_cov_0.356393	2195	No	8	2	Low-quality	7.04	AAI-based (high-confidence)
NODE_713_length_10093_cov_0.114369	10093	No	15	4	Low-quality	18.78	AAI-based (high-confidence)
NODE_713_length_1850_cov_0.260994	1850	No	1	1	Low-quality	27.22	AAI-based (high-confidence)
NODE_7132_length_1207_cov_0.096570	1207	No	4	2	Low-quality	3.43	AAI-based (high-confidence)
NODE_7138_length_2397_cov_0.144473	2397	No	2	2	Low-quality	4.44	AAI-based (high-confidence)
NODE_715_length_3276_cov_0.279509	3276	No	3	3	Low-quality	10.36	AAI-based (high-confidence)
NODE_715_length_6973_cov_0.980070	6973	No	8	4	Low-quality	11.97	AAI-based (high-confidence)
NODE_7151_length_2192_cov_0.054467	2192	No	2	1	Low-quality	6.04	AAI-based (high-confidence)
NODE_7155_length_2190_cov_0.175036	2190	No	5	1	Low-quality	3.48	AAI-based (high-confidence)
NODE_716_length_4752_cov_0.140125	4752	No	5	2	Low-quality	2.48	AAI-based (high-confidence)
NODE_7173_length_1052_cov_0.186779	1052	No	1	1	Low-quality	2.56	AAI-based (high-confidence)
NODE_7175_length_2195_cov_0.107824	2195	No	4	1	Low-quality	4.43	AAI-based (high-confidence)
NODE_7181_length_2027_cov_0.110996	2027	No	2	1	Low-quality	4.86	AAI-based (high-confidence)
NODE_7187_length_1204_cov_0.015385	1204	No	2	1	Low-quality	2.01	AAI-based (high-confidence)
NODE_719_length_1776_cov_0.118068	1776	No	7	2	Low-quality	2.77	AAI-based (high-confidence)
NODE_7190_length_1203_cov_0.163949	1203	No	2	1	Low-quality	3.97	AAI-based (high-confidence)
NODE_7194_length_2190_cov_0.918221	2190	No	6	3	Low-quality	6.08	AAI-based (high-confidence)
NODE_72_length_3973_cov_0.141714	3973	No	8	4	Low-quality	6.44	AAI-based (high-confidence)
NODE_720_length_1776_cov_0.116875	1776	No	1	1	Low-quality	26.87	AAI-based (high-confidence)
NODE_7200_length_1203_cov_0.051630	1203	No	2	1	Low-quality	2.01	AAI-based (high-confidence)
NODE_7205_length_1202_cov_0.135086	1202	No	2	2	Low-quality	2.08	AAI-based (high-confidence)
NODE_7211_length_2182_cov_0.180509	2182	No	5	2	Low-quality	6.5	AAI-based (high-confidence)
NODE_7212_length_1940_cov_0.228680	1940	No	3	1	Low-quality	5.67	AAI-based (high-confidence)
NODE_7214_length_2022_cov_0.081643	2022	No	5	4	Low-quality	2.04	AAI-based (high-confidence)
NODE_722_length_1579_cov_0.099324	1579	No	4	3	Low-quality	2.98	AAI-based (high-confidence)
NODE_7229_length_1200_cov_0.178020	1200	No	3	1	Low-quality	3.66	AAI-based (high-confidence)
NODE_723_length_1578_cov_0.096011	1578	No	3	1	Low-quality	2.92	AAI-based (high-confidence)
NODE_723_length_6283_cov_1.377587	6283	No	11	3	Low-quality	3.63	AAI-based (high-confidence)
NODE_7236_length_1299_cov_0.063333	1299	No	2	1	Low-quality	2.18	AAI-based (high-confidence)
NODE_7237_length_1299_cov_0.061667	1299	No	3	2	Low-quality	4.05	AAI-based (high-confidence)
NODE_724_length_1575_cov_0.056233	1575	No	2	2	Low-quality	8.25	AAI-based (high-confidence)
NODE_7242_length_2180_cov_0.137914	2180	No	2	1	Low-quality	3.78	AAI-based (high-confidence)
NODE_7244_length_1298_cov_0.098415	1298	No	2	2	Low-quality	3.32	AAI-based (high-confidence)
NODE_7264_length_2174_cov_0.317108	2174	No	5	1	Low-quality	4.93	AAI-based (high-confidence)
NODE_7269_length_1296_cov_0.061821	1296	No	3	1	Low-quality	4.01	AAI-based (high-confidence)
NODE_7280_length_1198_cov_0.050955	1198	No	4	1	Low-quality	2.21	AAI-based (high-confidence)
NODE_7282_length_2172_cov_0.177521	2172	No	3	1	Low-quality	3.95	AAI-based (high-confidence)
NODE_7287_length_1294_cov_0.125523	1294	No	2	1	Low-quality	3.3	AAI-based (high-confidence)
NODE_729_length_1637_cov_0.102731	1637	No	5	1	Low-quality	2.65	AAI-based (high-confidence)
NODE_7292_length_2170_cov_0.225012	2170	No	4	3	Low-quality	1.99	AAI-based (high-confidence)
NODE_7299_length_2169_cov_0.217874	2169	No	2	1	Low-quality	6.62	AAI-based (high-confidence)
NODE_73_length_3769_cov_0.122888	3769	No	8	4	Low-quality	6.1	AAI-based (high-confidence)
NODE_7311_length_2167_cov_0.134913	2167	No	3	2	Low-quality	4.77	AAI-based (high-confidence)

NODE_7313_length_2166_cov_0.146589	2166	No	4	1	Low-quality	5.02	AAI-based (high-confidence)
NODE_7313_length_2167_cov_0.104932	2167	No	3	2	Low-quality	4.96	AAI-based (high-confidence)
NODE_7324_length_2006_cov_0.128998	2006	No	4	1	Low-quality	5.84	AAI-based (high-confidence)
NODE_7329_length_2354_cov_0.137472	2354	No	2	1	Low-quality	4.38	AAI-based (high-confidence)
NODE_734_length_1632_cov_0.103718	1632	No	4	2	Low-quality	3.34	AAI-based (high-confidence)
NODE_734_length_3237_cov_0.205226	3237	No	5	1	Low-quality	5.18	AAI-based (high-confidence)
NODE_735_length_6239_cov_0.386971	6239	No	10	3	Low-quality	14.32	AAI-based (high-confidence)
NODE_7352_length_1192_cov_0.106130	1192	No	1	1	Low-quality	19.57	AAI-based (high-confidence)
NODE_7352_length_1288_cov_0.100084	1288	No	3	1	Low-quality	3.99	AAI-based (high-confidence)
NODE_736_length_1760_cov_0.123420	1760	No	4	2	Low-quality	5.27	AAI-based (high-confidence)
NODE_7381_length_1919_cov_0.130769	1919	No	3	2	Low-quality	3.44	AAI-based (high-confidence)
NODE_7389_length_2153_cov_0.144109	2153	No	4	1	Low-quality	3.37	AAI-based (high-confidence)
NODE_7391_length_1037_cov_0.214286	1037	No	3	1	Low-quality	2.06	AAI-based (high-confidence)
NODE_7398_length_1037_cov_0.058635	1037	No	5	1	Low-quality	3.1	AAI-based (high-confidence)
NODE_74_length_45215_cov_0.259797	45215	No	34	5	Low-quality	45.93	AAI-based (high-confidence)
NODE_74_length_8400_cov_0.232382	8400	No	8	1	Low-quality	23.82	AAI-based (high-confidence)
NODE_7401_length_1992_cov_0.142631	1992	No	5	1	Low-quality	6.68	AAI-based (high-confidence)
NODE_7403_length_1991_cov_0.135307	1991	No	5	3	Low-quality	5.45	AAI-based (high-confidence)
NODE_7418_length_1034_cov_0.232086	1034	No	2	1	Low-quality	3.86	AAI-based (high-confidence)
NODE_7418_length_2145_cov_0.085533	2145	No	4	2	Low-quality	5.77	AAI-based (high-confidence)
NODE_742_length_1866_cov_0.073005	1866	No	3	2	Low-quality	5.07	AAI-based (high-confidence)
NODE_7424_length_1187_cov_0.124081	1187	No	2	1	Low-quality	3.6	AAI-based (high-confidence)
NODE_7428_length_1187_cov_0.090074	1187	No	3	1	Low-quality	2.29	AAI-based (high-confidence)
NODE_7429_length_2144_cov_0.070905	2144	No	4	3	Low-quality	6.07	AAI-based (high-confidence)
NODE_7429_length_2146_cov_0.394724	2146	No	4	4	Low-quality	2.17	AAI-based (high-confidence)
NODE_7460_length_2136_cov_0.181149	2136	No	3	2	Low-quality	3.51	AAI-based (high-confidence)
NODE_7461_length_2136_cov_0.180167	2136	No	4	1	Low-quality	3.05	AAI-based (high-confidence)
NODE_7472_length_1905_cov_0.139535	1905	No	4	2	Low-quality	6.08	AAI-based (high-confidence)
NODE_7479_length_1183_cov_0.074723	1183	No	2	2	Low-quality	2.58	AAI-based (high-confidence)
NODE_7487_length_1275_cov_0.118197	1275	No	3	1	Low-quality	3.49	AAI-based (high-confidence)
NODE_7489_length_1903_cov_0.302661	1903	No	5	2	Low-quality	4.61	AAI-based (high-confidence)
NODE_7491_length_2135_cov_0.331041	2135	No	6	1	Low-quality	2.09	AAI-based (high-confidence)
NODE_7508_length_1974_cov_0.396800	1974	No	2	2	Low-quality	3.32	AAI-based (high-confidence)
NODE_7508_length_2311_cov_0.111212	2311	No	1	1	Low-quality	5.33	AAI-based (high-confidence)
NODE_752_length_1810_cov_0.089421	1810	No	3	1	Low-quality	4.64	AAI-based (high-confidence)
NODE_753_length_6167_cov_0.440508	6167	No	6	2	Low-quality	19.41	AAI-based (high-confidence)
NODE_7539_length_2123_cov_0.135375	2123	No	2	2	Low-quality	5.99	AAI-based (high-confidence)
NODE_754_length_1745_cov_0.085055	1745	No	3	3	Low-quality	3.01	AAI-based (high-confidence)
NODE_7540_length_1176_cov_0.117920	1176	No	1	1	Low-quality	2.52	AAI-based (high-confidence)
NODE_7545_length_1176_cov_0.074280	1176	No	1	1	Low-quality	2.6	AAI-based (high-confidence)
NODE_755_length_6154_cov_0.542527	6154	No	11	3	Low-quality	5.13	AAI-based (high-confidence)
NODE_7553_length_1268_cov_0.072712	1268	No	2	2	Low-quality	3.49	AAI-based (high-confidence)
NODE_7557_length_1267_cov_0.353596	1267	No	3	1	Low-quality	3.51	AAI-based (high-confidence)
NODE_7562_length_2120_cov_0.299852	2120	No	2	2	Low-quality	3.51	AAI-based (high-confidence)
NODE_757_length_1551_cov_0.072314	1551	No	4	2	Low-quality	2.68	AAI-based (high-confidence)
NODE_757_length_9825_cov_0.130681	9825	No	15	5	Low-quality	17.9	AAI-based (high-confidence)

NODE_7580_length_1265_cov_0.154374	1265	No	3	1	Low-quality	3.85	AAI-based (high-confidence)
NODE_7593_length_1172_cov_0.267474	1172	No	4	1	Low-quality	2.24	AAI-based (high-confidence)
NODE_7599_length_1263_cov_0.102234	1263	No	3	1	Low-quality	2.34	AAI-based (high-confidence)
NODE_760_length_1608_cov_0.092777	1608	No	3	1	Low-quality	4.96	AAI-based (high-confidence)
NODE_760_length_1741_cov_0.077954	1741	No	1	1	Low-quality	28.25	AAI-based (high-confidence)
NODE_7607_length_1171_cov_0.141791	1171	No	3	2	Low-quality	2.17	AAI-based (high-confidence)
NODE_761_length_6117_cov_0.137920	6117	No	9	1	Low-quality	7.49	AAI-based (high-confidence)
NODE_762_length_1606_cov_0.120106	1606	No	1	1	Low-quality	23.46	AAI-based (high-confidence)
NODE_7624_length_1170_cov_0.149393	1170	No	4	2	Low-quality	2.58	AAI-based (high-confidence)
NODE_7626_length_1261_cov_0.077453	1261	No	3	1	Low-quality	3.68	AAI-based (high-confidence)
NODE_7628_length_1888_cov_0.095584	1888	No	6	2	Low-quality	5.63	AAI-based (high-confidence)
NODE_7629_length_2280_cov_0.584136	2280	No	8	1	Low-quality	6.22	AAI-based (high-confidence)
NODE_763_length_1797_cov_0.167256	1797	No	4	1	Low-quality	3.12	AAI-based (high-confidence)
NODE_7633_length_1887_cov_0.122483	1887	No	2	1	Low-quality	3.45	AAI-based (high-confidence)
NODE_7635_length_1170_cov_0.046685	1170	No	1	1	Low-quality	3.24	AAI-based (high-confidence)
NODE_7638_length_2107_cov_0.088645	2107	No	5	1	Low-quality	3.51	AAI-based (high-confidence)
NODE_7644_length_2277_cov_0.166208	2277	No	9	1	Low-quality	3.56	AAI-based (high-confidence)
NODE_7647_length_2114_cov_0.198015	2114	No	3	1	Low-quality	3.19	AAI-based (high-confidence)
NODE_765_length_1835_cov_0.097926	1835	No	2	1	Low-quality	4.45	AAI-based (high-confidence)
NODE_7656_length_1258_cov_0.122519	1258	No	3	3	Low-quality	2.96	AAI-based (high-confidence)
NODE_7659_length_1883_cov_0.153027	1883	No	3	3	Low-quality	3.46	AAI-based (high-confidence)
NODE_766_length_1456_cov_0.114223	1456	No	4	4	Low-quality	3.11	AAI-based (high-confidence)
NODE_766_length_1599_cov_0.098000	1599	No	1	1	Low-quality	25.26	AAI-based (high-confidence)
NODE_766_length_8011_cov_0.343276	8011	No	5	2	Low-quality	23.31	AAI-based (high-confidence)
NODE_7665_length_1257_cov_0.090674	1257	No	1	1	Low-quality	2.07	AAI-based (high-confidence)
NODE_767_length_4564_cov_0.217469	4564	No	4	2	Low-quality	7.14	AAI-based (high-confidence)
NODE_767_length_6091_cov_0.221462	6091	No	8	4	Low-quality	15.87	AAI-based (high-confidence)
NODE_7670_length_1167_cov_0.090824	1167	No	1	1	Low-quality	2.21	AAI-based (high-confidence)
NODE_7679_length_2109_cov_0.210448	2109	No	2	1	Low-quality	3.63	AAI-based (high-confidence)
NODE_7683_length_2109_cov_0.093035	2109	No	4	2	Low-quality	6.48	AAI-based (high-confidence)
NODE_7684_length_1166_cov_0.228679	1166	No	2	1	Low-quality	3.68	AAI-based (high-confidence)
NODE_769_length_6751_cov_0.202495	6751	No	7	3	Low-quality	19.31	AAI-based (high-confidence)
NODE_7693_length_1878_cov_0.316470	1878	No	5	1	Low-quality	4.66	AAI-based (high-confidence)
NODE_77_length_2293_cov_0.108478	2293	No	3	1	Low-quality	6.4	AAI-based (high-confidence)
NODE_77_length_2898_cov_0.123258	2898	No	5	1	Low-quality	8.37	AAI-based (high-confidence)
NODE_77_length_3448_cov_0.171693	3448	No	4	2	Low-quality	9.59	AAI-based (high-confidence)
NODE_770_length_8001_cov_0.357631	8001	No	10	3	Low-quality	2.29	AAI-based (high-confidence)
NODE_7705_length_1942_cov_0.357027	1942	No	2	2	Low-quality	4.76	AAI-based (high-confidence)
NODE_7705_length_2266_cov_0.136133	2266	No	6	1	Low-quality	3.08	AAI-based (high-confidence)
NODE_7706_length_2266_cov_0.113060	2266	No	2	2	Low-quality	5.63	AAI-based (high-confidence)
NODE_771_length_6080_cov_0.201304	6080	No	8	3	Low-quality	3.61	AAI-based (high-confidence)
NODE_7720_length_1164_cov_0.072300	1164	No	4	2	Low-quality	2.63	AAI-based (high-confidence)
NODE_7724_length_2100_cov_0.171914	2100	No	5	1	Low-quality	2.57	AAI-based (high-confidence)
NODE_7731_length_2094_cov_0.829574	2094	No	2	1	Low-quality	6.44	AAI-based (high-confidence)
NODE_7732_length_2094_cov_0.532331	2094	No	6	1	Low-quality	3.39	AAI-based (high-confidence)
NODE_7736_length_1248_cov_0.268930	1248	No	3	2	Low-quality	3.5	AAI-based (high-confidence)

NODE_774_length_6075_cov_0.298527	6075	No	12	5	Low-quality	18.44	AAI-based (high-confidence)
NODE_775_length_1733_cov_0.122399	1733	No	2	1	Low-quality	2.91	AAI-based (high-confidence)
NODE_7756_length_1010_cov_0.074643	1010	No	3	1	Low-quality	2.4	AAI-based (high-confidence)
NODE_7758_length_2095_cov_1.688878	2095	No	4	2	Low-quality	4.95	AAI-based (high-confidence)
NODE_776_length_1732_cov_0.085732	1732	No	4	1	Low-quality	4.44	AAI-based (high-confidence)
NODE_776_length_6066_cov_0.190045	6066	No	7	5	Low-quality	13.72	AAI-based (high-confidence)
NODE_7767_length_2091_cov_0.078313	2091	No	6	1	Low-quality	6.26	AAI-based (high-confidence)
NODE_777_length_6726_cov_0.643428	6726	No	12	4	Low-quality	10.82	AAI-based (high-confidence)
NODE_7770_length_1868_cov_0.159977	1868	No	3	1	Low-quality	3.55	AAI-based (high-confidence)
NODE_7771_length_2090_cov_0.265696	2090	No	7	1	Low-quality	3.86	AAI-based (high-confidence)
NODE_7773_length_2090_cov_0.101457	2090	No	2	1	Low-quality	3.53	AAI-based (high-confidence)
NODE_7775_length_2090_cov_0.063787	2090	No	7	5	Low-quality	3.82	AAI-based (high-confidence)
NODE_7778_length_1008_cov_0.108911	1008	No	2	1	Low-quality	3.12	AAI-based (high-confidence)
NODE_7783_length_2091_cov_0.285141	2091	No	7	2	Low-quality	4.54	AAI-based (high-confidence)
NODE_78_length_14723_cov_0.470801	14723	No	15	9	Low-quality	36.56	AAI-based (high-confidence)
NODE_78_length_3543_cov_0.108595	3543	No	6	1	Low-quality	9.81	AAI-based (high-confidence)
NODE_7809_length_1925_cov_0.115553	1925	No	3	2	Low-quality	4.86	AAI-based (high-confidence)
NODE_7812_length_1863_cov_0.106009	1863	No	5	2	Low-quality	7.27	AAI-based (high-confidence)
NODE_782_length_6706_cov_0.456486	6706	No	8	3	Low-quality	11.1	AAI-based (high-confidence)
NODE_783_length_3152_cov_0.272191	3152	No	5	3	Low-quality	8.24	AAI-based (high-confidence)
NODE_783_length_7920_cov_0.382943	7920	No	17	4	Low-quality	24.34	AAI-based (high-confidence)
NODE_7831_length_1860_cov_0.124361	1860	No	3	1	Low-quality	2.98	AAI-based (high-confidence)
NODE_7831_length_2082_cov_0.089259	2082	No	3	3	Low-quality	3.48	AAI-based (high-confidence)
NODE_7831_length_2083_cov_0.152218	2083	No	3	1	Low-quality	3.91	AAI-based (high-confidence)
NODE_784_length_1289_cov_0.052941	1289	No	4	1	Low-quality	2.5	AAI-based (high-confidence)
NODE_7843_length_2080_cov_0.103988	2080	No	2	1	Low-quality	3.86	AAI-based (high-confidence)
NODE_7846_length_1236_cov_0.111697	1236	No	5	1	Low-quality	2.08	AAI-based (high-confidence)
NODE_785_length_7912_cov_0.335211	7912	No	18	4	Low-quality	21.37	AAI-based (high-confidence)
NODE_7851_length_2079_cov_0.134343	2079	No	3	1	Low-quality	6.51	AAI-based (high-confidence)
NODE_7853_length_1236_cov_0.040457	1236	No	2	2	Low-quality	3.82	AAI-based (high-confidence)
NODE_7855_length_2079_cov_0.073232	2079	No	2	2	Low-quality	5.74	AAI-based (high-confidence)
NODE_7858_length_1235_cov_0.110035	1235	No	2	1	Low-quality	3.78	AAI-based (high-confidence)
NODE_7861_length_2078_cov_0.142496	2078	No	2	1	Low-quality	3.89	AAI-based (high-confidence)
NODE_7873_length_2076_cov_0.221548	2076	No	4	2	Low-quality	5.06	AAI-based (high-confidence)
NODE_7876_length_2229_cov_0.068075	2229	No	4	3	Low-quality	4.5	AAI-based (high-confidence)
NODE_788_length_7905_cov_0.358186	7905	No	14	1	Low-quality	6.61	AAI-based (high-confidence)
NODE_7904_length_2072_cov_0.158135	2072	No	4	3	Low-quality	3.6	AAI-based (high-confidence)
NODE_7908_length_1910_cov_0.094423	1910	No	3	1	Low-quality	3.84	AAI-based (high-confidence)
NODE_791_length_1528_cov_0.145556	1528	No	2	1	Low-quality	3.05	AAI-based (high-confidence)
NODE_791_length_1769_cov_0.118563	1769	No	4	1	Low-quality	5.48	AAI-based (high-confidence)
NODE_791_length_6674_cov_0.421293	6674	No	9	7	Low-quality	6.37	AAI-based (high-confidence)
NODE_791_length_7881_cov_0.227833	7881	No	13	8	Low-quality	21.4	AAI-based (high-confidence)
NODE_792_length_3139_cov_0.120724	3139	No	4	2	Low-quality	9.56	AAI-based (high-confidence)
NODE_7922_length_2070_cov_0.173516	2070	No	4	3	Low-quality	1.99	AAI-based (high-confidence)
NODE_7931_length_2068_cov_0.286948	2068	No	2	2	Low-quality	3.77	AAI-based (high-confidence)
NODE_7942_length_1904_cov_0.131856	1904	No	3	3	Low-quality	4.09	AAI-based (high-confidence)

NODE_7942_length_2067_cov_0.177337	2067	No	6	4	Low-quality	3.73	AAI-based (high-confidence)
NODE_795_length_6006_cov_0.200440	6006	No	15	1	Low-quality	4.78	AAI-based (high-confidence)
NODE_795_length_6656_cov_0.280006	6656	No	13	4	Low-quality	3.41	AAI-based (high-confidence)
NODE_796_length_1526_cov_0.172390	1526	No	1	1	Low-quality	22.03	AAI-based (high-confidence)
NODE_7963_length_1842_cov_0.171543	1842	No	4	1	Low-quality	3.64	AAI-based (high-confidence)
NODE_7965_length_2062_cov_0.170657	2062	No	6	3	Low-quality	6.17	AAI-based (high-confidence)
NODE_797_length_6653_cov_0.258316	6653	No	6	2	Low-quality	3.73	AAI-based (high-confidence)
NODE_7977_length_1841_cov_0.107922	1841	No	3	1	Low-quality	5.4	AAI-based (high-confidence)
NODE_7978_length_1841_cov_0.105052	1841	No	1	1	Low-quality	4.91	AAI-based (high-confidence)
NODE_7978_length_2212_cov_0.892097	2212	No	3	2	Low-quality	4.09	AAI-based (high-confidence)
NODE_7985_length_2061_cov_0.090724	2061	No	3	2	Low-quality	3.38	AAI-based (high-confidence)
NODE_7987_length_2060_cov_0.447221	2060	No	2	2	Low-quality	3.43	AAI-based (high-confidence)
NODE_799_length_6730_cov_0.308551	6730	No	20	3	Low-quality	19.77	AAI-based (high-confidence)
NODE_7995_length_2059_cov_0.338265	2059	No	2	1	Low-quality	5.36	AAI-based (high-confidence)
NODE_8_length_6131_cov_0.131963	6131	No	11	4	Low-quality	18.88	AAI-based (high-confidence)
NODE_8_length_8468_cov_0.167642	8468	No	11	4	Low-quality	23.55	AAI-based (high-confidence)
NODE_8_length_8717_cov_0.191460	8717	No	16	6	Low-quality	24.08	AAI-based (high-confidence)
NODE_80_length_3348_cov_0.085565	3348	No	5	1	Low-quality	11.46	AAI-based (high-confidence)
NODE_80_length_3360_cov_0.103956	3360	No	3	2	Low-quality	5.48	AAI-based (high-confidence)
NODE_80_length_3676_cov_0.128040	3676	No	3	2	Low-quality	6.1	AAI-based (high-confidence)
NODE_800_length_1803_cov_0.104460	1803	No	2	1	Low-quality	3.03	AAI-based (high-confidence)
NODE_800_length_5993_cov_1.132168	5993	No	7	4	Low-quality	3.41	AAI-based (high-confidence)
NODE_8000_length_1838_cov_0.171938	1838	No	2	1	Low-quality	3.35	AAI-based (high-confidence)
NODE_8003_length_1145_cov_0.184512	1145	No	2	2	Low-quality	2.1	AAI-based (high-confidence)
NODE_8008_length_2057_cov_0.172114	2057	No	4	2	Low-quality	6.74	AAI-based (high-confidence)
NODE_8010_length_2057_cov_0.149132	2057	No	4	2	Low-quality	5.75	AAI-based (high-confidence)
NODE_8012_length_2057_cov_0.127171	2057	No	3	2	Low-quality	6.25	AAI-based (high-confidence)
NODE_8016_length_1895_cov_0.479399	1895	No	5	1	Low-quality	3.35	AAI-based (high-confidence)
NODE_8026_length_2054_cov_0.430179	2054	No	6	1	Low-quality	6.23	AAI-based (high-confidence)
NODE_8032_length_1834_cov_0.329107	1834	No	3	2	Low-quality	3.32	AAI-based (high-confidence)
NODE_8033_length_1834_cov_0.187320	1834	No	3	1	Low-quality	5.32	AAI-based (high-confidence)
NODE_804_length_4461_cov_0.192343	4461	No	10	3	Low-quality	13.24	AAI-based (high-confidence)
NODE_804_length_6616_cov_0.171705	6616	No	9	4	Low-quality	12.03	AAI-based (high-confidence)
NODE_8047_length_1219_cov_0.275000	1219	No	3	1	Low-quality	3.92	AAI-based (high-confidence)
NODE_8047_length_2051_cov_0.089652	2051	No	2	2	Low-quality	3.73	AAI-based (high-confidence)
NODE_8054_length_1832_cov_0.079054	1832	No	5	2	Low-quality	5.46	AAI-based (high-confidence)
NODE_806_length_4457_cov_0.449289	4457	No	8	3	Low-quality	9.81	AAI-based (high-confidence)
NODE_806_length_5970_cov_0.171180	5970	No	6	2	Low-quality	3.94	AAI-based (high-confidence)
NODE_8060_length_2047_cov_0.118583	2047	No	5	2	Low-quality	3.68	AAI-based (high-confidence)
NODE_8062_length_2047_cov_0.089836	2047	No	2	1	Low-quality	3.87	AAI-based (high-confidence)
NODE_8071_length_1141_cov_0.089251	1141	No	3	2	Low-quality	1.93	AAI-based (high-confidence)
NODE_8076_length_2045_cov_0.093525	2045	No	5	1	Low-quality	6.36	AAI-based (high-confidence)
NODE_808_length_2069_cov_0.099492	2069	No	2	1	Low-quality	5.3	AAI-based (high-confidence)
NODE_8081_length_1828_cov_0.299017	1828	No	3	1	Low-quality	3.45	AAI-based (high-confidence)
NODE_8083_length_1216_cov_0.350045	1216	No	4	2	Low-quality	3.41	AAI-based (high-confidence)
NODE_8085_length_2043_cov_0.231481	2043	No	3	1	Low-quality	5.66	AAI-based (high-confidence)

NODE_8088_length_1216_cov_0.079678	1216	No	2	1	Low-quality	3.71	AAI-based (high-confidence)
NODE_8094_length_2042_cov_0.407102	2042	No	5	2	Low-quality	4.27	AAI-based (high-confidence)
NODE_8095_length_2042_cov_0.109110	2042	No	4	1	Low-quality	5.84	AAI-based (high-confidence)
NODE_8097_length_1214_cov_0.145291	1214	No	4	1	Low-quality	2.67	AAI-based (high-confidence)
NODE_8098_length_2041_cov_2.163234	2041	No	4	1	Low-quality	4.83	AAI-based (high-confidence)
NODE_81_length_3354_cov_0.214439	3354	No	1	1	Low-quality	45.67	AAI-based (high-confidence)
NODE_81_length_3814_cov_0.092059	3814	No	4	2	Low-quality	6.34	AAI-based (high-confidence)
NODE_8114_length_2039_cov_0.175773	2039	No	5	1	Low-quality	4.73	AAI-based (high-confidence)
NODE_812_length_4431_cov_0.394968	4431	No	5	2	Low-quality	13.91	AAI-based (high-confidence)
NODE_8127_length_1879_cov_0.192135	1879	No	1	1	Low-quality	3.11	AAI-based (high-confidence)
NODE_8138_length_2039_cov_0.177320	2039	No	2	2	Low-quality	3.39	AAI-based (high-confidence)
NODE_8143_length_2038_cov_0.281073	2038	No	3	3	Low-quality	3.5	AAI-based (high-confidence)
NODE_8146_length_2034_cov_0.439276	2034	No	4	1	Low-quality	6.14	AAI-based (high-confidence)
NODE_8154_length_2034_cov_0.018088	2034	No	4	3	Low-quality	5.59	AAI-based (high-confidence)
NODE_8155_length_1135_cov_0.071429	1135	No	2	1	Low-quality	2.09	AAI-based (high-confidence)
NODE_816_length_1510_cov_0.152374	1510	No	3	1	Low-quality	5.29	AAI-based (high-confidence)
NODE_816_length_1702_cov_0.112289	1702	No	3	1	Low-quality	5	AAI-based (high-confidence)
NODE_8171_length_2034_cov_0.364858	2034	No	3	1	Low-quality	6.03	AAI-based (high-confidence)
NODE_8189_length_2032_cov_0.130367	2032	No	6	1	Low-quality	3.33	AAI-based (high-confidence)
NODE_8190_length_1206_cov_0.062331	1206	No	2	1	Low-quality	2.44	AAI-based (high-confidence)
NODE_82_length_3353_cov_0.105716	3353	No	4	1	Low-quality	10.47	AAI-based (high-confidence)
NODE_82_length_3887_cov_0.098205	3887	No	8	3	Low-quality	6.69	AAI-based (high-confidence)
NODE_820_length_3054_cov_0.217259	3054	No	7	1	Low-quality	8.27	AAI-based (high-confidence)
NODE_8202_length_1812_cov_0.121424	1812	No	2	1	Low-quality	3.12	AAI-based (high-confidence)
NODE_8203_length_1812_cov_0.104495	1812	No	4	2	Low-quality	5.71	AAI-based (high-confidence)
NODE_8216_length_1203_cov_1.595109	1203	No	1	1	Low-quality	2.92	AAI-based (high-confidence)
NODE_8216_length_2028_cov_0.657854	2028	No	2	2	Low-quality	6.36	AAI-based (high-confidence)
NODE_822_length_1507_cov_0.178267	1507	No	1	1	Low-quality	23.66	AAI-based (high-confidence)
NODE_822_length_3079_cov_0.199329	3079	No	5	1	Low-quality	10.49	AAI-based (high-confidence)
NODE_8220_length_2024_cov_0.082597	2024	No	2	1	Low-quality	3.45	AAI-based (high-confidence)
NODE_8224_length_2026_cov_0.186819	2026	No	4	1	Low-quality	3.76	AAI-based (high-confidence)
NODE_8228_length_2022_cov_0.205928	2022	No	5	1	Low-quality	5.49	AAI-based (high-confidence)
NODE_8229_length_1202_cov_0.306437	1202	No	3	1	Low-quality	2.73	AAI-based (high-confidence)
NODE_823_length_1700_cov_0.130543	1700	No	2	1	Low-quality	37.25	AAI-based (high-confidence)
NODE_8233_length_1807_cov_0.350703	1807	No	5	1	Low-quality	5.72	AAI-based (high-confidence)
NODE_8238_length_1129_cov_0.133981	1129	No	3	1	Low-quality	3.36	AAI-based (high-confidence)
NODE_824_length_6561_cov_0.463788	6561	No	12	5	Low-quality	18.45	AAI-based (high-confidence)
NODE_824_length_6579_cov_0.190586	6579	No	4	2	Low-quality	3.31	AAI-based (high-confidence)
NODE_8241_length_1862_cov_0.137266	1862	No	3	1	Low-quality	5.73	AAI-based (high-confidence)
NODE_8242_length_2024_cov_0.105455	2024	No	1	1	Low-quality	6.13	AAI-based (high-confidence)
NODE_825_length_1505_cov_0.128023	1505	No	1	1	Low-quality	31.33	AAI-based (high-confidence)
NODE_826_length_1505_cov_0.109531	1505	No	2	1	Low-quality	4.73	AAI-based (high-confidence)
NODE_826_length_1740_cov_0.079220	1740	No	2	1	Low-quality	26.32	AAI-based (high-confidence)
NODE_8263_length_1804_cov_0.328446	1804	No	3	2	Low-quality	4.52	AAI-based (high-confidence)
NODE_827_length_1739_cov_0.068293	1739	No	2	1	Low-quality	4.23	AAI-based (high-confidence)
NODE_8275_length_2014_cov_0.153525	2014	No	3	2	Low-quality	2.7	AAI-based (high-confidence)

NODE_829_length_3030_cov_0.123849	3030	No	3	1	Low-quality	9.58	AAI-based (high-confidence)
NODE_83_length_14100_cov_0.454682	14100	No	13	9	Low-quality	7.89	AAI-based (high-confidence)
NODE_83_length_2263_cov_0.090111	2263	No	6	1	Low-quality	3.66	AAI-based (high-confidence)
NODE_8300_length_1126_cov_0.033106	1126	No	3	2	Low-quality	3.2	AAI-based (high-confidence)
NODE_8308_length_2014_cov_0.161358	2014	No	4	2	Low-quality	3.43	AAI-based (high-confidence)
NODE_831_length_1775_cov_0.139618	1775	No	1	1	Low-quality	35.77	AAI-based (high-confidence)
NODE_8314_length_2009_cov_0.143979	2009	No	2	2	Low-quality	4.6	AAI-based (high-confidence)
NODE_8321_length_1796_cov_0.379493	1796	No	4	2	Low-quality	4.55	AAI-based (high-confidence)
NODE_8323_length_2008_cov_0.203772	2008	No	7	3	Low-quality	3.34	AAI-based (high-confidence)
NODE_8326_length_2007_cov_0.623166	2007	No	5	1	Low-quality	6.22	AAI-based (high-confidence)
NODE_8333_length_1194_cov_0.143379	1194	No	4	1	Low-quality	2.94	AAI-based (high-confidence)
NODE_8335_length_1123_cov_0.171875	1123	No	3	1	Low-quality	3.59	AAI-based (high-confidence)
NODE_8335_length_1194_cov_0.112329	1194	No	3	2	Low-quality	3.29	AAI-based (high-confidence)
NODE_8339_length_2005_cov_0.443861	2005	No	2	1	Low-quality	3.31	AAI-based (high-confidence)
NODE_8341_length_2004_cov_0.276115	2004	No	3	2	Low-quality	6.06	AAI-based (high-confidence)
NODE_835_length_1732_cov_0.105940	1732	No	1	1	Low-quality	34.11	AAI-based (high-confidence)
NODE_8354_length_2135_cov_1.418468	2135	No	6	1	Low-quality	6.01	AAI-based (high-confidence)
NODE_8359_length_2001_cov_0.208728	2001	No	5	1	Low-quality	6.23	AAI-based (high-confidence)
NODE_8364_length_1122_cov_0.060606	1122	No	4	1	Low-quality	3.32	AAI-based (high-confidence)
NODE_8366_length_1840_cov_0.368754	1840	No	4	1	Low-quality	5.39	AAI-based (high-confidence)
NODE_8370_length_2000_cov_0.103630	2000	No	3	2	Low-quality	4.91	AAI-based (high-confidence)
NODE_8379_length_1998_cov_0.183781	1998	No	3	1	Low-quality	6.16	AAI-based (high-confidence)
NODE_838_length_3061_cov_0.528359	3061	No	8	1	Low-quality	8.26	AAI-based (high-confidence)
NODE_8381_length_1998_cov_0.167457	1998	No	2	2	Low-quality	3.66	AAI-based (high-confidence)
NODE_839_length_1494_cov_0.139068	1494	No	2	1	Low-quality	2.95	AAI-based (high-confidence)
NODE_839_length_1729_cov_0.106748	1729	No	1	1	Low-quality	27.07	AAI-based (high-confidence)
NODE_8390_length_2001_cov_0.133544	2001	No	4	2	Low-quality	3.9	AAI-based (high-confidence)
NODE_8393_length_1996_cov_0.683184	1996	No	2	1	Low-quality	3.38	AAI-based (high-confidence)
NODE_84_length_13897_cov_0.517539	13897	No	14	11	Low-quality	32.69	AAI-based (high-confidence)
NODE_84_length_7846_cov_0.135665	7846	No	16	5	Low-quality	12.69	AAI-based (high-confidence)
NODE_8405_length_1995_cov_0.257384	1995	No	6	1	Low-quality	3.26	AAI-based (high-confidence)
NODE_841_length_3057_cov_0.135565	3057	No	5	3	Low-quality	8.01	AAI-based (high-confidence)
NODE_8426_length_1784_cov_0.472404	1784	No	2	1	Low-quality	4.34	AAI-based (high-confidence)
NODE_8433_length_1784_cov_0.099110	1784	No	4	2	Low-quality	4.49	AAI-based (high-confidence)
NODE_8434_length_1117_cov_0.097250	1117	No	2	1	Low-quality	2.22	AAI-based (high-confidence)
NODE_844_length_1419_cov_0.059091	1419	No	2	1	Low-quality	3	AAI-based (high-confidence)
NODE_844_length_9252_cov_0.119414	9252	No	10	8	Low-quality	21.62	AAI-based (high-confidence)
NODE_845_length_1418_cov_0.159970	1418	No	2	1	Low-quality	3.88	AAI-based (high-confidence)
NODE_8462_length_1781_cov_0.086801	1781	No	4	1	Low-quality	4.67	AAI-based (high-confidence)
NODE_847_length_1680_cov_0.290955	1680	No	1	1	Low-quality	24.59	AAI-based (high-confidence)
NODE_8470_length_1828_cov_0.271255	1828	No	6	3	Low-quality	3.07	AAI-based (high-confidence)
NODE_848_length_5844_cov_0.148825	5844	No	9	2	Low-quality	13.18	AAI-based (high-confidence)
NODE_849_length_2043_cov_0.097737	2043	No	2	1	Low-quality	3.47	AAI-based (high-confidence)
NODE_8501_length_1775_cov_0.325776	1775	No	2	2	Low-quality	2.92	AAI-based (high-confidence)
NODE_8508_length_1988_cov_0.164637	1988	No	5	1	Low-quality	6.06	AAI-based (high-confidence)
NODE_8510_length_1775_cov_0.102029	1775	No	1	1	Low-quality	5.22	AAI-based (high-confidence)

NODE_8512_length_1988_cov_0.115405	1988	No	3	1	Low-quality	5.78	AAI-based (high-confidence)
NODE_8525_length_1985_cov_0.243372	1985	No	5	3	Low-quality	3.08	AAI-based (high-confidence)
NODE_853_length_1507_cov_0.117898	1507	No	3	1	Low-quality	2.85	AAI-based (high-confidence)
NODE_8531_length_1772_cov_0.209205	1772	No	4	2	Low-quality	3.91	AAI-based (high-confidence)
NODE_8533_length_1111_cov_0.117589	1111	No	3	2	Low-quality	2.27	AAI-based (high-confidence)
NODE_8535_length_1111_cov_0.102767	1111	No	3	1	Low-quality	2.06	AAI-based (high-confidence)
NODE_8535_length_1978_cov_0.083555	1978	No	2	1	Low-quality	3.12	AAI-based (high-confidence)
NODE_854_length_1485_cov_0.072150	1485	No	4	1	Low-quality	3	AAI-based (high-confidence)
NODE_8549_length_1975_cov_0.163113	1975	No	6	1	Low-quality	3.56	AAI-based (high-confidence)
NODE_855_length_5800_cov_0.648307	5800	No	14	1	Low-quality	7.16	AAI-based (high-confidence)
NODE_8553_length_1769_cov_0.132934	1769	No	2	2	Low-quality	4.29	AAI-based (high-confidence)
NODE_856_length_3031_cov_0.318213	3031	No	4	2	Low-quality	9.18	AAI-based (high-confidence)
NODE_8565_length_1174_cov_0.242791	1174	No	2	1	Low-quality	1.91	AAI-based (high-confidence)
NODE_8571_length_1173_cov_0.236499	1173	No	3	1	Low-quality	2.86	AAI-based (high-confidence)
NODE_8578_length_1980_cov_0.128123	1980	No	1	1	Low-quality	3.69	AAI-based (high-confidence)
NODE_859_length_1712_cov_0.183509	1712	No	2	1	Low-quality	3.21	AAI-based (high-confidence)
NODE_8590_length_1108_cov_0.417245	1108	No	2	1	Low-quality	3.06	AAI-based (high-confidence)
NODE_8598_length_1763_cov_0.277043	1763	No	3	1	Low-quality	7.27	AAI-based (high-confidence)
NODE_86_length_2252_cov_0.058059	2252	No	3	2	Low-quality	6.95	AAI-based (high-confidence)
NODE_86_length_3857_cov_0.188132	3857	No	1	1	Low-quality	46.24	AAI-based (high-confidence)
NODE_862_length_1489_cov_0.091367	1489	No	1	1	Low-quality	23.29	AAI-based (high-confidence)
NODE_8639_length_1168_cov_0.071094	1168	No	4	1	Low-quality	3.19	AAI-based (high-confidence)
NODE_8646_length_1105_cov_0.059642	1105	No	1	1	Low-quality	2.06	AAI-based (high-confidence)
NODE_8656_length_1963_cov_0.655579	1963	No	3	1	Low-quality	5.97	AAI-based (high-confidence)
NODE_8657_length_1756_cov_0.191913	1756	No	2	1	Low-quality	4.48	AAI-based (high-confidence)
NODE_866_length_4256_cov_0.286745	4256	No	14	3	Low-quality	11.84	AAI-based (high-confidence)
NODE_8671_length_1962_cov_0.074074	1962	No	1	1	Low-quality	3.25	AAI-based (high-confidence)
NODE_8679_length_1164_cov_0.133333	1164	No	2	1	Low-quality	3.61	AAI-based (high-confidence)
NODE_8695_length_1959_cov_0.068817	1959	No	5	1	Low-quality	4.35	AAI-based (high-confidence)
NODE_87_length_2245_cov_0.092731	2245	No	4	3	Low-quality	4.78	AAI-based (high-confidence)
NODE_87_length_2817_cov_0.190213	2817	No	5	1	Low-quality	8.77	AAI-based (high-confidence)
NODE_870_length_1255_cov_0.062284	1255	No	1	1	Low-quality	2.07	AAI-based (high-confidence)
NODE_8709_length_1750_cov_0.141127	1750	No	5	2	Low-quality	5.54	AAI-based (high-confidence)
NODE_871_length_1665_cov_0.202427	1665	No	1	1	Low-quality	35.47	AAI-based (high-confidence)
NODE_8714_length_1100_cov_0.129870	1100	No	2	1	Low-quality	3.47	AAI-based (high-confidence)
NODE_872_length_1254_cov_0.031169	1254	No	3	1	Low-quality	3.8	AAI-based (high-confidence)
NODE_8732_length_1794_cov_0.089676	1794	No	4	1	Low-quality	3.38	AAI-based (high-confidence)
NODE_8732_length_2067_cov_0.144309	2067	No	5	2	Low-quality	5.45	AAI-based (high-confidence)
NODE_874_length_1664_cov_0.179553	1664	No	3	1	Low-quality	2.66	AAI-based (high-confidence)
NODE_8740_length_1099_cov_0.108000	1099	No	3	2	Low-quality	2.95	AAI-based (high-confidence)
NODE_8740_length_1747_cov_0.174150	1747	No	2	2	Low-quality	3.39	AAI-based (high-confidence)
NODE_875_length_1705_cov_0.273973	1705	No	1	1	Low-quality	27.8	AAI-based (high-confidence)
NODE_875_length_1738_cov_0.174497	1738	No	4	2	Low-quality	3.37	AAI-based (high-confidence)
NODE_8750_length_1746_cov_0.129326	1746	No	2	2	Low-quality	3.52	AAI-based (high-confidence)
NODE_8753_length_1746_cov_0.098968	1746	No	6	1	Low-quality	2.87	AAI-based (high-confidence)
NODE_876_length_7399_cov_0.302877	7399	No	8	3	Low-quality	4.18	AAI-based (high-confidence)

NODE_8760_length_1958_cov_0.270038	1958	No	4	2	Low-quality	5.14	AAI-based (high-confidence)
NODE_8769_length_1947_cov_0.395563	1947	No	6	1	Low-quality	4.64	AAI-based (high-confidence)
NODE_8790_length_1955_cov_0.379310	1955	No	2	2	Low-quality	3.16	AAI-based (high-confidence)
NODE_88_length_20791_cov_0.564566	20791	No	36	11	Low-quality	35.36	AAI-based (high-confidence)
NODE_88_length_3841_cov_0.332710	3841	No	4	2	Low-quality	10.7	AAI-based (high-confidence)
NODE_8801_length_1954_cov_0.173046	1954	No	1	1	Low-quality	3.22	AAI-based (high-confidence)
NODE_881_length_2994_cov_0.460104	2994	No	8	4	Low-quality	9.23	AAI-based (high-confidence)
NODE_8820_length_1951_cov_0.203024	1951	No	4	2	Low-quality	5.58	AAI-based (high-confidence)
NODE_883_length_5676_cov_0.262327	5676	No	8	3	Low-quality	2.91	AAI-based (high-confidence)
NODE_883_length_6344_cov_0.437630	6344	No	8	1	Low-quality	18.82	AAI-based (high-confidence)
NODE_884_length_1488_cov_0.077754	1488	No	4	1	Low-quality	3.72	AAI-based (high-confidence)
NODE_884_length_2991_cov_0.108921	2991	No	3	1	Low-quality	4.8	AAI-based (high-confidence)
NODE_8845_length_1779_cov_0.150595	1779	No	3	2	Low-quality	4.61	AAI-based (high-confidence)
NODE_8855_length_1150_cov_0.252141	1150	No	2	1	Low-quality	2.64	AAI-based (high-confidence)
NODE_8857_length_1094_cov_0.081407	1094	No	3	1	Low-quality	2.36	AAI-based (high-confidence)
NODE_886_length_4199_cov_0.232195	4199	No	9	3	Low-quality	6.79	AAI-based (high-confidence)
NODE_887_length_6329_cov_0.479133	6329	No	14	3	Low-quality	6.94	AAI-based (high-confidence)
NODE_8873_length_1093_cov_0.103622	1093	No	2	1	Low-quality	1.92	AAI-based (high-confidence)
NODE_8878_length_1943_cov_0.418655	1943	No	4	1	Low-quality	4.83	AAI-based (high-confidence)
NODE_8880_length_1774_cov_0.133134	1774	No	3	1	Low-quality	3.38	AAI-based (high-confidence)
NODE_8889_length_1773_cov_0.112903	1773	No	3	3	Low-quality	3.72	AAI-based (high-confidence)
NODE_8889_length_1929_cov_0.340437	1929	No	1	1	Low-quality	6.17	AAI-based (high-confidence)
NODE_889_length_1656_cov_0.150931	1656	No	1	1	Low-quality	33.55	AAI-based (high-confidence)
NODE_889_length_1698_cov_0.093183	1698	No	2	1	Low-quality	23.61	AAI-based (high-confidence)
NODE_889_length_7366_cov_0.687491	7366	No	9	3	Low-quality	11.12	AAI-based (high-confidence)
NODE_8890_length_1929_cov_0.321858	1929	No	3	1	Low-quality	4.55	AAI-based (high-confidence)
NODE_8907_length_1770_cov_0.404548	1770	No	1	1	Low-quality	3.68	AAI-based (high-confidence)
NODE_891_length_1399_cov_0.107692	1399	No	3	1	Low-quality	4.11	AAI-based (high-confidence)
NODE_891_length_1654_cov_0.118971	1654	No	4	2	Low-quality	4.61	AAI-based (high-confidence)
NODE_8919_length_1926_cov_0.194308	1926	No	2	2	Low-quality	3.21	AAI-based (high-confidence)
NODE_893_length_1722_cov_0.119532	1722	No	5	1	Low-quality	5.16	AAI-based (high-confidence)
NODE_8939_length_1727_cov_0.204545	1727	No	4	2	Low-quality	5.21	AAI-based (high-confidence)
NODE_894_length_7351_cov_0.377275	7351	No	8	4	Low-quality	2	AAI-based (high-confidence)
NODE_8943_length_1935_cov_0.559913	1935	No	2	2	Low-quality	5.4	AAI-based (high-confidence)
NODE_8945_length_1090_cov_0.028254	1090	No	1	1	Low-quality	2.82	AAI-based (high-confidence)
NODE_8947_length_1922_cov_0.536478	1922	No	1	1	Low-quality	3.17	AAI-based (high-confidence)
NODE_8952_length_1144_cov_0.022967	1144	No	2	2	Low-quality	3.2	AAI-based (high-confidence)
NODE_8953_length_1934_cov_0.111172	1934	No	7	1	Low-quality	6	AAI-based (high-confidence)
NODE_896_length_1651_cov_0.062500	1651	No	3	1	Low-quality	4.55	AAI-based (high-confidence)
NODE_8964_length_1724_cov_0.094769	1724	No	2	1	Low-quality	3.21	AAI-based (high-confidence)
NODE_8965_length_1933_cov_0.077972	1933	No	4	3	Low-quality	3.93	AAI-based (high-confidence)
NODE_897_length_2970_cov_0.151863	2970	No	7	4	Low-quality	8.19	AAI-based (high-confidence)
NODE_8970_length_1723_cov_0.243842	1723	No	4	3	Low-quality	3.11	AAI-based (high-confidence)
NODE_8976_length_1932_cov_0.041462	1932	No	2	1	Low-quality	3.26	AAI-based (high-confidence)
NODE_898_length_4161_cov_0.258986	4161	No	2	1	Low-quality	9.72	AAI-based (high-confidence)
NODE_898_length_6294_cov_0.737046	6294	No	5	3	Low-quality	10.41	AAI-based (high-confidence)

NODE_8989_length_1087_cov_0.080972	1087	No	2	2	Low-quality	1.99	AAI-based (high-confidence)
NODE_899_length_6291_cov_0.214147	6291	No	8	3	Low-quality	7.82	AAI-based (high-confidence)
NODE_9_length_5783_cov_0.100457	5783	No	8	4	Low-quality	16.21	AAI-based (high-confidence)
NODE_9_length_8660_cov_0.124285	8660	No	14	7	Low-quality	13.83	AAI-based (high-confidence)
NODE_9009_length_2016_cov_1.053208	2016	No	6	1	Low-quality	5.68	AAI-based (high-confidence)
NODE_9014_length_1916_cov_0.082003	1916	No	2	2	Low-quality	3.56	AAI-based (high-confidence)
NODE_9017_length_1915_cov_0.198789	1915	No	3	1	Low-quality	3.72	AAI-based (high-confidence)
NODE_9019_length_1758_cov_0.081374	1758	No	1	1	Low-quality	4.27	AAI-based (high-confidence)
NODE_9023_length_1137_cov_0.142582	1137	No	4	1	Low-quality	3.52	AAI-based (high-confidence)
NODE_903_length_1502_cov_0.148254	1502	No	2	1	Low-quality	4.2	AAI-based (high-confidence)
NODE_904_length_7293_cov_0.251182	7293	No	3	3	Low-quality	3.28	AAI-based (high-confidence)
NODE_905_length_1475_cov_0.068314	1475	No	3	1	Low-quality	3.71	AAI-based (high-confidence)
NODE_906_length_5638_cov_0.522477	5638	No	12	5	Low-quality	17.65	AAI-based (high-confidence)
NODE_907_length_1451_cov_0.082101	1451	No	3	1	Low-quality	4.23	AAI-based (high-confidence)
NODE_907_length_7290_cov_0.256710	7290	No	9	3	Low-quality	3.67	AAI-based (high-confidence)
NODE_908_length_2891_cov_0.368553	2891	No	6	1	Low-quality	3.63	AAI-based (high-confidence)
NODE_908_length_4136_cov_0.443399	4136	No	6	1	Low-quality	11.84	AAI-based (high-confidence)
NODE_9080_length_1711_cov_0.199132	1711	No	5	1	Low-quality	2.1	AAI-based (high-confidence)
NODE_9092_length_1906_cov_0.074156	1906	No	5	1	Low-quality	4.39	AAI-based (high-confidence)
NODE_9097_length_1709_cov_0.274534	1709	No	5	1	Low-quality	4.55	AAI-based (high-confidence)
NODE_91_length_3267_cov_0.099747	3267	No	5	3	Low-quality	5.19	AAI-based (high-confidence)
NODE_9108_length_1708_cov_0.128651	1708	No	1	1	Low-quality	3.07	AAI-based (high-confidence)
NODE_911_length_1709_cov_0.088199	1709	No	4	2	Low-quality	8.95	AAI-based (high-confidence)
NODE_9110_length_1998_cov_0.656135	1998	No	7	1	Low-quality	5.58	AAI-based (high-confidence)
NODE_9115_length_1903_cov_0.080931	1903	No	3	2	Low-quality	4.69	AAI-based (high-confidence)
NODE_912_length_5620_cov_0.260279	5620	No	8	3	Low-quality	2.83	AAI-based (high-confidence)
NODE_9122_length_1707_cov_0.070274	1707	No	3	1	Low-quality	4.7	AAI-based (high-confidence)
NODE_913_length_6261_cov_0.530834	6261	No	8	3	Low-quality	9.74	AAI-based (high-confidence)
NODE_9131_length_1900_cov_0.459745	1900	No	7	1	Low-quality	5.97	AAI-based (high-confidence)
NODE_9139_length_1704_cov_0.142679	1704	No	5	1	Low-quality	3.1	AAI-based (high-confidence)
NODE_9139_length_1910_cov_0.139150	1910	No	3	1	Low-quality	3.71	AAI-based (high-confidence)
NODE_915_length_1707_cov_0.090174	1707	No	1	1	Low-quality	2.86	AAI-based (high-confidence)
NODE_915_length_2950_cov_0.230445	2950	No	4	1	Low-quality	2.62	AAI-based (high-confidence)
NODE_9150_length_1898_cov_0.823235	1898	No	5	1	Low-quality	5.32	AAI-based (high-confidence)
NODE_9151_length_1909_cov_0.090055	1909	No	3	1	Low-quality	5.65	AAI-based (high-confidence)
NODE_9152_length_1909_cov_0.087293	1909	No	5	1	Low-quality	3.05	AAI-based (high-confidence)
NODE_916_length_1679_cov_0.091139	1679	No	2	1	Low-quality	4.53	AAI-based (high-confidence)
NODE_9163_length_1702_cov_0.147224	1702	No	5	3	Low-quality	3.15	AAI-based (high-confidence)
NODE_9167_length_1896_cov_1.111297	1896	No	4	2	Low-quality	5.54	AAI-based (high-confidence)
NODE_9177_length_1700_cov_0.572142	1700	No	2	2	Low-quality	5.17	AAI-based (high-confidence)
NODE_918_length_1678_cov_0.124763	1678	No	2	1	Low-quality	2.82	AAI-based (high-confidence)
NODE_9184_length_1739_cov_0.114634	1739	No	2	2	Low-quality	2.47	AAI-based (high-confidence)
NODE_9184_length_1905_cov_0.104651	1905	No	5	3	Low-quality	5.49	AAI-based (high-confidence)
NODE_9189_length_1904_cov_0.187812	1904	No	3	1	Low-quality	7.79	AAI-based (high-confidence)
NODE_919_length_7256_cov_0.137069	7256	No	8	4	Low-quality	19.74	AAI-based (high-confidence)
NODE_9195_length_1075_cov_0.069672	1075	No	1	1	Low-quality	15.53	AAI-based (high-confidence)

NODE_92_length_3259_cov_0.215823	3259	No	1	1	Low-quality	45.33	AAI-based (high-confidence)
NODE_920_length_2945_cov_0.192902	2945	No	1	1	Low-quality	9.38	AAI-based (high-confidence)
NODE_9222_length_1734_cov_0.456881	1734	No	2	1	Low-quality	2.86	AAI-based (high-confidence)
NODE_9224_length_1695_cov_0.478070	1695	No	2	1	Low-quality	3.63	AAI-based (high-confidence)
NODE_9225_length_1695_cov_0.280702	1695	No	2	1	Low-quality	2.69	AAI-based (high-confidence)
NODE_924_length_5590_cov_0.192679	5590	No	7	6	Low-quality	3.13	AAI-based (high-confidence)
NODE_9240_length_1887_cov_0.163311	1887	No	3	1	Low-quality	4.04	AAI-based (high-confidence)
NODE_9246_length_1886_cov_0.436486	1886	No	5	1	Low-quality	5.05	AAI-based (high-confidence)
NODE_925_length_1491_cov_0.110632	1491	No	4	1	Low-quality	4.07	AAI-based (high-confidence)
NODE_9251_length_1895_cov_0.117483	1895	No	4	1	Low-quality	3.53	AAI-based (high-confidence)
NODE_926_length_1456_cov_0.058217	1456	No	2	1	Low-quality	20.17	AAI-based (high-confidence)
NODE_926_length_6231_cov_0.257828	6231	No	10	2	Low-quality	17.57	AAI-based (high-confidence)
NODE_927_length_1672_cov_0.097902	1672	No	3	1	Low-quality	4	AAI-based (high-confidence)
NODE_929_length_7195_cov_0.215614	7195	No	7	4	Low-quality	13.21	AAI-based (high-confidence)
NODE_9292_length_1069_cov_0.195876	1069	No	2	2	Low-quality	2.06	AAI-based (high-confidence)
NODE_9309_length_1722_cov_0.133703	1722	No	4	1	Low-quality	5.63	AAI-based (high-confidence)
NODE_9311_length_1117_cov_0.053045	1117	No	1	1	Low-quality	2.05	AAI-based (high-confidence)
NODE_9320_length_1877_cov_0.271091	1877	No	5	1	Low-quality	3.83	AAI-based (high-confidence)
NODE_933_length_1438_cov_0.097087	1438	No	3	1	Low-quality	4.39	AAI-based (high-confidence)
NODE_9330_length_1719_cov_0.191975	1719	No	4	2	Low-quality	2.73	AAI-based (high-confidence)
NODE_9338_length_1115_cov_0.046260	1115	No	3	1	Low-quality	2.78	AAI-based (high-confidence)
NODE_934_length_1438_cov_0.081404	1438	No	2	2	Low-quality	4.38	AAI-based (high-confidence)
NODE_934_length_4070_cov_0.279275	4070	No	5	2	Low-quality	6.72	AAI-based (high-confidence)
NODE_9357_length_1067_cov_0.055785	1067	No	3	1	Low-quality	2.04	AAI-based (high-confidence)
NODE_936_length_1622_cov_0.151018	1622	No	2	1	Low-quality	5.47	AAI-based (high-confidence)
NODE_936_length_7117_cov_0.160017	7117	No	9	5	Low-quality	8.79	AAI-based (high-confidence)
NODE_9362_length_1872_cov_0.249295	1872	No	4	2	Low-quality	2.9	AAI-based (high-confidence)
NODE_9368_length_1112_cov_0.094768	1112	No	2	1	Low-quality	3.02	AAI-based (high-confidence)
NODE_937_length_4067_cov_0.147177	4067	No	4	3	Low-quality	6.38	AAI-based (high-confidence)
NODE_9370_length_1872_cov_0.081782	1872	No	4	2	Low-quality	5.66	AAI-based (high-confidence)
NODE_9375_length_1112_cov_0.070089	1112	No	2	1	Low-quality	2.27	AAI-based (high-confidence)
NODE_9378_length_1871_cov_0.110045	1871	No	1	1	Low-quality	4.24	AAI-based (high-confidence)
NODE_938_length_1437_cov_0.033632	1437	No	3	2	Low-quality	2.34	AAI-based (high-confidence)
NODE_938_length_2848_cov_0.173154	2848	No	6	1	Low-quality	4.66	AAI-based (high-confidence)
NODE_939_length_4065_cov_0.203227	4065	No	6	1	Low-quality	6.87	AAI-based (high-confidence)
NODE_9391_length_1679_cov_0.075316	1679	No	2	2	Low-quality	3.79	AAI-based (high-confidence)
NODE_94_length_20781_cov_0.208104	20781	No	28	4	Low-quality	25.84	AAI-based (high-confidence)
NODE_940_length_1482_cov_0.065799	1482	No	1	1	Low-quality	31.49	AAI-based (high-confidence)
NODE_9402_length_1711_cov_0.299007	1711	No	1	1	Low-quality	2.95	AAI-based (high-confidence)
NODE_9404_length_1868_cov_0.447145	1868	No	3	1	Low-quality	1.93	AAI-based (high-confidence)
NODE_941_length_2844_cov_0.176685	2844	No	6	3	Low-quality	8.51	AAI-based (high-confidence)
NODE_9414_length_1877_cov_0.130484	1877	No	1	1	Low-quality	5.22	AAI-based (high-confidence)
NODE_9417_length_1064_cov_0.045596	1064	No	2	2	Low-quality	1.97	AAI-based (high-confidence)
NODE_9426_length_1676_cov_0.124287	1676	No	3	2	Low-quality	4.97	AAI-based (high-confidence)
NODE_943_length_4048_cov_0.118005	4048	No	5	4	Low-quality	8.89	AAI-based (high-confidence)
NODE_9431_length_1108_cov_0.079286	1108	No	3	2	Low-quality	3.11	AAI-based (high-confidence)

NODE_9437_length_1675_cov_0.106599	1675	No	2	1	Low-quality	1.96	AAI-based (high-confidence)
NODE_9439_length_1874_cov_0.220282	1874	No	2	1	Low-quality	4.28	AAI-based (high-confidence)
NODE_9443_length_1674_cov_0.217778	1674	No	4	1	Low-quality	2.85	AAI-based (high-confidence)
NODE_9446_length_1873_cov_0.178692	1873	No	7	3	Low-quality	3.11	AAI-based (high-confidence)
NODE_945_length_1432_cov_0.093023	1432	No	4	2	Low-quality	2.62	AAI-based (high-confidence)
NODE_946_length_2840_cov_0.218169	2840	No	3	1	Low-quality	8.18	AAI-based (high-confidence)
NODE_946_length_6151_cov_0.445968	6151	No	6	2	Low-quality	9.47	AAI-based (high-confidence)
NODE_9468_length_1105_cov_0.154076	1105	No	3	1	Low-quality	2.87	AAI-based (high-confidence)
NODE_948_length_7074_cov_0.321434	7074	No	10	7	Low-quality	6.76	AAI-based (high-confidence)
NODE_9485_length_1104_cov_0.114428	1104	No	1	1	Low-quality	2.83	AAI-based (high-confidence)
NODE_9487_length_1869_cov_1.058192	1869	No	3	1	Low-quality	5.24	AAI-based (high-confidence)
NODE_9488_length_1700_cov_0.157402	1700	No	4	1	Low-quality	4.96	AAI-based (high-confidence)
NODE_949_length_1367_cov_0.070978	1367	No	1	1	Low-quality	2.93	AAI-based (high-confidence)
NODE_95_length_3729_cov_0.139118	3729	No	1	1	Low-quality	28.36	AAI-based (high-confidence)
NODE_9507_length_1867_cov_0.432127	1867	No	1	1	Low-quality	4.53	AAI-based (high-confidence)
NODE_951_length_1429_cov_0.121053	1429	No	1	1	Low-quality	26.52	AAI-based (high-confidence)
NODE_9519_length_1937_cov_0.077258	1937	No	2	1	Low-quality	6	AAI-based (high-confidence)
NODE_952_length_1439_cov_0.047761	1439	No	3	3	Low-quality	3.69	AAI-based (high-confidence)
NODE_952_length_1614_cov_0.106931	1614	No	1	1	Low-quality	24.58	AAI-based (high-confidence)
NODE_953_length_1365_cov_0.062401	1365	No	3	1	Low-quality	4.35	AAI-based (high-confidence)
NODE_9530_length_1666_cov_0.405233	1666	No	4	1	Low-quality	3.9	AAI-based (high-confidence)
NODE_9532_length_1934_cov_1.517711	1934	No	2	1	Low-quality	5.98	AAI-based (high-confidence)
NODE_9537_length_1665_cov_0.147510	1665	No	3	2	Low-quality	2.65	AAI-based (high-confidence)
NODE_9538_length_1853_cov_0.108324	1853	No	3	2	Low-quality	3.66	AAI-based (high-confidence)
NODE_954_length_4025_cov_0.501019	4025	No	8	1	Low-quality	11.61	AAI-based (high-confidence)
NODE_9547_length_1693_cov_0.098494	1693	No	2	2	Low-quality	4.74	AAI-based (high-confidence)
NODE_956_length_1363_cov_0.112342	1363	No	1	1	Low-quality	2.25	AAI-based (high-confidence)
NODE_958_length_2888_cov_0.184295	2888	No	3	1	Low-quality	4.47	AAI-based (high-confidence)
NODE_958_length_5482_cov_0.155675	5482	No	8	2	Low-quality	2.47	AAI-based (high-confidence)
NODE_9580_length_1056_cov_0.027168	1056	No	1	1	Low-quality	2.81	AAI-based (high-confidence)
NODE_9584_length_1097_cov_0.078156	1097	No	2	1	Low-quality	2.46	AAI-based (high-confidence)
NODE_959_length_2887_cov_0.102941	2887	No	6	3	Low-quality	6.53	AAI-based (high-confidence)
NODE_959_length_4020_cov_0.216526	4020	No	6	3	Low-quality	6.65	AAI-based (high-confidence)
NODE_9590_length_1846_cov_0.922152	1846	No	4	2	Low-quality	5.6	AAI-based (high-confidence)
NODE_9591_length_1661_cov_0.194622	1661	No	3	1	Low-quality	5.08	AAI-based (high-confidence)
NODE_96_length_18775_cov_0.189387	18775	No	15	2	Low-quality	19.07	AAI-based (high-confidence)
NODE_96_length_3476_cov_0.133847	3476	No	4	3	Low-quality	6.96	AAI-based (high-confidence)
NODE_960_length_6113_cov_0.329731	6113	No	5	4	Low-quality	3.42	AAI-based (high-confidence)
NODE_9602_length_1660_cov_0.518898	1660	No	3	2	Low-quality	5.13	AAI-based (high-confidence)
NODE_9604_length_1095_cov_0.221888	1095	No	4	1	Low-quality	3.46	AAI-based (high-confidence)
NODE_961_length_1669_cov_0.084713	1669	No	2	1	Low-quality	4.66	AAI-based (high-confidence)
NODE_961_length_6993_cov_0.335219	6993	No	12	3	Low-quality	22.16	AAI-based (high-confidence)
NODE_9618_length_1921_cov_0.065862	1921	No	3	1	Low-quality	7.84	AAI-based (high-confidence)
NODE_9620_length_1659_cov_0.110256	1659	No	2	1	Low-quality	3.13	AAI-based (high-confidence)
NODE_9637_length_1852_cov_0.107815	1852	No	3	2	Low-quality	4.88	AAI-based (high-confidence)
NODE_9659_length_1916_cov_0.102367	1916	No	3	2	Low-quality	4.63	AAI-based (high-confidence)

NODE_966_length_6094_cov_0.615680	6094	No	6	3	Low-quality	18.55	AAI-based (high-confidence)
NODE_9671_length_1090_cov_0.146317	1090	No	4	2	Low-quality	2.35	AAI-based (high-confidence)
NODE_9673_length_1090_cov_0.118063	1090	No	3	1	Low-quality	3.35	AAI-based (high-confidence)
NODE_9679_length_1835_cov_0.266129	1835	No	5	1	Low-quality	5.74	AAI-based (high-confidence)
NODE_968_length_4008_cov_0.119212	4008	No	5	3	Low-quality	9.64	AAI-based (high-confidence)
NODE_968_length_5459_cov_0.221642	5459	No	11	6	Low-quality	9.25	AAI-based (high-confidence)
NODE_9682_length_1654_cov_0.078457	1654	No	5	1	Low-quality	2.7	AAI-based (high-confidence)
NODE_969_length_6089_cov_0.162437	6089	No	6	5	Low-quality	2.84	AAI-based (high-confidence)
NODE_9695_length_1846_cov_0.401259	1846	No	3	1	Low-quality	4.97	AAI-based (high-confidence)
NODE_97_length_3208_cov_0.142168	3208	No	4	1	Low-quality	4.9	AAI-based (high-confidence)
NODE_970_length_6088_cov_0.508432	6088	No	10	6	Low-quality	5.85	AAI-based (high-confidence)
NODE_9703_length_1088_cov_0.116279	1088	No	2	1	Low-quality	3.07	AAI-based (high-confidence)
NODE_9704_length_1651_cov_0.164304	1651	No	2	2	Low-quality	3.07	AAI-based (high-confidence)
NODE_9708_length_1909_cov_0.185083	1909	No	3	1	Low-quality	5.19	AAI-based (high-confidence)
NODE_973_length_2806_cov_0.219431	2806	No	3	1	Low-quality	4.63	AAI-based (high-confidence)
NODE_973_length_6936_cov_0.403978	6936	No	10	5	Low-quality	6.44	AAI-based (high-confidence)
NODE_9732_length_1086_cov_0.050659	1086	No	2	2	Low-quality	2.02	AAI-based (high-confidence)
NODE_974_length_1417_cov_0.125190	1417	No	1	1	Low-quality	21.96	AAI-based (high-confidence)
NODE_9743_length_1647_cov_0.147933	1647	No	5	1	Low-quality	4.92	AAI-based (high-confidence)
NODE_975_length_6931_cov_0.170082	6931	No	9	6	Low-quality	17.03	AAI-based (high-confidence)
NODE_9754_length_1646_cov_0.358759	1646	No	2	1	Low-quality	2.58	AAI-based (high-confidence)
NODE_976_length_6903_cov_0.501323	6903	No	9	4	Low-quality	11.2	AAI-based (high-confidence)
NODE_9768_length_1047_cov_0.082278	1047	No	4	1	Low-quality	1.93	AAI-based (high-confidence)
NODE_977_length_3982_cov_0.430337	3982	No	4	1	Low-quality	12.23	AAI-based (high-confidence)
NODE_9776_length_1822_cov_0.611724	1822	No	6	3	Low-quality	2.87	AAI-based (high-confidence)
NODE_9780_length_1896_cov_0.063996	1896	No	2	1	Low-quality	3.15	AAI-based (high-confidence)
NODE_9791_length_1893_cov_3.117057	1893	No	4	1	Low-quality	5.63	AAI-based (high-confidence)
NODE_9792_length_1821_cov_0.200348	1821	No	3	2	Low-quality	4.66	AAI-based (high-confidence)
NODE_98_length_20076_cov_0.317465	20076	No	27	13	Low-quality	49.56	AAI-based (high-confidence)
NODE_98_length_7318_cov_0.267073	7318	No	5	5	Low-quality	12.1	AAI-based (high-confidence)
NODE_9806_length_1834_cov_0.097406	1834	No	2	2	Low-quality	3.5	AAI-based (high-confidence)
NODE_981_length_1635_cov_0.054036	1635	No	4	2	Low-quality	4.53	AAI-based (high-confidence)
NODE_9815_length_1642_cov_0.082955	1642	No	7	1	Low-quality	2.55	AAI-based (high-confidence)
NODE_9815_length_1832_cov_0.326024	1832	No	3	1	Low-quality	5.68	AAI-based (high-confidence)
NODE_9827_length_1818_cov_0.075625	1818	No	2	2	Low-quality	4.02	AAI-based (high-confidence)
NODE_983_length_3961_cov_0.147851	3961	No	11	1	Low-quality	12.11	AAI-based (high-confidence)
NODE_984_length_1457_cov_0.108247	1457	No	3	1	Low-quality	2.94	AAI-based (high-confidence)
NODE_984_length_6871_cov_0.153426	6871	No	11	5	Low-quality	3.65	AAI-based (high-confidence)
NODE_9845_length_1815_cov_0.167832	1815	No	6	3	Low-quality	3.24	AAI-based (high-confidence)
NODE_985_length_1655_cov_0.070051	1655	No	1	1	Low-quality	32.95	AAI-based (high-confidence)
NODE_9854_length_1814_cov_0.324198	1814	No	4	1	Low-quality	5.46	AAI-based (high-confidence)
NODE_9858_length_1043_cov_0.072034	1043	No	1	1	Low-quality	2.34	AAI-based (high-confidence)
NODE_9859_length_1813_cov_0.261960	1813	No	2	2	Low-quality	3.32	AAI-based (high-confidence)
NODE_986_length_2846_cov_0.275573	2846	No	4	1	Low-quality	4.77	AAI-based (high-confidence)
NODE_986_length_3959_cov_0.119171	3959	No	5	2	Low-quality	4.7	AAI-based (high-confidence)
NODE_9862_length_1813_cov_0.072929	1813	No	4	3	Low-quality	3.65	AAI-based (high-confidence)

NODE_988_length_1593_cov_0.117805	1593	No	1	1	Low-quality	31.58	AAI-based (high-confidence)
NODE_988_length_6019_cov_0.158108	6019	No	4	4	Low-quality	9.83	AAI-based (high-confidence)
NODE_9889_length_1823_cov_0.512761	1823	No	3	3	Low-quality	3.48	AAI-based (high-confidence)
NODE_9897_length_1822_cov_0.477075	1822	No	3	2	Low-quality	3.73	AAI-based (high-confidence)
NODE_990_length_6844_cov_0.415271	6844	No	8	7	Low-quality	8.18	AAI-based (high-confidence)
NODE_9904_length_1651_cov_0.148840	1651	No	2	1	Low-quality	4.42	AAI-based (high-confidence)
NODE_9908_length_1822_cov_0.091701	1822	No	3	1	Low-quality	4.93	AAI-based (high-confidence)
NODE_991_length_5393_cov_0.302607	5393	No	12	4	Low-quality	11.84	AAI-based (high-confidence)
NODE_9914_length_1807_cov_0.450234	1807	No	1	1	Low-quality	5.61	AAI-based (high-confidence)
NODE_9918_length_1807_cov_0.121194	1807	No	1	1	Low-quality	5.6	AAI-based (high-confidence)
NODE_992_length_1589_cov_0.170470	1589	No	3	1	Low-quality	2.9	AAI-based (high-confidence)
NODE_9921_length_1649_cov_0.192903	1649	No	4	1	Low-quality	2.68	AAI-based (high-confidence)
NODE_9927_length_1872_cov_0.152848	1872	No	4	2	Low-quality	4.83	AAI-based (high-confidence)
NODE_9932_length_1819_cov_0.066860	1819	No	5	3	Low-quality	2.3	AAI-based (high-confidence)
NODE_994_length_5386_cov_0.248156	5386	No	12	4	Low-quality	11.11	AAI-based (high-confidence)
NODE_9941_length_1818_cov_0.107621	1818	No	7	1	Low-quality	4.97	AAI-based (high-confidence)
NODE_9942_length_1631_cov_0.082898	1631	No	4	2	Low-quality	2.96	AAI-based (high-confidence)
NODE_9943_length_1631_cov_0.067885	1631	No	3	2	Low-quality	4.55	AAI-based (high-confidence)
NODE_995_length_2835_cov_0.205409	2835	No	5	4	Low-quality	2.56	AAI-based (high-confidence)
NODE_995_length_5378_cov_0.689146	5378	No	6	2	Low-quality	15	AAI-based (high-confidence)
NODE_9953_length_1803_cov_0.041080	1803	No	2	1	Low-quality	3.95	AAI-based (high-confidence)
NODE_996_length_5986_cov_0.191269	5986	No	6	6	Low-quality	3.42	AAI-based (high-confidence)
NODE_9965_length_1644_cov_0.190939	1644	No	4	2	Low-quality	4.95	AAI-based (high-confidence)
NODE_9969_length_1628_cov_0.201439	1628	No	3	1	Low-quality	3.77	AAI-based (high-confidence)
NODE_9971_length_1644_cov_0.093204	1644	No	3	1	Low-quality	4.12	AAI-based (high-confidence)
NODE_998_length_6814_cov_0.195681	6814	No	9	1	Low-quality	6.85	AAI-based (high-confidence)
NODE_9986_length_1642_cov_0.088140	1642	No	3	2	Low-quality	2.99	AAI-based (high-confidence)
NODE_999_length_8452_cov_1.007422	8452	No	13	4	Low-quality	26.05	AAI-based (high-confidence)
NODE_9993_length_1626_cov_0.182056	1626	No	2	1	Low-quality	2.56	AAI-based (high-confidence)
NODE_9994_length_1626_cov_0.181401	1626	No	2	2	Low-quality	2.94	AAI-based (high-confidence)
NODE_9997_length_1068_cov_0.230134	1068	No	3	1	Low-quality	2.48	AAI-based (high-confidence)

Table S4. Information of species names of operational taxonomic unit numbers and significant correlation coefficients between bacteria and bacteriophage lineages.

Bacteria				Bacteriophage				Spearman's rank correlation coefficient	*p*-value
Class	Genus	Species	OTU No.	*Family*	Genus	Species	OTU No.		
Actinomycetia	*Rhodoluna*	Genus level	bOTU049	*Ackermannviridae*	Family level	Family level	vOTU01	0.56	0.047
Gammaproteobacteria	*Methylophaga*	Genus level	bOTU032	*Ackermannviridae*	Family level	Family level	vOTU01	0.64	0.019
Alphaproteobacteria	*Sulfitobacter*	*Sulfitobacter profundi*	bOTU060	*Ackermannviridae*	*Serratia* phage	Genus level	vOTU27	0.64	0.018
Alphaproteobacteria	*Sulfitobacter*	Genus level	bOTU054	*Ackermannviridae*	*Serratia* phage	Genus level	vOTU27	0.64	0.018
Gammaproteobacteria	*Vibrio*	Genus level	bOTU057	*Ampullaviridae*	Family level	Family level	vOTU02	0.59	0.034
Gammaproteobacteria	*Colwellia*	Genus level	bOTU017	*Bicaudaviridae*	Family level	Family level	vOTU03	0.57	0.041
Flavobacteriia	*Polaribacter*	*Polaribacter haliotis*	bOTU072	*Bicaudaviridae*	Family level	Family level	vOTU03	0.59	0.033
Flavobacteriia	*Polaribacter*	*Polaribacter staleyi*	bOTU070	*Bicaudaviridae*	Family level	Family level	vOTU03	0.59	0.033
Flavobacteriia	*Winogradskyella*	Genus level	bOTU059	*Bicaudaviridae*	Family level	Family level	vOTU03	0.61	0.027
Gammaproteobacteria	*Paraglaciecola*	*Paraglaciecola polaris*	bOTU074	*Bicaudaviridae*	Family level	Family level	vOTU03	0.63	0.021
Flavobacteriia	*Polaribacter*	Genus level	bOTU041	*Bicaudaviridae*	Family level	Family level	vOTU03	0.63	0.021
Gammaproteobacteria	*Cognaticolwellia*	Genus level	bOTU016	*Bicaudaviridae*	Family level	Family level	vOTU03	0.66	0.014
Gammaproteobacteria	*Cognaticolwellia*	*Cognaticolwellia aestuarii*	bOTU064	*Bicaudaviridae*	Family level	Family level	vOTU03	0.68	0.010
Gammaproteobacteria	*Paraglaciecola*	Genus level	bOTU036	*Bicaudaviridae*	Family level	Family level	vOTU03	0.69	0.010
Flavobacteriia	Class level	Class level	bOTU003	*Bicaudaviridae*	Family level	Family level	vOTU03	0.69	0.009
Flavobacteriia	*Algibacter*	*Algibacter miyuki*	bOTU080	*Bicaudaviridae*	Family level	Family level	vOTU03	0.71	0.006
Flavobacteriia	*Algibacter*	Genus level	bOTU011	*Bicaudaviridae*	Family level	Family level	vOTU03	0.71	0.006
Flavobacteriia	*Lacinutrix*	Genus level	bOTU022	*Bicaudaviridae*	Family level	Family level	vOTU03	0.72	0.006
Flavobacteriia	*Cellulophaga*	Genus level	bOTU015	*Bicaudaviridae*	Family level	Family level	vOTU03	0.73	0.005
Flavobacteriia	*Lacinutrix*	*Lacinutrix algicola*	bOTU067	*Bicaudaviridae*	Family level	Family level	vOTU03	0.80	0.001
Gammaproteobacteria	*Psychrosphaera*	Genus level	bOTU048	*Bicaudaviridae*	Family level	Family level	vOTU03	0.80	0.001
Gammaproteobacteria	*Psychromonas*	Genus level	bOTU047	*Bicaudaviridae*	Family level	Family level	vOTU03	0.59	0.034
Flavobacteriia	*Maribacter*	Genus level	bOTU026	*Bicaudaviridae*	Family level	Family level	vOTU03	0.73	0.005

Deltaproteobacteria	Class level	Class level	bOTU007	*Herelleviridae*	Family level	Family level	vOTU04	0.55	0.050
Gammaproteobacteria	*Pseudomonas*	*Pseudomonas sabulinigri*	bOTU062	*Herelleviridae*	Family level	Family level	vOTU04	0.56	0.046
Alphaproteobacteria	*Magnetospira*	Genus level	bOTU025	*Herelleviridae*	Family level	Family level	vOTU04	0.59	0.032
Gammaproteobacteria	Class level	Class level	bOTU002	*Herelleviridae*	Family level	Family level	vOTU04	0.60	0.030
Alphaproteobacteria	*Amylibacter*	*Amylibacter cionae*	bOTU105	*Herelleviridae*	Family level	Family level	vOTU04	0.61	0.028
Gammaproteobacteria	*Moraxella*	*Moraxella oblonga*	bOTU083	*Herelleviridae*	Family level	Family level	vOTU04	0.61	0.027
Betaproteobacteria	Class level	Class level	bOTU005	*Herelleviridae*	Family level	Family level	vOTU04	0.63	0.023
Gammaproteobacteria	*Thioprofundum*	Genus level	bOTU056	*Herelleviridae*	Family level	Family level	vOTU04	0.63	0.020
Gammaproteobacteria	*Pseudoalteromonas*	*Pseudoalteromonas hodoensis*	bOTU069	*Herelleviridae*	Family level	Family level	vOTU04	0.66	0.014
Gammaproteobacteria	*Pseudoalteromonas*	Genus level	bOTU043	*Herelleviridae*	Family level	Family level	vOTU04	0.66	0.013
Gammaproteobacteria	*Colwellia*	*Colwellia echini*	bOTU066	*Herelleviridae*	Family level	Family level	vOTU04	0.67	0.013
Gammaproteobacteria	*Colwellia*	Genus level	bOTU017	*Herelleviridae*	Family level	Family level	vOTU04	0.67	0.012
Gammaproteobacteria	*Halomonas*	*Halomonas glaciei*	bOTU068	*Herelleviridae*	Family level	Family level	vOTU04	0.88	0.000
Flavobacteriia	*Aquibacter*	Genus level	bOTU013	*Herelleviridae*	*Bacillus* phage	Genus level	vOTU11	0.56	0.046
Deltaproteobacteria	Class level	Class level	bOTU007	*Herelleviridae*	*Bacillus* phage	Genus level	vOTU11	0.57	0.043
Gammaproteobacteria	*Porticoccus*	*Porticoccus hydrocarbonoclasticus*	bOTU082	*Herelleviridae*	*Bacillus* phage	Genus level	vOTU11	0.59	0.033
Gammaproteobacteria	*Porticoccus*	Genus level	bOTU042	*Herelleviridae*	*Bacillus* phage	Genus level	vOTU11	0.59	0.033
Gammaproteobacteria	*Vibrio*	Genus level	bOTU057	*Herelleviridae*	*Bacillus* phage	Genus level	vOTU11	0.59	0.033
Gammaproteobacteria	Class level	Class level	bOTU002	*Herelleviridae*	*Bacillus* phage	Genus level	vOTU11	0.61	0.028
Alphaproteobacteria	*Marinibaculum*	Genus level	bOTU028	*Herelleviridae*	*Bacillus* phage	Genus level	vOTU11	0.61	0.026
Alphaproteobacteria	*Novosphingobium*	Genus level	bOTU034	*Herelleviridae*	*Bacillus* phage	Genus level	vOTU11	0.63	0.020
Betaproteobacteria	Class level	Class level	bOTU005	*Herelleviridae*	*Bacillus* phage	Genus level	vOTU11	0.64	0.020
Gammaproteobacteria	*Sinobacterium*	Genus level	bOTU052	*Herelleviridae*	*Bacillus* phage	Genus level	vOTU11	0.65	0.016
Gammaproteobacteria	*Marinobacterium*	Genus level	bOTU029	*Herelleviridae*	*Bacillus* phage	Genus level	vOTU11	0.66	0.014
Alphaproteobacteria	*Amylibacter*	Genus level	bOTU029	*Herelleviridae*	*Bacillus* phage	Genus level	vOTU11	0.68	0.011
Gammaproteobacteria	*Thiohalobacter*	Genus level	bOTU029	*Herelleviridae*	*Bacillus* phage	Genus level	vOTU11	0.69	0.009
Cytophagia	Class level	Class level	bOTU029	*Herelleviridae*	*Bacillus* phage	Genus level	vOTU11	0.69	0.009
Gammaproteobacteria	*Moraxella*	*Moraxella oblonga*	bOTU029	*Herelleviridae*	*Bacillus* phage	Genus level	vOTU11	0.69	0.009

Gammaproteobacteria	*Moraxella*	Genus level	bOTU029	*Herelleviridae*	*Bacillus* phage	Genus level	vOTU11	0.69	0.009
Gammaproteobacteria	*Maribrevibacterium*	Genus level	bOTU029	*Herelleviridae*	*Bacillus* phage	Genus level	vOTU11	0.70	0.008
Gammaproteobacteria	*Pseudohongiella*	Genus level	bOTU029	*Herelleviridae*	*Bacillus* phage	Genus level	vOTU11	0.71	0.007
Alphaproteobacteria	*Amylibacter*	*Amylibacter cionae*	bOTU029	*Herelleviridae*	*Bacillus* phage	Genus level	vOTU11	0.73	0.005
Gammaproteobacteria	*Sedimenticola*	*Sedimenticola thiotaurini*	bOTU029	*Herelleviridae*	*Bacillus* phage	Genus level	vOTU11	0.75	0.003
Gammaproteobacteria	*Sedimenticola*	Genus level	bOTU029	*Herelleviridae*	*Bacillus* phage	Genus level	vOTU11	0.75	0.003
Gammaproteobacteria	*Eionea*	*Eionea flava*	bOTU029	*Herelleviridae*	*Bacillus* phage	Genus level	vOTU11	0.77	0.002
Gammaproteobacteria	*Eionea*	Genus level	bOTU029	*Herelleviridae*	*Bacillus* phage	Genus level	vOTU11	0.77	0.002
Gammaproteobacteria	*Parahaliea*	Genus level	bOTU029	*Herelleviridae*	*Bacillus* phage	Genus level	vOTU11	0.81	0.001
Alphaproteobacteria	*Ahrensia*	Genus level	bOTU029	*Herelleviridae*	*Bacillus* phage	Genus level	vOTU11	0.83	0.000
Alphaproteobacteria	*Magnetospira*	Genus level	bOTU029	*Herelleviridae*	*Bacillus* phage	Genus level	vOTU11	0.89	0.000
Gammaproteobacteria	*Moraxella*	Genus level	bOTU029	*Herelleviridae*	Family level	Family level	vOTU04	0.61	0.027
Gammaproteobacteria	*Halomonas*	Genus level	bOTU029	*Herelleviridae*	Family level	Family level	vOTU04	0.88	0.000
Acidimicrobiia	*Ilumatobacter*	*Ilumatobacter fluminis*	bOTU029	*Inoviridae*	Family level	Family level	vOTU05	0.57	0.044
Acidimicrobiia	*Ilumatobacter*	Genus level	bOTU029	*Inoviridae*	Family level	Family level	vOTU05	0.57	0.044
Alphaproteobacteria	*Sulfitobacter*	*Sulfitobacter profundi*	bOTU029	*Inoviridae*	Family level	Family level	vOTU05	0.60	0.031
Alphaproteobacteria	*Sulfitobacter*	Genus level	bOTU029	*Inoviridae*	Family level	Family level	vOTU05	0.60	0.031
Alphaproteobacteria	*Amylibacter*	*Amylibacter cionae*	bOTU029	*Microviridae*	Family level	Family level	vOTU06	0.56	0.048
Betaproteobacteria	Class level	Class level	bOTU029	*Microviridae*	Family level	Family level	vOTU06	0.58	0.036
Gammaproteobacteria	*Pseudomonas*	*Pseudomonas stutzeri*	bOTU029	*Microviridae*	Family level	Family level	vOTU06	0.59	0.034
Gammaproteobacteria	*Pseudomonas*	*Pseudomonas chloritidismutans*	bOTU029	*Microviridae*	Family level	Family level	vOTU06	0.60	0.029
Gammaproteobacteria	*Parahaliea*	Genus level	bOTU029	*Microviridae*	Family level	Family level	vOTU06	0.70	0.008
Flavobacteriia	Class level	Class level	bOTU029	*Myoviridae*	*Acinetobacter* phage	Genus level	vOTU08	0.56	0.046
Flavobacteriia	*Maribacter*	Genus level	bOTU029	*Myoviridae*	*Acinetobacter* phage	Genus level	vOTU08	0.62	0.024
Alphaproteobacteria	*Parasphingorhabdus*	*Parasphingorhabdus flavimaris*	bOTU029	*Myoviridae*	*Acinetobacter* phage	Genus level	vOTU08	0.62	0.023
Alphaproteobacteria	*Parasphingorhabdus*	Genus level	bOTU029	*Myoviridae*	*Acinetobacter* phage	Genus level	vOTU08	0.62	0.023
Flavobacteriia	*Aquibacter*	Genus level	bOTU029	*Myoviridae*	*Aeromonas* phage	Genus level	vOTU09	0.57	0.041
Gammaproteobacteria	*Paraglaciecola*	Genus level	bOTU029	*Myoviridae*	*Aeromonas* phage	Genus level	vOTU09	0.57	0.041

Gammaproteobacteria	*Pseudomonas*	*Pseudomonas sabulinigri*	bOTU029	*Myoviridae*	*Aeromonas* phage	Genus level	vOTU09	0.57	0.040
Alphaproteobacteria	*Marinibaculum*	Genus level	bOTU029	*Myoviridae*	*Aeromonas* phage	Genus level	vOTU09	0.60	0.030
Alphaproteobacteria	*Magnetospira*	Genus level	bOTU029	*Myoviridae*	*Aeromonas* phage	Genus level	vOTU09	0.60	0.029
Planctomycetia	Class level	Class level	bOTU029	*Myoviridae*	*Aeromonas* phage	Genus level	vOTU09	0.61	0.026
Cytophagia	*Marinoscillum*	Genus level	bOTU029	*Myoviridae*	*Aeromonas* phage	Genus level	vOTU09	0.62	0.023
Gammaproteobacteria	*Sedimenticola*	*Sedimenticola thiotaurini*	bOTU029	*Myoviridae*	*Aeromonas* phage	Genus level	vOTU09	0.63	0.021
Gammaproteobacteria	*Sedimenticola*	Genus level	bOTU029	*Myoviridae*	*Aeromonas* phage	Genus level	vOTU09	0.63	0.021
Gammaproteobacteria	*Maribrevibacterium*	Genus level	bOTU029	*Myoviridae*	*Aeromonas* phage	Genus level	vOTU09	0.63	0.021
Gammaproteobacteria	Class level	Class level	bOTU029	*Myoviridae*	*Aeromonas* phage	Genus level	vOTU09	0.64	0.020
Gammaproteobacteria	*Paraglaciecola*	*Paraglaciecola polaris*	bOTU029	*Myoviridae*	*Aeromonas* phage	Genus level	vOTU09	0.66	0.015
Deltaproteobacteria	Class level	Class level	bOTU029	*Myoviridae*	*Aeromonas* phage	Genus level	vOTU09	0.67	0.012
Gammaproteobacteria	*Pseudoalteromonas*	*Pseudoalteromonas hodoensis*	bOTU029	*Myoviridae*	*Aeromonas* phage	Genus level	vOTU09	0.68	0.011
Gammaproteobacteria	*Colwellia*	Genus level	bOTU029	*Myoviridae*	*Aeromonas* phage	Genus level	vOTU09	0.68	0.011
Cytophagia	Class level	Class level	bOTU029	*Myoviridae*	*Aeromonas* phage	Genus level	vOTU09	0.69	0.009
Alphaproteobacteria	*Novosphingobium*	Genus level	bOTU029	*Myoviridae*	*Aeromonas* phage	Genus level	vOTU09	0.70	0.008
Gammaproteobacteria	*Colwellia*	*Colwellia echini*	bOTU029	*Myoviridae*	*Aeromonas* phage	Genus level	vOTU09	0.70	0.008
Gammaproteobacteria	*Pseudoalteromonas*	Genus level	bOTU029	*Myoviridae*	*Aeromonas* phage	Genus level	vOTU09	0.71	0.006
Alphaproteobacteria	*Amylibacter*	*Amylibacter cionae*	bOTU029	*Myoviridae*	*Aeromonas* phage	Genus level	vOTU09	0.72	0.006
Betaproteobacteria	Class level	Class level	bOTU029	*Myoviridae*	*Aeromonas* phage	Genus level	vOTU09	0.73	0.004
Gammaproteobacteria	*Pseudohongiella*	Genus level	bOTU029	*Myoviridae*	*Aeromonas* phage	Genus level	vOTU09	0.74	0.004
Flavobacteriia	*Owenweeksia*	Genus level	bOTU029	*Myoviridae*	*Aeromonas* phage	Genus level	vOTU09	0.77	0.002
Alphaproteobacteria	*Amylibacter*	Genus level	bOTU029	*Myoviridae*	*Aeromonas* phage	Genus level	vOTU09	0.78	0.002
Gammaproteobacteria	*Thioprofundum*	Genus level	bOTU029	*Myoviridae*	*Aeromonas* phage	Genus level	vOTU09	0.80	0.001
Gammaproteobacteria	*Moraxella*	*Moraxella oblonga*	bOTU029	*Myoviridae*	*Aeromonas* phage	Genus level	vOTU09	0.81	0.001
Gammaproteobacteria	*Moraxella*	Genus level	bOTU029	*Myoviridae*	*Aeromonas* phage	Genus level	vOTU09	0.81	0.001
Gammaproteobacteria	*Halomonas*	*Halomonas glaciei*	bOTU029	*Myoviridae*	*Aeromonas* phage	Genus level	vOTU09	0.84	0.000
Gammaproteobacteria	*Halomonas*	Genus level	bOTU029	*Myoviridae*	*Aeromonas* phage	Genus level	vOTU09	0.84	0.000
Alphaproteobacteria	*Loktanella*	*Loktanella acticola*	bOTU029	*Myoviridae*	*Agrobacterium* phage	Genus level	vOTU10	0.81	0.001
Alphaproteobacteria	*Loktanella*	Genus level	bOTU029	*Myoviridae*	*Agrobacterium* phage	Genus level	vOTU10	0.81	0.001

Alphaproteobacteria	*Sulfitobacter*	*Sulfitobacter profundi*	bOTU029	*Myoviridae*	*Agrobacterium* phage	Genus level	vOTU10	0.81	0.001
Alphaproteobacteria	*Sulfitobacter*	Genus level	bOTU029	*Myoviridae*	*Agrobacterium* phage	Genus level	vOTU10	0.81	0.001
Alphaproteobacteria	Class level	Class level	bOTU029	*Myoviridae*	*Agrobacterium* phage	Genus level	vOTU10	0.82	0.001
Acidimicrobiia	*Ilumatobacter*	*Ilumatobacter fluminis*	bOTU029	*Myoviridae*	*Agrobacterium* phage	Genus level	vOTU10	0.84	0.000
Acidimicrobiia	*Ilumatobacter*	Genus level	bOTU029	*Myoviridae*	*Agrobacterium* phage	Genus level	vOTU10	0.84	0.000
Gammaproteobacteria	*Pseudoalteromonas*	Genus level	bOTU029	*Myoviridae*	*Campylobacter* phage	Genus level	vOTU12	0.64	0.020
Gammaproteobacteria	*Pseudoalteromonas*	*Pseudoalteromonas hodoensis*	bOTU029	*Myoviridae*	*Campylobacter* phage	Genus level	vOTU12	0.68	0.010
Gammaproteobacteria	*Pseudomonas*	Genus level	bOTU029	*Myoviridae*	*Clostridium* phage	Genus level	vOTU14	0.56	0.045
Alphaproteobacteria	*Parasphingorhabdus*	*Parasphingorhabdus flavimaris*	bOTU029	*Myoviridae*	*Clostridium* phage	Genus level	vOTU14	0.57	0.042
Alphaproteobacteria	*Parasphingorhabdus*	Genus level	bOTU029	*Myoviridae*	*Clostridium* phage	Genus level	vOTU14	0.57	0.042
Alphaproteobacteria	*Paraurantiacibacter*	Genus level	bOTU029	*Myoviridae*	*Clostridium* phage	Genus level	vOTU14	0.73	0.005
Flavobacteriia	*Lacinutrix*	*Lacinutrix algicola*	bOTU029	*Myoviridae*	*Croceibacter* phage	Genus level	vOTU15	0.55	0.049
Gammaproteobacteria	*Halomonas*	*Halomonas glaciei*	bOTU029	*Myoviridae*	*Croceibacter* phage	Genus level	vOTU15	0.56	0.046
Gammaproteobacteria	*Halomonas*	Genus level	bOTU029	*Myoviridae*	*Croceibacter* phage	Genus level	vOTU15	0.56	0.046
Gammaproteobacteria	*Cognaticolwellia*	*Cognaticolwellia aestuarii*	bOTU029	*Myoviridae*	*Croceibacter* phage	Genus level	vOTU15	0.57	0.043
Gammaproteobacteria	*Cognaticolwellia*	Genus level	bOTU029	*Myoviridae*	*Croceibacter* phage	Genus level	vOTU15	0.57	0.042
Gammaproteobacteria	*Psychrobacter*	Genus level	bOTU029	*Myoviridae*	*Croceibacter* phage	Genus level	vOTU15	0.58	0.037
Flavobacteriia	*Leeuwenhoekiella*	Genus level	bOTU029	*Myoviridae*	*Croceibacter* phage	Genus level	vOTU15	0.59	0.034
Gammaproteobacteria	*Psychrobacter*	*Psychrobacter nivimaris*	bOTU029	*Myoviridae*	*Croceibacter* phage	Genus level	vOTU15	0.61	0.026
Gammaproteobacteria	*Thioprofundum*	Genus level	bOTU029	*Myoviridae*	*Croceibacter* phage	Genus level	vOTU15	0.61	0.026
Flavobacteriia	Class level	Class level	bOTU029	*Myoviridae*	*Croceibacter* phage	Genus level	vOTU15	0.63	0.022
Betaproteobacteria	Class level	Class level	bOTU029	*Myoviridae*	*Croceibacter* phage	Genus level	vOTU15	0.64	0.020
Flavobacteriia	*Cellulophaga*	Genus level	bOTU029	*Myoviridae*	*Croceibacter* phage	Genus level	vOTU15	0.64	0.018
Planctomycetia	Class level	Class level	bOTU029	*Myoviridae*	*Croceibacter* phage	Genus level	vOTU15	0.64	0.017
Gammaproteobacteria	*Colwellia*	*Colwellia echini*	bOTU029	*Myoviridae*	*Croceibacter* phage	Genus level	vOTU15	0.65	0.015
Flavobacteriia	*Algibacter*	*Algibacter miyuki*	bOTU029	*Myoviridae*	*Croceibacter* phage	Genus level	vOTU15	0.67	0.013
Flavobacteriia	*Algibacter*	Genus level	bOTU029	*Myoviridae*	*Croceibacter* phage	Genus level	vOTU15	0.67	0.013
Gammaproteobacteria	*Colwellia*	Genus level	bOTU029	*Myoviridae*	*Croceibacter* phage	Genus level	vOTU15	0.68	0.011

Flavobacteriia	*Maribacter*	Genus level	bOTU029	*Myoviridae*	*Croceibacter* phage	Genus level	vOTU15	0.69	0.010
Flavobacteriia	*Winogradskyella*	Genus level	bOTU029	*Myoviridae*	*Croceibacter* phage	Genus level	vOTU15	0.70	0.008
Gammaproteobacteria	*Psychromonas*	Genus level	bOTU029	*Myoviridae*	*Croceibacter* phage	Genus level	vOTU15	0.70	0.008
Flavobacteriia	*Polaribacter*	*Polaribacter staleyi*	bOTU029	*Myoviridae*	*Croceibacter* phage	Genus level	vOTU15	0.72	0.006
Flavobacteriia	*Polaribacter*	Genus level	bOTU029	*Myoviridae*	*Croceibacter* phage	Genus level	vOTU15	0.72	0.006
Flavobacteriia	*Polaribacter*	*Polaribacter haliotis*	bOTU029	*Myoviridae*	*Croceibacter* phage	Genus level	vOTU15	0.75	0.003
Alphaproteobacteria	*Sphingomonas*	Genus level	bOTU029	*Myoviridae*	*Croceibacter* phage	Genus level	vOTU15	0.76	0.003
Alphaproteobacteria	*Paraurantiacibacter*	Genus level	bOTU029	*Myoviridae*	*Croceibacter* phage	Genus level	vOTU15	0.77	0.002
Gammaproteobacteria	*Pseudomonas*	Genus level	bOTU029	*Myoviridae*	*Croceibacter* phage	Genus level	vOTU15	0.79	0.001
Gammaproteobacteria	*Pseudomonas*	*Pseudomonas sabulinigri*	bOTU029	*Myoviridae*	*Croceibacter* phage	Genus level	vOTU15	0.86	0.000
Alphaproteobacteria	*Parasphingorhabdus*	*Parasphingorhabdus flavimaris*	bOTU029	*Myoviridae*	*Croceibacter* phage	Genus level	vOTU15	0.92	0.000
Alphaproteobacteria	*Parasphingorhabdus*	Genus level	bOTU029	*Myoviridae*	*Croceibacter* phage	Genus level	vOTU15	0.92	0.000
Gammaproteobacteria	*Shewanella*	Genus level	bOTU029	*Myoviridae*	*Escherichia* phage	Genus level	vOTU07	0.58	0.037
Gammaproteobacteria	*Psychrosphaera*	Genus level	bOTU029	*Myoviridae*	*Escherichia* phage	Genus level	vOTU07	0.62	0.024
Alphaproteobacteria	*Parasphingorhabdus*	*Parasphingorhabdus flavimaris*	bOTU029	*Myoviridae*	*Escherichia* phage	Genus level	vOTU07	0.65	0.016
Alphaproteobacteria	*Parasphingorhabdus*	Genus level	bOTU029	*Myoviridae*	*Escherichia* phage	Genus level	vOTU07	0.65	0.016
Flavobacteriia	*Polaribacter*	*Polaribacter haliotis*	bOTU029	*Myoviridae*	*Escherichia* phage	Genus level	vOTU07	0.65	0.016
Flavobacteriia	*Algibacter*	*Algibacter miyuki*	bOTU029	*Myoviridae*	*Escherichia* phage	Genus level	vOTU07	0.66	0.014
Flavobacteriia	*Algibacter*	Genus level	bOTU029	*Myoviridae*	*Escherichia* phage	Genus level	vOTU07	0.66	0.014
Flavobacteriia	*Polaribacter*	*Polaribacter staleyi*	bOTU029	*Myoviridae*	*Escherichia* phage	Genus level	vOTU07	0.67	0.013
Flavobacteriia	*Polaribacter*	Genus level	bOTU029	*Myoviridae*	*Escherichia* phage	Genus level	vOTU07	0.67	0.012
Flavobacteriia	*Cellulophaga*	Genus level	bOTU029	*Myoviridae*	*Escherichia* phage	Genus level	vOTU07	0.67	0.012
Flavobacteriia	*Lacinutrix*	Genus level	bOTU029	*Myoviridae*	*Escherichia* phage	Genus level	vOTU07	0.68	0.011
Flavobacteriia	*Winogradskyella*	Genus level	bOTU029	*Myoviridae*	*Escherichia* phage	Genus level	vOTU07	0.68	0.011
Gammaproteobacteria	*Halomonas*	*Halomonas glaciei*	bOTU029	*Myoviridae*	*Escherichia* phage	Genus level	vOTU07	0.69	0.010
Gammaproteobacteria	*Halomonas*	Genus level	bOTU029	*Myoviridae*	*Escherichia* phage	Genus level	vOTU07	0.69	0.010
Flavobacteriia	Class level	Class level	bOTU029	*Myoviridae*	*Escherichia* phage	Genus level	vOTU07	0.69	0.010
Planctomycetia	Class level	Class level	bOTU029	*Myoviridae*	*Escherichia* phage	Genus level	vOTU07	0.70	0.008

Gammaproteobacteria	*Cognaticolwellia*	*Cognaticolwellia aestuarii*	bOTU029	*Myoviridae*	*Escherichia* phage	Genus level	vOTU07	0.74	0.004
Gammaproteobacteria	*Cognaticolwellia*	Genus level	bOTU029	*Myoviridae*	*Escherichia* phage	Genus level	vOTU07	0.74	0.004
Gammaproteobacteria	*Paraglaciecola*	Genus level	bOTU029	*Myoviridae*	*Escherichia* phage	Genus level	vOTU07	0.75	0.003
Gammaproteobacteria	*Pseudomonas*	*Pseudomonas sabulinigri*	bOTU029	*Myoviridae*	*Escherichia* phage	Genus level	vOTU07	0.75	0.003
Gammaproteobacteria	*Colwellia*	*Colwellia echini*	bOTU029	*Myoviridae*	*Escherichia* phage	Genus level	vOTU07	0.77	0.002
Flavobacteriia	*Lacinutrix*	*Lacinutrix algicola*	bOTU029	*Myoviridae*	*Escherichia* phage	Genus level	vOTU07	0.79	0.001
Gammaproteobacteria	*Paraglaciecola*	*Paraglaciecola polaris*	bOTU029	*Myoviridae*	*Escherichia* phage	Genus level	vOTU07	0.79	0.001
Gammaproteobacteria	*Psychromonas*	Genus level	bOTU029	*Myoviridae*	*Escherichia* phage	Genus level	vOTU07	0.81	0.001
Gammaproteobacteria	*Colwellia*	Genus level	bOTU029	*Myoviridae*	*Escherichia* phage	Genus level	vOTU07	0.82	0.001
Flavobacteriia	*Maribacter*	Genus level	bOTU029	*Myoviridae*	*Escherichia* phage	Genus level	vOTU07	0.83	0.000
Gammaproteobacteria	*Methylophaga*	Genus level	bOTU029	*Myoviridae*	*Prochlorococcus* phage	Genus level	vOTU22	0.57	0.041
Actinomycetia	*Rhodoluna*	Genus level	bOTU029	*Myoviridae*	*Prochlorococcus* phage	Genus level	vOTU22	0.63	0.021
Alphaproteobacteria	*Loktanella*	*Loktanella acticola*	bOTU029	*Myoviridae*	*Prochlorococcus* phage	Genus level	vOTU22	0.77	0.002
Alphaproteobacteria	*Loktanella*	Genus level	bOTU029	*Myoviridae*	*Prochlorococcus* phage	Genus level	vOTU22	0.77	0.002
Alphaproteobacteria	*Sulfitobacter*	*Sulfitobacter profundi*	bOTU029	*Myoviridae*	*Prochlorococcus* phage	Genus level	vOTU22	0.78	0.002
Alphaproteobacteria	*Sulfitobacter*	Genus level	bOTU029	*Myoviridae*	*Prochlorococcus* phage	Genus level	vOTU22	0.78	0.002
Acidimicrobiia	*Ilumatobacter*	*Ilumatobacter fluminis*	bOTU029	*Myoviridae*	*Prochlorococcus* phage	Genus level	vOTU22	0.80	0.001
Acidimicrobiia	*Ilumatobacter*	Genus level	bOTU029	*Myoviridae*	*Prochlorococcus* phage	Genus level	vOTU22	0.80	0.001
Acidimicrobiia	*Ilumatobacter*	*Ilumatobacter fluminis*	bOTU029	*Myoviridae*	*Rhizobium* phage	Genus level	vOTU25	0.56	0.048
Acidimicrobiia	*Ilumatobacter*	Genus level	bOTU029	*Myoviridae*	*Rhizobium* phage	Genus level	vOTU25	0.56	0.048
Alphaproteobacteria	Class level	Class level	bOTU029	*Myoviridae*	*Rhizobium* phage	Genus level	vOTU25	0.56	0.045
Alphaproteobacteria	*Sulfitobacter*	*Sulfitobacter profundi*	bOTU029	*Myoviridae*	*Rhizobium* phage	Genus level	vOTU25	0.57	0.044
Alphaproteobacteria	*Sulfitobacter*	Genus level	bOTU029	*Myoviridae*	*Rhizobium* phage	Genus level	vOTU25	0.57	0.044
Alphaproteobacteria	*Loktanella*	*Loktanella acticola*	bOTU029	*Myoviridae*	*Rhizobium* phage	Genus level	vOTU25	0.61	0.026
Alphaproteobacteria	*Loktanella*	Genus level	bOTU029	*Myoviridae*	*Rhizobium* phage	Genus level	vOTU25	0.61	0.026
Cyanophyceae	*Foliisarcina*	Genus level	bOTU029	*Myoviridae*	*Rhizobium* phage	Genus level	vOTU25	0.76	0.003
Actinomycetia	*Rhodoluna*	Genus level	bOTU029	*Myoviridae*	*Rhizobium* phage	Genus level	vOTU25	0.78	0.002
Gammaproteobacteria	*Halomonas*	*Halomonas glaciei*	bOTU029	*Myoviridae*	*Sphingomonas* phage	Genus level	vOTU28	0.56	0.049
Gammaproteobacteria	*Halomonas*	Genus level	bOTU029	*Myoviridae*	*Sphingomonas* phage	Genus level	vOTU28	0.56	0.049

Flavobacteriia	*Owenweeksia*	Genus level	bOTU029	*Myoviridae*	*Sphingomonas* phage	Genus level	vOTU28	0.56	0.047
Gammaproteobacteria	*Pseudomonas*	*Pseudomonas stutzeri*	bOTU029	*Myoviridae*	*Sphingomonas* phage	Genus level	vOTU28	0.56	0.047
Flavobacteriia	*Cellulophaga*	Genus level	bOTU029	*Myoviridae*	*Sphingomonas* phage	Genus level	vOTU28	0.56	0.047
Flavobacteriia	*Polaribacter*	*Polaribacter staleyi*	bOTU029	*Myoviridae*	*Sphingomonas* phage	Genus level	vOTU28	0.56	0.045
Alphaproteobacteria	*Sphingomonas*	Genus level	bOTU029	*Myoviridae*	*Sphingomonas* phage	Genus level	vOTU28	0.58	0.039
Flavobacteriia	*Algibacter*	*Algibacter miyuki*	bOTU029	*Myoviridae*	*Sphingomonas* phage	Genus level	vOTU28	0.61	0.028
Flavobacteriia	*Algibacter*	Genus level	bOTU029	*Myoviridae*	*Sphingomonas* phage	Genus level	vOTU28	0.61	0.028
Flavobacteriia	*Polaribacter*	Genus level	bOTU029	*Myoviridae*	*Sphingomonas* phage	Genus level	vOTU28	0.61	0.028
Gammaproteobacteria	*Thioprofundum*	Genus level	bOTU029	*Myoviridae*	*Sphingomonas* phage	Genus level	vOTU28	0.62	0.025
Gammaproteobacteria	*Pseudomonas*	*Pseudomonas sabulinigri*	bOTU029	*Myoviridae*	*Sphingomonas* phage	Genus level	vOTU28	0.64	0.018
Gammaproteobacteria	*Psychrobacter*	*Psychrobacter submarinus*	bOTU029	*Myoviridae*	*Sphingomonas* phage	Genus level	vOTU28	0.64	0.018
Flavobacteriia	*Maribacter*	Genus level	bOTU029	*Myoviridae*	*Sphingomonas* phage	Genus level	vOTU28	0.64	0.018
Betaproteobacteria	Class level	Class level	bOTU029	*Myoviridae*	*Sphingomonas* phage	Genus level	vOTU28	0.64	0.018
Flavobacteriia	*Polaribacter*	*Polaribacter haliotis*	bOTU029	*Myoviridae*	*Sphingomonas* phage	Genus level	vOTU28	0.66	0.015
Gammaproteobacteria	*Psychrobacter*	Genus level	bOTU029	*Myoviridae*	*Sphingomonas* phage	Genus level	vOTU28	0.66	0.015
Alphaproteobacteria	*Parasphingorhabdus*	*Parasphingorhabdus flavimaris*	bOTU029	*Myoviridae*	*Sphingomonas* phage	Genus level	vOTU28	0.69	0.009
Alphaproteobacteria	*Parasphingorhabdus*	Genus level	bOTU029	*Myoviridae*	*Sphingomonas* phage	Genus level	vOTU28	0.69	0.009
Gammaproteobacteria	*Pseudomonas*	*Pseudomonas chloritidismutans*	bOTU029	*Myoviridae*	*Sphingomonas* phage	Genus level	vOTU28	0.69	0.009
Gammaproteobacteria	*Pseudomonas*	Genus level	bOTU029	*Myoviridae*	*Sphingomonas* phage	Genus level	vOTU28	0.73	0.005
Gammaproteobacteria	*Psychrobacter*	*Psychrobacter nivimaris*	bOTU029	*Myoviridae*	*Sphingomonas* phage	Genus level	vOTU28	0.78	0.002
Gammaproteobacteria	*Psychrobacter*	*Psychrobacter submarinus*	bOTU029	*Myoviridae*		Sphingomonas phage PAU	vOTU40	0.64	0.018
Acidimicrobiia	*Ilumatobacter*	*Ilumatobacter fluminis*	bOTU029	*Myoviridae*	*Staphylococcus* phage	Genus level	vOTU29	0.56	0.048
Acidimicrobiia	*Ilumatobacter*	Genus level	bOTU029	*Myoviridae*	*Staphylococcus* phage	Genus level	vOTU29	0.56	0.048
Alphaproteobacteria	Class level	Class level	bOTU029	*Myoviridae*	*Staphylococcus* phage	Genus level	vOTU29	0.56	0.045
Alphaproteobacteria	*Sulfitobacter*	*Sulfitobacter profundi*	bOTU029	*Myoviridae*	*Staphylococcus* phage	Genus level	vOTU29	0.57	0.044
Alphaproteobacteria	*Sulfitobacter*	Genus level	bOTU029	*Myoviridae*	*Staphylococcus* phage	Genus level	vOTU29	0.57	0.044
Alphaproteobacteria	*Loktanella*	*Loktanella acticola*	bOTU029	*Myoviridae*	*Staphylococcus* phage	Genus level	vOTU29	0.61	0.026
Alphaproteobacteria	*Loktanella*	Genus level	bOTU029	*Myoviridae*	*Staphylococcus* phage	Genus level	vOTU29	0.61	0.026

Cyanophyceae	*Foliisarcina*	Genus level	bOTU029	*Myoviridae*	*Staphylococcus* phage	Genus level	vOTU29	0.76	0.003
Actinomycetia	*Rhodoluna*	Genus level	bOTU029	*Myoviridae*	*Staphylococcus* phage	Genus level	vOTU29	0.78	0.002
Alphaproteobacteria	*Loktanella*	*Loktanella acticola*	bOTU029	*Myoviridae*	*Synechococcus* phage	Genus level	vOTU30	0.60	0.030
Alphaproteobacteria	*Loktanella*	Genus level	bOTU029	*Myoviridae*	*Synechococcus* phage	Genus level	vOTU30	0.60	0.030
Gammaproteobacteria	*Colwellia*	Genus level	bOTU029	*Myoviridae*	*Thermus* phage	Genus level	vOTU31	0.56	0.046
Gammaproteobacteria	*Pseudoalteromonas*	*Pseudoalteromonas hodoensis*	bOTU029	*Myoviridae*	*Thermus* phage	Genus level	vOTU31	0.59	0.035
Gammaproteobacteria	*Pseudoalteromonas*	Genus level	bOTU029	*Myoviridae*	*Thermus* phage	Genus level	vOTU31	0.60	0.032
Gammaproteobacteria	*Shewanella*	Genus level	bOTU029	*Myoviridae*	*Thermus* phage	Genus level	vOTU31	0.60	0.029
Gammaproteobacteria	*Colwellia*	*Colwellia echini*	bOTU029	*Myoviridae*	*Thermus* phage	Genus level	vOTU31	0.61	0.027
Gammaproteobacteria	*Halomonas*	*Halomonas glaciei*	bOTU029	*Myoviridae*	*Thermus* phage	Genus level	vOTU31	0.69	0.010
Gammaproteobacteria	*Halomonas*	Genus level	bOTU029	*Myoviridae*	*Thermus* phage	Genus level	vOTU31	0.69	0.010
Gammaproteobacteria	*Cognaticolwellia*	*Cognaticolwellia aestuarii*	bOTU029	*Myoviridae*	*Vibrio* phage	Genus level	vOTU32	0.56	0.045
Gammaproteobacteria	*Pseudomonas*	*Pseudomonas sabulinigri*	bOTU029	*Myoviridae*	*Vibrio* phage	Genus level	vOTU32	0.56	0.045
Flavobacteriia	*Polaribacter*	*Polaribacter staleyi*	bOTU029	*Myoviridae*	*Vibrio* phage	Genus level	vOTU32	0.59	0.034
Flavobacteriia	*Polaribacter*	Genus level	bOTU029	*Myoviridae*	*Vibrio* phage	Genus level	vOTU32	0.59	0.033
Gammaproteobacteria	*Paraglaciecola*	Genus level	bOTU029	*Myoviridae*	*Vibrio* phage	Genus level	vOTU32	0.60	0.030
Gammaproteobacteria	*Psychrosphaera*	Genus level	bOTU029	*Myoviridae*	*Vibrio* phage	Genus level	vOTU32	0.61	0.027
Flavobacteriia	*Polaribacter*	*Polaribacter haliotis*	bOTU029	*Myoviridae*	*Vibrio* phage	Genus level	vOTU32	0.61	0.026
Flavobacteriia	*Lacinutrix*	*Lacinutrix algicola*	bOTU029	*Myoviridae*	*Vibrio* phage	Genus level	vOTU32	0.63	0.022
Gammaproteobacteria	*Psychromonas*	Genus level	bOTU029	*Myoviridae*	*Vibrio* phage	Genus level	vOTU32	0.63	0.022
Flavobacteriia	*Algibacter*	*Algibacter miyuki*	bOTU029	*Myoviridae*	*Vibrio* phage	Genus level	vOTU32	0.65	0.017
Flavobacteriia	*Algibacter*	Genus level	bOTU029	*Myoviridae*	*Vibrio* phage	Genus level	vOTU32	0.65	0.017
Flavobacteriia	*Cellulophaga*	Genus level	bOTU029	*Myoviridae*	*Vibrio* phage	Genus level	vOTU32	0.66	0.014
Flavobacteriia	*Maribacter*	Genus level	bOTU029	*Myoviridae*	*Vibrio* phage	Genus level	vOTU32	0.81	0.001
Gammaproteobacteria	*Pseudomonas*	*Pseudomonas sabulinigri*	bOTU029	*Myoviridae*	*Yersinia* phage	Genus level	vOTU33	0.56	0.048
Flavobacteriia	Class level	Class level	bOTU029	*Myoviridae*	*Yersinia* phage	Genus level	vOTU33	0.57	0.044
Alphaproteobacteria	*Ahrensia*	Genus level	bOTU029	*Myoviridae*	*Yersinia* phage	Genus level	vOTU33	0.57	0.043
Gammaproteobacteria	*Pseudoalteromonas*	*Pseudoalteromonas hodoensis*	bOTU029	*Myoviridae*	*Yersinia* phage	Genus level	vOTU33	0.59	0.035

Flavobacteriia	*Mesoflavibacter*	Genus level	bOTU029	*Myoviridae*	*Yersinia* phage	Genus level	vOTU33	0.59	0.034
Gammaproteobacteria	*Colwellia*	*Colwellia echini*	bOTU029	*Myoviridae*	*Yersinia* phage	Genus level	vOTU33	0.60	0.031
Gammaproteobacteria	*Sinobacterium*	Genus level	bOTU029	*Myoviridae*	*Yersinia* phage	Genus level	vOTU33	0.60	0.031
Flavobacteriia	*Polaribacter*	*Polaribacter haliotis*	bOTU029	*Myoviridae*	*Yersinia* phage	Genus level	vOTU33	0.61	0.028
Alphaproteobacteria	*Magnetospira*	Genus level	bOTU029	*Myoviridae*	*Yersinia* phage	Genus level	vOTU33	0.61	0.026
Flavobacteriia	*Vicingus*	*Vicingus serpentipes*	bOTU029	*Myoviridae*	*Yersinia* phage	Genus level	vOTU33	0.61	0.026
Gammaproteobacteria	*Colwellia*	Genus level	bOTU029	*Myoviridae*	*Yersinia* phage	Genus level	vOTU33	0.61	0.026
Vicingus	Class level	Class level	bOTU029	*Myoviridae*	*Yersinia* phage	Genus level	vOTU33	0.61	0.026
Gammaproteobacteria	*Pseudoalteromonas*	Genus level	bOTU029	*Myoviridae*	*Yersinia* phage	Genus level	vOTU33	0.62	0.025
Flavobacteriia	*Lacinutrix*	*Lacinutrix algicola*	bOTU029	*Myoviridae*	*Yersinia* phage	Genus level	vOTU33	0.62	0.025
Alphaproteobacteria	*Planktomarina*	Genus level	bOTU029	*Myoviridae*	*Yersinia* phage	Genus level	vOTU33	0.64	0.019
Gammaproteobacteria	*Porticoccus*	*Porticoccus hydrocarbonoclasticus*	bOTU029	*Myoviridae*	*Yersinia* phage	Genus level	vOTU33	0.64	0.019
Gammaproteobacteria	*Porticoccus*	Genus level	bOTU029	*Myoviridae*	*Yersinia* phage	Genus level	vOTU33	0.64	0.019
Gammaproteobacteria	*Psychromonas*	Genus level	bOTU029	*Myoviridae*	*Yersinia* phage	Genus level	vOTU33	0.66	0.013
Epsilonproteobacteria	Class level	Class level	bOTU029	*Myoviridae*	*Yersinia* phage	Genus level	vOTU33	0.67	0.013
Planctomycetia	Class level	Class level	bOTU029	*Myoviridae*	*Yersinia* phage	Genus level	vOTU33	0.67	0.012
Flavobacteriia	*Polaribacter*	Genus level	bOTU029	*Myoviridae*	*Yersinia* phage	Genus level	vOTU33	0.67	0.012
Betaproteobacteria	Class level	Class level	bOTU029	*Myoviridae*	*Yersinia* phage	Genus level	vOTU33	0.68	0.011
Gammaproteobacteria	*Paraglaciecola*	*Paraglaciecola polaris*	bOTU029	*Myoviridae*	*Yersinia* phage	Genus level	vOTU33	0.68	0.011
Flavobacteriia	*Aquibacter*	Genus level	bOTU029	*Myoviridae*	*Yersinia* phage	Genus level	vOTU33	0.68	0.011
Gammaproteobacteria	*Azotobacter*	Genus level	bOTU029	*Myoviridae*	*Yersinia* phage	Genus level	vOTU33	0.68	0.011
Flavobacteriia	*Polaribacter*	*Polaribacter staleyi*	bOTU029	*Myoviridae*	*Yersinia* phage	Genus level	vOTU33	0.68	0.010
Alphaproteobacteria	*Amylibacter*	*Amylibacter cionae*	bOTU029	*Myoviridae*	*Yersinia* phage	Genus level	vOTU33	0.70	0.008
Gammaproteobacteria	*Cognaticolwellia*	*Cognaticolwellia aestuarii*	bOTU029	*Myoviridae*	*Yersinia* phage	Genus level	vOTU33	0.70	0.007
Gammaproteobacteria	*Cognaticolwellia*	Genus level	bOTU029	*Myoviridae*	*Yersinia* phage	Genus level	vOTU33	0.71	0.007
Flavobacteriia	*Owenweeksia*	Genus level	bOTU029	*Myoviridae*	*Yersinia* phage	Genus level	vOTU33	0.71	0.006
Gammaproteobacteria	Class level	Class level	bOTU029	*Myoviridae*	*Yersinia* phage	Genus level	vOTU33	0.72	0.006
Gammaproteobacteria	*Eionea*	*Eionea flava*	bOTU029	*Myoviridae*	*Yersinia* phage	Genus level	vOTU33	0.73	0.005
Gammaproteobacteria	*Eionea*	Genus level	bOTU029	*Myoviridae*	*Yersinia* phage	Genus level	vOTU33	0.73	0.005

Gammaproteobacteria	*Paraglaciecola*	Genus level	bOTU029	*Myoviridae*	*Yersinia* phage	Genus level	vOTU33	0.73	0.005
Gammaproteobacteria	*Sedimenticola*	*Sedimenticola thiotaurini*	bOTU029	*Myoviridae*	*Yersinia* phage	Genus level	vOTU33	0.74	0.004
Gammaproteobacteria	*Sedimenticola*	Genus level	bOTU029	*Myoviridae*	*Yersinia* phage	Genus level	vOTU33	0.74	0.004
Alphaproteobacteria	*Amylibacter*	Genus level	bOTU029	*Myoviridae*	*Yersinia* phage	Genus level	vOTU33	0.75	0.003
Gammaproteobacteria	*Pseudohongiella*	Genus level	bOTU029	*Myoviridae*	*Yersinia* phage	Genus level	vOTU33	0.76	0.003
Deltaproteobacteria	Class level	Class level	bOTU029	*Myoviridae*	*Yersinia* phage	Genus level	vOTU33	0.76	0.003
Gammaproteobacteria	*Thioprofundum*	Genus level	bOTU029	*Myoviridae*	*Yersinia* phage	Genus level	vOTU33	0.76	0.002
Gammaproteobacteria	*Maribrevibacterium*	Genus level	bOTU029	*Myoviridae*	*Yersinia* phage	Genus level	vOTU33	0.77	0.002
Gammaproteobacteria	*Moraxella*	*Moraxella oblonga*	bOTU029	*Myoviridae*	*Yersinia* phage	Genus level	vOTU33	0.77	0.002
Gammaproteobacteria	*Moraxella*	Genus level	bOTU029	*Myoviridae*	*Yersinia* phage	Genus level	vOTU33	0.77	0.002
Alphaproteobacteria	*Marinibaculum*	Genus level	bOTU029	*Myoviridae*	*Yersinia* phage	Genus level	vOTU33	0.81	0.001
Alphaproteobacteria	*Novosphingobium*	Genus level	bOTU029	*Myoviridae*	*Yersinia* phage	Genus level	vOTU33	0.83	0.001
Cytophagia	Class level	Class level	bOTU029	*Myoviridae*	*Yersinia* phage	Genus level	vOTU33	0.84	0.000
Cytophagia	*Marinoscillum*	Genus level	bOTU029	*Myoviridae*	*Yersinia* phage	Genus level	vOTU33	0.86	0.000
Flavobacteriia	*Owenweeksia*	Genus level	bOTU029	*Myoviridae*	*Aerosvirus*	Aeromonas virus 65	vOTU34	0.55	0.049
Gammaproteobacteria	*Sinobacterium*	Genus level	bOTU029	*Myoviridae*	*Aerosvirus*	Aeromonas virus 65	vOTU34	0.59	0.033
Gammaproteobacteria	*Thioprofundum*	Genus level	bOTU029	*Myoviridae*	*Aerosvirus*	Aeromonas virus 65	vOTU34	0.59	0.033
Gammaproteobacteria	*Maribrevibacterium*	Genus level	bOTU029	*Myoviridae*	*Aerosvirus*	Aeromonas virus 65	vOTU34	0.60	0.031
Cytophagia	Class level	Class level	bOTU029	*Myoviridae*	*Aerosvirus*	Aeromonas virus 65	vOTU34	0.61	0.027
Alphaproteobacteria	*Amylibacter*	*Amylibacter cionae*	bOTU029	*Myoviridae*	*Aerosvirus*	Aeromonas virus 65	vOTU34	0.63	0.021
Flavobacteriia	*Aquibacter*	Genus level	bOTU029	*Myoviridae*	*Aerosvirus*	Aeromonas virus 65	vOTU34	0.63	0.022
Alphaproteobacteria	*Novosphingobium*	Genus level	bOTU029	*Myoviridae*	*Aerosvirus*	Aeromonas virus 65	vOTU34	0.63	0.022
Gammaproteobacteria	*Parahaliea*	Genus level	bOTU029	*Myoviridae*	*Aerosvirus*	Aeromonas virus 65	vOTU34	0.64	0.020
Gammaproteobacteria	*Sedimenticola*	*Sedimenticola thiotaurini*	bOTU029	*Myoviridae*	*Aerosvirus*	Aeromonas virus 65	vOTU34	0.64	0.018
Gammaproteobacteria	*Sedimenticola*	Genus level	bOTU029	*Myoviridae*	*Aerosvirus*	Aeromonas virus 65	vOTU34	0.64	0.018
Gammaproteobacteria	*Marinobacterium*	Genus level	bOTU029	*Myoviridae*	*Aerosvirus*	Aeromonas virus 65	vOTU34	0.65	0.017
Alphaproteobacteria	*Magnetospira*	Genus level	bOTU029	*Myoviridae*	*Aerosvirus*	Aeromonas virus 65	vOTU34	0.66	0.015
Gammaproteobacteria	*Halomonas*	*Halomonas glaciei*	bOTU029	*Myoviridae*	*Aerosvirus*	Aeromonas virus 65	vOTU34	0.66	0.014
Gammaproteobacteria	*Halomonas*	Genus level	bOTU029	*Myoviridae*	*Aerosvirus*	Aeromonas virus 65	vOTU34	0.66	0.014

Deltaproteobacteria	Class level	Class level	bOTU029	*Myoviridae*	*Aerosvirus*	Aeromonas virus 65	vOTU34	0.66	0.014
Alphaproteobacteria	*Amylibacter*	Genus level	bOTU029	*Myoviridae*	*Aerosvirus*	Aeromonas virus 65	vOTU34	0.66	0.013
Gammaproteobacteria	Class level	Class level	bOTU029	*Myoviridae*	*Aerosvirus*	Aeromonas virus 65	vOTU34	0.67	0.013
Betaproteobacteria	Class level	Class level	bOTU029	*Myoviridae*	*Aerosvirus*	Aeromonas virus 65	vOTU34	0.67	0.011
Alphaproteobacteria	*Ahrensia*	Genus level	bOTU029	*Myoviridae*	*Aerosvirus*	Aeromonas virus 65	vOTU34	0.69	0.010
Gammaproteobacteria	*Pseudohongiella*	Genus level	bOTU029	*Myoviridae*	*Aerosvirus*	Aeromonas virus 65	vOTU34	0.71	0.007
Gammaproteobacteria	*Moraxella*	*Moraxella oblonga*	bOTU029	*Myoviridae*	*Aerosvirus*	Aeromonas virus 65	vOTU34	0.73	0.005
Gammaproteobacteria	*Moraxella*	Genus level	bOTU029	*Myoviridae*	*Aerosvirus*	Aeromonas virus 65	vOTU34	0.73	0.005
Gammaproteobacteria	*Halomonas*	*Halomonas glaciei*	bOTU029	*Myoviridae*		Sphingomonas phage PAU	vOTU40	0.56	0.049
Gammaproteobacteria	*Halomonas*	Genus level	bOTU029	*Myoviridae*		Sphingomonas phage PAU	vOTU40	0.56	0.049
Flavobacteriia	*Owenweeksia*	Genus level	bOTU029	*Myoviridae*		Sphingomonas phage PAU	vOTU40	0.56	0.047
Gammaproteobacteria	*Pseudomonas*	*Pseudomonas stutzeri*	bOTU029	*Myoviridae*		Sphingomonas phage PAU	vOTU40	0.56	0.047
Flavobacteriia	*Cellulophaga*	Genus level	bOTU029	*Myoviridae*		Sphingomonas phage PAU	vOTU40	0.56	0.047
Flavobacteriia	*Polaribacter*	*Polaribacter staleyi*	bOTU029	*Myoviridae*		Sphingomonas phage PAU	vOTU40	0.56	0.045
Alphaproteobacteria	*Sphingomonas*	Genus level	bOTU029	*Myoviridae*		Sphingomonas phage PAU	vOTU40	0.58	0.039
Flavobacteriia	*Algibacter*	*Algibacter miyuki*	bOTU029	*Myoviridae*		Sphingomonas phage PAU	vOTU40	0.61	0.028
Flavobacteriia	*Algibacter*	Genus level	bOTU029	*Myoviridae*		Sphingomonas phage PAU	vOTU40	0.61	0.028
Flavobacteriia	*Polaribacter*	Genus level	bOTU029	*Myoviridae*		Sphingomonas phage PAU	vOTU40	0.61	0.028
Gammaproteobacteria	*Thioprofundum*	Genus level	bOTU029	*Myoviridae*		Sphingomonas phage PAU	vOTU40	0.62	0.025
Gammaproteobacteria	*Pseudomonas*	*Pseudomonas sabulinigri*	bOTU029	*Myoviridae*		Sphingomonas phage PAU	vOTU40	0.64	0.018
Flavobacteriia	*Maribacter*	Genus level	bOTU029	*Myoviridae*		Sphingomonas phage PAU	vOTU40	0.64	0.018
Betaproteobacteria	Class level	Class level	bOTU029	*Myoviridae*		Sphingomonas phage PAU	vOTU40	0.64	0.018
Flavobacteriia	*Polaribacter*	*Polaribacter haliotis*	bOTU029	*Myoviridae*		Sphingomonas phage PAU	vOTU40	0.66	0.015
Gammaproteobacteria	*Psychrobacter*	Genus level	bOTU029	*Myoviridae*		Sphingomonas phage PAU	vOTU40	0.66	0.015
Alphaproteobacteria	*Parasphingorhabdus*	*Parasphingorhabdus flavimaris*	bOTU029	*Myoviridae*		Sphingomonas phage PAU	vOTU40	0.69	0.009
Alphaproteobacteria	*Parasphingorhabdus*	Genus level	bOTU029	*Myoviridae*		Sphingomonas phage PAU	vOTU40	0.69	0.009
Gammaproteobacteria	*Pseudomonas*	*Pseudomonas chloritidismutans*	bOTU029	*Myoviridae*		Sphingomonas phage PAU	vOTU40	0.69	0.009
Gammaproteobacteria	*Pseudomonas*	Genus level	bOTU029	*Myoviridae*		Sphingomonas phage PAU	vOTU40	0.73	0.005

Gammaproteobacteria	*Psychrobacter*	*Psychrobacter nivimaris*	bOTU029	*Myoviridae*		Sphingomonas phage PAU	vOTU40	0.78	0.002
Actinomycetia	*Rhodoluna*	Genus level	bOTU029	*Myoviridae*	*Lipsvirus*	Synechococcus phage S-SSM7	vOTU41	0.68	0.011
Acidimicrobiia	*Ilumatobacter*	*Ilumatobacter fluminis*	bOTU029	*Myoviridae*	*Lipsvirus*	Synechococcus phage S-SSM7	vOTU41	0.82	0.001
Acidimicrobiia	*Ilumatobacter*	Genus level	bOTU029	*Myoviridae*	*Lipsvirus*	Synechococcus phage S-SSM7	vOTU41	0.82	0.001
Alphaproteobacteria	Class level	Class level	bOTU029	*Myoviridae*	*Lipsvirus*	Synechococcus phage S-SSM7	vOTU41	0.82	0.001
Alphaproteobacteria	*Sulfitobacter*	*Sulfitobacter profundi*	bOTU029	*Myoviridae*	*Lipsvirus*	Synechococcus phage S-SSM7	vOTU41	0.85	0.000
Alphaproteobacteria	*Sulfitobacter*	Genus level	bOTU029	*Myoviridae*	*Lipsvirus*	Synechococcus phage S-SSM7	vOTU41	0.85	0.000
Alphaproteobacteria	*Loktanella*	*Loktanella acticola*	bOTU029	*Myoviridae*	*Lipsvirus*	Synechococcus phage S-SSM7	vOTU41	0.88	0.000
Alphaproteobacteria	*Loktanella*	Genus level	bOTU029	*Myoviridae*	*Lipsvirus*	Synechococcus phage S-SSM7	vOTU41	0.88	0.000
Gammaproteobacteria	*Colwellia*	*Colwellia echini*	bOTU029	*Myoviridae*	*Lipsvirus*	Synechococcus phage S-WAM2	vOTU42	0.59	0.033
Alphaproteobacteria	*Sphingomonas*	Genus level	bOTU029	*Myoviridae*	*Lipsvirus*	Synechococcus phage S-WAM2	vOTU42	0.60	0.032
Betaproteobacteria	Class level	Class level	bOTU029	*Myoviridae*	*Lipsvirus*	Synechococcus phage S-WAM2	vOTU42	0.60	0.031
Gammaproteobacteria	*Pseudomonas*	Genus level	bOTU029	*Myoviridae*	*Lipsvirus*	Synechococcus phage S-WAM2	vOTU42	0.61	0.028
Gammaproteobacteria	*Cognaticolwellia*	Genus level	bOTU029	*Myoviridae*	*Lipsvirus*	Synechococcus phage S-WAM2	vOTU42	0.63	0.021
Gammaproteobacteria	*Colwellia*	Genus level	bOTU029	*Myoviridae*	*Lipsvirus*	Synechococcus phage S-WAM2	vOTU42	0.63	0.020
Gammaproteobacteria	*Thioprofundum*	Genus level	bOTU029	*Myoviridae*	*Lipsvirus*	Synechococcus phage S-WAM2	vOTU42	0.63	0.020
Gammaproteobacteria	*Cognaticolwellia*	*Cognaticolwellia aestuarii*	bOTU029	*Myoviridae*	*Lipsvirus*	Synechococcus phage S-WAM2	vOTU42	0.64	0.018
Flavobacteriia	*Winogradskyella*	Genus level	bOTU029	*Myoviridae*	*Lipsvirus*	Synechococcus phage S-WAM2	vOTU42	0.65	0.017
Gammaproteobacteria	*Psychrobacter*	*Psychrobacter nivimaris*	bOTU029	*Myoviridae*	*Lipsvirus*	Synechococcus phage S-WAM2	vOTU42	0.67	0.012
Alphaproteobacteria	*Parasphingorhabdus*	*Parasphingorhabdus flavimaris*	bOTU029	*Myoviridae*	*Lipsvirus*	Synechococcus phage S-WAM2	vOTU42	0.67	0.012
Alphaproteobacteria	*Parasphingorhabdus*	Genus level	bOTU029	*Myoviridae*	*Lipsvirus*	Synechococcus phage S-WAM2	vOTU42	0.67	0.012
Gammaproteobacteria	*Pseudomonas*	*Pseudomonas sabulinigri*	bOTU029	*Myoviridae*	*Lipsvirus*	Synechococcus phage S-WAM2	vOTU42	0.68	0.010
Planctomycetia	Class level	Class level	bOTU029	*Myoviridae*	*Lipsvirus*	Synechococcus phage S-WAM2	vOTU42	0.69	0.009
Gammaproteobacteria	*Psychrosphaera*	Genus level	bOTU029	*Myoviridae*	*Lipsvirus*	Synechococcus phage S-WAM2	vOTU42	0.70	0.008
Gammaproteobacteria	*Psychrobacter*	Genus level	bOTU029	*Myoviridae*	*Lipsvirus*	Synechococcus phage S-WAM2	vOTU42	0.71	0.006
Gammaproteobacteria	*Psychromonas*	Genus level	bOTU029	*Myoviridae*	*Lipsvirus*	Synechococcus phage S-WAM2	vOTU42	0.73	0.004
Gammaproteobacteria	*Paraglaciecola*	*Paraglaciecola polaris*	bOTU029	*Myoviridae*	*Lipsvirus*	Synechococcus phage S-WAM2	vOTU42	0.74	0.004

Flavobacteriia	*Cellulophaga*	Genus level	bOTU029	*Myoviridae*	*Lipsvirus*	Synechococcus phage S-WAM2	vOTU42	0.74	0.004
Flavobacteriia	*Algibacter*	*Algibacter miyuki*	bOTU029	*Myoviridae*	*Lipsvirus*	Synechococcus phage S-WAM2	vOTU42	0.76	0.003
Flavobacteriia	*Algibacter*	Genus level	bOTU029	*Myoviridae*	*Lipsvirus*	Synechococcus phage S-WAM2	vOTU42	0.76	0.003
Flavobacteriia	*Lacinutrix*	Genus level	bOTU029	*Myoviridae*	*Lipsvirus*	Synechococcus phage S-WAM2	vOTU42	0.77	0.002
Gammaproteobacteria	*Paraglaciecola*	Genus level	bOTU029	*Myoviridae*	*Lipsvirus*	Synechococcus phage S-WAM2	vOTU42	0.78	0.002
Flavobacteriia	Class level	Class level	bOTU029	*Myoviridae*	*Lipsvirus*	Synechococcus phage S-WAM2	vOTU42	0.80	0.001
Flavobacteriia	*Polaribacter*	Genus level	bOTU029	*Myoviridae*	*Lipsvirus*	Synechococcus phage S-WAM2	vOTU42	0.82	0.001
Flavobacteriia	*Polaribacter*	*Polaribacter haliotis*	bOTU029	*Myoviridae*	*Lipsvirus*	Synechococcus phage S-WAM2	vOTU42	0.83	0.000
Flavobacteriia	*Polaribacter*	*Polaribacter staleyi*	bOTU029	*Myoviridae*	*Lipsvirus*	Synechococcus phage S-WAM2	vOTU42	0.84	0.000
Flavobacteriia	*Lacinutrix*	*Lacinutrix algicola*	bOTU029	*Myoviridae*	*Lipsvirus*	Synechococcus phage S-WAM2	vOTU42	0.88	0.000
Flavobacteriia	*Maribacter*	Genus level	bOTU029	*Myoviridae*	*Lipsvirus*	Synechococcus phage S-WAM2	vOTU42	0.91	0.000
Gammaproteobacteria	*Pseudomonas*	*Pseudomonas sabulinigri*	bOTU029	*Myoviridae*	*Eneladusvirus*	Yersinia phage fHe-Yen9-04	vOTU44	0.56	0.048
Flavobacteriia	Class level	Class level	bOTU029	*Myoviridae*	*Eneladusvirus*	Yersinia phage fHe-Yen9-04	vOTU44	0.57	0.044
Alphaproteobacteria	*Ahrensia*	Genus level	bOTU029	*Myoviridae*	*Eneladusvirus*	Yersinia phage fHe-Yen9-04	vOTU44	0.57	0.043
Gammaproteobacteria	*Pseudoalteromonas*	*Pseudoalteromonas hodoensis*	bOTU029	*Myoviridae*	*Eneladusvirus*	Yersinia phage fHe-Yen9-04	vOTU44	0.59	0.035
Flavobacteriia	*Mesoflavibacter*	Genus level	bOTU029	*Myoviridae*	*Eneladusvirus*	Yersinia phage fHe-Yen9-04	vOTU44	0.59	0.034
Gammaproteobacteria	*Colwellia*	*Colwellia echini*	bOTU029	*Myoviridae*	*Eneladusvirus*	Yersinia phage fHe-Yen9-04	vOTU44	0.60	0.031
Gammaproteobacteria	*Sinobacterium*	Genus level	bOTU029	*Myoviridae*	*Eneladusvirus*	Yersinia phage fHe-Yen9-04	vOTU44	0.60	0.031
Flavobacteriia	*Polaribacter*	*Polaribacter haliotis*	bOTU029	*Myoviridae*	*Eneladusvirus*	Yersinia phage fHe-Yen9-04	vOTU44	0.61	0.028
Alphaproteobacteria	*Magnetospira*	Genus level	bOTU029	*Myoviridae*	*Eneladusvirus*	Yersinia phage fHe-Yen9-04	vOTU44	0.61	0.026
Flavobacteriia	*Vicingus*	*Vicingus serpentipes*	bOTU029	*Myoviridae*	*Eneladusvirus*	Yersinia phage fHe-Yen9-04	vOTU44	0.61	0.026
Gammaproteobacteria	*Colwellia*	Genus level	bOTU029	*Myoviridae*	*Eneladusvirus*	Yersinia phage fHe-Yen9-04	vOTU44	0.61	0.026
Vicingus	Class level	Class level	bOTU029	*Myoviridae*	*Eneladusvirus*	Yersinia phage fHe-Yen9-04	vOTU44	0.61	0.026
Gammaproteobacteria	*Pseudoalteromonas*	Genus level	bOTU029	*Myoviridae*	*Eneladusvirus*	Yersinia phage fHe-Yen9-04	vOTU44	0.62	0.025
Flavobacteriia	*Lacinutrix*	*Lacinutrix algicola*	bOTU029	*Myoviridae*	*Eneladusvirus*	Yersinia phage fHe-Yen9-04	vOTU44	0.62	0.025
Alphaproteobacteria	*Planktomarina*	Genus level	bOTU029	*Myoviridae*	*Eneladusvirus*	Yersinia phage fHe-Yen9-04	vOTU44	0.64	0.019
Gammaproteobacteria	*Porticoccus*	*Porticoccus hydrocarbonoclasticus*	bOTU029	*Myoviridae*	*Eneladusvirus*	Yersinia phage fHe-Yen9-04	vOTU44	0.64	0.019

Gammaproteobacteria	*Porticoccus*	Genus level	bOTU029	*Myoviridae*	*Eneladusvirus*	Yersinia phage fHe-Yen9-04	vOTU44	0.64	0.019
Gammaproteobacteria	*Psychromonas*	Genus level	bOTU029	*Myoviridae*	*Eneladusvirus*	Yersinia phage fHe-Yen9-04	vOTU44	0.66	0.013
Epsilonproteobacteria	Class level	Class level	bOTU029	*Myoviridae*	*Eneladusvirus*	Yersinia phage fHe-Yen9-04	vOTU44	0.67	0.013
Planctomycetia	Class level	Class level	bOTU029	*Myoviridae*	*Eneladusvirus*	Yersinia phage fHe-Yen9-04	vOTU44	0.67	0.012
Flavobacteriia	*Polaribacter*	Genus level	bOTU029	*Myoviridae*	*Eneladusvirus*	Yersinia phage fHe-Yen9-04	vOTU44	0.67	0.012
Betaproteobacteria	Class level	Class level	bOTU029	*Myoviridae*	*Eneladusvirus*	Yersinia phage fHe-Yen9-04	vOTU44	0.68	0.011
Gammaproteobacteria	*Paraglaciecola*	*Paraglaciecola polaris*	bOTU029	*Myoviridae*	*Eneladusvirus*	Yersinia phage fHe-Yen9-04	vOTU44	0.68	0.011
Flavobacteriia	*Aquibacter*	Genus level	bOTU029	*Myoviridae*	*Eneladusvirus*	Yersinia phage fHe-Yen9-04	vOTU44	0.68	0.011
Gammaproteobacteria	*Azotobacter*	Genus level	bOTU029	*Myoviridae*	*Eneladusvirus*	Yersinia phage fHe-Yen9-04	vOTU44	0.68	0.011
Flavobacteriia	*Polaribacter*	*Polaribacter staleyi*	bOTU029	*Myoviridae*	*Eneladusvirus*	Yersinia phage fHe-Yen9-04	vOTU44	0.68	0.010
Alphaproteobacteria	*Amylibacter*	*Amylibacter cionae*	bOTU029	*Myoviridae*	*Eneladusvirus*	Yersinia phage fHe-Yen9-04	vOTU44	0.70	0.008
Gammaproteobacteria	*Cognaticolwellia*	*Cognaticolwellia aestuarii*	bOTU029	*Myoviridae*	*Eneladusvirus*	Yersinia phage fHe-Yen9-04	vOTU44	0.70	0.007
Gammaproteobacteria	*Cognaticolwellia*	Genus level	bOTU029	*Myoviridae*	*Eneladusvirus*	Yersinia phage fHe-Yen9-04	vOTU44	0.71	0.007
Flavobacteriia	*Owenweeksia*	Genus level	bOTU029	*Myoviridae*	*Eneladusvirus*	Yersinia phage fHe-Yen9-04	vOTU44	0.71	0.006
Gammaproteobacteria	Class level	Class level	bOTU029	*Myoviridae*	*Eneladusvirus*	Yersinia phage fHe-Yen9-04	vOTU44	0.72	0.006
Gammaproteobacteria	*Eionea*	*Eionea flava*	bOTU029	*Myoviridae*	*Eneladusvirus*	Yersinia phage fHe-Yen9-04	vOTU44	0.73	0.005
Gammaproteobacteria	*Eionea*	Genus level	bOTU029	*Myoviridae*	*Eneladusvirus*	Yersinia phage fHe-Yen9-04	vOTU44	0.73	0.005
Gammaproteobacteria	*Paraglaciecola*	Genus level	bOTU029	*Myoviridae*	*Eneladusvirus*	Yersinia phage fHe-Yen9-04	vOTU44	0.73	0.005
Gammaproteobacteria	*Sedimenticola*	*Sedimenticola thiotaurini*	bOTU029	*Myoviridae*	*Eneladusvirus*	Yersinia phage fHe-Yen9-04	vOTU44	0.74	0.004
Gammaproteobacteria	*Sedimenticola*	Genus level	bOTU029	*Myoviridae*	*Eneladusvirus*	Yersinia phage fHe-Yen9-04	vOTU44	0.74	0.004
Alphaproteobacteria	*Amylibacter*	Genus level	bOTU029	*Myoviridae*	*Eneladusvirus*	Yersinia phage fHe-Yen9-04	vOTU44	0.75	0.003
Gammaproteobacteria	*Pseudohongiella*	Genus level	bOTU029	*Myoviridae*	*Eneladusvirus*	Yersinia phage fHe-Yen9-04	vOTU44	0.76	0.003
Deltaproteobacteria	Class level	Class level	bOTU029	*Myoviridae*	*Eneladusvirus*	Yersinia phage fHe-Yen9-04	vOTU44	0.76	0.003
Gammaproteobacteria	*Thioprofundum*	Genus level	bOTU029	*Myoviridae*	*Eneladusvirus*	Yersinia phage fHe-Yen9-04	vOTU44	0.76	0.002
Gammaproteobacteria	*Maribrevibacterium*	Genus level	bOTU029	*Myoviridae*	*Eneladusvirus*	Yersinia phage fHe-Yen9-04	vOTU44	0.77	0.002
Gammaproteobacteria	*Moraxella*	*Moraxella oblonga*	bOTU029	*Myoviridae*	*Eneladusvirus*	Yersinia phage fHe-Yen9-04	vOTU44	0.77	0.002
Gammaproteobacteria	*Moraxella*	Genus level	bOTU029	*Myoviridae*	*Eneladusvirus*	Yersinia phage fHe-Yen9-04	vOTU44	0.77	0.002

Alphaproteobacteria	*Marinibaculum*	Genus level	bOTU029	*Myoviridae*	*Eneladusvirus*	Yersinia phage fHe-Yen9-04	vOTU44	0.81	0.001
Alphaproteobacteria	*Novosphingobium*	Genus level	bOTU029	*Myoviridae*	*Eneladusvirus*	Yersinia phage fHe-Yen9-04	vOTU44	0.83	0.001
Cytophagia	Class level	Class level	bOTU029	*Myoviridae*	*Eneladusvirus*	Yersinia phage fHe-Yen9-04	vOTU44	0.84	0.000
Cytophagia	*Marinoscillum*	Genus level	bOTU029	*Myoviridae*	*Eneladusvirus*	Yersinia phage fHe-Yen9-04	vOTU44	0.86	0.000
Gammaproteobacteria	*Psychrobacter*	*Psychrobacter submarinus*	bOTU029	*Podoviridae*	*Nonlabens* phage	Genus level	vOTU19	0.59	0.034
Gammaproteobacteria	*Pseudomonas*	*Pseudomonas stutzeri*	bOTU029	*Podoviridae*	*Nonlabens* phage	Genus level	vOTU19	0.60	0.029
Gammaproteobacteria	*Parahaliea*	Genus level	bOTU029	*Podoviridae*	*Nonlabens* phage	Genus level	vOTU19	0.64	0.019
Gammaproteobacteria	*Pseudomonas*	*Pseudomonas chloritidismutans*	bOTU029	*Podoviridae*	*Nonlabens* phage	Genus level	vOTU19	0.66	0.015
Acidimicrobiia	*Acidimicrobiia*	Genus level	bOTU029	*Podoviridae*	*Podovirus* phage	Genus level	vOTU20	0.57	0.041
Flavobacteriia	*Leeuwenhoekiella*	Genus level	bOTU029	*Podoviridae*	*Podovirus* phage	Genus level	vOTU20	0.70	0.008
Gammaproteobacteria	*Pseudoalteromonas*	Genus level	bOTU029	*Podoviridae*	*Pseudomonas* phage	Genus level	vOTU23	0.66	0.013
Gammaproteobacteria	*Pseudoalteromonas*	*Pseudoalteromonas hodoensis*	bOTU029	*Podoviridae*	*Pseudomonas* phage	Genus level	vOTU23	0.67	0.012
Gammaproteobacteria	*Psychrobacter*	*Psychrobacter nivimaris*	bOTU029	*Podoviridae*	*Pseudomonas* phage	Genus level	vOTU23	0.67	0.011
Gammaproteobacteria	*Thiohalobacter*	Genus level	bOTU029	*Podoviridae*	*Puniceispirillum* phage	Genus level	vOTU24	0.56	0.048
Gammaproteobacteria	*Psychrobacter*	Genus level	bOTU029	*Podoviridae*	*Puniceispirillum* phage	Genus level	vOTU24	0.58	0.037
Gammaproteobacteria	*Porticoccus*	*Porticoccus hydrocarbonoclasticus*	bOTU029	*Podoviridae*	*Puniceispirillum* phage	Genus level	vOTU24	0.58	0.036
Gammaproteobacteria	*Porticoccus*	Genus level	bOTU029	*Podoviridae*	*Puniceispirillum* phage	Genus level	vOTU24	0.58	0.036
Gammaproteobacteria	*Psychromonas*	Genus level	bOTU029	*Podoviridae*	*Puniceispirillum* phage	Genus level	vOTU24	0.59	0.036
Flavobacteriia	*Polaribacter*	*Polaribacter haliotis*	bOTU029	*Podoviridae*	*Puniceispirillum* phage	Genus level	vOTU24	0.59	0.032
Gammaproteobacteria	*Halomonas*	*Halomonas glaciei*	bOTU029	*Podoviridae*	*Puniceispirillum* phage	Genus level	vOTU24	0.60	0.032
Gammaproteobacteria	*Halomonas*	Genus level	bOTU029	*Podoviridae*	*Puniceispirillum* phage	Genus level	vOTU24	0.60	0.032
Flavobacteriia	*Polaribacter*	*Polaribacter staleyi*	bOTU029	*Podoviridae*	*Puniceispirillum* phage	Genus level	vOTU24	0.61	0.026
Gammaproteobacteria	*Pseudomonas*	*Pseudomonas chloritidismutans*	bOTU029	*Podoviridae*	*Puniceispirillum* phage	Genus level	vOTU24	0.62	0.025
Flavobacteriia	*Polaribacter*	Genus level	bOTU029	*Podoviridae*	*Puniceispirillum* phage	Genus level	vOTU24	0.62	0.024
Gammaproteobacteria	*Pseudoalteromonas*	Genus level	bOTU029	*Podoviridae*	*Puniceispirillum* phage	Genus level	vOTU24	0.63	0.021
Gammaproteobacteria	*Pseudomonas*	*Pseudomonas sabulinigri*	bOTU029	*Podoviridae*	*Puniceispirillum* phage	Genus level	vOTU24	0.64	0.019
Flavobacteriia	*Vicingus*	*Vicingus serpentipes*	bOTU029	*Podoviridae*	*Puniceispirillum* phage	Genus level	vOTU24	0.64	0.019
Vicingus	Class level	Class level	bOTU029	*Podoviridae*	*Puniceispirillum* phage	Genus level	vOTU24	0.64	0.019

Gammaproteobacteria	*Colwellia*	Genus level	bOTU029	*Podoviridae*	*Puniceispirillum* phage	Genus level	vOTU24	0.64	0.019
Gammaproteobacteria	*Colwellia*	*Colwellia echini*	bOTU029	*Podoviridae*	*Puniceispirillum* phage	Genus level	vOTU24	0.64	0.018
Gammaproteobacteria	*Pseudoalteromonas*	*Pseudoalteromonas hodoensis*	bOTU029	*Podoviridae*	*Puniceispirillum* phage	Genus level	vOTU24	0.64	0.018
Gammaproteobacteria	*Marinobacterium*	Genus level	bOTU029	*Podoviridae*	*Puniceispirillum* phage	Genus level	vOTU24	0.65	0.016
Gammaproteobacteria	*Pseudomonas*	Genus level	bOTU029	*Podoviridae*	*Puniceispirillum* phage	Genus level	vOTU24	0.65	0.016
Gammaproteobacteria	*Parahaliea*	Genus level	bOTU029	*Podoviridae*	*Puniceispirillum* phage	Genus level	vOTU24	0.66	0.014
Gammaproteobacteria	*Sinobacterium*	Genus level	bOTU029	*Podoviridae*	*Puniceispirillum* phage	Genus level	vOTU24	0.68	0.010
Gammaproteobacteria	*Paraglaciecola*	*Paraglaciecola polaris*	bOTU029	*Podoviridae*	*Puniceispirillum* phage	Genus level	vOTU24	0.68	0.010
Gammaproteobacteria	*Paraglaciecola*	Genus level	bOTU029	*Podoviridae*	*Puniceispirillum* phage	Genus level	vOTU24	0.69	0.009
Gammaproteobacteria	*Cognaticolwellia*	*Cognaticolwellia aestuarii*	bOTU029	*Podoviridae*	*Puniceispirillum* phage	Genus level	vOTU24	0.71	0.006
Gammaproteobacteria	*Cognaticolwellia*	Genus level	bOTU029	*Podoviridae*	*Puniceispirillum* phage	Genus level	vOTU24	0.72	0.005
Planctomycetia	Class level	Class level	bOTU029	*Podoviridae*	*Puniceispirillum* phage	Genus level	vOTU24	0.73	0.005
Alphaproteobacteria	*Magnetospira*	Genus level	bOTU029	*Podoviridae*	*Puniceispirillum* phage	Genus level	vOTU24	0.74	0.004
Alphaproteobacteria	*Ahrensia*	Genus level	bOTU029	*Podoviridae*	*Puniceispirillum* phage	Genus level	vOTU24	0.74	0.004
Gammaproteobacteria	*Eionea*	*Eionea flava*	bOTU029	*Podoviridae*	*Puniceispirillum* phage	Genus level	vOTU24	0.76	0.002
Gammaproteobacteria	*Eionea*	Genus level	bOTU029	*Podoviridae*	*Puniceispirillum* phage	Genus level	vOTU24	0.76	0.002
Epsilonproteobacteria	Class level	Class level	bOTU029	*Podoviridae*	*Puniceispirillum* phage	Genus level	vOTU24	0.77	0.002
Cytophagia	*Marinoscillum*	Genus level	bOTU029	*Podoviridae*	*Puniceispirillum* phage	Genus level	vOTU24	0.77	0.002
Gammaproteobacteria	*Pseudomonas*	*Pseudomonas stutzeri*	bOTU029	*Podoviridae*	*Puniceispirillum* phage	Genus level	vOTU24	0.78	0.002
Gammaproteobacteria	*Maribrevibacterium*	Genus level	bOTU029	*Podoviridae*	*Puniceispirillum* phage	Genus level	vOTU24	0.78	0.002
Gammaproteobacteria	*Thioprofundum*	Genus level	bOTU029	*Podoviridae*	*Puniceispirillum* phage	Genus level	vOTU24	0.78	0.002
Alphaproteobacteria	*Marinibaculum*	Genus level	bOTU029	*Podoviridae*	*Puniceispirillum* phage	Genus level	vOTU24	0.81	0.001
Alphaproteobacteria	*Amylibacter*	*Amylibacter cionae*	bOTU029	*Podoviridae*	*Puniceispirillum* phage	Genus level	vOTU24	0.81	0.001
Alphaproteobacteria	*Amylibacter*	Genus level	bOTU029	*Podoviridae*	*Puniceispirillum* phage	Genus level	vOTU24	0.82	0.001
Flavobacteriia	*Owenweeksia*	Genus level	bOTU029	*Podoviridae*	*Puniceispirillum* phage	Genus level	vOTU24	0.82	0.001
Betaproteobacteria	Class level	Class level	bOTU029	*Podoviridae*	*Puniceispirillum* phage	Genus level	vOTU24	0.82	0.001
Gammaproteobacteria	*Pseudohongiella*	Genus level	bOTU029	*Podoviridae*	*Puniceispirillum* phage	Genus level	vOTU24	0.83	0.000
Flavobacteriia	*Aquibacter*	Genus level	bOTU029	*Podoviridae*	*Puniceispirillum* phage	Genus level	vOTU24	0.83	0.000
Gammaproteobacteria	*Sedimenticola*	*Sedimenticola thiotaurini*	bOTU029	*Podoviridae*	*Puniceispirillum* phage	Genus level	vOTU24	0.84	0.000

Gammaproteobacteria	*Sedimenticola*	Genus level	bOTU029	*Podoviridae*	*Puniceispirillum* phage	Genus level	vOTU24	0.84	0.000
Gammaproteobacteria	*Moraxella*	*Moraxella oblonga*	bOTU029	*Podoviridae*	*Puniceispirillum* phage	Genus level	vOTU24	0.85	0.000
Gammaproteobacteria	*Moraxella*	Genus level	bOTU029	*Podoviridae*	*Puniceispirillum* phage	Genus level	vOTU24	0.85	0.000
Alphaproteobacteria	*Novosphingobium*	Genus level	bOTU029	*Podoviridae*	*Puniceispirillum* phage	Genus level	vOTU24	0.87	0.000
Gammaproteobacteria	Class level	Class level	bOTU029	*Podoviridae*	*Puniceispirillum* phage	Genus level	vOTU24	0.88	0.000
Cytophagia	Class level	Class level	bOTU029	*Podoviridae*	*Puniceispirillum* phage	Genus level	vOTU24	0.89	0.000
Deltaproteobacteria	Class level	Class level	bOTU029	*Podoviridae*	*Puniceispirillum* phage	Genus level	vOTU24	0.90	0.000
Cyanophyceae	*Foliisarcina*	Genus level	bOTU029	*Podoviridae*	*Callevirus*	Cellulophaga phage phi38:1	vOTU35	0.57	0.044
Planctomycetia	Class level	Class level	bOTU029	*Podoviridae*		Pelagibacter phage HTVC010P	vOTU37	0.55	0.049
Gammaproteobacteria	*Porticoccus*	*Porticoccus hydrocarbonoclasticus*	bOTU029	*Podoviridae*		Pelagibacter phage HTVC010P	vOTU37	0.56	0.049
Gammaproteobacteria	*Porticoccus*	Genus level	bOTU029	*Podoviridae*		Pelagibacter phage HTVC010P	vOTU37	0.56	0.049
Flavobacteriia	*Owenweeksia*	Genus level	bOTU029	*Podoviridae*		Pelagibacter phage HTVC010P	vOTU37	0.56	0.046
Alphaproteobacteria	*Planktomarina*	Genus level	bOTU029	*Podoviridae*		Pelagibacter phage HTVC010P	vOTU37	0.57	0.044
Gammaproteobacteria	*Moraxella*	*Moraxella oblonga*	bOTU029	*Podoviridae*		Pelagibacter phage HTVC010P	vOTU37	0.57	0.044
Gammaproteobacteria	*Moraxella*	Genus level	bOTU029	*Podoviridae*		Pelagibacter phage HTVC010P	vOTU37	0.57	0.044
Gammaproteobacteria	*Sedimenticola*	*Sedimenticola thiotaurini*	bOTU029	*Podoviridae*		Pelagibacter phage HTVC010P	vOTU37	0.58	0.040
Flavobacteriia	*Polaribacter*	Genus level	bOTU029	*Podoviridae*		Pelagibacter phage HTVC010P	vOTU37	0.58	0.040
Gammaproteobacteria	*Sedimenticola*	Genus level	bOTU029	*Podoviridae*		Pelagibacter phage HTVC010P	vOTU37	0.58	0.040
Flavobacteriia	*Polaribacter*	*Polaribacter staleyi*	bOTU029	*Podoviridae*		Pelagibacter phage HTVC010P	vOTU37	0.58	0.038
Gammaproteobacteria	*Maribrevibacterium*	Genus level	bOTU029	*Podoviridae*		Pelagibacter phage HTVC010P	vOTU37	0.58	0.038
Gammaproteobacteria	*Paraglaciecola*	Genus level	bOTU029	*Podoviridae*		Pelagibacter phage HTVC010P	vOTU37	0.58	0.037
Alphaproteobacteria	*Amylibacter*	Genus level	bOTU029	*Podoviridae*		Pelagibacter phage HTVC010P	vOTU37	0.59	0.033
Gammaproteobacteria	*Eionea*	*Eionea flava*	bOTU029	*Podoviridae*		Pelagibacter phage HTVC010P	vOTU37	0.59	0.032
Gammaproteobacteria	*Eionea*	Genus level	bOTU029	*Podoviridae*		Pelagibacter phage HTVC010P	vOTU37	0.59	0.032
Alphaproteobacteria	*Magnetospira*	Genus level	bOTU029	*Podoviridae*		Pelagibacter phage HTVC010P	vOTU37	0.60	0.029
Alphaproteobacteria	*Amylibacter*	*Amylibacter cionae*	bOTU029	*Podoviridae*		Pelagibacter phage HTVC010P	vOTU37	0.61	0.028
Gammaproteobacteria	*Thioprofundum*	Genus level	bOTU029	*Podoviridae*		Pelagibacter phage HTVC010P	vOTU37	0.61	0.028

Epsilonproteobacteria	Class level	Class level	bOTU029	*Podoviridae*		Pelagibacter phage HTVC010P	vOTU37	0.61	0.026
Gammaproteobacteria	*Psychromonas*	Genus level	bOTU029	*Podoviridae*		Pelagibacter phage HTVC010P	vOTU37	0.62	0.025
Gammaproteobacteria	*Thiohalobacter*	Genus level	bOTU029	*Podoviridae*		Pelagibacter phage HTVC010P	vOTU37	0.62	0.023
Deltaproteobacteria	Class level	Class level	bOTU029	*Podoviridae*		Pelagibacter phage HTVC010P	vOTU37	0.64	0.017
Gammaproteobacteria	*Cognaticolwellia*	*Cognaticolwellia aestuarii*	bOTU029	*Podoviridae*		Pelagibacter phage HTVC010P	vOTU37	0.66	0.015
Gammaproteobacteria	*Cognaticolwellia*	Genus level	bOTU029	*Podoviridae*		Pelagibacter phage HTVC010P	vOTU37	0.66	0.014
Gammaproteobacteria	Class level	Class level	bOTU029	*Podoviridae*		Pelagibacter phage HTVC010P	vOTU37	0.68	0.011
Alphaproteobacteria	*Marinibaculum*	Genus level	bOTU029	*Podoviridae*		Pelagibacter phage HTVC010P	vOTU37	0.70	0.008
Alphaproteobacteria	*Novosphingobium*	Genus level	bOTU029	*Podoviridae*		Pelagibacter phage HTVC010P	vOTU37	0.71	0.007
Cytophagia	*Marinoscillum*	Genus level	bOTU029	*Podoviridae*		Pelagibacter phage HTVC010P	vOTU37	0.74	0.004
Cytophagia	Class level	Class level	bOTU029	*Podoviridae*		Pelagibacter phage HTVC010P	vOTU37	0.74	0.004
Gammaproteobacteria	*Colwellia*	Genus level	bOTU029	*Podoviridae*		Pelagibacter phage HTVC019P	vOTU38	0.56	0.046
Epsilonproteobacteria	Class level	Class level	bOTU029	*Podoviridae*		Pelagibacter phage HTVC019P	vOTU38	0.59	0.035
Flavobacteriia	Class level	Class level	bOTU029	*Podoviridae*		Pelagibacter phage HTVC019P	vOTU38	0.59	0.034
Flavobacteriia	*Winogradskyella*	Genus level	bOTU029	*Podoviridae*		Pelagibacter phage HTVC019P	vOTU38	0.60	0.032
Flavobacteriia	*Lacinutrix*	*Lacinutrix algicola*	bOTU029	*Podoviridae*		Pelagibacter phage HTVC019P	vOTU38	0.60	0.030
Gammaproteobacteria	*Cognaticolwellia*	Genus level	bOTU029	*Podoviridae*		Pelagibacter phage HTVC019P	vOTU38	0.61	0.028
Gammaproteobacteria	*Cognaticolwellia*	*Cognaticolwellia aestuarii*	bOTU029	*Podoviridae*		Pelagibacter phage HTVC019P	vOTU38	0.62	0.024
Flavobacteriia	*Algibacter*	*Algibacter miyuki*	bOTU029	*Podoviridae*		Pelagibacter phage HTVC019P	vOTU38	0.62	0.024
Flavobacteriia	*Algibacter*	Genus level	bOTU029	*Podoviridae*		Pelagibacter phage HTVC019P	vOTU38	0.62	0.024
Alphaproteobacteria	*Paraurantiacibacter*	Genus level	bOTU029	*Podoviridae*		Pelagibacter phage HTVC019P	vOTU38	0.63	0.022
Alphaproteobacteria	*Parasphingorhabdus*	*Parasphingorhabdus flavimaris*	bOTU029	*Podoviridae*		Pelagibacter phage HTVC019P	vOTU38	0.63	0.021
Alphaproteobacteria	*Parasphingorhabdus*	Genus level	bOTU029	*Podoviridae*		Pelagibacter phage HTVC019P	vOTU38	0.63	0.021
Flavobacteriia	*Cellulophaga*	Genus level	bOTU029	*Podoviridae*		Pelagibacter phage HTVC019P	vOTU38	0.64	0.019
Gammaproteobacteria	*Pseudomonas*	*Pseudomonas sabulinigri*	bOTU029	*Podoviridae*		Pelagibacter phage HTVC019P	vOTU38	0.65	0.017
Flavobacteriia	*Polaribacter*	Genus level	bOTU029	*Podoviridae*		Pelagibacter phage HTVC019P	vOTU38	0.68	0.010
Flavobacteriia	*Maribacter*	Genus level	bOTU029	*Podoviridae*		Pelagibacter phage HTVC019P	vOTU38	0.69	0.009

Flavobacteriia	*Polaribacter*	*Polaribacter staleyi*	bOTU029	*Podoviridae*		Pelagibacter phage HTVC019P	vOTU38	0.70	0.008
Flavobacteriia	*Polaribacter*	*Polaribacter haliotis*	bOTU029	*Podoviridae*		Pelagibacter phage HTVC019P	vOTU38	0.71	0.007
Gammaproteobacteria	*Psychromonas*	Genus level	bOTU029	*Podoviridae*		Pelagibacter phage HTVC019P	vOTU38	0.73	0.004
Gammaproteobacteria	*Azotobacter*	Genus level	bOTU029	*Podoviridae*	*Puniceispirillum* phage	Genus level	vOTU24	0.62	0.023
Gammaproteobacteria	*Thiohalobacter*	Genus level	bOTU029	*Podoviridae*		Puniceispirillum phage HMO-2011	vOTU39	0.56	0.048
Gammaproteobacteria	*Psychrobacter*	Genus level	bOTU029	*Podoviridae*		Puniceispirillum phage HMO-2011	vOTU39	0.58	0.037
Gammaproteobacteria	*Porticoccus*	*Porticoccus hydrocarbonoclasticus*	bOTU029	*Podoviridae*		Puniceispirillum phage HMO-2011	vOTU39	0.58	0.036
Gammaproteobacteria	*Porticoccus*	Genus level	bOTU029	*Podoviridae*		Puniceispirillum phage HMO-2011	vOTU39	0.58	0.036
Gammaproteobacteria	*Psychromonas*	Genus level	bOTU029	*Podoviridae*		Puniceispirillum phage HMO-2011	vOTU39	0.59	0.036
Flavobacteriia	*Polaribacter*	*Polaribacter haliotis*	bOTU029	*Podoviridae*		Puniceispirillum phage HMO-2011	vOTU39	0.59	0.032
Gammaproteobacteria	*Halomonas*	*Halomonas glaciei*	bOTU029	*Podoviridae*		Puniceispirillum phage HMO-2011	vOTU39	0.60	0.032
Gammaproteobacteria	*Halomonas*	Genus level	bOTU029	*Podoviridae*		Puniceispirillum phage HMO-2011	vOTU39	0.60	0.032
Flavobacteriia	*Polaribacter*	*Polaribacter staleyi*	bOTU029	*Podoviridae*		Puniceispirillum phage HMO-2011	vOTU39	0.61	0.026
Gammaproteobacteria	*Pseudomonas*	*Pseudomonas chloritidismutans*	bOTU029	*Podoviridae*		Puniceispirillum phage HMO-2011	vOTU39	0.62	0.025
Flavobacteriia	*Polaribacter*	Genus level	bOTU029	*Podoviridae*		Puniceispirillum phage HMO-2011	vOTU39	0.62	0.024
Gammaproteobacteria	*Azotobacter*	Genus level	bOTU029	*Podoviridae*		Puniceispirillum phage HMO-2011	vOTU39	0.62	0.023
Gammaproteobacteria	*Pseudoalteromonas*	Genus level	bOTU029	*Podoviridae*		Puniceispirillum phage HMO-2011	vOTU39	0.63	0.021
Gammaproteobacteria	*Pseudomonas*	*Pseudomonas sabulinigri*	bOTU029	*Podoviridae*		Puniceispirillum phage HMO-2011	vOTU39	0.64	0.019
Flavobacteriia	*Vicingus*	*Vicingus serpentipes*	bOTU029	*Podoviridae*		Puniceispirillum phage HMO-2011	vOTU39	0.64	0.019
Vicingus	Class level	Class level	bOTU029	*Podoviridae*		Puniceispirillum phage HMO-2011	vOTU39	0.64	0.019
Gammaproteobacteria	*Colwellia*	Genus level	bOTU029	*Podoviridae*		Puniceispirillum phage HMO-2011	vOTU39	0.64	0.019
Gammaproteobacteria	*Colwellia*	*Colwellia echini*	bOTU029	*Podoviridae*		Puniceispirillum phage HMO-2011	vOTU39	0.64	0.018
Gammaproteobacteria	*Pseudoalteromonas*	*Pseudoalteromonas hodoensis*	bOTU029	*Podoviridae*		Puniceispirillum phage HMO-2011	vOTU39	0.64	0.018
Gammaproteobacteria	*Marinobacterium*	Genus level	bOTU029	*Podoviridae*		Puniceispirillum phage HMO-2011	vOTU39	0.65	0.016
Gammaproteobacteria	*Pseudomonas*	Genus level	bOTU029	*Podoviridae*		Puniceispirillum phage HMO-2011	vOTU39	0.65	0.016
Gammaproteobacteria	*Parahaliea*	Genus level	bOTU029	*Podoviridae*		Puniceispirillum phage HMO-2011	vOTU39	0.66	0.014
Gammaproteobacteria	*Sinobacterium*	Genus level	bOTU029	*Podoviridae*		Puniceispirillum phage HMO-2011	vOTU39	0.68	0.010

Gammaproteobacteria	*Paraglaciecola*	*Paraglaciecola polaris*	bOTU029	*Podoviridae*		Puniceispirillum phage HMO-2011	vOTU39	0.68	0.010
Gammaproteobacteria	*Paraglaciecola*	Genus level	bOTU029	*Podoviridae*		Puniceispirillum phage HMO-2011	vOTU39	0.69	0.009
Gammaproteobacteria	*Cognaticolwellia*	*Cognaticolwellia aestuarii*	bOTU029	*Podoviridae*		Puniceispirillum phage HMO-2011	vOTU39	0.71	0.006
Gammaproteobacteria	*Cognaticolwellia*	Genus level	bOTU029	*Podoviridae*		Puniceispirillum phage HMO-2011	vOTU39	0.72	0.005
Planctomycetia	Class level	Class level	bOTU029	*Podoviridae*		Puniceispirillum phage HMO-2011	vOTU39	0.73	0.005
Alphaproteobacteria	*Magnetospira*	Genus level	bOTU029	*Podoviridae*		Puniceispirillum phage HMO-2011	vOTU39	0.74	0.004
Alphaproteobacteria	*Ahrensia*	Genus level	bOTU029	*Podoviridae*		Puniceispirillum phage HMO-2011	vOTU39	0.74	0.004
Gammaproteobacteria	*Eionea*	*Eionea flava*	bOTU029	*Podoviridae*		Puniceispirillum phage HMO-2011	vOTU39	0.76	0.002
Gammaproteobacteria	*Eionea*	Genus level	bOTU029	*Podoviridae*		Puniceispirillum phage HMO-2011	vOTU39	0.76	0.002
Epsilonproteobacteria	Class level	Class level	bOTU029	*Podoviridae*		Puniceispirillum phage HMO-2011	vOTU39	0.77	0.002
Cytophagia	*Marinoscillum*	Genus level	bOTU029	*Podoviridae*		Puniceispirillum phage HMO-2011	vOTU39	0.77	0.002
Gammaproteobacteria	*Pseudomonas*	*Pseudomonas stutzeri*	bOTU029	*Podoviridae*		Puniceispirillum phage HMO-2011	vOTU39	0.78	0.002
Gammaproteobacteria	*Maribrevibacterium*	Genus level	bOTU029	*Podoviridae*		Puniceispirillum phage HMO-2011	vOTU39	0.78	0.002
Gammaproteobacteria	*Thioprofundum*	Genus level	bOTU029	*Podoviridae*		Puniceispirillum phage HMO-2011	vOTU39	0.78	0.002
Alphaproteobacteria	*Marinibaculum*	Genus level	bOTU029	*Podoviridae*		Puniceispirillum phage HMO-2011	vOTU39	0.81	0.001
Alphaproteobacteria	*Amylibacter*	*Amylibacter cionae*	bOTU029	*Podoviridae*		Puniceispirillum phage HMO-2011	vOTU39	0.81	0.001
Alphaproteobacteria	*Amylibacter*	Genus level	bOTU029	*Podoviridae*		Puniceispirillum phage HMO-2011	vOTU39	0.82	0.001
Flavobacteriia	*Owenweeksia*	Genus level	bOTU029	*Podoviridae*		Puniceispirillum phage HMO-2011	vOTU39	0.82	0.001
Betaproteobacteria	Class level	Class level	bOTU029	*Podoviridae*		Puniceispirillum phage HMO-2011	vOTU39	0.82	0.001
Gammaproteobacteria	*Pseudohongiella*	Genus level	bOTU029	*Podoviridae*		Puniceispirillum phage HMO-2011	vOTU39	0.83	0.000
Flavobacteriia	*Aquibacter*	Genus level	bOTU029	*Podoviridae*		Puniceispirillum phage HMO-2011	vOTU39	0.83	0.000
Gammaproteobacteria	*Sedimenticola*	*Sedimenticola thiotaurini*	bOTU029	*Podoviridae*		Puniceispirillum phage HMO-2011	vOTU39	0.84	0.000
Gammaproteobacteria	*Sedimenticola*	Genus level	bOTU029	*Podoviridae*		Puniceispirillum phage HMO-2011	vOTU39	0.84	0.000
Gammaproteobacteria	*Moraxella*	*Moraxella oblonga*	bOTU029	*Podoviridae*		Puniceispirillum phage HMO-2011	vOTU39	0.85	0.000
Gammaproteobacteria	*Moraxella*	Genus level	bOTU029	*Podoviridae*		Puniceispirillum phage HMO-2011	vOTU39	0.85	0.000
Alphaproteobacteria	*Novosphingobium*	Genus level	bOTU029	*Podoviridae*		Puniceispirillum phage HMO-2011	vOTU39	0.87	0.000
Gammaproteobacteria	Class level	Class level	bOTU029	*Podoviridae*		Puniceispirillum phage HMO-2011	vOTU39	0.88	0.000

Cytophagia	Class level	Class level	bOTU029	*Podoviridae*		Puniceispirillum phage HMO-2011	vOTU39	0.89	0.000
Deltaproteobacteria	Class level	Class level	bOTU029	*Podoviridae*		Puniceispirillum phage HMO-2011	vOTU39	0.90	0.000
Flavobacteriia	*Polaribacter*	Genus level	bOTU029	*Podoviridae*		Vibrio phage CHOED	vOTU43	0.55	0.050
Flavobacteriia	Class level	Class level	bOTU029	*Podoviridae*		Vibrio phage CHOED	vOTU43	0.58	0.037
Flavobacteriia	*Algibacter*	*Algibacter miyuki*	bOTU029	*Podoviridae*		Vibrio phage CHOED	vOTU43	0.59	0.034
Flavobacteriia	*Algibacter*	Genus level	bOTU029	*Podoviridae*		Vibrio phage CHOED	vOTU43	0.59	0.034
Flavobacteriia	*Cellulophaga*	Genus level	bOTU029	*Podoviridae*		Vibrio phage CHOED	vOTU43	0.60	0.032
Flavobacteriia	*Lacinutrix*	*Lacinutrix algicola*	bOTU029	*Podoviridae*		Vibrio phage CHOED	vOTU43	0.67	0.012
Flavobacteriia	*Lacinutrix*	Genus level	bOTU029	*Podoviridae*		Vibrio phage CHOED	vOTU43	0.71	0.007
Gammaproteobacteria	*Psychrosphaera*	Genus level	bOTU029	*Podoviridae*		Vibrio phage CHOED	vOTU43	0.71	0.007
Flavobacteriia	*Maribacter*	Genus level	bOTU029	*Podoviridae*		Vibrio phage CHOED	vOTU43	0.76	0.002
Cyanophyceae	*Foliisarcina*	Genus level	bOTU029	*Siphoviridae*	*Cellulophaga* phage	Genus level	vOTU13	0.56	0.047
Alphaproteobacteria	*Loktanella*	*Loktanella acticola*	bOTU029	*Siphoviridae*	*Cellulophaga* phage	Genus level	vOTU13	0.70	0.008
Alphaproteobacteria	*Loktanella*	Genus level	bOTU029	*Siphoviridae*	*Cellulophaga* phage	Genus level	vOTU13	0.70	0.008
Acidimicrobiia	*Ilumatobacter*	*Ilumatobacter fluminis*	bOTU029	*Siphoviridae*	*Cellulophaga* phage	Genus level	vOTU13	0.73	0.004
Acidimicrobiia	*Ilumatobacter*	Genus level	bOTU029	*Siphoviridae*	*Cellulophaga* phage	Genus level	vOTU13	0.73	0.004
Actinomycetia	*Rhodoluna*	Genus level	bOTU029	*Siphoviridae*	*Flavobacterium* phage	Genus level	vOTU16	0.55	0.050
Cyanophyceae	*Foliisarcina*	Genus level	bOTU029	*Siphoviridae*	*Flavobacterium* phage	Genus level	vOTU16	0.57	0.043
Alphaproteobacteria	*Sulfitobacter*	*Sulfitobacter profundi*	bOTU029	*Siphoviridae*	*Flavobacterium* phage	Genus level	vOTU16	0.76	0.003
Alphaproteobacteria	*Sulfitobacter*	Genus level	bOTU029	*Siphoviridae*	*Flavobacterium* phage	Genus level	vOTU16	0.76	0.003
Alphaproteobacteria	Class level	Class level	bOTU029	*Siphoviridae*	*Flavobacterium* phage	Genus level	vOTU16	0.83	0.000
Acidimicrobiia	*Ilumatobacter*	*Ilumatobacter fluminis*	bOTU029	*Siphoviridae*	*Flavobacterium* phage	Genus level	vOTU16	0.87	0.000
Acidimicrobiia	*Ilumatobacter*	Genus level	bOTU029	*Siphoviridae*	*Flavobacterium* phage	Genus level	vOTU16	0.87	0.000
Alphaproteobacteria	*Loktanella*	*Loktanella acticola*	bOTU029	*Siphoviridae*	*Flavobacterium* phage	Genus level	vOTU16	0.88	0.000
Alphaproteobacteria	*Loktanella*	Genus level	bOTU029	*Siphoviridae*	*Flavobacterium* phage	Genus level	vOTU16	0.88	0.000
Gammaproteobacteria	*Shewanella*	Genus level	bOTU029	*Siphoviridae*	*Lactobacillus* phage	Genus level	vOTU17	0.85	0.000
Flavobacteriia	*Algibacter*	*Algibacter miyuki*	bOTU029	*Siphoviridae*	*Polaribacter* phage	Genus level	vOTU21	0.56	0.047
Flavobacteriia	*Algibacter*	Genus level	bOTU029	*Siphoviridae*	*Polaribacter* phage	Genus level	vOTU21	0.56	0.047

Flavobacteriia	Class level	Class level	bOTU029	*Siphoviridae*	*Polaribacter* phage	Genus level	vOTU21	0.56	0.046
Flavobacteriia	*Lacinutrix*	*Lacinutrix algicola*	bOTU029	*Siphoviridae*	*Polaribacter* phage	Genus level	vOTU21	0.59	0.032
Gammaproteobacteria	*Psychrosphaera*	Genus level	bOTU029	*Siphoviridae*	*Polaribacter* phage	Genus level	vOTU21	0.73	0.005
Flavobacteriia	*Lacinutrix*	Genus level	bOTU029	*Siphoviridae*	*Polaribacter* phage	Genus level	vOTU21	0.83	0.001
Gammaproteobacteria	*Porticoccus*	*Porticoccus hydrocarbonoclasticus*	bOTU029	*Siphoviridae*	*Roseobacter* phage	Genus level	vOTU26	0.57	0.044
Gammaproteobacteria	*Porticoccus*	Genus level	bOTU029	*Siphoviridae*	*Roseobacter* phage	Genus level	vOTU26	0.57	0.044
Alphaproteobacteria	*Ahrensia*	Genus level	bOTU029	*Siphoviridae*	*Roseobacter* phage	Genus level	vOTU26	0.57	0.043
Alphaproteobacteria	*Planktomarina*	Genus level	bOTU029	*Siphoviridae*	*Roseobacter* phage	Genus level	vOTU26	0.61	0.028
Gammaproteobacteria	*Sedimenticola*	*Sedimenticola thiotaurini*	bOTU029	*Siphoviridae*	*Roseobacter* phage	Genus level	vOTU26	0.61	0.027
Gammaproteobacteria	*Sedimenticola*	Genus level	bOTU029	*Siphoviridae*	*Roseobacter* phage	Genus level	vOTU26	0.61	0.027
Alphaproteobacteria	*Marinibaculum*	Genus level	bOTU029	*Siphoviridae*	*Roseobacter* phage	Genus level	vOTU26	0.62	0.025
Cytophagia	*Marinoscillum*	Genus level	bOTU029	*Siphoviridae*	*Roseobacter* phage	Genus level	vOTU26	0.62	0.025
Gammaproteobacteria	*Sinobacterium*	Genus level	bOTU029	*Siphoviridae*	*Roseobacter* phage	Genus level	vOTU26	0.68	0.010
Gammaproteobacteria	*Marinobacterium*	Genus level	bOTU029	*Siphoviridae*	*Roseobacter* phage	Genus level	vOTU26	0.70	0.008
Gammaproteobacteria	*Maribrevibacterium*	Genus level	bOTU027	*Siphoviridae*	*Roseobacter* phage	Genus level	vOTU26	0.71	0.006
Gammaproteobacteria	*Eionea*	*Eionea flava*	bOTU063	*Siphoviridae*	*Roseobacter* phage	Genus level	vOTU26	0.72	0.006
Gammaproteobacteria	*Eionea*	Genus level	bOTU018	*Siphoviridae*	*Roseobacter* phage	Genus level	vOTU26	0.72	0.006
Alphaproteobacteria	*Sphingomonas*	Genus level	bOTU053	*Siphoviridae*	*Inhavirus*	Nonlabens phage P12024S	vOTU36	0.56	0.047
Gammaproteobacteria	*Psychrobacter*	Genus level	bOTU046	*Siphoviridae*	*Inhavirus*	Nonlabens phage P12024S	vOTU36	0.57	0.044
Gammaproteobacteria	*Pseudomonas*	*Pseudomonas chloritidismutans*	bOTU078	*Siphoviridae*	*Inhavirus*	Nonlabens phage P12024S	vOTU36	0.57	0.043
Gammaproteobacteria	*Psychrobacter*	*Psychrobacter nivimaris*	bOTU079	*Siphoviridae*	*Inhavirus*	Nonlabens phage P12024S	vOTU36	0.69	0.009
Gammaproteobacteria	*Shewanella*	Genus level	bOTU051	*Siphoviridae*	*Morganella* phage	Genus level	vOTU100	0.74	0.004
Gammaproteobacteria	*Psychrobacter*	*Psychrobacter submarinus*	bOTU076	*Siphoviridae*	*Inhavirus*	Nonlabens phage P12024S	vOTU36	0.68	0.010

Table S5. Classification Information of operational taxonomic units (OTUs) of common bacteria and bacteriophage

Bacteria			
**Class**	**Genus**	**Species**	**OTU No.**
Alphaproteobacteria	Class level	Class level	bOTU001
Gammaproteobacteri a	Class level	Class level	bOTU002
Flavobacteriia	Class level	Class level	bOTU003
Betaproteobacteria	Class level	Class level	bOTU005
Cytophagia	Class level	Class level	bOTU006
Deltaproteobacteria	Class level	Class level	bOTU007
Planctomycetia	Class level	Class level	bOTU008
Epsilonproteobacteria	Class level	Class level	bOTU009
Alphaproteobacteria	*Ahrensia*	Genus level	bOTU010
Flavobacteriia	*Algibacter*	Genus level	bOTU011
Alphaproteobacteria	*Amylibacter*	Genus level	bOTU012
Flavobacteriia	*Aquibacter*	Genus level	bOTU013
Gammaproteobacteri a	*Azotobacter*	Genus level	bOTU014
Flavobacteriia	*Cellulophaga*	Genus level	bOTU015
Gammaproteobacteri a	*Cognaticolwellia*	Genus level	bOTU016
Gammaproteobacteri a	*Colwellia*	Genus level	bOTU017
Gammaproteobacteri a	*Eionea*	Genus level	bOTU018
Cyanobacteriota	*Foliisarcina*	Genus level	bOTU019
Gammaproteobacteri a	*Halomonas*	Genus level	bOTU020
Acidimicrobiia	*Ilumatobacter*	Genus level	bOTU021
Flavobacteriia	*Lacinutrix*	Genus level	bOTU022
Flavobacteriia	*Leeuwenhoekiella*	Genus level	bOTU023
Alphaproteobacteria	*Loktanella*	Genus level	bOTU024
Alphaproteobacteria	*Magnetospira*	Genus level	bOTU025
Flavobacteriia	*Maribacter*	Genus level	bOTU026
Flavobacteriia	*Maribrevibacterium*	Genus level	bOTU027
Alphaproteobacteria	*Marinibaculum*	Genus level	bOTU028
Gammaproteobacteri a	*Marinobacterium*	Genus level	bOTU029
Cytophagia	*Marinoscillum*	Genus level	bOTU030
Flavobacteriia	*Mesoflavibacter*	Genus level	bOTU031
Gammaproteobacteri a	*Methylophaga*	Genus level	bOTU032
Gammaproteobacteri a	*Moraxella*	Genus level	bOTU033
Alphaproteobacteria	*Novosphingobium*	Genus level	bOTU034

Bacteriophage			
**Family**	**Genus**	**Species**	**OTU No.**
Ackermannvirida e	Family level	Family level	vOTU01
Ampullaviridae	Family level	Family level	vOTU02
Bicaudaviridae	Family level	Family level	vOTU03
Herelleviridae	Family level	Family level	vOTU04
Inoviridae	Family level	Family level	vOTU05
Microviridae	Family level	Family level	vOTU06
Myoviridae	*Escherichia* phage	Genus level	vOTU07
Myoviridae	*Acinetobacter* phage	Genus level	vOTU08
Myoviridae	*Aeromonas* phage	Genus level	vOTU09
Myoviridae	*Agrobacterium* phage	Genus level	vOTU10
Herelleviridae	*Bacillus* phage	Genus level	vOTU11
Myoviridae	*Campylobacter* phage	Genus level	vOTU12
Siphoviridae	*Cellulophaga* phage	Genus level	vOTU13
Myoviridae	*Clostridium* phage	Genus level	vOTU14
Myoviridae	*Croceibacter* phage	Genus level	vOTU15
Siphoviridae	*Flavobacterium* phage	Genus level	vOTU16
Siphoviridae	*Lactobacillus* phage	Genus level	vOTU17
Siphoviridae	*Nonlabens* phage	Genus level	vOTU19
Podoviridae	*Nonlabens* phage	Genus level	vOTU20
Siphoviridae	*Polaribacter* phage	Genus level	vOTU21
Myoviridae	*Prochlorococcus* phage	Genus level	vOTU22
Podoviridae	*Pseudomonas* phage	Genus level	vOTU23
Podoviridae	*Puniceispirillum* phage	Genus level	vOTU24
Myoviridae	*Rhizobium* phage	Genus level	vOTU25
Siphoviridae	*Roseobacter* phage	Genus level	vOTU26
Ackermannvirida e	*Serratia* phage	Genus level	vOTU27
Myoviridae	*Sphingomonas* phage	Genus level	vOTU28
Myoviridae	*Staphylococcus* phage	Genus level	vOTU29
Myoviridae	*Synechococcus* phage	Genus level	vOTU30
Myoviridae	*Thermus* phage	Genus level	vOTU31
Myoviridae	*Vibrio* phage	Genus level	vOTU32
Myoviridae	*Yersinia* phage	Genus level	vOTU33
Myoviridae	*Ishigurovirus*	Aeromonas virus 65	vOTU34

Flavobacteriia	*Owenweeksia*	Genus level	bOTU035
Gammaproteobacteria	*Paraglaciecola*	Genus level	bOTU036
Gammaproteobacteria	*Parahaliea*	Genus level	bOTU037
Alphaproteobacteria	*Parasphingorhabdus*	Genus level	bOTU038
Alphaproteobacteria	*Paraurantiacibacter*	Genus level	bOTU039
Alphaproteobacteria	*Planktomarina*	Genus level	bOTU040
Flavobacteriia	*Polaribacter*	Genus level	bOTU041
Gammaproteobacteria	*Porticoccus*	Genus level	bOTU042
Gammaproteobacteria	*Pseudoalteromonas*	Genus level	bOTU043
Gammaproteobacteria	*Pseudohongiella*	Genus level	bOTU044
Gammaproteobacteria	*Pseudomonas*	Genus level	bOTU045
Gammaproteobacteria	*Psychrobacter*	Genus level	bOTU046
Gammaproteobacteria	*Psychromonas*	Genus level	bOTU047
Gammaproteobacteria	*Psychrosphaera*	Genus level	bOTU048
Actinomycetia	*Rhodoluna*	Genus level	bOTU049
Gammaproteobacteria	*Sedimenticola*	Genus level	bOTU050
Gammaproteobacteria	*Shewanella*	Genus level	bOTU051
Gammaproteobacteria	*Sinobacterium*	Genus level	bOTU052
Gammaproteobacteria	*Sphingomonas*	Genus level	bOTU053
Alphaproteobacteria	*Sulfitobacter*	Genus level	bOTU054
Gammaproteobacteria	*Thiohalobacter*	Genus level	bOTU055
Gammaproteobacteria	*Thioprofundum*	Genus level	bOTU056
Gammaproteobacteria	*Vibrio*	Genus level	bOTU057
Flavobacteriia	*Winogradskyella*	Genus level	bOTU059
Alphaproteobacteria	*Sulfitobacter*	*Sulfitobacter profundi*	bOTU060
Alphaproteobacteria	*Loktanella*	*Loktanella acticola*	bOTU061
Gammaproteobacteria	*Pseudomonas*	*Pseudomonas sabulinigri*	bOTU062
Gammaproteobacteria	*Eionea*	*Eionea flava*	bOTU063
Gammaproteobacteria	*Cognaticolwellia*	*Cognaticolwellia aestuarii*	bOTU064
Gammaproteobacteria	*Pseudomonas*	*Pseudomonas stutzeri*	bOTU065
Gammaproteobacteria	*Colwellia*	*Colwellia echini*	bOTU066
Flavobacteriia	*Lacinutrix*	*Lacinutrix algicola*	bOTU067
Gammaproteobacteria	*Halomonas*	*Halomonas glaciei*	bOTU068
Gammaproteobacteria	*Pseudoalteromonas*	*Pseudoalteromonas hodoensis*	bOTU069
Flavobacteriia	*Polaribacter*	*Polaribacter staleyi*	bOTU070

Podoviridae	*Callevirus*	Cellulophaga phage phi38:1	vOTU35
Siphoviridae	*Inhavirus*	Nonlabens phage P12024S	vOTU36
Podoviridae		Pelagibacter phage HTVC010P	vOTU37
Podoviridae	*Pelagivirus*	Pelagibacter phage HTVC019P	vOTU38
Podoviridae		Puniceispirillum phage HMO-2011	vOTU39
Myoviridae		Sphingomonas phage PAU	vOTU40
Myoviridae	*Lipsvirus*	Synechococcus phage S-SSM7	vOTU41
Myoviridae	*Cymopoleiavirus*	Synechococcus phage S-WAM2	vOTU42
Podoviridae		Vibrio phage CHOED	vOTU43
Myoviridae	*Eneladusvirus*	Yersinia phage fHe-Yen9-04	vOTU44
Herelleviridae	*Nitunavirus*	Bacillus phage phiNIT1	vOTU45
Herelleviridae	*Hopescreekvirus*	Lactobacillus phage LfeInf	vOTU46
Herelleviridae	*Kayvirus*	Staphylococcus phage S25-3	vOTU47
Myoviridae	*Metrivirus*	Acinetobacter phage vB_AbaM_ME3	vOTU48
Myoviridae	*Ceceduovirus*	Aeromonas phage CC2	vOTU49
Myoviridae	*Polybotosvirus*	Agrobacterium phage Atu_ph07	vOTU50
Myoviridae	*Nitunavirus*	Bacillus virus G	vOTU51
Myoviridae	*Firehammervirus*	Campylobacter virus CP21	vOTU52
Myoviridae	*Sherbrookevirus*	Clostridium phage phiCDHM13	vOTU53
Myoviridae	*Mimasvirus*	Cronobacter phage vB_CsaM_GAP32	vOTU54
Myoviridae	*Libanvirus*	Cyanophage P-RSM6	vOTU55
Myoviridae	*Aurunvirus*	Cyanophage S-TIM5	vOTU56
Myoviridae	*Vequintavirinae*	Cyanophage Syn30	vOTU57
Myoviridae	*Efquatrovirus*	Enterobacteria phage phi92	vOTU58
Myoviridae	*Vequintavirus*	Escherichia phage 121Q	vOTU59
Myoviridae	*Teseptimavirus*	Escherichia phage FV3	vOTU60
Myoviridae	*Mosugukvirus*	Pectobacterium bacteriophage PM2	vOTU61
Myoviridae		Pectobacterium phage CBB	vOTU62
Myoviridae		Pelagibacter phage HTVC008M	vOTU63
Myoviridae	*Salacisavirus*	Prochlorococcus phage P-SSM2	vOTU64
Myoviridae	*Palaemonvirus*	Prochlorococcus phage P-SSM7	vOTU65
Myoviridae	*Haifavirus*	Prochlorococcus phage P-TIM68	vOTU66
Myoviridae	*Kleczkowskavirus*	Rhizobium phage RHEph06	vOTU67
Myoviridae	*Eneladusvirus*	Serratia phage BF	vOTU68
Myoviridae	*Emdodecavirus*	Sinorhizobium phage phiM12	vOTU69

Alphaproteobacteria	*Parasphingorhabdus*	*Parasphingorhabdus flavimaris*	bOTU071
Flavobacteriia	*Polaribacter*	*Polaribacter haliotis*	bOTU072
Acidimicrobiia	*Ilumatobacter*	*Ilumatobacter fluminis*	bOTU073
Gammaproteobacteri a	*Paraglaciecola*	*Paraglaciecola polaris*	bOTU074
Gammaproteobacteri a	*Sedimenticola*	*Sedimenticola thiotaurini*	bOTU075
Gammaproteobacteri a	*Sedimenticola*	*Psychrobacter submarinus*	bOTU076
Alphaproteobacteria	*Amylibacter*	*Amylibacter cionae*	bOTU077
Gammaproteobacteri a	*Pseudomonas*	*Pseudomonas chloritidismutans*	bOTU078
Gammaproteobacteri a	*Psychrobacter*	*Psychrobacter nivimaris*	bOTU079
Flavobacteriia	*Algibacter*	*Algibacter miyuki*	bOTU080
Flavobacteriia	*Vicingus*	*Vicingus serpentipes*	bOTU081
Gammaproteobacteri a	*Porticoccus*	*Porticoccus hydrocarbonoclasticus*	bOTU082
Gammaproteobacteri a	*Moraxella*	*Moraxella oblonga*	bOTU083
Vicingus	Class level	Class level	bOTU084
Acidimicrobiia	Class level	Class level	bOTU085
Actinobacteria	*Actinomarinicola*	*Actinomarinicola tropica*	bOTU086
Actinobacteria	*Longivirga*	*Longivirga aurantiaca*	bOTU087
Actinobacteria	*Rhodoluna*	*Rhodoluna lacicola*	bOTU088
Actinobacteria	*Nocardioides*	*Nocardioides salarius*	bOTU089
Bacteroidetes	*Marinoscillum*	*Marinoscillum pacificum*	bOTU090
Bacteroidetes	*Aquibacter*	*Aquibacter zeaxanthinifaciens*	bOTU091
Bacteroidetes	*Cellulophaga*	*Cellulophaga algicola*	bOTU092
Bacteroidetes	*Lacinutrix*	*Lacinutrix gracilariae*	bOTU093
Bacteroidetes	*Leeuwenhoekiella*	*Leeuwenhoekiella aequorea*	bOTU094
Bacteroidetes	*Maribacter*	*Maribacter aquivivus*	bOTU095
Bacteroidetes	*Mesoflavibacter*	*Mesoflavibacter sabulilitoris*	bOTU096
Bacteroidetes	*Mesoflavibacter*	*Mesoflavibacter zeaxanthinifaciens*	bOTU097
Bacteroidetes	*Owenweeksia*	*Owenweeksia hongkongensis*	bOTU098
Bacteroidetes	*Phaeocystidibacter*	*Phaeocystidibacter marisrubri*	bOTU099
Bacteroidetes	*Polaribacter*	*Polaribacter atrinae*	bOTU100
Bacteroidetes	*Winogradskyella*	*Winogradskyella litoriviva*	bOTU101
Bacteroidetes	*Winogradskyella*	*Winogradskyella psychrotolerans*	bOTU102
Cyanobacteria	*Foliisarcina*	*Foliisarcina bertiogensis*	bOTU103
Proteobacteria	*Ahrensia*	*Ahrensia marina*	bOTU104
Proteobacteria	*Amylibacter*	*Amylibacter ulvae*	bOTU105
Proteobacteria	*Emcibacter*	*Emcibacter nanhaiensis*	bOTU106

Myoviridae	*Lowelvirus*	Synechococcus phage ACG-2014d	vOTU70
Myoviridae	*Atlauavirus*	Synechococcus phage ACG-2014f	vOTU71
Myoviridae	*Sedonavirus*	Synechococcus phage ACG-2014h	vOTU72
Myoviridae	*Anaposvirus*	Synechococcus phage S-CAM1	vOTU73
Myoviridae	*Cymopoleiavirus*	Synechococcus phage S-RSM4	vOTU74
Myoviridae	*Llyrvirus*	Synechococcus phage S-SKS1	vOTU75
Myoviridae		Thermus phage TMA	vOTU76
Podoviridae	*Siovirus*	Celeribacter phage P12053L	vOTU77
Podoviridae	*Akihdevirus*	Cellulophaga phage phi14:2	vOTU78
Podoviridae	*Baltivirus*	Cellulophaga phage phi18:3	vOTU79
Podoviridae	*Baltivirus*	Cellulophaga phage phi19:3	vOTU80
Podoviridae		Escherichia phage T7	vOTU81
Podoviridae	*Minipunavirus*	Morganella phage vB_MmoP_MP2	vOTU82
Podoviridae	*Stopavirus*	Pelagibacter phage HTVC011P	vOTU83
Podoviridae	*Lauvirus*	Podovirus Lau218	vOTU84
Podoviridae	*Lingvirus*	Prochlorococcus phage P-GSP1	vOTU85
Podoviridae	*Phutvirus*	Pseudomonas phage PPpW-4	vOTU86
Podoviridae	*Siovirus*	Roseobacter virus SIO1	vOTU87
Podoviridae		Thalassomonas phage BA3	vOTU88
Podoviridae	*Chatterjeevirus*	Vibrio phage ICP3	vOTU89
Siphoviridae	*Cebadecemvirus*	Cellulophaga phage phi10:1	vOTU90
Siphoviridae		Clostridium phage phiCD211	vOTU91
Siphoviridae		Clostridium phage vB_CpeS-CP51	vOTU92
Siphoviridae		Croceibacter phage P2559Y	vOTU93
Siphoviridae		Cronobacter phage ENT47670	vOTU94
Siphoviridae	*Asteriusvirus*	Enterococcus phage EfaCPT1	vOTU95
Siphoviridae		Flavobacterium phage 11b	vOTU96
Siphoviridae	*Inhavirus*	Nonlabens phage P12024L	vOTU97
Siphoviridae	*Incheonvrus*	Polaribacter phage P12002L	vOTU98
Siphoviridae		Synechococcus phage S-CBS2	vOTU99

Proteobacteria	*Magnetospira*	*Magnetospira thiophila*	bOTU107
Proteobacteria	*Marinibaculum*	*Marinibaculum pumilum*	bOTU108
Proteobacteria	*Novosphingobium*	*Novosphingobium fuchskuhlense*	bOTU109
Proteobacteria	*Paraurantiacibacter*	*Paraurantiacibacter namhicola*	bOTU110
Proteobacteria	*Planktomarina*	*Planktomarina temperata*	bOTU111
Proteobacteria	*Sphingomonas*	*Sphingomonas spermidinifaciens*	bOTU112
Proteobacteria	*Sulfitobacter*	*Sulfitobacter litoralis*	bOTU113
Proteobacteria	*Azotobacter*	*Azotobacter chroococcum*	bOTU114
Proteobacteria	*Cognaticolwellia*	*Cognaticolwellia sediminilitoris*	bOTU115
Proteobacteria	*Colwellia*	*Colwellia piezophila*	bOTU116
Proteobacteria	*Colwellia*	*Colwellia psychrerythraea*	bOTU117
Proteobacteria	*Colwellia*	*Colwellia rossensis*	bOTU118
Proteobacteria	*Maribrevibacterium*	*Maribrevibacterium harenarium*	bOTU119
Proteobacteria	*Marimicrobium*	*Marimicrobium arenosum*	bOTU120
Proteobacteria	*Marinobacterium*	*Marinobacterium boryeongense*	bOTU121
Proteobacteria	*Methylophaga*	*Methylophaga lonarensis*	bOTU122
Proteobacteria	*Methylophaga*	*Methylophaga nitratireducenticrescens*	bOTU123
Proteobacteria	*Paraglaciecola*	*Paraglaciecola hydrolytica*	bOTU124
Proteobacteria	*Parahaliea*	*Parahaliea maris*	bOTU125
Proteobacteria	*Pseudoalteromonas*	*Pseudoalteromonas piratica*	bOTU126
Proteobacteria	*Pseudohongiella*	*Pseudohongiella acticola*	bOTU127
Proteobacteria	*Pseudomonas*	*Pseudomonas salina*	bOTU128
Proteobacteria	*Psychromonas*	*Psychromonas aquatilis*	bOTU129
Proteobacteria	*Psychrosphaera*	*Psychrosphaera aquimarina*	bOTU130
Proteobacteria	*Sedimenticola*	*Sedimenticola selenatireducens*	bOTU131
Proteobacteria	*Shewanella*	*Shewanella japonica*	bOTU132
Proteobacteria	*Sinobacterium*	*Sinobacterium norvegicum*	bOTU133
Proteobacteria	*Thiohalobacter*	*Thiohalobacter thiocyanaticus*	bOTU134
Proteobacteria	*Thioprofundum*	*Thioprofundum lithotrophicum*	bOTU135
Proteobacteria	*Vibrio*	*Vibrio cidicii*	bOTU136

Table S6. The significant results of Local similarity correlations (LSA) in the Network Analysis in Figure 6.

Source Node				Target Node				LS	Xs	Ys	Len	SPCC	Pspcc	*p*-vlaue	*q*-value
OTU No.	Family	Genus	Species	OTU No.	Class	Genus	Species								
vOTU05	*Inoviridae*	Family level	Family level	pOTU01	*Alphaproteobacteria*	Class level	Class level	0.735075	11	11	3	0.6278	0.0216	0.0490	0.0456
vOTU10	*Myoviridae*	*Agrobacterium* phage	Genus level	pOTU01	*Alphaproteobacteria*	Class level	Class level	1.107433	9	9	5	0.9420	0.0000	0.0020	0.0066
vOTU15	*Myoviridae*	*Croceibacter* phage	Genus level	pOTU01	*Alphaproteobacteria*	Class level	Class level	-0.909939	3	3	11	-0.6842	0.0099	0.0440	0.0425
vOTU16	*Siphoviridae*	*Flavobacterium* phage	Genus level	pOTU01	*Alphaproteobacteria*	Class level	Class level	1.085026	9	9	5	0.8431	0.0003	0.0030	0.0082
vOTU25	*Myoviridae*	*Rhizobium* phage	Genus level	pOTU01	*Alphaproteobacteria*	Class level	Class level	0.912286	9	9	5	0.5722	0.0410	0.0290	0.0330
vOTU28	*Myoviridae*	*Sphingomonas* phage	Genus level	pOTU01	*Alphaproteobacteria*	Class level	Class level	-0.640499	1	1	13	-0.7322	0.0044	0.0190	0.0257
vOTU30	*Myoviridae*	*Synechococcus* phage	Genus level	pOTU01	*Alphaproteobacteria*	Class level	Class level	0.636348	5	5	9	0.5869	0.0350	0.0190	0.0257
vOTU35	*Podoviridae*		Cellulophaga phage phi38:1	pOTU01	*Alphaproteobacteria*	Class level	Class level	0.583024	4	4	10	0.4573	0.1161	0.0300	0.0337
vOTU38	*Podoviridae*		Pelagibacter phage HTVC019P	pOTU01	*Alphaproteobacteria*	Class level	Class level	-0.575041	2	2	12	-0.6677	0.0126	0.0350	0.0372
vOTU40	*Myoviridae*		Sphingomonas phage PAU	pOTU01	*Alphaproteobacteria*	Class level	Class level	-0.640499	1	1	13	-0.7322	0.0044	0.0150	0.0221
vOTU41	*Myoviridae*		Synechococcus phage S-SSM7	pOTU01	*Alphaproteobacteria*	Class level	Class level	0.55004	1	1	13	0.8473	0.0003	0.0000	0.0000
vOTU42	*Myoviridae*		Synechococcus phage S-WAM2	pOTU01	*Alphaproteobacteria*	Class level	Class level	-0.635087	3	3	11	-0.7271	0.0049	0.0130	0.0202
vOTU44	*Myoviridae*		Yersinia phage fHe-Yen9-04	pOTU01	*Alphaproteobacteria*	Class level	Class level	-0.570439	1	1	13	-0.6491	0.0164	0.0470	0.0444
vOTU04	*Herelleviridae*	Family level	Family level	pOTU02	*Gammaproteobacteria*	Class level	Class level	0.579752	1	1	13	0.4990	0.0826	0.0360	0.0378
vOTU07	*Myoviridae*	*Escherichia* phage	Genus level	pOTU02	*Gammaproteobacteria*	Class level	Class level	0.550217	2	2	12	0.5747	0.0399	0.0460	0.0438
vOTU09	*Myoviridae*	*Aeromonas* phage	Genus level	pOTU02	*Gammaproteobacteria*	Class level	Class level	0.603671	1	1	13	0.5864	0.0352	0.0160	0.0231
vOTU10	*Myoviridae*	*Agrobacterium* phage	Genus level	pOTU02	*Gammaproteobacteria*	Class level	Class level	-1.020431	9	9	5	-0.8756	0.0001	0.0060	0.0124
vOTU11	*Herelleviridae*	*Bacillus* phage	Genus level	pOTU02	*Gammaproteobacteria*	Class level	Class level	0.6344	1	1	12	0.5276	0.0639	0.0200	0.0265
vOTU13	*Siphoviridae*	*Cellulophaga* phage	Genus level	pOTU02	*Gammaproteobacteria*	Class level	Class level	-0.612636	1	1	13	-0.7829	0.0016	0.0310	0.0343
vOTU16	*Siphoviridae*	*Flavobacterium* phage	Genus level	pOTU02	*Gammaproteobacteria*	Class level	Class level	-1.152692	9	9	5	-0.8334	0.0004	0.0010	0.0043
vOTU22	*Myoviridae*	*Prochlorococcus* phage	Genus level	pOTU02	*Gammaproteobacteria*	Class level	Class level	-0.72693	1	1	13	-0.6035	0.0290	0.0090	0.0159
vOTU24	*Podoviridae*	*Puniceispirillum* phage	Genus level	pOTU02	*Gammaproteobacteria*	Class level	Class level	0.771184	1	1	13	0.8191	0.0006	0.0010	0.0043
vOTU25	*Myoviridae*	*Rhizobium* phage	Genus level	pOTU02	*Gammaproteobacteria*	Class level	Class level	-1.048684	9	9	5	-0.6588	0.0143	0.0010	0.0043
vOTU29	*Myoviridae*	*Staphylococcus* phage	Genus level	pOTU02	*Gammaproteobacteria*	Class level	Class level	-0.84944	9	9	5	-0.6457	0.0171	0.0210	0.0272
vOTU33	*Myoviridae*	*Yersinia* phage	Genus level	pOTU02	*Gammaproteobacteria*	Class level	Class level	0.701328	1	1	13	0.6813	0.0103	0.0070	0.0137

vOTU34	*Myoviridae*		Aeromonas virus 65	pOTU02	*Gammaproteobacteria*	Class level	Class level	0.449033	1	1	13	0.5694	0.0422	0.0280	0.0324
vOTU37	*Podoviridae*		Pelagibacter phage HTVC010P	pOTU02	*Gammaproteobacteria*	Class level	Class level	0.617882	1	1	13	0.5973	0.0311	0.0300	0.0337
vOTU39	*Podoviridae*		Puniceispirillum phage HMO-2011	pOTU02	*Gammaproteobacteria*	Class level	Class level	0.771184	1	1	13	0.8191	0.0006	0.0020	0.0066
vOTU41	*Myoviridae*		Synechococcus phage S-SSM7	pOTU02	*Gammaproteobacteria*	Class level	Class level	-0.558565	1	1	13	-0.8352	0.0004	0.0000	0.0000
vOTU44	*Myoviridae*		Yersinia phage fHe-Yen9-04	pOTU02	*Gammaproteobacteria*	Class level	Class level	0.701328	1	1	13	0.6813	0.0103	0.0120	0.0192
vOTU03	*Bicaudaviridae*	Family level	Family level	pOTU03	*Flavobacteriia*	Class level	Class level	0.468423	1	1	13	0.6080	0.0275	0.0100	0.0171
vOTU07	*Myoviridae*	*Escherichia* phage	Genus level	pOTU03	*Flavobacteriia*	Class level	Class level	0.619641	1	1	13	0.5498	0.0516	0.0170	0.0241
vOTU10	*Myoviridae*	*Agrobacterium* phage	Genus level	pOTU03	*Flavobacteriia*	Class level	Class level	-0.923222	9	9	5	-0.6799	0.0106	0.0240	0.0296
vOTU16	*Siphoviridae*	*Flavobacterium* phage	Genus level	pOTU03	*Flavobacteriia*	Class level	Class level	-1.052336	9	9	5	-0.7628	0.0024	0.0030	0.0082
vOTU21	*Siphoviridae*	*Polaribacter* phage	Genus level	pOTU03	*Flavobacteriia*	Class level	Class level	0.454664	1	1	12	0.5514	0.0508	0.0280	0.0324
vOTU25	*Myoviridae*	*Rhizobium* phage	Genus level	pOTU03	*Flavobacteriia*	Class level	Class level	-0.899021	9	9	3	-0.5669	0.0433	0.0210	0.0272
vOTU30	*Myoviridae*	*Synechococcus* phage	Genus level	pOTU03	*Flavobacteriia*	Class level	Class level	-0.726542	5	5	9	-0.7409	0.0038	0.0040	0.0097
vOTU33	*Myoviridae*	*Yersinia* phage	Genus level	pOTU03	*Flavobacteriia*	Class level	Class level	0.663041	4	4	10	0.6015	0.0297	0.0140	0.0211
vOTU35	*Podoviridae*		Cellulophaga phage phi38:1	pOTU03	*Flavobacteriia*	Class level	Class level	-0.725625	1	1	13	-0.6982	0.0079	0.0090	0.0159
vOTU41	*Myoviridae*		Synechococcus phage S-SSM7	pOTU03	*Flavobacteriia*	Class level	Class level	-0.483599	4	4	10	-0.6017	0.0296	0.0110	0.0182
vOTU42	*Myoviridae*		Synechococcus phage S-WAM2	pOTU03	*Flavobacteriia*	Class level	Class level	0.757005	1	1	13	0.7763	0.0018	0.0020	0.0066
vOTU43	*Podoviridae*		Vibrio phage CHOED	pOTU03	*Flavobacteriia*	Class level	Class level	0.637354	1	1	11	0.5979	0.0309	0.0130	0.0202
vOTU44	*Myoviridae*		Yersinia phage fHe-Yen9-04	pOTU03	*Flavobacteriia*	Class level	Class level	0.663041	4	4	10	0.6015	0.0297	0.0160	0.0231
vOTU06	*Microviridae*	Family level	Family level	pOTU05	*Betaproteobacteria*	Class level	Class level	0.930541	2	2	11	0.4890	0.0899	0.0270	0.0317
vOTU09	*Myoviridae*	*Aeromonas* phage	Genus level	pOTU05	*Betaproteobacteria*	Class level	Class level	0.621585	1	1	13	0.5836	0.0363	0.0150	0.0221
vOTU10	*Myoviridae*	*Agrobacterium* phage	Genus level	pOTU05	*Betaproteobacteria*	Class level	Class level	-1.020431	9	9	5	-0.8208	0.0006	0.0100	0.0171
vOTU11	*Herelleviridae*	*Bacillus* phage	Genus level	pOTU05	*Betaproteobacteria*	Class level	Class level	0.620769	1	1	12	0.4760	0.1001	0.0280	0.0324
vOTU13	*Siphoviridae*	*Cellulophaga* phage	Genus level	pOTU05	*Betaproteobacteria*	Class level	Class level	-0.685892	1	1	13	-0.7848	0.0015	0.0050	0.0110
vOTU16	*Siphoviridae*	*Flavobacterium* phage	Genus level	pOTU05	*Betaproteobacteria*	Class level	Class level	-1.152692	9	9	5	-0.8178	0.0006	0.0000	0.0000
vOTU22	*Myoviridae*	*Prochlorococcus* phage	Genus level	pOTU05	*Betaproteobacteria*	Class level	Class level	-0.687555	1	1	13	-0.5690	0.0424	0.0070	0.0137
vOTU24	*Podoviridae*	*Puniceispirillum* phage	Genus level	pOTU05	*Betaproteobacteria*	Class level	Class level	0.728648	1	1	13	0.7949	0.0012	0.0050	0.0110
vOTU25	*Myoviridae*	*Rhizobium* phage	Genus level	pOTU05	*Betaproteobacteria*	Class level	Class level	-1.048684	9	9	5	-0.6489	0.0164	0.0010	0.0043
vOTU29	*Myoviridae*	*Staphylococcus* phage	Genus level	pOTU05	*Betaproteobacteria*	Class level	Class level	-0.84944	9	9	5	-0.6599	0.0141	0.0220	0.0280
vOTU33	*Myoviridae*	*Yersinia* phage	Genus level	pOTU05	*Betaproteobacteria*	Class level	Class level	0.723904	1	1	13	0.6544	0.0152	0.0030	0.0082

vOTU39	*Podoviridae*		Puniceispirillum phage HMO-2011	pOTU05	*Betaproteobacteria*	Class level	Class level	0.728648	1	1	13	0.7949	0.0012	0.0020	0.0066
vOTU41	*Myoviridae*		Synechococcus phage S-SSM7	pOTU05	*Betaproteobacteria*	Class level	Class level	-0.476315	1	1	13	-0.7587	0.0026	0.0060	0.0124
vOTU42	*Myoviridae*		Synechococcus phage S-WAM2	pOTU05	*Betaproteobacteria*	Class level	Class level	0.565814	2	2	12	0.6586	0.0144	0.0420	0.0413
vOTU44	*Myoviridae*		Yersinia phage fHe-Yen9-04	pOTU05	*Betaproteobacteria*	Class level	Class level	0.723904	1	1	13	0.6544	0.0152	0.0050	0.0110
vOTU09	*Myoviridae*	*Aeromonas* phage	Genus level	pOTU06	*Cytophagia*	Class level	Class level	0.532965	1	1	13	0.6387	0.0188	0.0490	0.0456
vOTU10	*Myoviridae*	*Agrobacterium* phage	Genus level	pOTU06	*Cytophagia*	Class level	Class level	-0.974821	9	9	5	-0.7315	0.0045	0.0200	0.0265
vOTU11	*Herelleviridae*	*Bacillus* phage	Genus level	pOTU06	*Cytophagia*	Class level	Class level	0.660649	1	1	12	0.4628	0.1113	0.0160	0.0231
vOTU13	*Siphoviridae*	*Cellulophaga* phage	Genus level	pOTU06	*Cytophagia*	Class level	Class level	-0.625451	1	1	13	-0.7667	0.0022	0.0170	0.0241
vOTU16	*Siphoviridae*	*Flavobacterium* phage	Genus level	pOTU06	*Cytophagia*	Class level	Class level	-1.114664	9	9	5	-0.7840	0.0015	0.0000	0.0000
vOTU22	*Myoviridae*	*Prochlorococcus* phage	Genus level	pOTU06	*Cytophagia*	Class level	Class level	-0.650805	1	1	13	-0.5521	0.0504	0.0190	0.0257
vOTU24	*Podoviridae*	*Puniceispirillum* phage	Genus level	pOTU06	*Cytophagia*	Class level	Class level	0.792425	1	1	13	0.8166	0.0007	0.0010	0.0043
vOTU25	*Myoviridae*	*Rhizobium* phage	Genus level	pOTU06	*Cytophagia*	Class level	Class level	-1.005543	9	9	5	-0.6823	0.0102	0.0080	0.0148
vOTU29	*Myoviridae*	*Staphylococcus* phage	Genus level	pOTU06	*Cytophagia*	Class level	Class level	-0.806299	9	9	5	-0.6995	0.0078	0.0480	0.0450
vOTU33	*Myoviridae*	*Yersinia* phage	Genus level	pOTU06	*Cytophagia*	Class level	Class level	0.792676	1	1	13	0.7082	0.0068	0.0020	0.0066
vOTU37	*Podoviridae*		Pelagibacter phage HTVC010P	pOTU06	*Cytophagia*	Class level	Class level	0.711214	1	1	13	0.6363	0.0194	0.0130	0.0202
vOTU39	*Podoviridae*		Puniceispirillum phage HMO-2011	pOTU06	*Cytophagia*	Class level	Class level	0.792425	1	1	13	0.8166	0.0007	0.0000	0.0000
vOTU41	*Myoviridae*		Synechococcus phage S-SSM7	pOTU06	*Cytophagia*	Class level	Class level	-0.500467	1	1	13	-0.7507	0.0031	0.0020	0.0066
vOTU44	*Myoviridae*		Yersinia phage fHe-Yen9-04	pOTU06	*Cytophagia*	Class level	Class level	0.792676	1	1	13	0.7082	0.0068	0.0010	0.0043
vOTU05	*Inoviridae*	Family level	Family level	pOTU07	*Deltaproteobacteria*	Class level	Class level	-0.945446	11	11	3	-0.8251	0.0005	0.0080	0.0148
vOTU06	*Microviridae*	Family level	Family level	pOTU07	*Deltaproteobacteria*	Class level	Class level	0.942529	2	2	11	0.4500	0.1229	0.0210	0.0272
vOTU09	*Myoviridae*	*Aeromonas* phage	Genus level	pOTU07	*Deltaproteobacteria*	Class level	Class level	0.572359	1	1	13	0.6229	0.0230	0.0320	0.0351
vOTU10	*Myoviridae*	*Agrobacterium* phage	Genus level	pOTU07	*Deltaproteobacteria*	Class level	Class level	-1.145233	9	9	5	-0.9175	0.0000	0.0010	0.0043
vOTU11	*Herelleviridae*	*Bacillus* phage	Genus level	pOTU07	*Deltaproteobacteria*	Class level	Class level	0.561114	1	1	12	0.4854	0.0927	0.0430	0.0419
vOTU13	*Siphoviridae*	*Cellulophaga* phage	Genus level	pOTU07	*Deltaproteobacteria*	Class level	Class level	-0.614442	1	1	13	-0.6851	0.0098	0.0150	0.0221
vOTU16	*Siphoviridae*	*Flavobacterium* phage	Genus level	pOTU07	*Deltaproteobacteria*	Class level	Class level	-0.9856	9	9	5	-0.6616	0.0138	0.0100	0.0171
vOTU22	*Myoviridae*	*Prochlorococcus* phage	Genus level	pOTU07	*Deltaproteobacteria*	Class level	Class level	-0.741119	1	1	13	-0.8662	0.0001	0.0020	0.0066
vOTU24	*Podoviridae*	*Puniceispirillum* phage	Genus level	pOTU07	*Deltaproteobacteria*	Class level	Class level	0.754976	1	1	13	0.8011	0.0010	0.0030	0.0082
vOTU27	*Ackermannviridae*	*Serratia* phage	Genus level	pOTU07	*Deltaproteobacteria*	Class level	Class level	-0.858517	9	9	5	-0.5045	0.0787	0.0350	0.0372
vOTU33	*Myoviridae*	*Yersinia* phage	Genus level	pOTU07	*Deltaproteobacteria*	Class level	Class level	0.638379	1	1	13	0.5495	0.0517	0.0210	0.0272

vOTU34	*Myoviridae*		Aeromonas virus 65	pOTU07	*Deltaproteobacteria*	Class level	Class level	0.418275	1	1	13	0.6788	0.0107	0.0350	0.0372
vOTU39	*Podoviridae*		Puniceispirillum phage HMO-2011	pOTU07	*Deltaproteobacteria*	Class level	Class level	0.754976	1	1	13	0.8011	0.0010	0.0020	0.0066
vOTU41	*Myoviridae*		Synechococcus phage S-SSM7	pOTU07	*Deltaproteobacteria*	Class level	Class level	-0.394784	1	1	13	-0.6541	0.0153	0.0300	0.0337
vOTU44	*Myoviridae*		Yersinia phage fHe-Yen9-04	pOTU07	*Deltaproteobacteria*	Class level	Class level	0.638379	1	1	13	0.5495	0.0517	0.0130	0.0202
vOTU05	*Inoviridae*	Family level	Family level	pOTU08	*Planctomycetia*	Class level	Class level	-0.675907	11	11	3	-0.7334	0.0043	0.0140	0.0211
vOTU07	*Myoviridae*	*Escherichia* phage	Genus level	pOTU08	*Planctomycetia*	Class level	Class level	0.433134	1	1	13	0.5472	0.0529	0.0240	0.0296
vOTU10	*Myoviridae*	*Agrobacterium* phage	Genus level	pOTU08	*Planctomycetia*	Class level	Class level	-0.873926	9	9	5	-0.9375	0.0000	0.0000	0.0000
vOTU13	*Siphoviridae*	*Cellulophaga* phage	Genus level	pOTU08	*Planctomycetia*	Class level	Class level	-0.518406	2	2	12	-0.8266	0.0005	0.0040	0.0097
vOTU16	*Siphoviridae*	*Flavobacterium* phage	Genus level	pOTU08	*Planctomycetia*	Class level	Class level	-0.853493	9	9	5	-0.8281	0.0005	0.0020	0.0066
vOTU20	*Podoviridae*	*Nonlabens* phage	Genus level	pOTU08	*Planctomycetia*	Class level	Class level	0.272801	4	4	9	0.4687	0.1062	0.0460	0.0438
vOTU24	*Podoviridae*	*Puniceispirillum* phage	Genus level	pOTU08	*Planctomycetia*	Class level	Class level	0.50105	6	6	8	0.7914	0.0013	0.0070	0.0137
vOTU25	*Myoviridae*	*Rhizobium* phage	Genus level	pOTU08	*Planctomycetia*	Class level	Class level	-0.674384	9	9	5	-0.4083	0.1661	0.0150	0.0221
vOTU27	*Ackermannviridae*	*Serratia* phage	Genus level	pOTU08	*Planctomycetia*	Class level	Class level	-0.698145	9	9	5	-0.4506	0.1223	0.0270	0.0317
vOTU33	*Myoviridae*	*Yersinia* phage	Genus level	pOTU08	*Planctomycetia*	Class level	Class level	0.498659	4	4	10	0.6261	0.0221	0.0090	0.0159
vOTU35	*Podoviridae*		Cellulophaga phage phi38:1	pOTU08	*Planctomycetia*	Class level	Class level	-0.524195	1	1	13	-0.5784	0.0384	0.0030	0.0082
vOTU39	*Podoviridae*		Puniceispirillum phage HMO-2011	pOTU08	*Planctomycetia*	Class level	Class level	0.50105	6	6	8	0.7914	0.0013	0.0060	0.0124
vOTU41	*Myoviridae*		Synechococcus phage S-SSM7	pOTU08	*Planctomycetia*	Class level	Class level	-0.286714	4	4	10	-0.6465	0.0169	0.0420	0.0413
vOTU42	*Myoviridae*		Synechococcus phage S-WAM2	pOTU08	*Planctomycetia*	Class level	Class level	0.429854	1	1	13	0.7580	0.0027	0.0230	0.0288
vOTU44	*Myoviridae*		Yersinia phage fHe-Yen9-04	pOTU08	*Planctomycetia*	Class level	Class level	0.498659	4	4	10	0.6261	0.0221	0.0090	0.0159
vOTU05	*Inoviridae*	Family level	Family level	pOTU09	*Epsilonproteobacteria*	Class level	Class level	-0.945446	11	11	3	-0.9288	0.0000	0.0090	0.0159
vOTU10	*Myoviridae*	*Agrobacterium* phage	Genus level	pOTU09	*Epsilonproteobacteria*	Class level	Class level	-1.083628	10	10	4	-0.8351	0.0004	0.0040	0.0097
vOTU16	*Siphoviridae*	*Flavobacterium* phage	Genus level	pOTU09	*Epsilonproteobacteria*	Class level	Class level	-0.851348	10	10	4	-0.4938	0.0863	0.0330	0.0358
vOTU22	*Myoviridae*	*Prochlorococcus* phage	Genus level	pOTU09	*Epsilonproteobacteria*	Class level	Class level	-0.762575	1	1	13	-0.8682	0.0001	0.0040	0.0097
vOTU24	*Podoviridae*	*Puniceispirillum* phage	Genus level	pOTU09	*Epsilonproteobacteria*	Class level	Class level	0.656167	1	1	13	0.5583	0.0474	0.0080	0.0148
vOTU32	*Myoviridae*	*Vibrio* phage	Genus level	pOTU09	*Epsilonproteobacteria*	Class level	Class level	0.53238	3	3	11	0.5106	0.0746	0.0450	0.0432
vOTU33	*Myoviridae*	*Yersinia* phage	Genus level	pOTU09	*Epsilonproteobacteria*	Class level	Class level	0.632072	1	1	13	0.4045	0.1704	0.0230	0.0288
vOTU38	*Podoviridae*		Pelagibacter phage HTVC019P	pOTU09	*Epsilonproteobacteria*	Class level	Class level	0.612929	2	2	12	0.6926	0.0087	0.0170	0.0241
vOTU39	*Podoviridae*		Puniceispirillum phage HMO-2011	pOTU09	*Epsilonproteobacteria*	Class level	Class level	0.656167	1	1	13	0.5583	0.0474	0.0140	0.0211

vOTU41	*Myoviridae*		Synechococcus phage S-SSM7	pOTU09	*Epsilonproteobacteria*	Class level	Class level	-0.400368	1	1	13	-0.6058	0.0282	0.0270	0.0317
vOTU44	*Myoviridae*		Yersinia phage fHe-Yen9-04	pOTU09	*Epsilonproteobacteria*	Class level	Class level	0.632072	1	1	13	0.4045	0.1704	0.0140	0.0211
vOTU11	*Herelleviridae*	*Bacillus* phage	Genus level	pOTU10	*Alphaproteobacteria*	*Ahrensia*	Genus level	0.740735	1	1	11	0.4580	0.1155	0.0030	0.0082
vOTU13	*Siphoviridae*	*Cellulophaga* phage	Genus level	pOTU10	*Alphaproteobacteria*	*Ahrensia*	Genus level	-0.571302	1	1	11	-0.4552	0.1180	0.0420	0.0413
vOTU22	*Myoviridae*	*Prochlorococcus* phage	Genus level	pOTU10	*Alphaproteobacteria*	*Ahrensia*	Genus level	-0.599166	1	1	11	-0.5576	0.0477	0.0340	0.0365
vOTU24	*Podoviridae*	*Puniceispirillum* phage	Genus level	pOTU10	*Alphaproteobacteria*	*Ahrensia*	Genus level	0.675692	1	1	11	0.6004	0.0300	0.0120	0.0192
vOTU25	*Myoviridae*	*Rhizobium* phage	Genus level	pOTU10	*Alphaproteobacteria*	*Ahrensia*	Genus level	-0.847523	9	9	3	-0.4224	0.1505	0.0300	0.0337
vOTU26	*Siphoviridae*	*Roseobacter* phage	Genus level	pOTU10	*Alphaproteobacteria*	*Ahrensia*	Genus level	0.640341	1	1	13	0.6386	0.0188	0.0370	0.0384
vOTU33	*Myoviridae*	*Yersinia* phage	Genus level	pOTU10	*Alphaproteobacteria*	*Ahrensia*	Genus level	0.643794	1	1	13	0.4786	0.0980	0.0150	0.0221
vOTU39	*Podoviridae*		Puniceispirillum phage HMO-2011	pOTU10	*Alphaproteobacteria*	*Ahrensia*	Genus level	0.675692	1	1	11	0.6004	0.0300	0.0080	0.0148
vOTU44	*Myoviridae*		Yersinia phage fHe-Yen9-04	pOTU10	*Alphaproteobacteria*	*Ahrensia*	Genus level	0.643794	1	1	13	0.4786	0.0980	0.0160	0.0231
vOTU03	*Bicaudaviridae*	Family level	Family level	pOTU11	*Flavobacteriia*	*Algibacter*	Genus level	0.26582	2	2	12	0.5634	0.0450	0.0420	0.0413
vOTU10	*Myoviridae*	*Agrobacterium* phage	Genus level	pOTU11	*Flavobacteriia*	*Algibacter*	Genus level	-0.724035	9	9	5	-0.4669	0.1077	0.0060	0.0124
vOTU15	*Myoviridae*	*Croceibacter* phage	Genus level	pOTU11	*Flavobacteriia*	*Algibacter*	Genus level	0.664429	2	2	12	0.4910	0.0884	0.0150	0.0221
vOTU16	*Siphoviridae*	*Flavobacterium* phage	Genus level	pOTU11	*Flavobacteriia*	*Algibacter*	Genus level	-0.724035	9	9	5	-0.4055	0.1693	0.0040	0.0097
vOTU28	*Myoviridae*	*Sphingomonas* phage	Genus level	pOTU11	*Flavobacteriia*	*Algibacter*	Genus level	0.463869	2	2	12	0.6240	0.0226	0.0040	0.0097
vOTU32	*Myoviridae*	*Vibrio* phage	Genus level	pOTU11	*Flavobacteriia*	*Algibacter*	Genus level	0.404745	2	2	12	0.7368	0.0041	0.0280	0.0324
vOTU35	*Podoviridae*		Cellulophaga phage phi38:1	pOTU11	*Flavobacteriia*	*Algibacter*	Genus level	-0.483219	2	2	12	-0.4667	0.1079	0.0030	0.0082
vOTU38	*Podoviridae*		Pelagibacter phage HTVC019P	pOTU11	*Flavobacteriia*	*Algibacter*	Genus level	0.406082	4	4	10	0.4911	0.0883	0.0200	0.0265
vOTU40	*Myoviridae*		Sphingomonas phage PAU	pOTU11	*Flavobacteriia*	*Algibacter*	Genus level	0.463869	2	2	12	0.6240	0.0226	0.0070	0.0137
vOTU41	*Myoviridae*		Synechococcus phage S-SSM7	pOTU11	*Flavobacteriia*	*Algibacter*	Genus level	-0.312363	4	4	10	-0.4586	0.1150	0.0070	0.0137
vOTU42	*Myoviridae*		Synechococcus phage S-WAM2	pOTU11	*Flavobacteriia*	*Algibacter*	Genus level	0.466966	2	2	12	0.6851	0.0098	0.0050	0.0110
vOTU09	*Myoviridae*	*Aeromonas* phage	Genus level	pOTU12	*Alphaproteobacteria*	*Amylibacter*	Genus level	0.656135	1	1	13	0.7217	0.0053	0.0080	0.0148
vOTU10	*Myoviridae*	*Agrobacterium* phage	Genus level	pOTU12	*Alphaproteobacteria*	*Amylibacter*	Genus level	-0.9307	9	9	5	-0.7027	0.0074	0.0220	0.0280
vOTU11	*Herelleviridae*	*Bacillus* phage	Genus level	pOTU12	*Alphaproteobacteria*	*Amylibacter*	Genus level	0.64983	1	1	12	0.4510	0.1219	0.0240	0.0296
vOTU13	*Siphoviridae*	*Cellulophaga* phage	Genus level	pOTU12	*Alphaproteobacteria*	*Amylibacter*	Genus level	-0.682304	1	1	13	-0.6947	0.0084	0.0110	0.0182

vOTU16	*Siphoviridae*	*Flavobacterium* phage	Genus level	pOTU12	*Alphaproteobacteria*	*Amylibacter*	Genus level	-1.077879	9	9	5	-0.7054	0.0071	0.0010	0.0043
vOTU22	*Myoviridae*	*Prochlorococcus* phage	Genus level	pOTU12	*Alphaproteobacteria*	*Amylibacter*	Genus level	-0.610224	1	1	13	-0.5431	0.0551	0.0440	0.0425
vOTU24	*Podoviridae*	*Puniceispirillum* phage	Genus level	pOTU12	*Alphaproteobacteria*	*Amylibacter*	Genus level	0.727747	1	1	13	0.7714	0.0020	0.0060	0.0124
vOTU25	*Myoviridae*	*Rhizobium* phage	Genus level	pOTU12	*Alphaproteobacteria*	*Amylibacter*	Genus level	-0.963812	9	9	5	-0.6047	0.0286	0.0100	0.0171
vOTU33	*Myoviridae*	*Yersinia* phage	Genus level	pOTU12	*Alphaproteobacteria*	*Amylibacter*	Genus level	0.734657	1	1	13	0.6218	0.0233	0.0040	0.0097
vOTU39	*Podoviridae*		Puniceispirillum phage HMO-2011	pOTU12	*Alphaproteobacteria*	*Amylibacter*	Genus level	0.727747	1	1	13	0.7714	0.0020	0.0030	0.0082
vOTU41	*Myoviridae*		Synechococcus phage S-SSM7	pOTU12	*Alphaproteobacteria*	*Amylibacter*	Genus level	-0.437493	1	1	13	-0.6358	0.0195	0.0170	0.0241
vOTU44	*Myoviridae*		Yersinia phage fHe-Yen9-04	pOTU12	*Alphaproteobacteria*	*Amylibacter*	Genus level	0.734657	1	1	13	0.6218	0.0233	0.0040	0.0097
vOTU06	*Microviridae*	Family level	Family level	pOTU13	*Flavobacteriia*	*Aquibacter*	Genus level	0.983367	2	2	11	0.5911	0.0334	0.0210	0.0272
vOTU10	*Myoviridae*	*Agrobacterium* phage	Genus level	pOTU13	*Flavobacteriia*	*Aquibacter*	Genus level	-0.877611	9	9	5	-0.6615	0.0138	0.0390	0.0396
vOTU11	*Herelleviridae*	*Bacillus* phage	Genus level	pOTU13	*Flavobacteriia*	*Aquibacter*	Genus level	0.586466	1	1	11	0.3056	0.3100	0.0400	0.0402
vOTU13	*Siphoviridae*	*Cellulophaga* phage	Genus level	pOTU13	*Flavobacteriia*	*Aquibacter*	Genus level	-0.584622	1	1	13	-0.6301	0.0210	0.0330	0.0358
vOTU16	*Siphoviridae*	*Flavobacterium* phage	Genus level	pOTU13	*Flavobacteriia*	*Aquibacter*	Genus level	-1.014308	9	9	5	-0.6727	0.0118	0.0040	0.0097
vOTU24	*Podoviridae*	*Puniceispirillum* phage	Genus level	pOTU13	*Flavobacteriia*	*Aquibacter*	Genus level	0.73103	1	1	13	0.7410	0.0038	0.0020	0.0066
vOTU25	*Myoviridae*	*Rhizobium* phage	Genus level	pOTU13	*Flavobacteriia*	*Aquibacter*	Genus level	-0.899021	9	9	3	-0.4535	0.1196	0.0160	0.0231
vOTU33	*Myoviridae*	*Yersinia* phage	Genus level	pOTU13	*Flavobacteriia*	*Aquibacter*	Genus level	0.616866	1	1	13	0.4687	0.1062	0.0340	0.0365
vOTU39	*Podoviridae*		Puniceispirillum phage HMO-2011	pOTU13	*Flavobacteriia*	*Aquibacter*	Genus level	0.73103	1	1	13	0.7410	0.0038	0.0020	0.0066
vOTU44	*Myoviridae*		Yersinia phage fHe-Yen9-04	pOTU13	*Flavobacteriia*	*Aquibacter*	Genus level	0.616866	1	1	13	0.4687	0.1062	0.0270	0.0317
vOTU25	*Myoviridae*	*Rhizobium* phage	Genus level	pOTU14	*Gammaproteobacteria*	*Azotobacter*	Genus level	-0.963812	9	9	3	-0.3077	0.3064	0.0120	0.0192
vOTU03	*Bicaudaviridae*	Family level	Family level	pOTU15	*Flavobacteriia*	*Cellulophaga*	Genus level	0.292858	2	2	12	0.6056	0.0283	0.0380	0.0390
vOTU10	*Myoviridae*	*Agrobacterium* phage	Genus level	pOTU15	*Flavobacteriia*	*Cellulophaga*	Genus level	-0.724035	9	9	5	-0.5064	0.0774	0.0090	0.0159
vOTU15	*Myoviridae*	*Croceibacter* phage	Genus level	pOTU15	*Flavobacteriia*	*Cellulophaga*	Genus level	0.634299	4	4	10	0.6000	0.0302	0.0290	0.0330
vOTU16	*Siphoviridae*	*Flavobacterium* phage	Genus level	pOTU15	*Flavobacteriia*	*Cellulophaga*	Genus level	-0.724035	9	9	5	-0.4398	0.1327	0.0090	0.0159
vOTU28	*Myoviridae*	*Sphingomonas* phage	Genus level	pOTU15	*Flavobacteriia*	*Cellulophaga*	Genus level	0.436832	2	2	12	0.6657	0.0130	0.0200	0.0265
vOTU35	*Podoviridae*		Cellulophaga phage phi38:1	pOTU15	*Flavobacteriia*	*Cellulophaga*	Genus level	-0.500709	2	2	12	-0.5043	0.0789	0.0060	0.0124
vOTU38	*Podoviridae*		Pelagibacter phage HTVC019P	pOTU15	*Flavobacteriia*	*Cellulophaga*	Genus level	0.406082	4	4	10	0.5124	0.0734	0.0330	0.0358

vOTU40	*Myoviridae*		Sphingomonas phage PAU	pOTU15	*Flavobacteriia*	*Cellulophaga*	Genus level	0.436832	2	2	12	0.6657	0.0130	0.0070	0.0137
vOTU41	*Myoviridae*		Synechococcus phage S-SSM7	pOTU15	*Flavobacteriia*	*Cellulophaga*	Genus level	-0.312363	4	4	10	-0.5289	0.0631	0.0110	0.0182
vOTU42	*Myoviridae*		Synechococcus phage S-WAM2	pOTU15	*Flavobacteriia*	*Cellulophaga*	Genus level	0.439928	2	2	12	0.6341	0.0199	0.0120	0.0192
vOTU07	*Myoviridae*	*Escherichia* phage	Genus level	pOTU16	*Gammaproteobacteri a*	*Cognaticolwellia*	Genus level	0.667727	2	2	12	0.6115	0.0264	0.0160	0.0231
vOTU10	*Myoviridae*	*Agrobacterium* phage	Genus level	pOTU16	*Gammaproteobacteri a*	*Cognaticolwellia*	Genus level	-1.155045	9	9	5	-0.3257	0.2774	0.0030	0.0082
vOTU11	*Herelleviridae*	*Bacillus* phage	Genus level	pOTU16	*Gammaproteobacteri a*	*Cognaticolwellia*	Genus level	0.617517	6	6	7	0.7558	0.0028	0.0400	0.0402
vOTU13	*Siphoviridae*	*Cellulophaga* phage	Genus level	pOTU16	*Gammaproteobacteri a*	*Cognaticolwellia*	Genus level	-0.722801	2	2	12	-0.3689	0.2148	0.0090	0.0159
vOTU24	*Podoviridae*	*Puniceispirillum* phage	Genus level	pOTU16	*Gammaproteobacteri a*	*Cognaticolwellia*	Genus level	0.747587	6	6	8	0.4539	0.1192	0.0050	0.0110
vOTU27	*Ackermannviridae*	*Serratia* phage	Genus level	pOTU16	*Gammaproteobacteri a*	*Cognaticolwellia*	Genus level	-0.972192	9	9	5	0.3815	0.1984	0.0290	0.0330
vOTU30	*Myoviridae*	*Synechococcus* phage	Genus level	pOTU16	*Gammaproteobacteri a*	*Cognaticolwellia*	Genus level	-0.67363	5	5	9	-0.4135	0.1602	0.0200	0.0265
vOTU33	*Myoviridae*	*Yersinia* phage	Genus level	pOTU16	*Gammaproteobacteri a*	*Cognaticolwellia*	Genus level	0.790261	4	4	10	0.7475	0.0033	0.0020	0.0066
vOTU37	*Podoviridae*		Pelagibacter phage HTVC010P	pOTU16	*Gammaproteobacteri a*	*Cognaticolwellia*	Genus level	0.741668	4	4	10	0.7039	0.0072	0.0110	0.0182
vOTU39	*Podoviridae*		Puniceispirillum phage HMO-2011	pOTU16	*Gammaproteobacteri a*	*Cognaticolwellia*	Genus level	0.747587	6	6	8	0.4539	0.1192	0.0080	0.0148
vOTU41	*Myoviridae*		Synechococcus phage S-SSM7	pOTU16	*Gammaproteobacteri a*	*Cognaticolwellia*	Genus level	-0.54612	4	4	10	-0.4159	0.1575	0.0000	0.0000
vOTU44	*Myoviridae*		Yersinia phage fHe-Yen9-04	pOTU16	*Gammaproteobacteri a*	*Cognaticolwellia*	Genus level	0.790261	4	4	10	0.7475	0.0033	0.0020	0.0066
vOTU04	*Herelleviridae*	Family level	Family level	pOTU17	*Gammaproteobacteri a*	*Colwellia*	Genus level	0.568666	2	2	12	0.5330	0.0607	0.0360	0.0378
vOTU07	*Myoviridae*	*Escherichia* phage	Genus level	pOTU17	*Gammaproteobacteri a*	*Colwellia*	Genus level	0.733835	2	2	12	0.5765	0.0392	0.0020	0.0066
vOTU10	*Myoviridae*	*Agrobacterium* phage	Genus level	pOTU17	*Gammaproteobacteri a*	*Colwellia*	Genus level	-1.009903	9	9	5	-0.4689	0.1061	0.0100	0.0171
vOTU13	*Siphoviridae*	*Cellulophaga* phage	Genus level	pOTU17	*Gammaproteobacteri a*	*Colwellia*	Genus level	-0.691699	2	2	12	-0.4003	0.1753	0.0060	0.0124
vOTU15	*Myoviridae*	*Croceibacter* phage	Genus level	pOTU17	*Gammaproteobacteri a*	*Colwellia*	Genus level	0.920323	2	2	12	0.6609	0.0139	0.0260	0.0311
vOTU16	*Siphoviridae*	*Flavobacterium* phage	Genus level	pOTU17	*Gammaproteobacteri a*	*Colwellia*	Genus level	-1.074497	9	9	5	-0.4074	0.1670	0.0050	0.0110
vOTU31	*Myoviridae*	*Thermus* phage	Genus level	pOTU17	*Gammaproteobacteri a*	*Colwellia*	Genus level	0.949491	2	2	9	0.7927	0.0012	0.0190	0.0257
vOTU35	*Podoviridae*		Cellulophaga phage phi38:1	pOTU17	*Gammaproteobacteri a*	*Colwellia*	Genus level	-0.676291	2	2	12	-0.5054	0.0781	0.0050	0.0110
vOTU41	*Myoviridae*		Synechococcus phage S-SSM7	pOTU17	*Gammaproteobacteri a*	*Colwellia*	Genus level	-0.445046	3	3	11	-0.3402	0.2555	0.0100	0.0171
vOTU11	*Herelleviridae*	*Bacillus* phage	Genus level	pOTU18	*Gammaproteobacteri a*	*Eionea*	Genus level	0.750628	1	1	11	0.3856	0.1932	0.0010	0.0043
vOTU13	*Siphoviridae*	*Cellulophaga* phage	Genus level	pOTU18	*Gammaproteobacteri a*	*Eionea*	Genus level	-0.5892	1	1	13	-0.4499	0.1229	0.0300	0.0337

vOTU16	*Siphoviridae*	*Flavobacterium* phage	Genus level	pOTU18	*Gammaproteobacteria*	*Eionea*	Genus level	-0.982157	9	9	5	-0.4635	0.1107	0.0060	0.0124
vOTU22	*Myoviridae*	*Prochlorococcus* phage	Genus level	pOTU18	*Gammaproteobacteria*	*Eionea*	Genus level	-0.662836	1	1	13	-0.6810	0.0104	0.0210	0.0272
vOTU24	*Podoviridae*	*Puniceispirillum* phage	Genus level	pOTU18	*Gammaproteobacteria*	*Eionea*	Genus level	0.716302	1	1	13	0.5941	0.0323	0.0040	0.0097
vOTU25	*Myoviridae*	*Rhizobium* phage	Genus level	pOTU18	*Gammaproteobacteria*	*Eionea*	Genus level	-0.963812	9	9	3	-0.3485	0.2432	0.0070	0.0137
vOTU26	*Siphoviridae*	*Roseobacter* phage	Genus level	pOTU18	*Gammaproteobacteria*	*Eionea*	Genus level	0.692269	1	1	13	0.6952	0.0083	0.0120	0.0192
vOTU30	*Myoviridae*	*Synechococcus* phage	Genus level	pOTU18	*Gammaproteobacteria*	*Eionea*	Genus level	-0.670975	2	2	12	-0.3668	0.2176	0.0100	0.0171
vOTU33	*Myoviridae*	*Yersinia* phage	Genus level	pOTU18	*Gammaproteobacteria*	*Eionea*	Genus level	0.725297	1	1	13	0.4942	0.0860	0.0050	0.0110
vOTU39	*Podoviridae*		Puniceispirillum phage HMO-2011	pOTU18	*Gammaproteobacteria*	*Eionea*	Genus level	0.716302	1	1	13	0.5941	0.0323	0.0090	0.0159
vOTU41	*Myoviridae*		Synechococcus phage S-SSM7	pOTU18	*Gammaproteobacteria*	*Eionea*	Genus level	-0.498861	1	1	13	-0.6965	0.0082	0.0010	0.0043
vOTU44	*Myoviridae*		Yersinia phage fHe-Yen9-04	pOTU18	*Gammaproteobacteria*	*Eionea*	Genus level	0.725297	1	1	13	0.4942	0.0860	0.0020	0.0066
vOTU05	*Inoviridae*	Family level	Family level	pOTU19	*Cyanobacteriota*	*Foliisarcina*	Genus level	0.568926	11	11	3	0.4692	0.1058	0.0240	0.0296
vOTU10	*Myoviridae*	*Agrobacterium* phage	Genus level	pOTU19	*Cyanobacteriota*	*Foliisarcina*	Genus level	0.669889	9	9	5	0.4105	0.1636	0.0200	0.0265
vOTU13	*Siphoviridae*	*Cellulophaga* phage	Genus level	pOTU19	*Cyanobacteriota*	*Foliisarcina*	Genus level	0.460311	2	2	12	0.3753	0.2063	0.0100	0.0171
vOTU15	*Myoviridae*	*Croceibacter* phage	Genus level	pOTU19	*Cyanobacteriota*	*Foliisarcina*	Genus level	-0.800254	2	2	12	-0.3145	0.2953	0.0000	0.0000
vOTU27	*Ackermannviridae*	*Serratia* phage	Genus level	pOTU19	*Cyanobacteriota*	*Foliisarcina*	Genus level	0.690872	6	6	8	0.6143	0.0255	0.0180	0.0250
vOTU38	*Podoviridae*		Pelagibacter phage HTVC019P	pOTU19	*Cyanobacteriota*	*Foliisarcina*	Genus level	-0.472984	1	1	13	-0.3423	0.2522	0.0080	0.0148
vOTU42	*Myoviridae*		Synechococcus phage S-WAM2	pOTU19	*Cyanobacteriota*	*Foliisarcina*	Genus level	-0.506857	1	1	13	-0.3385	0.2579	0.0030	0.0082
vOTU04	*Herelleviridae*	Family level	Family level	pOTU20	*Gammaproteobacteria*	*Halomonas*	Genus level	0.475564	1	1	13	0.5606	0.0463	0.0050	0.0110
vOTU07	*Myoviridae*	*Escherichia* phage	Genus level	pOTU20	*Gammaproteobacteria*	*Halomonas*	Genus level	0.484995	2	2	12	0.6668	0.0128	0.0030	0.0082
vOTU10	*Myoviridae*	*Agrobacterium* phage	Genus level	pOTU20	*Gammaproteobacteria*	*Halomonas*	Genus level	-0.724035	9	9	5	-0.3479	0.2441	0.0060	0.0124
vOTU13	*Siphoviridae*	*Cellulophaga* phage	Genus level	pOTU20	*Gammaproteobacteria*	*Halomonas*	Genus level	-0.510745	1	1	13	-0.3495	0.2418	0.0000	0.0000
vOTU16	*Siphoviridae*	*Flavobacterium* phage	Genus level	pOTU20	*Gammaproteobacteria*	*Halomonas*	Genus level	-0.724035	9	9	5	-0.3021	0.3158	0.0040	0.0097
vOTU17	*Siphoviridae*	*Lactobacillus* phage	Genus level	pOTU20	*Gammaproteobacteria*	*Halomonas*	Genus level	0.603226	2	2	10	0.9641	0.0000	0.0120	0.0192
vOTU23	*Podoviridae*	*Pseudomonas* phage	Genus level	pOTU20	*Gammaproteobacteria*	*Halomonas*	Genus level	0.277317	1	1	13	0.6631	0.0135	0.0360	0.0378
vOTU28	*Myoviridae*	*Sphingomonas* phage	Genus level	pOTU20	*Gammaproteobacteria*	*Halomonas*	Genus level	0.392194	1	1	13	0.5504	0.0513	0.0240	0.0296
vOTU31	*Myoviridae*	*Thermus* phage	Genus level	pOTU20	*Gammaproteobacteria*	*Halomonas*	Genus level	0.786197	1	1	10	0.6726	0.0118	0.0020	0.0066

vOTU34	*Myoviridae*		Aeromonas virus 65	pOTU20	*Gammaproteobacteria*	*Halomonas*	Genus level	0.280246	1	1	13	0.3522	0.2379	0.0380	0.0390
vOTU35	*Podoviridae*		Cellulophaga phage phi38:1	pOTU20	*Gammaproteobacteria*	*Halomonas*	Genus level	-0.41418	2	2	12	-0.3858	0.1929	0.0270	0.0317
vOTU40	*Myoviridae*		Sphingomonas phage PAU	pOTU20	*Gammaproteobacteria*	*Halomonas*	Genus level	0.392194	1	1	13	0.5504	0.0513	0.0260	0.0311
vOTU07	*Myoviridae*	*Escherichia* phage	Genus level	pOTU21	*Acidimicrobiia*	*Ilumatobacter*	Genus level	-0.653827	2	2	12	-0.4311	0.1413	0.0100	0.0171
vOTU08	*Myoviridae*	*Acinetobacter* phage	Genus level	pOTU21	*Acidimicrobiia*	*Ilumatobacter*	Genus level	-0.458532	2	2	12	-0.4154	0.1581	0.0240	0.0296
vOTU09	*Myoviridae*	*Aeromonas* phage	Genus level	pOTU21	*Acidimicrobiia*	*Ilumatobacter*	Genus level	-0.607851	1	1	13	-0.4433	0.1292	0.0150	0.0221
vOTU10	*Myoviridae*	*Agrobacterium* phage	Genus level	pOTU21	*Acidimicrobiia*	*Ilumatobacter*	Genus level	1.145309	9	9	5	0.7064	0.0070	0.0010	0.0043
vOTU13	*Siphoviridae*	*Cellulophaga* phage	Genus level	pOTU21	*Acidimicrobiia*	*Ilumatobacter*	Genus level	0.722466	1	1	13	0.5641	0.0446	0.0050	0.0110
vOTU16	*Siphoviridae*	*Flavobacterium* phage	Genus level	pOTU21	*Acidimicrobiia*	*Ilumatobacter*	Genus level	1.154113	9	9	5	0.6873	0.0094	0.0020	0.0066
vOTU22	*Myoviridae*	*Prochlorococcus* phage	Genus level	pOTU21	*Acidimicrobiia*	*Ilumatobacter*	Genus level	0.730859	1	1	13	0.4177	0.1555	0.0100	0.0171
vOTU23	*Podoviridae*	*Pseudomonas* phage	Genus level	pOTU21	*Acidimicrobiia*	*Ilumatobacter*	Genus level	-0.430706	1	1	13	-0.3957	0.1808	0.0210	0.0272
vOTU24	*Podoviridae*	*Puniceispirillum* phage	Genus level	pOTU21	*Acidimicrobiia*	*Ilumatobacter*	Genus level	-0.599858	1	1	13	-0.6348	0.0198	0.0330	0.0358
vOTU28	*Myoviridae*	*Sphingomonas* phage	Genus level	pOTU21	*Acidimicrobiia*	*Ilumatobacter*	Genus level	-0.775053	1	1	13	-0.5636	0.0449	0.0000	0.0000
vOTU32	*Myoviridae*	*Vibrio* phage	Genus level	pOTU21	*Acidimicrobiia*	*Ilumatobacter*	Genus level	-0.669141	2	2	12	-0.4283	0.1442	0.0120	0.0192
vOTU33	*Myoviridae*	*Yersinia* phage	Genus level	pOTU21	*Acidimicrobiia*	*Ilumatobacter*	Genus level	-0.589114	1	1	13	-0.4279	0.1447	0.0270	0.0317
vOTU38	*Podoviridae*		Pelagibacter phage HTVC019P	pOTU21	*Acidimicrobiia*	*Ilumatobacter*	Genus level	-0.58277	3	3	11	-0.4410	0.1315	0.0290	0.0330
vOTU39	*Podoviridae*		Puniceispirillum phage HMO-2011	pOTU21	*Acidimicrobiia*	*Ilumatobacter*	Genus level	-0.599858	1	1	13	-0.6348	0.0198	0.0390	0.0396
vOTU40	*Myoviridae*		Sphingomonas phage PAU	pOTU21	*Acidimicrobiia*	*Ilumatobacter*	Genus level	-0.775053	1	1	13	-0.5636	0.0449	0.0000	0.0000
vOTU41	*Myoviridae*		Synechococcus phage S-SSM7	pOTU21	*Acidimicrobiia*	*Ilumatobacter*	Genus level	0.432541	1	1	13	0.4282	0.1444	0.0220	0.0280
vOTU42	*Myoviridae*		Synechococcus phage S-WAM2	pOTU21	*Acidimicrobiia*	*Ilumatobacter*	Genus level	-0.709561	2	2	12	-0.4775	0.0989	0.0040	0.0097
vOTU43	*Podoviridae*		Vibrio phage CHOED	pOTU21	*Acidimicrobiia*	*Ilumatobacter*	Genus level	-0.586959	2	2	12	-0.3680	0.2160	0.0400	0.0402
vOTU44	*Myoviridae*		Yersinia phage fHe-Yen9-04	pOTU21	*Acidimicrobiia*	*Ilumatobacter*	Genus level	-0.589114	1	1	13	-0.4279	0.1447	0.0390	0.0396
vOTU03	*Bicaudaviridae*	Family level	Family level	pOTU22	*Flavobacteriia*	*Lacinutrix*	Genus level	0.446508	1	1	10	0.7236	0.0052	0.0240	0.0296
vOTU07	*Myoviridae*	*Escherichia* phage	Genus level	pOTU22	*Flavobacteriia*	*Lacinutrix*	Genus level	0.626519	1	1	12	0.4729	0.1026	0.0220	0.0280
vOTU13	*Siphoviridae*	*Cellulophaga* phage	Genus level	pOTU22	*Flavobacteriia*	*Lacinutrix*	Genus level	-0.583553	2	2	11	-0.4570	0.1164	0.0410	0.0408
vOTU21	*Siphoviridae*	*Polaribacter* phage	Genus level	pOTU22	*Flavobacteriia*	*Lacinutrix*	Genus level	0.556544	1	1	13	0.8198	0.0006	0.0010	0.0043

vOTU25	*Myoviridae*	*Rhizobium* phage	Genus level	pOTU22	*Flavobacteriia*	*Lacinutrix*	Genus level	-0.8731	9	9	5	-0.3127	0.2982	0.0270	0.0317
vOTU30	*Myoviridae*	*Synechococcus* phage	Genus level	pOTU22	*Flavobacteriia*	*Lacinutrix*	Genus level	-0.621999	1	1	11	-0.4022	0.1731	0.0330	0.0358
vOTU33	*Myoviridae*	*Yersinia* phage	Genus level	pOTU22	*Flavobacteriia*	*Lacinutrix*	Genus level	0.606099	4	4	9	0.4202	0.1529	0.0380	0.0390
vOTU35	*Podoviridae*		Cellulophaga phage phi38:1	pOTU22	*Flavobacteriia*	*Lacinutrix*	Genus level	-0.807635	1	1	12	-0.6253	0.0223	0.0000	0.0000
vOTU41	*Myoviridae*		Synechococcus phage S-SSM7	pOTU22	*Flavobacteriia*	*Lacinutrix*	Genus level	-0.415928	4	4	9	-0.3225	0.2825	0.0290	0.0330
vOTU42	*Myoviridae*		Synechococcus phage S-WAM2	pOTU22	*Flavobacteriia*	*Lacinutrix*	Genus level	0.72506	1	1	12	0.7260	0.0050	0.0020	0.0066
vOTU43	*Podoviridae*		Vibrio phage CHOED	pOTU22	*Flavobacteriia*	*Lacinutrix*	Genus level	0.630677	1	1	13	0.5651	0.0442	0.0050	0.0110
vOTU44	*Myoviridae*		Yersinia phage fHe-Yen9-04	pOTU22	*Flavobacteriia*	*Lacinutrix*	Genus level	0.606099	4	4	9	0.4202	0.1529	0.0370	0.0384
vOTU15	*Myoviridae*	*Croceibacter* phage	Genus level	pOTU23	*Flavobacteriia*	*Leeuwenhoekiella*	Genus level	2.462935	3	3	11	0.5007	0.0813	0.0270	0.0317
vOTU20	*Podoviridae*	*Nonlabens* phage	Genus level	pOTU23	*Flavobacteriia*	*Leeuwenhoekiella*	Genus level	0.824022	4	4	10	0.8615	0.0002	0.0000	0.0000
vOTU07	*Myoviridae*	*Escherichia* phage	Genus level	pOTU24	*Alphaproteobacteria*	*Loktanella*	Genus level	-0.536095	2	2	12	-0.5705	0.0418	0.0480	0.0450
vOTU10	*Myoviridae*	*Agrobacterium* phage	Genus level	pOTU24	*Alphaproteobacteria*	*Loktanella*	Genus level	1.056444	9	9	5	0.7973	0.0011	0.0080	0.0148
vOTU11	*Herelleviridae*	*Bacillus* phage	Genus level	pOTU24	*Alphaproteobacteria*	*Loktanella*	Genus level	-0.567641	1	1	12	-0.3909	0.1866	0.0460	0.0438
vOTU13	*Siphoviridae*	*Cellulophaga* phage	Genus level	pOTU24	*Alphaproteobacteria*	*Loktanella*	Genus level	0.645221	1	1	13	0.8407	0.0003	0.0080	0.0148
vOTU16	*Siphoviridae*	*Flavobacterium* phage	Genus level	pOTU24	*Alphaproteobacteria*	*Loktanella*	Genus level	1.216218	9	9	5	0.9511	0.0000	0.0000	0.0000
vOTU22	*Myoviridae*	*Prochlorococcus* phage	Genus level	pOTU24	*Alphaproteobacteria*	*Loktanella*	Genus level	0.746614	1	1	13	0.3964	0.1800	0.0080	0.0148
vOTU23	*Podoviridae*	*Pseudomonas* phage	Genus level	pOTU24	*Alphaproteobacteria*	*Loktanella*	Genus level	-0.400885	1	1	13	-0.4844	0.0935	0.0380	0.0390
vOTU24	*Podoviridae*	*Puniceispirillum* phage	Genus level	pOTU24	*Alphaproteobacteria*	*Loktanella*	Genus level	-0.729768	1	1	13	-0.8162	0.0007	0.0040	0.0097
vOTU25	*Myoviridae*	*Rhizobium* phage	Genus level	pOTU24	*Alphaproteobacteria*	*Loktanella*	Genus level	0.955065	9	9	5	0.6635	0.0134	0.0080	0.0148
vOTU28	*Myoviridae*	*Sphingomonas* phage	Genus level	pOTU24	*Alphaproteobacteria*	*Loktanella*	Genus level	-0.609123	1	1	13	-0.6027	0.0292	0.0240	0.0296
vOTU29	*Myoviridae*	*Staphylococcus* phage	Genus level	pOTU24	*Alphaproteobacteria*	*Loktanella*	Genus level	0.84944	9	9	5	0.6840	0.0099	0.0190	0.0257
vOTU30	*Myoviridae*	*Synechococcus* phage	Genus level	pOTU24	*Alphaproteobacteria*	*Loktanella*	Genus level	0.658334	2	2	12	0.6813	0.0104	0.0180	0.0250
vOTU32	*Myoviridae*	*Vibrio* phage	Genus level	pOTU24	*Alphaproteobacteria*	*Loktanella*	Genus level	-0.558227	2	2	12	-0.4465	0.1261	0.0460	0.0438
vOTU33	*Myoviridae*	*Yersinia* phage	Genus level	pOTU24	*Alphaproteobacteria*	*Loktanella*	Genus level	-0.70514	1	1	13	-0.7066	0.0069	0.0090	0.0159
vOTU39	*Podoviridae*		Puniceispirillum phage HMO-2011	pOTU24	*Alphaproteobacteria*	*Loktanella*	Genus level	-0.729768	1	1	13	-0.8162	0.0007	0.0030	0.0082
vOTU40	*Myoviridae*		Sphingomonas phage PAU	pOTU24	*Alphaproteobacteria*	*Loktanella*	Genus level	-0.609123	1	1	13	-0.6027	0.0292	0.0220	0.0280

vOTU41	*Myoviridae*		Synechococcus phage S-SSM7	pOTU24	*Alphaproteobacteria*	*Loktanella*	Genus level	0.524484	1	1	13	0.6740	0.0115	0.0000	0.0000
vOTU42	*Myoviridae*		Synechococcus phage S-WAM2	pOTU24	*Alphaproteobacteria*	*Loktanella*	Genus level	-0.657611	2	2	12	-0.6457	0.0171	0.0080	0.0148
vOTU43	*Podoviridae*		Vibrio phage CHOED	pOTU24	*Alphaproteobacteria*	*Loktanella*	Genus level	-0.591365	2	2	12	-0.5090	0.0756	0.0350	0.0372
vOTU44	*Myoviridae*		Yersinia phage fHe-Yen9-04	pOTU24	*Alphaproteobacteria*	*Loktanella*	Genus level	-0.70514	1	1	13	-0.7066	0.0069	0.0090	0.0159
vOTU10	*Myoviridae*	*Agrobacterium* phage	Genus level	pOTU25	*Alphaproteobacteria*	*Magnetospira*	Genus level	-0.863256	9	9	4	-0.4564	0.1169	0.0410	0.0408
vOTU11	*Herelleviridae*	*Bacillus* phage	Genus level	pOTU25	*Alphaproteobacteria*	*Magnetospira*	Genus level	0.78188	1	1	13	0.4330	0.1394	0.0020	0.0066
vOTU16	*Siphoviridae*	*Flavobacterium* phage	Genus level	pOTU25	*Alphaproteobacteria*	*Magnetospira*	Genus level	-0.906878	9	9	4	-0.3771	0.2040	0.0180	0.0250
vOTU22	*Myoviridae*	*Prochlorococcus* phage	Genus level	pOTU25	*Alphaproteobacteria*	*Magnetospira*	Genus level	-0.70276	1	1	12	-0.6319	0.0205	0.0080	0.0148
vOTU24	*Podoviridae*	*Puniceispirillum* phage	Genus level	pOTU25	*Alphaproteobacteria*	*Magnetospira*	Genus level	0.707051	1	1	12	0.5988	0.0306	0.0040	0.0097
vOTU25	*Myoviridae*	*Rhizobium* phage	Genus level	pOTU25	*Alphaproteobacteria*	*Magnetospira*	Genus level	-0.977076	9	9	5	-0.3775	0.2035	0.0140	0.0211
vOTU33	*Myoviridae*	*Yersinia* phage	Genus level	pOTU25	*Alphaproteobacteria*	*Magnetospira*	Genus level	0.676874	1	1	12	0.4662	0.1083	0.0070	0.0137
vOTU37	*Podoviridae*		Pelagibacter phage HTVC010P	pOTU25	*Alphaproteobacteria*	*Magnetospira*	Genus level	0.612926	1	1	12	0.4833	0.0943	0.0340	0.0365
vOTU39	*Podoviridae*		Puniceispirillum phage HMO-2011	pOTU25	*Alphaproteobacteria*	*Magnetospira*	Genus level	0.707051	1	1	12	0.5988	0.0306	0.0080	0.0148
vOTU41	*Myoviridae*		Synechococcus phage S-SSM7	pOTU25	*Alphaproteobacteria*	*Magnetospira*	Genus level	-0.481933	1	1	12	-0.5815	0.0371	0.0050	0.0110
vOTU44	*Myoviridae*		Yersinia phage fHe-Yen9-04	pOTU25	*Alphaproteobacteria*	*Magnetospira*	Genus level	0.676874	1	1	12	0.4662	0.1083	0.0120	0.0192
vOTU03	*Bicaudaviridae*	Family level	Family level	pOTU26	*Flavobacteriia*	*Maribacter*	Genus level	0.278659	1	1	13	0.7494	0.0032	0.0330	0.0358
vOTU07	*Myoviridae*	*Escherichia* phage	Genus level	pOTU26	*Flavobacteriia*	*Maribacter*	Genus level	0.498743	1	1	13	0.7259	0.0050	0.0020	0.0066
vOTU10	*Myoviridae*	*Agrobacterium* phage	Genus level	pOTU26	*Flavobacteriia*	*Maribacter*	Genus level	-0.724035	9	9	5	-0.6244	0.0225	0.0050	0.0110
vOTU13	*Siphoviridae*	*Cellulophaga* phage	Genus level	pOTU26	*Flavobacteriia*	*Maribacter*	Genus level	-0.521451	2	2	12	-0.5856	0.0355	0.0000	0.0000
vOTU16	*Siphoviridae*	*Flavobacterium* phage	Genus level	pOTU26	*Flavobacteriia*	*Maribacter*	Genus level	-0.724035	9	9	5	-0.5422	0.0556	0.0030	0.0082
vOTU23	*Podoviridae*	*Pseudomonas* phage	Genus level	pOTU26	*Flavobacteriia*	*Maribacter*	Genus level	0.267479	2	2	12	0.7530	0.0030	0.0380	0.0390
vOTU25	*Myoviridae*	*Rhizobium* phage	Genus level	pOTU26	*Flavobacteriia*	*Maribacter*	Genus level	-0.606832	9	9	5	-0.3583	0.2293	0.0230	0.0288
vOTU28	*Myoviridae*	*Sphingomonas* phage	Genus level	pOTU26	*Flavobacteriia*	*Maribacter*	Genus level	0.420572	2	2	12	0.6405	0.0184	0.0220	0.0280
vOTU32	*Myoviridae*	*Vibrio* phage	Genus level	pOTU26	*Flavobacteriia*	*Maribacter*	Genus level	0.419251	1	1	13	0.6442	0.0175	0.0220	0.0280
vOTU35	*Podoviridae*		Cellulophaga phage phi38:1	pOTU26	*Flavobacteriia*	*Maribacter*	Genus level	-0.505958	1	1	13	-0.6479	0.0166	0.0020	0.0066
vOTU40	*Myoviridae*		Sphingomonas phage PAU	pOTU26	*Flavobacteriia*	*Maribacter*	Genus level	0.420572	2	2	12	0.6405	0.0184	0.0240	0.0296

vOTU41	*Myoviridae*		Synechococcus phage S-SSM7	pOTU26	*Flavobacteriia*	*Maribacter*	Genus level	-0.273282	4	4	10	-0.3506	0.2402	0.0230	0.0288
vOTU42	*Myoviridae*		Synechococcus phage S-WAM2	pOTU26	*Flavobacteriia*	*Maribacter*	Genus level	0.507598	1	1	13	0.8136	0.0007	0.0010	0.0043
vOTU43	*Podoviridae*		Vibrio phage CHOED	pOTU26	*Flavobacteriia*	*Maribacter*	Genus level	0.401394	1	1	13	0.7062	0.0070	0.0260	0.0311
vOTU09	*Myoviridae*	*Aeromonas* phage	Genus level	pOTU27	*Flavobacteriia*	*Maribrevibacterium*	Genus level	0.422045	1	1	13	0.6326	0.0203	0.0300	0.0337
vOTU10	*Myoviridae*	*Agrobacterium* phage	Genus level	pOTU27	*Flavobacteriia*	*Maribrevibacterium*	Genus level	-0.789343	9	9	5	-0.4656	0.1089	0.0040	0.0097
vOTU11	*Herelleviridae*	*Bacillus* phage	Genus level	pOTU27	*Flavobacteriia*	*Maribrevibacterium*	Genus level	0.537906	1	1	12	0.3128	0.2981	0.0050	0.0110
vOTU13	*Siphoviridae*	*Cellulophaga* phage	Genus level	pOTU27	*Flavobacteriia*	*Maribrevibacterium*	Genus level	-0.457534	1	1	13	-0.4152	0.1583	0.0220	0.0280
vOTU16	*Siphoviridae*	*Flavobacterium* phage	Genus level	pOTU27	*Flavobacteriia*	*Maribrevibacterium*	Genus level	-0.820441	9	9	5	-0.4142	0.1594	0.0020	0.0066
vOTU22	*Myoviridae*	*Prochlorococcus* phage	Genus level	pOTU27	*Flavobacteriia*	*Maribrevibacterium*	Genus level	-0.548544	1	1	13	-0.6777	0.0109	0.0040	0.0097
vOTU24	*Podoviridae*	*Puniceispirillum* phage	Genus level	pOTU27	*Flavobacteriia*	*Maribrevibacterium*	Genus level	0.550091	1	1	13	0.5588	0.0471	0.0010	0.0043
vOTU26	*Siphoviridae*	*Roseobacter* phage	Genus level	pOTU27	*Flavobacteriia*	*Maribrevibacterium*	Genus level	0.461551	1	1	13	0.6052	0.0284	0.0390	0.0396
vOTU33	*Myoviridae*	*Yersinia* phage	Genus level	pOTU27	*Flavobacteriia*	*Maribrevibacterium*	Genus level	0.550902	1	1	13	0.4553	0.1180	0.0020	0.0066
vOTU34	*Myoviridae*		Aeromonas virus 65	pOTU27	*Flavobacteriia*	*Maribrevibacterium*	Genus level	0.310294	1	1	13	0.6859	0.0097	0.0400	0.0402
vOTU39	*Podoviridae*		Puniceispirillum phage HMO-2011	pOTU27	*Flavobacteriia*	*Maribrevibacterium*	Genus level	0.550091	1	1	13	0.5588	0.0471	0.0000	0.0000
vOTU41	*Myoviridae*		Synechococcus phage S-SSM7	pOTU27	*Flavobacteriia*	*Maribrevibacterium*	Genus level	-0.373995	1	1	13	-0.6485	0.0165	0.0020	0.0066
vOTU44	*Myoviridae*		Yersinia phage fHe-Yen9-04	pOTU27	*Flavobacteriia*	*Maribrevibacterium*	Genus level	0.550902	1	1	13	0.4553	0.1180	0.0010	0.0043
vOTU09	*Myoviridae*	*Aeromonas* phage	Genus level	pOTU28	*Alphaproteobacteria*	*Marinibaculum*	Genus level	0.594769	1	1	13	0.6669	0.0128	0.0280	0.0324
vOTU10	*Myoviridae*	*Agrobacterium* phage	Genus level	pOTU28	*Alphaproteobacteria*	*Marinibaculum*	Genus level	-1.163008	9	9	5	-0.6239	0.0227	0.0010	0.0043
vOTU11	*Herelleviridae*	*Bacillus* phage	Genus level	pOTU28	*Alphaproteobacteria*	*Marinibaculum*	Genus level	0.699771	1	1	12	0.3809	0.1991	0.0080	0.0148
vOTU13	*Siphoviridae*	*Cellulophaga* phage	Genus level	pOTU28	*Alphaproteobacteria*	*Marinibaculum*	Genus level	-0.671588	1	1	13	-0.5657	0.0439	0.0190	0.0257
vOTU16	*Siphoviridae*	*Flavobacterium* phage	Genus level	pOTU28	*Alphaproteobacteria*	*Marinibaculum*	Genus level	-1.128373	9	9	5	-0.5490	0.0520	0.0020	0.0066
vOTU22	*Myoviridae*	*Prochlorococcus* phage	Genus level	pOTU28	*Alphaproteobacteria*	*Marinibaculum*	Genus level	-0.800169	1	1	13	-0.7182	0.0057	0.0030	0.0082
vOTU24	*Podoviridae*	*Puniceispirillum* phage	Genus level	pOTU28	*Alphaproteobacteria*	*Marinibaculum*	Genus level	0.746027	1	1	13	0.6787	0.0108	0.0020	0.0066
vOTU25	*Myoviridae*	*Rhizobium* phage	Genus level	pOTU28	*Alphaproteobacteria*	*Marinibaculum*	Genus level	-0.869145	9	9	5	-0.3625	0.2235	0.0400	0.0402
vOTU30	*Myoviridae*	*Synechococcus* phage	Genus level	pOTU28	*Alphaproteobacteria*	*Marinibaculum*	Genus level	-0.660407	2	2	12	-0.3283	0.2734	0.0260	0.0311
vOTU33	*Myoviridae*	*Yersinia* phage	Genus level	pOTU28	*Alphaproteobacteria*	*Marinibaculum*	Genus level	0.800988	1	1	13	0.5711	0.0415	0.0010	0.0043

vOTU39	*Podoviridae*		Puniceispirillum phage HMO-2011	pOTU28	*Alphaproteobacteria*	*Marinibaculum*	Genus level	0.746027	1	1	13	0.6787	0.0108	0.0020	0.0066
vOTU41	*Myoviridae*		Synechococcus phage S-SSM7	pOTU28	*Alphaproteobacteria*	*Marinibaculum*	Genus level	-0.550652	1	1	13	-0.7022	0.0075	0.0010	0.0043
vOTU42	*Myoviridae*		Synechococcus phage S-WAM2	pOTU28	*Alphaproteobacteria*	*Marinibaculum*	Genus level	0.629121	2	2	12	0.3516	0.2388	0.0190	0.0257
vOTU44	*Myoviridae*		Yersinia phage fHe-Yen9-04	pOTU28	*Alphaproteobacteria*	*Marinibaculum*	Genus level	0.800988	1	1	13	0.5711	0.0415	0.0010	0.0043
vOTU09	*Myoviridae*	*Aeromonas* phage	Genus level	pOTU29	*Gammaproteobacteria*	*Marinobacterium*	Genus level	0.430813	1	1	13	0.7301	0.0046	0.0070	0.0137
vOTU10	*Myoviridae*	*Agrobacterium* phage	Genus level	pOTU29	*Gammaproteobacteria*	*Marinobacterium*	Genus level	-0.724035	9	9	5	-0.5016	0.0807	0.0020	0.0066
vOTU11	*Herelleviridae*	*Bacillus* phage	Genus level	pOTU29	*Gammaproteobacteria*	*Marinobacterium*	Genus level	0.479101	1	1	12	0.3565	0.2318	0.0030	0.0082
vOTU13	*Siphoviridae*	*Cellulophaga* phage	Genus level	pOTU29	*Gammaproteobacteria*	*Marinobacterium*	Genus level	-0.469646	1	1	13	-0.4770	0.0993	0.0100	0.0171
vOTU16	*Siphoviridae*	*Flavobacterium* phage	Genus level	pOTU29	*Gammaproteobacteria*	*Marinobacterium*	Genus level	-0.724035	9	9	5	-0.4356	0.1368	0.0070	0.0137
vOTU22	*Myoviridae*	*Prochlorococcus* phage	Genus level	pOTU29	*Gammaproteobacteria*	*Marinobacterium*	Genus level	-0.604826	1	1	13	-0.7221	0.0053	0.0000	0.0000
vOTU24	*Podoviridae*	*Puniceispirillum* phage	Genus level	pOTU29	*Gammaproteobacteria*	*Marinobacterium*	Genus level	0.504084	1	1	13	0.5682	0.0428	0.0020	0.0066
vOTU33	*Myoviridae*	*Yersinia* phage	Genus level	pOTU29	*Gammaproteobacteria*	*Marinobacterium*	Genus level	0.461051	1	1	13	0.3561	0.2324	0.0060	0.0124
vOTU34	*Myoviridae*		Aeromonas virus 65	pOTU29	*Gammaproteobacteria*	*Marinobacterium*	Genus level	0.328314	1	1	13	0.8036	0.0009	0.0050	0.0110
vOTU39	*Podoviridae*		Puniceispirillum phage HMO-2011	pOTU29	*Gammaproteobacteria*	*Marinobacterium*	Genus level	0.504084	1	1	13	0.5682	0.0428	0.0030	0.0082
vOTU40	*Myoviridae*		Sphingomonas phage PAU	pOTU29	*Gammaproteobacteria*	*Marinobacterium*	Genus level	0.359342	1	1	13	0.4426	0.1299	0.0470	0.0444
vOTU41	*Myoviridae*		Synechococcus phage S-SSM7	pOTU29	*Gammaproteobacteria*	*Marinobacterium*	Genus level	-0.337712	1	1	13	-0.5864	0.0352	0.0030	0.0082
vOTU44	*Myoviridae*		Yersinia phage fHe-Yen9-04	pOTU29	*Gammaproteobacteria*	*Marinobacterium*	Genus level	0.461051	1	1	13	0.3561	0.2324	0.0040	0.0097
vOTU10	*Myoviridae*	*Agrobacterium* phage	Genus level	pOTU30	*Cytophagia*	*Marinoscillum*	Genus level	-0.974821	9	9	5	-0.5654	0.0440	0.0190	0.0257
vOTU13	*Siphoviridae*	*Cellulophaga* phage	Genus level	pOTU30	*Cytophagia*	*Marinoscillum*	Genus level	-0.625451	1	1	13	-0.5032	0.0796	0.0190	0.0257
vOTU16	*Siphoviridae*	*Flavobacterium* phage	Genus level	pOTU30	*Cytophagia*	*Marinoscillum*	Genus level	-1.114664	9	9	5	-0.5030	0.0798	0.0010	0.0043
vOTU22	*Myoviridae*	*Prochlorococcus* phage	Genus level	pOTU30	*Cytophagia*	*Marinoscillum*	Genus level	-0.650805	1	1	13	-0.6588	0.0143	0.0220	0.0280
vOTU24	*Podoviridae*	*Puniceispirillum* phage	Genus level	pOTU30	*Cytophagia*	*Marinoscillum*	Genus level	0.792425	1	1	13	0.6193	0.0240	0.0000	0.0000
vOTU25	*Myoviridae*	*Rhizobium* phage	Genus level	pOTU30	*Cytophagia*	*Marinoscillum*	Genus level	-1.005543	9	9	5	-0.3539	0.2355	0.0050	0.0110
vOTU29	*Myoviridae*	*Staphylococcus* phage	Genus level	pOTU30	*Cytophagia*	*Marinoscillum*	Genus level	-0.806299	9	9	5	-0.3297	0.2713	0.0440	0.0425
vOTU33	*Myoviridae*	*Yersinia* phage	Genus level	pOTU30	*Cytophagia*	*Marinoscillum*	Genus level	0.792676	1	1	13	0.4672	0.1075	0.0020	0.0066
vOTU37	*Podoviridae*		Pelagibacter phage HTVC010P	pOTU30	*Cytophagia*	*Marinoscillum*	Genus level	0.711214	1	1	13	0.4804	0.0966	0.0090	0.0159

vOTU39	*Podoviridae*		Puniceispirillum phage HMO-2011	pOTU30	*Cytophagia*	*Marinoscillum*	Genus level	0.792425	1	1	13	0.6193	0.0240	0.0010	0.0043
vOTU41	*Myoviridae*		Synechococcus phage S-SSM7	pOTU30	*Cytophagia*	*Marinoscillum*	Genus level	-0.500467	1	1	13	-0.6301	0.0210	0.0000	0.0000
vOTU44	*Myoviridae*		Yersinia phage fHe-Yen9-04	pOTU30	*Cytophagia*	*Marinoscillum*	Genus level	0.792676	1	1	13	0.4672	0.1075	0.0000	0.0000
vOTU01	*Ackermannviridae*	Family level	Family level	pOTU32	*Gammaproteobacteria*	*Methylophaga*	Genus level	0.415011	1	1	12	0.4639	0.1103	0.0420	0.0413
vOTU22	*Myoviridae*	*Prochlorococcus* phage	Genus level	pOTU32	*Gammaproteobacteria*	*Methylophaga*	Genus level	0.617883	1	1	13	0.5951	0.0319	0.0260	0.0311
vOTU07	*Myoviridae*	*Escherichia* phage	Genus level	pOTU33	*Gammaproteobacteria*	*Moraxella*	Genus level	0.582503	2	2	12	0.3359	0.2618	0.0290	0.0330
vOTU09	*Myoviridae*	*Aeromonas* phage	Genus level	pOTU33	*Gammaproteobacteria*	*Moraxella*	Genus level	0.659636	1	1	13	0.6617	0.0138	0.0100	0.0171
vOTU10	*Myoviridae*	*Agrobacterium* phage	Genus level	pOTU33	*Gammaproteobacteria*	*Moraxella*	Genus level	-0.974821	9	9	5	-0.7103	0.0065	0.0150	0.0221
vOTU11	*Herelleviridae*	*Bacillus* phage	Genus level	pOTU33	*Gammaproteobacteria*	*Moraxella*	Genus level	0.744617	1	1	12	0.4781	0.0984	0.0010	0.0043
vOTU13	*Siphoviridae*	*Cellulophaga* phage	Genus level	pOTU33	*Gammaproteobacteria*	*Moraxella*	Genus level	-0.742362	1	1	13	-0.6993	0.0078	0.0010	0.0043
vOTU16	*Siphoviridae*	*Flavobacterium* phage	Genus level	pOTU33	*Gammaproteobacteria*	*Moraxella*	Genus level	-1.114664	9	9	5	-0.6569	0.0147	0.0010	0.0043
vOTU22	*Myoviridae*	*Prochlorococcus* phage	Genus level	pOTU33	*Gammaproteobacteria*	*Moraxella*	Genus level	-0.705008	1	1	13	-0.6717	0.0119	0.0100	0.0171
vOTU24	*Podoviridae*	*Puniceispirillum* phage	Genus level	pOTU33	*Gammaproteobacteria*	*Moraxella*	Genus level	0.767256	1	1	13	0.7603	0.0026	0.0020	0.0066
vOTU25	*Myoviridae*	*Rhizobium* phage	Genus level	pOTU33	*Gammaproteobacteria*	*Moraxella*	Genus level	-1.005543	9	9	5	-0.4734	0.1022	0.0040	0.0097
vOTU30	*Myoviridae*	*Synechococcus* phage	Genus level	pOTU33	*Gammaproteobacteria*	*Moraxella*	Genus level	-0.570298	2	2	12	-0.4306	0.1419	0.0390	0.0396
vOTU33	*Myoviridae*	*Yersinia* phage	Genus level	pOTU33	*Gammaproteobacteria*	*Moraxella*	Genus level	0.768454	1	1	13	0.6052	0.0284	0.0020	0.0066
vOTU34	*Myoviridae*		Aeromonas virus 65	pOTU33	*Gammaproteobacteria*	*Moraxella*	Genus level	0.427491	1	1	13	0.7003	0.0077	0.0450	0.0432
vOTU37	*Podoviridae*		Pelagibacter phage HTVC010P	pOTU33	*Gammaproteobacteria*	*Moraxella*	Genus level	0.62098	3	3	11	0.5032	0.0796	0.0360	0.0378
vOTU39	*Podoviridae*		Puniceispirillum phage HMO-2011	pOTU33	*Gammaproteobacteria*	*Moraxella*	Genus level	0.767256	1	1	13	0.7603	0.0026	0.0010	0.0043
vOTU41	*Myoviridae*		Synechococcus phage S-SSM7	pOTU33	*Gammaproteobacteria*	*Moraxella*	Genus level	-0.483812	1	1	13	-0.6468	0.0169	0.0050	0.0110
vOTU42	*Myoviridae*		Synechococcus phage S-WAM2	pOTU33	*Gammaproteobacteria*	*Moraxella*	Genus level	0.55405	2	2	12	0.5115	0.0740	0.0440	0.0425
vOTU44	*Myoviridae*		Yersinia phage fHe-Yen9-04	pOTU33	*Gammaproteobacteria*	*Moraxella*	Genus level	0.768454	1	1	13	0.6052	0.0284	0.0000	0.0000
vOTU10	*Myoviridae*	*Agrobacterium* phage	Genus level	pOTU34	*Alphaproteobacteria*	*Novosphingobium*	Genus level	-1.155045	9	9	5	-0.6481	0.0166	0.0040	0.0097
vOTU11	*Herelleviridae*	*Bacillus* phage	Genus level	pOTU34	*Alphaproteobacteria*	*Novosphingobium*	Genus level	0.675187	1	1	12	0.3119	0.2996	0.0160	0.0231
vOTU13	*Siphoviridae*	*Cellulophaga* phage	Genus level	pOTU34	*Alphaproteobacteria*	*Novosphingobium*	Genus level	-0.685243	1	1	13	-0.5861	0.0353	0.0110	0.0182
vOTU16	*Siphoviridae*	*Flavobacterium* phage	Genus level	pOTU34	*Alphaproteobacteria*	*Novosphingobium*	Genus level	-1.202269	9	9	5	-0.5659	0.0438	0.0010	0.0043

vOTU22	*Myoviridae*	*Prochlorococcus* phage	Genus level	pOTU34	*Alphaproteobacteria*	*Novosphingobium*	Genus level	-0.795209	1	1	13	-0.6944	0.0085	0.0040	0.0097
vOTU24	*Podoviridae*	*Puniceispirillum* phage	Genus level	pOTU34	*Alphaproteobacteria*	*Novosphingobium*	Genus level	0.819151	1	1	13	0.6795	0.0106	0.0010	0.0043
vOTU25	*Myoviridae*	*Rhizobium* phage	Genus level	pOTU34	*Alphaproteobacteria*	*Novosphingobium*	Genus level	-1.099389	9	9	5	-0.3677	0.2164	0.0030	0.0082
vOTU27	*Ackermannviridae*	*Serratia* phage	Genus level	pOTU34	*Alphaproteobacteria*	*Novosphingobium*	Genus level	-0.972192	9	9	5	-0.3485	0.2432	0.0350	0.0372
vOTU29	*Myoviridae*	*Staphylococcus* phage	Genus level	pOTU34	*Alphaproteobacteria*	*Novosphingobium*	Genus level	-0.825929	9	9	5	-0.3505	0.2404	0.0440	0.0425
vOTU30	*Myoviridae*	*Synechococcus* phage	Genus level	pOTU34	*Alphaproteobacteria*	*Novosphingobium*	Genus level	-0.624629	3	3	11	-0.3232	0.2814	0.0270	0.0317
vOTU33	*Myoviridae*	*Yersinia* phage	Genus level	pOTU34	*Alphaproteobacteria*	*Novosphingobium*	Genus level	0.828368	1	1	13	0.5588	0.0471	0.0020	0.0066
vOTU37	*Podoviridae*		Pelagibacter phage HTVC010P	pOTU34	*Alphaproteobacteria*	*Novosphingobium*	Genus level	0.755238	1	1	13	0.5636	0.0448	0.0120	0.0192
vOTU39	*Podoviridae*		Puniceispirillum phage HMO-2011	pOTU34	*Alphaproteobacteria*	*Novosphingobium*	Genus level	0.819151	1	1	13	0.6795	0.0106	0.0010	0.0043
vOTU41	*Myoviridae*		Synechococcus phage S-SSM7	pOTU34	*Alphaproteobacteria*	*Novosphingobium*	Genus level	-0.57534	1	1	13	-0.6787	0.0108	0.0000	0.0000
vOTU44	*Myoviridae*		Yersinia phage fHe-Yen9-04	pOTU34	*Alphaproteobacteria*	*Novosphingobium*	Genus level	0.828368	1	1	13	0.5588	0.0471	0.0000	0.0000
vOTU09	*Myoviridae*	*Aeromonas* phage	Genus level	pOTU35	*Flavobacteriia*	*Owenweeksia*	Genus level	0.62336	1	1	13	0.6870	0.0095	0.0090	0.0159
vOTU10	*Myoviridae*	*Agrobacterium* phage	Genus level	pOTU35	*Flavobacteriia*	*Owenweeksia*	Genus level	-1.065216	9	9	5	-0.7989	0.0011	0.0050	0.0110
vOTU13	*Siphoviridae*	*Cellulophaga* phage	Genus level	pOTU35	*Flavobacteriia*	*Owenweeksia*	Genus level	-0.740121	1	1	13	-0.7236	0.0052	0.0040	0.0097
vOTU16	*Siphoviridae*	*Flavobacterium* phage	Genus level	pOTU35	*Flavobacteriia*	*Owenweeksia*	Genus level	-1.155793	9	9	5	-0.7034	0.0073	0.0000	0.0000
vOTU24	*Podoviridae*	*Puniceispirillum* phage	Genus level	pOTU35	*Flavobacteriia*	*Owenweeksia*	Genus level	0.771503	1	1	13	0.8042	0.0009	0.0010	0.0043
vOTU25	*Myoviridae*	*Rhizobium* phage	Genus level	pOTU35	*Flavobacteriia*	*Owenweeksia*	Genus level	-0.972852	9	9	5	-0.4645	0.1098	0.0060	0.0124
vOTU33	*Myoviridae*	*Yersinia* phage	Genus level	pOTU35	*Flavobacteriia*	*Owenweeksia*	Genus level	0.701881	1	1	13	0.4998	0.0820	0.0070	0.0137
vOTU39	*Podoviridae*		Puniceispirillum phage HMO-2011	pOTU35	*Flavobacteriia*	*Owenweeksia*	Genus level	0.771503	1	1	13	0.8042	0.0009	0.0040	0.0097
vOTU41	*Myoviridae*		Synechococcus phage S-SSM7	pOTU35	*Flavobacteriia*	*Owenweeksia*	Genus level	-0.390665	1	1	13	-0.4508	0.1221	0.0400	0.0402
vOTU42	*Myoviridae*		Synechococcus phage S-WAM2	pOTU35	*Flavobacteriia*	*Owenweeksia*	Genus level	0.55904	2	2	12	0.5211	0.0678	0.0380	0.0390
vOTU44	*Myoviridae*		Yersinia phage fHe-Yen9-04	pOTU35	*Flavobacteriia*	*Owenweeksia*	Genus level	0.701881	1	1	13	0.4998	0.0820	0.0050	0.0110
vOTU03	*Bicaudaviridae*	Family level	Family level	pOTU36	*Gammaproteobacteria*	*Paraglaciecola*	Genus level	0.403383	2	2	12	0.6423	0.0179	0.0360	0.0378
vOTU07	*Myoviridae*	*Escherichia* phage	Genus level	pOTU36	*Gammaproteobacteria*	*Paraglaciecola*	Genus level	0.672745	2	2	12	0.6459	0.0171	0.0040	0.0097
vOTU10	*Myoviridae*	*Agrobacterium* phage	Genus level	pOTU36	*Gammaproteobacteria*	*Paraglaciecola*	Genus level	-0.960611	9	9	5	-0.5415	0.0560	0.0150	0.0221
vOTU13	*Siphoviridae*	*Cellulophaga* phage	Genus level	pOTU36	*Gammaproteobacteria*	*Paraglaciecola*	Genus level	-0.764967	2	2	12	-0.6234	0.0228	0.0010	0.0043

vOTU16	*Siphoviridae*	*Flavobacterium* phage	Genus level	pOTU36	*Gammaproteobacteria*	*Paraglaciecola*	Genus level	-1.074497	9	9	5	-0.4702	0.1049	0.0010	0.0043
vOTU21	*Siphoviridae*	*Polaribacter* phage	Genus level	pOTU36	*Gammaproteobacteria*	*Paraglaciecola*	Genus level	0.418894	2	2	11	0.7148	0.0060	0.0490	0.0456
vOTU24	*Podoviridae*	*Puniceispirillum* phage	Genus level	pOTU36	*Gammaproteobacteria*	*Paraglaciecola*	Genus level	0.619338	6	6	8	0.3930	0.1840	0.0120	0.0192
vOTU25	*Myoviridae*	*Rhizobium* phage	Genus level	pOTU36	*Gammaproteobacteria*	*Paraglaciecola*	Genus level	-1.05107	9	9	5	-0.3115	0.3001	0.0000	0.0000
vOTU33	*Myoviridae*	*Yersinia* phage	Genus level	pOTU36	*Gammaproteobacteria*	*Paraglaciecola*	Genus level	0.747692	4	4	10	0.6360	0.0195	0.0000	0.0000
vOTU35	*Podoviridae*		Cellulophaga phage phi38:1	pOTU36	*Gammaproteobacteria*	*Paraglaciecola*	Genus level	-0.708751	2	2	12	-0.5276	0.0639	0.0040	0.0097
vOTU37	*Podoviridae*		Pelagibacter phage HTVC010P	pOTU36	*Gammaproteobacteria*	*Paraglaciecola*	Genus level	0.726683	4	4	10	0.4927	0.0871	0.0040	0.0097
vOTU39	*Podoviridae*		Puniceispirillum phage HMO-2011	pOTU36	*Gammaproteobacteria*	*Paraglaciecola*	Genus level	0.619338	6	6	8	0.3930	0.1840	0.0120	0.0192
vOTU42	*Myoviridae*		Synechococcus phage S-WAM2	pOTU36	*Gammaproteobacteria*	*Paraglaciecola*	Genus level	0.614365	2	2	12	0.6447	0.0174	0.0130	0.0202
vOTU44	*Myoviridae*		Yersinia phage fHe-Yen9-04	pOTU36	*Gammaproteobacteria*	*Paraglaciecola*	Genus level	0.747692	4	4	10	0.6360	0.0195	0.0020	0.0066
vOTU11	*Herelleviridae*	*Bacillus* phage	Genus level	pOTU37	*Gammaproteobacteria*	*Parahaliea*	Genus level	0.563033	1	1	13	0.3834	0.1960	0.0000	0.0000
vOTU13	*Siphoviridae*	*Cellulophaga* phage	Genus level	pOTU37	*Gammaproteobacteria*	*Parahaliea*	Genus level	-0.470242	1	1	13	-0.5017	0.0807	0.0210	0.0272
vOTU16	*Siphoviridae*	*Flavobacterium* phage	Genus level	pOTU37	*Gammaproteobacteria*	*Parahaliea*	Genus level	-0.704748	9	9	5	-0.4411	0.1314	0.0090	0.0159
vOTU22	*Myoviridae*	*Prochlorococcus* phage	Genus level	pOTU37	*Gammaproteobacteria*	*Parahaliea*	Genus level	-0.508208	1	1	13	-0.6814	0.0103	0.0140	0.0211
vOTU24	*Podoviridae*	*Puniceispirillum* phage	Genus level	pOTU37	*Gammaproteobacteria*	*Parahaliea*	Genus level	0.517144	1	1	13	0.5978	0.0309	0.0040	0.0097
vOTU25	*Myoviridae*	*Rhizobium* phage	Genus level	pOTU37	*Gammaproteobacteria*	*Parahaliea*	Genus level	-0.749713	9	9	5	-0.3458	0.2472	0.0010	0.0043
vOTU29	*Myoviridae*	*Staphylococcus* phage	Genus level	pOTU37	*Gammaproteobacteria*	*Parahaliea*	Genus level	-0.607272	9	9	5	-0.3264	0.2764	0.0190	0.0257
vOTU30	*Myoviridae*	*Synechococcus* phage	Genus level	pOTU37	*Gammaproteobacteria*	*Parahaliea*	Genus level	-0.418406	2	2	10	-0.3262	0.2767	0.0480	0.0450
vOTU33	*Myoviridae*	*Yersinia* phage	Genus level	pOTU37	*Gammaproteobacteria*	*Parahaliea*	Genus level	0.485995	1	1	13	0.3955	0.1810	0.0210	0.0272
vOTU39	*Podoviridae*		Puniceispirillum phage HMO-2011	pOTU37	*Gammaproteobacteria*	*Parahaliea*	Genus level	0.517144	1	1	13	0.5978	0.0309	0.0020	0.0066
vOTU41	*Myoviridae*		Synechococcus phage S-SSM7	pOTU37	*Gammaproteobacteria*	*Parahaliea*	Genus level	-0.32572	1	1	13	-0.5646	0.0444	0.0080	0.0148
vOTU44	*Myoviridae*		Yersinia phage fHe-Yen9-04	pOTU37	*Gammaproteobacteria*	*Parahaliea*	Genus level	0.485995	1	1	13	0.3955	0.1810	0.0120	0.0192
vOTU07	*Myoviridae*	*Escherichia* phage	Genus level	pOTU38	*Alphaproteobacteria*	*Parasphingorhabdus*	Genus level	0.482652	1	1	13	0.3302	0.2705	0.0030	0.0082
vOTU10	*Myoviridae*	*Agrobacterium* phage	Genus level	pOTU38	*Alphaproteobacteria*	*Parasphingorhabdus*	Genus level	-0.724035	9	9	5	-0.3889	0.1891	0.0100	0.0171
vOTU15	*Myoviridae*	*Croceibacter* phage	Genus level	pOTU38	*Alphaproteobacteria*	*Parasphingorhabdus*	Genus level	0.818972	2	2	12	0.7028	0.0074	0.0000	0.0000
vOTU16	*Siphoviridae*	*Flavobacterium* phage	Genus level	pOTU38	*Alphaproteobacteria*	*Parasphingorhabdus*	Genus level	-0.724035	9	9	5	-0.3377	0.2592	0.0080	0.0148

vOTU28	*Myoviridae*	*Sphingomonas* phage	Genus level	pOTU38	*Alphaproteobacteria*	*Parasphingorhabdus*	Genus level	0.390382	2	2	12	0.4014	0.1740	0.0430	0.0419
vOTU31	*Myoviridae*	*Thermus* phage	Genus level	pOTU38	*Alphaproteobacteria*	*Parasphingorhabdus*	Genus level	0.598942	2	2	9	0.6054	0.0283	0.0440	0.0425
vOTU35	*Podoviridae*		Cellulophaga phage phi38:1	pOTU38	*Alphaproteobacteria*	*Parasphingorhabdus*	Genus level	-0.527488	1	1	13	-0.4218	0.1511	0.0020	0.0066
vOTU38	*Podoviridae*		Pelagibacter phage HTVC019P	pOTU38	*Alphaproteobacteria*	*Parasphingorhabdus*	Genus level	0.452761	1	1	13	0.3552	0.2336	0.0140	0.0211
vOTU40	*Myoviridae*		Sphingomonas phage PAU	pOTU38	*Alphaproteobacteria*	*Parasphingorhabdus*	Genus level	0.390382	2	2	12	0.4014	0.1740	0.0480	0.0450
vOTU14	*Myoviridae*	*Clostridium* phage	Genus level	pOTU39	*Alphaproteobacteria*	*Paraurantiacibacter*	Genus level	0.634659	3	3	11	0.7281	0.0048	0.0280	0.0324
vOTU15	*Myoviridae*	*Croceibacter* phage	Genus level	pOTU39	*Alphaproteobacteria*	*Paraurantiacibacter*	Genus level	1.079709	3	3	11	0.4474	0.1253	0.0060	0.0124
vOTU22	*Myoviridae*	*Prochlorococcus* phage	Genus level	pOTU39	*Alphaproteobacteria*	*Paraurantiacibacter*	Genus level	-0.590598	3	3	11	-0.3777	0.2032	0.0470	0.0444
vOTU32	*Myoviridae*	*Vibrio* phage	Genus level	pOTU39	*Alphaproteobacteria*	*Paraurantiacibacter*	Genus level	0.551707	1	1	13	0.3657	0.2192	0.0390	0.0396
vOTU38	*Podoviridae*		Pelagibacter phage HTVC019P	pOTU39	*Alphaproteobacteria*	*Paraurantiacibacter*	Genus level	0.771568	1	1	13	0.6412	0.0182	0.0000	0.0000
vOTU41	*Myoviridae*		Synechococcus phage S-SSM7	pOTU39	*Alphaproteobacteria*	*Paraurantiacibacter*	Genus level	-0.427472	3	3	11	-0.3615	0.2248	0.0170	0.0241
vOTU25	*Myoviridae*	*Rhizobium* phage	Genus level	pOTU40	*Alphaproteobacteria*	*Planktomarina*	Genus level	-0.899021	9	9	3	-0.4561	0.1172	0.0180	0.0250
vOTU33	*Myoviridae*	*Yersinia* phage	Genus level	pOTU40	*Alphaproteobacteria*	*Planktomarina*	Genus level	0.583783	1	1	11	0.4218	0.1511	0.0480	0.0450
vOTU03	*Bicaudaviridae*	Family level	Family level	pOTU41	*Flavobacteriia*	*Polaribacter*	Genus level	0.397353	1	1	13	0.5811	0.0373	0.0460	0.0438
vOTU07	*Myoviridae*	*Escherichia* phage	Genus level	pOTU41	*Flavobacteriia*	*Polaribacter*	Genus level	0.579931	1	1	13	0.3199	0.2866	0.0320	0.0351
vOTU10	*Myoviridae*	*Agrobacterium* phage	Genus level	pOTU41	*Flavobacteriia*	*Polaribacter*	Genus level	-1.076883	9	9	5	-0.6653	0.0131	0.0040	0.0097
vOTU13	*Siphoviridae*	*Cellulophaga* phage	Genus level	pOTU41	*Flavobacteriia*	*Polaribacter*	Genus level	-0.615095	2	2	12	-0.4817	0.0956	0.0290	0.0330
vOTU15	*Myoviridae*	*Croceibacter* phage	Genus level	pOTU41	*Flavobacteriia*	*Polaribacter*	Genus level	0.902492	2	2	12	0.5413	0.0561	0.0410	0.0408
vOTU16	*Siphoviridae*	*Flavobacterium* phage	Genus level	pOTU41	*Flavobacteriia*	*Polaribacter*	Genus level	-1.198879	9	9	5	-0.6239	0.0227	0.0000	0.0000
vOTU24	*Podoviridae*	*Puniceispirillum* phage	Genus level	pOTU41	*Flavobacteriia*	*Polaribacter*	Genus level	0.615785	6	6	8	0.4656	0.1088	0.0440	0.0425
vOTU25	*Myoviridae*	*Rhizobium* phage	Genus level	pOTU41	*Flavobacteriia*	*Polaribacter*	Genus level	-0.932396	9	9	5	-0.3810	0.1990	0.0150	0.0221
vOTU28	*Myoviridae*	*Sphingomonas* phage	Genus level	pOTU41	*Flavobacteriia*	*Polaribacter*	Genus level	0.575412	2	2	12	0.5945	0.0321	0.0280	0.0324
vOTU30	*Myoviridae*	*Synechococcus* phage	Genus level	pOTU41	*Flavobacteriia*	*Polaribacter*	Genus level	-0.660213	5	5	9	-0.5686	0.0426	0.0140	0.0211
vOTU33	*Myoviridae*	*Yersinia* phage	Genus level	pOTU41	*Flavobacteriia*	*Polaribacter*	Genus level	0.690654	4	4	10	0.4333	0.1391	0.0070	0.0137
vOTU35	*Podoviridae*		Cellulophaga phage phi38:1	pOTU41	*Flavobacteriia*	*Polaribacter*	Genus level	-0.763628	1	1	13	-0.5711	0.0415	0.0010	0.0043
vOTU38	*Podoviridae*		Pelagibacter phage HTVC019P	pOTU41	*Flavobacteriia*	*Polaribacter*	Genus level	0.58422	1	1	13	0.6238	0.0227	0.0300	0.0337

vOTU39	*Podoviridae*		Puniceispirillum phage HMO-2011	pOTU41	*Flavobacteriia*	*Polaribacter*	Genus level	0.615785	6	6	8	0.4656	0.1088	0.0210	0.0272
vOTU40	*Myoviridae*		Sphingomonas phage PAU	pOTU41	*Flavobacteriia*	*Polaribacter*	Genus level	0.575412	2	2	12	0.5945	0.0321	0.0370	0.0384
vOTU41	*Myoviridae*		Synechococcus phage S-SSM7	pOTU41	*Flavobacteriia*	*Polaribacter*	Genus level	-0.463736	4	4	10	-0.5652	0.0441	0.0090	0.0159
vOTU42	*Myoviridae*		Synechococcus phage S-WAM2	pOTU41	*Flavobacteriia*	*Polaribacter*	Genus level	0.764829	1	1	13	0.8085	0.0008	0.0000	0.0000
vOTU44	*Myoviridae*		Yersinia phage fHe-Yen9-04	pOTU41	*Flavobacteriia*	*Polaribacter*	Genus level	0.690654	4	4	10	0.4333	0.1391	0.0050	0.0110
vOTU16	*Siphoviridae*	*Flavobacterium* phage	Genus level	pOTU42	*Gammaproteobacteria*	*Porticoccus*	Genus level	-0.932172	9	9	3	-0.3948	0.1819	0.0150	0.0221
vOTU24	*Podoviridae*	*Puniceispirillum* phage	Genus level	pOTU42	*Gammaproteobacteria*	*Porticoccus*	Genus level	0.614017	1	1	11	0.4094	0.1648	0.0200	0.0265
vOTU25	*Myoviridae*	*Rhizobium* phage	Genus level	pOTU42	*Gammaproteobacteria*	*Porticoccus*	Genus level	-0.991527	9	9	3	-0.5236	0.0663	0.0080	0.0148
vOTU29	*Myoviridae*	*Staphylococcus* phage	Genus level	pOTU42	*Gammaproteobacteria*	*Porticoccus*	Genus level	-0.843781	9	9	3	-0.4659	0.1086	0.0330	0.0358
vOTU33	*Myoviridae*	*Yersinia* phage	Genus level	pOTU42	*Gammaproteobacteria*	*Porticoccus*	Genus level	0.613063	1	1	11	0.4438	0.1288	0.0250	0.0304
vOTU37	*Podoviridae*		Pelagibacter phage HTVC010P	pOTU42	*Gammaproteobacteria*	*Porticoccus*	Genus level	0.613465	1	1	11	0.3908	0.1868	0.0430	0.0419
vOTU39	*Podoviridae*		Puniceispirillum phage HMO-2011	pOTU42	*Gammaproteobacteria*	*Porticoccus*	Genus level	0.614017	1	1	11	0.4094	0.1648	0.0230	0.0288
vOTU41	*Myoviridae*		Synechococcus phage S-SSM7	pOTU42	*Gammaproteobacteria*	*Porticoccus*	Genus level	-0.384577	1	1	11	-0.4508	0.1221	0.0430	0.0419
vOTU44	*Myoviridae*		Yersinia phage fHe-Yen9-04	pOTU42	*Gammaproteobacteria*	*Porticoccus*	Genus level	0.613063	1	1	11	0.4438	0.1288	0.0210	0.0272
vOTU09	*Myoviridae*	*Aeromonas* phage	Genus level	pOTU43	*Gammaproteobacteria*	*Pseudoalteromonas*	Genus level	0.48208	1	1	13	0.8527	0.0002	0.0020	0.0066
vOTU12	*Myoviridae*	*Campylobacter* phage	Genus level	pOTU43	*Gammaproteobacteria*	*Pseudoalteromonas*	Genus level	0.261362	1	1	13	0.6492	0.0164	0.0360	0.0378
vOTU22	*Myoviridae*	*Prochlorococcus* phage	Genus level	pOTU43	*Gammaproteobacteria*	*Pseudoalteromonas*	Genus level	-0.480777	1	1	13	-0.4391	0.1334	0.0140	0.0211
vOTU23	*Podoviridae*	*Pseudomonas* phage	Genus level	pOTU43	*Gammaproteobacteria*	*Pseudoalteromonas*	Genus level	0.278132	1	1	13	0.4939	0.0863	0.0210	0.0272
vOTU24	*Podoviridae*	*Puniceispirillum* phage	Genus level	pOTU43	*Gammaproteobacteria*	*Pseudoalteromonas*	Genus level	0.457111	1	1	13	0.3498	0.2413	0.0080	0.0148
vOTU31	*Myoviridae*	*Thermus* phage	Genus level	pOTU43	*Gammaproteobacteria*	*Pseudoalteromonas*	Genus level	0.625853	1	1	10	0.5068	0.0771	0.0260	0.0311
vOTU39	*Podoviridae*		Puniceispirillum phage HMO-2011	pOTU43	*Gammaproteobacteria*	*Pseudoalteromonas*	Genus level	0.457111	1	1	13	0.3498	0.2413	0.0130	0.0202
vOTU41	*Myoviridae*		Synechococcus phage S-SSM7	pOTU43	*Gammaproteobacteria*	*Pseudoalteromonas*	Genus level	-0.319571	1	1	13	-0.3901	0.1876	0.0040	0.0097
vOTU09	*Myoviridae*	*Aeromonas* phage	Genus level	pOTU44	*Gammaproteobacteria*	*Pseudohongiella*	Genus level	0.583623	1	1	13	0.4979	0.0833	0.0390	0.0396
vOTU10	*Myoviridae*	*Agrobacterium* phage	Genus level	pOTU44	*Gammaproteobacteria*	*Pseudohongiella*	Genus level	-1.047976	9	9	5	-0.5403	0.0566	0.0130	0.0202
vOTU11	*Herelleviridae*	*Bacillus* phage	Genus level	pOTU44	*Gammaproteobacteria*	*Pseudohongiella*	Genus level	0.768457	1	1	12	0.3565	0.2319	0.0020	0.0066
vOTU13	*Siphoviridae*	*Cellulophaga* phage	Genus level	pOTU44	*Gammaproteobacteria*	*Pseudohongiella*	Genus level	-0.656917	1	1	13	-0.5361	0.0590	0.0260	0.0311

vOTU16	*Siphoviridae*	*Flavobacterium* phage	Genus level	pOTU44	*Gammaproteobacteria*	*Pseudohongiella*	Genus level	-1.144794	9	9	5	-0.5042	0.0789	0.0030	0.0082
vOTU22	*Myoviridae*	*Prochlorococcus* phage	Genus level	pOTU44	*Gammaproteobacteria*	*Pseudohongiella*	Genus level	-0.710792	1	1	13	-0.6141	0.0255	0.0240	0.0296
vOTU24	*Podoviridae*	*Puniceispirillum* phage	Genus level	pOTU44	*Gammaproteobacteria*	*Pseudohongiella*	Genus level	0.815266	1	1	13	0.6215	0.0234	0.0000	0.0000
vOTU25	*Myoviridae*	*Rhizobium* phage	Genus level	pOTU44	*Gammaproteobacteria*	*Pseudohongiella*	Genus level	-1.038029	9	9	5	-0.3646	0.2207	0.0130	0.0202
vOTU30	*Myoviridae*	*Synechococcus* phage	Genus level	pOTU44	*Gammaproteobacteria*	*Pseudohongiella*	Genus level	-0.626127	2	2	12	-0.3425	0.2520	0.0320	0.0351
vOTU33	*Myoviridae*	*Yersinia* phage	Genus level	pOTU44	*Gammaproteobacteria*	*Pseudohongiella*	Genus level	0.764411	1	1	13	0.4837	0.0940	0.0050	0.0110
vOTU39	*Podoviridae*		Puniceispirillum phage HMO-2011	pOTU44	*Gammaproteobacteria*	*Pseudohongiella*	Genus level	0.815266	1	1	13	0.6215	0.0234	0.0000	0.0000
vOTU41	*Myoviridae*		Synechococcus phage S-SSM7	pOTU44	*Gammaproteobacteria*	*Pseudohongiella*	Genus level	-0.521452	1	1	13	-0.5684	0.0427	0.0030	0.0082
vOTU44	*Myoviridae*		Yersinia phage fHe-Yen9-04	pOTU44	*Gammaproteobacteria*	*Pseudohongiella*	Genus level	0.764411	1	1	13	0.4837	0.0940	0.0030	0.0082
vOTU10	*Myoviridae*	*Agrobacterium* phage	Genus level	pOTU45	*Gammaproteobacteria*	*Pseudomonas*	Genus level	-0.792508	9	9	5	-0.5804	0.0376	0.0060	0.0124
vOTU15	*Myoviridae*	*Croceibacter* phage	Genus level	pOTU45	*Gammaproteobacteria*	*Pseudomonas*	Genus level	0.728408	2	2	12	0.6526	0.0156	0.0180	0.0250
vOTU16	*Siphoviridae*	*Flavobacterium* phage	Genus level	pOTU45	*Gammaproteobacteria*	*Pseudomonas*	Genus level	-0.828511	9	9	5	-0.5040	0.0791	0.0010	0.0043
vOTU24	*Podoviridae*	*Puniceispirillum* phage	Genus level	pOTU45	*Gammaproteobacteria*	*Pseudomonas*	Genus level	0.429759	7	7	7	0.4361	0.1363	0.0380	0.0390
vOTU25	*Myoviridae*	*Rhizobium* phage	Genus level	pOTU45	*Gammaproteobacteria*	*Pseudomonas*	Genus level	-0.818348	9	9	5	-0.3332	0.2660	0.0000	0.0000
vOTU27	*Ackermannviridae*	*Serratia* phage	Genus level	pOTU45	*Gammaproteobacteria*	*Pseudomonas*	Genus level	-0.668562	6	6	8	-0.4083	0.1660	0.0360	0.0378
vOTU28	*Myoviridae*	*Sphingomonas* phage	Genus level	pOTU45	*Gammaproteobacteria*	*Pseudomonas*	Genus level	0.429991	1	1	13	0.5582	0.0474	0.0250	0.0304
vOTU29	*Myoviridae*	*Staphylococcus* phage	Genus level	pOTU45	*Gammaproteobacteria*	*Pseudomonas*	Genus level	-0.675907	9	9	5	-0.3107	0.3015	0.0180	0.0250
vOTU35	*Podoviridae*		Cellulophaga phage phi38:1	pOTU45	*Gammaproteobacteria*	*Pseudomonas*	Genus level	-0.45868	2	2	12	-0.4120	0.1619	0.0180	0.0250
vOTU39	*Podoviridae*		Puniceispirillum phage HMO-2011	pOTU45	*Gammaproteobacteria*	*Pseudomonas*	Genus level	0.429759	7	7	7	0.4361	0.1363	0.0400	0.0402
vOTU40	*Myoviridae*		Sphingomonas phage PAU	pOTU45	*Gammaproteobacteria*	*Pseudomonas*	Genus level	0.429991	1	1	13	0.5582	0.0474	0.0300	0.0337
vOTU10	*Myoviridae*	*Agrobacterium* phage	Genus level	pOTU46	*Gammaproteobacteria*	*Psychrobacter*	Genus level	-0.724035	9	9	5	-0.4363	0.1361	0.0050	0.0110
vOTU15	*Myoviridae*	*Croceibacter* phage	Genus level	pOTU46	*Gammaproteobacteria*	*Psychrobacter*	Genus level	0.608865	2	2	12	0.4529	0.1202	0.0330	0.0358
vOTU16	*Siphoviridae*	*Flavobacterium* phage	Genus level	pOTU46	*Gammaproteobacteria*	*Psychrobacter*	Genus level	-0.724035	9	9	5	-0.3789	0.2017	0.0040	0.0097
vOTU24	*Podoviridae*	*Puniceispirillum* phage	Genus level	pOTU46	*Gammaproteobacteria*	*Psychrobacter*	Genus level	0.390677	1	1	13	0.3924	0.1847	0.0400	0.0402
vOTU28	*Myoviridae*	*Sphingomonas* phage	Genus level	pOTU46	*Gammaproteobacteria*	*Psychrobacter*	Genus level	0.469336	1	1	13	0.5924	0.0329	0.0080	0.0148
vOTU32	*Myoviridae*	*Vibrio* phage	Genus level	pOTU46	*Gammaproteobacteria*	*Psychrobacter*	Genus level	0.377647	2	2	12	0.5338	0.0603	0.0430	0.0419

vOTU35	*Podoviridae*		Cellulophaga phage phi38:1	pOTU46	*Gammaproteobacteria*	*Psychrobacter*	Genus level	-0.430379	2	2	12	-0.3936	0.1833	0.0160	0.0231
vOTU36	*Siphoviridae*		Nonlabens phage P12024S	pOTU46	*Gammaproteobacteria*	*Psychrobacter*	Genus level	0.423518	2	2	11	0.8479	0.0003	0.0290	0.0330
vOTU39	*Podoviridae*		Puniceispirillum phage HMO-2011	pOTU46	*Gammaproteobacteria*	*Psychrobacter*	Genus level	0.390677	1	1	13	0.3924	0.1847	0.0420	0.0413
vOTU40	*Myoviridae*		Sphingomonas phage PAU	pOTU46	*Gammaproteobacteria*	*Psychrobacter*	Genus level	0.469336	1	1	13	0.5924	0.0329	0.0030	0.0082
vOTU42	*Myoviridae*		Synechococcus phage S-WAM2	pOTU46	*Gammaproteobacteria*	*Psychrobacter*	Genus level	0.476545	2	2	12	0.6267	0.0219	0.0040	0.0097
vOTU07	*Myoviridae*	*Escherichia* phage	Genus level	pOTU47	*Gammaproteobacteria*	*Psychromonas*	Genus level	0.420869	1	1	13	0.5572	0.0479	0.0160	0.0231
vOTU10	*Myoviridae*	*Agrobacterium* phage	Genus level	pOTU47	*Gammaproteobacteria*	*Psychromonas*	Genus level	-0.724035	9	9	5	-0.6836	0.0100	0.0050	0.0110
vOTU13	*Siphoviridae*	*Cellulophaga* phage	Genus level	pOTU47	*Gammaproteobacteria*	*Psychromonas*	Genus level	-0.468271	3	3	11	-0.6340	0.0200	0.0010	0.0043
vOTU16	*Siphoviridae*	*Flavobacterium* phage	Genus level	pOTU47	*Gammaproteobacteria*	*Psychromonas*	Genus level	-0.724035	9	9	5	-0.5936	0.0324	0.0030	0.0082
vOTU24	*Podoviridae*	*Puniceispirillum* phage	Genus level	pOTU47	*Gammaproteobacteria*	*Psychromonas*	Genus level	0.456542	6	6	8	0.5638	0.0448	0.0060	0.0124
vOTU25	*Myoviridae*	*Rhizobium* phage	Genus level	pOTU47	*Gammaproteobacteria*	*Psychromonas*	Genus level	-0.606832	9	9	5	-0.3923	0.1849	0.0290	0.0330
vOTU33	*Myoviridae*	*Yersinia* phage	Genus level	pOTU47	*Gammaproteobacteria*	*Psychromonas*	Genus level	0.538522	3	3	11	0.6887	0.0092	0.0010	0.0043
vOTU35	*Podoviridae*		Cellulophaga phage phi38:1	pOTU47	*Gammaproteobacteria*	*Psychromonas*	Genus level	-0.464262	1	1	13	-0.5748	0.0399	0.0050	0.0110
vOTU37	*Podoviridae*		Pelagibacter phage HTVC010P	pOTU47	*Gammaproteobacteria*	*Psychromonas*	Genus level	0.491598	3	3	11	0.7046	0.0072	0.0090	0.0159
vOTU38	*Podoviridae*		Pelagibacter phage HTVC019P	pOTU47	*Gammaproteobacteria*	*Psychromonas*	Genus level	0.385757	1	1	13	0.5867	0.0351	0.0410	0.0408
vOTU39	*Podoviridae*		Puniceispirillum phage HMO-2011	pOTU47	*Gammaproteobacteria*	*Psychromonas*	Genus level	0.456542	6	6	8	0.5638	0.0448	0.0120	0.0192
vOTU41	*Myoviridae*		Synechococcus phage S-SSM7	pOTU47	*Gammaproteobacteria*	*Psychromonas*	Genus level	-0.291287	3	3	11	-0.4134	0.1603	0.0190	0.0257
vOTU42	*Myoviridae*		Synechococcus phage S-WAM2	pOTU47	*Gammaproteobacteria*	*Psychromonas*	Genus level	0.397921	1	1	13	0.6006	0.0300	0.0260	0.0311
vOTU44	*Myoviridae*		Yersinia phage fHe-Yen9-04	pOTU47	*Gammaproteobacteria*	*Psychromonas*	Genus level	0.538522	3	3	11	0.6887	0.0092	0.0000	0.0000
vOTU03	*Bicaudaviridae*	Family level	Family level	pOTU48	*Gammaproteobacteria*	*Psychrosphaera*	Genus level	0.801401	2	2	6	0.3698	0.2137	0.0010	0.0043
vOTU07	*Myoviridae*	*Escherichia* phage	Genus level	pOTU48	*Gammaproteobacteria*	*Psychrosphaera*	Genus level	0.890498	2	2	12	0.5502	0.0514	0.0480	0.0450
vOTU21	*Siphoviridae*	*Polaribacter* phage	Genus level	pOTU48	*Gammaproteobacteria*	*Psychrosphaera*	Genus level	0.677402	2	2	6	0.3243	0.2796	0.0300	0.0337
vOTU30	*Myoviridae*	*Synechococcus* phage	Genus level	pOTU48	*Gammaproteobacteria*	*Psychrosphaera*	Genus level	-0.901971	2	2	6	-0.3840	0.1952	0.0390	0.0396
vOTU33	*Myoviridae*	*Yersinia* phage	Genus level	pOTU48	*Gammaproteobacteria*	*Psychrosphaera*	Genus level	0.953089	1	1	13	0.7080	0.0068	0.0450	0.0432
vOTU43	*Podoviridae*		Vibrio phage CHOED	pOTU48	*Gammaproteobacteria*	*Psychrosphaera*	Genus level	1.020852	2	2	6	0.4263	0.1464	0.0120	0.0192
vOTU44	*Myoviridae*		Yersinia phage fHe-Yen9-04	pOTU48	*Gammaproteobacteria*	*Psychrosphaera*	Genus level	0.953089	1	1	13	0.7080	0.0068	0.0470	0.0444

vOTU11	*Herelleviridae*	*Bacillus* phage	Genus level	pOTU49	*Actinomycetia*	*Rhodoluna*	Genus level	-0.591176	1	1	13	-0.4413	0.1311	0.0460	0.0438
vOTU12	*Myoviridae*	*Campylobacter* phage	Genus level	pOTU49	*Actinomycetia*	*Rhodoluna*	Genus level	-0.414678	1	1	13	-0.3842	0.1949	0.0240	0.0296
vOTU16	*Siphoviridae*	*Flavobacterium* phage	Genus level	pOTU49	*Actinomycetia*	*Rhodoluna*	Genus level	0.912375	9	9	4	0.3256	0.2776	0.0250	0.0304
vOTU23	*Podoviridae*	*Pseudomonas* phage	Genus level	pOTU49	*Actinomycetia*	*Rhodoluna*	Genus level	-0.389476	1	1	11	-0.3565	0.2319	0.0410	0.0408
vOTU25	*Myoviridae*	*Rhizobium* phage	Genus level	pOTU49	*Actinomycetia*	*Rhodoluna*	Genus level	0.995638	9	9	5	0.7916	0.0013	0.0050	0.0110
vOTU29	*Myoviridae*	*Staphylococcus* phage	Genus level	pOTU49	*Actinomycetia*	*Rhodoluna*	Genus level	0.995638	9	9	5	0.5496	0.0517	0.0040	0.0097
vOTU41	*Myoviridae*		Synechococcus phage S-SSM7	pOTU49	*Actinomycetia*	*Rhodoluna*	Genus level	0.387792	1	1	12	0.4719	0.1035	0.0470	0.0444
vOTU43	*Podoviridae*		Vibrio phage CHOED	pOTU49	*Actinomycetia*	*Rhodoluna*	Genus level	-0.569745	2	2	12	-0.3912	0.1863	0.0330	0.0358
vOTU09	*Myoviridae*	*Aeromonas* phage	Genus level	pOTU50	*Gammaproteobacteria*	*Sedimenticola*	Genus level	0.557635	1	1	13	0.7189	0.0056	0.0360	0.0378
vOTU10	*Myoviridae*	*Agrobacterium* phage	Genus level	pOTU50	*Gammaproteobacteria*	*Sedimenticola*	Genus level	-0.9307	9	9	5	-0.5583	0.0474	0.0210	0.0272
vOTU11	*Herelleviridae*	*Bacillus* phage	Genus level	pOTU50	*Gammaproteobacteria*	*Sedimenticola*	Genus level	0.769484	1	1	12	0.4346	0.1378	0.0010	0.0043
vOTU13	*Siphoviridae*	*Cellulophaga* phage	Genus level	pOTU50	*Gammaproteobacteria*	*Sedimenticola*	Genus level	-0.671203	1	1	13	-0.5113	0.0741	0.0110	0.0182
vOTU16	*Siphoviridae*	*Flavobacterium* phage	Genus level	pOTU50	*Gammaproteobacteria*	*Sedimenticola*	Genus level	-1.077879	9	9	5	-0.5076	0.0766	0.0040	0.0097
vOTU22	*Myoviridae*	*Prochlorococcus* phage	Genus level	pOTU50	*Gammaproteobacteria*	*Sedimenticola*	Genus level	-0.714257	1	1	13	-0.7163	0.0059	0.0140	0.0211
vOTU24	*Podoviridae*	*Puniceispirillum* phage	Genus level	pOTU50	*Gammaproteobacteria*	*Sedimenticola*	Genus level	0.775803	1	1	13	0.6664	0.0129	0.0010	0.0043
vOTU25	*Myoviridae*	*Rhizobium* phage	Genus level	pOTU50	*Gammaproteobacteria*	*Sedimenticola*	Genus level	-0.963812	9	9	5	-0.3597	0.2273	0.0100	0.0171
vOTU30	*Myoviridae*	*Synechococcus* phage	Genus level	pOTU50	*Gammaproteobacteria*	*Sedimenticola*	Genus level	-0.665174	2	2	12	-0.3200	0.2864	0.0060	0.0124
vOTU33	*Myoviridae*	*Yersinia* phage	Genus level	pOTU50	*Gammaproteobacteria*	*Sedimenticola*	Genus level	0.755147	1	1	13	0.5419	0.0558	0.0030	0.0082
vOTU34	*Myoviridae*		Aeromonas virus 65	pOTU50	*Gammaproteobacteria*	*Sedimenticola*	Genus level	0.407671	1	1	13	0.7708	0.0020	0.0480	0.0450
vOTU39	*Podoviridae*		Puniceispirillum phage HMO-2011	pOTU50	*Gammaproteobacteria*	*Sedimenticola*	Genus level	0.775803	1	1	13	0.6664	0.0129	0.0020	0.0066
vOTU41	*Myoviridae*		Synechococcus phage S-SSM7	pOTU50	*Gammaproteobacteria*	*Sedimenticola*	Genus level	-0.510003	1	1	13	-0.6927	0.0087	0.0010	0.0043
vOTU44	*Myoviridae*		Yersinia phage fHe-Yen9-04	pOTU50	*Gammaproteobacteria*	*Sedimenticola*	Genus level	0.755147	1	1	13	0.5419	0.0558	0.0010	0.0043
vOTU04	*Herelleviridae*	Family level	Family level	pOTU51	*Gammaproteobacteria*	*Shewanella*	Genus level	0.951906	1	1	13	0.5616	0.0458	0.0180	0.0250
vOTU07	*Myoviridae*	*Escherichia* phage	Genus level	pOTU51	*Gammaproteobacteria*	*Shewanella*	Genus level	1.012057	2	2	12	0.7147	0.0060	0.0030	0.0082
vOTU12	*Myoviridae*	*Campylobacter* phage	Genus level	pOTU51	*Gammaproteobacteria*	*Shewanella*	Genus level	0.63088	1	1	13	0.3220	0.2832	0.0130	0.0202
vOTU17	*Siphoviridae*	*Lactobacillus* phage	Genus level	pOTU51	*Gammaproteobacteria*	*Shewanella*	Genus level	1.985064	2	2	12	0.9864	0.0000	0.0160	0.0231

vOTU23	*Podoviridae*	*Pseudomonas* phage	Genus level	pOTU51	*Gammaproteobacteria*	*Shewanella*	Genus level	0.642718	1	1	5	0.6982	0.0079	0.0110	0.0182
vOTU31	*Myoviridae*	*Thermus* phage	Genus level	pOTU51	*Gammaproteobacteria*	*Shewanella*	Genus level	2.28989	1	1	13	0.6043	0.0287	0.0260	0.0311
vOTU10	*Myoviridae*	*Agrobacterium* phage	Genus level	pOTU52	*Gammaproteobacteria*	*Sinobacterium*	Genus level	-0.957492	9	9	5	-0.4930	0.0870	0.0180	0.0250
vOTU11	*Herelleviridae*	*Bacillus* phage	Genus level	pOTU52	*Gammaproteobacteria*	*Sinobacterium*	Genus level	0.672108	1	1	11	0.3883	0.1898	0.0070	0.0137
vOTU13	*Siphoviridae*	*Cellulophaga* phage	Genus level	pOTU52	*Gammaproteobacteria*	*Sinobacterium*	Genus level	-0.593402	1	1	13	-0.4267	0.1459	0.0210	0.0272
vOTU16	*Siphoviridae*	*Flavobacterium* phage	Genus level	pOTU52	*Gammaproteobacteria*	*Sinobacterium*	Genus level	-0.987887	9	9	5	-0.4328	0.1397	0.0040	0.0097
vOTU22	*Myoviridae*	*Prochlorococcus* phage	Genus level	pOTU52	*Gammaproteobacteria*	*Sinobacterium*	Genus level	-0.718411	1	1	13	-0.7120	0.0063	0.0080	0.0148
vOTU24	*Podoviridae*	*Puniceispirillum* phage	Genus level	pOTU52	*Gammaproteobacteria*	*Sinobacterium*	Genus level	0.714939	1	1	13	0.5991	0.0305	0.0020	0.0066
vOTU26	*Siphoviridae*	*Roseobacter* phage	Genus level	pOTU52	*Gammaproteobacteria*	*Sinobacterium*	Genus level	0.625723	1	1	13	0.6081	0.0274	0.0350	0.0372
vOTU33	*Myoviridae*	*Yersinia* phage	Genus level	pOTU52	*Gammaproteobacteria*	*Sinobacterium*	Genus level	0.720004	1	1	13	0.5157	0.0712	0.0010	0.0043
vOTU39	*Podoviridae*		Puniceispirillum phage HMO-2011	pOTU52	*Gammaproteobacteria*	*Sinobacterium*	Genus level	0.714939	1	1	13	0.5991	0.0305	0.0050	0.0110
vOTU41	*Myoviridae*		Synechococcus phage S-SSM7	pOTU52	*Gammaproteobacteria*	*Sinobacterium*	Genus level	-0.484162	1	1	13	-0.7016	0.0075	0.0040	0.0097
vOTU44	*Myoviridae*		Yersinia phage fHe-Yen9-04	pOTU52	*Gammaproteobacteria*	*Sinobacterium*	Genus level	0.720004	1	1	13	0.5157	0.0712	0.0010	0.0043
vOTU15	*Myoviridae*	*Croceibacter* phage	Genus level	pOTU53	*Gammaproteobacteria*	*Sphingomonas*	Genus level	0.93203	4	4	10	0.6397	0.0185	0.0320	0.0351
vOTU27	*Ackermannviridae*	*Serratia* phage	Genus level	pOTU53	*Gammaproteobacteria*	*Sphingomonas*	Genus level	-1.050718	6	6	7	-0.5530	0.0500	0.0060	0.0124
vOTU35	*Podoviridae*		Cellulophaga phage phi38:1	pOTU53	*Gammaproteobacteria*	*Sphingomonas*	Genus level	-0.625033	1	1	12	-0.4555	0.1178	0.0200	0.0265
vOTU10	*Myoviridae*	*Agrobacterium* phage	Genus level	pOTU54	*Alphaproteobacteria*	*Sulfitobacter*	Genus level	1.085026	9	9	5	0.8842	0.0001	0.0030	0.0082
vOTU15	*Myoviridae*	*Croceibacter* phage	Genus level	pOTU54	*Alphaproteobacteria*	*Sulfitobacter*	Genus level	-0.915394	3	3	11	-0.6617	0.0138	0.0360	0.0378
vOTU16	*Siphoviridae*	*Flavobacterium* phage	Genus level	pOTU54	*Alphaproteobacteria*	*Sulfitobacter*	Genus level	0.974244	9	9	5	0.6392	0.0187	0.0120	0.0192
vOTU22	*Myoviridae*	*Prochlorococcus* phage	Genus level	pOTU54	*Alphaproteobacteria*	*Sulfitobacter*	Genus level	0.836798	1	1	13	0.7197	0.0055	0.0000	0.0000
vOTU25	*Myoviridae*	*Rhizobium* phage	Genus level	pOTU54	*Alphaproteobacteria*	*Sulfitobacter*	Genus level	0.955746	9	9	5	0.4499	0.1230	0.0120	0.0192
vOTU27	*Ackermannviridae*	*Serratia* phage	Genus level	pOTU54	*Alphaproteobacteria*	*Sulfitobacter*	Genus level	0.895141	9	9	5	0.5285	0.0633	0.0400	0.0402
vOTU32	*Myoviridae*	*Vibrio* phage	Genus level	pOTU54	*Alphaproteobacteria*	*Sulfitobacter*	Genus level	-0.582539	3	3	11	-0.5453	0.0539	0.0370	0.0384
vOTU33	*Myoviridae*	*Yersinia* phage	Genus level	pOTU54	*Alphaproteobacteria*	*Sulfitobacter*	Genus level	-0.567907	1	1	13	-0.5403	0.0566	0.0450	0.0432
vOTU38	*Podoviridae*		Pelagibacter phage HTVC019P	pOTU54	*Alphaproteobacteria*	*Sulfitobacter*	Genus level	-0.655036	2	2	12	-0.6695	0.0123	0.0080	0.0148
vOTU40	*Myoviridae*		Sphingomonas phage PAU	pOTU54	*Alphaproteobacteria*	*Sulfitobacter*	Genus level	-0.574669	1	1	13	-0.6749	0.0114	0.0400	0.0402

vOTU41	*Myoviridae*		Synechococcus phage S-SSM7	pOTU54	*Alphaproteobacteria*	*Sulfitobacter*	Genus level	0.559445	1	1	13	0.8161	0.0007	0.0000	0.0000
vOTU06	*Microviridae*	Family level	Family level	pOTU55	*Gammaproteobacteria*	*Thiohalobacter*	Genus level	0.917196	3	3	11	0.4506	0.1223	0.0300	0.0337
vOTU19	*Siphoviridae*	*Nonlabens* phage	Genus level	pOTU55	*Gammaproteobacteria*	*Thiohalobacter*	Genus level	0.637966	3	3	11	0.5250	0.0654	0.0240	0.0296
vOTU22	*Myoviridae*	*Prochlorococcus* phage	Genus level	pOTU55	*Gammaproteobacteria*	*Thiohalobacter*	Genus level	-0.646347	1	1	12	-0.5421	0.0556	0.0280	0.0324
vOTU41	*Myoviridae*		Synechococcus phage S-SSM7	pOTU55	*Gammaproteobacteria*	*Thiohalobacter*	Genus level	-0.416141	1	1	12	-0.5352	0.0595	0.0210	0.0272
vOTU09	*Myoviridae*	*Aeromonas* phage	Genus level	pOTU56	*Gammaproteobacteria*	*Thioprofundum*	Genus level	0.493082	1	1	13	0.5920	0.0331	0.0090	0.0159
vOTU10	*Myoviridae*	*Agrobacterium* phage	Genus level	pOTU56	*Gammaproteobacteria*	*Thioprofundum*	Genus level	-0.853493	9	9	5	-0.8618	0.0002	0.0010	0.0043
vOTU13	*Siphoviridae*	*Cellulophaga* phage	Genus level	pOTU56	*Gammaproteobacteria*	*Thioprofundum*	Genus level	-0.529856	1	1	13	-0.7985	0.0011	0.0020	0.0066
vOTU16	*Siphoviridae*	*Flavobacterium* phage	Genus level	pOTU56	*Gammaproteobacteria*	*Thioprofundum*	Genus level	-0.873926	9	9	5	-0.7535	0.0029	0.0010	0.0043
vOTU24	*Podoviridae*	*Puniceispirillum* phage	Genus level	pOTU56	*Gammaproteobacteria*	*Thioprofundum*	Genus level	0.505771	1	1	13	0.8162	0.0007	0.0060	0.0124
vOTU25	*Myoviridae*	*Rhizobium* phage	Genus level	pOTU56	*Gammaproteobacteria*	*Thioprofundum*	Genus level	-0.702092	9	9	5	-0.5023	0.0802	0.0050	0.0110
vOTU29	*Myoviridae*	*Staphylococcus* phage	Genus level	pOTU56	*Gammaproteobacteria*	*Thioprofundum*	Genus level	-0.559651	9	9	5	-0.4677	0.1070	0.0420	0.0413
vOTU33	*Myoviridae*	*Yersinia* phage	Genus level	pOTU56	*Gammaproteobacteria*	*Thioprofundum*	Genus level	0.585293	1	1	13	0.6643	0.0133	0.0010	0.0043
vOTU37	*Podoviridae*		Pelagibacter phage HTVC010P	pOTU56	*Gammaproteobacteria*	*Thioprofundum*	Genus level	0.472783	3	3	11	0.6034	0.0290	0.0270	0.0317
vOTU38	*Podoviridae*		Pelagibacter phage HTVC019P	pOTU56	*Gammaproteobacteria*	*Thioprofundum*	Genus level	0.409417	3	3	11	0.6994	0.0078	0.0470	0.0444
vOTU39	*Podoviridae*		Puniceispirillum phage HMO-2011	pOTU56	*Gammaproteobacteria*	*Thioprofundum*	Genus level	0.505771	1	1	13	0.8162	0.0007	0.0060	0.0124
vOTU41	*Myoviridae*		Synechococcus phage S-SSM7	pOTU56	*Gammaproteobacteria*	*Thioprofundum*	Genus level	-0.324144	1	1	13	-0.6635	0.0134	0.0170	0.0241
vOTU42	*Myoviridae*		Synechococcus phage S-WAM2	pOTU56	*Gammaproteobacteria*	*Thioprofundum*	Genus level	0.42831	2	2	12	0.7267	0.0049	0.0350	0.0372
vOTU44	*Myoviridae*		Yersinia phage fHe-Yen9-04	pOTU56	*Gammaproteobacteria*	*Thioprofundum*	Genus level	0.585293	1	1	13	0.6643	0.0133	0.0000	0.0000
vOTU02	*Ampullaviridae*	Family level	Family level	pOTU57	*Gammaproteobacteria*	*Vibrio*	Genus level	0.802376	8	8	6	0.4711	0.1042	0.0380	0.0390
vOTU05	*Inoviridae*	Family level	Family level	pOTU57	*Gammaproteobacteria*	*Vibrio*	Genus level	0.843781	12	12	2	0.7136	0.0062	0.0230	0.0288
vOTU07	*Myoviridae*	*Escherichia* phage	Genus level	pOTU59	*Flavobacteriia*	*Winogradskyella*	Genus level	0.41332	1	1	13	0.3807	0.1993	0.0230	0.0288
vOTU10	*Myoviridae*	*Agrobacterium* phage	Genus level	pOTU59	*Flavobacteriia*	*Winogradskyella*	Genus level	-0.634682	9	9	5	-0.3749	0.2068	0.0140	0.0211
vOTU15	*Myoviridae*	*Croceibacter* phage	Genus level	pOTU59	*Flavobacteriia*	*Winogradskyella*	Genus level	0.774125	2	2	12	0.6851	0.0098	0.0030	0.0082
vOTU16	*Siphoviridae*	*Flavobacterium* phage	Genus level	pOTU59	*Flavobacteriia*	*Winogradskyella*	Genus level	-0.616866	9	9	5	-0.3259	0.2772	0.0160	0.0231
vOTU35	*Podoviridae*		Cellulophaga phage phi38:1	pOTU59	*Flavobacteriia*	*Winogradskyella*	Genus level	-0.536592	1	1	13	-0.4216	0.1514	0.0000	0.0000

vOTU38	*Podoviridae*		Pelagibacter phage HTVC019P	pOTU59	*Flavobacteriia*	*Winogradskyella*	Genus level	0.43351	1	1	13	0.3685	0.2153	0.0210	0.0272
vOTU41	*Myoviridae*		Synechococcus phage S-SSM7	pOTU59	*Flavobacteriia*	*Winogradskyella*	Genus level	-0.2561	4	4	10	-0.3768	0.2044	0.0400	0.0402
vOTU10	*Myoviridae*	*Agrobacterium* phage	Genus level	pOTU60	*Alphaproteobacteria*	*Sulfitobacter*	*Sulfitobacter profundi*	1.085026	9	9	5	0.8851	0.0001	0.0050	0.0110
vOTU14	*Myoviridae*	*Clostridium* phage	Genus level	pOTU60	*Alphaproteobacteria*	*Sulfitobacter*	*Sulfitobacter profundi*	-0.613728	2	2	12	-0.5725	0.0409	0.0430	0.0419
vOTU15	*Myoviridae*	*Croceibacter* phage	Genus level	pOTU60	*Alphaproteobacteria*	*Sulfitobacter*	*Sulfitobacter profundi*	-0.915394	3	3	11	-0.6657	0.0130	0.0340	0.0365
vOTU16	*Siphoviridae*	*Flavobacterium* phage	Genus level	pOTU60	*Alphaproteobacteria*	*Sulfitobacter*	*Sulfitobacter profundi*	0.974244	9	9	5	0.6405	0.0184	0.0070	0.0137
vOTU22	*Myoviridae*	*Prochlorococcus* phage	Genus level	pOTU60	*Alphaproteobacteria*	*Sulfitobacter*	*Sulfitobacter profundi*	0.836798	1	1	13	0.7188	0.0056	0.0000	0.0000
vOTU25	*Myoviridae*	*Rhizobium* phage	Genus level	pOTU60	*Alphaproteobacteria*	*Sulfitobacter*	*Sulfitobacter profundi*	0.955746	9	9	5	0.4506	0.1223	0.0100	0.0171
vOTU27	*Ackermannviridae*	*Serratia* phage	Genus level	pOTU60	*Alphaproteobacteria*	*Sulfitobacter*	*Sulfitobacter profundi*	0.895141	9	9	5	0.5302	0.0623	0.0430	0.0419
vOTU28	*Myoviridae*	*Sphingomonas* phage	Genus level	pOTU60	*Alphaproteobacteria*	*Sulfitobacter*	*Sulfitobacter profundi*	-0.574669	1	1	13	-0.6769	0.0111	0.0350	0.0372
vOTU32	*Myoviridae*	*Vibrio* phage	Genus level	pOTU60	*Alphaproteobacteria*	*Sulfitobacter*	*Sulfitobacter profundi*	-0.582539	3	3	11	-0.5445	0.0543	0.0430	0.0419
vOTU38	*Podoviridae*		Pelagibacter phage HTVC019P	pOTU60	*Alphaproteobacteria*	*Sulfitobacter*	*Sulfitobacter profundi*	-0.655036	2	2	12	-0.6701	0.0122	0.0180	0.0250
vOTU40	*Myoviridae*		Sphingomonas phage PAU	pOTU60	*Alphaproteobacteria*	*Sulfitobacter*	*Sulfitobacter profundi*	-0.574669	1	1	13	-0.6769	0.0111	0.0380	0.0390
vOTU41	*Myoviridae*		Synechococcus phage S-SSM7	pOTU60	*Alphaproteobacteria*	*Sulfitobacter*	*Sulfitobacter profundi*	0.559445	1	1	13	0.8141	0.0007	0.0000	0.0000
vOTU10	*Myoviridae*	*Agrobacterium* phage	Genus level	pOTU61	*Alphaproteobacteria*	*Loktanella*	*Loktanella acticola*	1.056444	9	9	5	0.7973	0.0011	0.0040	0.0097
vOTU12	*Myoviridae*	*Campylobacter* phage	Genus level	pOTU61	*Alphaproteobacteria*	*Loktanella*	*Loktanella acticola*	-0.374464	1	1	10	-0.4146	0.1590	0.0350	0.0372
vOTU13	*Siphoviridae*	*Cellulophaga* phage	Genus level	pOTU61	*Alphaproteobacteria*	*Loktanella*	*Loktanella acticola*	0.645221	1	1	13	0.8407	0.0003	0.0150	0.0221
vOTU16	*Siphoviridae*	*Flavobacterium* phage	Genus level	pOTU61	*Alphaproteobacteria*	*Loktanella*	*Loktanella acticola*	1.216218	9	9	5	0.9511	0.0000	0.0000	0.0000
vOTU22	*Myoviridae*	*Prochlorococcus* phage	Genus level	pOTU61	*Alphaproteobacteria*	*Loktanella*	*Loktanella acticola*	0.746614	1	1	13	0.3964	0.1800	0.0020	0.0066
vOTU23	*Podoviridae*	*Pseudomonas* phage	Genus level	pOTU61	*Alphaproteobacteria*	*Loktanella*	*Loktanella acticola*	-0.400885	1	1	13	-0.4844	0.0935	0.0380	0.0390
vOTU24	*Podoviridae*	*Puniceispirillum* phage	Genus level	pOTU61	*Alphaproteobacteria*	*Loktanella*	*Loktanella acticola*	-0.729768	1	1	13	-0.8162	0.0007	0.0020	0.0066
vOTU25	*Myoviridae*	*Rhizobium* phage	Genus level	pOTU61	*Alphaproteobacteria*	*Loktanella*	*Loktanella acticola*	0.955065	9	9	5	0.6635	0.0134	0.0130	0.0202
vOTU26	*Siphoviridae*	*Roseobacter* phage	Genus level	pOTU61	*Alphaproteobacteria*	*Loktanella*	*Loktanella acticola*	-0.610936	1	1	11	-0.3707	0.2124	0.0480	0.0450
vOTU28	*Myoviridae*	*Sphingomonas* phage	Genus level	pOTU61	*Alphaproteobacteria*	*Loktanella*	*Loktanella acticola*	-0.609123	1	1	13	-0.6027	0.0292	0.0310	0.0343
vOTU29	*Myoviridae*	*Staphylococcus* phage	Genus level	pOTU61	*Alphaproteobacteria*	*Loktanella*	*Loktanella acticola*	0.84944	9	9	5	0.6840	0.0099	0.0190	0.0257
vOTU30	*Myoviridae*	*Synechococcus* phage	Genus level	pOTU61	*Alphaproteobacteria*	*Loktanella*	*Loktanella acticola*	0.658334	2	2	12	0.6813	0.0104	0.0220	0.0280

vOTU32	*Myoviridae*	*Vibrio* phage	Genus level	pOTU61	*Alphaproteobacteria*	*Loktanella*	*Loktanella acticola*	-0.558227	2	2	12	-0.4465	0.1261	0.0440	0.0425
vOTU33	*Myoviridae*	*Yersinia* phage	Genus level	pOTU61	*Alphaproteobacteria*	*Loktanella*	*Loktanella acticola*	-0.70514	1	1	13	-0.7066	0.0069	0.0050	0.0110
vOTU39	*Podoviridae*		Puniceispirillum phage HMO-2011	pOTU61	*Alphaproteobacteria*	*Loktanella*	*Loktanella acticola*	-0.729768	1	1	13	-0.8162	0.0007	0.0010	0.0043
vOTU40	*Myoviridae*		Sphingomonas phage PAU	pOTU61	*Alphaproteobacteria*	*Loktanella*	*Loktanella acticola*	-0.609123	1	1	13	-0.6027	0.0292	0.0190	0.0257
vOTU41	*Myoviridae*		Synechococcus phage S-SSM7	pOTU61	*Alphaproteobacteria*	*Loktanella*	*Loktanella acticola*	0.524484	1	1	13	0.6740	0.0115	0.0000	0.0000
vOTU42	*Myoviridae*		Synechococcus phage S-WAM2	pOTU61	*Alphaproteobacteria*	*Loktanella*	*Loktanella acticola*	-0.657611	2	2	12	-0.6457	0.0171	0.0070	0.0137
vOTU43	*Podoviridae*		Vibrio phage CHOED	pOTU61	*Alphaproteobacteria*	*Loktanella*	*Loktanella acticola*	-0.591365	2	2	12	-0.5090	0.0756	0.0340	0.0365
vOTU44	*Myoviridae*		Yersinia phage fHe-Yen9-04	pOTU61	*Alphaproteobacteria*	*Loktanella*	*Loktanella acticola*	-0.70514	1	1	13	-0.7066	0.0069	0.0060	0.0124
vOTU10	*Myoviridae*	*Agrobacterium* phage	Genus level	pOTU62	*Gammaproteobacteria*	*Pseudomonas*	*Pseudomonas sabulinigri*	-0.792508	9	9	5	-0.4377	0.1347	0.0050	0.0110
vOTU13	*Siphoviridae*	*Cellulophaga* phage	Genus level	pOTU62	*Gammaproteobacteria*	*Pseudomonas*	*Pseudomonas sabulinigri*	-0.45435	2	2	12	-0.3328	0.2666	0.0200	0.0265
vOTU15	*Myoviridae*	*Croceibacter* phage	Genus level	pOTU62	*Gammaproteobacteria*	*Pseudomonas*	*Pseudomonas sabulinigri*	0.818972	2	2	12	0.6523	0.0157	0.0010	0.0043
vOTU16	*Siphoviridae*	*Flavobacterium* phage	Genus level	pOTU62	*Gammaproteobacteria*	*Pseudomonas*	*Pseudomonas sabulinigri*	-0.828511	9	9	5	-0.3800	0.2003	0.0020	0.0066
vOTU35	*Podoviridae*		Cellulophaga phage phi38:1	pOTU62	*Gammaproteobacteria*	*Pseudomonas*	*Pseudomonas sabulinigri*	-0.510197	1	1	13	-0.4601	0.1137	0.0060	0.0124
vOTU11	*Herelleviridae*	*Bacillus* phage	Genus level	pOTU63	*Gammaproteobacteria*	*Eionea*	*Eionea flava*	0.750628	1	1	11	0.3856	0.1932	0.0020	0.0066
vOTU13	*Siphoviridae*	*Cellulophaga* phage	Genus level	pOTU63	*Gammaproteobacteria*	*Eionea*	*Eionea flava*	-0.5892	1	1	13	-0.4499	0.1229	0.0290	0.0330
vOTU16	*Siphoviridae*	*Flavobacterium* phage	Genus level	pOTU63	*Gammaproteobacteria*	*Eionea*	*Eionea flava*	-0.982157	9	9	5	-0.4635	0.1107	0.0110	0.0182
vOTU22	*Myoviridae*	*Prochlorococcus* phage	Genus level	pOTU63	*Gammaproteobacteria*	*Eionea*	*Eionea flava*	-0.662836	1	1	13	-0.6810	0.0104	0.0180	0.0250
vOTU24	*Podoviridae*	*Puniceispirillum* phage	Genus level	pOTU63	*Gammaproteobacteria*	*Eionea*	*Eionea flava*	0.716302	1	1	13	0.5941	0.0323	0.0040	0.0097
vOTU25	*Myoviridae*	*Rhizobium* phage	Genus level	pOTU63	*Gammaproteobacteria*	*Eionea*	*Eionea flava*	-0.963812	9	9	3	-0.3485	0.2432	0.0100	0.0171
vOTU26	*Siphoviridae*	*Roseobacter* phage	Genus level	pOTU63	*Gammaproteobacteria*	*Eionea*	*Eionea flava*	0.692269	1	1	13	0.6952	0.0083	0.0110	0.0182
vOTU30	*Myoviridae*	*Synechococcus* phage	Genus level	pOTU63	*Gammaproteobacteria*	*Eionea*	*Eionea flava*	-0.670975	2	2	12	-0.3668	0.2176	0.0180	0.0250
vOTU33	*Myoviridae*	*Yersinia* phage	Genus level	pOTU63	*Gammaproteobacteria*	*Eionea*	*Eionea flava*	0.725297	1	1	13	0.4942	0.0860	0.0030	0.0082
vOTU37	*Podoviridae*		Pelagibacter phage HTVC010P	pOTU63	*Gammaproteobacteria*	*Eionea*	*Eionea flava*	0.596744	1	1	11	0.4638	0.1104	0.0480	0.0450
vOTU39	*Podoviridae*		Puniceispirillum phage HMO-2011	pOTU63	*Gammaproteobacteria*	*Eionea*	*Eionea flava*	0.716302	1	1	13	0.5941	0.0323	0.0030	0.0082
vOTU41	*Myoviridae*		Synechococcus phage S-SSM7	pOTU63	*Gammaproteobacteria*	*Eionea*	*Eionea flava*	-0.498861	1	1	13	-0.6965	0.0082	0.0010	0.0043
vOTU44	*Myoviridae*		Yersinia phage fHe-Yen9-04	pOTU63	*Gammaproteobacteria*	*Eionea*	*Eionea flava*	0.725297	1	1	13	0.4942	0.0860	0.0060	0.0124

vOTU07	*Myoviridae*	*Escherichia* phage	Genus level	pOTU64	*Gammaproteobacteria*	*Cognaticolwellia*	*Cognaticolwellia aestuarii*	0.667727	2	2	12	0.6080	0.0275	0.0110	0.0182
vOTU10	*Myoviridae*	*Agrobacterium* phage	Genus level	pOTU64	*Gammaproteobacteria*	*Cognaticolwellia*	*Cognaticolwellia aestuarii*	-1.155045	9	9	5	-0.3219	0.2835	0.0030	0.0082
vOTU11	*Herelleviridae*	*Bacillus* phage	Genus level	pOTU64	*Gammaproteobacteria*	*Cognaticolwellia*	*Cognaticolwellia aestuarii*	0.617517	6	6	7	0.7578	0.0027	0.0430	0.0419
vOTU13	*Siphoviridae*	*Cellulophaga* phage	Genus level	pOTU64	*Gammaproteobacteria*	*Cognaticolwellia*	*Cognaticolwellia aestuarii*	-0.722801	2	2	12	-0.3663	0.2183	0.0060	0.0124
vOTU24	*Podoviridae*	*Puniceispirillum* phage	Genus level	pOTU64	*Gammaproteobacteria*	*Cognaticolwellia*	*Cognaticolwellia aestuarii*	0.747587	6	6	8	0.4532	0.1199	0.0050	0.0110
vOTU27	*Ackermannviridae*	*Serratia* phage	Genus level	pOTU64	*Gammaproteobacteria*	*Cognaticolwellia*	*Cognaticolwellia aestuarii*	-0.972192	9	9	5	0.3830	0.1965	0.0420	0.0413
vOTU30	*Myoviridae*	*Synechococcus* phage	Genus level	pOTU64	*Gammaproteobacteria*	*Cognaticolwellia*	*Cognaticolwellia aestuarii*	-0.67363	5	5	9	-0.4130	0.1607	0.0290	0.0330
vOTU33	*Myoviridae*	*Yersinia* phage	Genus level	pOTU64	*Gammaproteobacteria*	*Cognaticolwellia*	*Cognaticolwellia aestuarii*	0.790261	4	4	10	0.7465	0.0034	0.0020	0.0066
vOTU37	*Podoviridae*		Pelagibacter phage HTVC010P	pOTU64	*Gammaproteobacteria*	*Cognaticolwellia*	*Cognaticolwellia aestuarii*	0.741668	4	4	10	0.7020	0.0075	0.0170	0.0241
vOTU39	*Podoviridae*		Puniceispirillum phage HMO-2011	pOTU64	*Gammaproteobacteria*	*Cognaticolwellia*	*Cognaticolwellia aestuarii*	0.747587	6	6	8	0.4532	0.1199	0.0050	0.0110
vOTU41	*Myoviridae*		Synechococcus phage S-SSM7	pOTU64	*Gammaproteobacteria*	*Cognaticolwellia*	*Cognaticolwellia aestuarii*	-0.54612	4	4	10	-0.4128	0.1609	0.0000	0.0000
vOTU44	*Myoviridae*		Yersinia phage fHe-Yen9-04	pOTU64	*Gammaproteobacteria*	*Cognaticolwellia*	*Cognaticolwellia aestuarii*	0.790261	4	4	10	0.7465	0.0034	0.0040	0.0097
vOTU06	*Microviridae*	Family level	Family level	pOTU65	*Gammaproteobacteria*	*Pseudomonas*	*Pseudomonas stutzeri*	0.611691	2	2	11	0.5358	0.0591	0.0450	0.0432
vOTU10	*Myoviridae*	*Agrobacterium* phage	Genus level	pOTU65	*Gammaproteobacteria*	*Pseudomonas*	*Pseudomonas stutzeri*	-0.724035	9	9	5	-0.4337	0.1387	0.0080	0.0148
vOTU16	*Siphoviridae*	*Flavobacterium* phage	Genus level	pOTU65	*Gammaproteobacteria*	*Pseudomonas*	*Pseudomonas stutzeri*	-0.724035	9	9	5	-0.3766	0.2047	0.0030	0.0082
vOTU19	*Siphoviridae*	*Nonlabens* phage	Genus level	pOTU65	*Gammaproteobacteria*	*Pseudomonas*	*Pseudomonas stutzeri*	0.374296	2	2	11	0.5607	0.0462	0.0380	0.0390
vOTU22	*Myoviridae*	*Prochlorococcus* phage	Genus level	pOTU65	*Gammaproteobacteria*	*Pseudomonas*	*Pseudomonas stutzeri*	-0.442592	1	1	13	-0.3825	0.1971	0.0220	0.0280
vOTU24	*Podoviridae*	*Puniceispirillum* phage	Genus level	pOTU65	*Gammaproteobacteria*	*Pseudomonas*	*Pseudomonas stutzeri*	0.490347	1	1	13	0.4489	0.1239	0.0040	0.0097
vOTU28	*Myoviridae*	*Sphingomonas* phage	Genus level	pOTU65	*Gammaproteobacteria*	*Pseudomonas*	*Pseudomonas stutzeri*	0.430755	1	1	13	0.5712	0.0415	0.0110	0.0182
vOTU30	*Myoviridae*	*Synechococcus* phage	Genus level	pOTU65	*Gammaproteobacteria*	*Pseudomonas*	*Pseudomonas stutzeri*	-0.402245	2	2	12	-0.3285	0.2731	0.0290	0.0330
vOTU32	*Myoviridae*	*Vibrio* phage	Genus level	pOTU65	*Gammaproteobacteria*	*Pseudomonas*	*Pseudomonas stutzeri*	0.388939	2	2	12	0.3627	0.2233	0.0330	0.0358
vOTU39	*Podoviridae*		Puniceispirillum phage HMO-2011	pOTU65	*Gammaproteobacteria*	*Pseudomonas*	*Pseudomonas stutzeri*	0.490347	1	1	13	0.4489	0.1239	0.0020	0.0066
vOTU40	*Myoviridae*		Sphingomonas phage PAU	pOTU65	*Gammaproteobacteria*	*Pseudomonas*	*Pseudomonas stutzeri*	0.430755	1	1	13	0.5712	0.0415	0.0150	0.0221
vOTU41	*Myoviridae*		Synechococcus phage S-SSM7	pOTU65	*Gammaproteobacteria*	*Pseudomonas*	*Pseudomonas stutzeri*	-0.299531	1	1	13	-0.3188	0.2884	0.0090	0.0159
vOTU42	*Myoviridae*		Synechococcus phage S-WAM2	pOTU65	*Gammaproteobacteria*	*Pseudomonas*	*Pseudomonas stutzeri*	0.437984	2	2	12	0.4116	0.1623	0.0020	0.0066
vOTU04	*Herelleviridae*	Family level	Family level	pOTU66	*Gammaproteobacteria*	*Colwellia*	*Colwellia echini*	0.398671	1	1	13	0.4567	0.1167	0.0260	0.0311

vOTU07	*Myoviridae*	*Escherichia* phage	Genus level	pOTU66	*Gammaproteobacteria*	*Colwellia*	*Colwellia echini*	0.467674	2	2	12	0.4618	0.1122	0.0020	0.0066
vOTU10	*Myoviridae*	*Agrobacterium* phage	Genus level	pOTU66	*Gammaproteobacteria*	*Colwellia*	*Colwellia echini*	-0.724035	9	9	5	-0.4035	0.1715	0.0080	0.0148
vOTU13	*Siphoviridae*	*Cellulophaga* phage	Genus level	pOTU66	*Gammaproteobacteria*	*Colwellia*	*Colwellia echini*	-0.471589	1	1	13	-0.3126	0.2983	0.0060	0.0124
vOTU15	*Myoviridae*	*Croceibacter* phage	Genus level	pOTU66	*Gammaproteobacteria*	*Colwellia*	*Colwellia echini*	0.65501	2	2	12	0.6246	0.0225	0.0190	0.0257
vOTU16	*Siphoviridae*	*Flavobacterium* phage	Genus level	pOTU66	*Gammaproteobacteria*	*Colwellia*	*Colwellia echini*	-0.724035	9	9	5	-0.3504	0.2405	0.0080	0.0148
vOTU17	*Siphoviridae*	*Lactobacillus* phage	Genus level	pOTU66	*Gammaproteobacteria*	*Colwellia*	*Colwellia echini*	0.50825	2	2	10	0.4209	0.1520	0.0440	0.0425
vOTU31	*Myoviridae*	*Thermus* phage	Genus level	pOTU66	*Gammaproteobacteria*	*Colwellia*	*Colwellia echini*	0.716752	1	1	10	0.7642	0.0024	0.0070	0.0137
vOTU35	*Podoviridae*		Cellulophaga phage phi38:1	pOTU66	*Gammaproteobacteria*	*Colwellia*	*Colwellia echini*	-0.492368	2	2	12	-0.4619	0.1120	0.0020	0.0066
vOTU41	*Myoviridae*		Synechococcus phage S-SSM7	pOTU66	*Gammaproteobacteria*	*Colwellia*	*Colwellia echini*	-0.274162	1	1	13	-0.3210	0.2848	0.0270	0.0317
vOTU03	*Bicaudaviridae*	Family level	Family level	pOTU67	*Flavobacteriia*	*Lacinutrix*	*Lacinutrix algicola*	0.463947	1	1	13	0.7507	0.0031	0.0160	0.0231
vOTU07	*Myoviridae*	*Escherichia* phage	Genus level	pOTU67	*Flavobacteriia*	*Lacinutrix*	*Lacinutrix algicola*	0.688654	1	1	13	0.5061	0.0776	0.0050	0.0110
vOTU10	*Myoviridae*	*Agrobacterium* phage	Genus level	pOTU67	*Flavobacteriia*	*Lacinutrix*	*Lacinutrix algicola*	-0.987152	9	9	5	-0.5580	0.0475	0.0190	0.0257
vOTU13	*Siphoviridae*	*Cellulophaga* phage	Genus level	pOTU67	*Flavobacteriia*	*Lacinutrix*	*Lacinutrix algicola*	-0.720302	2	2	12	-0.5019	0.0805	0.0030	0.0082
vOTU16	*Siphoviridae*	*Flavobacterium* phage	Genus level	pOTU67	*Flavobacteriia*	*Lacinutrix*	*Lacinutrix algicola*	-1.124066	9	9	5	-0.4956	0.0850	0.0010	0.0043
vOTU21	*Siphoviridae*	*Polaribacter* phage	Genus level	pOTU67	*Flavobacteriia*	*Lacinutrix*	*Lacinutrix algicola*	0.469892	1	1	12	0.7761	0.0018	0.0180	0.0250
vOTU25	*Myoviridae*	*Rhizobium* phage	Genus level	pOTU67	*Flavobacteriia*	*Lacinutrix*	*Lacinutrix algicola*	-0.847523	9	9	5	-0.3323	0.2673	0.0360	0.0378
vOTU30	*Myoviridae*	*Synechococcus* phage	Genus level	pOTU67	*Flavobacteriia*	*Lacinutrix*	*Lacinutrix algicola*	-0.617631	5	5	9	-0.3967	0.1796	0.0260	0.0311
vOTU33	*Myoviridae*	*Yersinia* phage	Genus level	pOTU67	*Flavobacteriia*	*Lacinutrix*	*Lacinutrix algicola*	0.739497	4	4	10	0.4499	0.1230	0.0050	0.0110
vOTU35	*Podoviridae*		Cellulophaga phage phi38:1	pOTU67	*Flavobacteriia*	*Lacinutrix*	*Lacinutrix algicola*	-0.791701	1	1	13	-0.6109	0.0265	0.0010	0.0043
vOTU37	*Podoviridae*		Pelagibacter phage HTVC010P	pOTU67	*Flavobacteriia*	*Lacinutrix*	*Lacinutrix algicola*	0.625886	4	4	10	0.3805	0.1997	0.0350	0.0372
vOTU41	*Myoviridae*		Synechococcus phage S-SSM7	pOTU67	*Flavobacteriia*	*Lacinutrix*	*Lacinutrix algicola*	-0.420025	4	4	10	-0.3624	0.2237	0.0260	0.0311
vOTU42	*Myoviridae*		Synechococcus phage S-WAM2	pOTU67	*Flavobacteriia*	*Lacinutrix*	*Lacinutrix algicola*	0.803237	1	1	13	0.7538	0.0029	0.0000	0.0000
vOTU43	*Podoviridae*		Vibrio phage CHOED	pOTU67	*Flavobacteriia*	*Lacinutrix*	*Lacinutrix algicola*	0.587703	1	1	13	0.5884	0.0344	0.0330	0.0358
vOTU44	*Myoviridae*		Yersinia phage fHe-Yen9-04	pOTU67	*Flavobacteriia*	*Lacinutrix*	*Lacinutrix algicola*	0.739497	4	4	10	0.4499	0.1230	0.0010	0.0043
vOTU04	*Herelleviridae*	Family level	Family level	pOTU68	*Gammaproteobacteria*	*Halomonas*	*Halomonas glaciei*	0.475564	1	1	13	0.5606	0.0463	0.0040	0.0097
vOTU07	*Myoviridae*	*Escherichia* phage	Genus level	pOTU68	*Gammaproteobacteria*	*Halomonas*	*Halomonas glaciei*	0.484995	2	2	12	0.6668	0.0128	0.0020	0.0066

vOTU10	*Myoviridae*	*Agrobacterium* phage	Genus level	pOTU68	*Gammaproteobacteria*	*Halomonas*	*Halomonas glaciei*	-0.724035	9	9	5	-0.3479	0.2441	0.0120	0.0192
vOTU13	*Siphoviridae*	*Cellulophaga* phage	Genus level	pOTU68	*Gammaproteobacteria*	*Halomonas*	*Halomonas glaciei*	-0.510745	1	1	13	-0.3495	0.2418	0.0010	0.0043
vOTU16	*Siphoviridae*	*Flavobacterium* phage	Genus level	pOTU68	*Gammaproteobacteria*	*Halomonas*	*Halomonas glaciei*	-0.724035	9	9	5	-0.3021	0.3158	0.0030	0.0082
vOTU17	*Siphoviridae*	*Lactobacillus* phage	Genus level	pOTU68	*Gammaproteobacteria*	*Halomonas*	*Halomonas glaciei*	0.603226	2	2	10	0.9641	0.0000	0.0120	0.0192
vOTU23	*Podoviridae*	*Pseudomonas* phage	Genus level	pOTU68	*Gammaproteobacteria*	*Halomonas*	*Halomonas glaciei*	0.277317	1	1	13	0.6631	0.0135	0.0390	0.0396
vOTU28	*Myoviridae*	*Sphingomonas* phage	Genus level	pOTU68	*Gammaproteobacteria*	*Halomonas*	*Halomonas glaciei*	0.392194	1	1	13	0.5504	0.0513	0.0250	0.0304
vOTU31	*Myoviridae*	*Thermus* phage	Genus level	pOTU68	*Gammaproteobacteria*	*Halomonas*	*Halomonas glaciei*	0.786197	1	1	10	0.6726	0.0118	0.0000	0.0000
vOTU34	*Myoviridae*		Aeromonas virus 65	pOTU68	*Gammaproteobacteria*	*Halomonas*	*Halomonas glaciei*	0.280246	1	1	13	0.3522	0.2379	0.0340	0.0365
vOTU35	*Podoviridae*		Cellulophaga phage phi38:1	pOTU68	*Gammaproteobacteria*	*Halomonas*	*Halomonas glaciei*	-0.41418	2	2	12	-0.3858	0.1929	0.0310	0.0343
vOTU40	*Myoviridae*		Sphingomonas phage PAU	pOTU68	*Gammaproteobacteria*	*Halomonas*	*Halomonas glaciei*	0.392194	1	1	13	0.5504	0.0513	0.0280	0.0324
vOTU09	*Myoviridae*	*Aeromonas* phage	Genus level	pOTU69	*Gammaproteobacteria*	*Pseudoalteromonas*	*Pseudoalteromonas hodoensis*	0.48208	1	1	13	0.8535	0.0002	0.0030	0.0082
vOTU12	*Myoviridae*	*Campylobacter* phage	Genus level	pOTU69	*Gammaproteobacteria*	*Pseudoalteromonas*	*Pseudoalteromonas hodoensis*	0.261362	1	1	13	0.6508	0.0160	0.0440	0.0425
vOTU22	*Myoviridae*	*Prochlorococcus* phage	Genus level	pOTU69	*Gammaproteobacteria*	*Pseudoalteromonas*	*Pseudoalteromonas hodoensis*	-0.480777	1	1	13	-0.4410	0.1314	0.0070	0.0137
vOTU23	*Podoviridae*	*Pseudomonas* phage	Genus level	pOTU69	*Gammaproteobacteria*	*Pseudoalteromonas*	*Pseudoalteromonas hodoensis*	0.278132	1	1	13	0.4962	0.0846	0.0370	0.0384
vOTU24	*Podoviridae*	*Puniceispirillum* phage	Genus level	pOTU69	*Gammaproteobacteria*	*Pseudoalteromonas*	*Pseudoalteromonas hodoensis*	0.457111	1	1	13	0.3517	0.2386	0.0070	0.0137
vOTU31	*Myoviridae*	*Thermus* phage	Genus level	pOTU69	*Gammaproteobacteria*	*Pseudoalteromonas*	*Pseudoalteromonas hodoensis*	0.625853	1	1	10	0.5078	0.0765	0.0360	0.0378
vOTU39	*Podoviridae*		Puniceispirillum phage HMO-2011	pOTU69	*Gammaproteobacteria*	*Pseudoalteromonas*	*Pseudoalteromonas hodoensis*	0.457111	1	1	13	0.3517	0.2386	0.0090	0.0159
vOTU41	*Myoviridae*		Synechococcus phage S-SSM7	pOTU69	*Gammaproteobacteria*	*Pseudoalteromonas*	*Pseudoalteromonas hodoensis*	-0.319571	1	1	13	-0.3915	0.1859	0.0050	0.0110
vOTU10	*Myoviridae*	*Agrobacterium* phage	Genus level	pOTU70	*Flavobacteriia*	*Polaribacter*	*Polaribacter staleyi*	-1.031272	9	9	5	-0.6507	0.0160	0.0070	0.0137
vOTU13	*Siphoviridae*	*Cellulophaga* phage	Genus level	pOTU70	*Flavobacteriia*	*Polaribacter*	*Polaribacter staleyi*	-0.59034	3	3	11	-0.4914	0.0881	0.0320	0.0351
vOTU15	*Myoviridae*	*Croceibacter* phage	Genus level	pOTU70	*Flavobacteriia*	*Polaribacter*	*Polaribacter staleyi*	0.897843	3	3	11	0.5112	0.0742	0.0450	0.0432
vOTU16	*Siphoviridae*	*Flavobacterium* phage	Genus level	pOTU70	*Flavobacteriia*	*Polaribacter*	*Polaribacter staleyi*	-1.160851	9	9	5	-0.6300	0.0210	0.0000	0.0000
vOTU24	*Podoviridae*	*Puniceispirillum* phage	Genus level	pOTU70	*Flavobacteriia*	*Polaribacter*	*Polaribacter staleyi*	0.630602	6	6	8	0.4409	0.1316	0.0220	0.0280
vOTU25	*Myoviridae*	*Rhizobium* phage	Genus level	pOTU70	*Flavobacteriia*	*Polaribacter*	*Polaribacter staleyi*	-0.889255	9	9	5	-0.3711	0.2119	0.0300	0.0337
vOTU30	*Myoviridae*	*Synechococcus* phage	Genus level	pOTU70	*Flavobacteriia*	*Polaribacter*	*Polaribacter staleyi*	-0.650407	5	5	9	-0.5306	0.0621	0.0250	0.0304
vOTU33	*Myoviridae*	*Yersinia* phage	Genus level	pOTU70	*Flavobacteriia*	*Polaribacter*	*Polaribacter staleyi*	0.712246	3	3	11	0.4701	0.1050	0.0040	0.0097

vOTU35	*Podoviridae*		Cellulophaga phage phi38:1	pOTU70	*Flavobacteriia*	*Polaribacter*	*Polaribacter staleyi*	-0.729171	1	1	13	-0.5765	0.0392	0.0040	0.0097
vOTU37	*Podoviridae*		Pelagibacter phage HTVC010P	pOTU70	*Flavobacteriia*	*Polaribacter*	*Polaribacter staleyi*	0.606497	3	3	11	0.4657	0.1088	0.0480	0.0450
vOTU38	*Podoviridae*		Pelagibacter phage HTVC019P	pOTU70	*Flavobacteriia*	*Polaribacter*	*Polaribacter staleyi*	0.615192	1	1	13	0.6071	0.0278	0.0260	0.0311
vOTU39	*Podoviridae*		Puniceispirillum phage HMO-2011	pOTU70	*Flavobacteriia*	*Polaribacter*	*Polaribacter staleyi*	0.630602	6	6	8	0.4409	0.1316	0.0260	0.0311
vOTU40	*Myoviridae*		Sphingomonas phage PAU	pOTU70	*Flavobacteriia*	*Polaribacter*	*Polaribacter staleyi*	0.543021	3	3	11	0.5306	0.0621	0.0490	0.0456
vOTU41	*Myoviridae*		Synechococcus phage S-SSM7	pOTU70	*Flavobacteriia*	*Polaribacter*	*Polaribacter staleyi*	-0.449512	3	3	11	-0.5314	0.0616	0.0080	0.0148
vOTU42	*Myoviridae*		Synechococcus phage S-WAM2	pOTU70	*Flavobacteriia*	*Polaribacter*	*Polaribacter staleyi*	0.773664	1	1	13	0.7804	0.0016	0.0000	0.0000
vOTU44	*Myoviridae*		Yersinia phage fHe-Yen9-04	pOTU70	*Flavobacteriia*	*Polaribacter*	*Polaribacter staleyi*	0.712246	3	3	11	0.4701	0.1050	0.0050	0.0110
vOTU07	*Myoviridae*	*Escherichia* phage	Genus level	pOTU71	*Alphaproteobacteria*	*Parasphingorhabdus*	*Parasphingorhabdus flavimaris*	0.482652	1	1	13	0.3302	0.2705	0.0040	0.0097
vOTU10	*Myoviridae*	*Agrobacterium* phage	Genus level	pOTU71	*Alphaproteobacteria*	*Parasphingorhabdus*	*Parasphingorhabdus flavimaris*	-0.724035	9	9	5	-0.3889	0.1891	0.0080	0.0148
vOTU15	*Myoviridae*	*Croceibacter* phage	Genus level	pOTU71	*Alphaproteobacteria*	*Parasphingorhabdus*	*Parasphingorhabdus flavimaris*	0.818972	2	2	12	0.7028	0.0074	0.0000	0.0000
vOTU16	*Siphoviridae*	*Flavobacterium* phage	Genus level	pOTU71	*Alphaproteobacteria*	*Parasphingorhabdus*	*Parasphingorhabdus flavimaris*	-0.724035	9	9	5	-0.3377	0.2592	0.0070	0.0137
vOTU28	*Myoviridae*	*Sphingomonas* phage	Genus level	pOTU71	*Alphaproteobacteria*	*Parasphingorhabdus*	*Parasphingorhabdus flavimaris*	0.390382	2	2	12	0.4014	0.1740	0.0430	0.0419
vOTU35	*Podoviridae*		Cellulophaga phage phi38:1	pOTU71	*Alphaproteobacteria*	*Parasphingorhabdus*	*Parasphingorhabdus flavimaris*	-0.527488	1	1	13	-0.4218	0.1511	0.0010	0.0043
vOTU38	*Podoviridae*		Pelagibacter phage HTVC019P	pOTU71	*Alphaproteobacteria*	*Parasphingorhabdus*	*Parasphingorhabdus flavimaris*	0.452761	1	1	13	0.3552	0.2336	0.0150	0.0221
vOTU40	*Myoviridae*		Sphingomonas phage PAU	pOTU71	*Alphaproteobacteria*	*Parasphingorhabdus*	*Parasphingorhabdus flavimaris*	0.390382	2	2	12	0.4014	0.1740	0.0420	0.0413
vOTU07	*Myoviridae*	*Escherichia* phage	Genus level	pOTU72	*Flavobacteriia*	*Polaribacter*	*Polaribacter haliotis*	0.588795	1	1	13	0.3787	0.2020	0.0270	0.0317
vOTU10	*Myoviridae*	*Agrobacterium* phage	Genus level	pOTU72	*Flavobacteriia*	*Polaribacter*	*Polaribacter haliotis*	-1.057073	9	9	5	-0.7031	0.0073	0.0060	0.0124
vOTU13	*Siphoviridae*	*Cellulophaga* phage	Genus level	pOTU72	*Flavobacteriia*	*Polaribacter*	*Polaribacter haliotis*	-0.578167	2	2	12	-0.4760	0.1001	0.0310	0.0343
vOTU15	*Myoviridae*	*Croceibacter* phage	Genus level	pOTU72	*Flavobacteriia*	*Polaribacter*	*Polaribacter haliotis*	0.997831	2	2	12	0.6039	0.0288	0.0150	0.0221
vOTU16	*Siphoviridae*	*Flavobacterium* phage	Genus level	pOTU72	*Flavobacteriia*	*Polaribacter*	*Polaribacter haliotis*	-1.163935	9	9	5	-0.6162	0.0249	0.0000	0.0000
vOTU25	*Myoviridae*	*Rhizobium* phage	Genus level	pOTU72	*Flavobacteriia*	*Polaribacter*	*Polaribacter haliotis*	-0.983894	9	9	5	-0.4042	0.1707	0.0060	0.0124
vOTU28	*Myoviridae*	*Sphingomonas* phage	Genus level	pOTU72	*Flavobacteriia*	*Polaribacter*	*Polaribacter haliotis*	0.627568	2	2	12	0.7220	0.0053	0.0160	0.0231
vOTU29	*Myoviridae*	*Staphylococcus* phage	Genus level	pOTU72	*Flavobacteriia*	*Polaribacter*	*Polaribacter haliotis*	-0.78465	9	9	5	-0.3806	0.1996	0.0490	0.0456
vOTU30	*Myoviridae*	*Synechococcus* phage	Genus level	pOTU72	*Flavobacteriia*	*Polaribacter*	*Polaribacter haliotis*	-0.670692	5	5	9	-0.6474	0.0167	0.0140	0.0211
vOTU33	*Myoviridae*	*Yersinia* phage	Genus level	pOTU72	*Flavobacteriia*	*Polaribacter*	*Polaribacter haliotis*	0.608128	4	4	10	0.3601	0.2268	0.0210	0.0272

vOTU35	*Podoviridae*		Cellulophaga phage phi38:1	pOTU72	*Flavobacteriia*	*Polaribacter*	*Polaribacter haliotis*	-0.747013	1	1	13	-0.5262	0.0647	0.0050	0.0110
vOTU38	*Podoviridae*		Pelagibacter phage HTVC019P	pOTU72	*Flavobacteriia*	*Polaribacter*	*Polaribacter haliotis*	0.564797	1	1	13	0.6472	0.0168	0.0450	0.0432
vOTU39	*Podoviridae*		Puniceispirillum phage HMO-2011	pOTU72	*Flavobacteriia*	*Polaribacter*	*Polaribacter haliotis*	0.564846	6	6	8	0.5621	0.0456	0.0470	0.0444
vOTU40	*Myoviridae*		Sphingomonas phage PAU	pOTU72	*Flavobacteriia*	*Polaribacter*	*Polaribacter haliotis*	0.627568	2	2	12	0.7220	0.0053	0.0230	0.0288
vOTU41	*Myoviridae*		Synechococcus phage S-SSM7	pOTU72	*Flavobacteriia*	*Polaribacter*	*Polaribacter haliotis*	-0.489101	4	4	10	-0.6388	0.0188	0.0030	0.0082
vOTU42	*Myoviridae*		Synechococcus phage S-WAM2	pOTU72	*Flavobacteriia*	*Polaribacter*	*Polaribacter haliotis*	0.740102	1	1	13	0.8291	0.0005	0.0010	0.0043
vOTU44	*Myoviridae*		Yersinia phage fHe-Yen9-04	pOTU72	*Flavobacteriia*	*Polaribacter*	*Polaribacter haliotis*	0.608128	4	4	10	0.3601	0.2268	0.0310	0.0343
vOTU07	*Myoviridae*	*Escherichia* phage	Genus level	pOTU73	*Acidimicrobiia*	*Ilumatobacter*	*Ilumatobacter fluminis*	-0.653827	2	2	12	-0.4311	0.1413	0.0080	0.0148
vOTU08	*Myoviridae*	*Acinetobacter* phage	Genus level	pOTU73	*Acidimicrobiia*	*Ilumatobacter*	*Ilumatobacter fluminis*	-0.458532	2	2	12	-0.4154	0.1581	0.0180	0.0250
vOTU09	*Myoviridae*	*Aeromonas* phage	Genus level	pOTU73	*Acidimicrobiia*	*Ilumatobacter*	*Ilumatobacter fluminis*	-0.607851	1	1	13	-0.4433	0.1292	0.0150	0.0221
vOTU10	*Myoviridae*	*Agrobacterium* phage	Genus level	pOTU73	*Acidimicrobiia*	*Ilumatobacter*	*Ilumatobacter fluminis*	1.145309	9	9	5	0.7064	0.0070	0.0000	0.0000
vOTU13	*Siphoviridae*	*Cellulophaga* phage	Genus level	pOTU73	*Acidimicrobiia*	*Ilumatobacter*	*Ilumatobacter fluminis*	0.722466	1	1	13	0.5641	0.0446	0.0000	0.0000
vOTU16	*Siphoviridae*	*Flavobacterium* phage	Genus level	pOTU73	*Acidimicrobiia*	*Ilumatobacter*	*Ilumatobacter fluminis*	1.154113	9	9	5	0.6873	0.0094	0.0000	0.0000
vOTU22	*Myoviridae*	*Prochlorococcus* phage	Genus level	pOTU73	*Acidimicrobiia*	*Ilumatobacter*	*Ilumatobacter fluminis*	0.730859	1	1	13	0.4177	0.1555	0.0050	0.0110
vOTU23	*Podoviridae*	*Pseudomonas* phage	Genus level	pOTU73	*Acidimicrobiia*	*Ilumatobacter*	*Ilumatobacter fluminis*	-0.430706	1	1	13	-0.3957	0.1808	0.0180	0.0250
vOTU24	*Podoviridae*	*Puniceispirillum* phage	Genus level	pOTU73	*Acidimicrobiia*	*Ilumatobacter*	*Ilumatobacter fluminis*	-0.599858	1	1	13	-0.6348	0.0198	0.0390	0.0396
vOTU28	*Myoviridae*	*Sphingomonas* phage	Genus level	pOTU73	*Acidimicrobiia*	*Ilumatobacter*	*Ilumatobacter fluminis*	-0.775053	1	1	13	-0.5636	0.0449	0.0000	0.0000
vOTU32	*Myoviridae*	*Vibrio* phage	Genus level	pOTU73	*Acidimicrobiia*	*Ilumatobacter*	*Ilumatobacter fluminis*	-0.669141	2	2	12	-0.4283	0.1442	0.0110	0.0182
vOTU33	*Myoviridae*	*Yersinia* phage	Genus level	pOTU73	*Acidimicrobiia*	*Ilumatobacter*	*Ilumatobacter fluminis*	-0.589114	1	1	13	-0.4279	0.1447	0.0330	0.0358
vOTU38	*Podoviridae*		Pelagibacter phage HTVC019P	pOTU73	*Acidimicrobiia*	*Ilumatobacter*	*Ilumatobacter fluminis*	-0.58277	3	3	11	-0.4410	0.1315	0.0310	0.0343
vOTU39	*Podoviridae*		Puniceispirillum phage HMO-2011	pOTU73	*Acidimicrobiia*	*Ilumatobacter*	*Ilumatobacter fluminis*	-0.599858	1	1	13	-0.6348	0.0198	0.0300	0.0337
vOTU40	*Myoviridae*		Sphingomonas phage PAU	pOTU73	*Acidimicrobiia*	*Ilumatobacter*	*Ilumatobacter fluminis*	-0.775053	1	1	13	-0.5636	0.0449	0.0020	0.0066
vOTU41	*Myoviridae*		Synechococcus phage S-SSM7	pOTU73	*Acidimicrobiia*	*Ilumatobacter*	*Ilumatobacter fluminis*	0.432541	1	1	13	0.4282	0.1444	0.0200	0.0265
vOTU42	*Myoviridae*		Synechococcus phage S-WAM2	pOTU73	*Acidimicrobiia*	*Ilumatobacter*	*Ilumatobacter fluminis*	-0.709561	2	2	12	-0.4775	0.0989	0.0030	0.0082
vOTU43	*Podoviridae*		Vibrio phage CHOED	pOTU73	*Acidimicrobiia*	*Ilumatobacter*	*Ilumatobacter fluminis*	-0.586959	2	2	12	-0.3680	0.2160	0.0290	0.0330
vOTU44	*Myoviridae*		Yersinia phage fHe-Yen9-04	pOTU73	*Acidimicrobiia*	*Ilumatobacter*	*Ilumatobacter fluminis*	-0.589114	1	1	13	-0.4279	0.1447	0.0340	0.0365

vOTU07	*Myoviridae*	*Escherichia* phage	Genus level	pOTU74	*Gammaproteobacteria*	*Paraglaciecola*	*Paraglaciecola polaris*	0.479205	2	2	12	0.4948	0.0856	0.0030	0.0082
vOTU09	*Myoviridae*	*Aeromonas* phage	Genus level	pOTU74	*Gammaproteobacteria*	*Paraglaciecola*	*Paraglaciecola polaris*	0.388041	2	2	12	0.3032	0.3140	0.0370	0.0384
vOTU10	*Myoviridae*	*Agrobacterium* phage	Genus level	pOTU74	*Gammaproteobacteria*	*Paraglaciecola*	*Paraglaciecola polaris*	-0.724035	9	9	5	-0.4696	0.1055	0.0080	0.0148
vOTU13	*Siphoviridae*	*Cellulophaga* phage	Genus level	pOTU74	*Gammaproteobacteria*	*Paraglaciecola*	*Paraglaciecola polaris*	-0.547691	2	2	12	-0.5618	0.0457	0.0010	0.0043
vOTU16	*Siphoviridae*	*Flavobacterium* phage	Genus level	pOTU74	*Gammaproteobacteria*	*Paraglaciecola*	*Paraglaciecola polaris*	-0.724035	9	9	5	-0.4077	0.1667	0.0040	0.0097
vOTU33	*Myoviridae*	*Yersinia* phage	Genus level	pOTU74	*Gammaproteobacteria*	*Paraglaciecola*	*Paraglaciecola polaris*	0.489551	4	4	10	0.5018	0.0806	0.0030	0.0082
vOTU35	*Podoviridae*		Cellulophaga phage phi38:1	pOTU74	*Gammaproteobacteria*	*Paraglaciecola*	*Paraglaciecola polaris*	-0.490335	2	2	12	-0.4820	0.0953	0.0020	0.0066
vOTU37	*Podoviridae*		Pelagibacter phage HTVC010P	pOTU74	*Gammaproteobacteria*	*Paraglaciecola*	*Paraglaciecola polaris*	0.439817	4	4	10	0.3805	0.1997	0.0250	0.0304
vOTU42	*Myoviridae*		Synechococcus phage S-WAM2	pOTU74	*Gammaproteobacteria*	*Paraglaciecola*	*Paraglaciecola polaris*	0.423654	2	2	12	0.5660	0.0438	0.0160	0.0231
vOTU44	*Myoviridae*		Yersinia phage fHe-Yen9-04	pOTU74	*Gammaproteobacteria*	*Paraglaciecola*	*Paraglaciecola polaris*	0.489551	4	4	10	0.5018	0.0806	0.0020	0.0066
vOTU09	*Myoviridae*	*Aeromonas* phage	Genus level	pOTU75	*Gammaproteobacteria*	*Sedimenticola*	*Sedimenticola thiotaurini*	0.557635	1	1	13	0.6986	0.0079	0.0320	0.0351
vOTU10	*Myoviridae*	*Agrobacterium* phage	Genus level	pOTU75	*Gammaproteobacteria*	*Sedimenticola*	*Sedimenticola thiotaurini*	-0.9307	9	9	5	-0.5482	0.0524	0.0280	0.0324
vOTU11	*Herelleviridae*	*Bacillus* phage	Genus level	pOTU75	*Gammaproteobacteria*	*Sedimenticola*	*Sedimenticola thiotaurini*	0.769484	1	1	12	0.4347	0.1377	0.0010	0.0043
vOTU13	*Siphoviridae*	*Cellulophaga* phage	Genus level	pOTU75	*Gammaproteobacteria*	*Sedimenticola*	*Sedimenticola thiotaurini*	-0.671203	1	1	13	-0.5070	0.0770	0.0070	0.0137
vOTU16	*Siphoviridae*	*Flavobacterium* phage	Genus level	pOTU75	*Gammaproteobacteria*	*Sedimenticola*	*Sedimenticola thiotaurini*	-1.077879	9	9	5	-0.5022	0.0803	0.0070	0.0137
vOTU22	*Myoviridae*	*Prochlorococcus* phage	Genus level	pOTU75	*Gammaproteobacteria*	*Sedimenticola*	*Sedimenticola thiotaurini*	-0.714257	1	1	13	-0.7030	0.0074	0.0100	0.0171
vOTU24	*Podoviridae*	*Puniceispirillum* phage	Genus level	pOTU75	*Gammaproteobacteria*	*Sedimenticola*	*Sedimenticola thiotaurini*	0.775803	1	1	13	0.6592	0.0142	0.0000	0.0000
vOTU25	*Myoviridae*	*Rhizobium* phage	Genus level	pOTU75	*Gammaproteobacteria*	*Sedimenticola*	*Sedimenticola thiotaurini*	-0.963812	9	9	5	-0.3611	0.2255	0.0130	0.0202
vOTU30	*Myoviridae*	*Synechococcus* phage	Genus level	pOTU75	*Gammaproteobacteria*	*Sedimenticola*	*Sedimenticola thiotaurini*	-0.665174	2	2	12	-0.3224	0.2826	0.0110	0.0182
vOTU33	*Myoviridae*	*Yersinia* phage	Genus level	pOTU75	*Gammaproteobacteria*	*Sedimenticola*	*Sedimenticola thiotaurini*	0.755147	1	1	13	0.5476	0.0527	0.0030	0.0082
vOTU39	*Podoviridae*		Puniceispirillum phage HMO-2011	pOTU75	*Gammaproteobacteria*	*Sedimenticola*	*Sedimenticola thiotaurini*	0.775803	1	1	13	0.6592	0.0142	0.0000	0.0000
vOTU41	*Myoviridae*		Synechococcus phage S-SSM7	pOTU75	*Gammaproteobacteria*	*Sedimenticola*	*Sedimenticola thiotaurini*	-0.510003	1	1	13	-0.6890	0.0092	0.0040	0.0097
vOTU44	*Myoviridae*		Yersinia phage fHe-Yen9-04	pOTU75	*Gammaproteobacteria*	*Sedimenticola*	*Sedimenticola thiotaurini*	0.755147	1	1	13	0.5476	0.0527	0.0030	0.0082
vOTU10	*Myoviridae*	*Agrobacterium* phage	Genus level	pOTU76	*Gammaproteobacteria*	*Sedimenticola*	*Psychrobacter submarinus*	-0.724035	9	9	5	-0.3598	0.2272	0.0080	0.0148
vOTU16	*Siphoviridae*	*Flavobacterium* phage	Genus level	pOTU76	*Gammaproteobacteria*	*Sedimenticola*	*Psychrobacter submarinus*	-0.724035	9	9	5	-0.3124	0.2987	0.0070	0.0137
vOTU24	*Podoviridae*	*Puniceispirillum* phage	Genus level	pOTU76	*Gammaproteobacteria*	*Sedimenticola*	*Psychrobacter submarinus*	0.43483	1	1	13	0.3661	0.2187	0.0300	0.0337

vOTU28	*Myoviridae*	*Sphingomonas* phage	Genus level	pOTU76	*Gammaproteobacteria*	*Sedimenticola*	*Psychrobacter submarinus*	0.400168	1	1	13	0.4848	0.0931	0.0330	0.0358
vOTU39	*Podoviridae*		Puniceispirillum phage HMO-2011	pOTU76	*Gammaproteobacteria*	*Sedimenticola*	*Psychrobacter submarinus*	0.43483	1	1	13	0.3661	0.2187	0.0160	0.0231
vOTU40	*Myoviridae*		Sphingomonas phage PAU	pOTU76	*Gammaproteobacteria*	*Sedimenticola*	*Psychrobacter submarinus*	0.400168	1	1	13	0.4848	0.0931	0.0280	0.0324
vOTU42	*Myoviridae*		Synechococcus phage S-WAM2	pOTU76	*Gammaproteobacteria*	*Sedimenticola*	*Psychrobacter submarinus*	0.436113	2	2	12	0.5081	0.0762	0.0280	0.0324
vOTU09	*Myoviridae*	*Aeromonas* phage	Genus level	pOTU77	*Alphaproteobacteria*	*Amylibacter*	*Amylibacter cionae*	0.664537	1	1	13	0.7685	0.0021	0.0100	0.0171
vOTU10	*Myoviridae*	*Agrobacterium* phage	Genus level	pOTU77	*Alphaproteobacteria*	*Amylibacter*	*Amylibacter cionae*	-0.987443	9	9	5	-0.7541	0.0029	0.0190	0.0257
vOTU11	*Herelleviridae*	*Bacillus* phage	Genus level	pOTU77	*Alphaproteobacteria*	*Amylibacter*	*Amylibacter cionae*	0.685954	1	1	12	0.4961	0.0847	0.0100	0.0171
vOTU13	*Siphoviridae*	*Cellulophaga* phage	Genus level	pOTU77	*Alphaproteobacteria*	*Amylibacter*	*Amylibacter cionae*	-0.656453	1	1	13	-0.7063	0.0070	0.0140	0.0211
vOTU16	*Siphoviridae*	*Flavobacterium* phage	Genus level	pOTU77	*Alphaproteobacteria*	*Amylibacter*	*Amylibacter cionae*	-0.991608	9	9	5	-0.6478	0.0167	0.0130	0.0202
vOTU22	*Myoviridae*	*Prochlorococcus* phage	Genus level	pOTU77	*Alphaproteobacteria*	*Amylibacter*	*Amylibacter cionae*	-0.794974	1	1	13	-0.7460	0.0034	0.0020	0.0066
vOTU24	*Podoviridae*	*Puniceispirillum* phage	Genus level	pOTU77	*Alphaproteobacteria*	*Amylibacter*	*Amylibacter cionae*	0.693907	1	1	13	0.8080	0.0008	0.0110	0.0182
vOTU25	*Myoviridae*	*Rhizobium* phage	Genus level	pOTU77	*Alphaproteobacteria*	*Amylibacter*	*Amylibacter cionae*	-1.010935	9	9	5	-0.4659	0.1086	0.0050	0.0110
vOTU33	*Myoviridae*	*Yersinia* phage	Genus level	pOTU77	*Alphaproteobacteria*	*Amylibacter*	*Amylibacter cionae*	0.655506	1	1	13	0.6057	0.0282	0.0160	0.0231
vOTU34	*Myoviridae*		Aeromonas virus 65	pOTU77	*Alphaproteobacteria*	*Amylibacter*	*Amylibacter cionae*	0.417374	1	1	13	0.7706	0.0021	0.0370	0.0384
vOTU39	*Podoviridae*		Puniceispirillum phage HMO-2011	pOTU77	*Alphaproteobacteria*	*Amylibacter*	*Amylibacter cionae*	0.693907	1	1	13	0.8080	0.0008	0.0110	0.0182
vOTU41	*Myoviridae*		Synechococcus phage S-SSM7	pOTU77	*Alphaproteobacteria*	*Amylibacter*	*Amylibacter cionae*	-0.483043	1	1	13	-0.6742	0.0115	0.0010	0.0043
vOTU44	*Myoviridae*		Yersinia phage fHe-Yen9-04	pOTU77	*Alphaproteobacteria*	*Amylibacter*	*Amylibacter cionae*	0.655506	1	1	13	0.6057	0.0282	0.0160	0.0231
vOTU22	*Myoviridae*	*Prochlorococcus* phage	Genus level	pOTU78	*Gammaproteobacteria*	*Pseudomonas*	*Pseudomonas chloritidismutans*	-1.161843	1	1	7	-0.5126	0.0733	0.0050	0.0110
vOTU24	*Podoviridae*	*Puniceispirillum* phage	Genus level	pOTU78	*Gammaproteobacteria*	*Pseudomonas*	*Pseudomonas chloritidismutans*	0.972415	1	1	13	0.4430	0.1295	0.0460	0.0438
vOTU34	*Myoviridae*		Aeromonas virus 65	pOTU78	*Gammaproteobacteria*	*Pseudomonas*	*Pseudomonas chloritidismutans*	0.689809	1	1	6	0.4060	0.1686	0.0300	0.0337
vOTU39	*Podoviridae*		Puniceispirillum phage HMO-2011	pOTU78	*Gammaproteobacteria*	*Pseudomonas*	*Pseudomonas chloritidismutans*	0.972415	1	1	13	0.4430	0.1295	0.0280	0.0324
vOTU10	*Myoviridae*	*Agrobacterium* phage	Genus level	pOTU79	*Gammaproteobacteria*	*Psychrobacter*	*Psychrobacter nivimaris*	-0.724035	9	9	5	-0.5148	0.0719	0.0090	0.0159
vOTU13	*Siphoviridae*	*Cellulophaga* phage	Genus level	pOTU79	*Gammaproteobacteria*	*Psychrobacter*	*Psychrobacter nivimaris*	-0.416986	1	1	13	-0.3460	0.2468	0.0260	0.0311
vOTU16	*Siphoviridae*	*Flavobacterium* phage	Genus level	pOTU79	*Gammaproteobacteria*	*Psychrobacter*	*Psychrobacter nivimaris*	-0.724035	9	9	5	-0.4470	0.1257	0.0030	0.0082
vOTU23	*Podoviridae*	*Pseudomonas* phage	Genus level	pOTU79	*Gammaproteobacteria*	*Psychrobacter*	*Psychrobacter nivimaris*	0.255983	1	1	13	0.5287	0.0633	0.0440	0.0425
vOTU28	*Myoviridae*	*Sphingomonas* phage	Genus level	pOTU79	*Gammaproteobacteria*	*Psychrobacter*	*Psychrobacter nivimaris*	0.488465	1	1	13	0.7044	0.0072	0.0010	0.0043

vOTU32	*Myoviridae*	*Vibrio* phage	Genus level	pOTU79	*Gammaproteobacteria*	*Psychrobacter*	*Psychrobacter nivimaris*	0.398589	2	2	12	0.6348	0.0198	0.0270	0.0317
vOTU35	*Podoviridae*		Cellulophaga phage phi38:1	pOTU79	*Gammaproteobacteria*	*Psychrobacter*	*Psychrobacter nivimaris*	-0.439551	2	2	12	-0.4980	0.0833	0.0150	0.0221
vOTU36	*Siphoviridae*		Nonlabens phage P12024S	pOTU79	*Gammaproteobacteria*	*Psychrobacter*	*Psychrobacter nivimaris*	0.429465	2	2	11	0.8910	0.0000	0.0120	0.0192
vOTU39	*Podoviridae*		Puniceispirillum phage HMO-2011	pOTU79	*Gammaproteobacteria*	*Psychrobacter*	*Psychrobacter nivimaris*	0.375558	1	1	13	0.3992	0.1766	0.0420	0.0413
vOTU40	*Myoviridae*		Sphingomonas phage PAU	pOTU79	*Gammaproteobacteria*	*Psychrobacter*	*Psychrobacter nivimaris*	0.488465	1	1	13	0.7044	0.0072	0.0040	0.0097
vOTU42	*Myoviridae*		Synechococcus phage S-WAM2	pOTU79	*Gammaproteobacteria*	*Psychrobacter*	*Psychrobacter nivimaris*	0.495675	2	2	12	0.7522	0.0030	0.0010	0.0043
vOTU03	*Bicaudaviridae*	Family level	Family level	pOTU80	*Flavobacteriia*	*Algibacter*	*Algibacter miyuki*	0.26582	2	2	12	0.5634	0.0450	0.0410	0.0408
vOTU10	*Myoviridae*	*Agrobacterium* phage	Genus level	pOTU80	*Flavobacteriia*	*Algibacter*	*Algibacter miyuki*	-0.724035	9	9	5	-0.4669	0.1077	0.0060	0.0124
vOTU15	*Myoviridae*	*Croceibacter* phage	Genus level	pOTU80	*Flavobacteriia*	*Algibacter*	*Algibacter miyuki*	0.664429	2	2	12	0.4910	0.0884	0.0130	0.0202
vOTU16	*Siphoviridae*	*Flavobacterium* phage	Genus level	pOTU80	*Flavobacteriia*	*Algibacter*	*Algibacter miyuki*	-0.724035	9	9	5	-0.4055	0.1693	0.0040	0.0097
vOTU28	*Myoviridae*	*Sphingomonas* phage	Genus level	pOTU80	*Flavobacteriia*	*Algibacter*	*Algibacter miyuki*	0.463869	2	2	12	0.6240	0.0226	0.0020	0.0066
vOTU32	*Myoviridae*	*Vibrio* phage	Genus level	pOTU80	*Flavobacteriia*	*Algibacter*	*Algibacter miyuki*	0.404745	2	2	12	0.7368	0.0041	0.0330	0.0358
vOTU35	*Podoviridae*		Cellulophaga phage phi38:1	pOTU80	*Flavobacteriia*	*Algibacter*	*Algibacter miyuki*	-0.483219	2	2	12	-0.4667	0.1079	0.0040	0.0097
vOTU38	*Podoviridae*		Pelagibacter phage HTVC019P	pOTU80	*Flavobacteriia*	*Algibacter*	*Algibacter miyuki*	0.406082	4	4	10	0.4911	0.0883	0.0190	0.0257
vOTU40	*Myoviridae*		Sphingomonas phage PAU	pOTU80	*Flavobacteriia*	*Algibacter*	*Algibacter miyuki*	0.463869	2	2	12	0.6240	0.0226	0.0090	0.0159
vOTU41	*Myoviridae*		Synechococcus phage S-SSM7	pOTU80	*Flavobacteriia*	*Algibacter*	*Algibacter miyuki*	-0.312363	4	4	10	-0.4586	0.1150	0.0080	0.0148
vOTU42	*Myoviridae*		Synechococcus phage S-WAM2	pOTU80	*Flavobacteriia*	*Algibacter*	*Algibacter miyuki*	0.466966	2	2	12	0.6851	0.0098	0.0080	0.0148
vOTU24	*Podoviridae*	*Puniceispirillum* phage	Genus level	pOTU81	*Flavobacteriia*	*Vicingus*	*Vicingus serpentipes*	0.56953	1	1	13	0.3356	0.2622	0.0490	0.0456
vOTU39	*Podoviridae*		Puniceispirillum phage HMO-2011	pOTU81	*Flavobacteriia*	*Vicingus*	*Vicingus serpentipes*	0.56953	1	1	13	0.3356	0.2622	0.0370	0.0384
vOTU16	*Siphoviridae*	*Flavobacterium* phage	Genus level	pOTU82	*Gammaproteobacteria*	*Porticoccus*	*Porticoccus hydrocarbonoclasticus*	-0.932172	9	9	3	-0.3948	0.1819	0.0140	0.0211
vOTU24	*Podoviridae*	*Puniceispirillum* phage	Genus level	pOTU82	*Gammaproteobacteria*	*Porticoccus*	*Porticoccus hydrocarbonoclasticus*	0.614017	1	1	11	0.4094	0.1648	0.0280	0.0324
vOTU25	*Myoviridae*	*Rhizobium* phage	Genus level	pOTU82	*Gammaproteobacteria*	*Porticoccus*	*Porticoccus hydrocarbonoclasticus*	-0.991527	9	9	3	-0.5236	0.0663	0.0080	0.0148
vOTU29	*Myoviridae*	*Staphylococcus* phage	Genus level	pOTU82	*Gammaproteobacteria*	*Porticoccus*	*Porticoccus hydrocarbonoclasticus*	-0.843781	9	9	3	-0.4659	0.1086	0.0180	0.0250
vOTU33	*Myoviridae*	*Yersinia* phage	Genus level	pOTU82	*Gammaproteobacteria*	*Porticoccus*	*Porticoccus hydrocarbonoclasticus*	0.613063	1	1	11	0.4438	0.1288	0.0310	0.0343
vOTU37	*Podoviridae*		Pelagibacter phage HTVC010P	pOTU82	*Gammaproteobacteria*	*Porticoccus*	*Porticoccus hydrocarbonoclasticus*	0.613465	1	1	11	0.3908	0.1868	0.0470	0.0444
vOTU39	*Podoviridae*		Puniceispirillum phage HMO-2011	pOTU82	*Gammaproteobacteria*	*Porticoccus*	*Porticoccus hydrocarbonoclasticus*	0.614017	1	1	11	0.4094	0.1648	0.0280	0.0324

vOTU41	*Myoviridae*		Synechococcus phage S-SSM7	pOTU82	*Gammaproteobacteria*	*Porticoccus*	*Porticoccus hydrocarbonoclasticus*	-0.384577	1	1	11	-0.4508	0.1221	0.0400	0.0402
vOTU44	*Myoviridae*		Yersinia phage fHe-Yen9-04	pOTU82	*Gammaproteobacteria*	*Porticoccus*	*Porticoccus hydrocarbonoclasticus*	0.613063	1	1	11	0.4438	0.1288	0.0360	0.0378
vOTU07	*Myoviridae*	*Escherichia* phage	Genus level	pOTU83	*Gammaproteobacteria*	*Moraxella*	*Moraxella oblonga*	0.582503	2	2	12	0.3359	0.2618	0.0250	0.0304
vOTU09	*Myoviridae*	*Aeromonas* phage	Genus level	pOTU83	*Gammaproteobacteria*	*Moraxella*	*Moraxella oblonga*	0.659636	1	1	13	0.6617	0.0138	0.0030	0.0082
vOTU10	*Myoviridae*	*Agrobacterium* phage	Genus level	pOTU83	*Gammaproteobacteria*	*Moraxella*	*Moraxella oblonga*	-0.974821	9	9	5	-0.7103	0.0065	0.0110	0.0182
vOTU11	*Herelleviridae*	*Bacillus* phage	Genus level	pOTU83	*Gammaproteobacteria*	*Moraxella*	*Moraxella oblonga*	0.744617	1	1	12	0.4781	0.0984	0.0020	0.0066
vOTU13	*Siphoviridae*	*Cellulophaga* phage	Genus level	pOTU83	*Gammaproteobacteria*	*Moraxella*	*Moraxella oblonga*	-0.742362	1	1	13	-0.6993	0.0078	0.0050	0.0110
vOTU16	*Siphoviridae*	*Flavobacterium* phage	Genus level	pOTU83	*Gammaproteobacteria*	*Moraxella*	*Moraxella oblonga*	-1.114664	9	9	5	-0.6569	0.0147	0.0000	0.0000
vOTU22	*Myoviridae*	*Prochlorococcus* phage	Genus level	pOTU83	*Gammaproteobacteria*	*Moraxella*	*Moraxella oblonga*	-0.705008	1	1	13	-0.6717	0.0119	0.0090	0.0159
vOTU24	*Podoviridae*	*Puniceispirillum* phage	Genus level	pOTU83	*Gammaproteobacteria*	*Moraxella*	*Moraxella oblonga*	0.767256	1	1	13	0.7603	0.0026	0.0000	0.0000
vOTU25	*Myoviridae*	*Rhizobium* phage	Genus level	pOTU83	*Gammaproteobacteria*	*Moraxella*	*Moraxella oblonga*	-1.005543	9	9	5	-0.4734	0.1022	0.0040	0.0097
vOTU29	*Myoviridae*	*Staphylococcus* phage	Genus level	pOTU83	*Gammaproteobacteria*	*Moraxella*	*Moraxella oblonga*	-0.806299	9	9	5	-0.4393	0.1331	0.0430	0.0419
vOTU33	*Myoviridae*	*Yersinia* phage	Genus level	pOTU83	*Gammaproteobacteria*	*Moraxella*	*Moraxella oblonga*	0.768454	1	1	13	0.6052	0.0284	0.0010	0.0043
vOTU34	*Myoviridae*		Aeromonas virus 65	pOTU83	*Gammaproteobacteria*	*Moraxella*	*Moraxella oblonga*	0.427491	1	1	13	0.7003	0.0077	0.0290	0.0330
vOTU39	*Podoviridae*		Puniceispirillum phage HMO-2011	pOTU83	*Gammaproteobacteria*	*Moraxella*	*Moraxella oblonga*	0.767256	1	1	13	0.7603	0.0026	0.0020	0.0066
vOTU41	*Myoviridae*		Synechococcus phage S-SSM7	pOTU83	*Gammaproteobacteria*	*Moraxella*	*Moraxella oblonga*	-0.483812	1	1	13	-0.6468	0.0169	0.0030	0.0082
vOTU44	*Myoviridae*		Yersinia phage fHe-Yen9-04	pOTU83	*Gammaproteobacteria*	*Moraxella*	*Moraxella oblonga*	0.768454	1	1	13	0.6052	0.0284	0.0020	0.0066

Abbreviations: LS, local similarity; SPCC and Pspcc are the Spearman's Correlation Coefficient. Xs and Ys note the day in which the LS correlation begins, and 'Length,' indicates the length of the LS correlation in time.

References

1. Kim, K. E.; Joo, H. M.; Lee, T. K.; Kim, H. J.; Kim, Y. J.; Kim, B. K.; Ha, S. Y.; Jung, S. W. Covariance of Marine Nucleocytoplasmic Large DNA Viruses with Eukaryotic Plankton Communities in the sub-Arctic Kongsfjorden Ecosystem: A Metagenomic Analysis of Marine Microbial Ecosystems. *Microorganisms* **2023**, *11* (1), 169. DOI: 10.3390/microorganisms11010169
2. John, S. G.; Mendez, C. B.; Deng, L.; Poulos, B.; Kauffman, A. K.; Kern, S.; Brum, J.; Polz, M. F.; Boyle, E. A.; Sullivan, M. B. A Simple and Efficient Method for Concentration of Ocean Viruses by Chemical Flocculation. *Environ. Microbiol. Rep.* **2011**, *3* (2), 195–202. DOI: 10.1111/j.1758-2229.2010.00208.x
3. Kim, K. E.; Jung, S. W.; Park, J. S.; Kim, H.-J.; Lee, C.; Ha, S.-Y.; Lee, T.-K. Optimized Metavirome Analysis of Marine DNA Virus Communities for Taxonomic Profiling. *Ocean Sci. J.* **2022**, *57* (2), 259–268. DOI: 10.1007/s12601-022-00064-0
4. Nurk, S.; Meleshko, D.; Korobeynikov, A.; Pevzner, P. A. metaSPAdes: A New Versatile Metagenomic Assembler. *Genome Res.* **2017**, *27* (5), 824–834. DOI: 10.1101/gr.213959.116
5. Nayfach, S.; Camargo, A. P.; Schulz, F.; Eloe-Fadrosh, E.; Roux, S.; Kyrpides, N. C. CheckV Assesses the Quality and Completeness of Metagenome-Assembled Viral Genomes. *Nat. Biotechnol.* **2021**, *39* (5), 578–585. DOI: 10.1038/s41587-020-00774-7
6. Rognes, T.; Flouri, T.; Nichols, B.; Quince, C.; Mahé, F. VSEARCH: A Versatile Open Source Tool for Metagenomics. *PeerJ* **2016**, *4*, e2584. DOI: 10.7717/peerj.2584
7. Roux, S.; Adriaenssens, E. M.; Dutilh, B. E.; Koonin, E. V.; Kropinski, A. M.; Krupovic, M.; Kuhn, J. H.; Lavigne, R.; Brister, J. R.; Varsani, A.; Amid, C.; Aziz, R. K.; Bordenstein, S. R.; Bork, P.; Breitbart, M.; Cochrane, G. R.; Daly, R. A.; Desnues, C.; Duhaime, M. B.; Emerson, J. B.; Enault, F.; Fuhrman, J. A.; Hingamp, P.; Hugenholtz, P.; Hurwitz, B. L.; Ivanova, N. N.; Labonté, J. M.; Lee, K. B.; Malmstrom, R. R.; Martinez-Garcia, M.; Mizrachi, I. K.; Ogata, H.; Páez-Espino, D.; Petit, M. A.; Putonti, C.; Rattei, T.; Reyes, A.; Rodriguez-Valera, F.; Rosario, K.; Schriml, L.; Schulz, F.; Steward, G. F.; Sullivan, M. B.; Sunagawa, S.; Suttle, C. A.; Temperton, B.; Tringe, S. G.; Thurber, R. V.; Webster, N. S.; Whiteson, K. L.; Wilhelm, S. W.; Wommack, K. E.; Woyke, T.; Wrighton, K. C.; Yilmaz, P.; Yoshida, T.; Young, M. J.; Yutin, N.; Allen, L. Z.; Kyrpides, N. C.; Eloe-Fadrosh, E. A. Minimum Information About an Uncultivated Virus Genome (MIUViG). *Nat. Biotechnol.* **2019**, *37* (1), 29–37. DOI: 10.1038/nbt.4306
8. Bushnell, B. *BBMap: A Fast, Accurate, Splice-Aware Aligner **2014**, No. LBNL-7065E*; Ernest Orlando Lawrence Berkeley National Laboratory.
9. Jeong, G.; Kim, H. J.; Kim, K. E.; Kim, Y. J.; Lee, T. K.; Shim, W. J.; Jung, S. W. Selective Attachment of Prokaryotes and Emergence of Potentially Pathogenic Prokaryotes on Four Plastic Surfaces: Adhesion Study in a Natural Marine Environment. *Mar. Pollut. Bull.* **2023**, *193*, 115149. DOI: 10.1016/j.marpolbul.2023.115149
10. Andrews, S. FastQC: A Quality Control Tool for High Throughput Sequence Data. In *Babraham Bioinformatics*. http://www.bioinformatics.babraham.ac.uk/projects/fastqc; Babraham Institute, 2010.
11. Magoč, T.; Salzberg, S. L. FLASH: Fast Length Adjustment of Short Reads to Improve Genome Assemblies. *Bioinformatics* **2011**, *27* (21), 2957–2963. DOI: 10.1093/bioinformatics/btr507
12. Altschul, S. F.; Gish, W.; Miller, W.; Myers, E. W.; Lipman, D. J. Basic Local Alignment Search Tool. *J. Mol. Biol.* **1990**, 215 (3), 403–410. DOI: 10.1016/S0022-2836(05)80360-2
13. Li, R. W.; Connor, E. E.; Li, C.; Baldwin Vi, R. L.; Sparks, M. E. Characterization of the Rumen Microbiota of Pre-ruminant Calves Using Metagenomic Tools. *Environ. Microbiol.* **2012**, *14* (1), 129–139. DOI: 10.1111/j.1462-2920.2011. 02543.x
14. Schloss, P. D.; Westcott, S. L.; Ryabin, T.; Hall, J. R.; Hartmann, M.; Hollister, E. B.; Lesniewski, R. A.; Oakley, B. B.; Parks, D. H.; Robinson, C. J.; Sahl, J. W.; Stres, B.; Thallinger, G. G.; Van Horn, D. J.; Weber, C. F. Introducing Mothur: Open-Source, Platform-Independent, Community-Supported Software for Describing and Comparing Microbial Communities. *Appl. Environ. Microbiol.* **2009**, 75 (23), 7537–7541. DOI: 10.1128/AEM.01541-09
